# Catalytic enantioselective addition of Grignard reagents to aromatic silyl ketimines

**DOI:** 10.1038/ncomms13780

**Published:** 2016-12-23

**Authors:** Jiawei Rong, Juan F. Collados, Pablo Ortiz, Ravindra P. Jumde, Edwin Otten, Syuzanna R. Harutyunyan

**Affiliations:** 1 Stratingh Institute for Chemistry, University of Groningen, Nijenborgh 4, 9747 AG Groningen, The Netherlands

## Abstract

α-Chiral amines are of significant importance in medicinal chemistry, asymmetric synthesis and material science, but methods for their efficient synthesis are scarce. In particular, the synthesis of α-chiral amines with the challenging tetrasubstituted carbon stereocentre is a long-standing problem and catalytic asymmetric additions of organometallic reagents to ketimines that would give direct access to these molecules are underdeveloped. Here we report a highly enantioselective catalytic synthesis of *N*-sulfonyl protected α-chiral silyl amines via the addition of inexpensive, easy to handle and readily available Grignard reagents to silyl ketimines. The key to this success was our ability to suppress any unselective background addition reactions and side reduction pathway, through the identification of an inexpensive, chiral Cu-complex as the catalytically active structure.

## Abstract (web summary)

The stereoselective addition of Grignard reagents to ketimines is potentially a straightforward route to α-chiral amines. Here the authors report a catalytic, asymmetric Grignard addition to silyl ketimines, giving *N*-sulfonyl protected α-chiral silyl amines with tetrasubstituted stereocentres.

α-Chiral amines are ubiquitous building blocks in natural products, pharmacophores, agrochemicals and functional materials^1^. As a result, the production of these compounds is a prominent topic in organic chemistry, with broad industrial applications. One of the most straightforward and atom-efficient approaches for the synthesis of α-chiral amines is the catalytic enantioselective alkylation of imines^2^,^3^,^4^. Tremendous advances have been made in the catalytic asymmetric addition of carbon-based nucleophiles to imines derived from aldehydes (aldimines), affording α-chiral amines with trisubstituted carbon stereocentres^2^,^3^. However, the analogous alkylation reaction of ketimines, which leads to the formation of α-chiral amines with tetrasubstituted carbon stereocentres, has not been solved and remains a long-standing challenge^2^,^3^,^4^.

The main factors responsible for the paucity of methods available for the asymmetric alkylation of ketimines are (i) their sterically more congested structures, (ii) the low reactivity of their C=N bonds (versus C=O in ketones and C=N in aldimines), and (iii) the fact that ketimines, unlike ketones exist as *E* or *Z* isomers. Altogether, these features render the enantiodiscrimination by the chiral catalyst and the reactivity towards the alkylation particularly difficult. Over the past decade, great effort has been put into the development of general protocols, but the main successes have been based on the use of stabilized nucleophiles, such as Strecker and Mannich-type reactions^2^,^3^,^4^. Methods involving non-stabilized nucleophiles remain significantly underdeveloped, with only a few reports to date. The main advances in this area have been achieved in the field of Rh- and Pd-catalysed addition of Csp^2^-nucleophiles to *N*-sulfonyl ketimines using boron reagents^5^,^6^,^7^. However, the addition of Csp^3^-nucleophiles is restricted to methylations and ethylations of a small set of ketimines using organozinc or organoaluminium reagents with Cu, Zr or Rh catalysts^8^,^9^,^10^.

The highly reactive Grignard reagents are probably the most commonly used non-stabilized nucleophiles in both laboratory and industry^11^. Apart from being easy to handle, inexpensive and readily available, these organometallic reagents provide high atom economy due to the transfer of all alkyl groups when compared with organozinc or organoaluminium reagents. Furthermore, if used in the alkylation of ketimines, their high reactivity might be helpful to overcome the low reactivity of the latter. Despite these obvious advantages, and the plethora of methods employing Grignard reagents for catalytic asymmetric C–C bond formation with Michael acceptors^12^,^13^,^14^, aldehydes^15^ and ketones^16^,^17^ not a single catalytic asymmetric reaction involving the direct addition of Grignard reagents to ketimines has been reported. Thus, a general protocol for the catalytic enantioselective addition of Grignard reagents to ketimines would be an important step forward in the synthesis of α-chiral amines with tetrasubstituted carbon stereocentre.

We decided to evaluate the use of Grignard reagents in the Cu-catalysed alkylation of ketimines derived from acylsilanes, in order to access chiral α-silyl amines with tetrasubstituted carbon stereocentre. Organic compounds with one or more carbon atoms replaced by silicon are increasingly popular in medicinal chemistry, because of several attractive characteristics of silicon atoms, such as their similar valency and tetrahedral bonding pattern, lipophilic nature and their low toxicity^18^. For instance, α-silyl amine motifs with trisubstituted carbon stereocentres were recently used in the synthesis of peptide isosteres and are also present in several effective proteolitic enzyme inhibitors^19^,^20^. In contrast, chiral α-silyl amines with tetrasubstituted carbon have not been investigated yet, due to a lack of synthetic methods to access them. So far, only a handful of examples of catalytic asymmetric synthesis of chiral α-silyl amines with trisubstituted carbon stereocentres have been reported^21^,^22^,^23^,^24^. For the formation of α-silyl amines with tetrasubstituted carbon stereocentre, no efficient methods have been developed yet, with two examples of racemic synthesis having been reported until now^25^,^26^. The only report^23^ in which synthesis of chiral α-silyl amines with tetrasubstituted carbon was presented is based on silyl transfer strategy and describes few products with low yields (13–20%) and e.e.'s (60–79%).

Here we report that Grignard reagents are suitable nucleophiles for highly enantioselective addition reactions to silylated ketimines, promoted by a copper-based chiral catalyst. This transformation allows to access wide variety of *N*-protected α-chiral silyl amines with tetrasubstituted carbon stereocentres.

## Results

### Optimization of the reaction conditions

We set out by exploring the addition of *n*-hexylmagnesium bromide to *N*-tosyl silyl ketimines **1**, which were synthesized by condensation of *para-*toluenesulfonamide with the corresponding acylsilanes. It is worth noting that all the ketimines prepared for this work were obtained with >95% *E*-configurational purity (see Supplementary Table 1). Aiming to overcome the low reactivity of the ketimine by use of highly reactive Grignard reagents, we also anticipated several potential problems in this system. The main complication in using Grignard reagents in addition reactions to ketimines is the control over the chemoselectivity: alkyl Grignard reagents with a hydrogen atom at the β-position bear the risk of reducing the ketimine substrate via β-hydride transfer, leading to the formation of undesired reduction side product, namely racemic secondary amine. This chemoselectivity issue can be further enhanced due to the bulkiness introduced by the silyl group, which might impede the formation of the hindered tetrasubstituted chiral carbon, and thus favour β-hydride transfer.

To evaluate our reaction system we first treated *N*-tosyl silyl ketimine (**1ab**) with *n*-hexylmagnesium bromide (2 equiv) in methyl *tert*-butyl ether (MTBE) at –78 °C (Table 1). As expected, the blank reaction led mainly to the reduction product **3ab** (addition:reduction=1:2) via β-hydride transfer (entry 1). To avoid the reduction pathway and at the same time activate the imine towards the addition of Grignard reagents, the optimization studies for the catalytic reaction were carried out in the presence of super-stoichiometric amounts of a mixture of BF_3_·OEt_2_ and CeCl_3_. This Lewis acid assisted synthesis was developed during our previous studies, where we found that a mixture of BF_3_·OEt_2_ and CeCl_3_ prevents the undesired reduction pathway in the synthesis of tertiary alcohols^16^ and that BF_3_·OEt_2_ can activate inherently unreactive substrates such as alkenyl-heteroarenes towards nucleophilic addition of Grignard reagents^14^.

The chiral catalytic systems screened in this study were comprises 5 mol% of CuBr·SMe_2_ and 6 mol% of various chiral ligands. Using chiral ferrocenyl-based diphosphine ligand **L1** the reaction proceeded fairly smoothly, with an addition to reduction ratio of 3:1 and a moderate 31% enantiomeric excess (entry 2). Preliminary optimization of the reaction parameters pointed to MTBE as a superior solvent to toluene, diethyl ether and dichloromethane (entries 2–5). Further ligand screening revealed biphenyl diphosphine-type ligand **L2** and phosphoramidite-type ligand **L3** to be inefficient (entries 6 and 7), while ferrocenyl ligand **L4** outcompeted **L1** both in reactivity and enantioselectivity and **L5** in reactivity (entries 8 and 9).

Next, we tested the steric effect of the silyl group on ketimines **1**. Substituting SiPhMe_2_ (**1ab**) for a bulkier SiPh_2_Me (**1a**) group boosted the enantioselectivity drastically from 37 to 75% (entries 9 and 10). The ketimine with the smaller silyl group SiEt_3_ (**1ac**) afforded the product with an enantioselectivity comparable to that obtained with SiPhMe_2_ (entry 11), while the ketimines **1ad** and **1ae**, with bulky SiPh_3_ and SiPh_2_tBu groups were significantly less reactive (entries 12 and 13).

Subsequently, a comprehensive ligand screening was carried out, customized to *N*-tosyl ketimine **1a**, bearing a SiPh_2_Me moiety. Several ferrocenyl diphosphine ligands (**L5**–**L8**) led to product formation with satisfying yields and enantioselectivities (entries 14-17). The highest enantioselectivity (86%), as well as excellent reactivity and chemoselectivity, were obtained with the ferrocenyl ligand **L8** (entry 17). We were delighted that an excellent addition to reduction ratio (22:1) could be obtained with this chiral ligand without the assistance of Lewis acids (entry 18).

### Effect of the sulfonyl-protecting group

**Figure 1:**
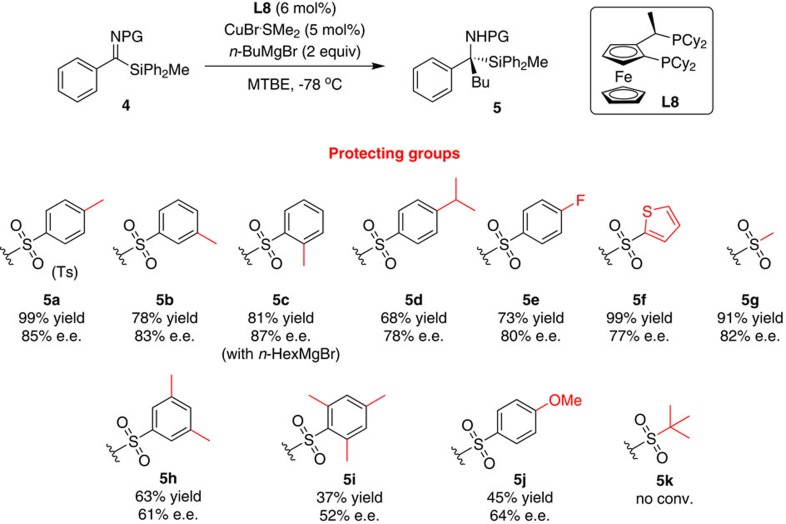
Effect of the sulfonyl-protecting group. Evaluation of sulfonyl-protecting group on the reactivity of the ketimines and the enantioselectivity of the addition reaction.

Following this, we shifted our focus to the effect of the steric and electronic properties of the nitrogen sulfonyl-protecting group on the reactivity of the ketimine and its enantioselectivity in the addition reaction (Fig. 1). Various sulfonyl-protecting groups were evaluated in the addition of *n*-butylmagnesium bromide to ketimines **4** using the CuBr·SMe_2_/**L8** catalytic system. Remarkably, our protocol tolerated a broad range of sulfonyl protecting groups. Aryl sulfonyl-protecting groups with a methyl substituent in *para-*, *meta-* or *ortho-* position of the aromatic group provided the corresponding products (**5a, 5b** and **5c**, respectively) with comparable yields and enantioselectivities. Similarly, ketimines with the bulkier isopropyl or electron-withdrawing fluorine substituents in the *para-*position furnished the products **5d** and **5e**, respectively, with good yields and enantioselectivities. Both selectivity and reactivity were also found to be unabated when the aryl group was replaced by a thiophenyl or a methyl group (products **5f** and **5g**). Increasing the bulkiness of the sulfonyl-protecting group by moving from 4-methyl to 3,5-dimetyl and 2,4,5-trimethyl substituted phenyl groups, led to a gradual decrease in both the reactivity of the ketimine and the enantiopurity of the products (compare **5a**, **5h** and **5i**). A relatively low yield and enantioselectivity were also obtained for the product **5j** derived from the ketimine with the electron donating *para*-methoxy phenyl sulfonyl group. The introduction of a bulky *tert-*butyl sulfonyl-protecting group inhibited the reactivity of the ketimine completely (Fig. 1, product **5k**).

### Ketimine substrate scope

For the evaluation of the substrate scope we chose the reaction between *N*-tosyl silyl ketimines **1** and *n*-hexylmagnesium bromide (*n*-HexMgBr) under optimized reaction conditions, namely 5 mol% of CuBr·SMe_2_, 6 mol% of **L8**, 2 equiv of Grignard reagent, 1–10 h reaction time, –78 °C and MTBE as a solvent (Table 2). The examination of a wide range of ketimines revealed that the reaction is remarkably general. With all examined ketimines we obtained mainly the desired addition product, providing the corresponding α-silyl amines with tetrasubstituted carbon stereocentre with excellent isolated yields, ranging from 77 to 99% and high enantioselectivities ranging from 70 to 94%.

Halogen substituents (F, Cl, Br) in the *meta*- and *para*-positions of the aryl silyl ketimines were tolerated, and the corresponding products (**6b, 6c, 6e–6h**) were obtained with high yields and enantiopurities (entries 2, 3 and 5–8). As expected, *ortho*-substituted aryl silyl ketimines were less reactive. Thus, the assistance of a Lewis acid mixture was required to obtain the corresponding *ortho*-F-substituted aryl silyl amine **6d** in 87% yield and 70% e.e. (entry 4). In the case of *para-* and *meta-*trifluoromethyl substituted aryl silyl ketimines, the corresponding amine products **6i** and **6j** were obtained with high yields (89 and 77%) and e.e.'s (93% and 79%, entries 9 and 10). Ketimines with electron-donating *para*- and *meta*-methyl groups, as well as *para*- and *meta*-methoxy substituents, furnished the corresponding amines **6k–6n** with good yields (72–83%) and enantioselectivities (82-87%) (entries 11–14). The reaction with another ketimine **1o**, bearing a bulky, electron-donating *tert-*butyl group in the *para*-position afforded the corresponding alcohol **6o** with a very good 87% yield and 91% e.e. (entry 15). In addition, high yields and enantioselectivities were obtained for amines **6p–6r** derived from the corresponding *para-* and *meta*-OCF_3_ and 2-naphtyl substituted ketimines **1p–1r** (entries 16–18). Heteroaromatic substituent is also tolerated, as in the case of ketimine **1s**, derived from 2-thiophenyl methyl ketone (entry 19). In order to access aliphatic amines, we attempted the synthesis of alkyl silyl ketimines, but were unable to obtain the corresponding substrates due to the low reactivity of the aliphatic acylsilane precursor. Thus, we decided to test instead aryl alkyl ketimine **1t**, derived from 1-phenylbutan-1-one. A major issue of alkyl substrates, when used in combination with Grignard reagents, is the substrate enolization. Addition of HexMgBr to **1t** using reaction conditions optimized for silyl ketimines furnished the desired product with low yield and nearly racemic. This is not surprising since the geometry of this substrate is very different from that of silyl ketimines. However, our preliminary optimization studies pointed that the enantioselective reaction with enolizable aryl alkyl ketimines is possible (90% yield and 65% e.e.) when a solvent mixture (MTBE/Et_2_O=1/1) and ligand **L1** are used instead of the standard reaction conditions.

All catalytic reaction were analysed after 10 h, to accommodate the reaction times required for the additions to ketimines with electron-donating groups. However, we noted that the reaction times for the additions to ketimines with electron-withdrawing groups were distinctly shorter (1 h to 4 h).

### Grignard reagent scope

To complete the assessment of the scope of the reaction, we turned our attention to the Grignard reagents. A variety of Grignard reagents were examined under the optimized reaction conditions using *meta*-Cl substituted aryl silyl ketimine **1f** as substrate (Table 3). We were delighted to observe that our catalytic system, in contrast to previous reports on alkylations of ketimines^8^,^9^,^10^, was suitable not only for the addition of methyl and ethyl nucleophiles, but that longer alkyl chains were also tolerated. In general, we found that with an increase of the alkyl chain length, the enantioselectivity of the reaction increased as well. The addition of MeMgBr led to the corresponding amine **7a** with excellent yield (92%) and 60% enantioselectivity (entry 1). Importantly, after a single crystallization, the enantiopurity of the amine **7a** (found in mother liquor) was amplified to 96%, indicating a strong difference in solubility between the homo- and heterochiral species.

Increasing the chain length of the Grignard reagents to *Et, n-Bu* and *n*-*Hex* furnished the corresponding products (**7b, 7c** and **6f**) with high yields and enantioselectivities (entries 2–4). Product **7d**, derived from the addition of δ-branched Grignard reagent was obtained with 97% yield and 90% e.e. (entry 5). The presence of an olefinic moiety or a chlorine atom in the Grignard reagents was also tolerated, demonstrating that functionalized amine products **7e**–**7g** can be obtained in high enantiopurity as well (entries 6–8).

In our previous studies on the asymmetric addition of Grignard reagents to ketones, the more sterically hindered *i*-BuMgBr emerged as the optimal reagent in terms of yield and enantioselectivity^17^. Curiously, when we tested this reagent in the current reaction, no conversion of the ketimine substrate was observed, neither towards the addition or the reduction product. Similarly, no conversion was obtained with α-branched Grignard reagents (i-PrMgBr). We attribute this lack of reactivity towards bulky Grignard reagents to steric crowding of the silylated ketimine substrate.

To examine the potential for the scaling up of these reactions, we performed a single experiment on a preparative scale (1 mmol) and were able to obtain the product **6e** with excellent yield (90%) and enantioselectivity (91%), without any erosion from the original values obtained in the small-scale reaction (Table 3, entry 5). The cleavage of the *N*-tosyl protecting group, demonstrated with protected amine **6t**, was achieved using Birch-type reduction. The free amine was obtained with 84% yield (product **8**, see Supplementary information/Supplementary methods). Finally, we could also reduce the catalyst loading (to 1 mol% of CuBr·SMe_2_ and 1.1 mol% of **L8**) and obtain **6f** with only slight decrease of the yield (68%) and enantioselectivity (90%) compared with that obtained with catalyst loading of 5 mol% of CuBr·SMe_2_ and 6 mol% of **L8** (Table 2, entry 6).

## Discussion

We have demonstrated that readily available and cost-efficient Grignard reagents are suitable nucleophiles for the highly enantioselective addition reaction to silyl ketimines, promoted by a copper-based chiral catalyst. This unprecedented transformation allows a highly efficient and enantioselective route to *N*-sulfonyl protected α-chiral silyl amines with tetrasubstituted carbon stereocentre. The feasibility of this approach for the synthesis of amines from enolizable aryl alkyl ketimines has been also demonstrated. This methodology offers an opportunity for future application of newly synthesized enantioenriched α-silyl amines in synthetic and medicinal chemistry. Furthermore, these results could herald a breakthrough in light of the broader paucity of methods for the catalytic enantioselective addition of Grignard reagents to ketimines. Application of the approach developed in this work to various kinds of ketimines is currently in progress.

## Methods

### Copper-catalysed addition of Grignard reagents to silyl ketimines

The general procedure is described for the synthesis of product **6e** via the copper-catalysed alkylation of ketimine **1e** with *n*-HexMgBr. A Schlenk tube equipped with septum and stirring bar was charged with ketimine **1e** (0.1 mmol), CuBr·SMe_2_ (0.005 mmol) and ligand (*S,R*_*Fe*_)-**L8** (0.006 mmol). Dry MTBE (2.5 ml) was added and the solution was stirred under nitrogen at room temperature for 10 min. Then the resulting solution was cooled to –78 °C and stirred for another 15 min. In a separate Schlenk tube, the corresponding Grignard reagent (0.2 mmol, 2M in Et_2_O) was diluted with MTBE (combined volume of 1 ml) under nitrogen and added dropwise to the reaction mixture during 40 min using a syringe pump. Once the addition was complete, the mixture was stirred for 10 h at –78 °C. The reaction was quenched with a saturated aqueous NH_4_Cl solution and the mixture was warmed to room temperature, diluted with dichloromethane and the phases were separated. The aqueous layer was extracted with dichloromethane (3 × 10 ml) and the combined organic layers were dried with anhydrous MgSO_4_, filtered and the solvent was evaporated in *vacuo*. Purification was performed by flash chromatography on silica gel using different mixtures of *n*-pentane:Et_2_O as the eluent and the enantiomeric excess was determined by chiral high-performance liquid chromatography analysis.

### Data availability

The authors declare that the data supporting the findings of this study are available within the article and its Supplementary Information files. For the experimental procedures and spectroscopic and physical data of compounds, see Supplementary Methods. For NMR and high-performance liquid chromatography analysis of the compounds in this article, see Supplementary Figs 1–123. The CCDC 1510318 (**7a**) and CCDC 1510319 (**1g**) contains the supplementary crystallographic data for this paper (Supplementary Table 1). These data can be obtained free of charge from The Cambridge Crystallographic Data Centre via http://www.ccdc.cam.ac.uk/data_request/cif.

## Additional information

**How to cite this article**: Rong, J. *et al*. Catalytic enantioselective addition of Grignard reagents to aromatic silyl ketimines. *Nat. Commun.* **7**, 13780 doi: 10.1038/ncomms13780 (2016).

**Publisher's note:** Springer Nature remains neutral with regard to jurisdictional claims in published maps and institutional affiliations.

## Supplementary Material

Supplementary Information Supplementary Figures, Supplementary Table, Supplementary Discussions, Supplementary Methods, Supplementary References.

## Figures and Tables

**Table 1:**
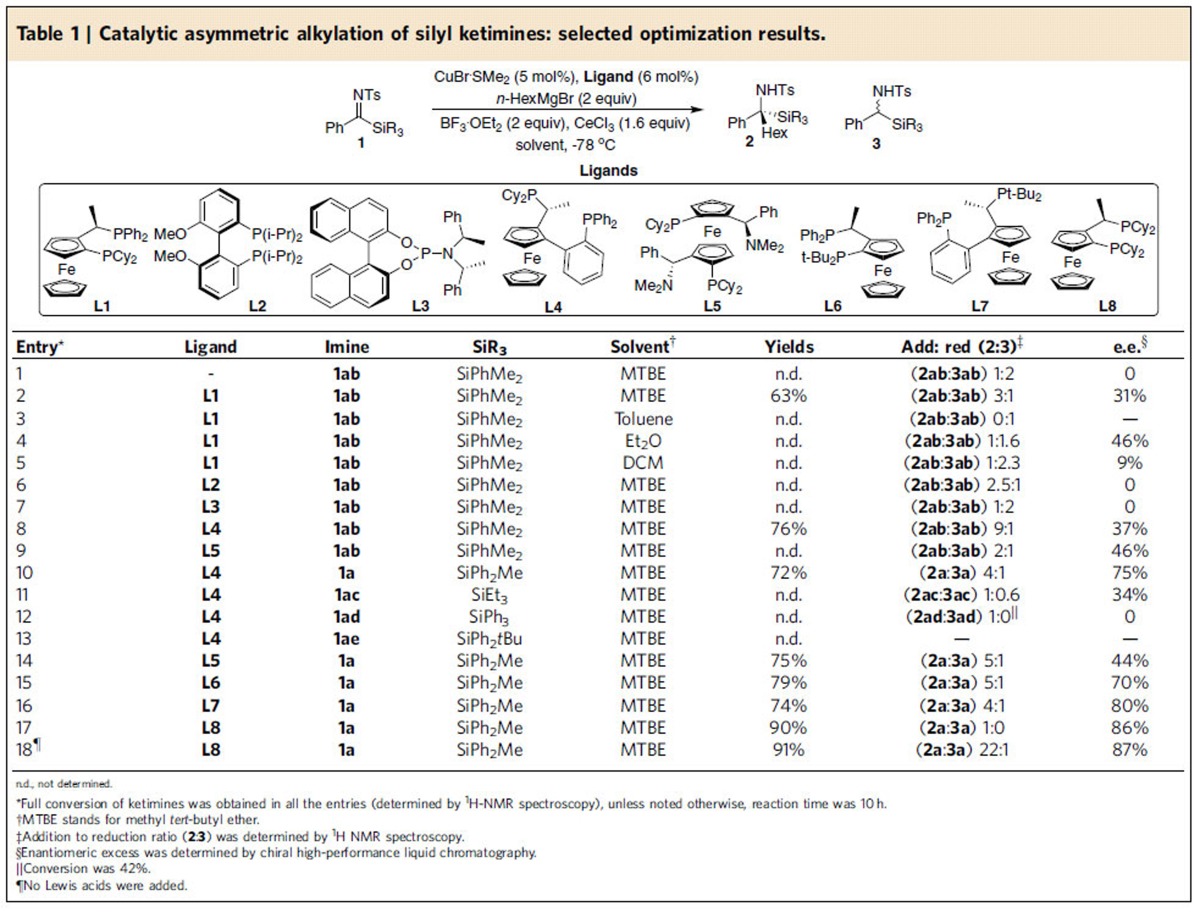
Catalytic asymmetric alkylation of silyl ketimines: selected optimization results.

^n.d., not determined.^

^*^Full conversion of ketimines was obtained in all the entries (determined by ^1^H-NMR spectroscopy), unless noted otherwise, reaction time was 10 h.

^†^MTBE stands for methyl *tert*-butyl ether.

^‡^Addition to reduction ratio (**2**:**3**) was determined by ^1^H NMR spectroscopy.

^§^Enantiomeric excess was determined by chiral high-performance liquid chromatography.

^||^Conversion was 42%.

^¶^No Lewis acids were added.

**Table 2:**
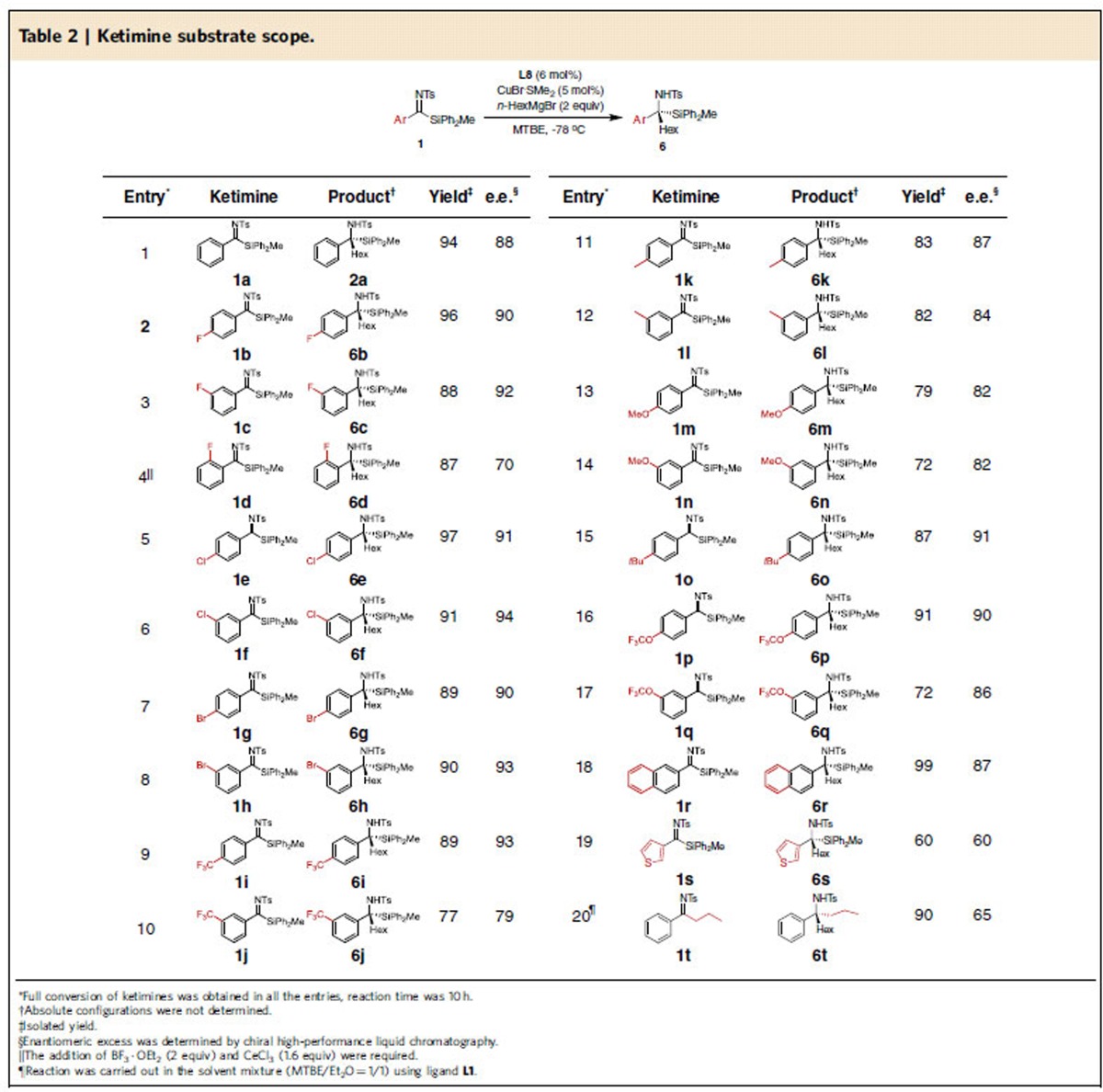
Ketimine substrate scope.

^*^Full conversion of ketimines was obtained in all the entries, reaction time was 10 h.

^†^Absolute configurations were not determined.

^‡^Isolated yield.

^§^Enantiomeric excess was determined by chiral high-performance liquid chromatography.

^||^The addition of BF_3_·OEt_2_ (2 equiv) and CeCl_3_ (1.6 equiv) were required.

^¶^Reaction was carried out in the solvent mixture (MTBE/Et_2_O=1/1) using ligand **L1**.

**Table 3:**
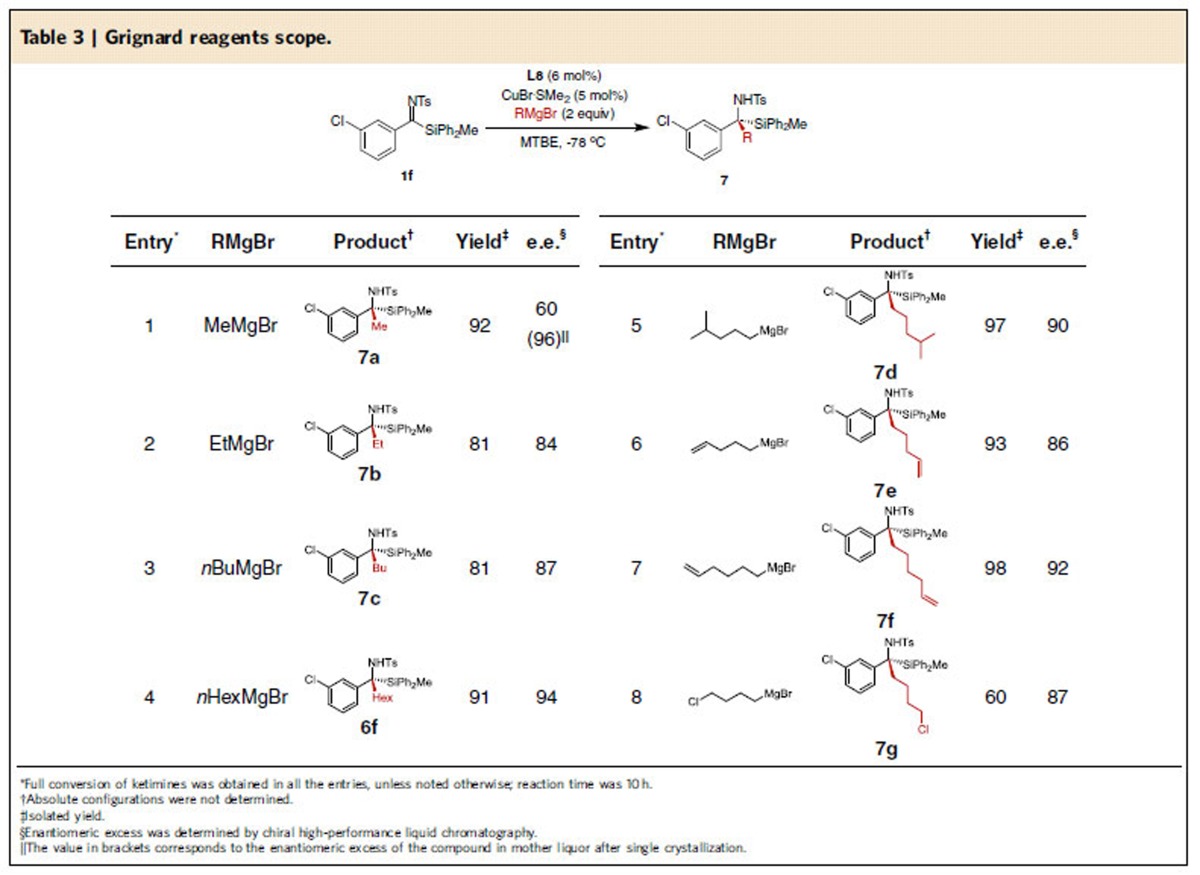
Grignard reagents scope.

^*^Full conversion of ketimines was obtained in all the entries, unless noted otherwise; reaction time was 10 h.

^†^Absolute configurations were not determined.

^‡^Isolated yield.

^§^Enantiomeric excess was determined by chiral high-performance liquid chromatography.

^||^The value in brackets corresponds to the enantiomeric excess of the compound in mother liquor after single crystallization.
