# Supplementary material for: Catalytic enantioselective addition of Grignard reagents to aromatic silyl ketimines
Source: Nat Commun. 2016 Dec 23;7:13780. doi: 10.1038/ncomms13780 (PMC5196062; doi:10.1038/ncomms13780)
Supplement: Supplementary Information — Supplementary Figures, Supplementary Table, Supplementary Discussions, Supplementary Methods, Supplementary References. [file ncomms13780-s1.pdf]

Supplementary Figure 1. 1H, 13C, 19F-NMR spectra of product **9d**.

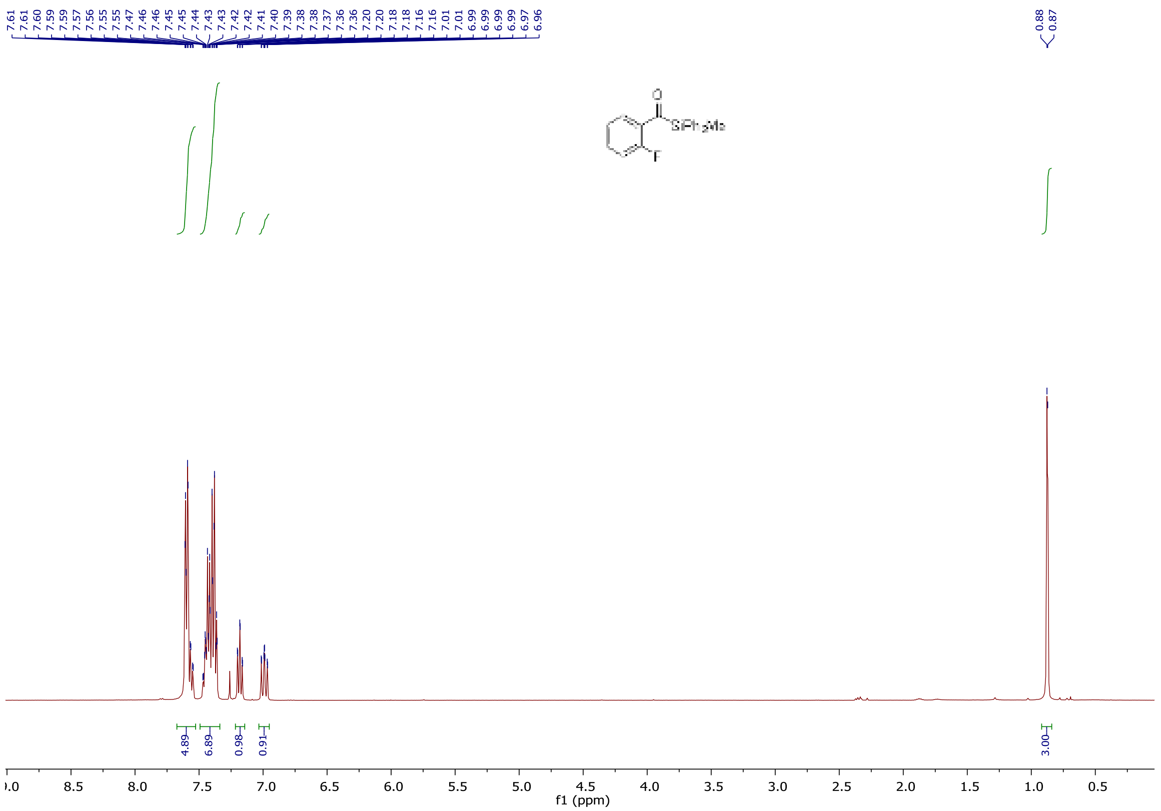

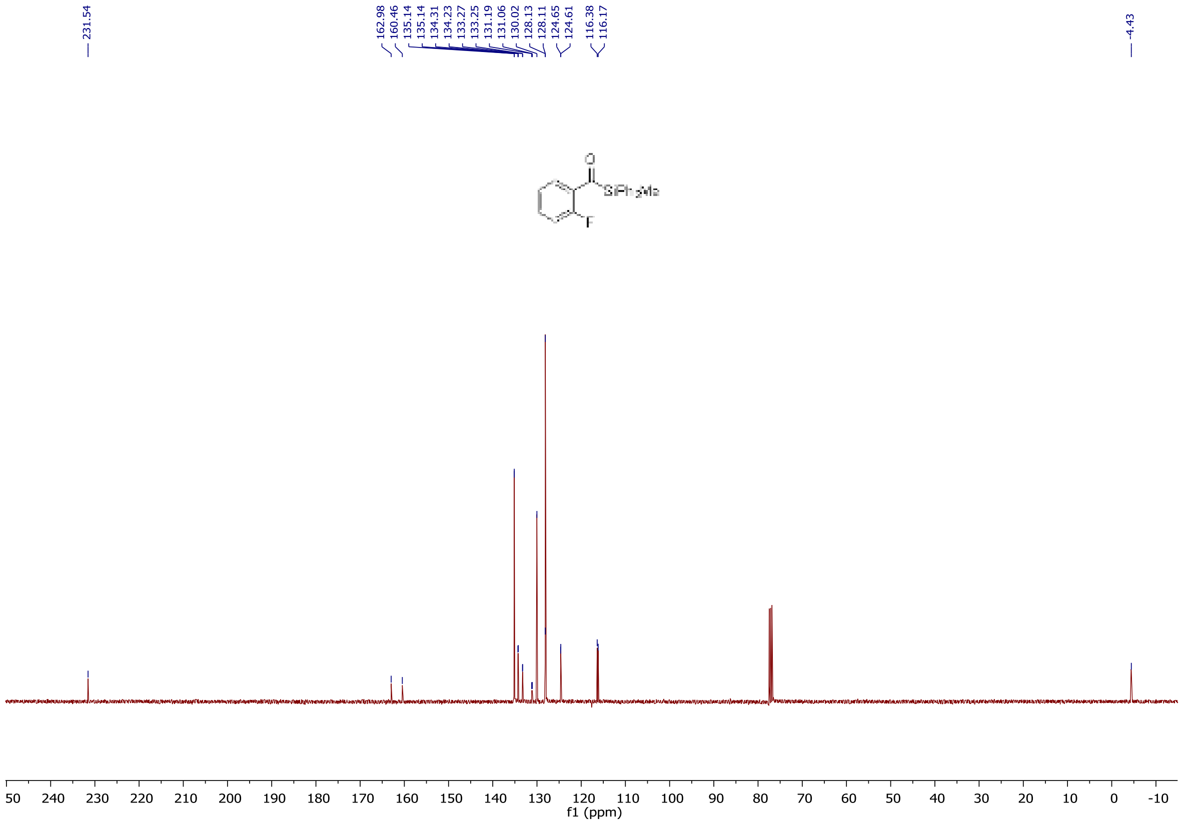

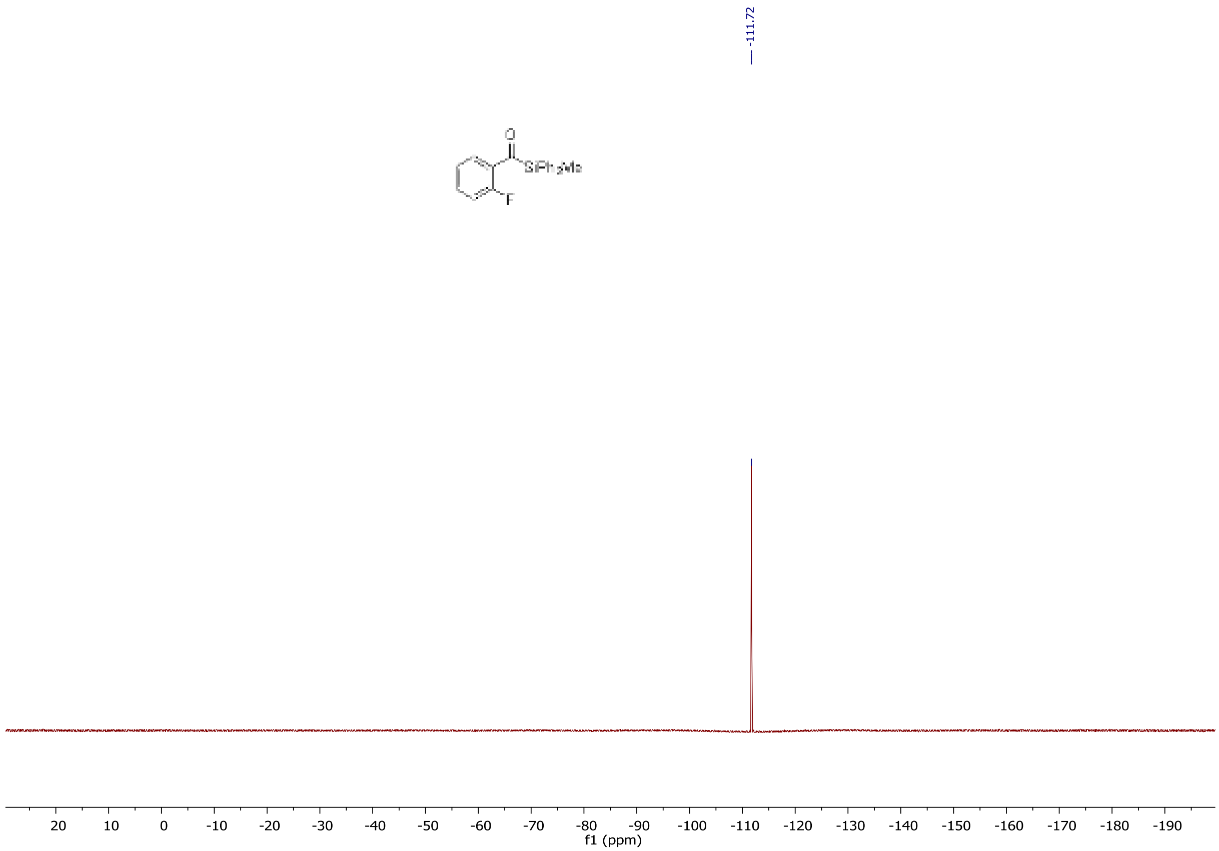
-111.72
O
SiPh2Me
F
20 10 0 -10 -20 -30 -40 -50 -60 -70 -80 -90 -100 -110 -120 -130 -140 -150 -160 -170 -180 -190
f1 (ppm)

Supplementary Figure 2. 1H, 13C-NMR spectra of product **9e**.

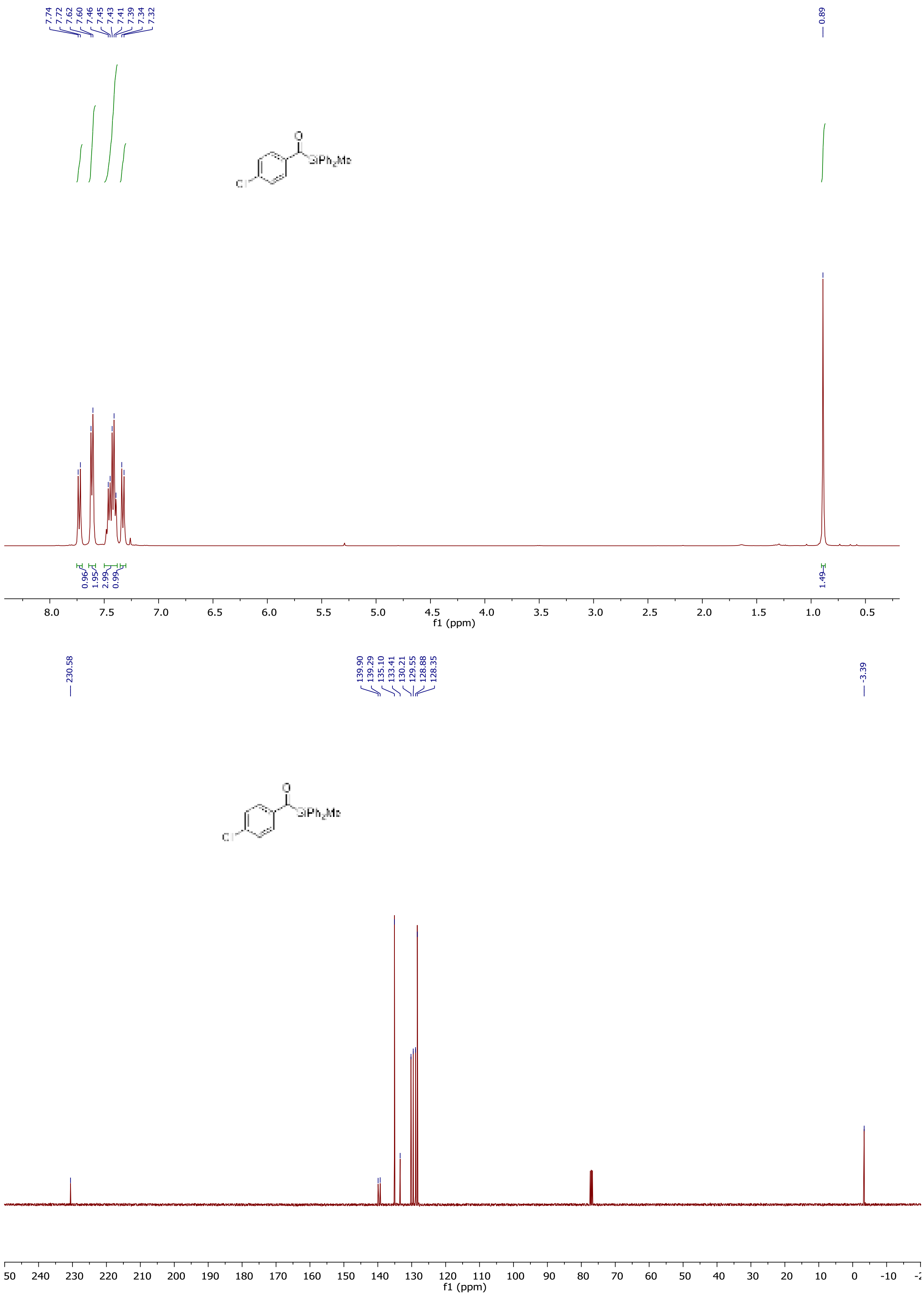

Supplementary Figure 3. 1H, 13C-NMR spectra of product **9f**.

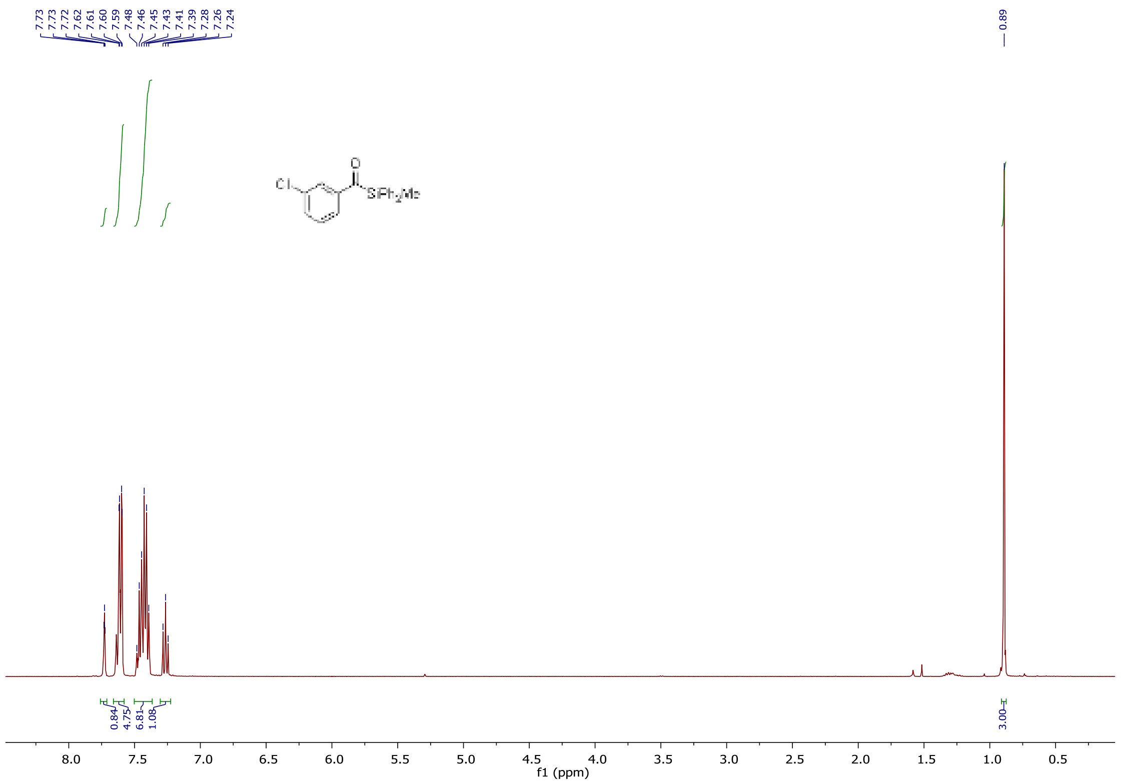

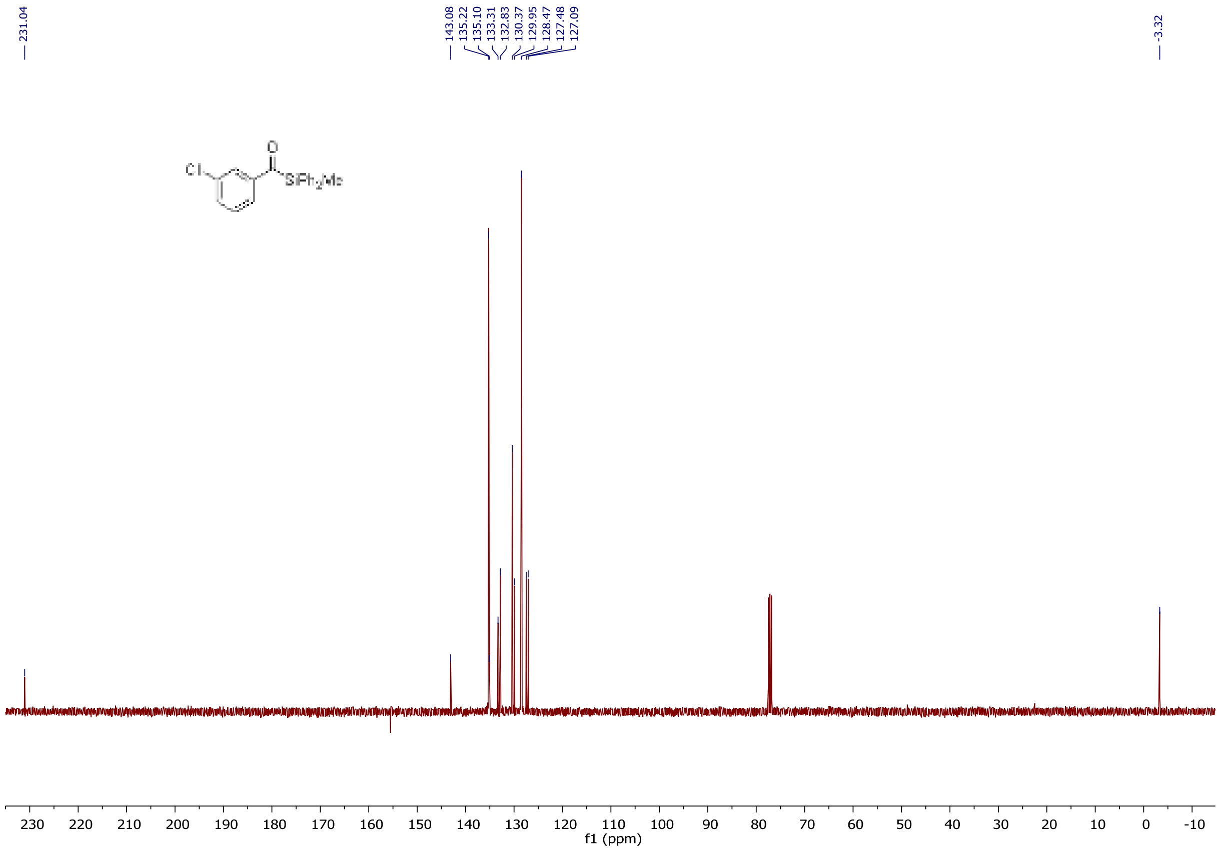

Supplementary Figure 4. 1H, 13C-NMR spectra of product **9h**.

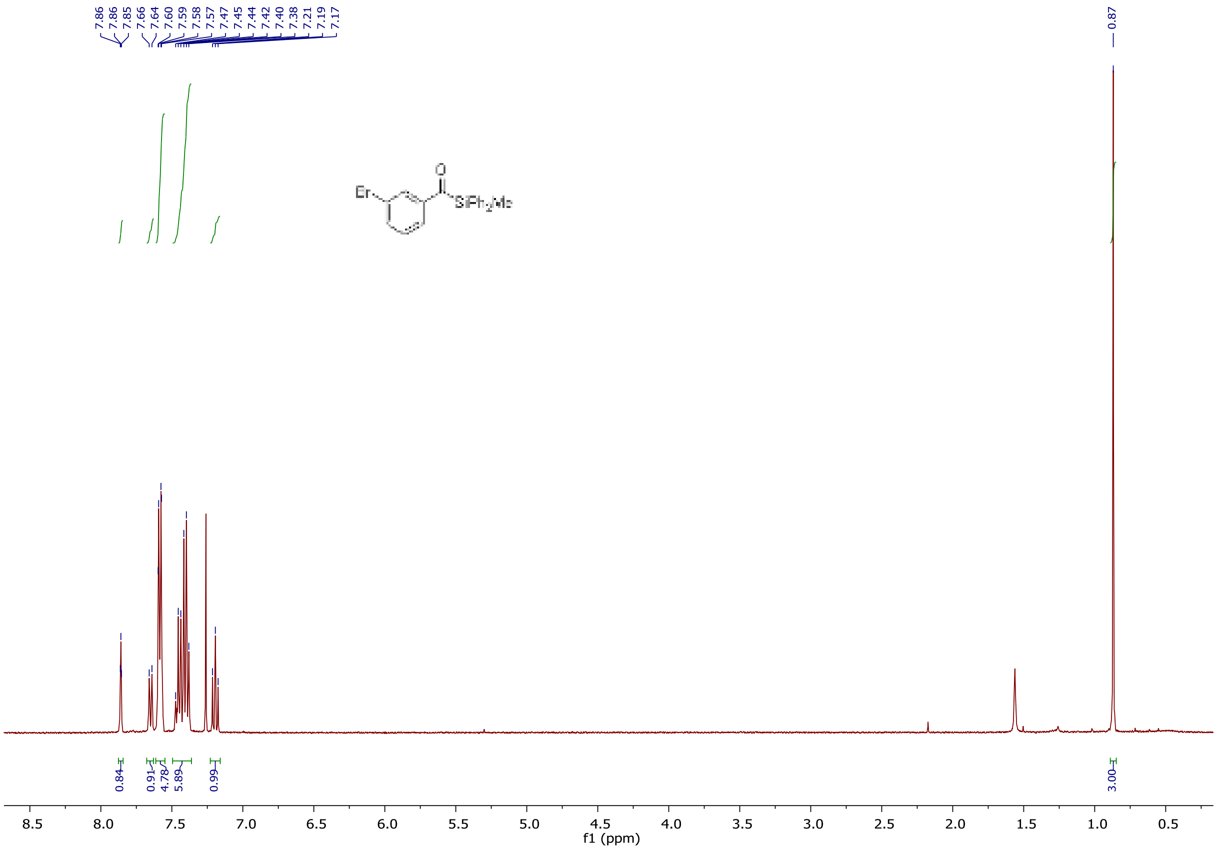

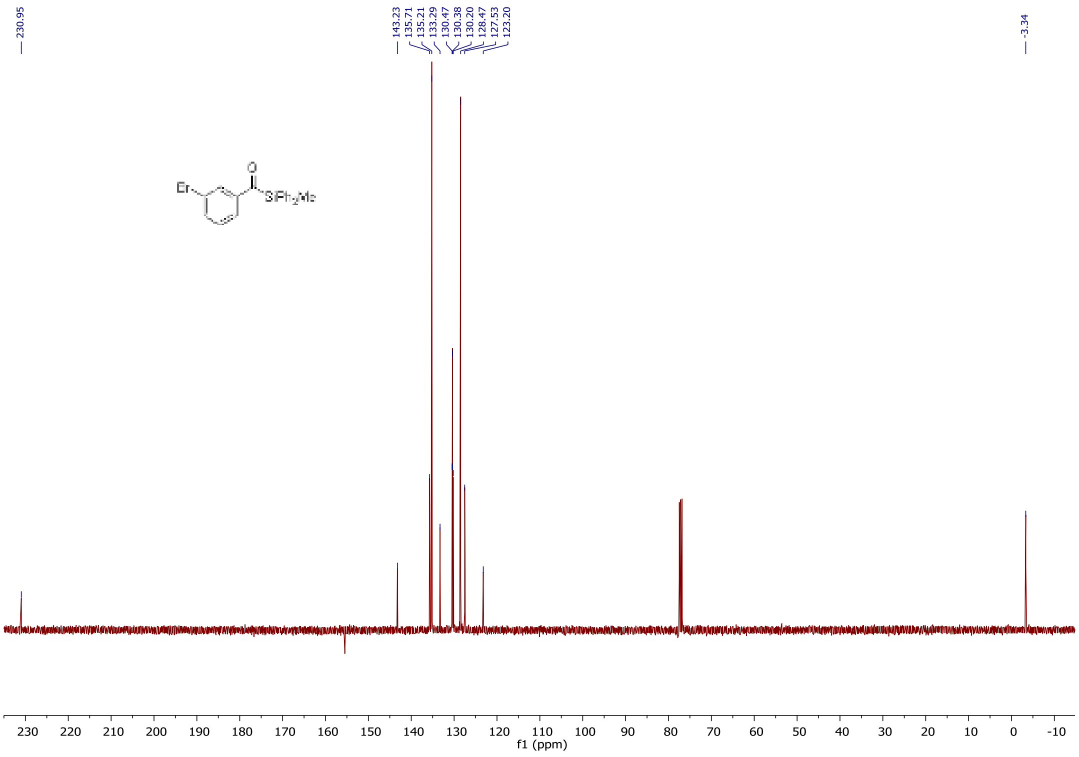

Supplementary Figure 5. 1H, 13C, 19F-NMR spectra of product **9i**.

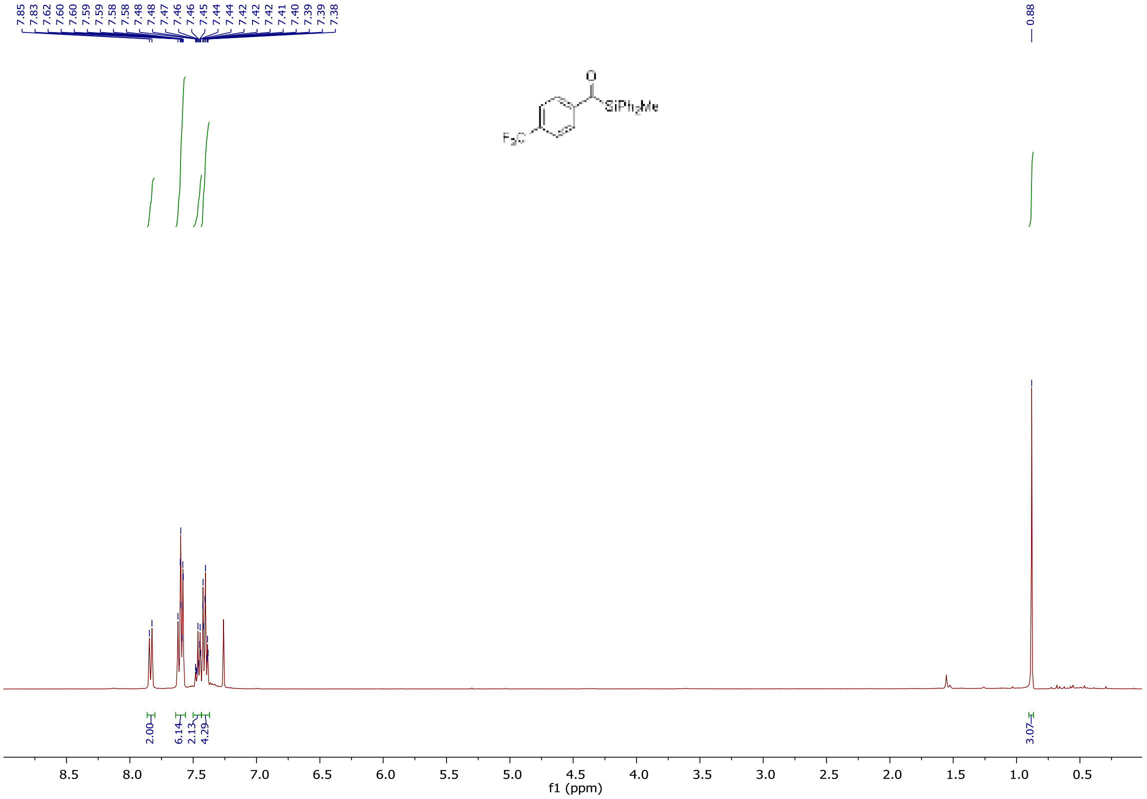

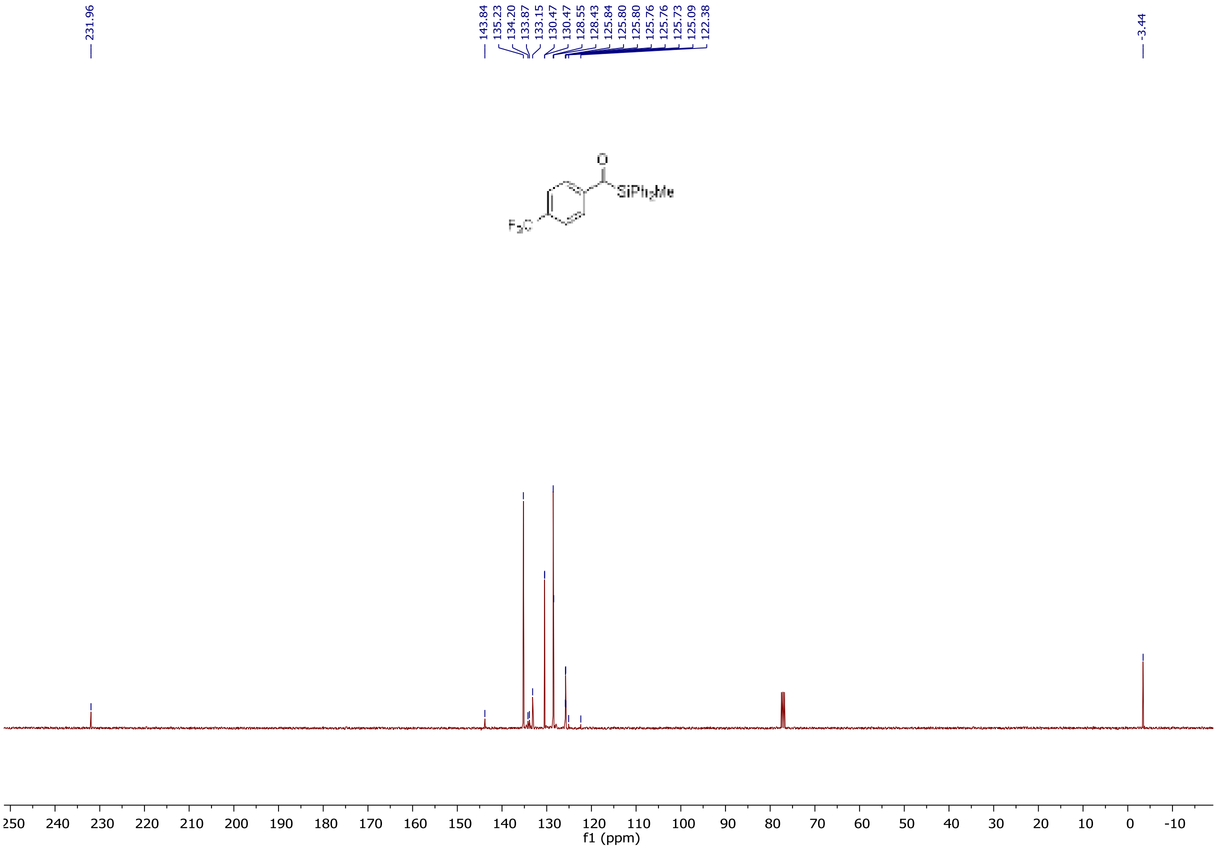

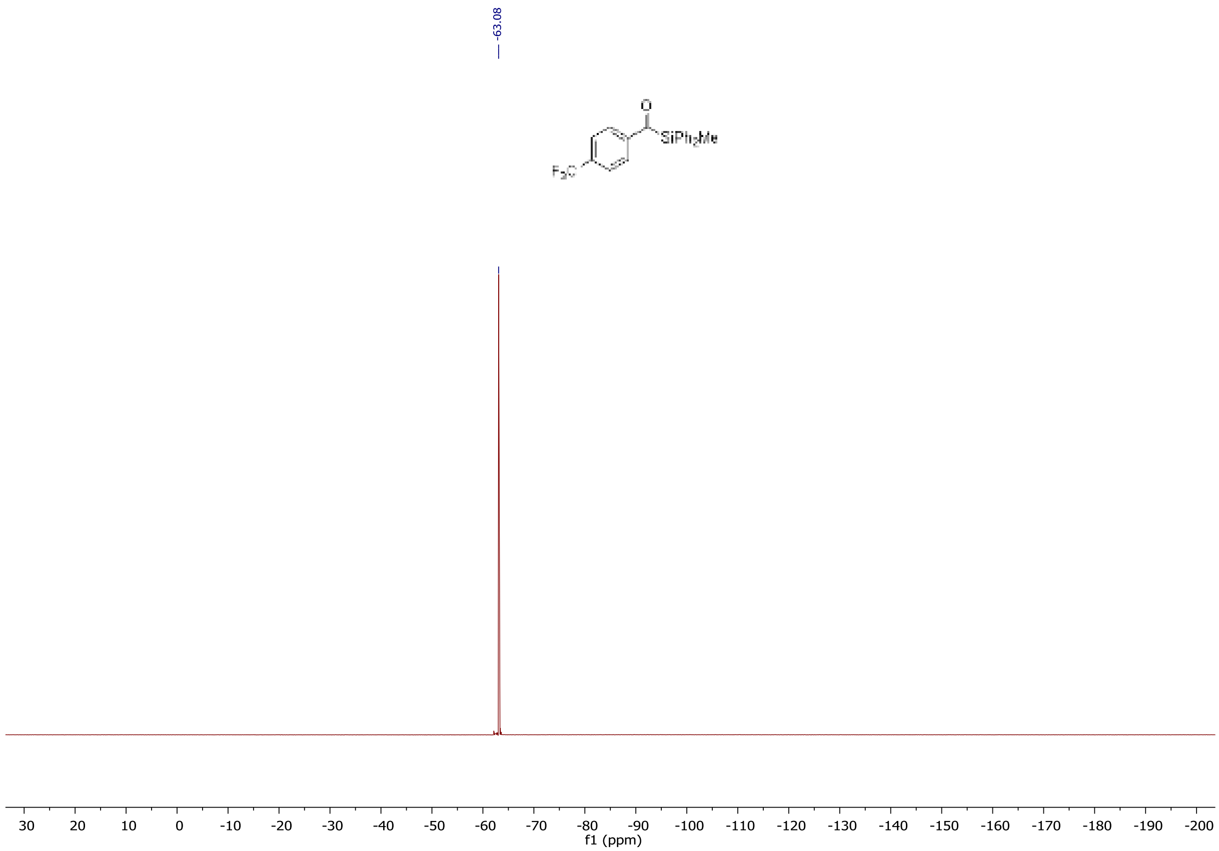
-63.08
SiPh2Me
F3C
30
20
10
0
-10
-20
-30
-40
-50
-60
-70
-80
-90
-100
-110
-120
-130
-140
-150
-160
-170
-180
-190
-200
f1 (ppm)

Supplementary Figure 6. 1H, 13C, 19F--NMR spectra of product **9j**.

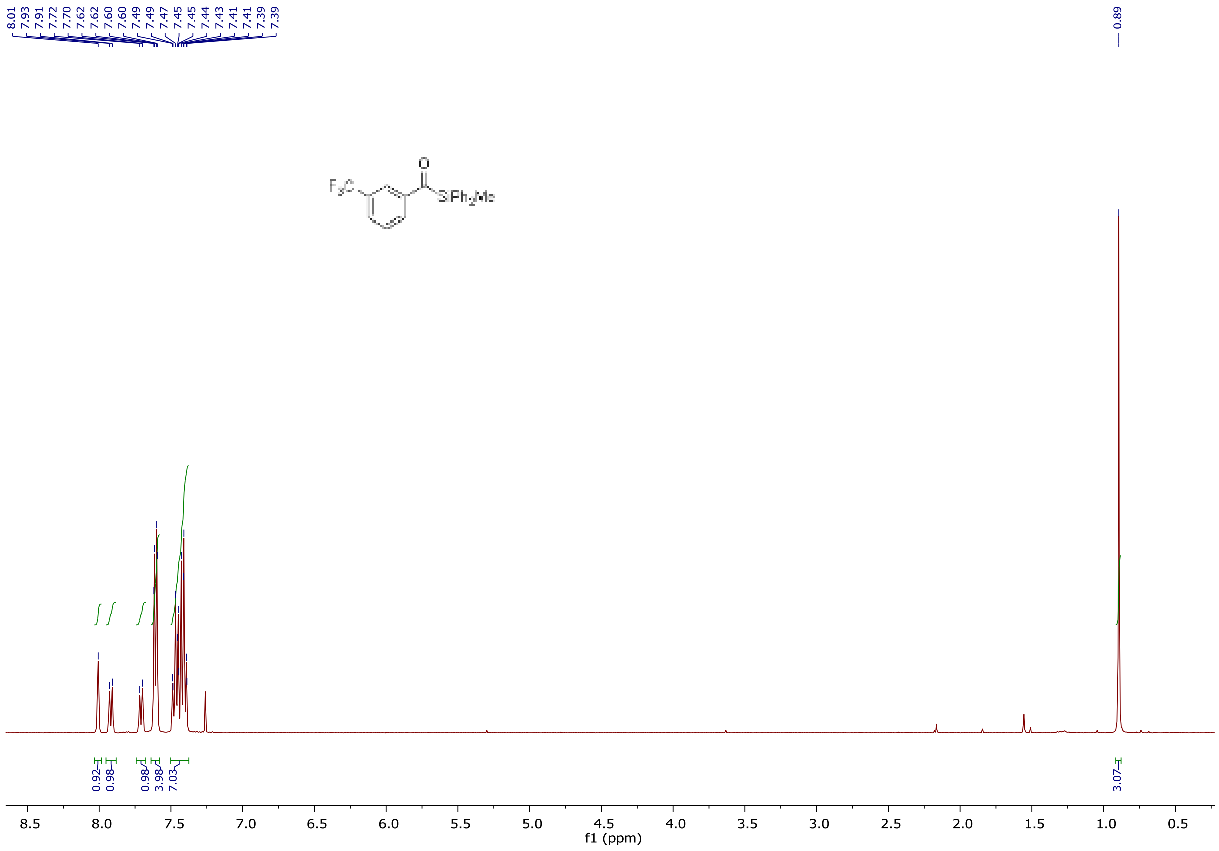

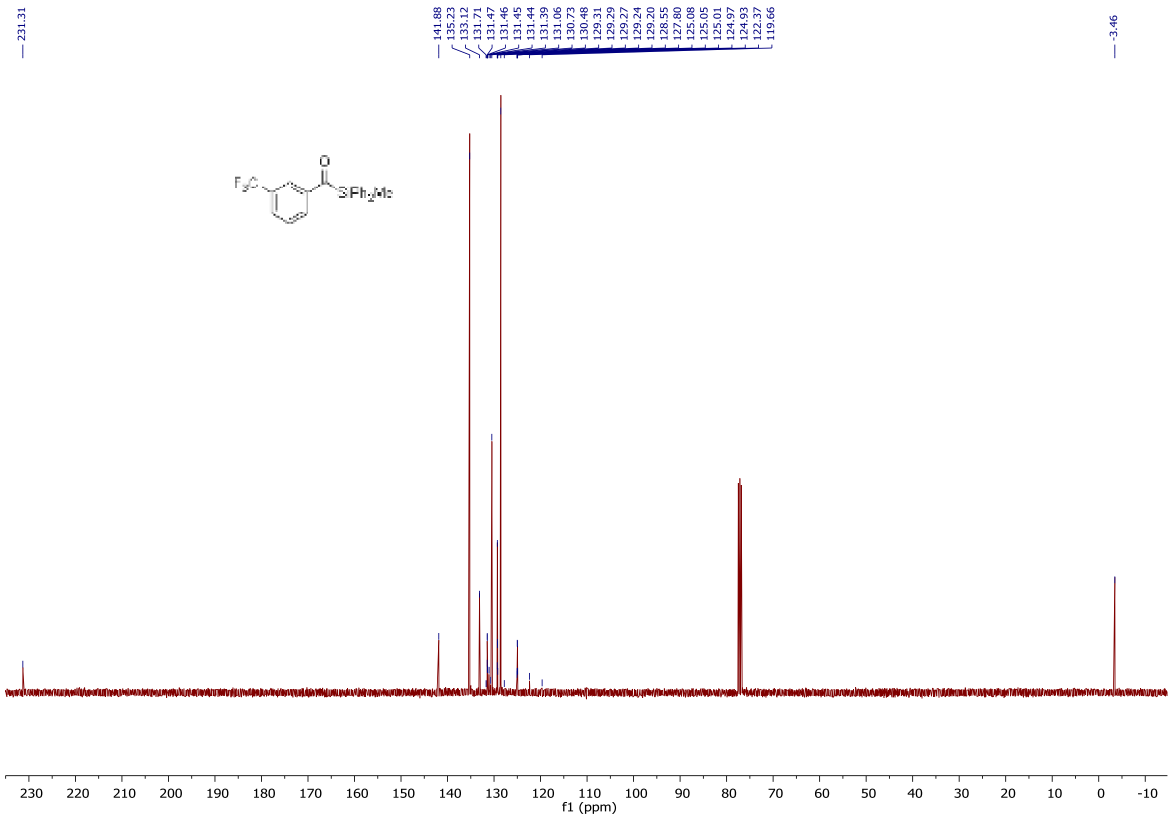

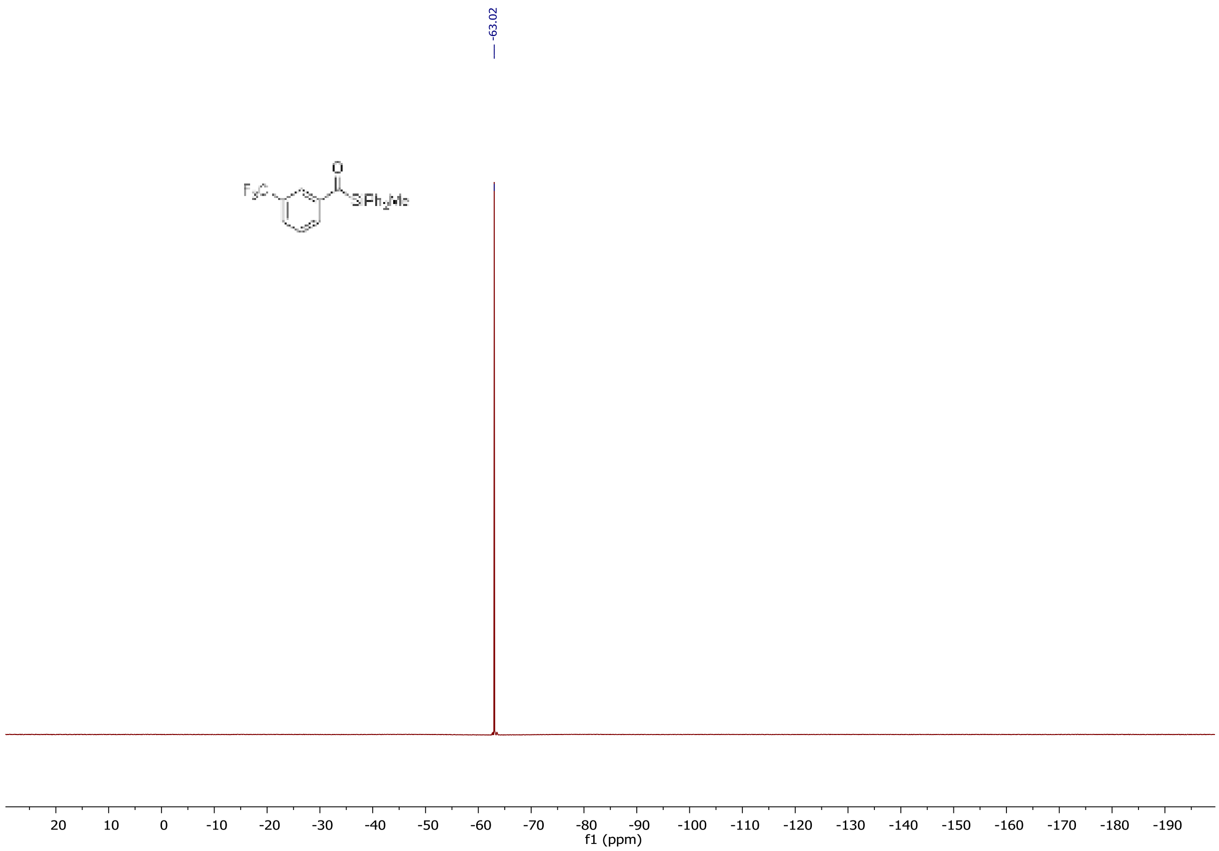

-63.02
F3C
O
SiPh2Me
20
10
0
-10
-20
-30
-40
-50
-60
-70
-80
-90
-100
-110
-120
-130
-140
-150
-160
-170
-180
-190
f1 (ppm)

Supplementary Figure 7. 1H, 13C,19F-NMR spectra of product **9p**.

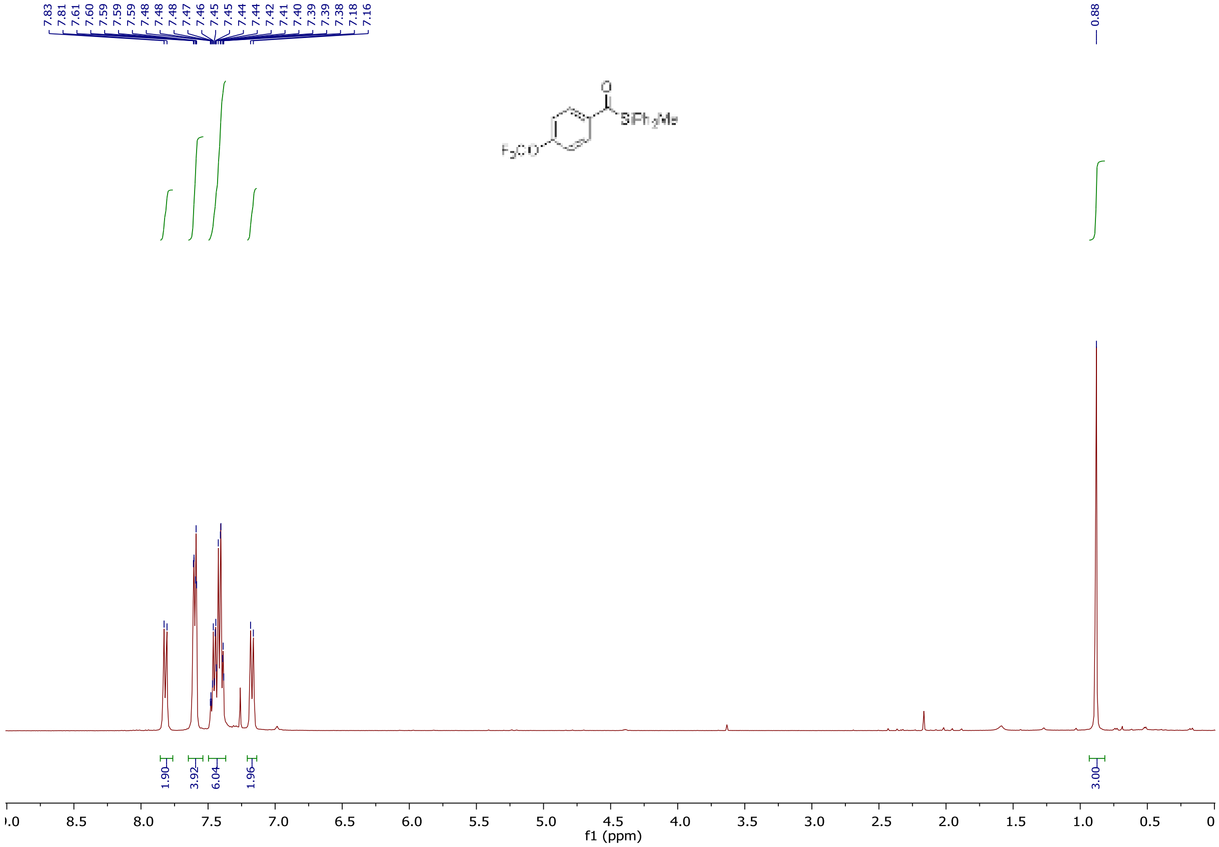

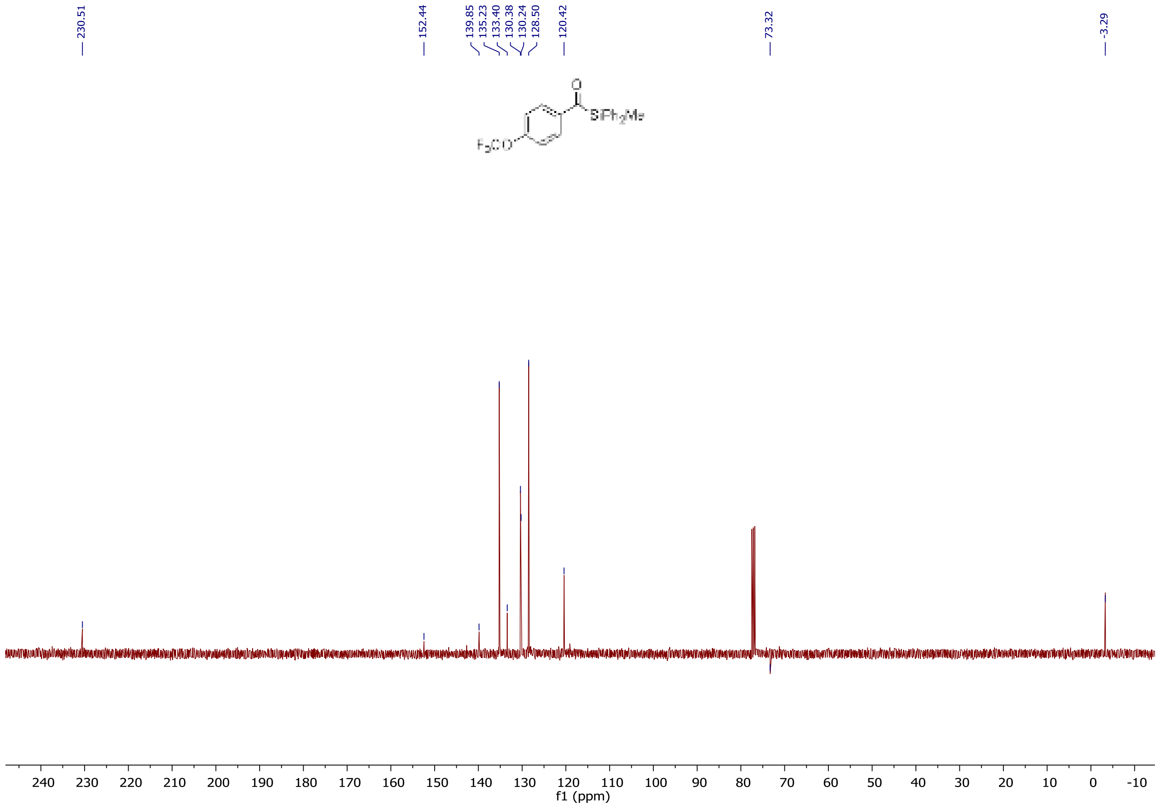

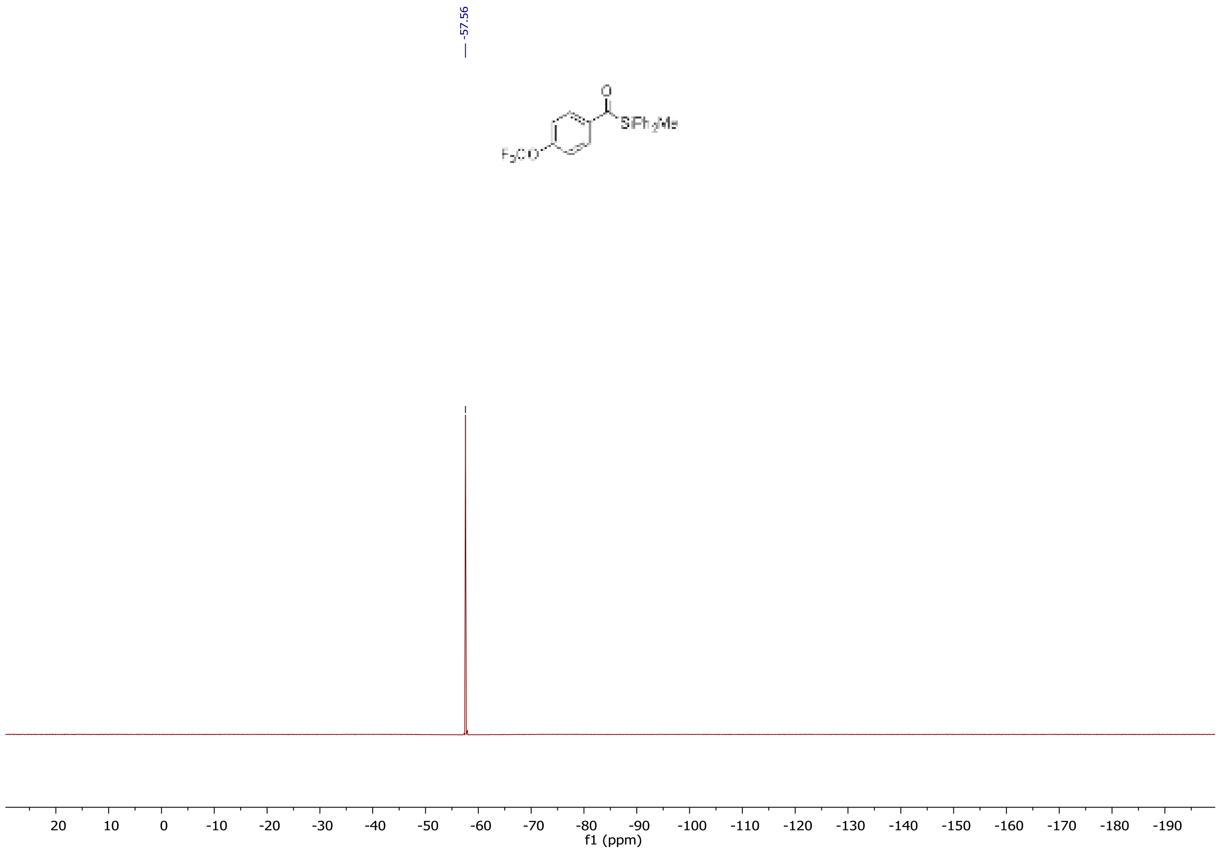
-57.56
F3CO
SiPh2Me
20 10 0 -10 -20 -30 -40 -50 -60 -70 -80 -90 -100 -110 -120 -130 -140 -150 -160 -170 -180 -190
f1 (ppm)

Supplementary Figure 8. 1H, 13C, 19F-NMR spectra of product **9q**.

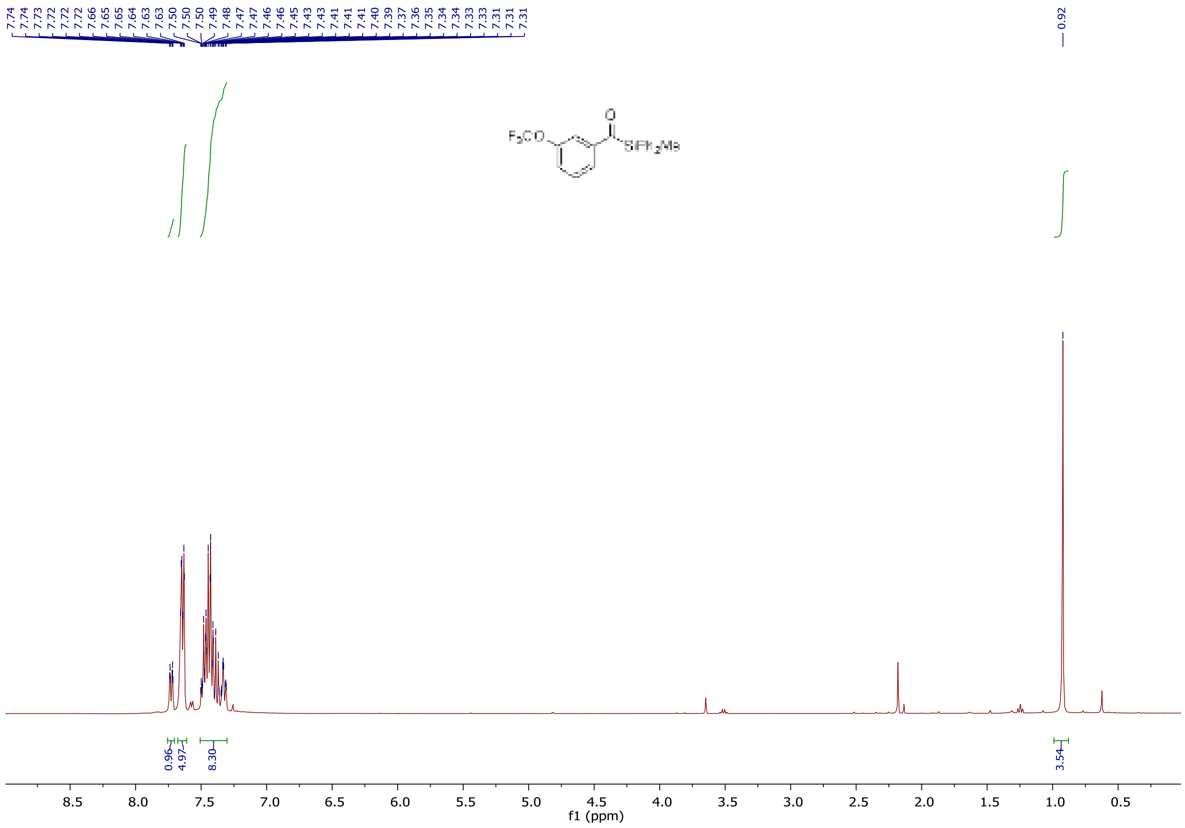

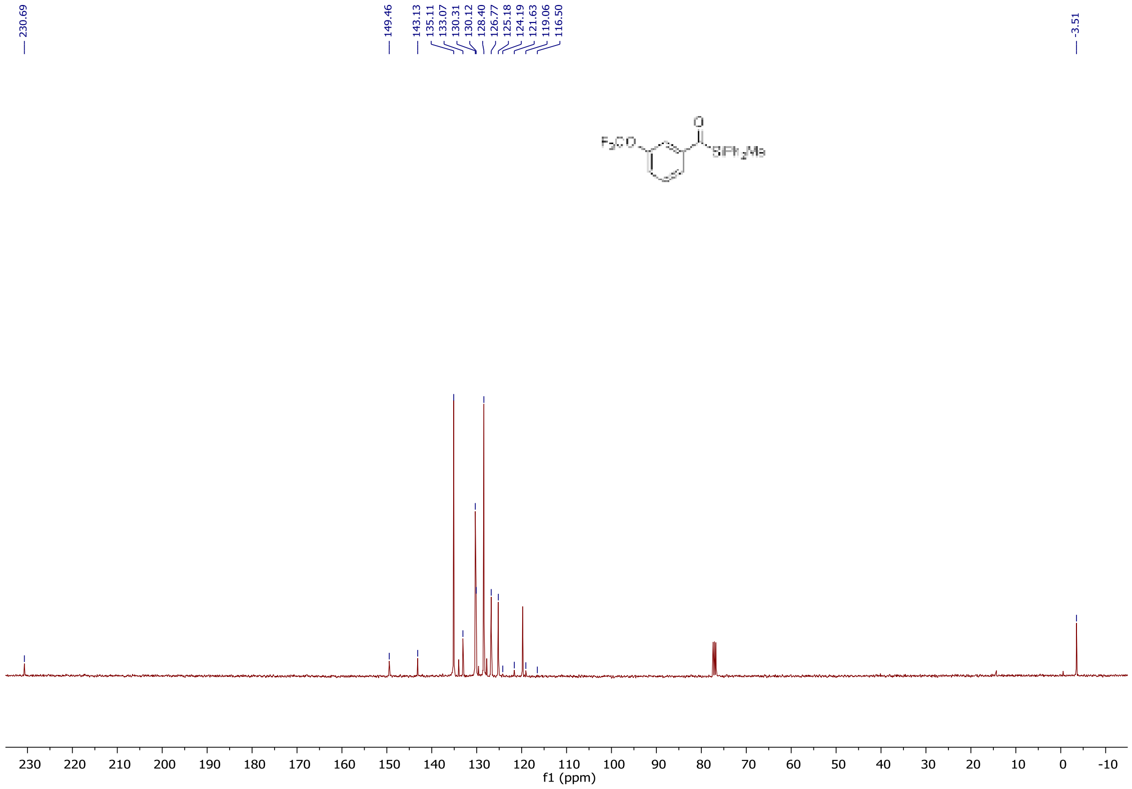

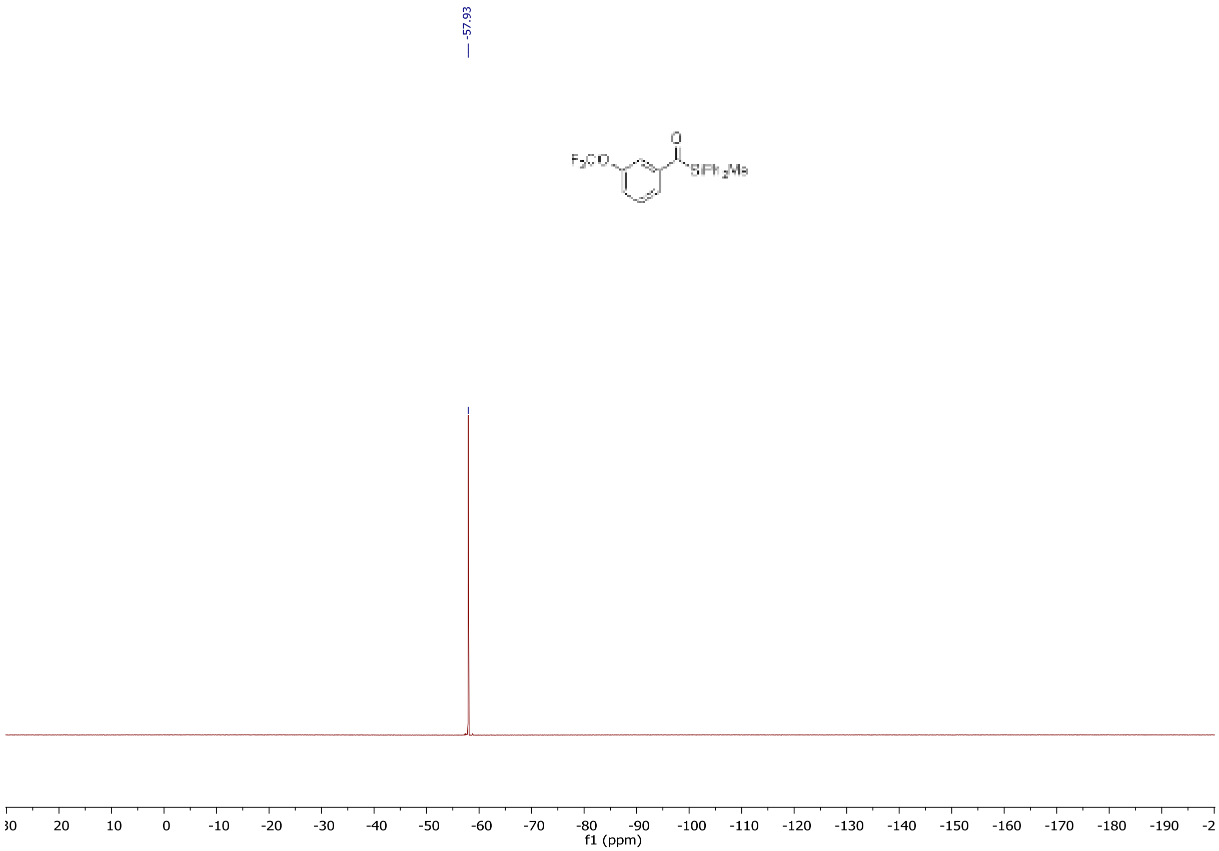
-57.93
F3CO
O
SiPh2Me
f1 (ppm)

Supplementary Figure 9. 1H, 13C-NMR spectra of product **1a**.

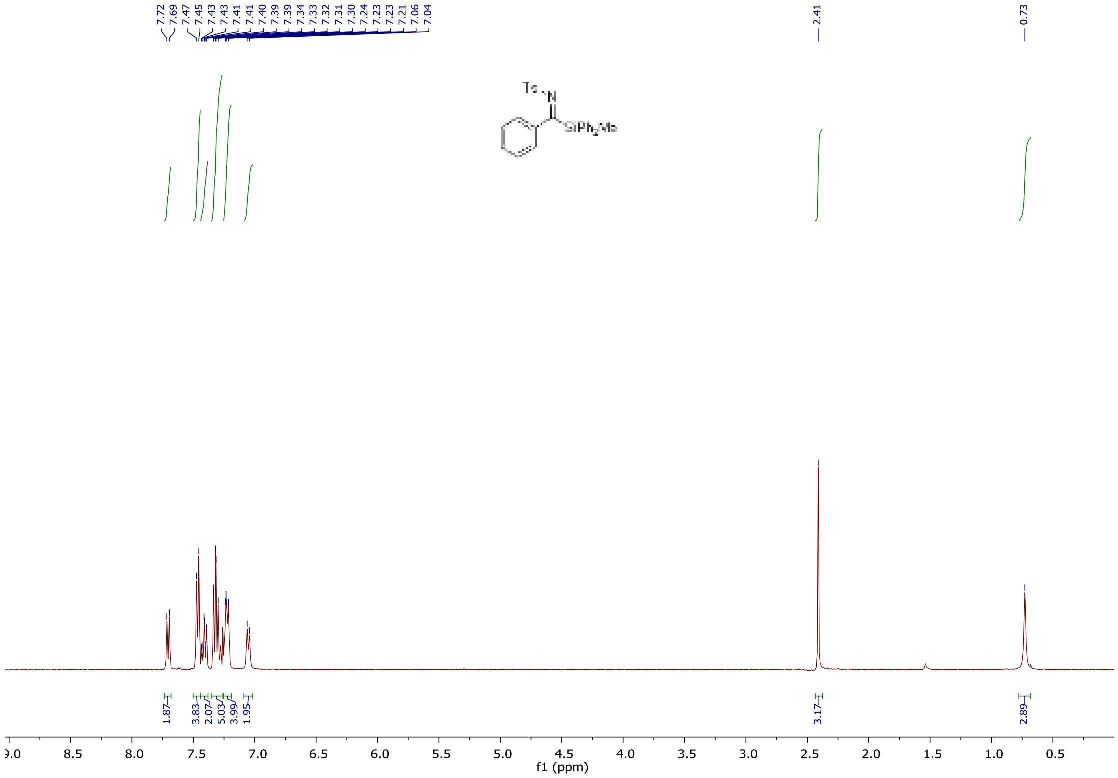

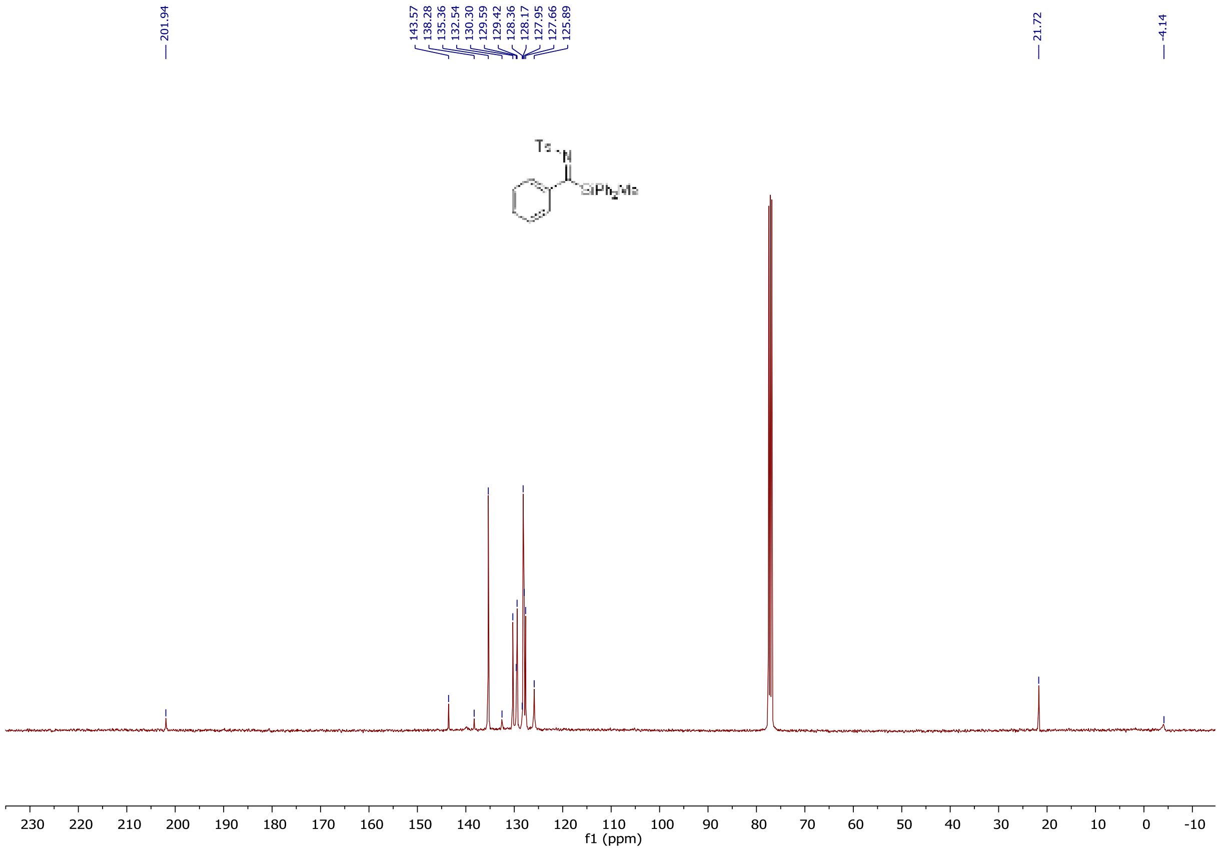

Supplementary Figure 10. 1H, 13C-NMR spectra of product **1ab**.

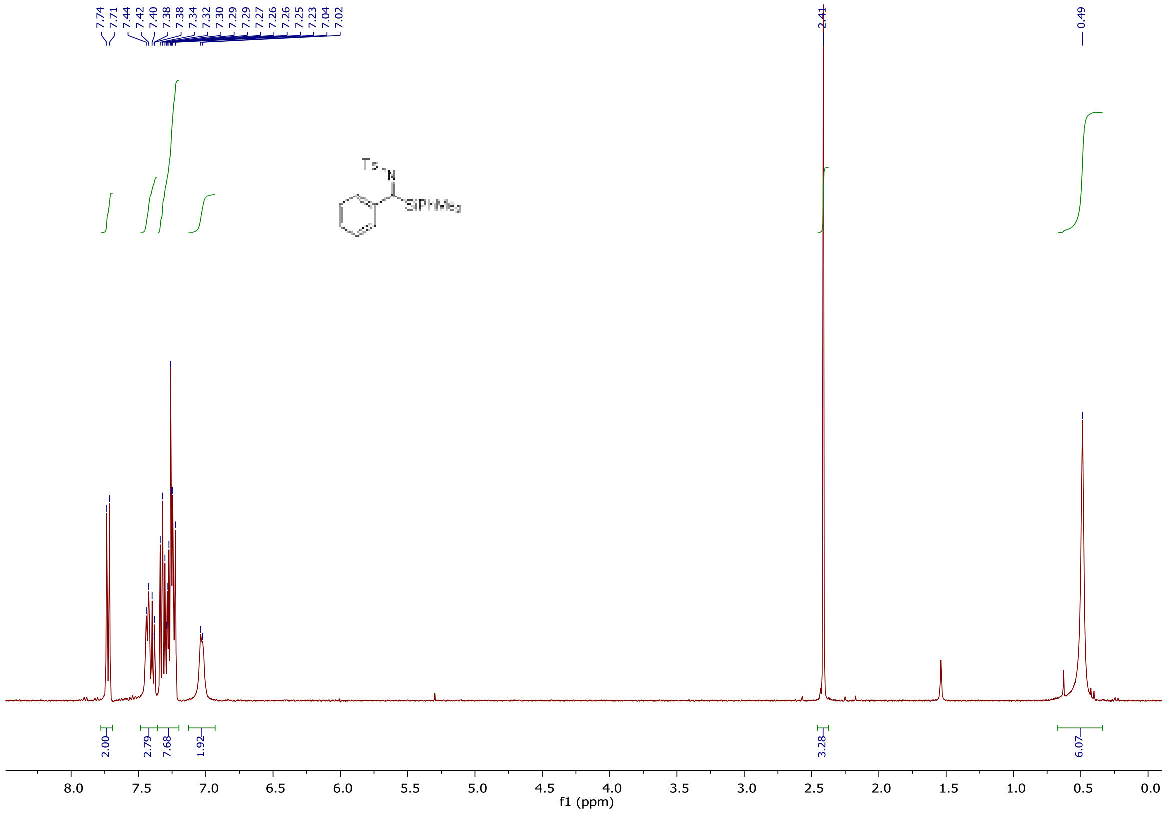

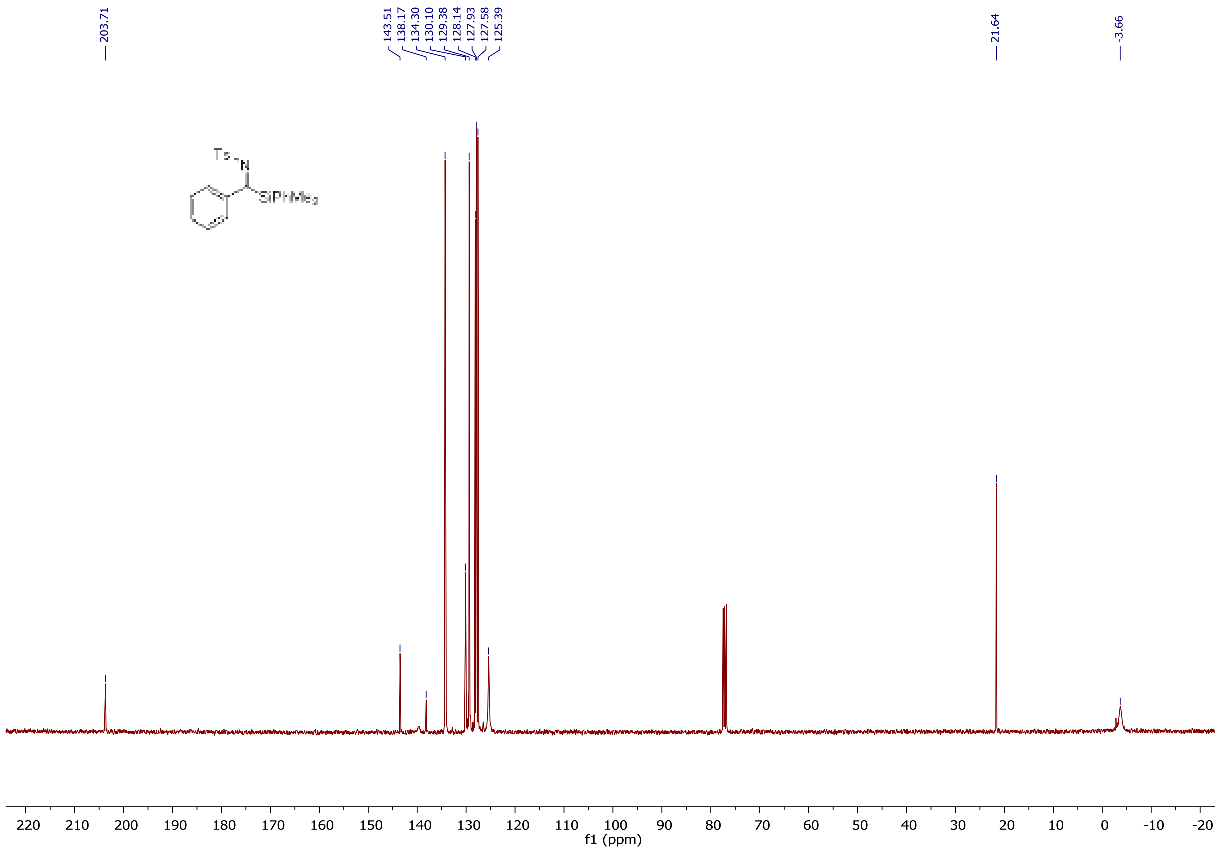

Supplementary Figure 11. 1H, 13C-NMR spectra of product **1ac**.

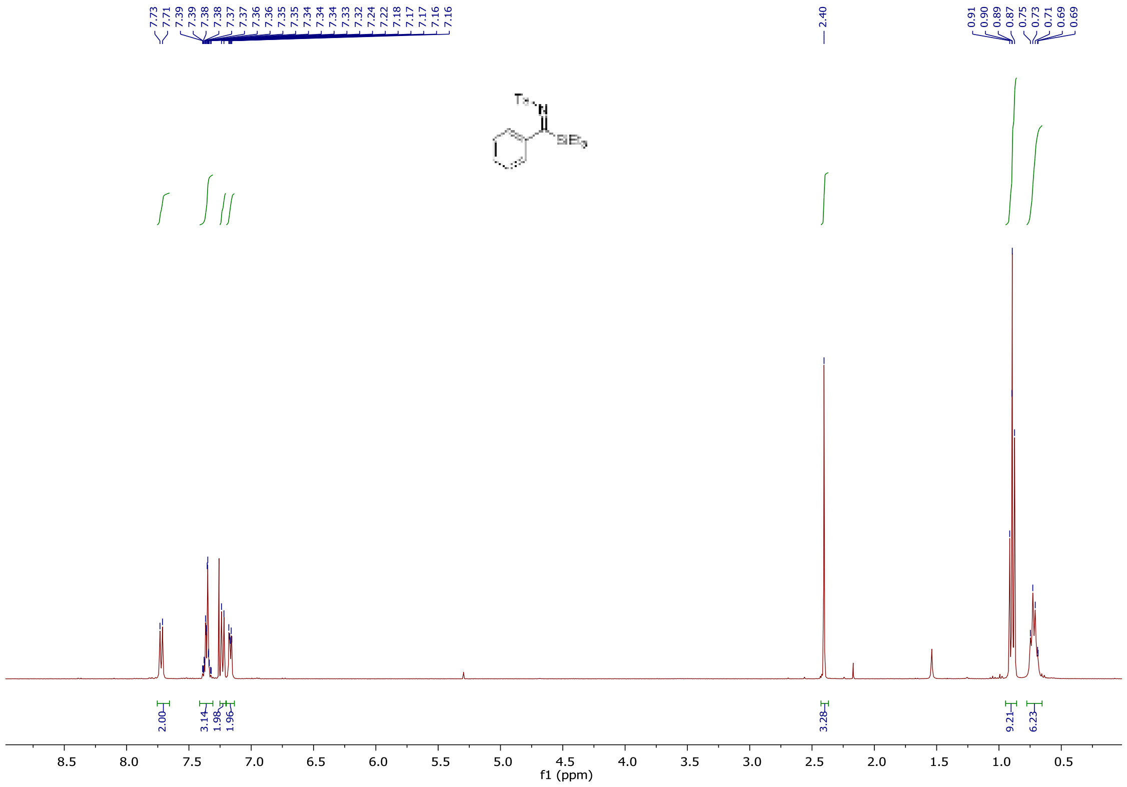

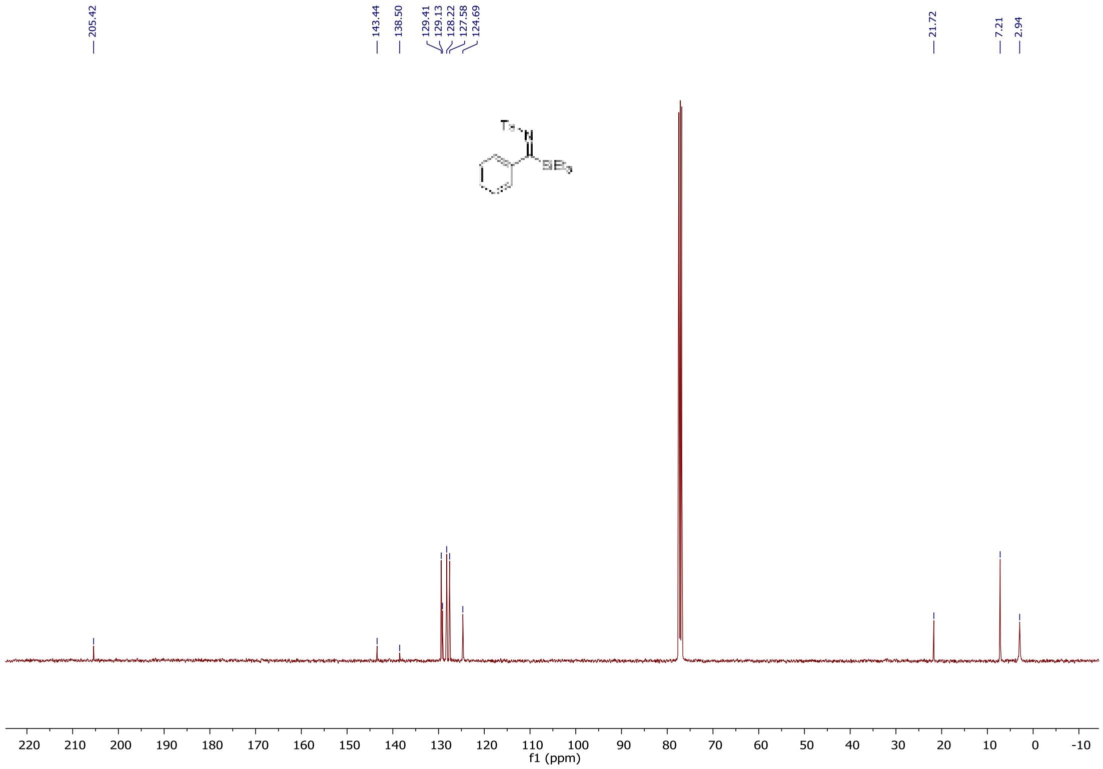

Supplementary Figure 12. 1H, 13C-NMR spectra of product **1ad**

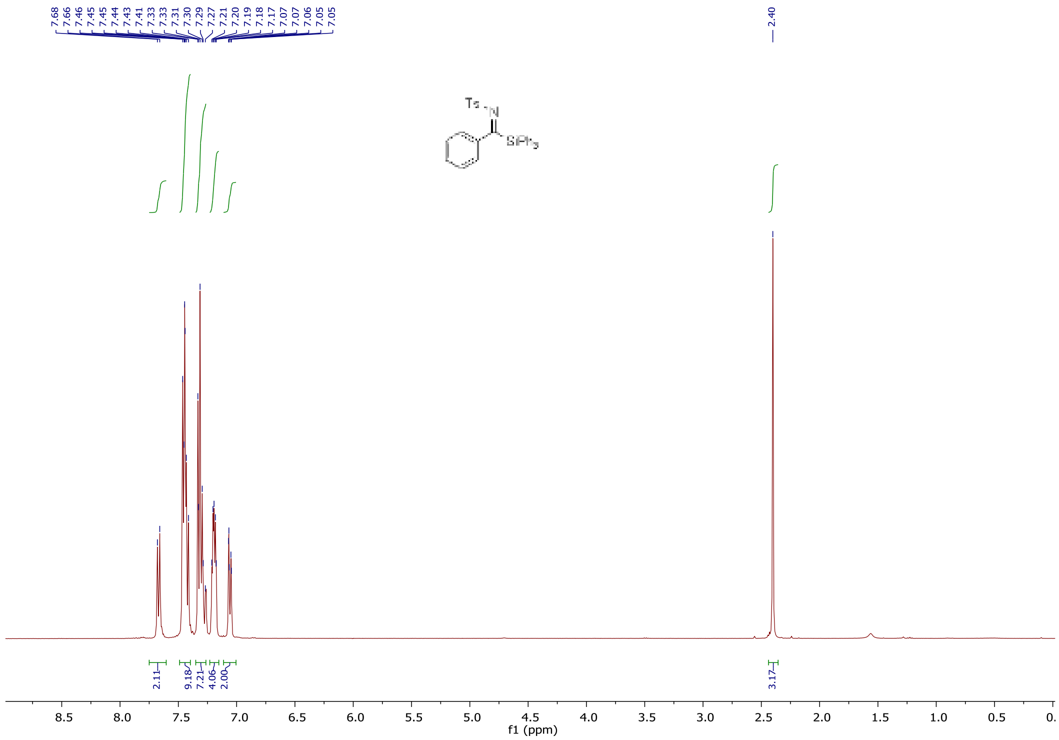

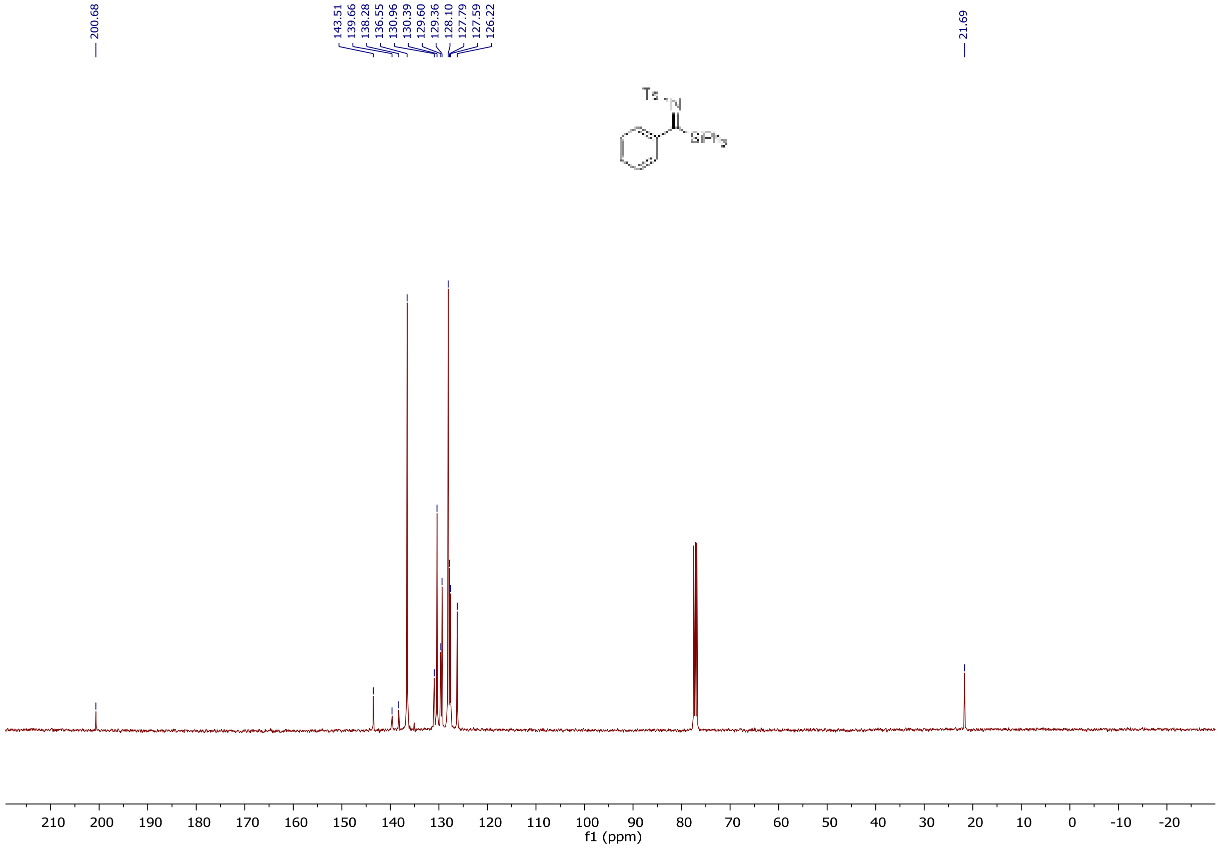

Supplementary Figure 13. 1H, 13C-NMR spectra of product **1ae**.

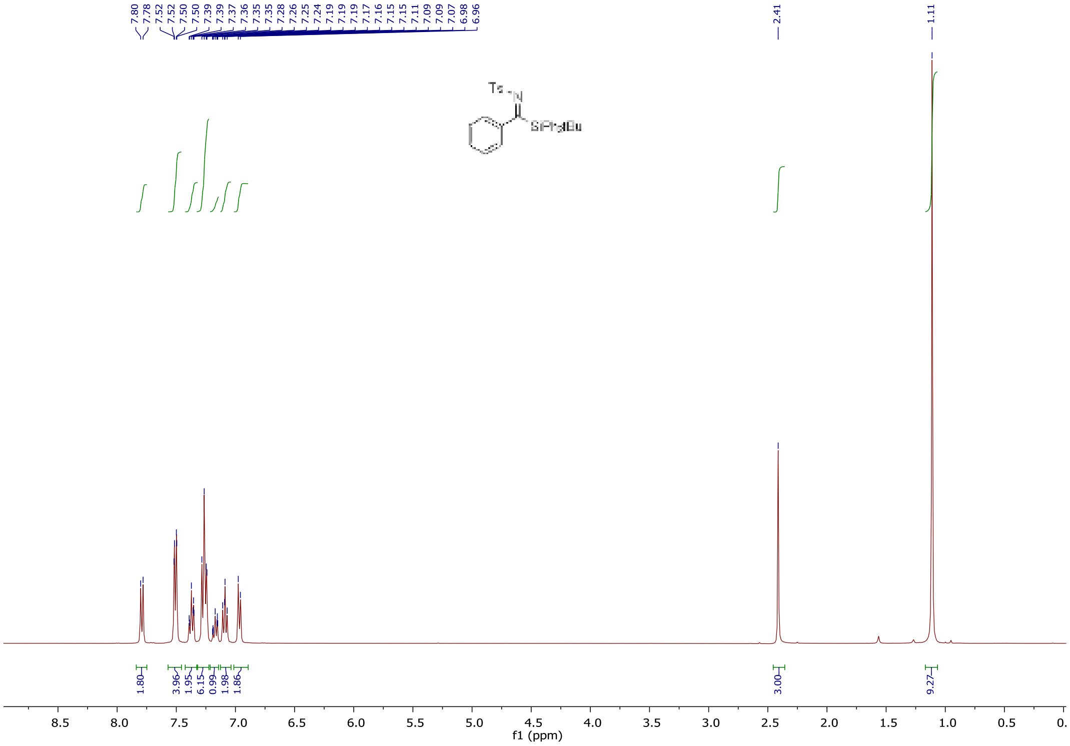

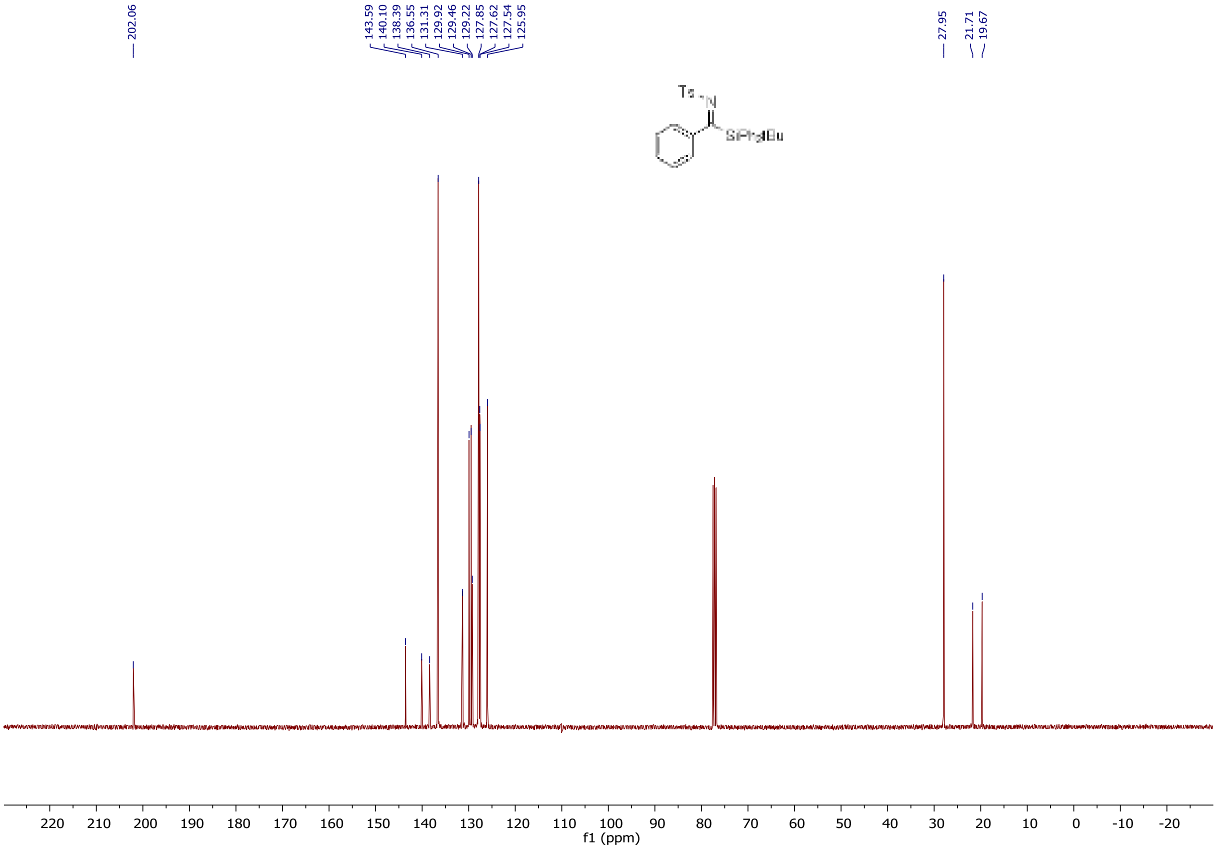

Supplementary Figure 14. 1H, 13C, 19F-NMR spectra of product **1b**.

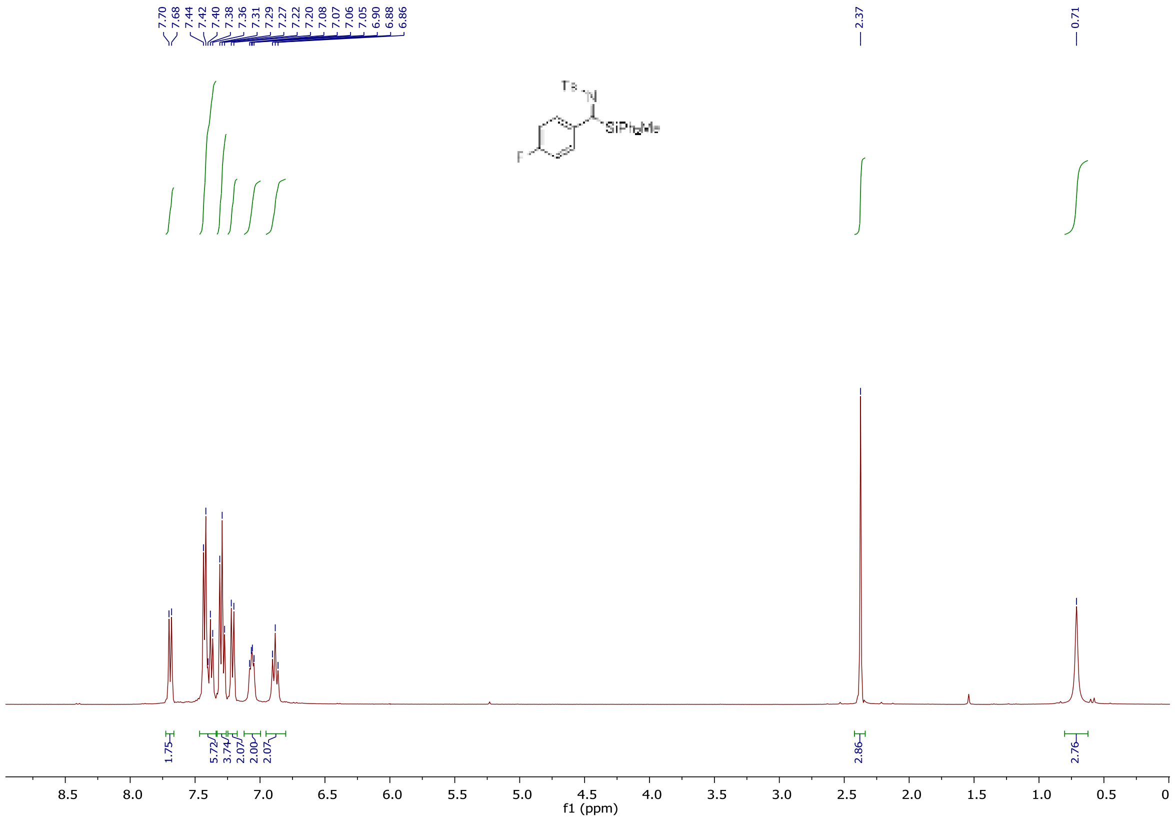

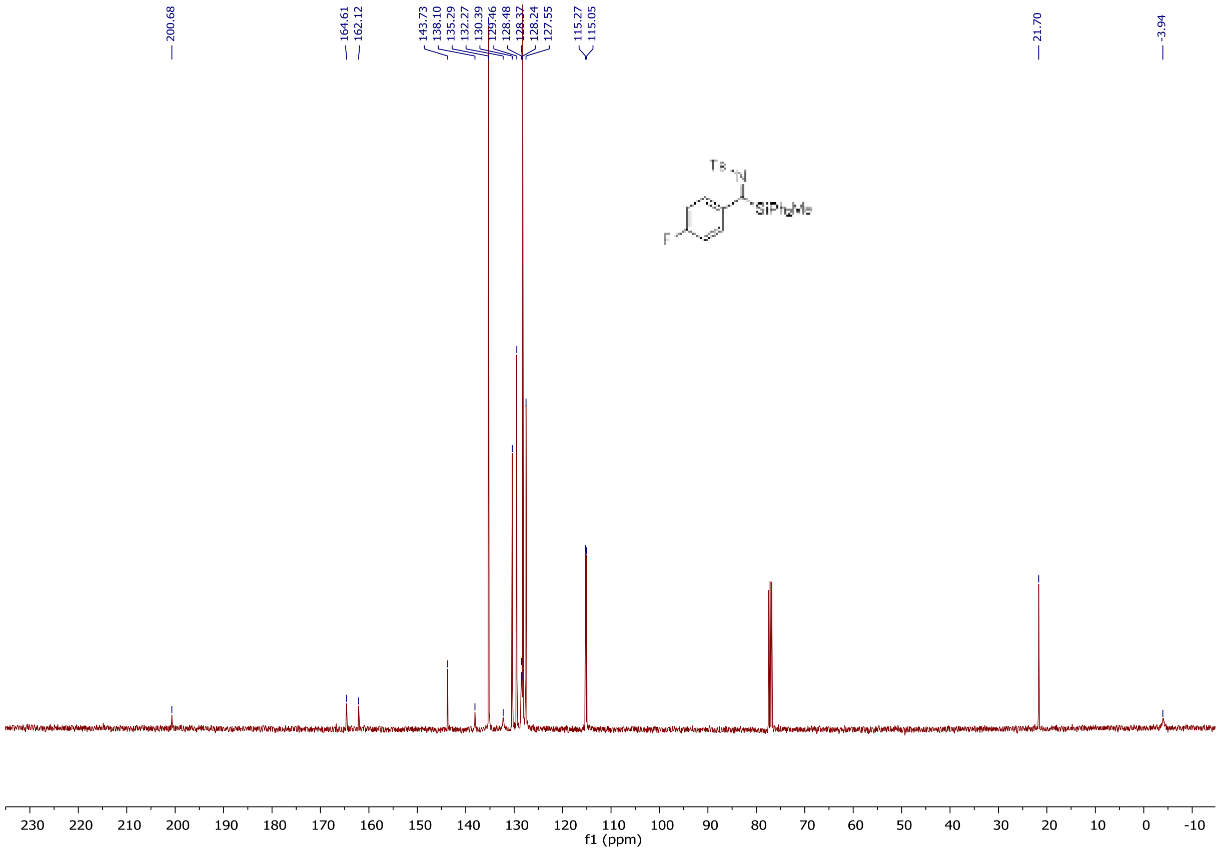

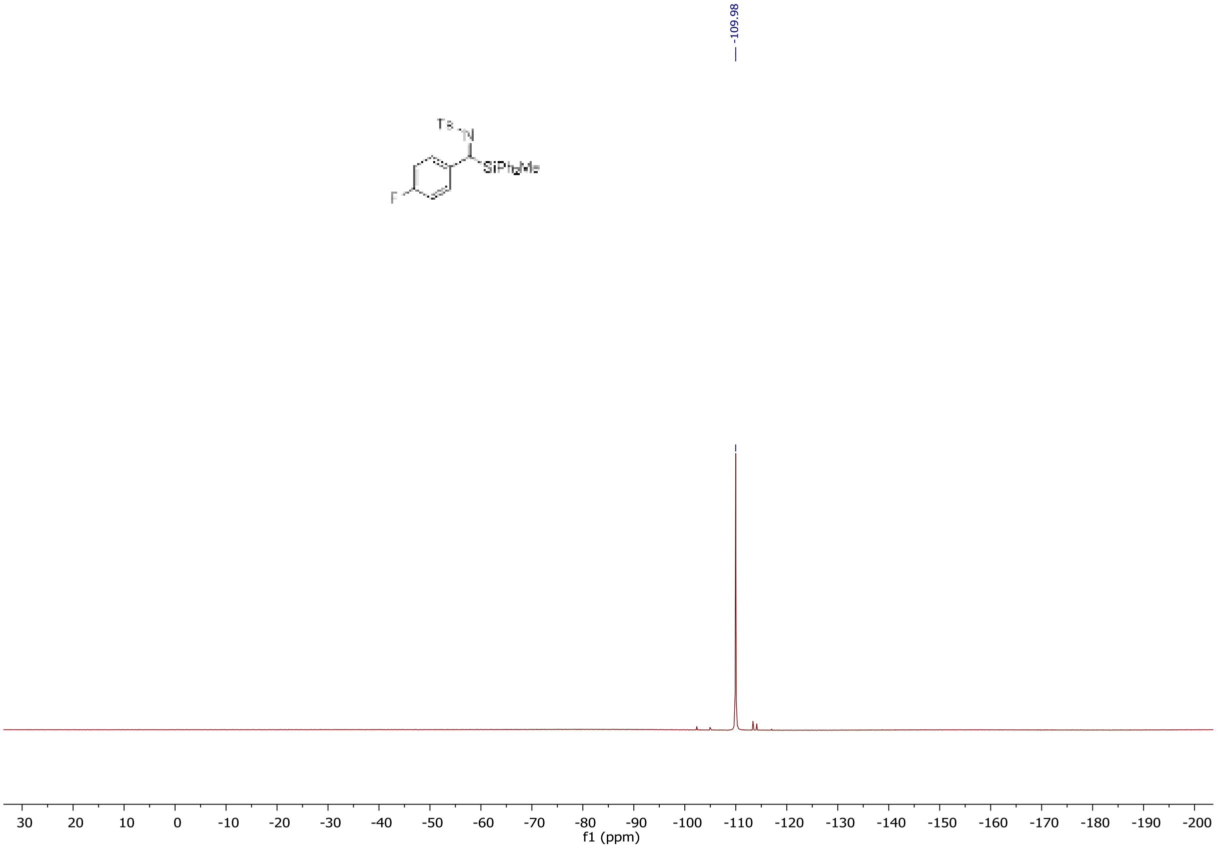
-109.98
Ts
N
SiPh2Me
F
30 20 10 0 -10 -20 -30 -40 -50 -60 -70 -80 -90 -100 -110 -120 -130 -140 -150 -160 -170 -180 -190 -200
f1 (ppm)

Supplementary Figure 15. 1H, 13C, 19F-NMR spectra of product **1c**.

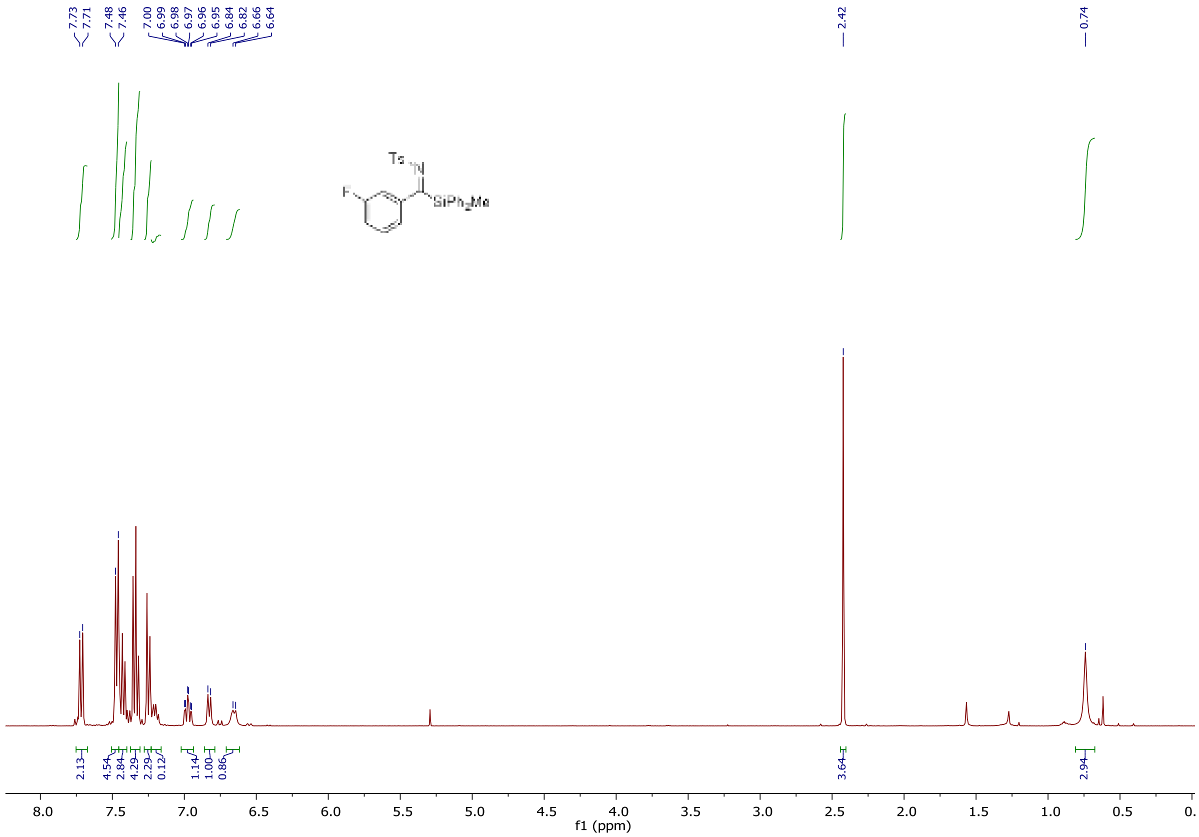

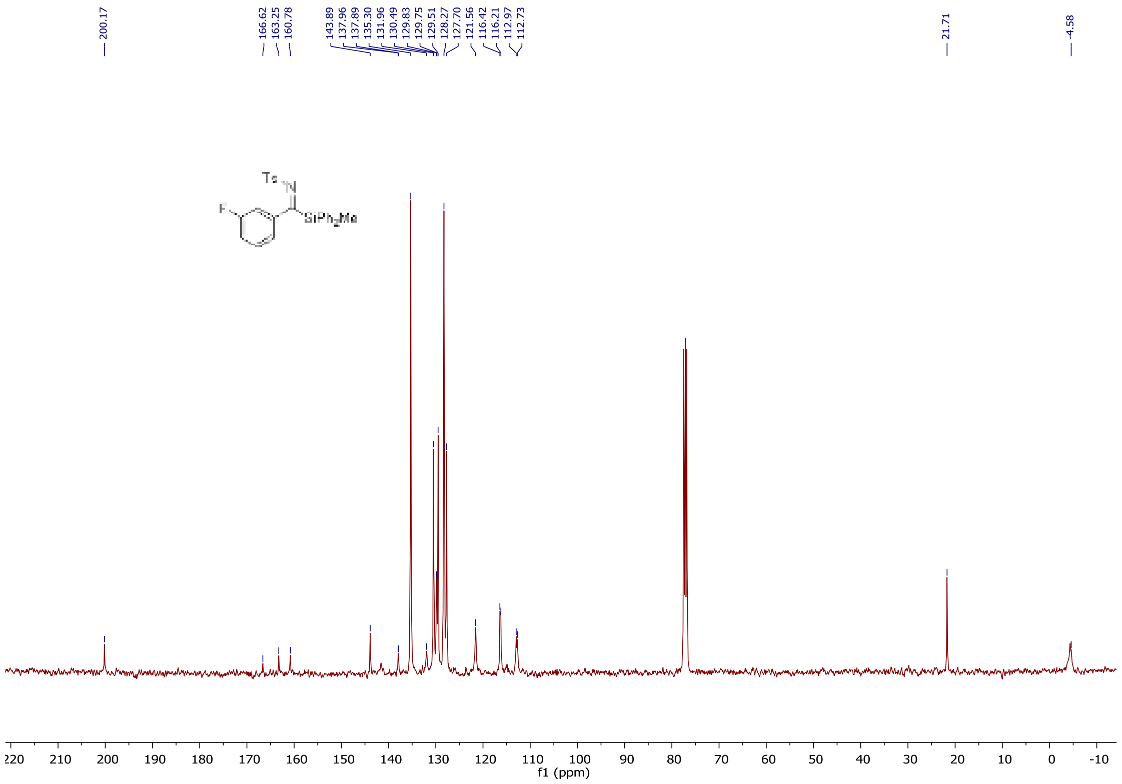

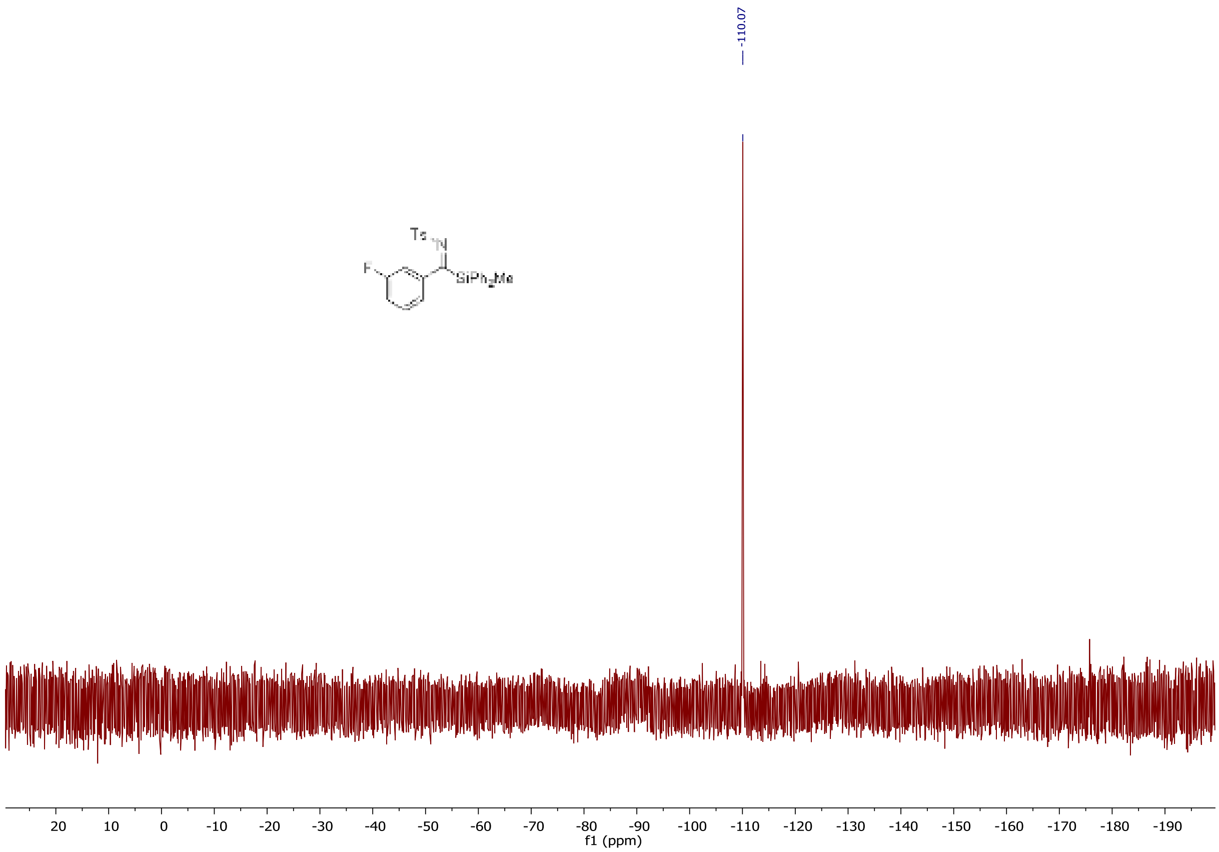
-110.07
20
10
0
-10
-20
-30
-40
-50
-60
-70
-80
-90
-100
-110
-120
-130
-140
-150
-160
-170
-180
-190
f1 (ppm)

Supplementary Figure 16. 1H, 13C, 19F-NMR spectra of product **1d**.

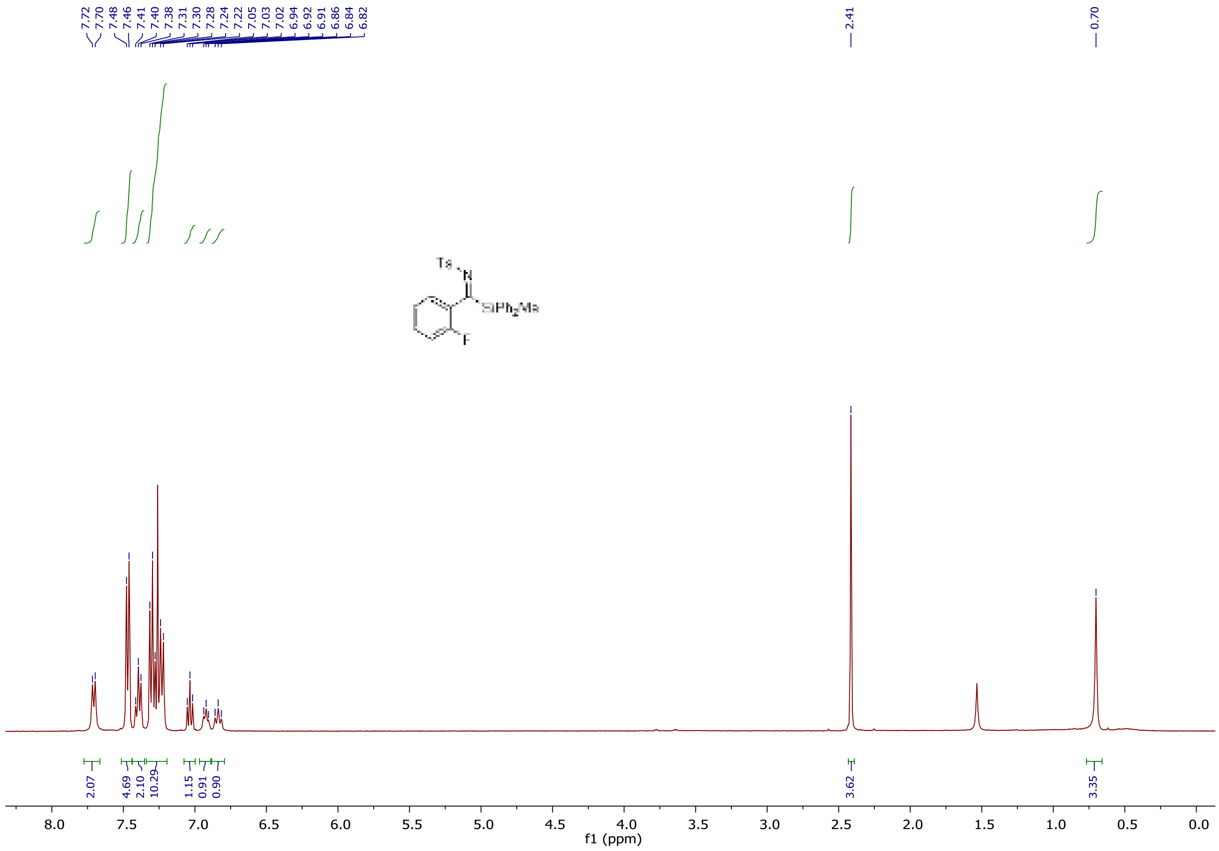

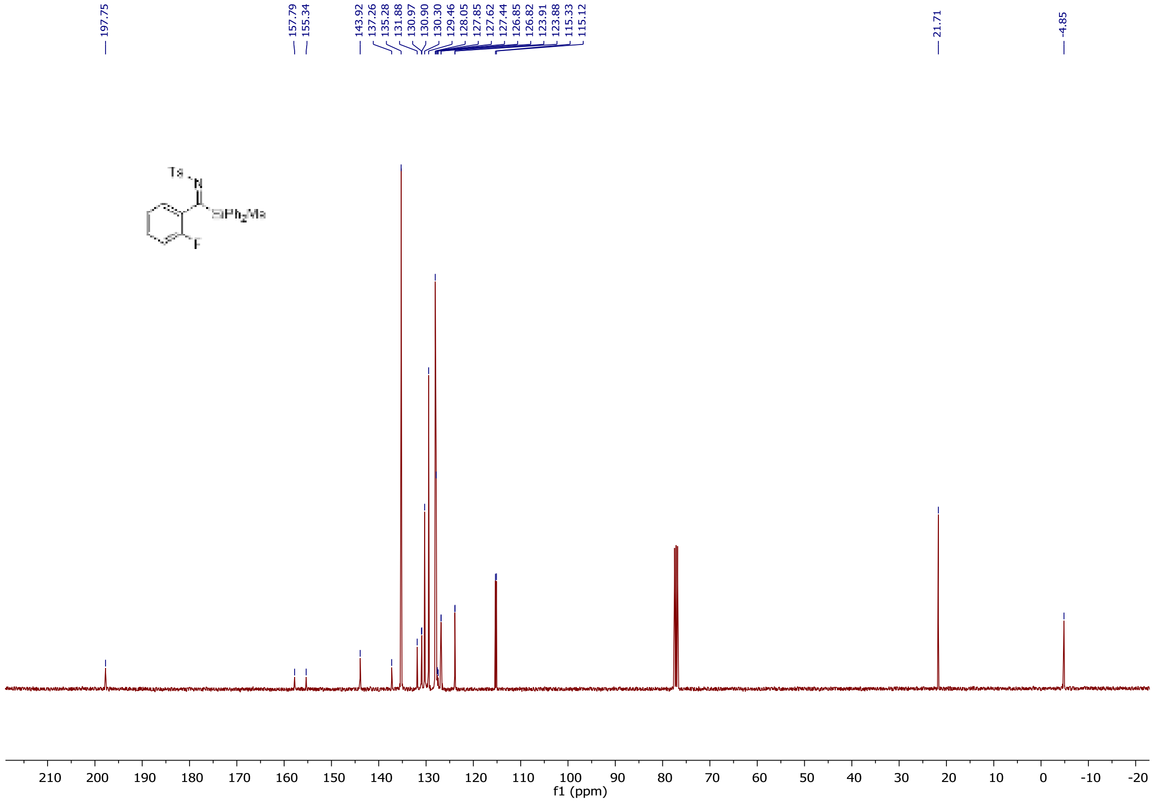

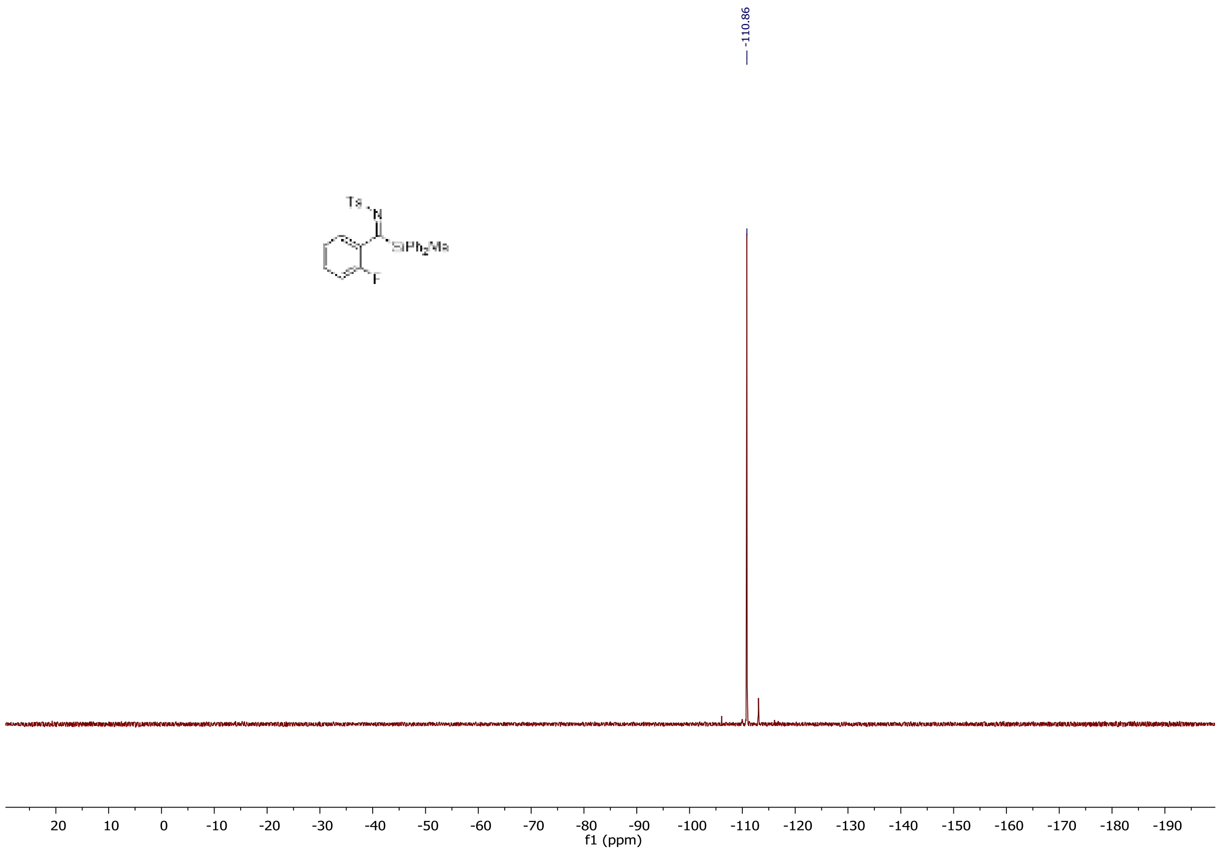
-110.86
Ts
N
SiPh2Me
F
20 10 0 -10 -20 -30 -40 -50 -60 -70 -80 -90 -100 -110 -120 -130 -140 -150 -160 -170 -180 -190
f1 (ppm)

Supplementary Figure 17. 1H, 13C-NMR spectra of product **1e**.

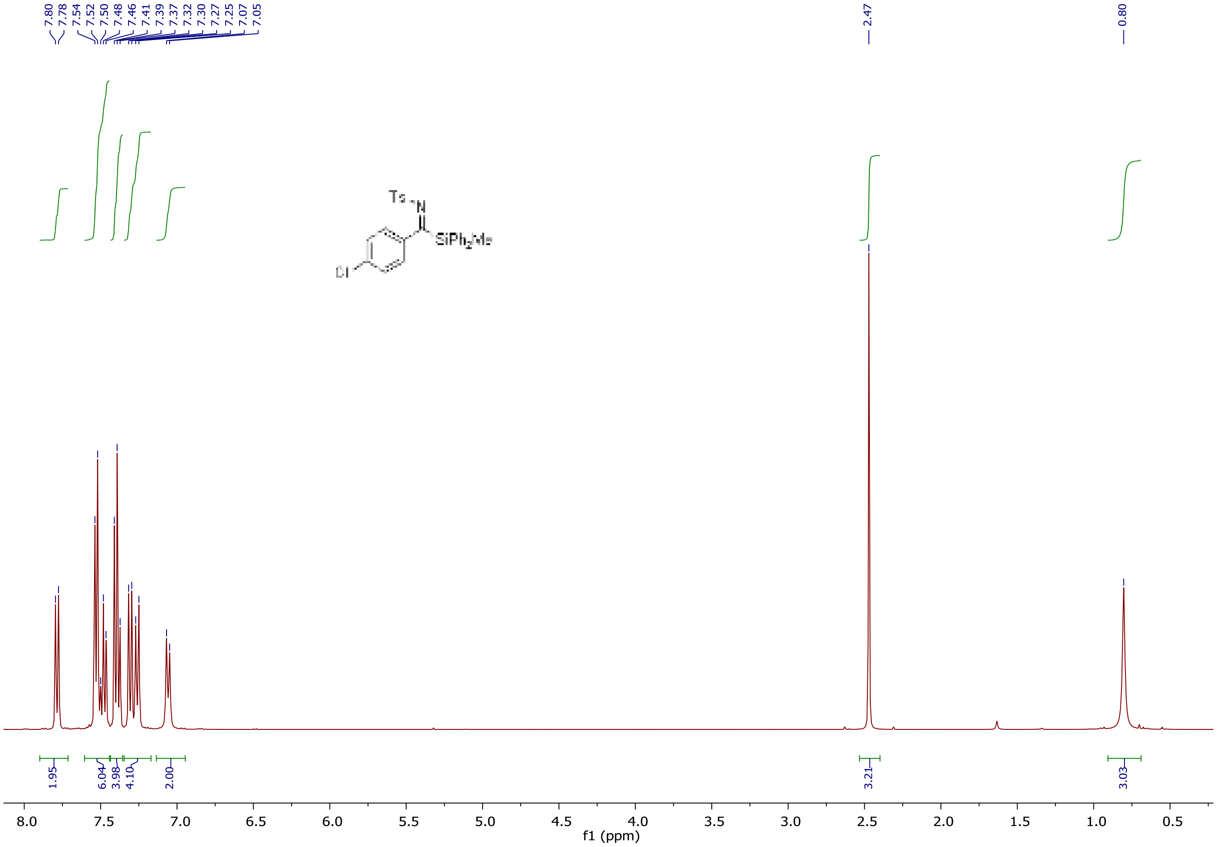

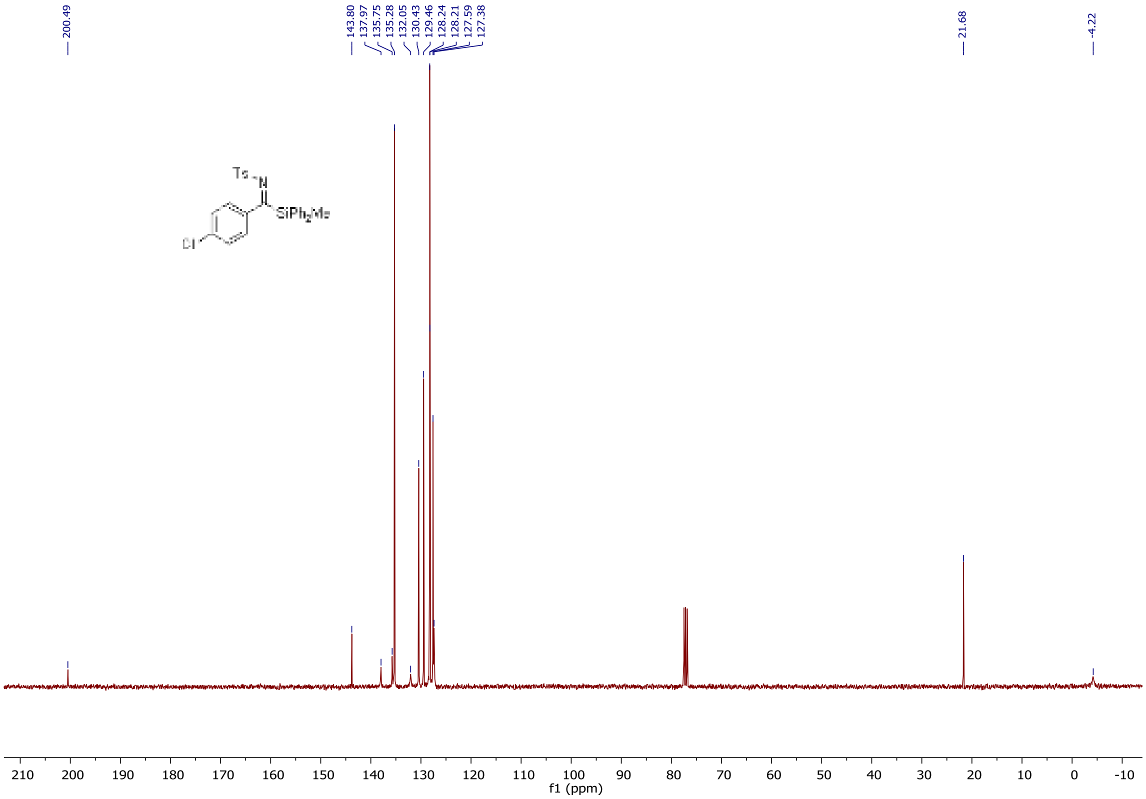

Supplementary Figure 18. 1H, 13C-NMR spectra of product **1f**.

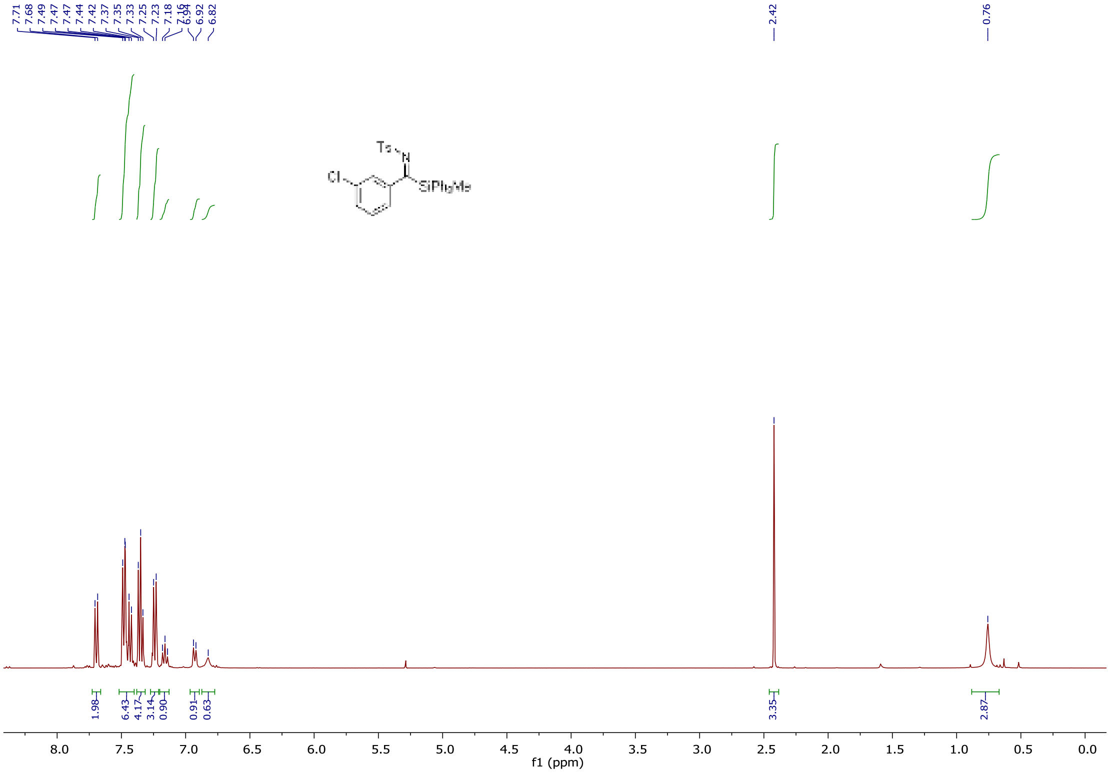

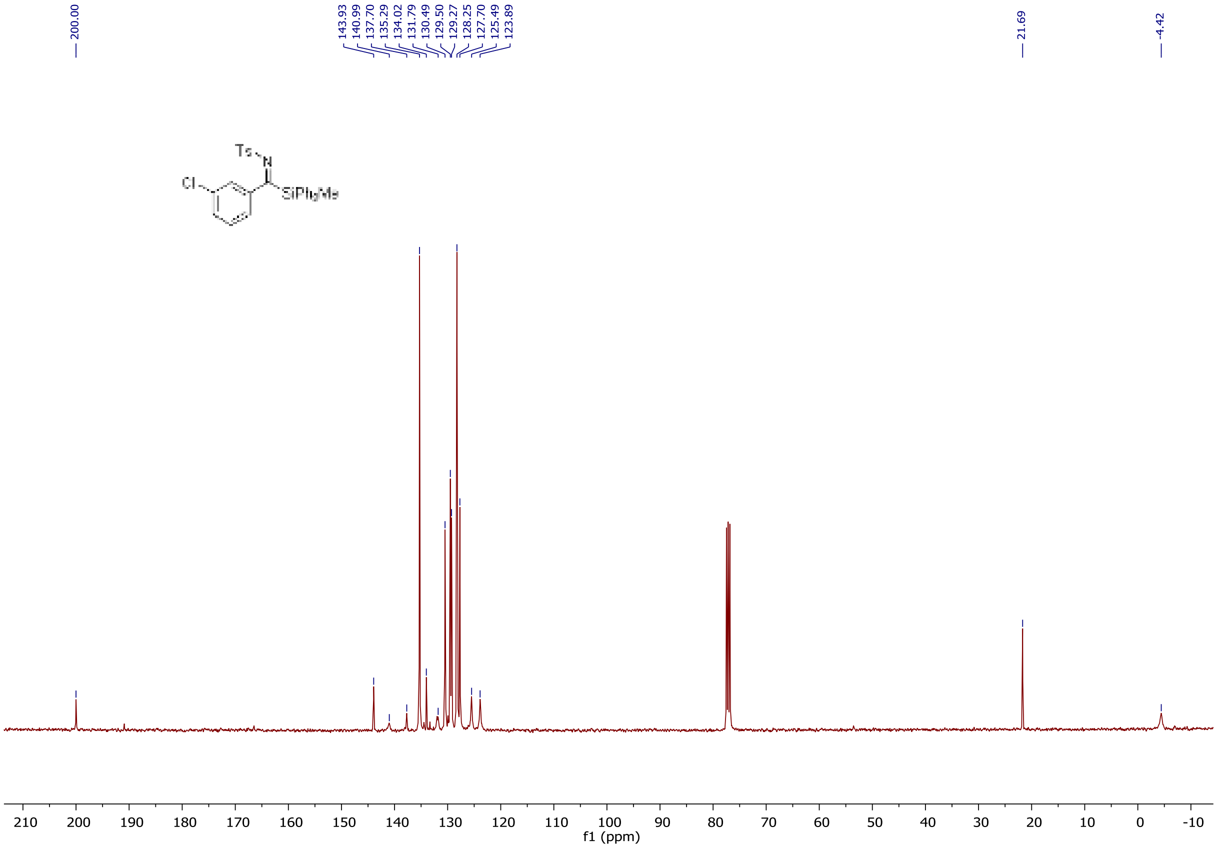

Supplementary Figure 19. 1H, 13C-NMR spectra of product **1g**.

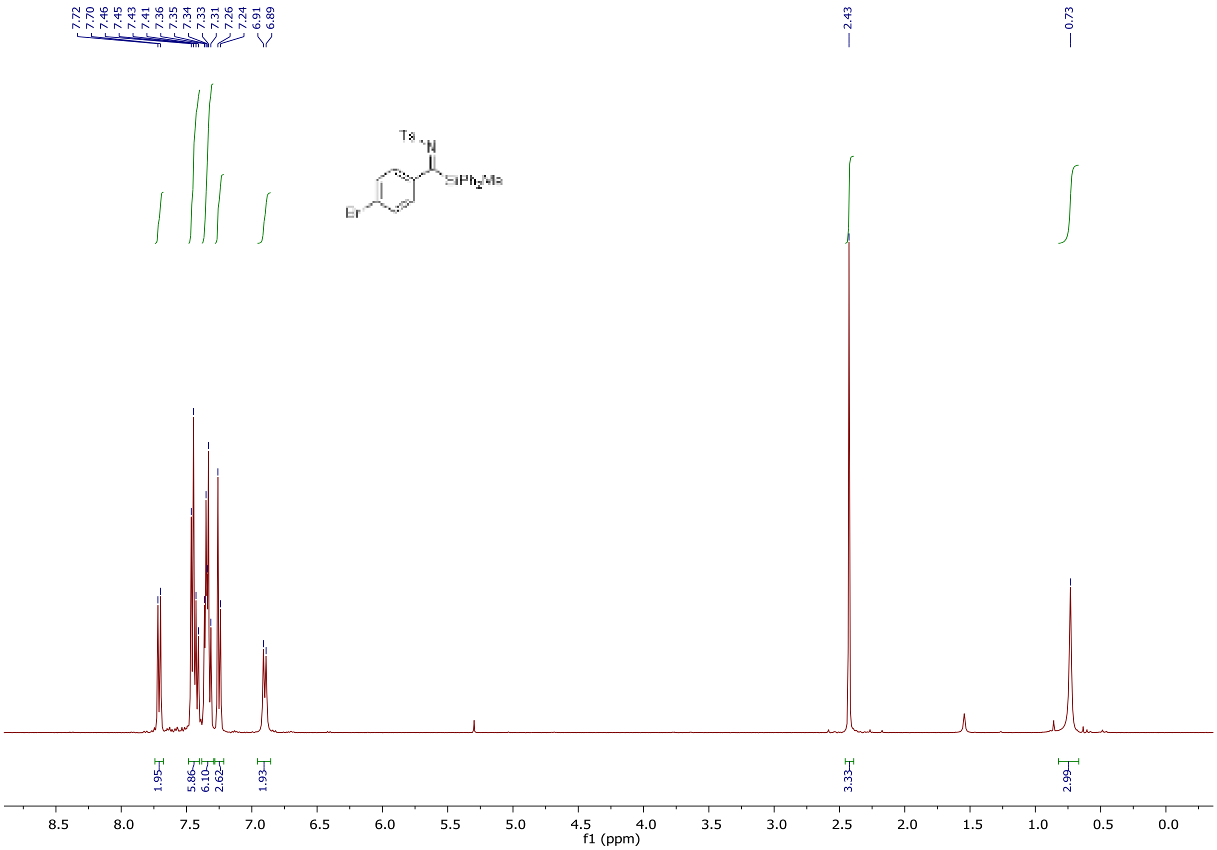

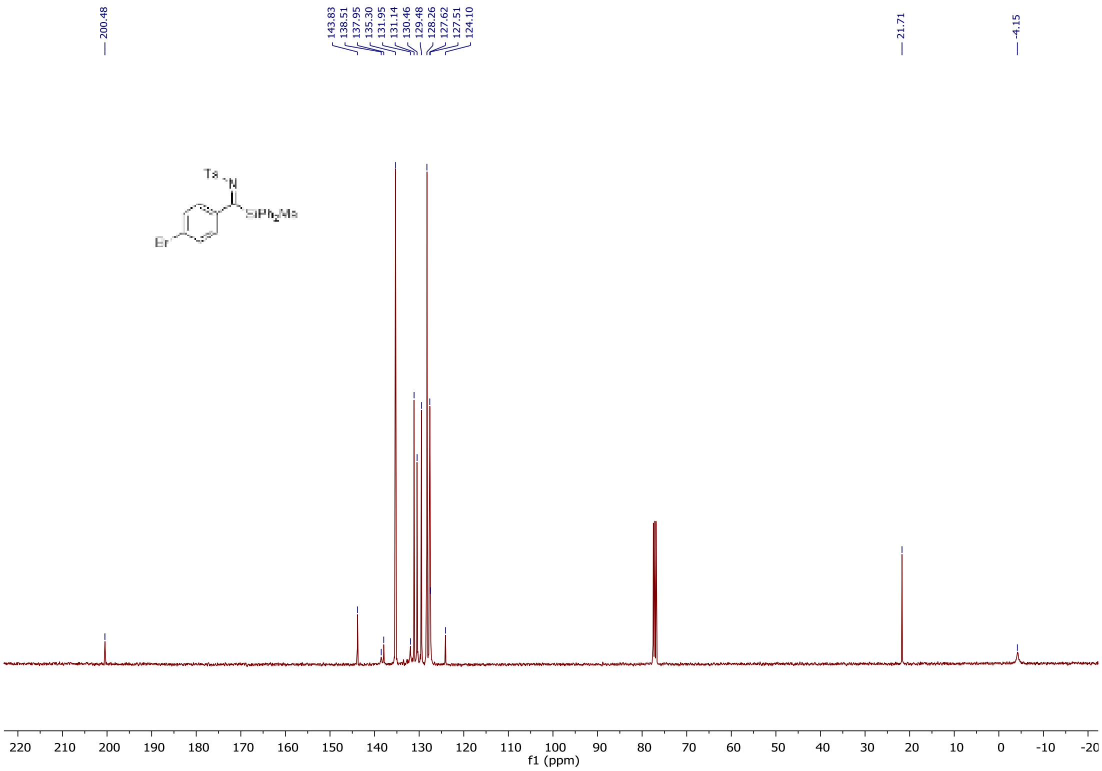

Supplementary Figure 20. 1H, 13C-NMR spectra of product **1h**.

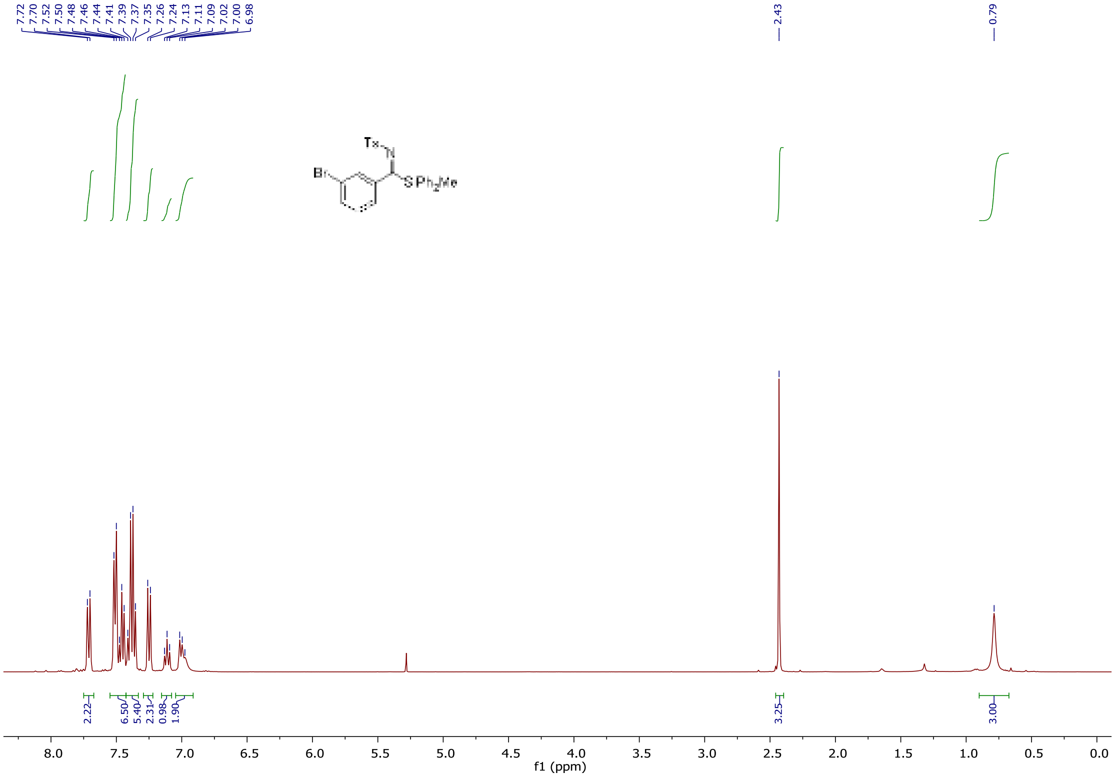

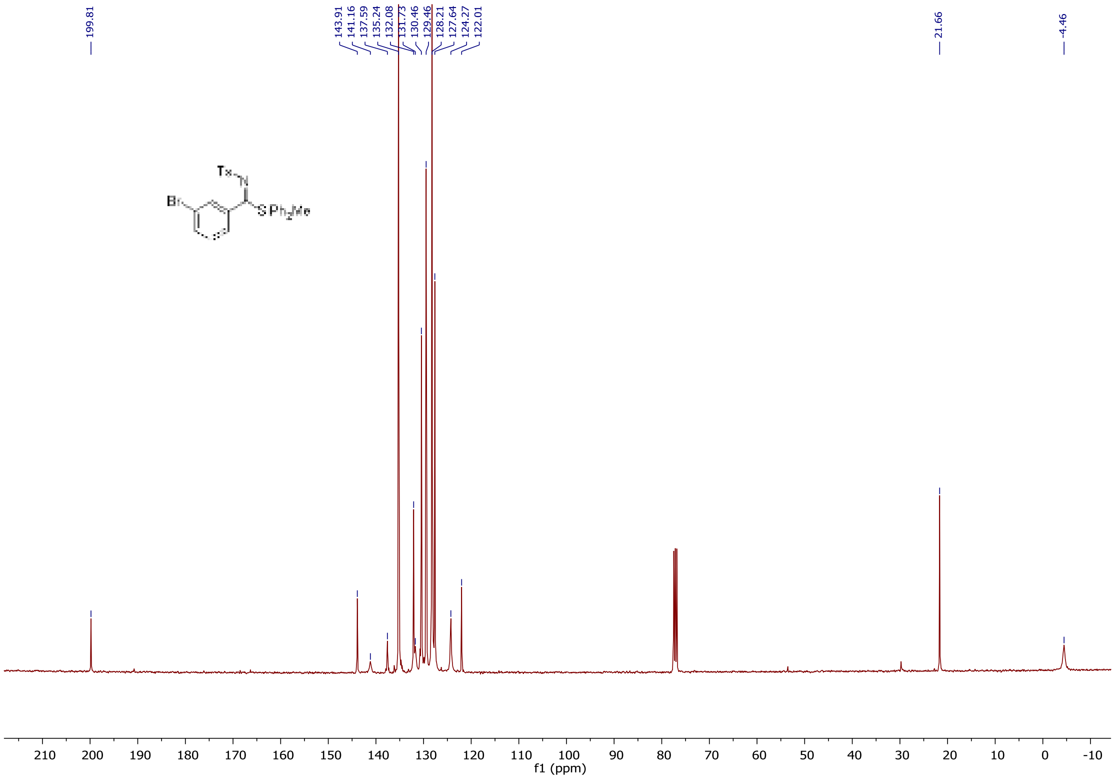

Supplementary Figure 21. 1H, 13C. 19F-NMR spectra of product **1i**.

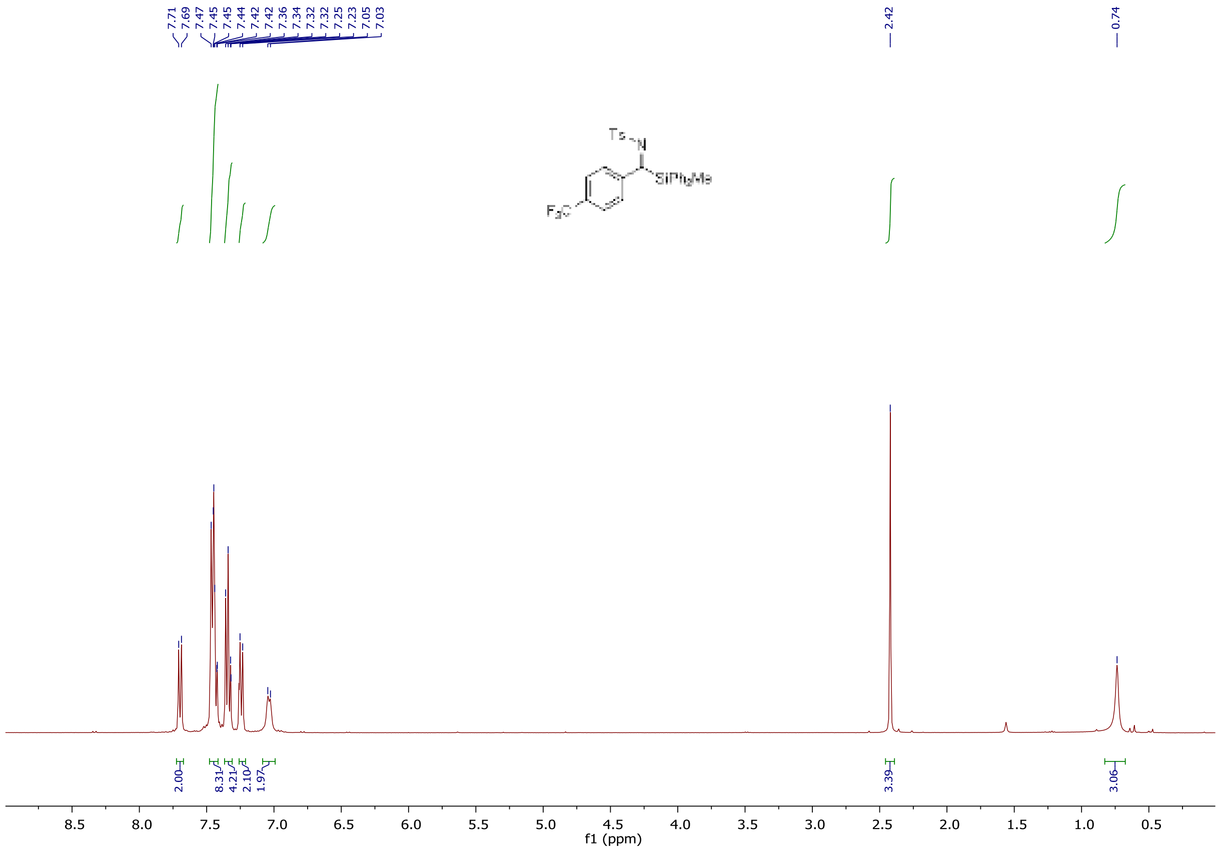

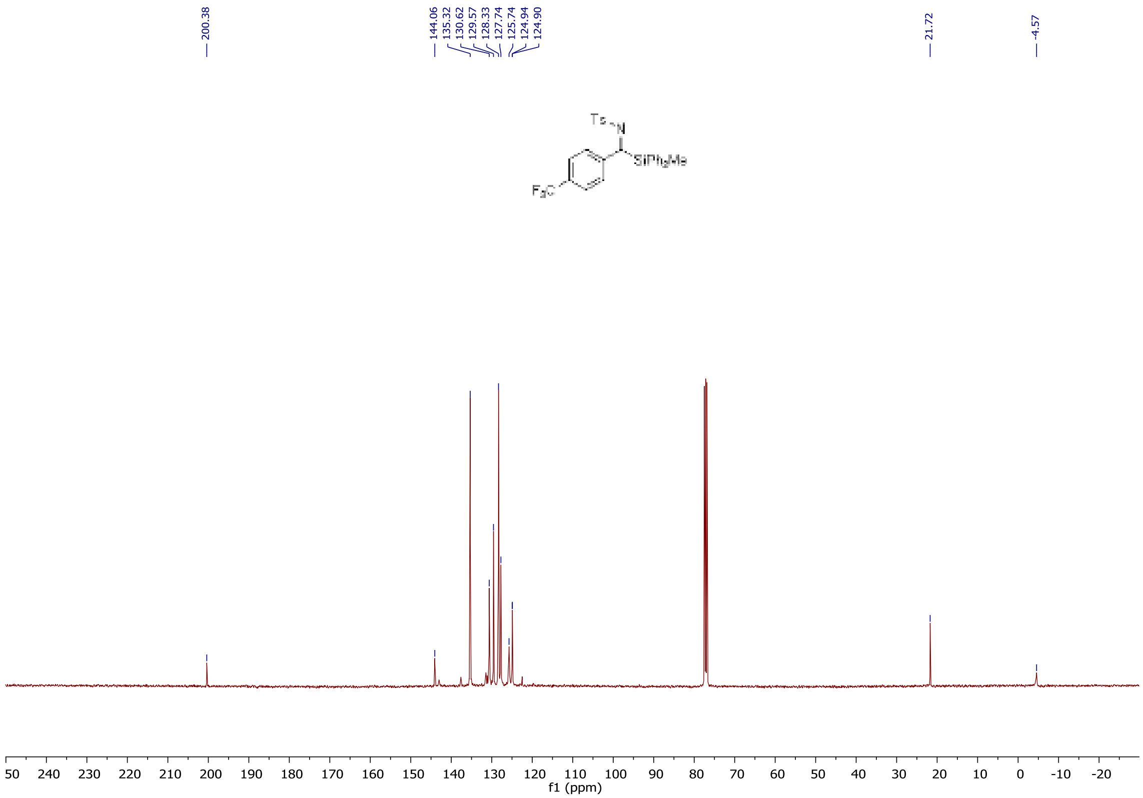

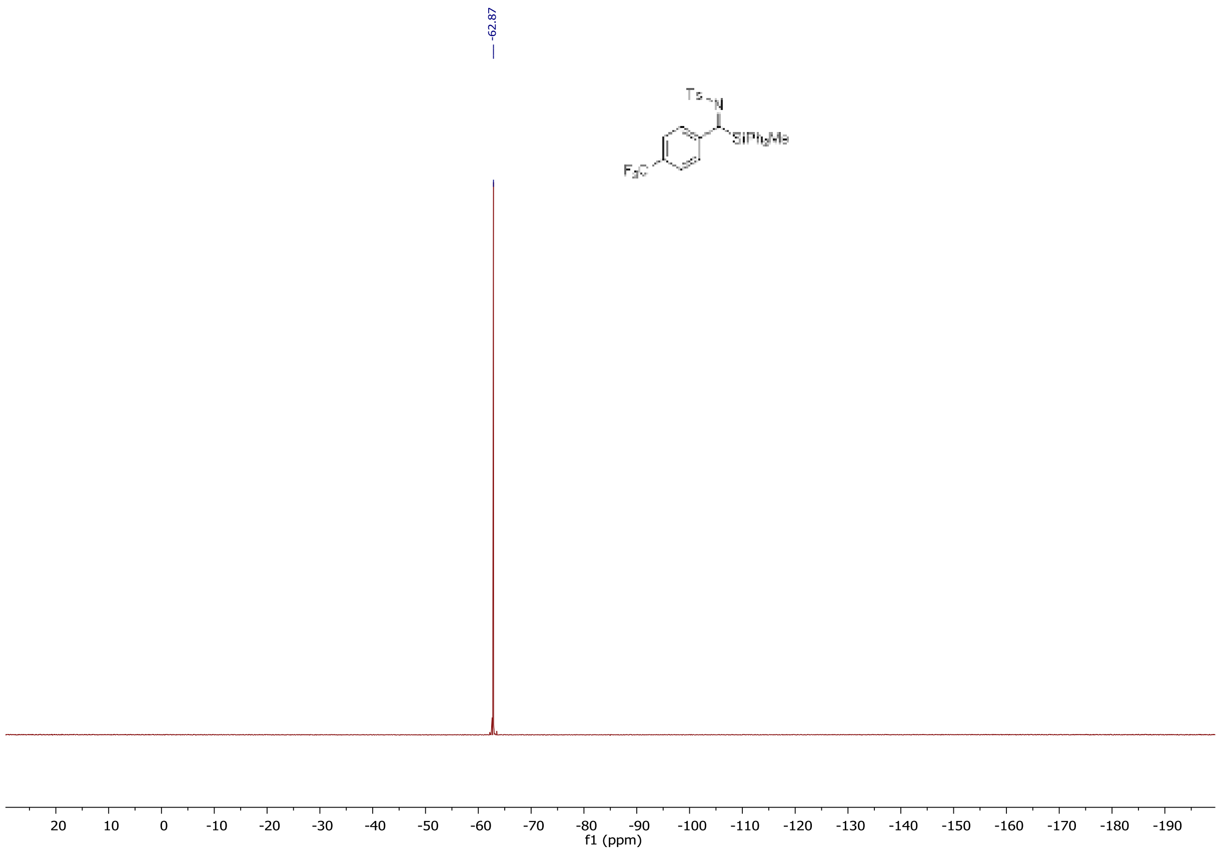

-62.87
Ts
N
SiPh2Me
F3C
20
10
0
-10
-20
-30
-40
-50
-60
-70
-80
-90
-100
-110
-120
-130
-140
-150
-160
-170
-180
-190
f1 (ppm)

Supplementary Figure 22. 1H, 13C, 19F-NMR spectra of product **1j**.

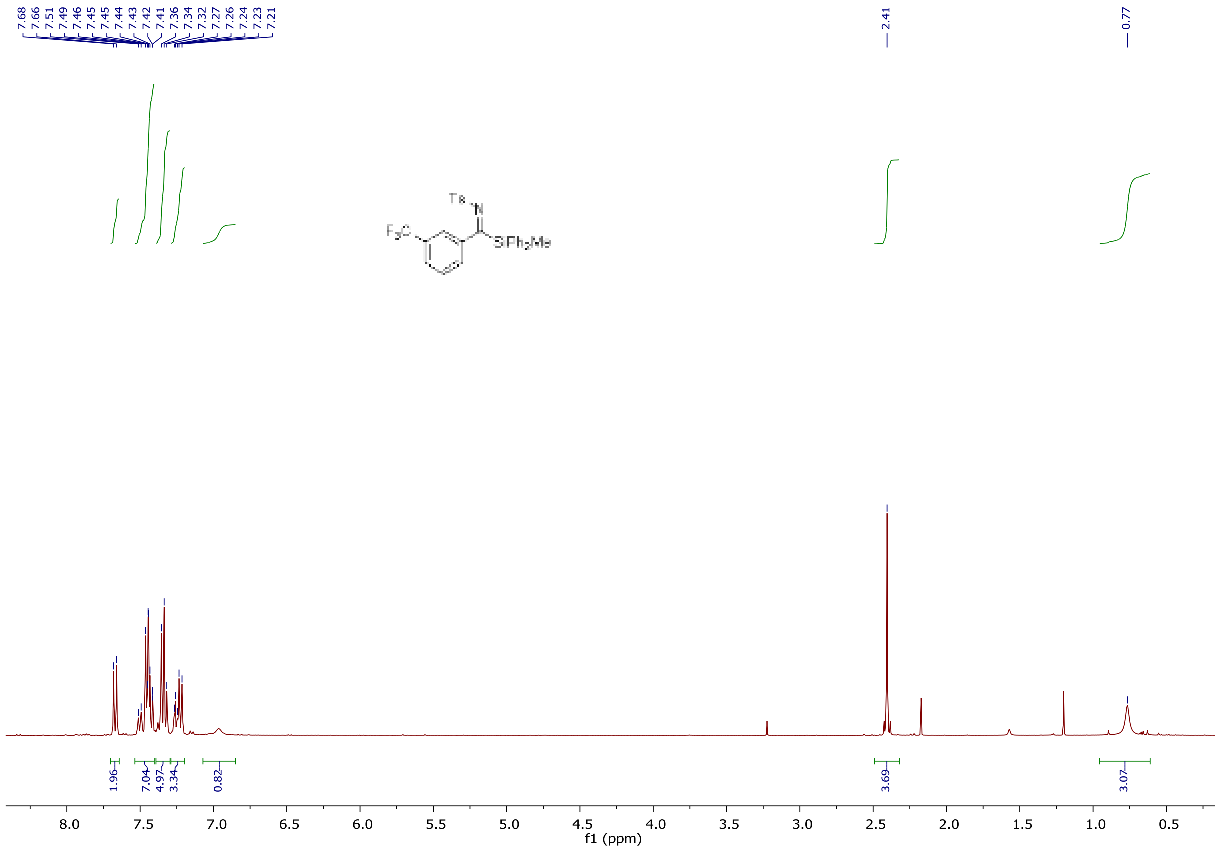

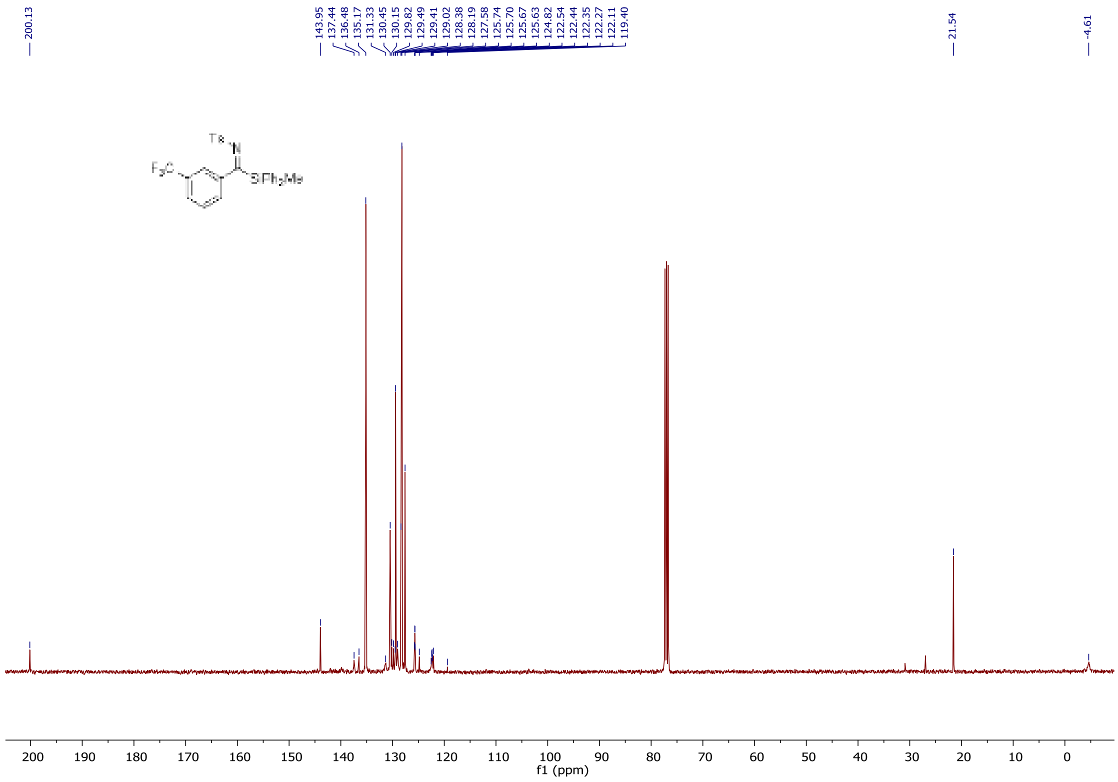

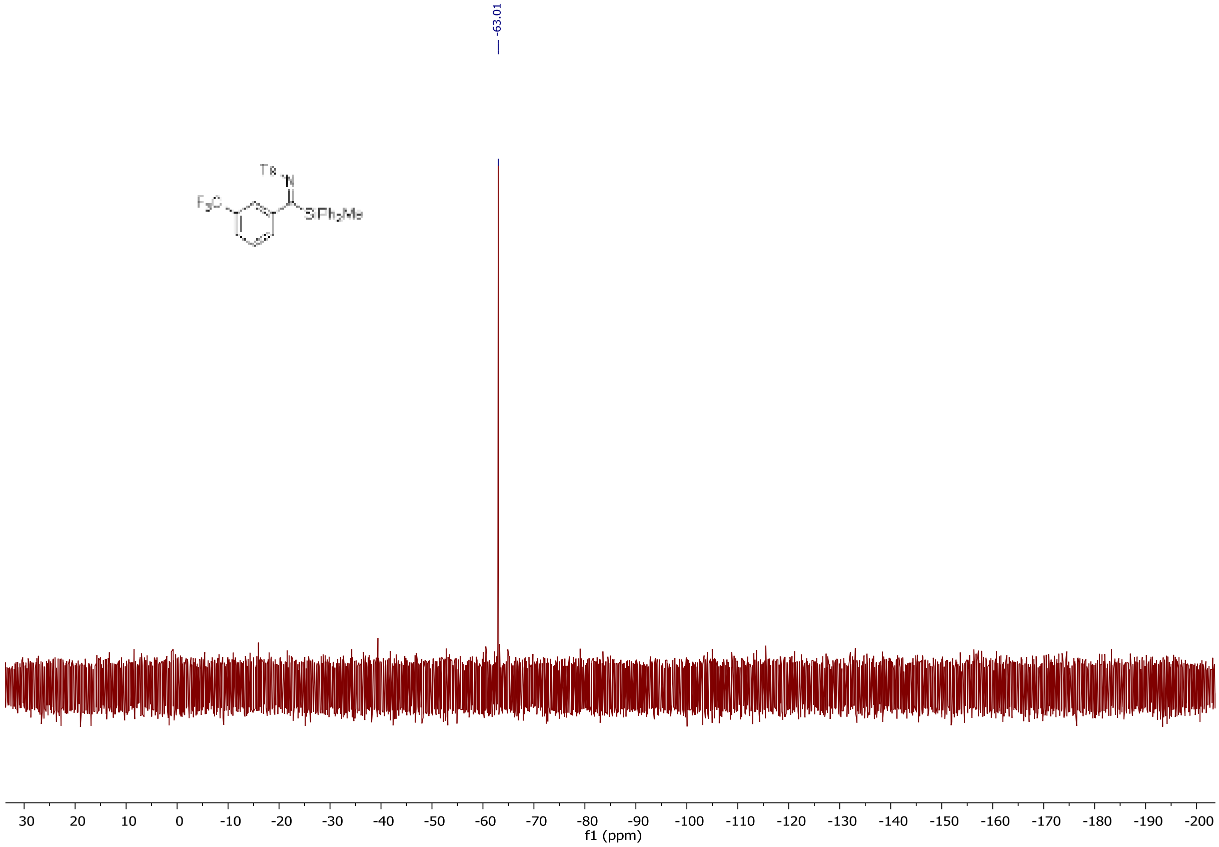
-63.01
30
20
10
0
-10
-20
-30
-40
-50
-60
-70
-80
-90
-100
-110
-120
-130
-140
-150
-160
-170
-180
-190
-200
f1 (ppm)

Supplementary Figure 23. 1H, 13C-NMR spectra of product **1k**.

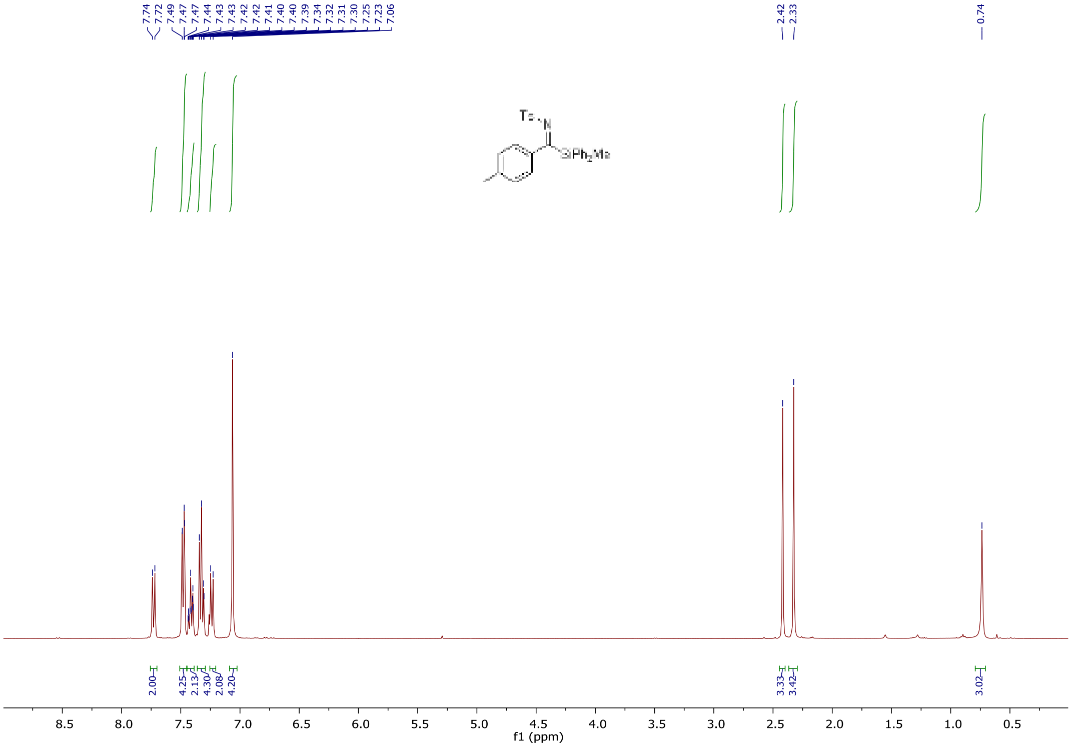

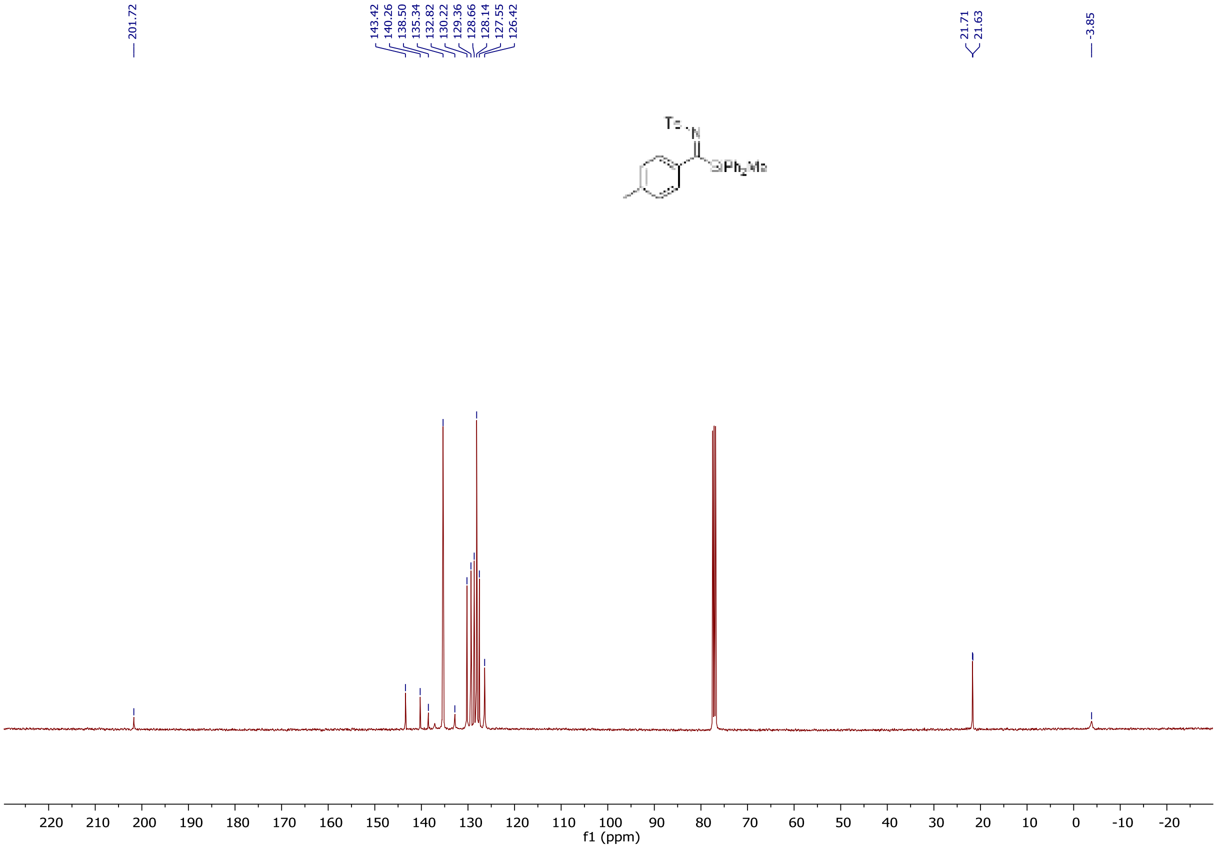

Supplementary Figure 24. 1H, 13C-NMR spectra of product **1l**.

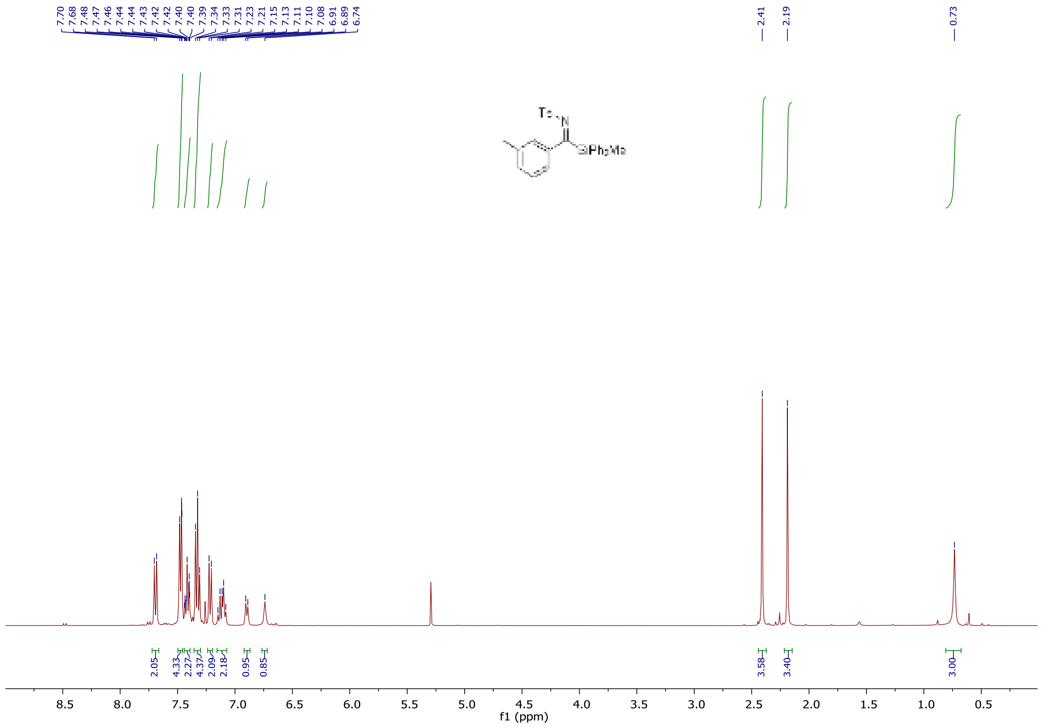

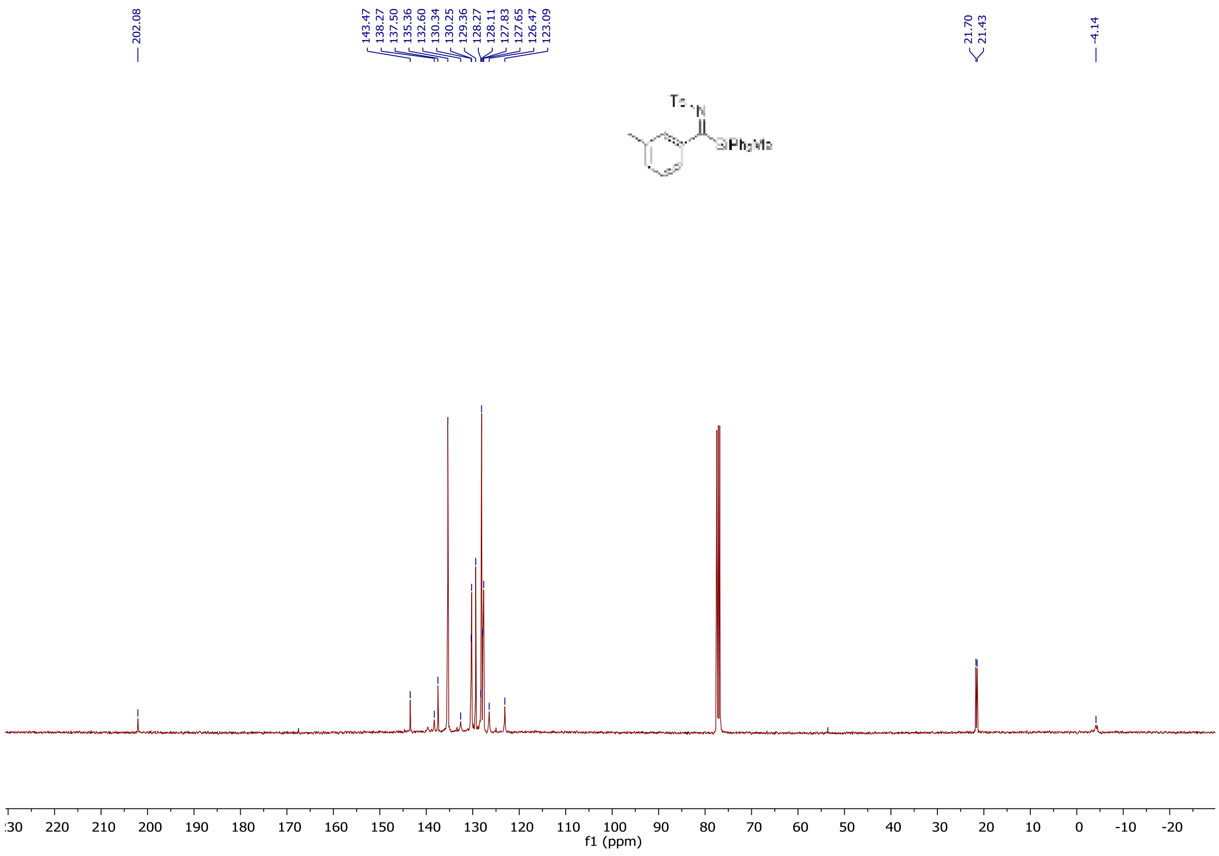

Supplementary Figure 25. 1H, 13C-NMR spectra of product **1m**.

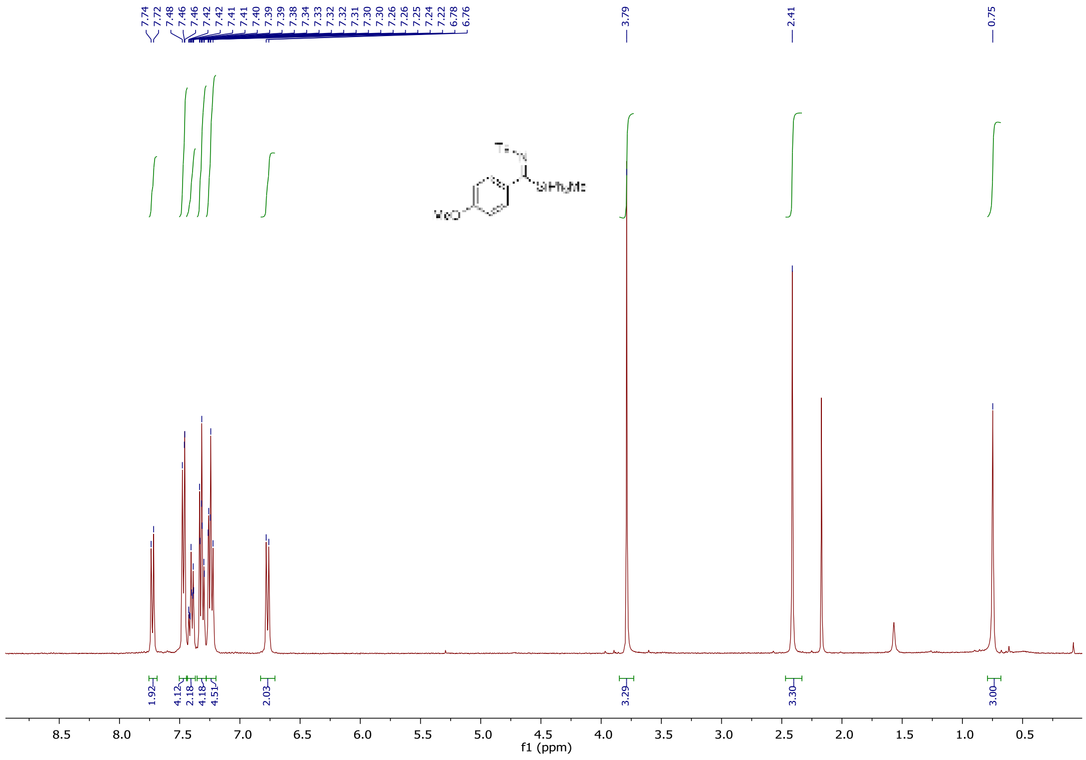

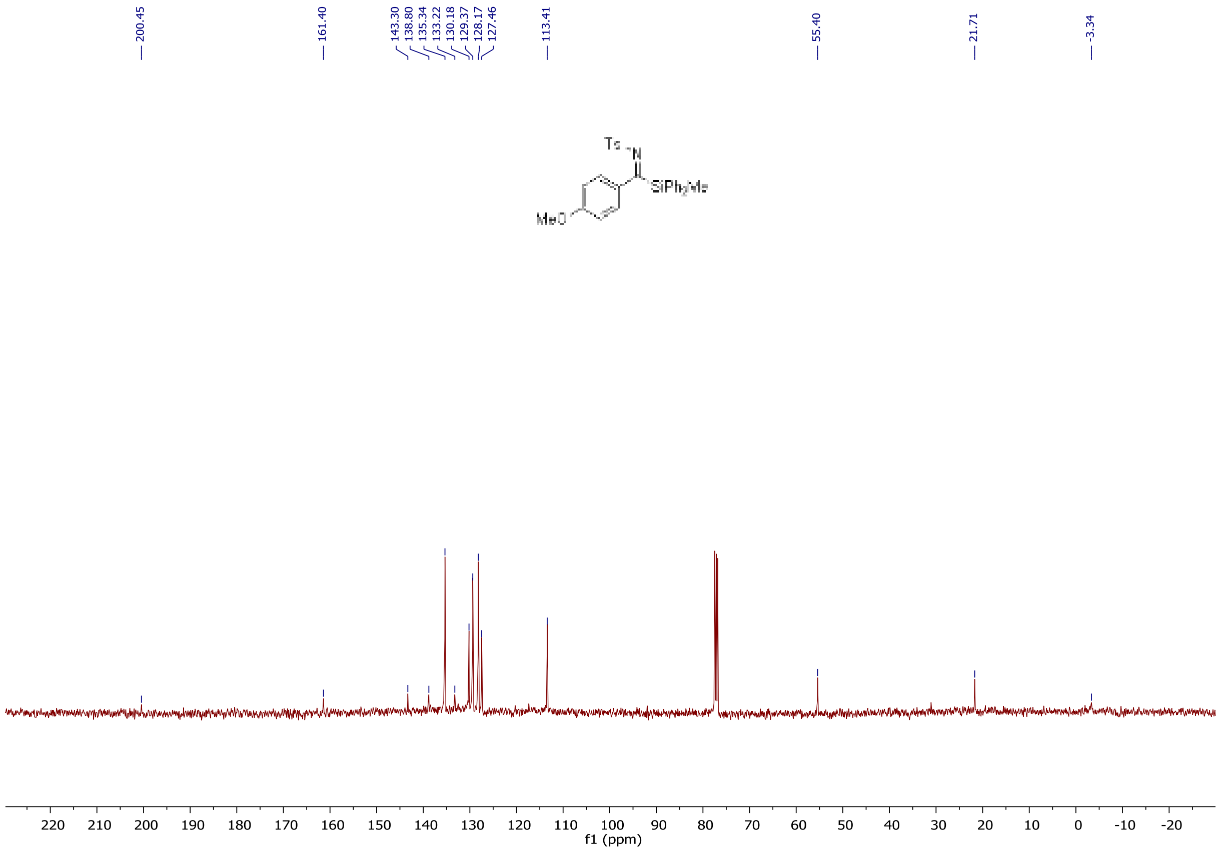

Supplementary Figure 26. 1H, 13C-NMR spectra of product **1n**.

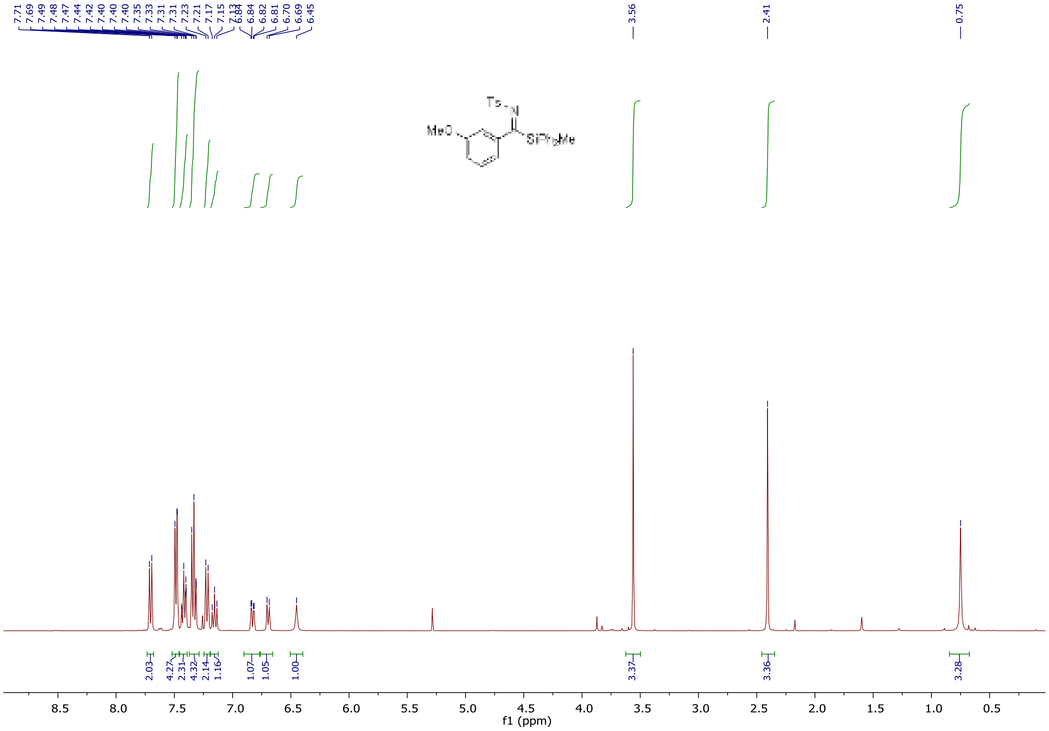

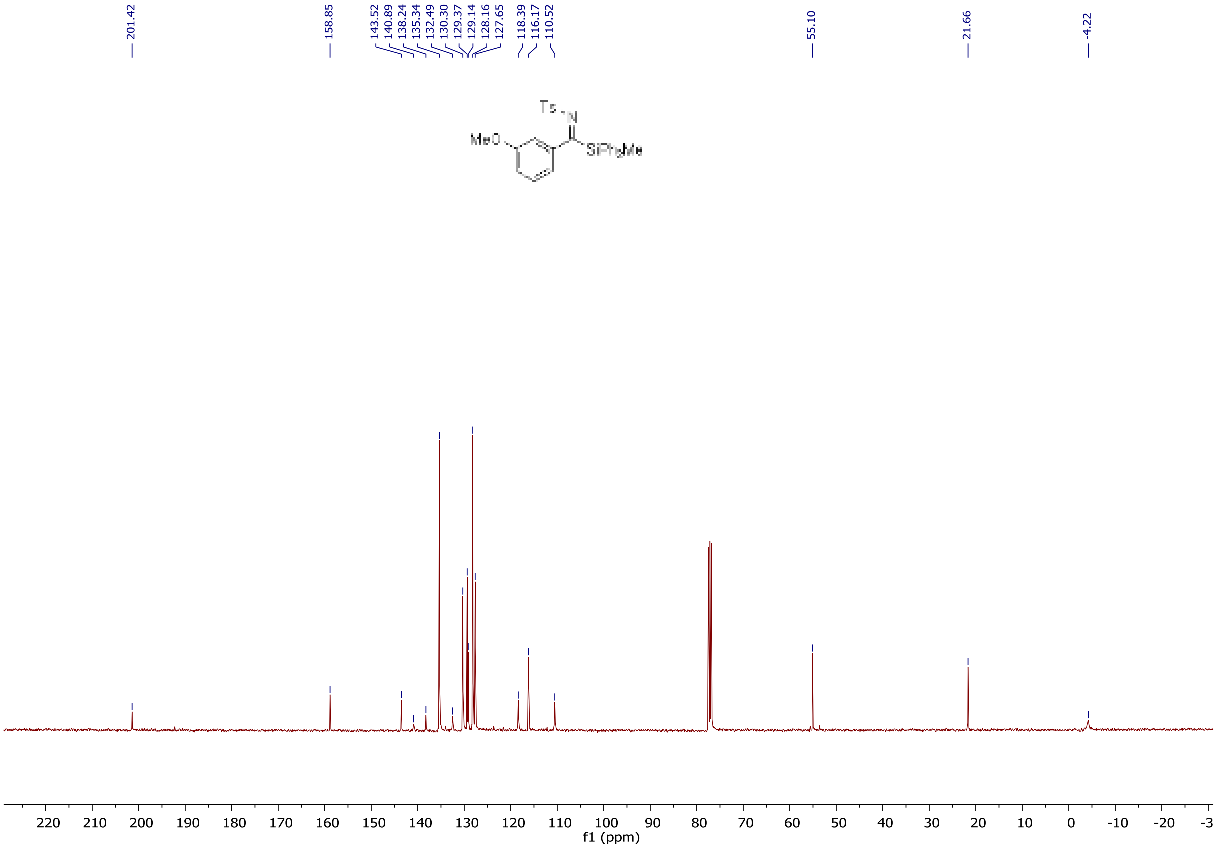

Supplementary Figure 27. 1H, 13C-NMR spectra of product **1o**.

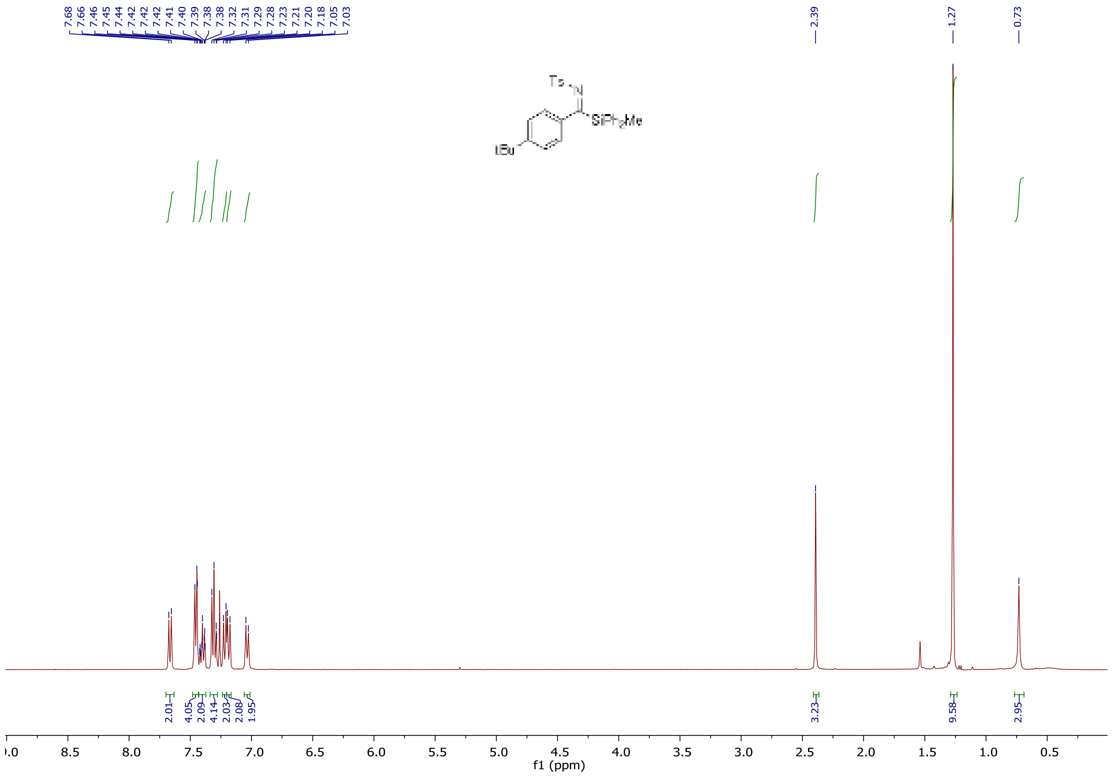

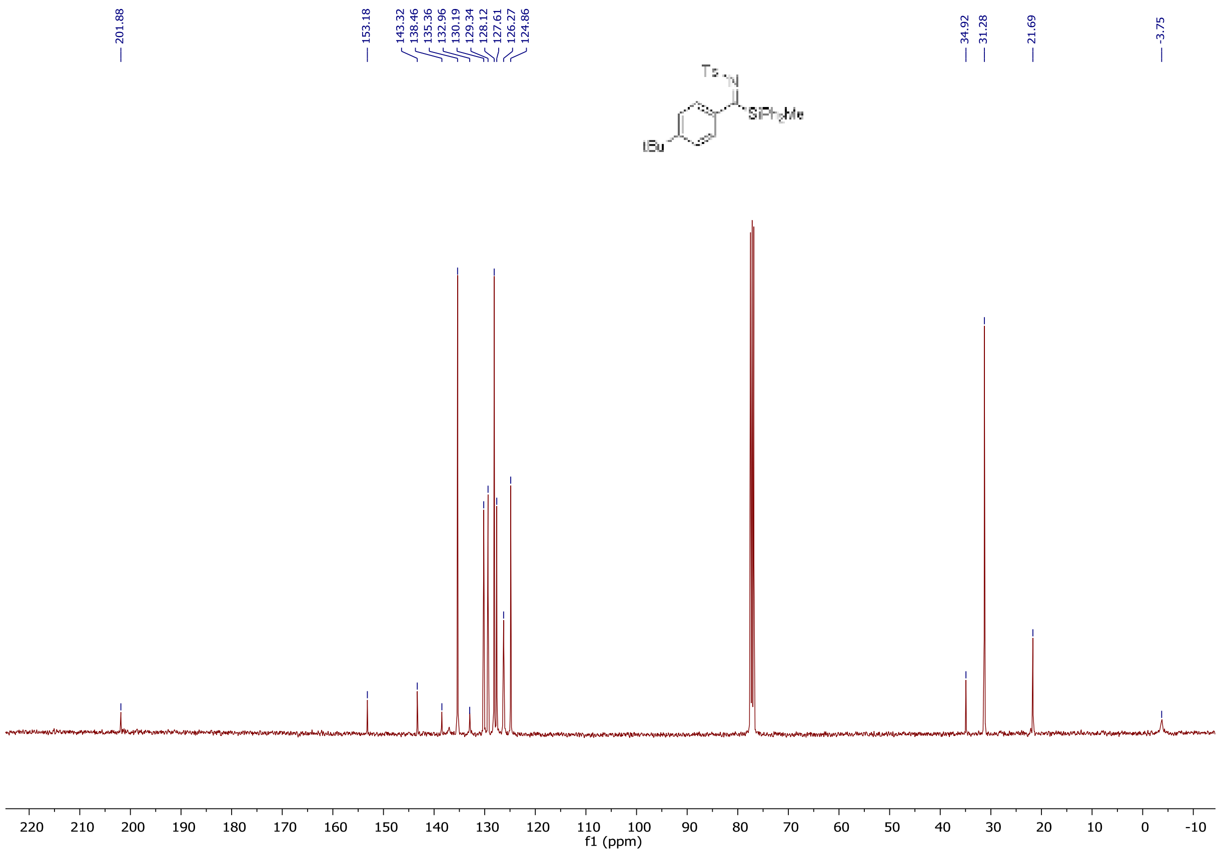

Supplementary Figure 28. 1H, 13C, 19-F-NMR spectra of product **1p**.

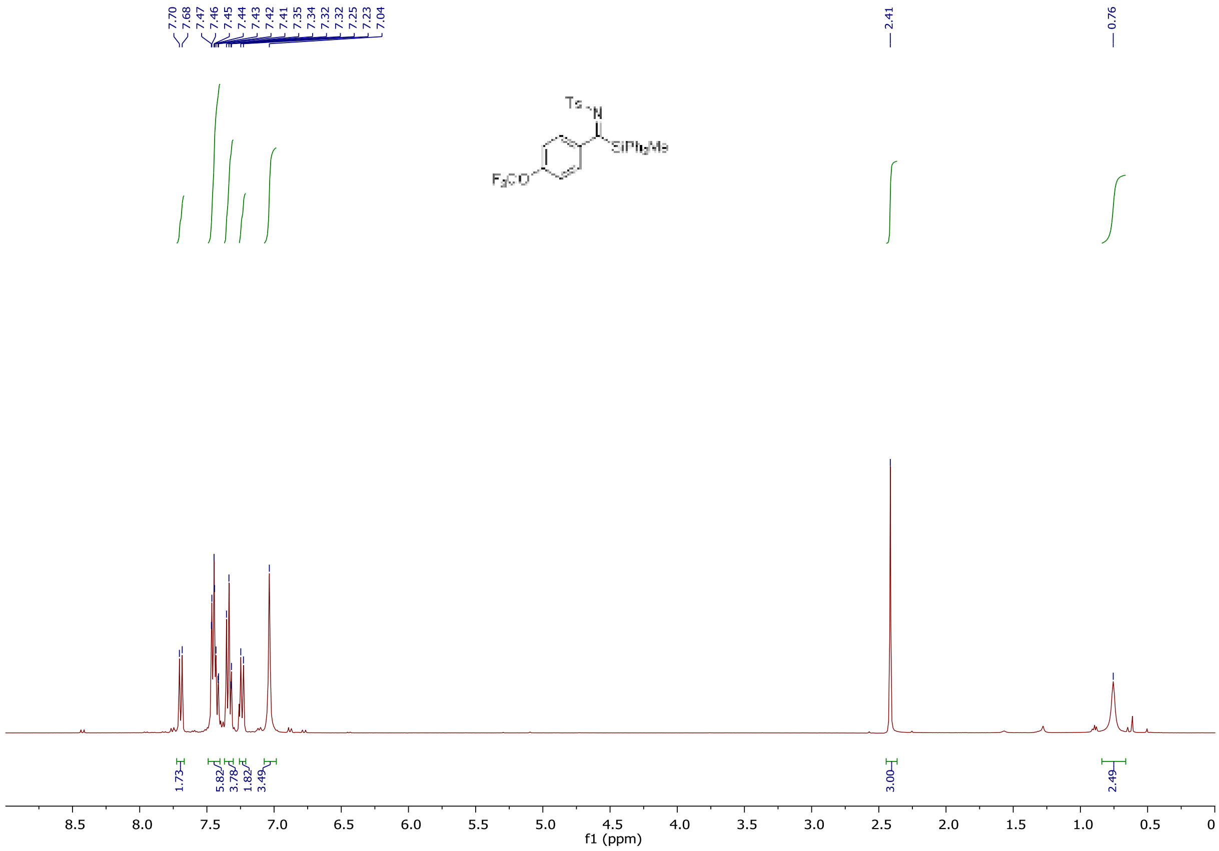

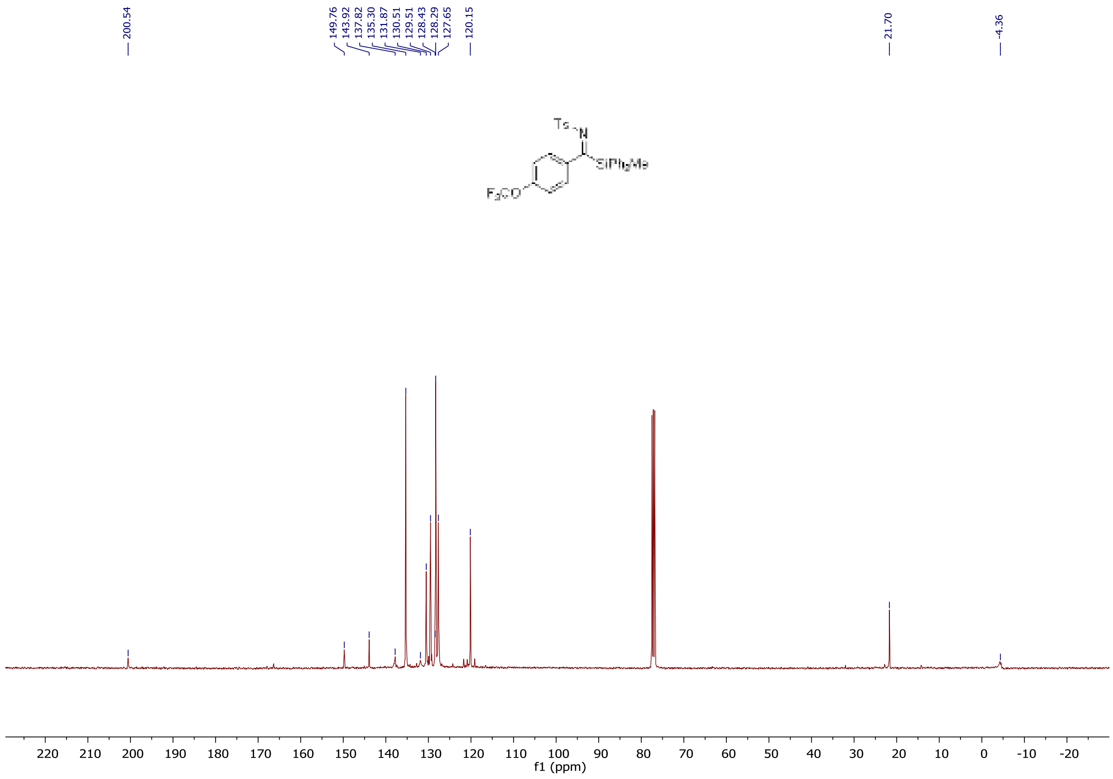

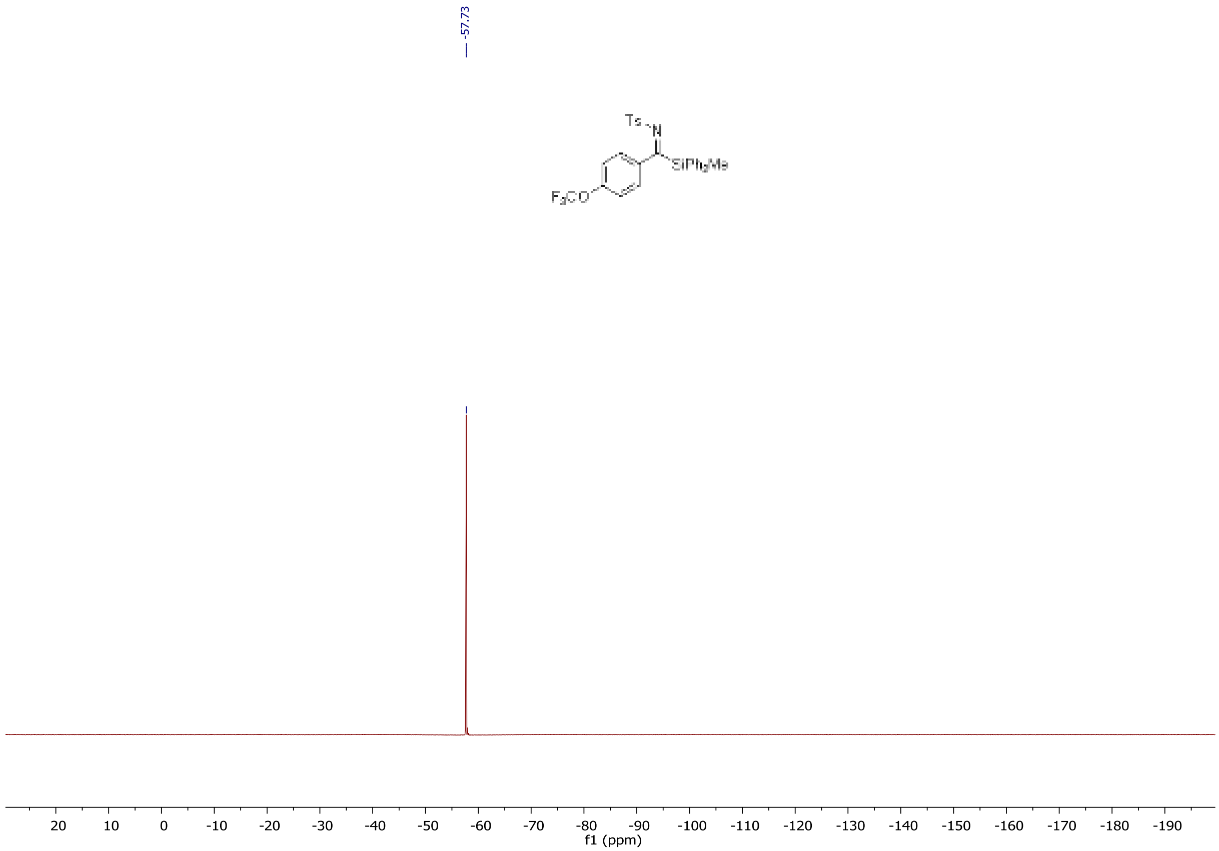
-57.73
Ts
N
SiPh2Me
F3CO
20
10
0
-10
-20
-30
-40
-50
-60
-70
-80
-90
-100
-110
-120
-130
-140
-150
-160
-170
-180
-190
f1 (ppm)

Supplementary Figure 29. 1H, 13C, 19-F-NMR spectra of product **1q**.

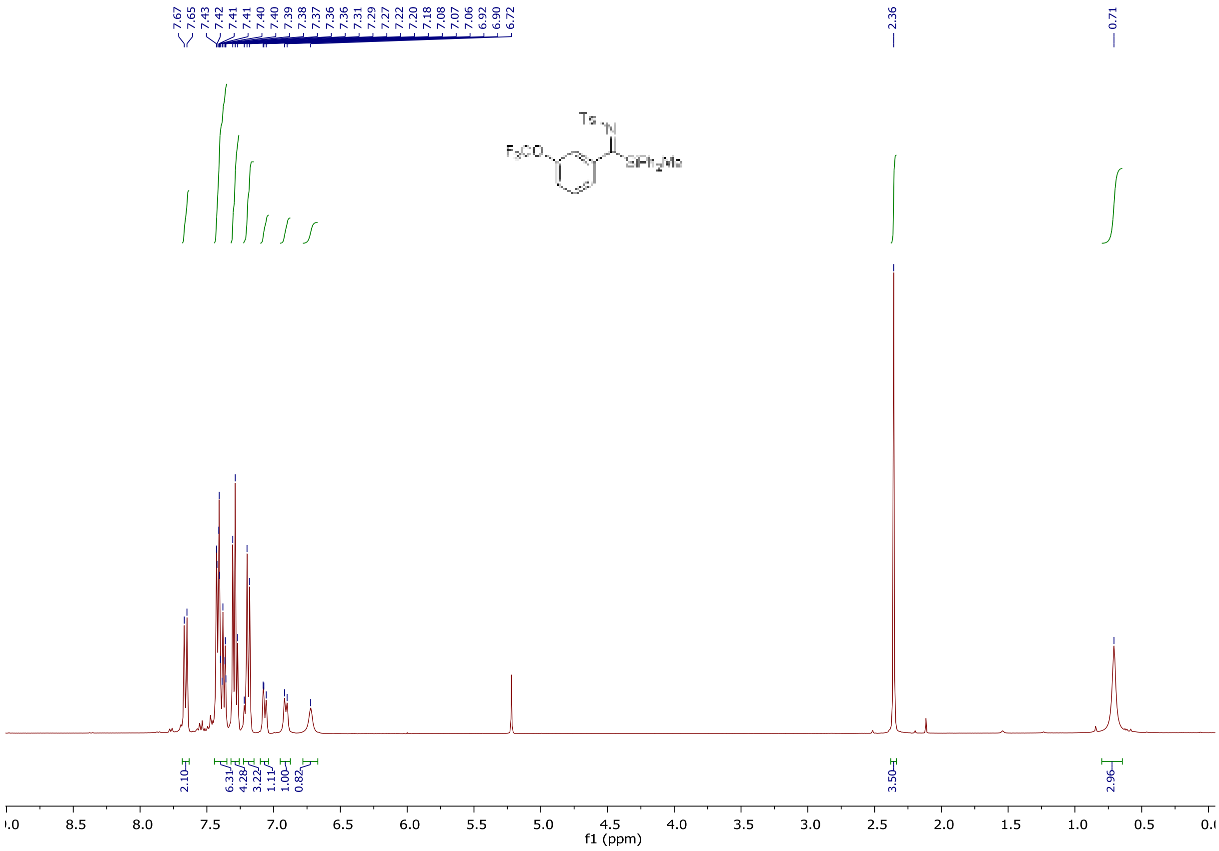

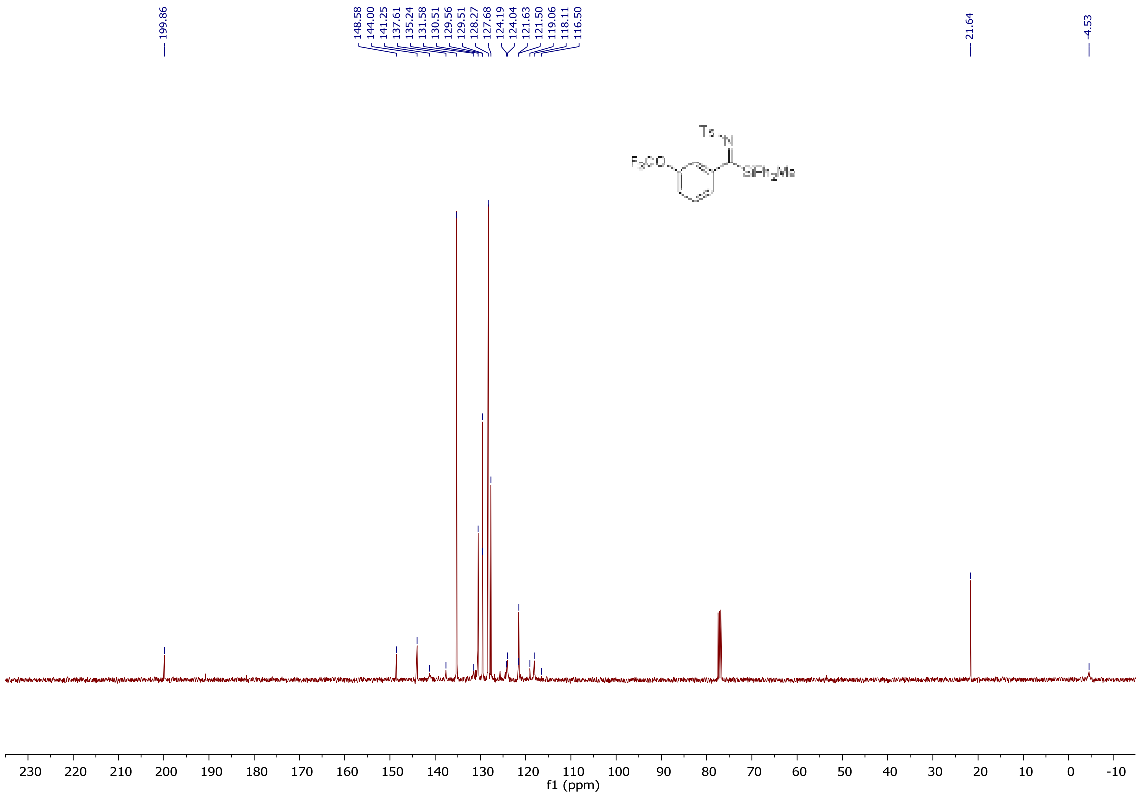

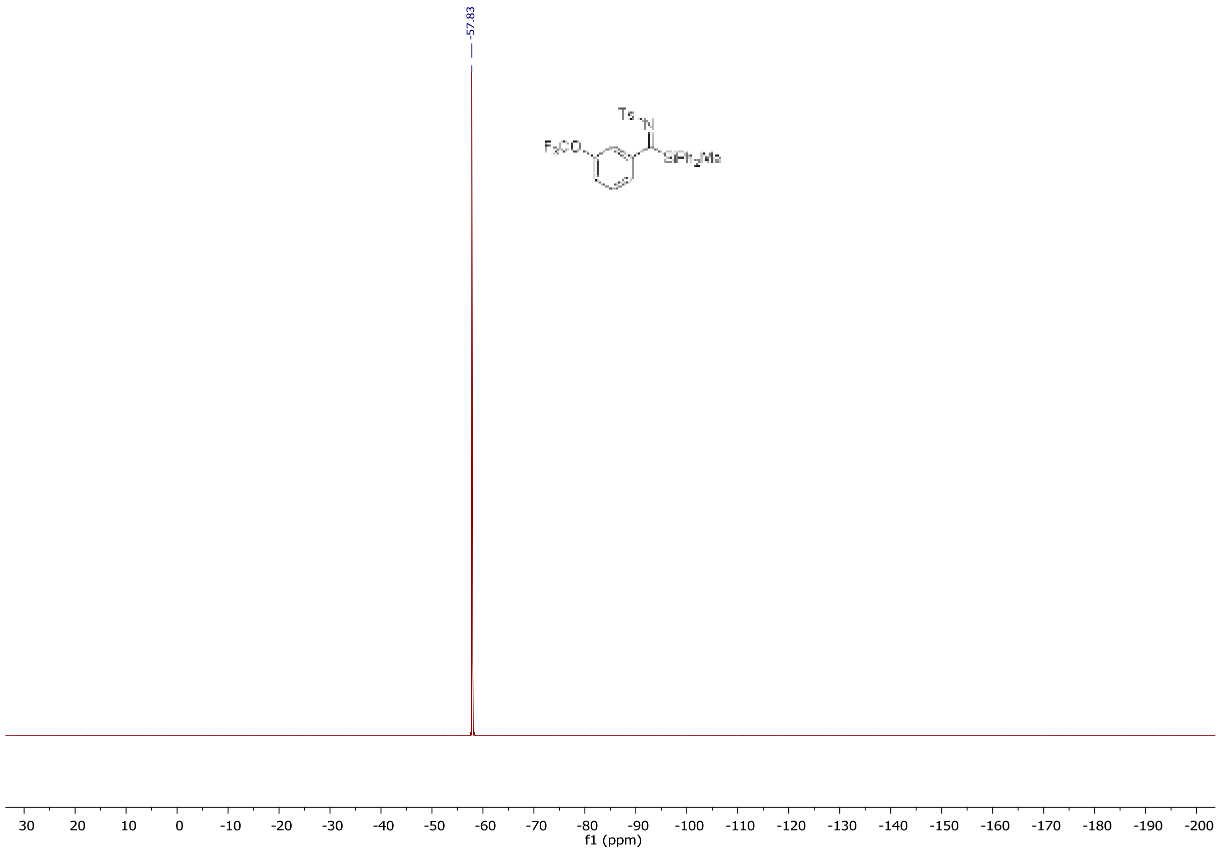

-57.83
Ts
N
F3CO
SiPh2Me
30
20
10
0
-10
-20
-30
-40
-50
-60
-70
-80
-90
-100
-110
-120
-130
-140
-150
-160
-170
-180
-190
-200
f1 (ppm)

Supplementary Figure 30. 1H, 13C-NMR spectra of product **1r**.

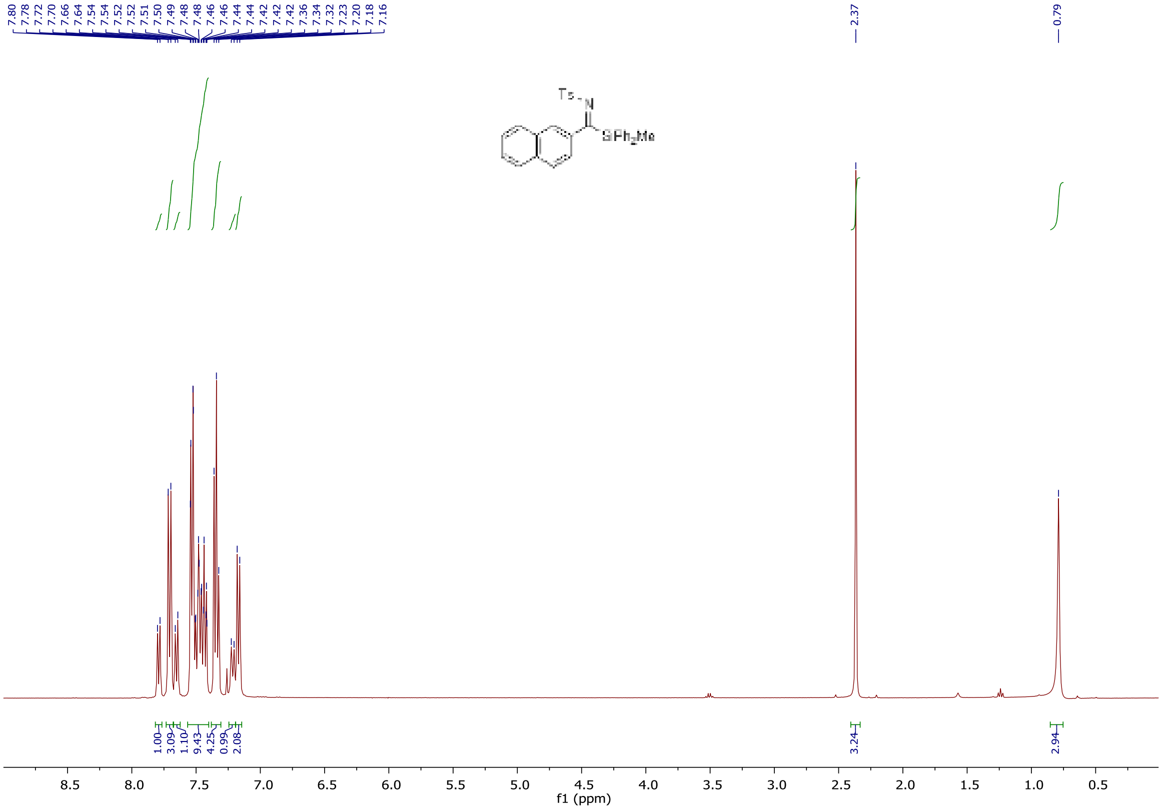

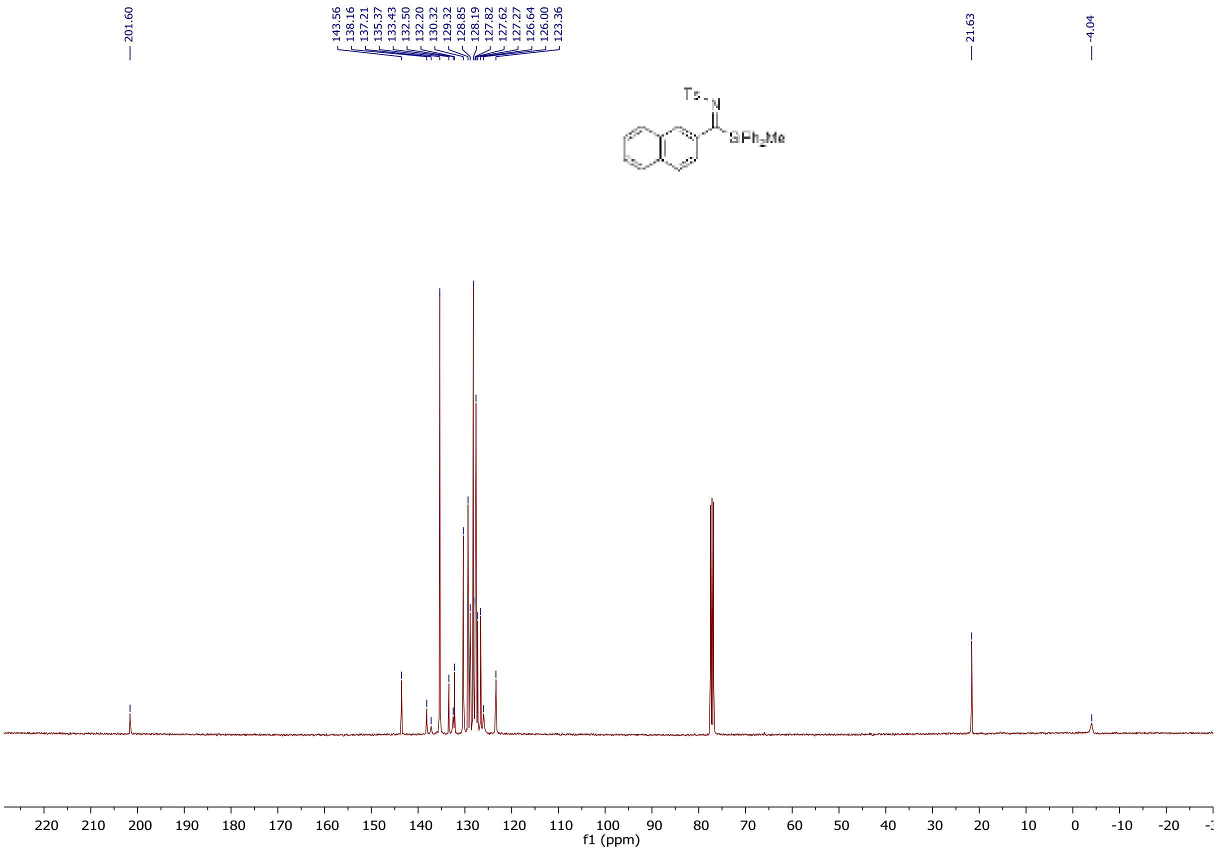

Supplementary Figure 31. 1H, 13C-NMR spectra of product **1s**.

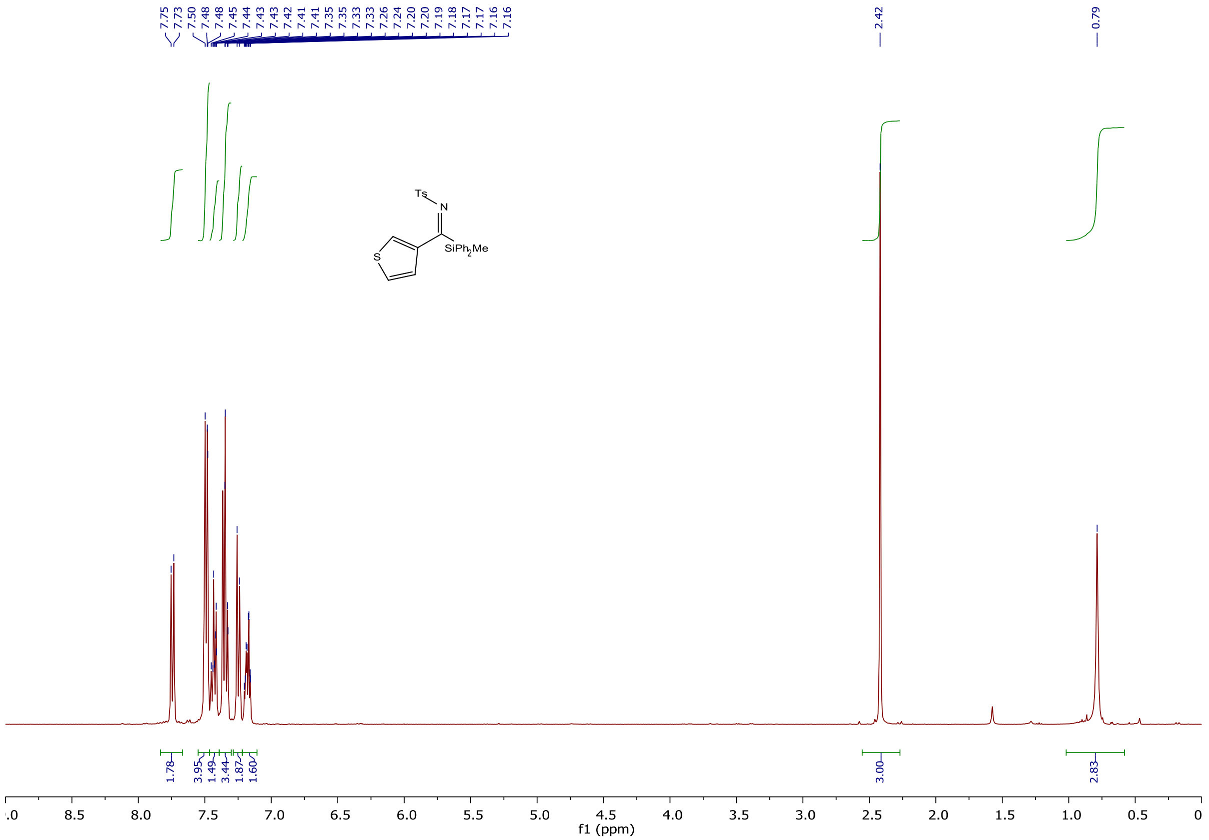

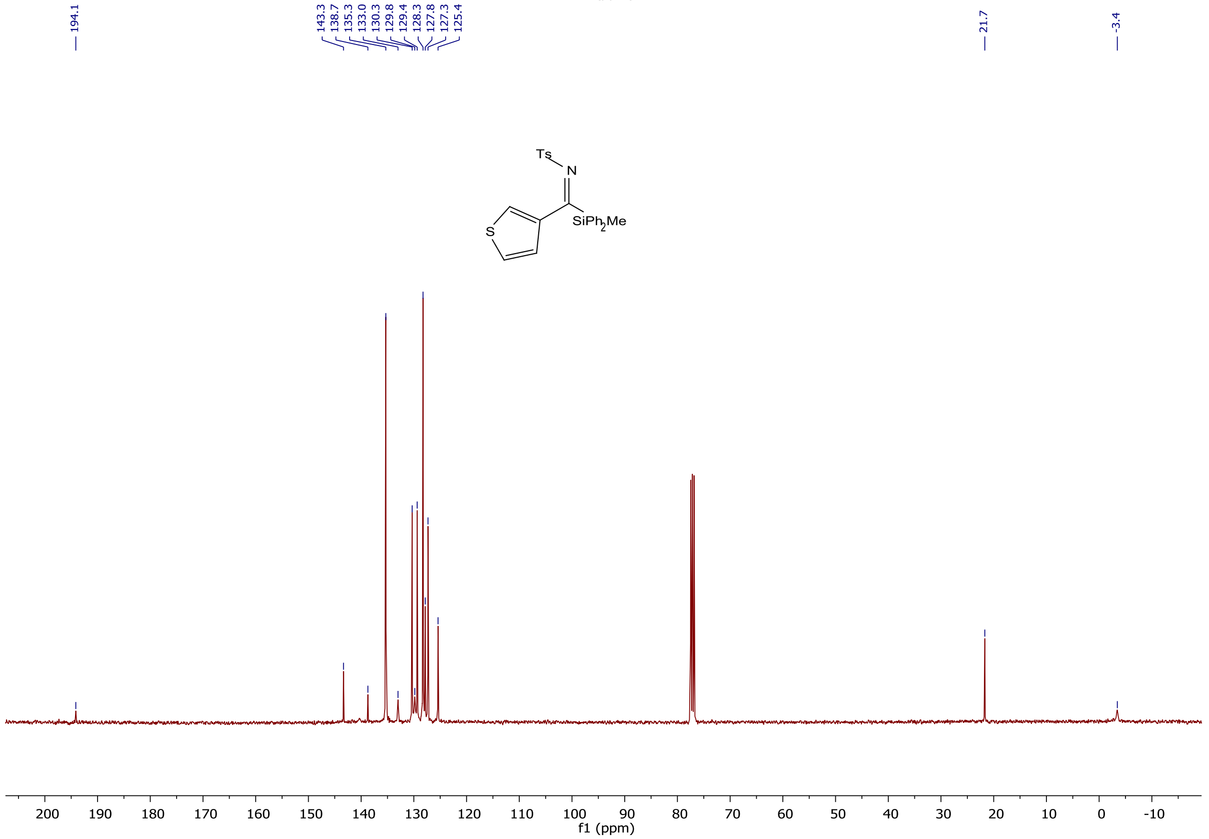

Supplementary Figure 32. 1H, 13C-NMR spectra of product **1t.**

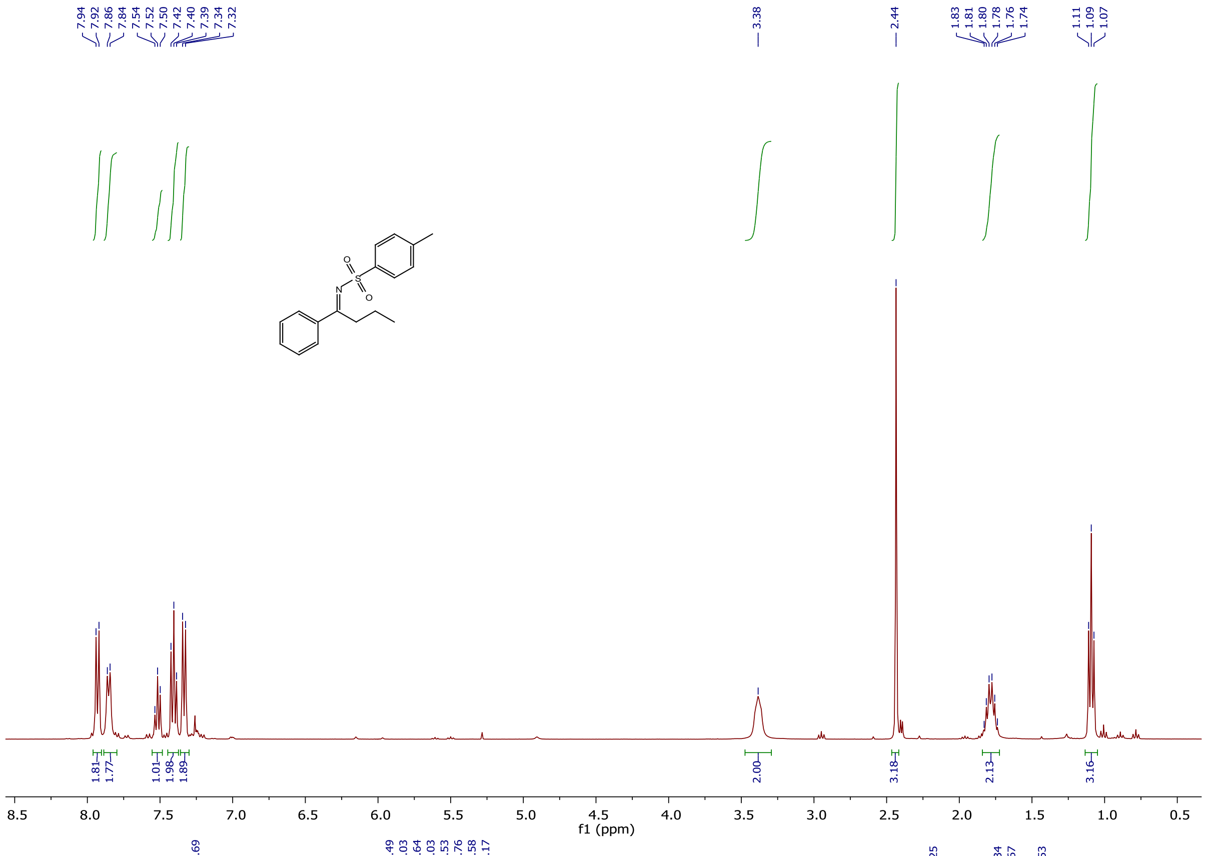

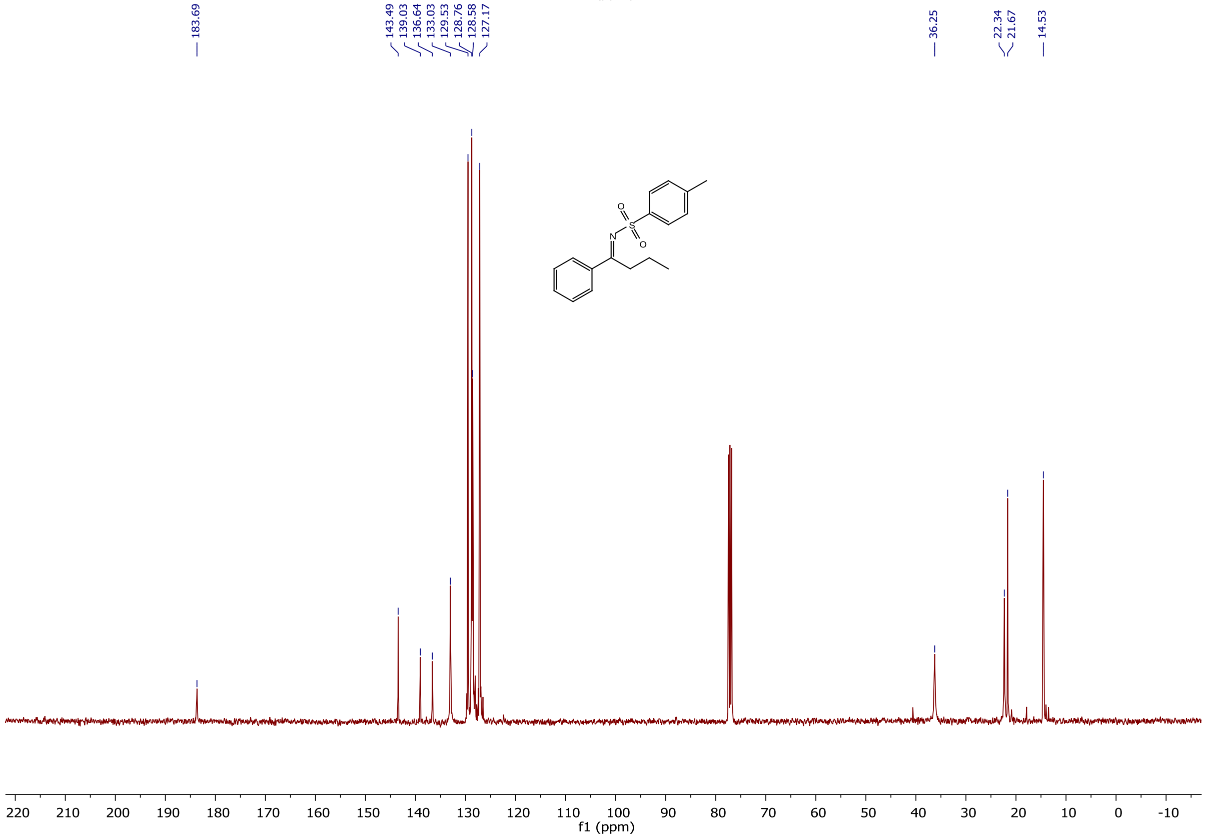

Supplementary Figure 33. 1H, 13C-NMR spectra of product **4b**.

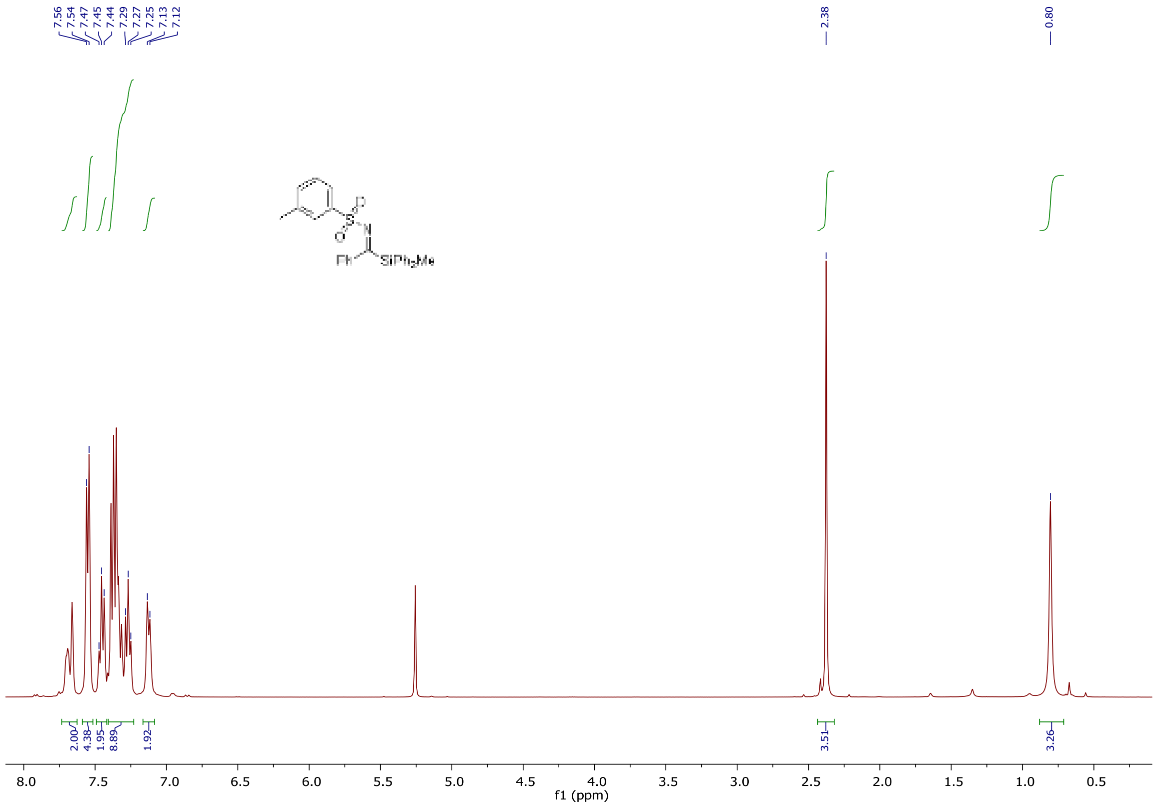

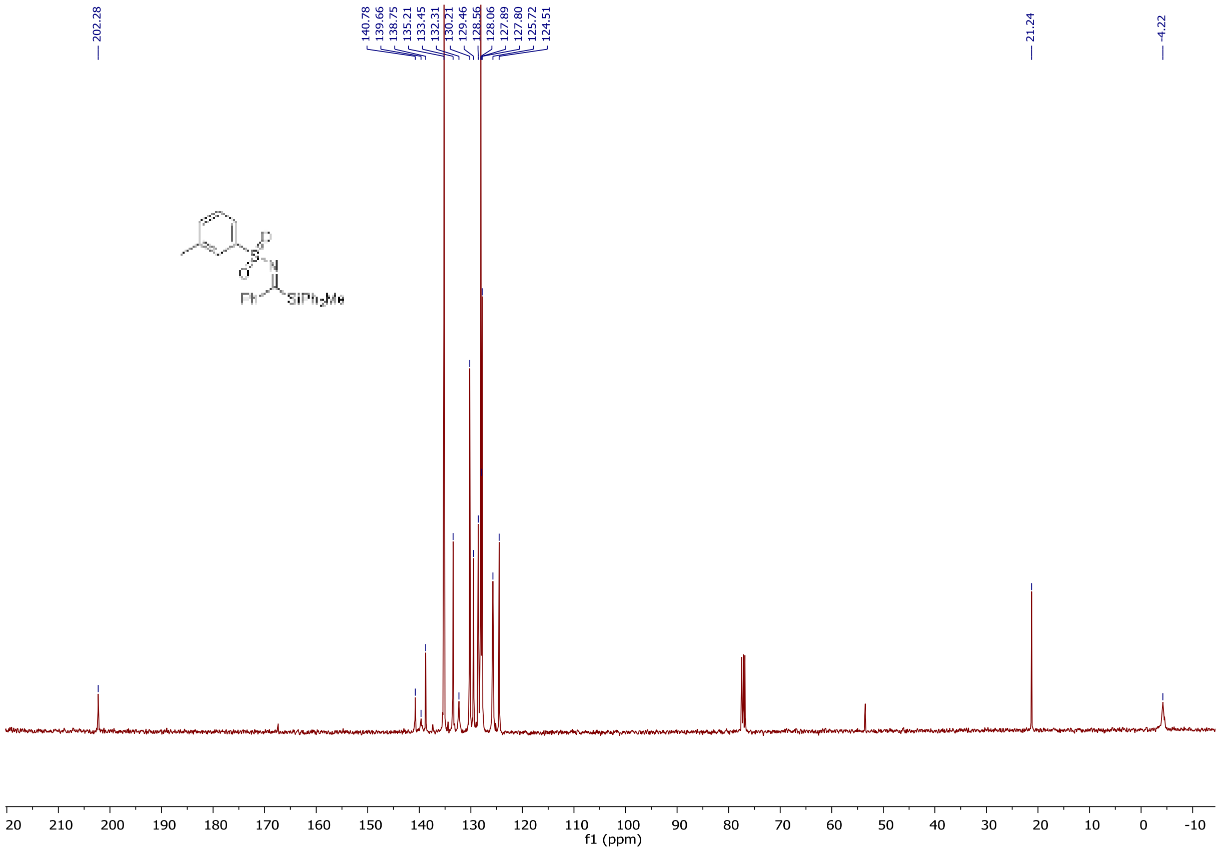

Supplementary Figure 34. 1H, 13C-NMR spectra of product **4c**.

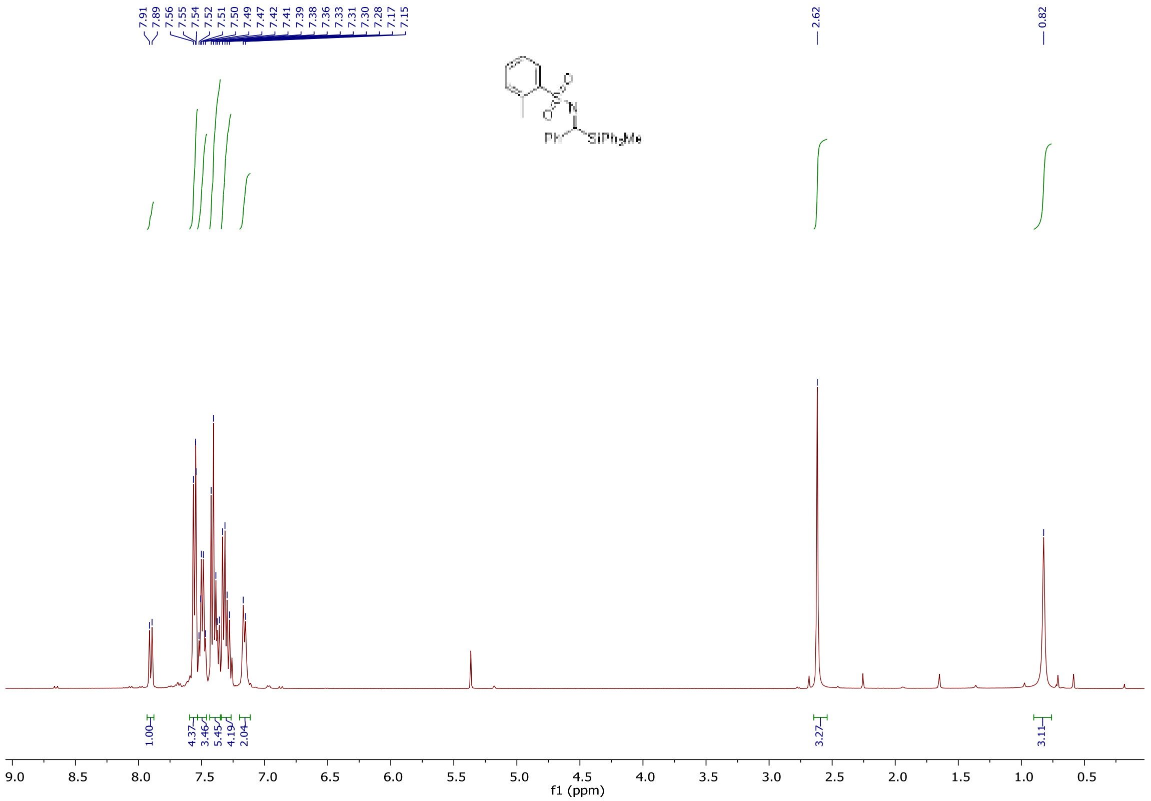

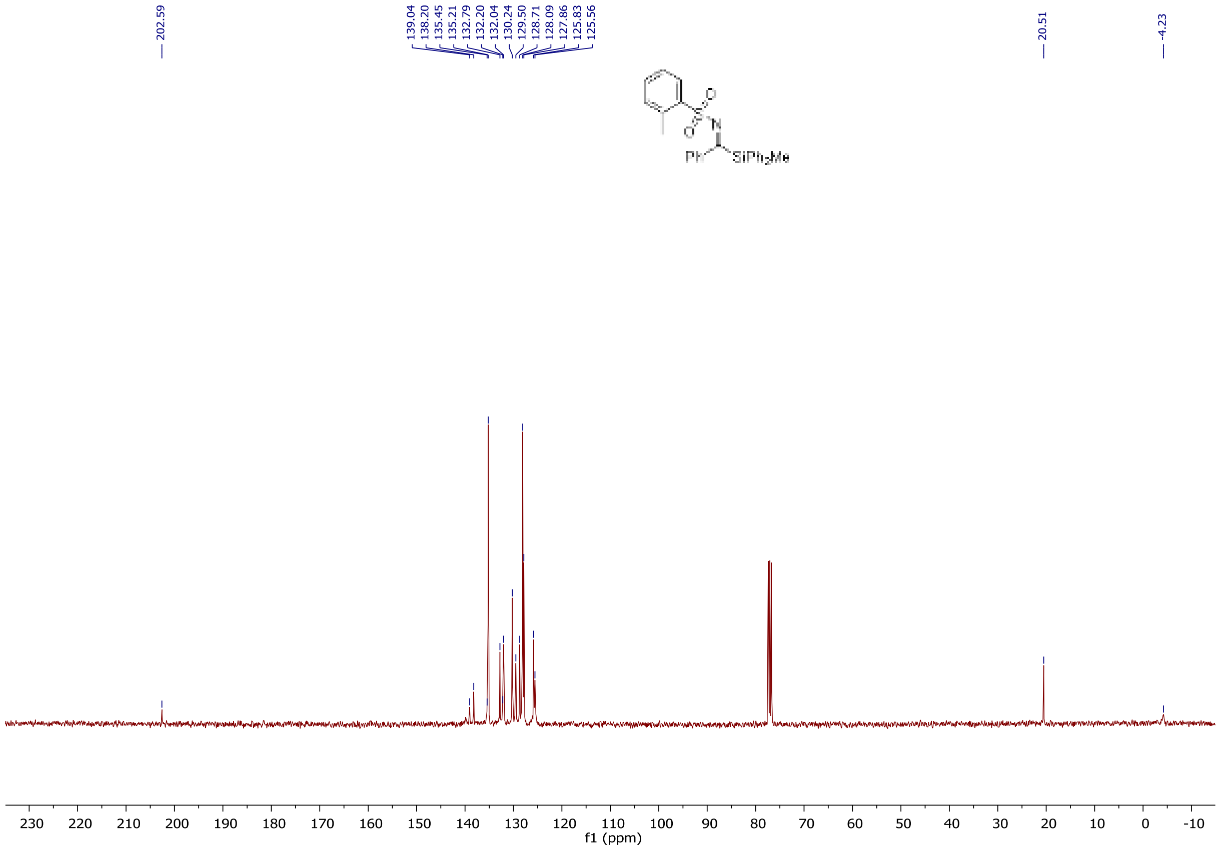

Supplementary Figure 35. 1H, 13C-NMR spectra of product **4d**.

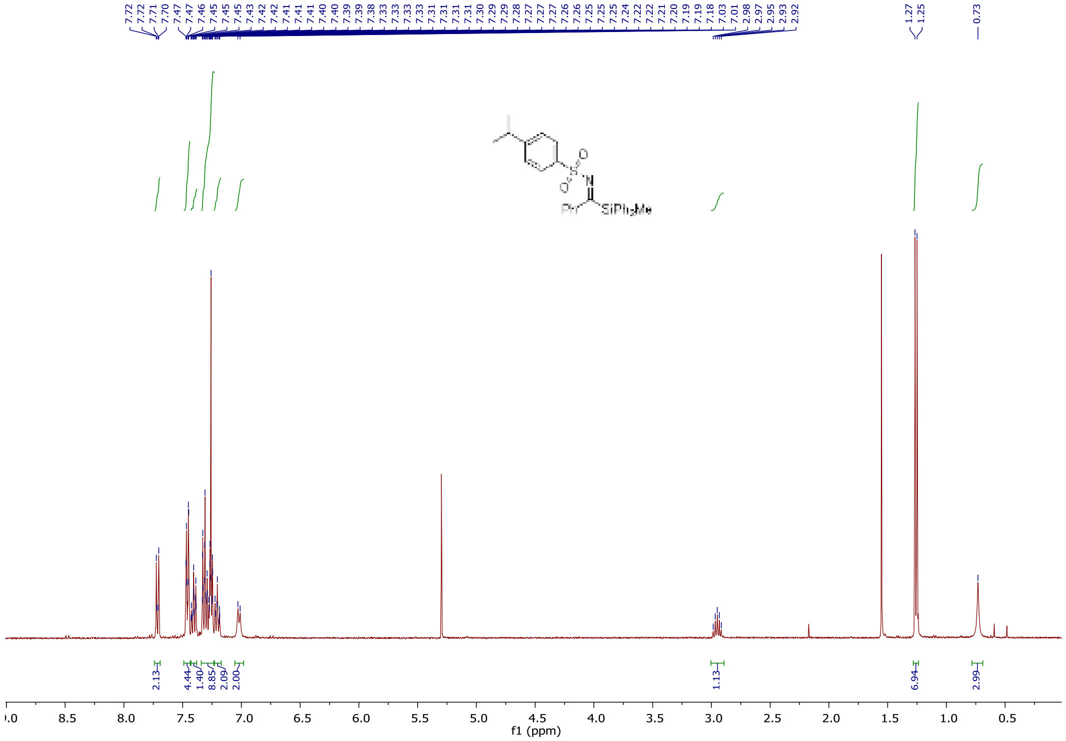

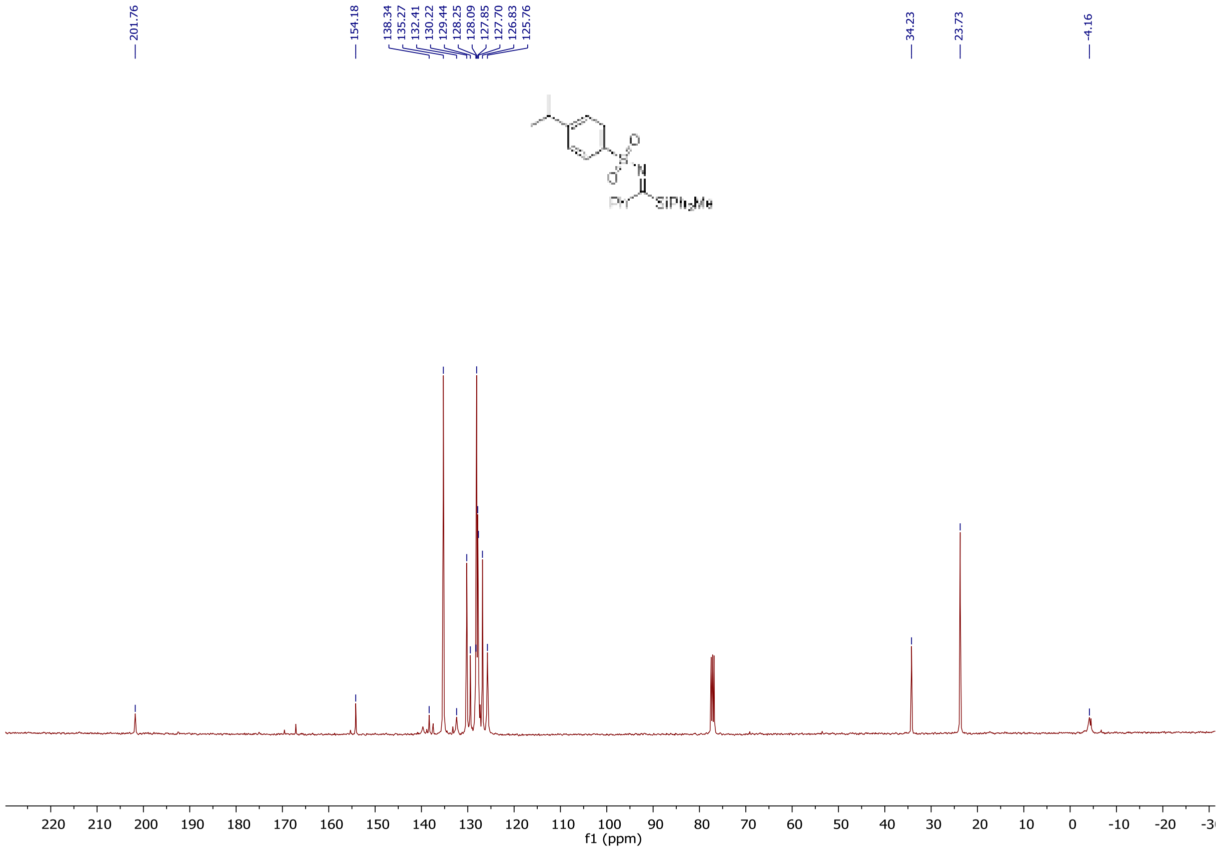

Supplementary Figure 36. 1H, 13C, 19F-NMR spectra of product **4e.**

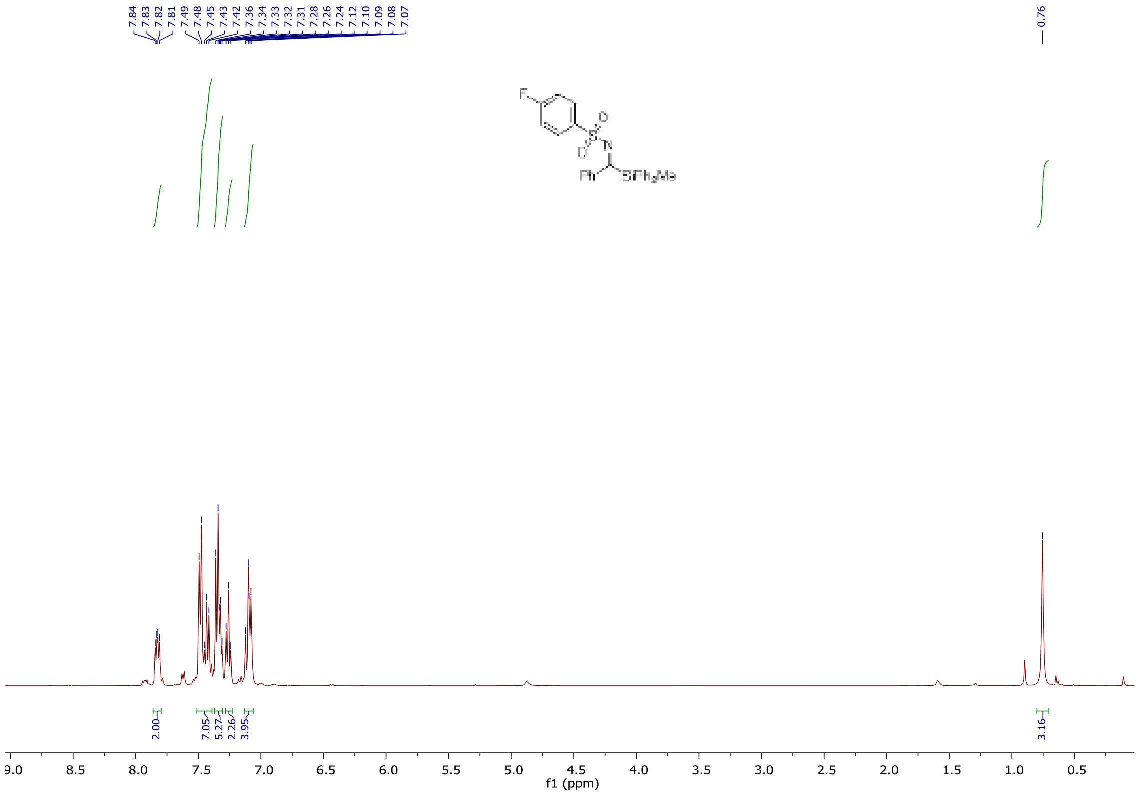

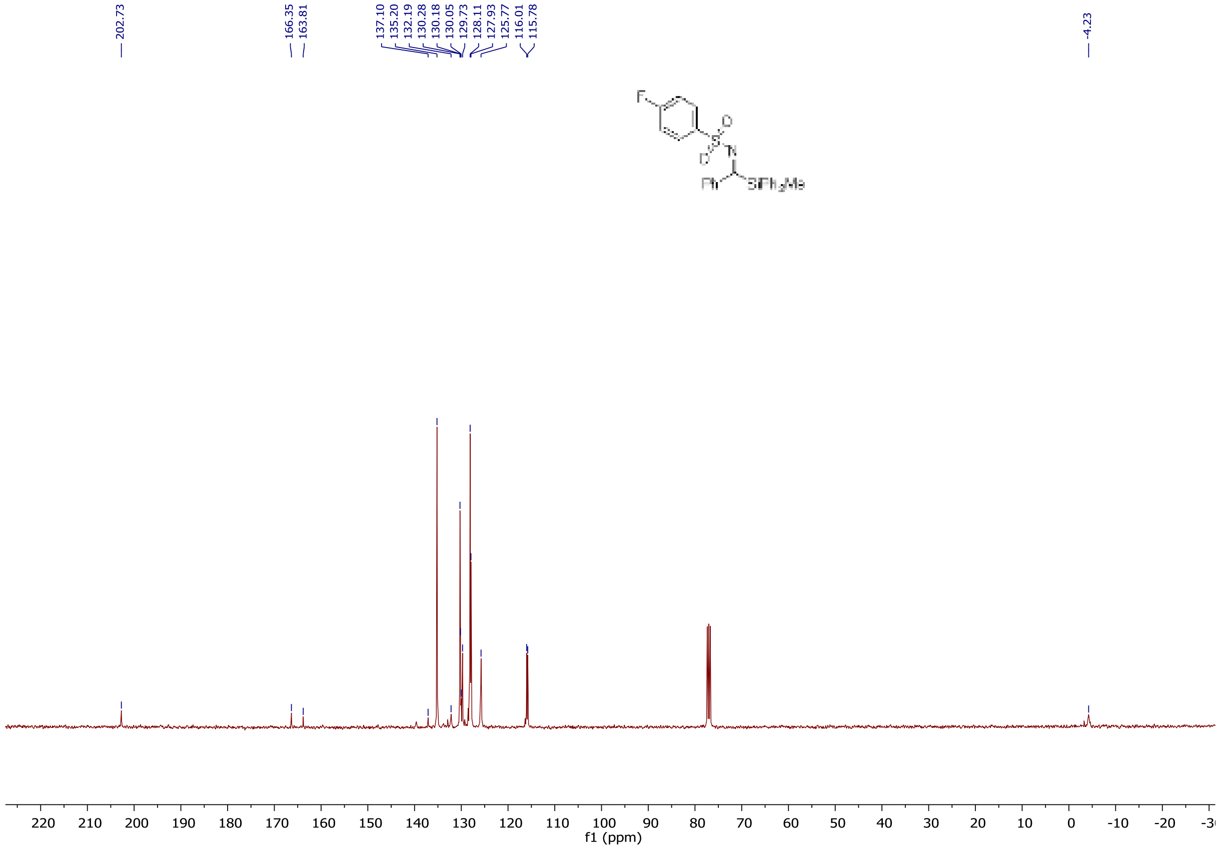

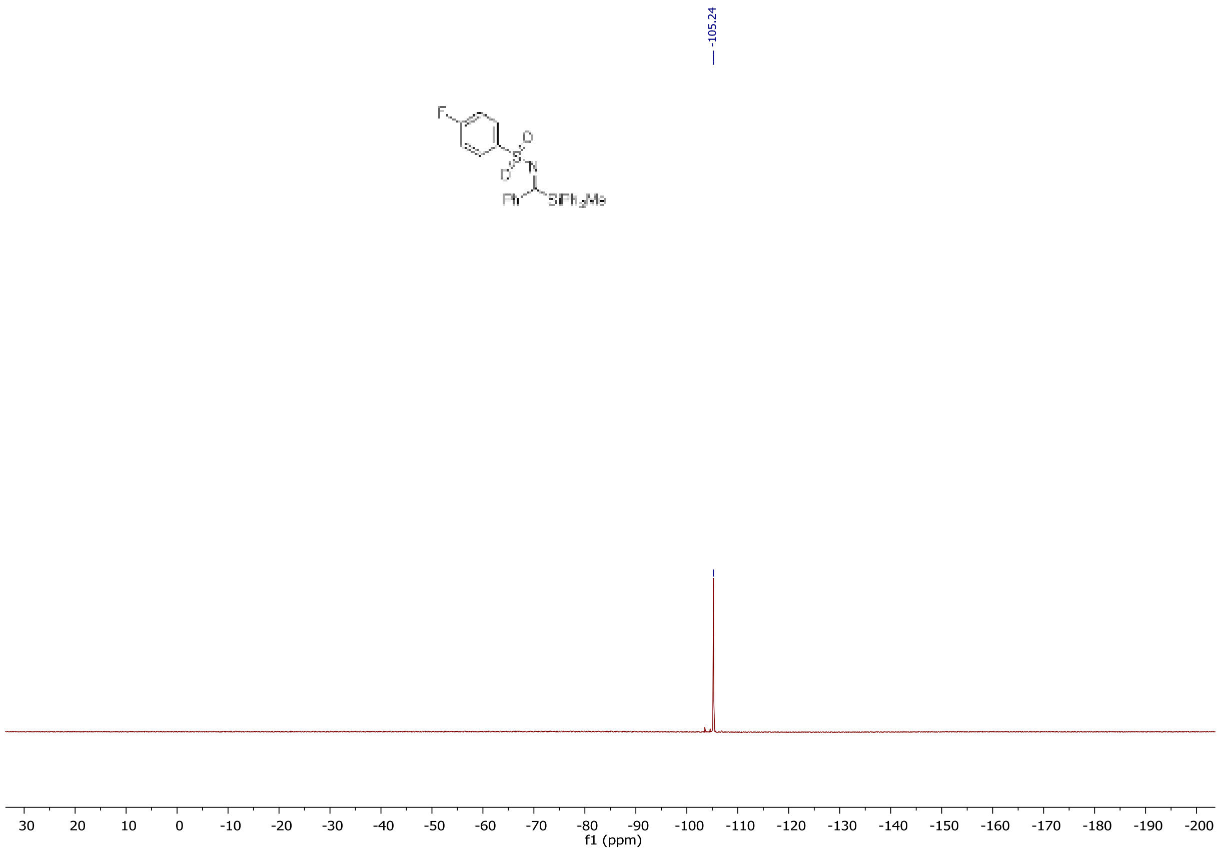
-105.24
F
O
S
O
N
Ph
SiPh2Me
30
20
10
0
-10
-20
-30
-40
-50
-60
-70
-80
-90
-100
-110
-120
-130
-140
-150
-160
-170
-180
-190
-200
f1 (ppm)

Supplementary Figure 37. 1H, 13C-NMR spectra of product **4f**.

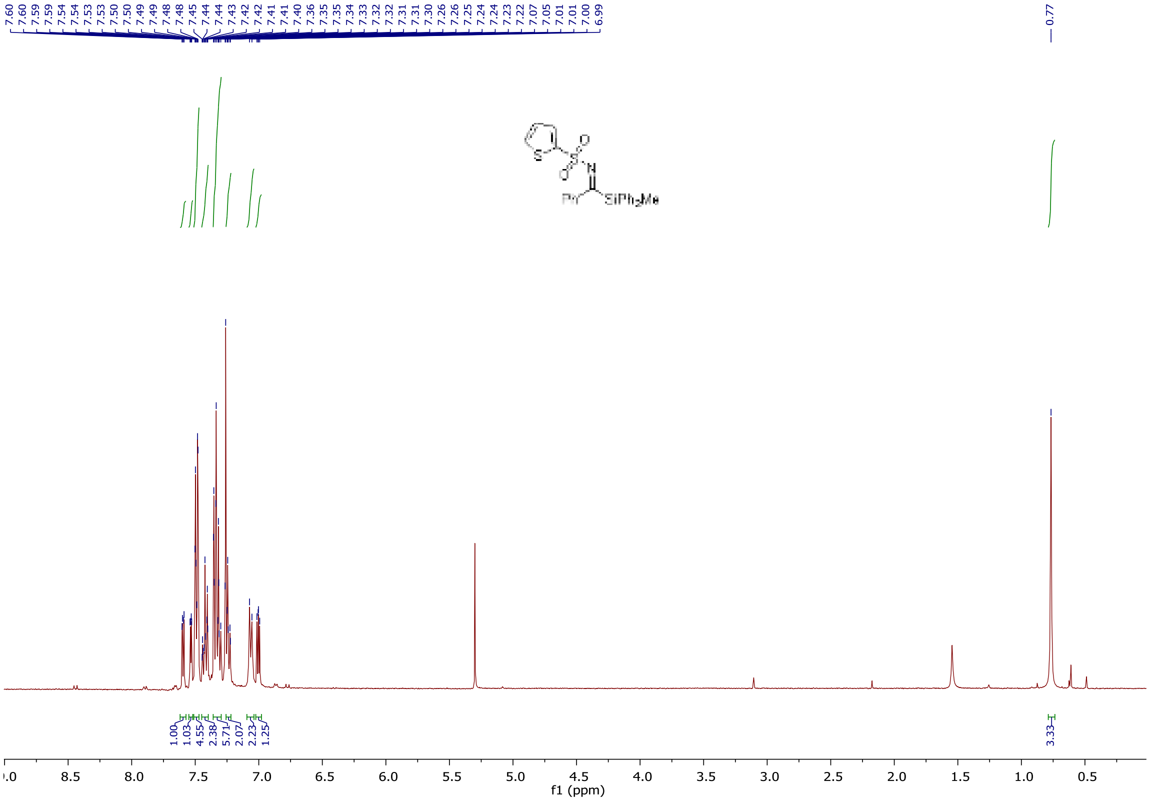

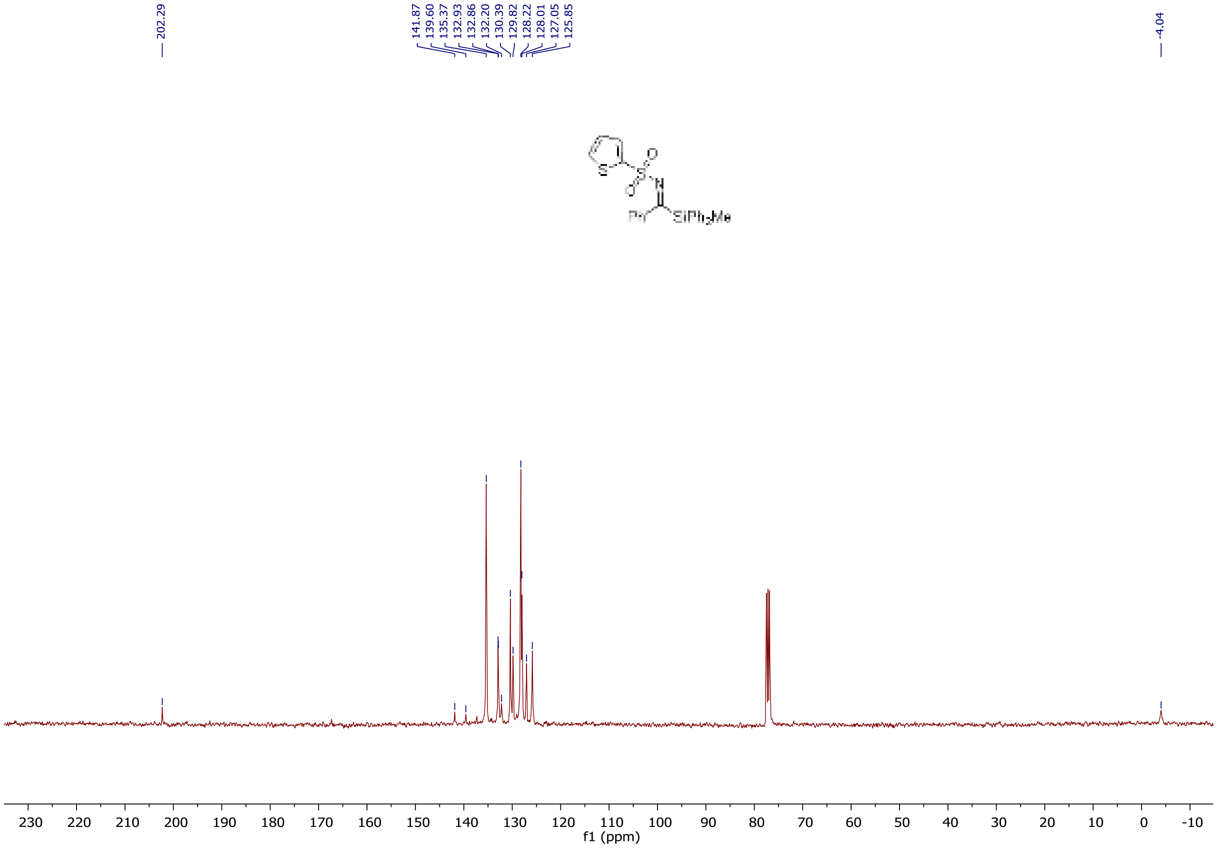

Supplementary Figure 38. 1H, 13C-NMR spectra of product **4g**.

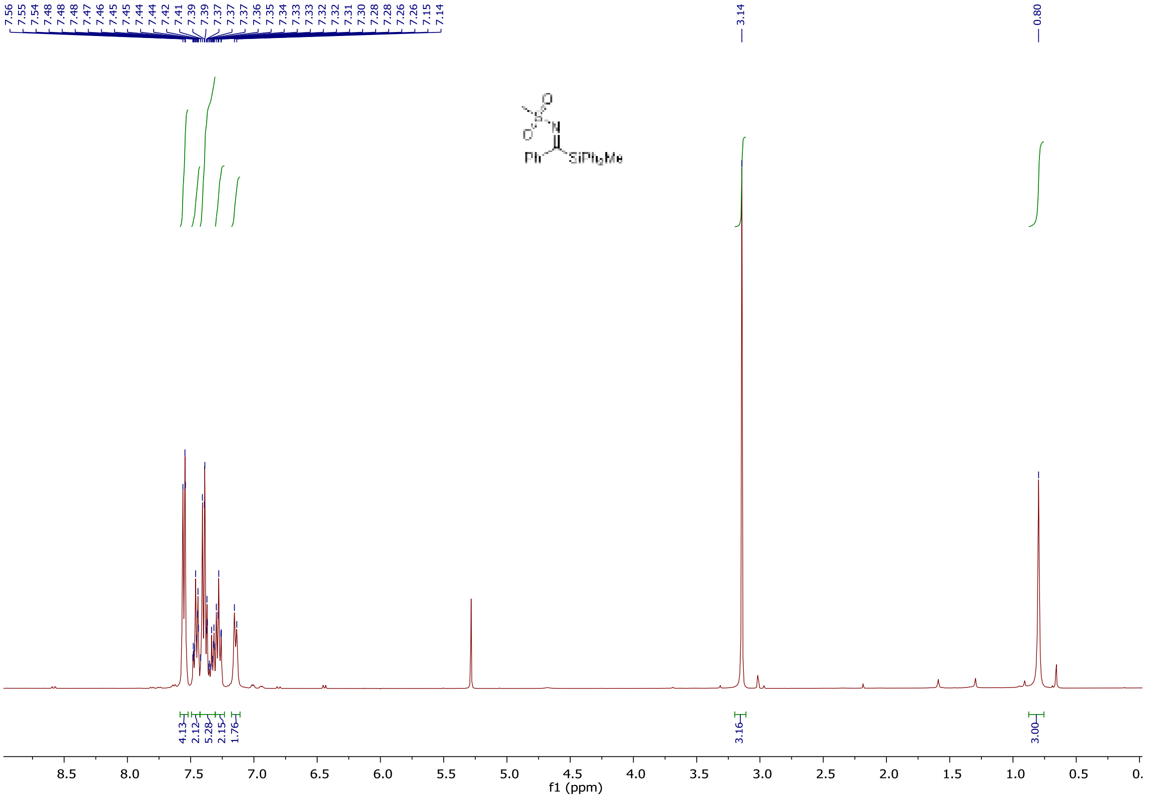

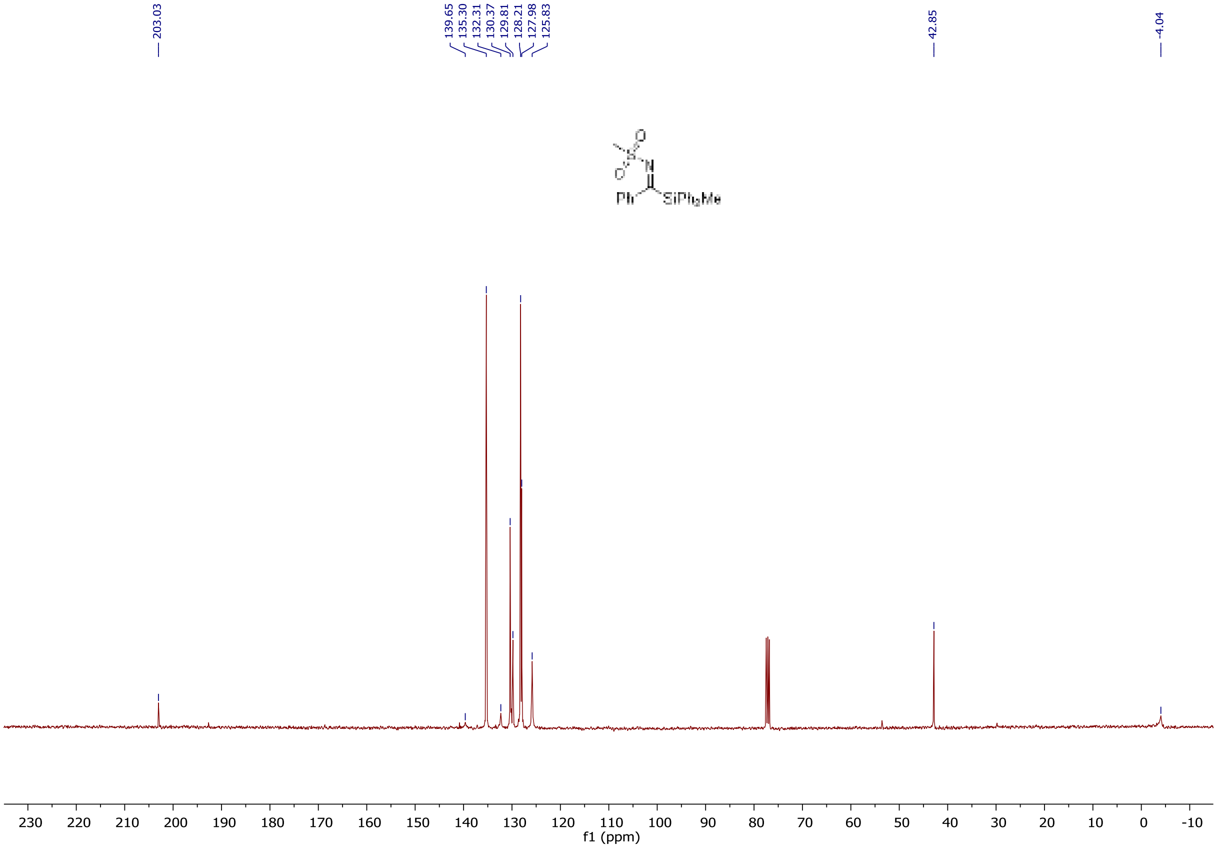

Supplementary Figure 39. 1H, 13C-NMR spectra of product **4h**.

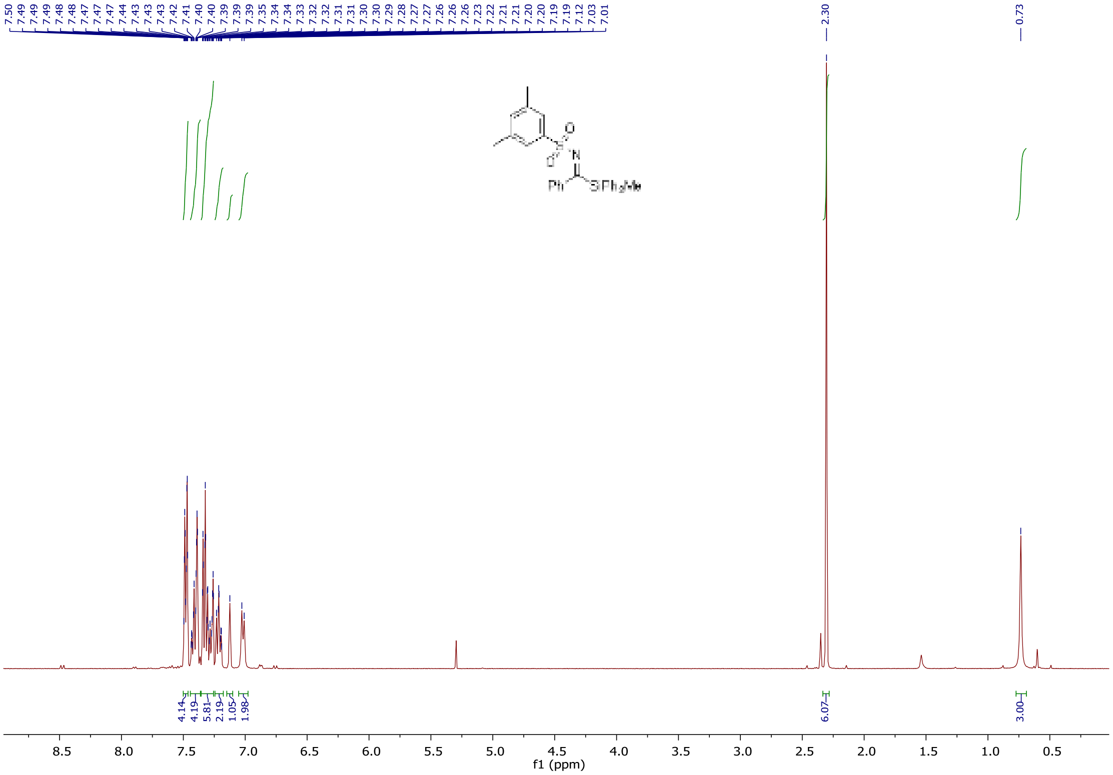

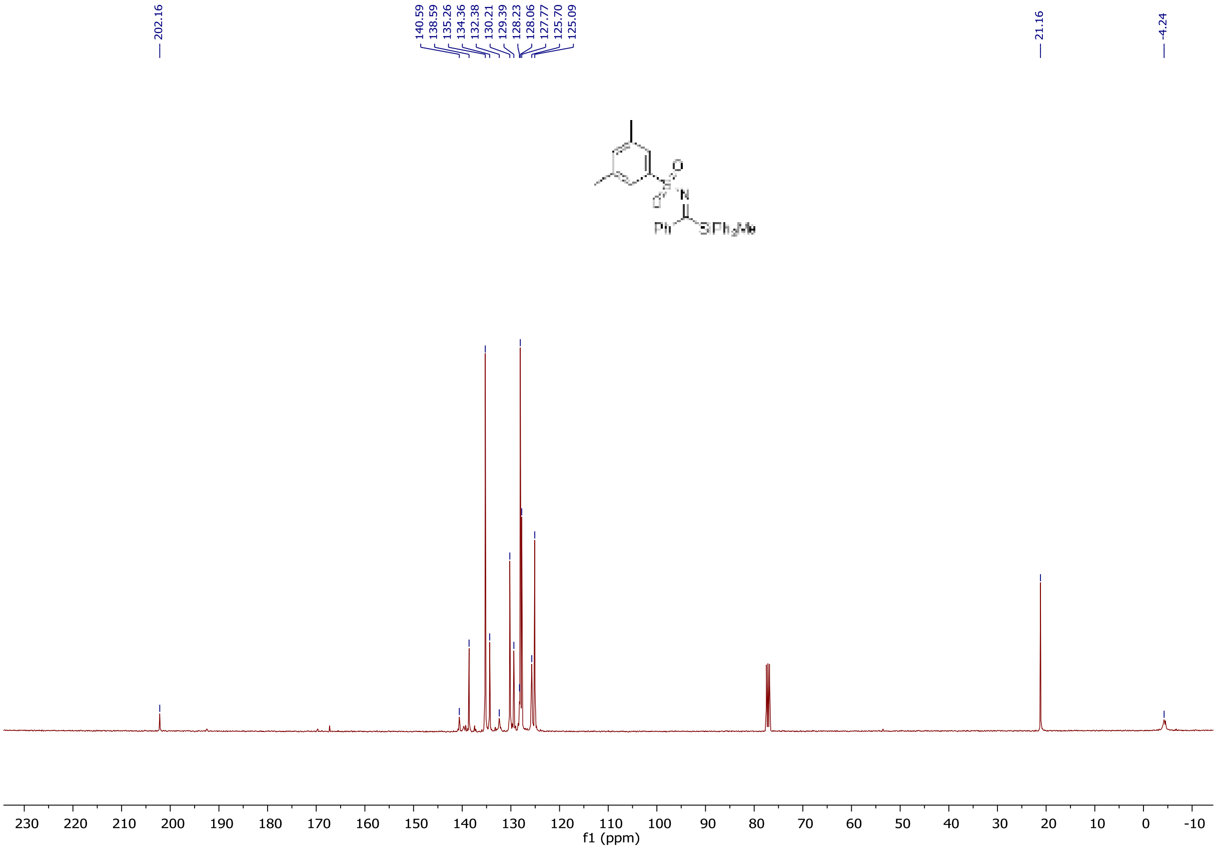

Supplementary Figure 40. 1H, 13C-NMR spectra of product **4i**.

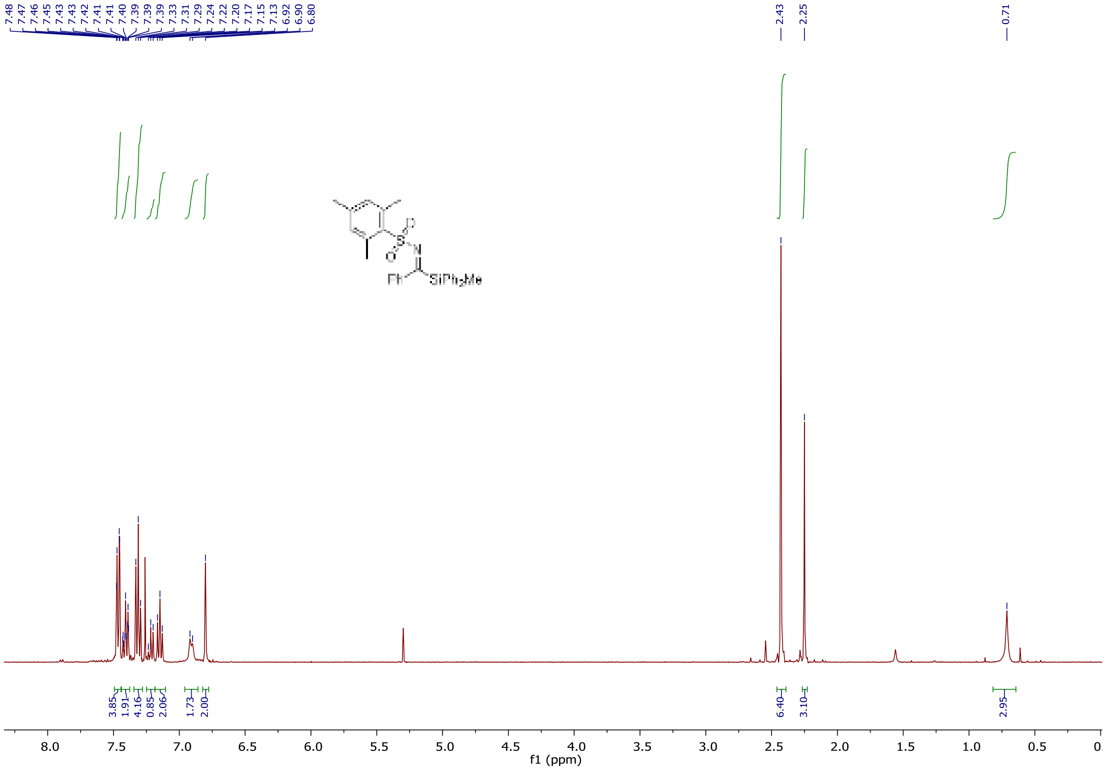

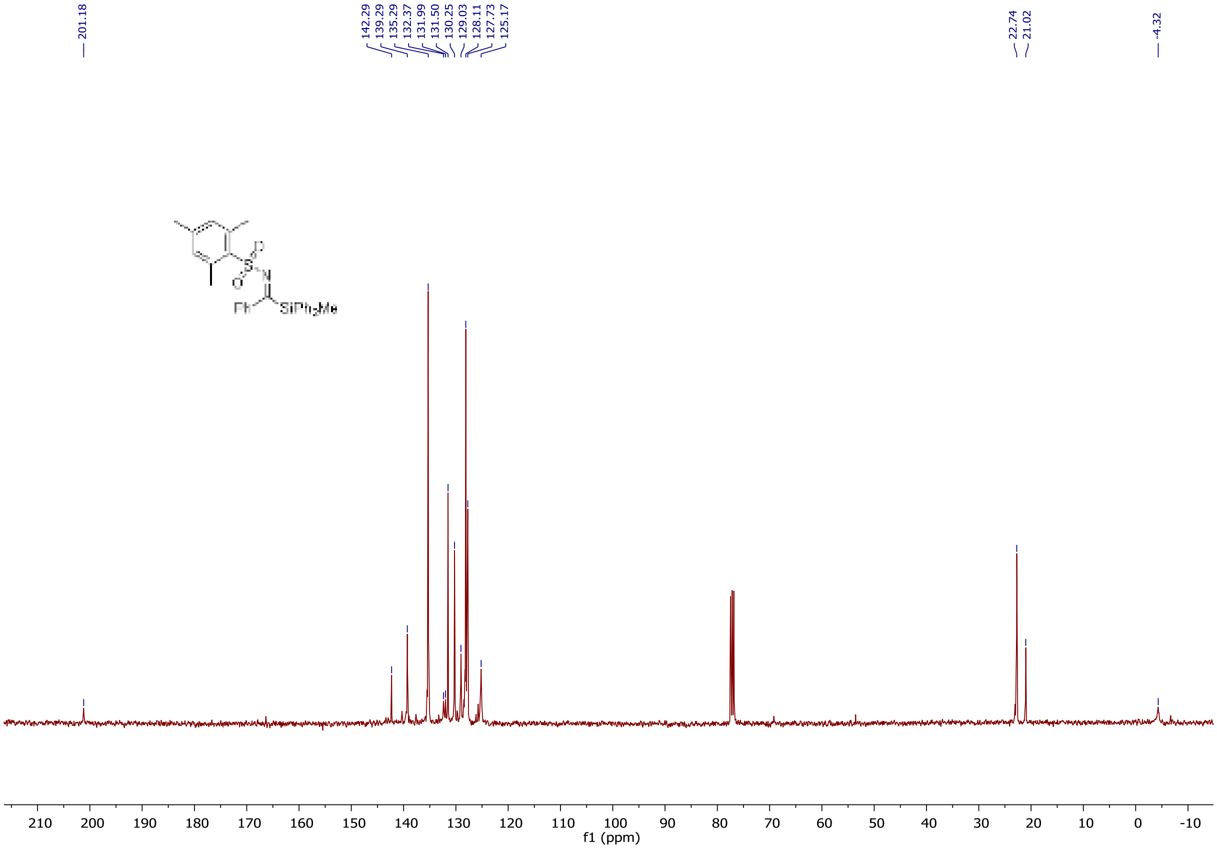

Supplementary Figure 41. 1H, 13C-NMR spectra of product **4j**.

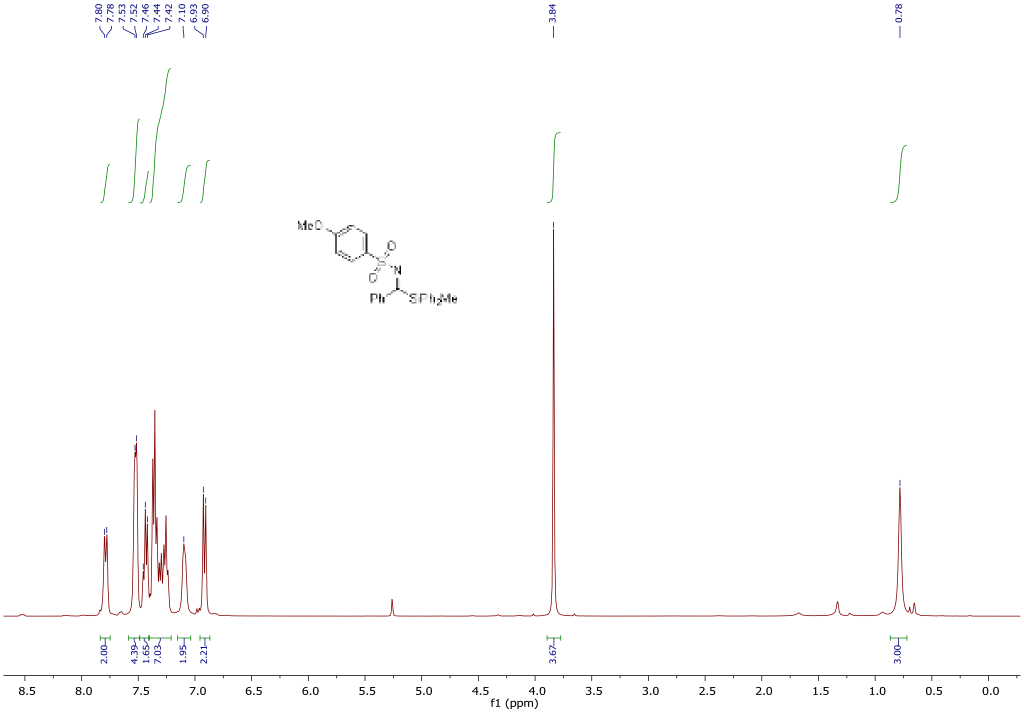

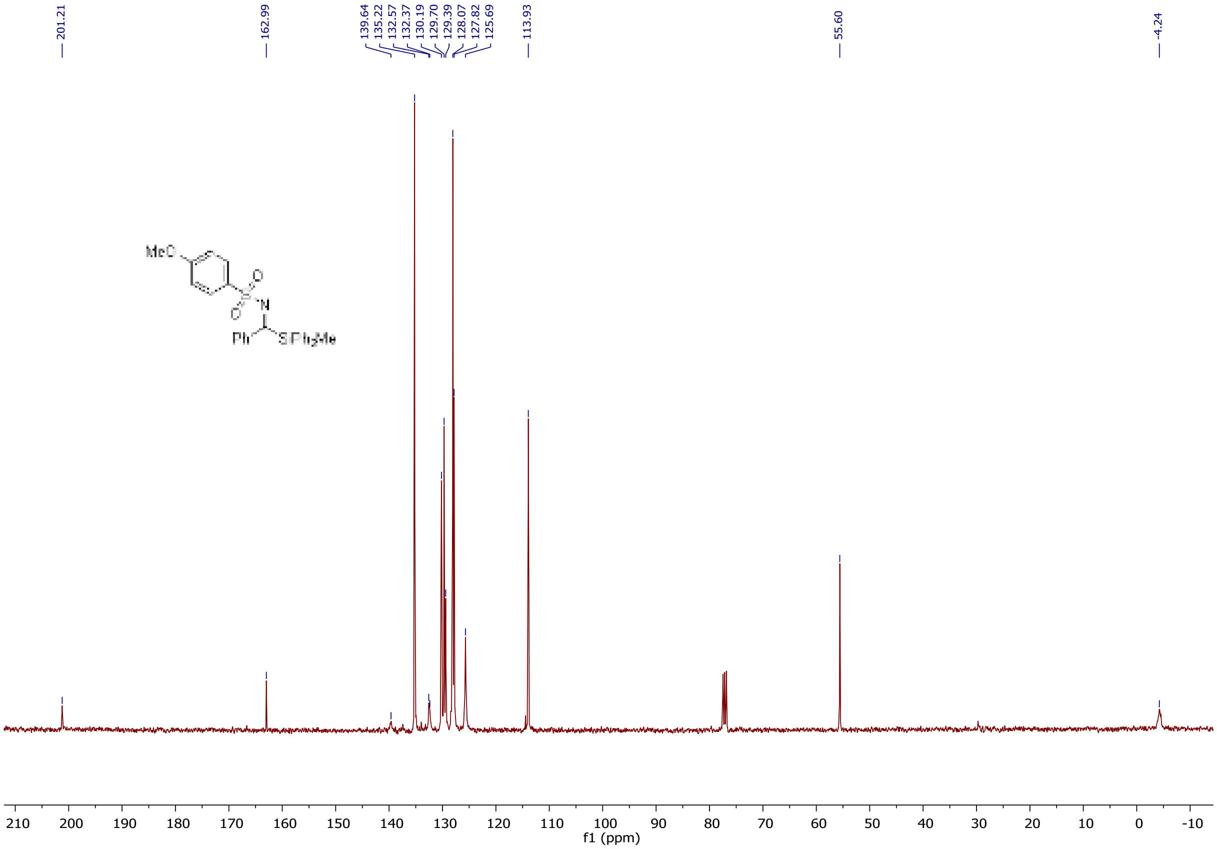

Supplementary Figure 42. 1H, 13C-NMR spectra of product **4k**.

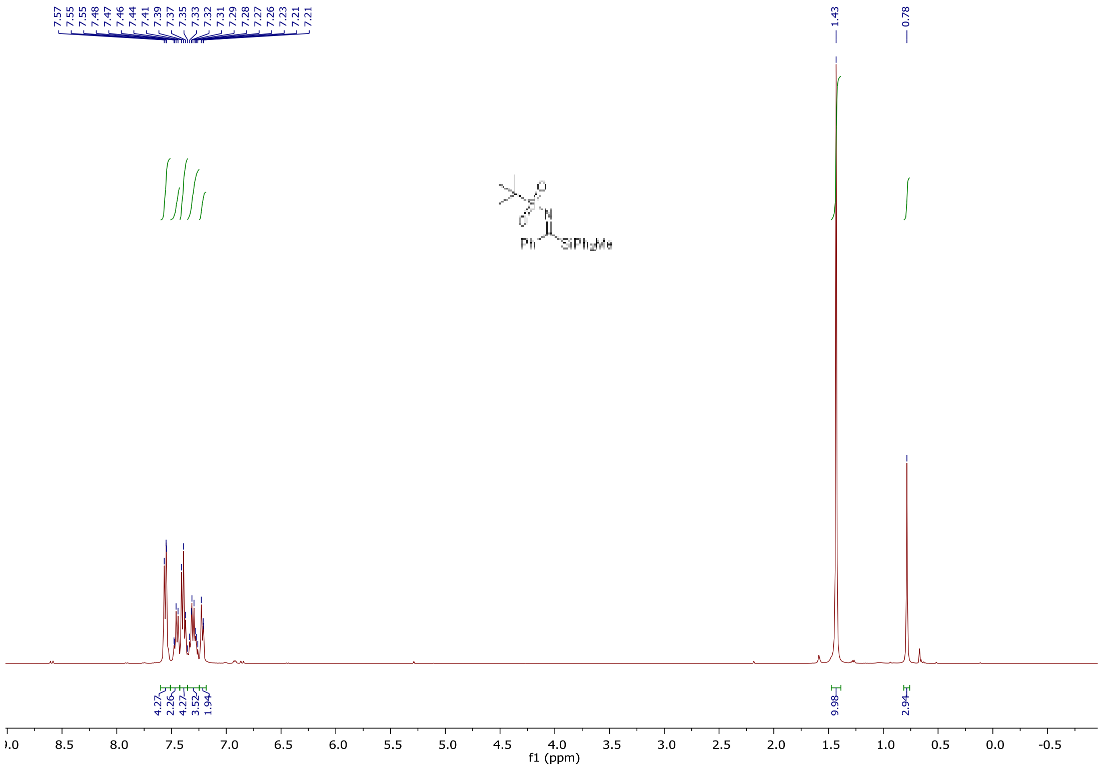

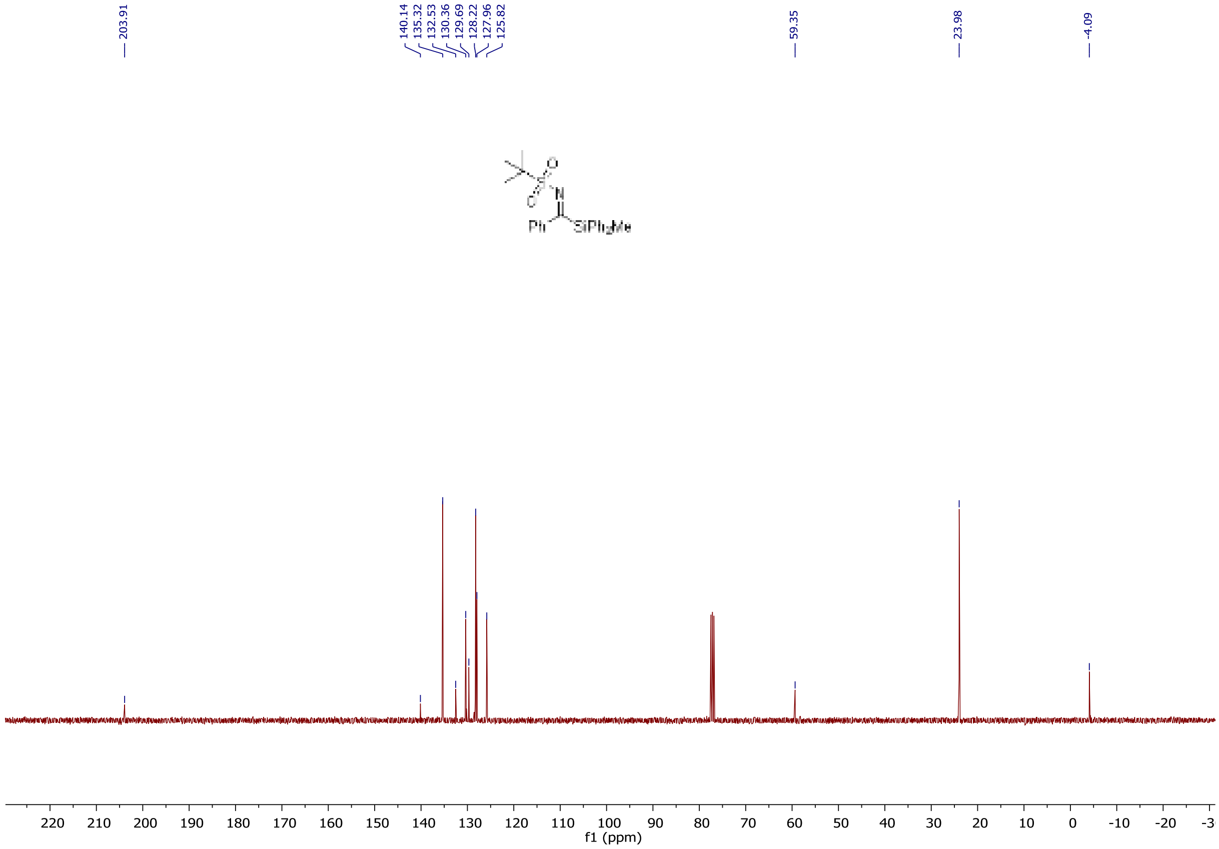

Supplementary Figure 43. 1H, 13C-NMR spectra of product **2a**.

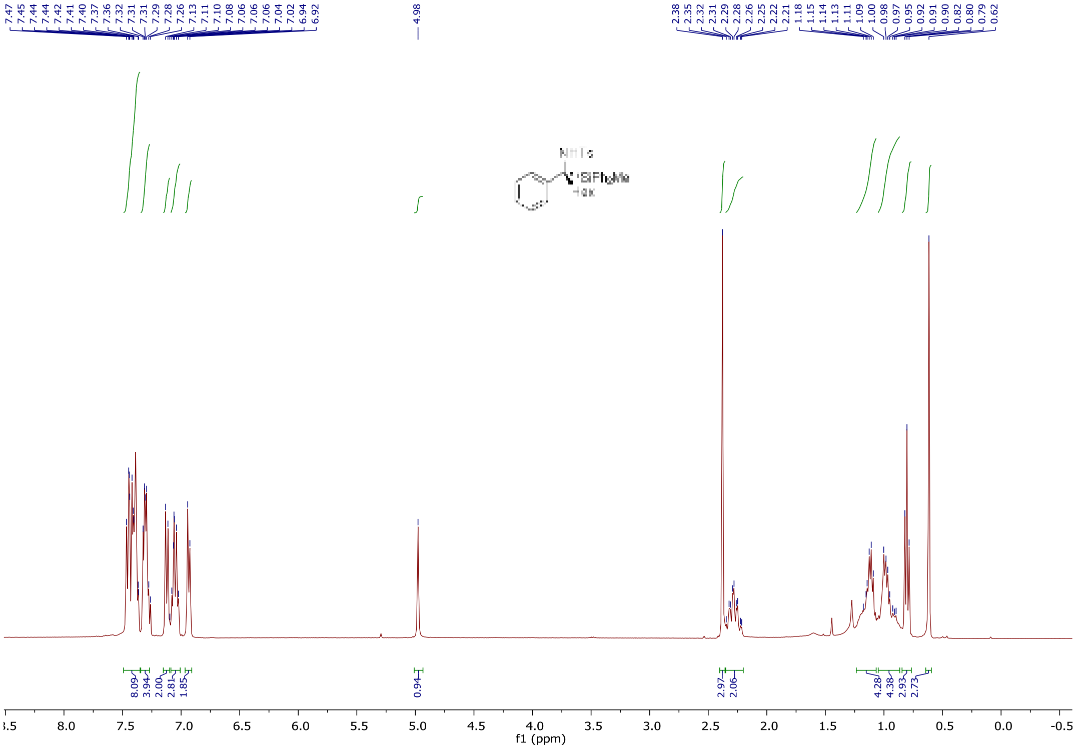

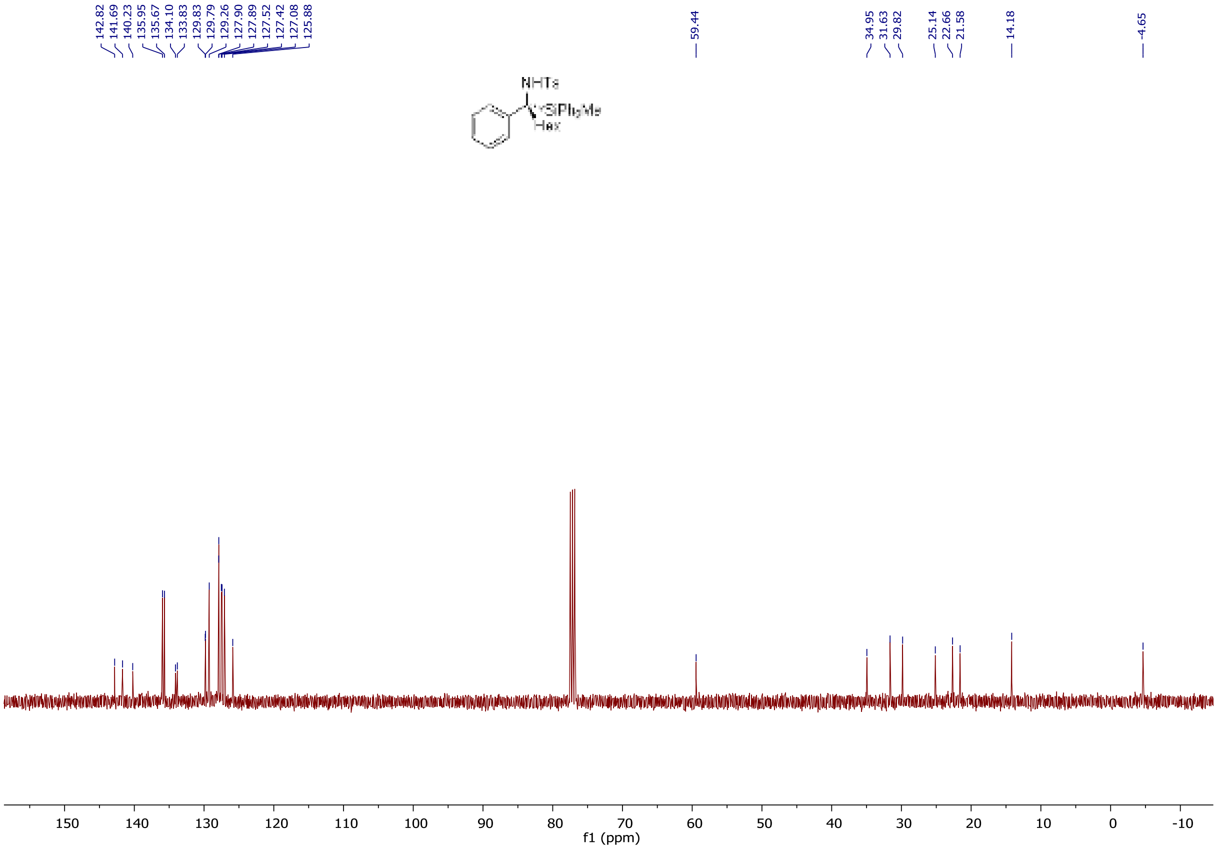

Supplementary Figure 44. 1H, 13C-NMR spectra of product **2ab**.

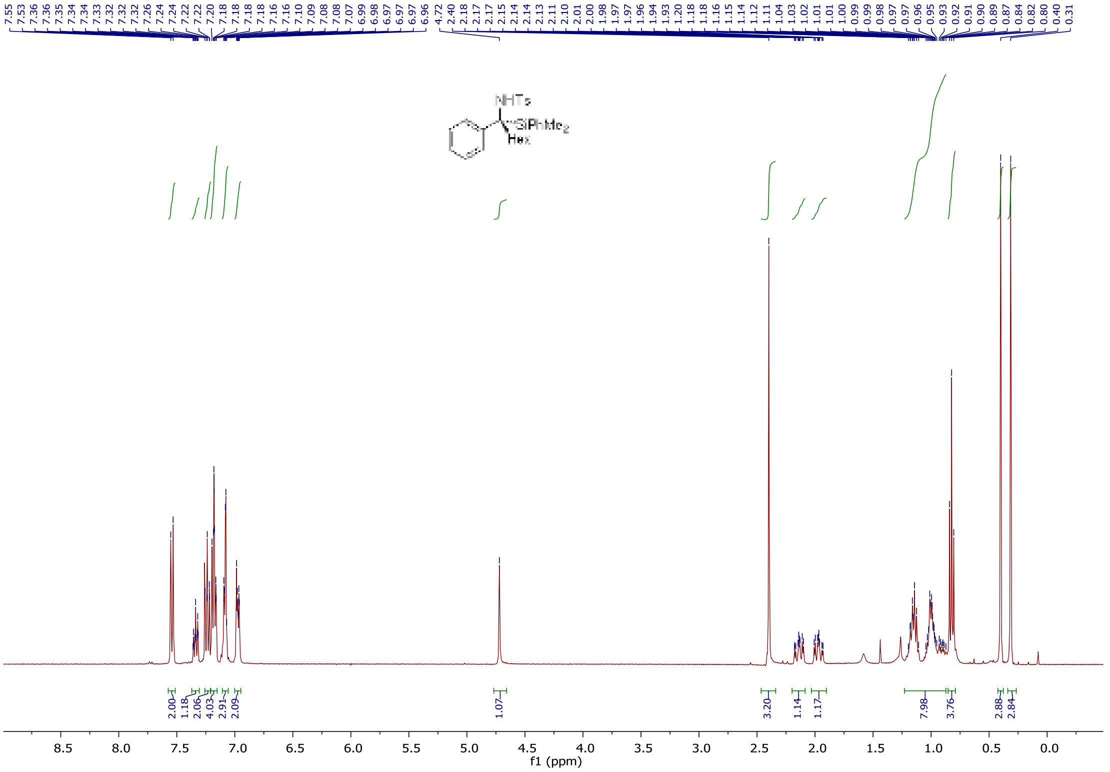

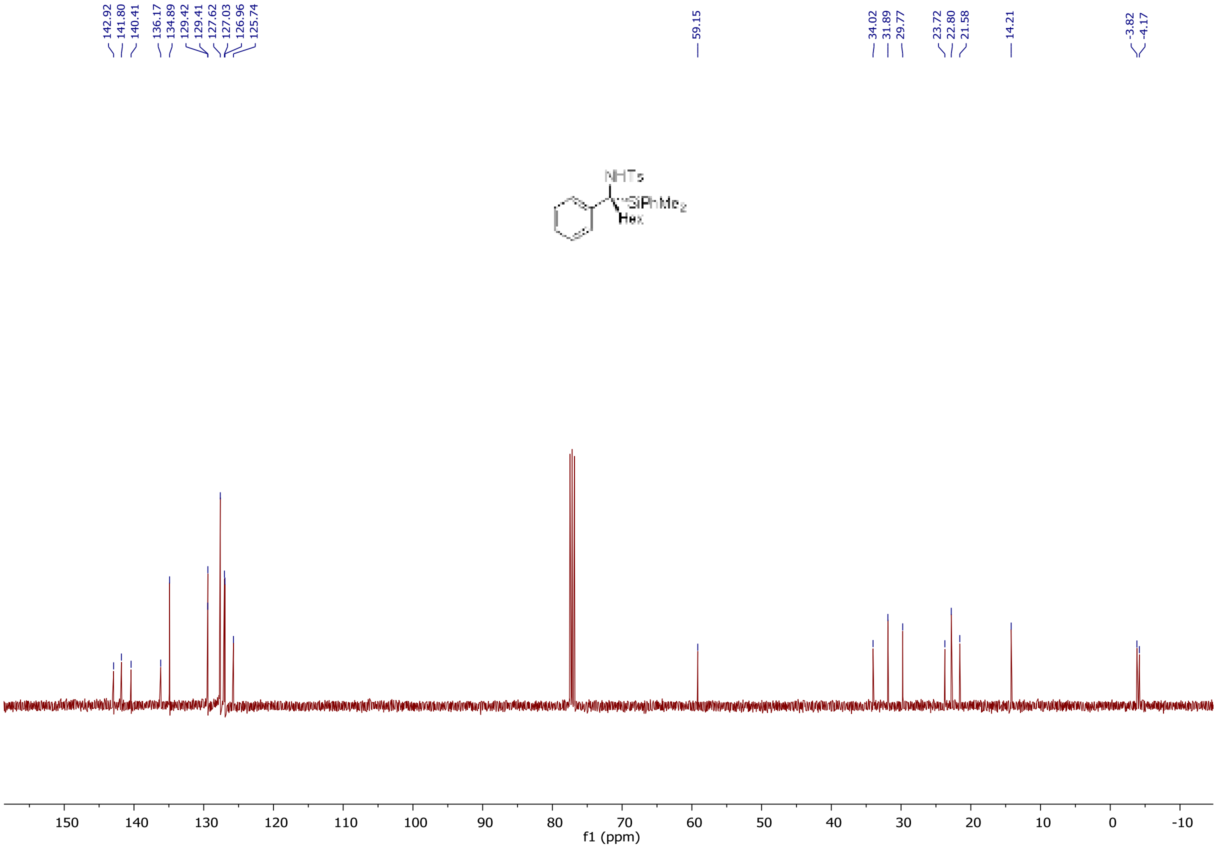

Supplementary Figure 45. 1H, 13C-NMR spectra of product **2ac.**

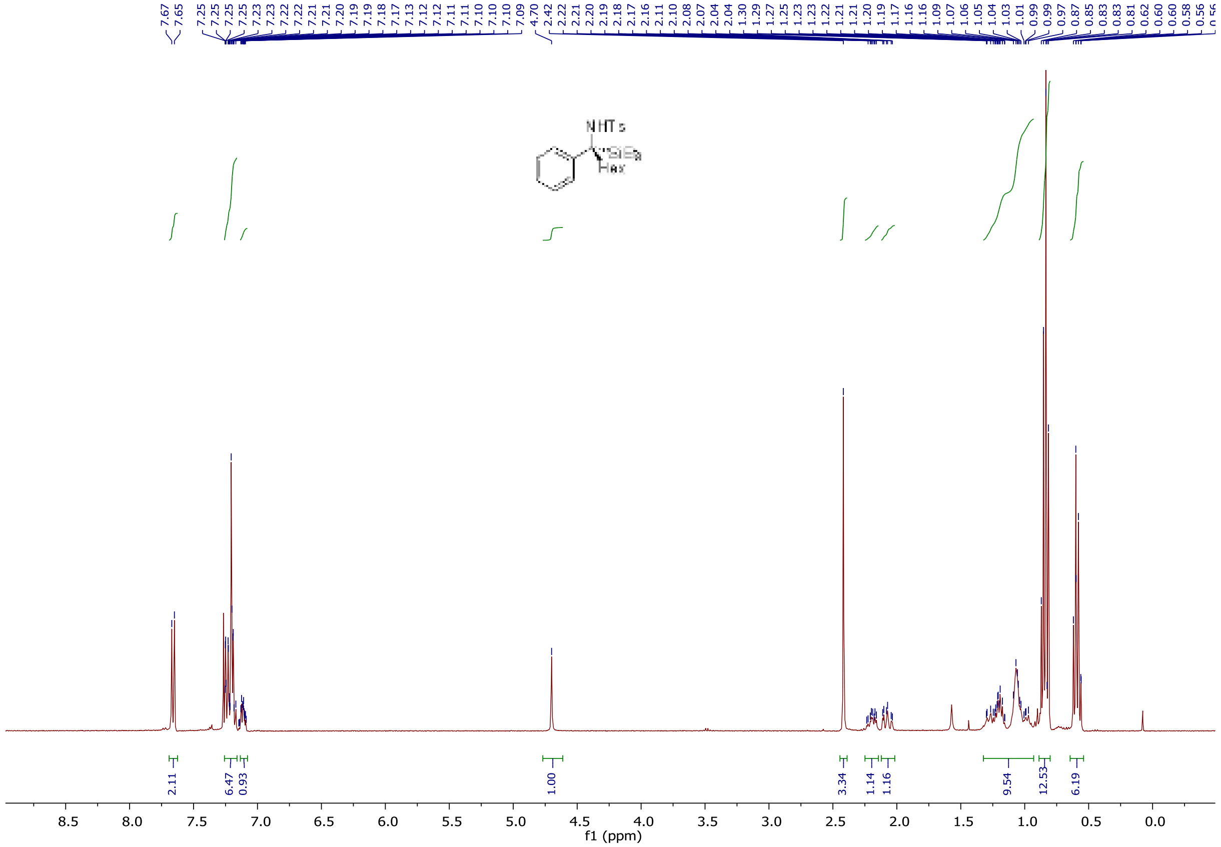

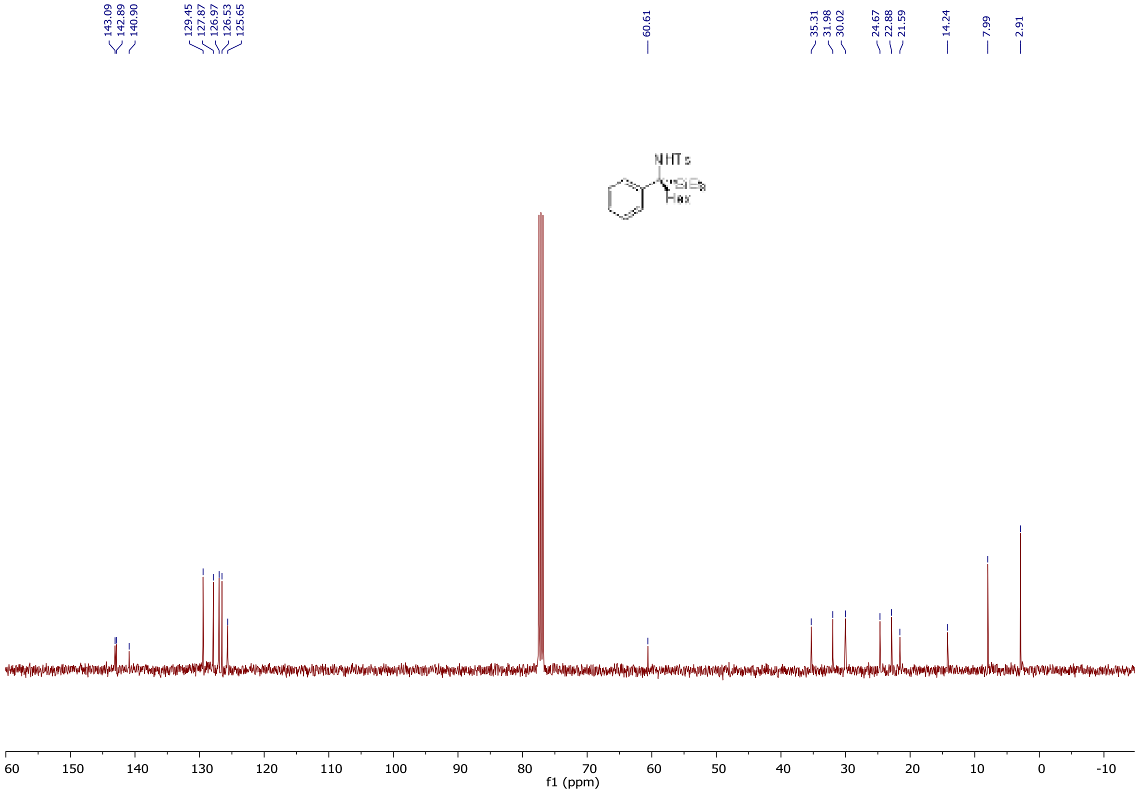

Supplementary Figure 46. 1H, 13C-NMR spectra of product **5a**.

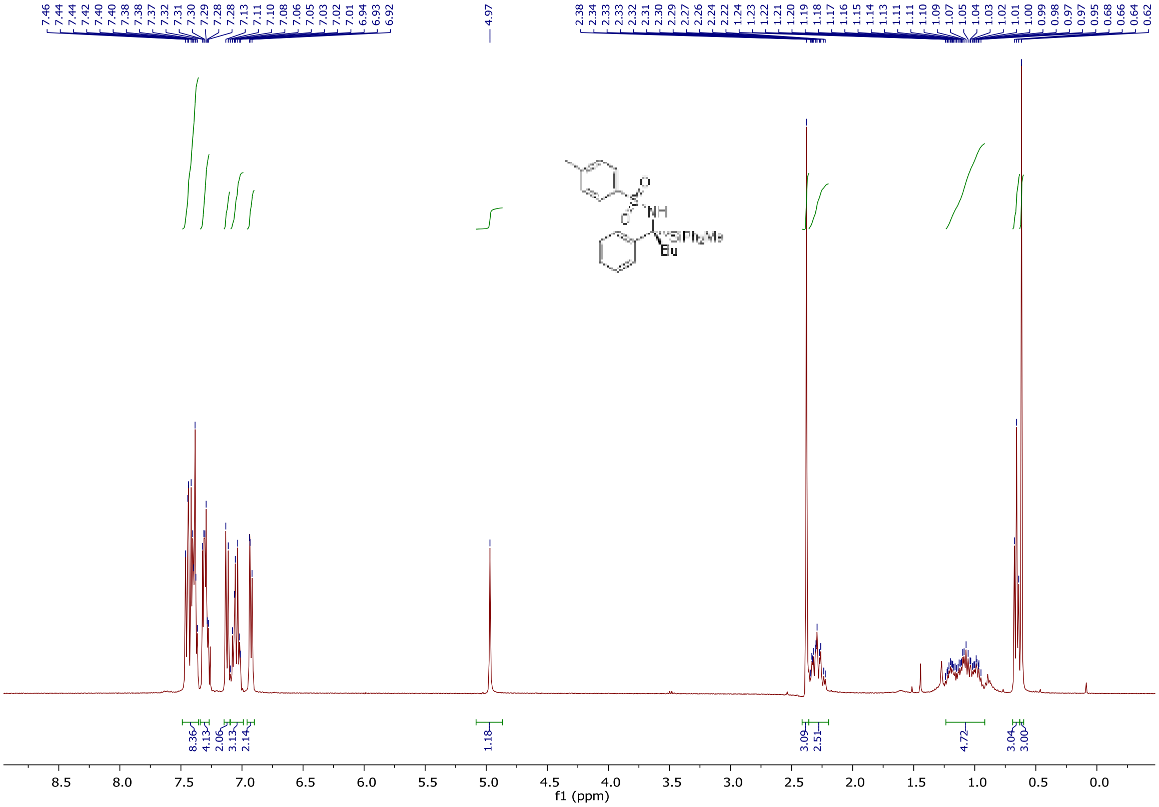

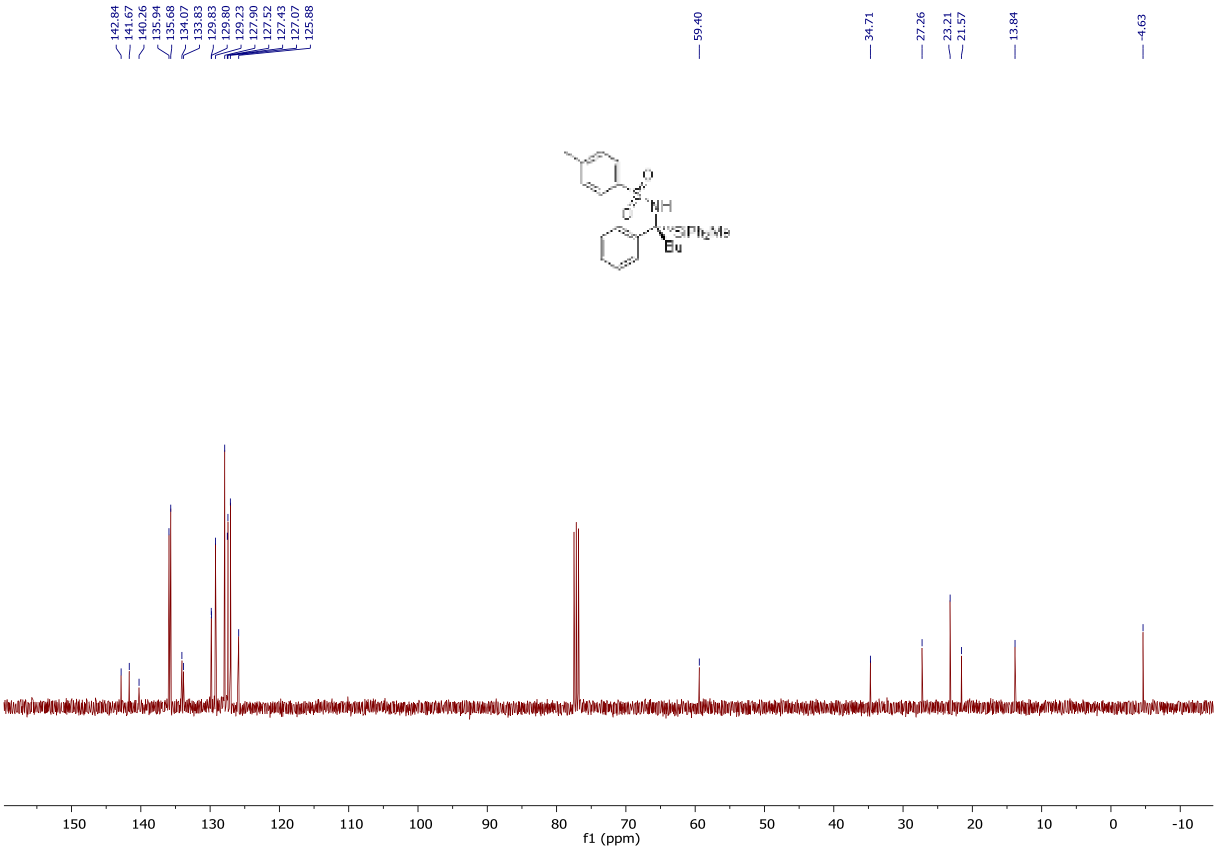

Supplementary Figure 47. 1H, 13C-NMR spectra of product **5b**.

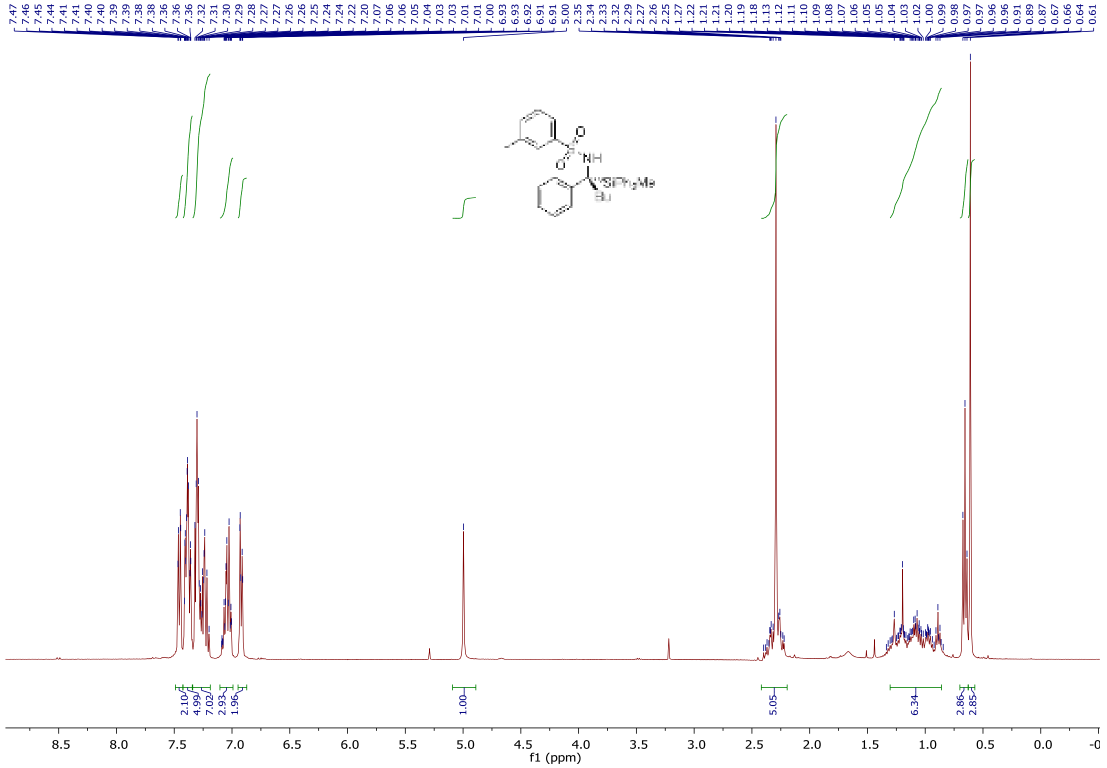

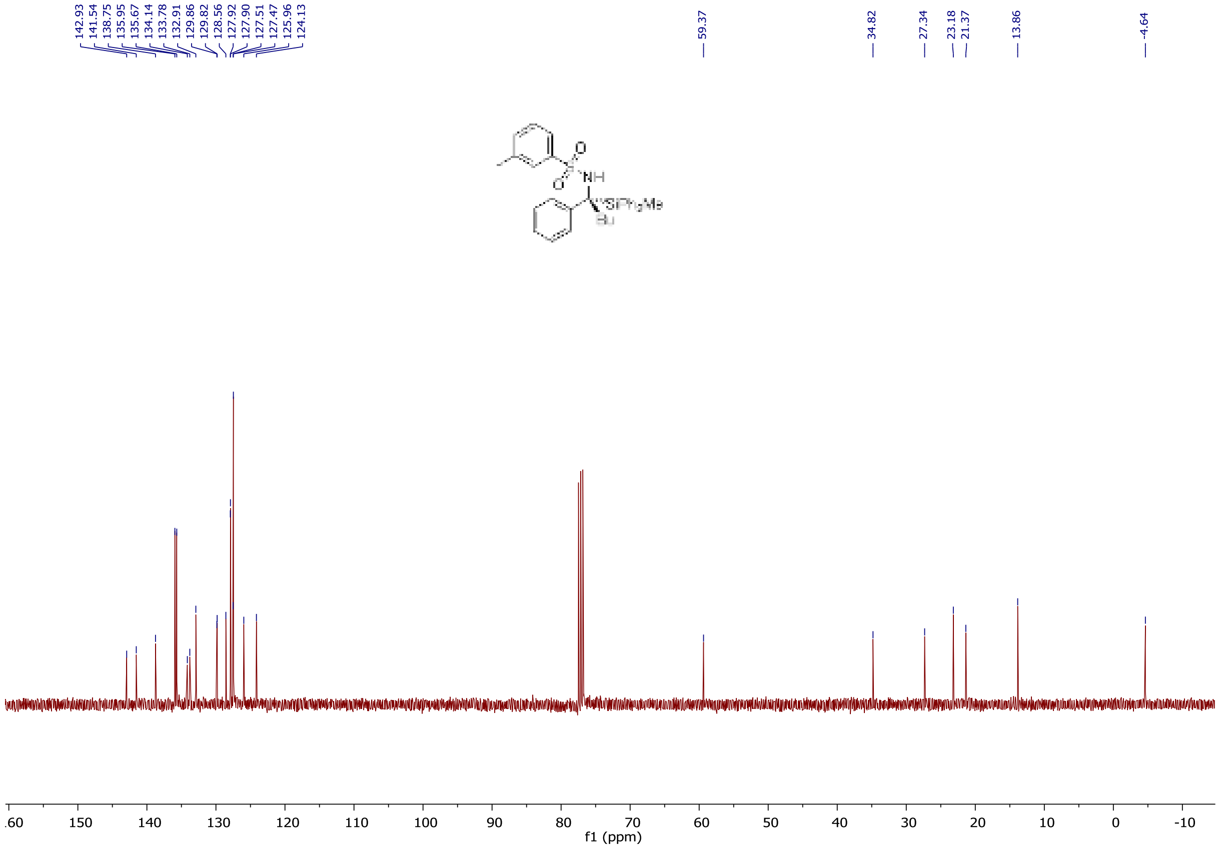

Supplementary Figure 48. 1H, 13C-NMR spectra of product **5c**.

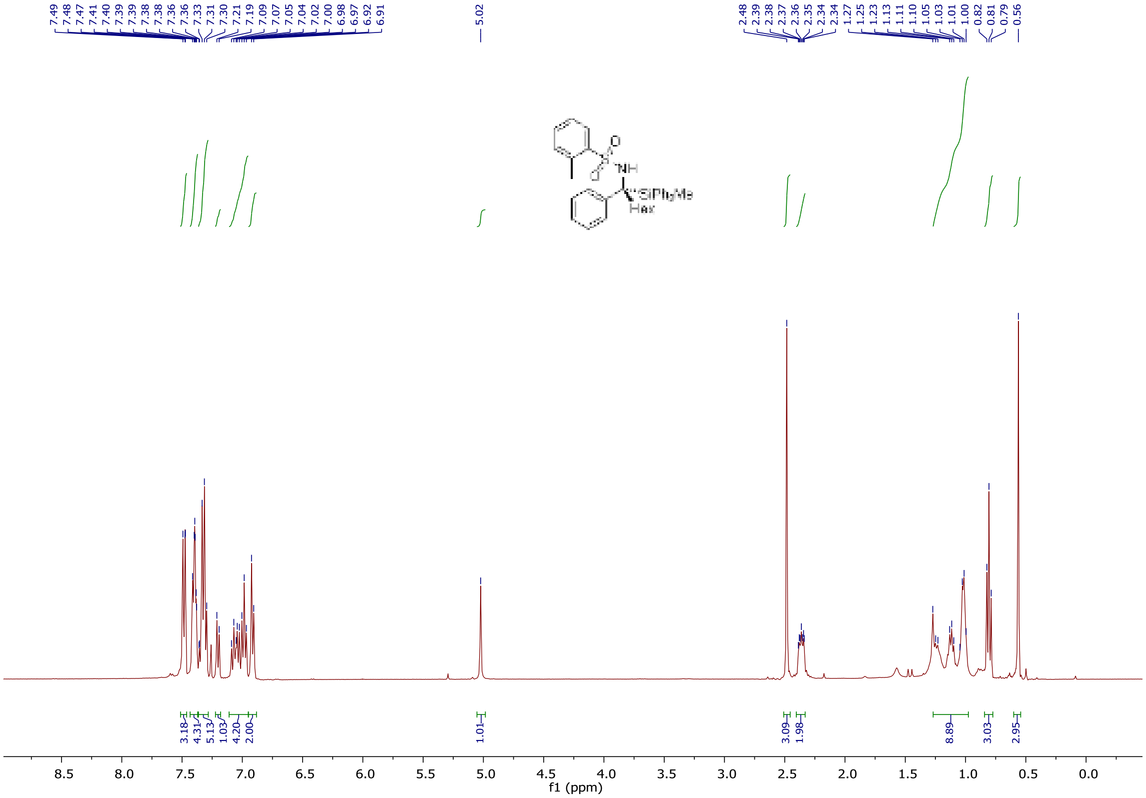

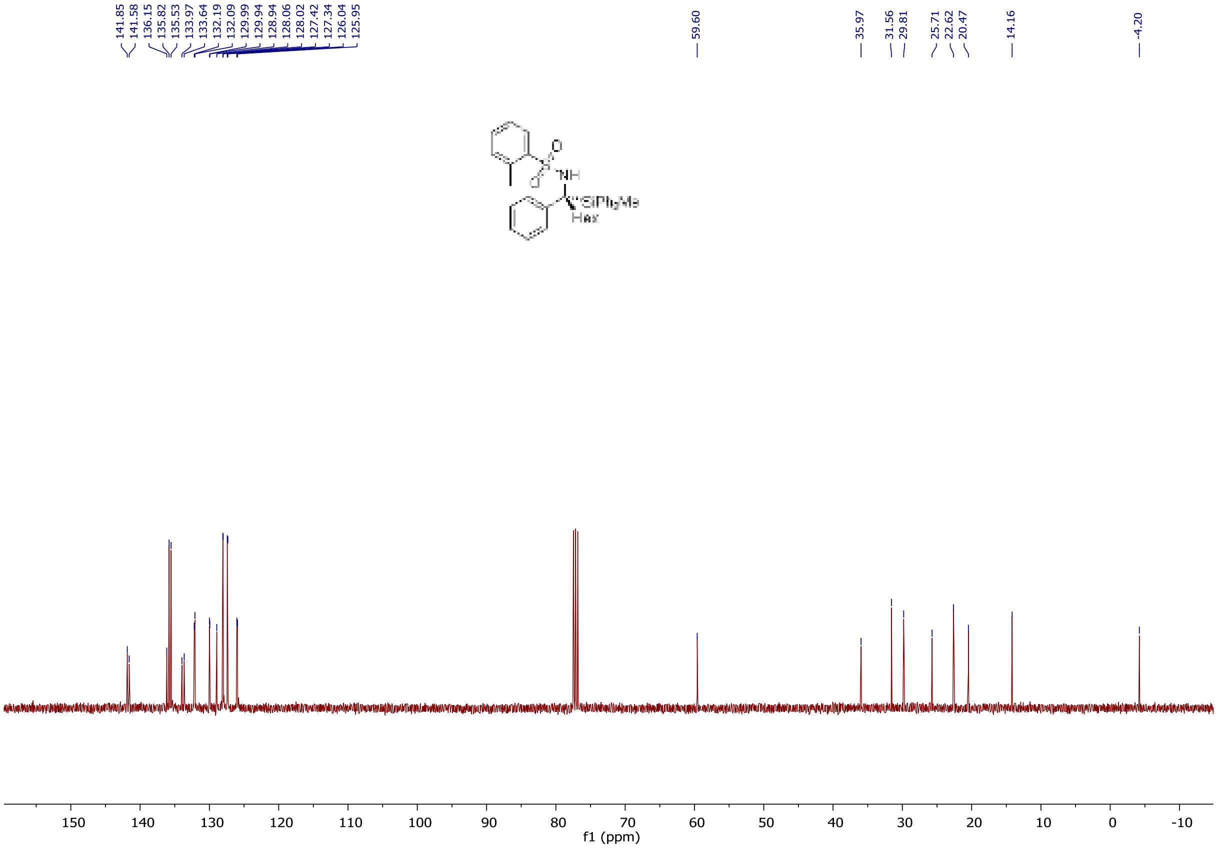

Supplementary Figure 49. 1H, 13C-NMR spectra of product **5d**.

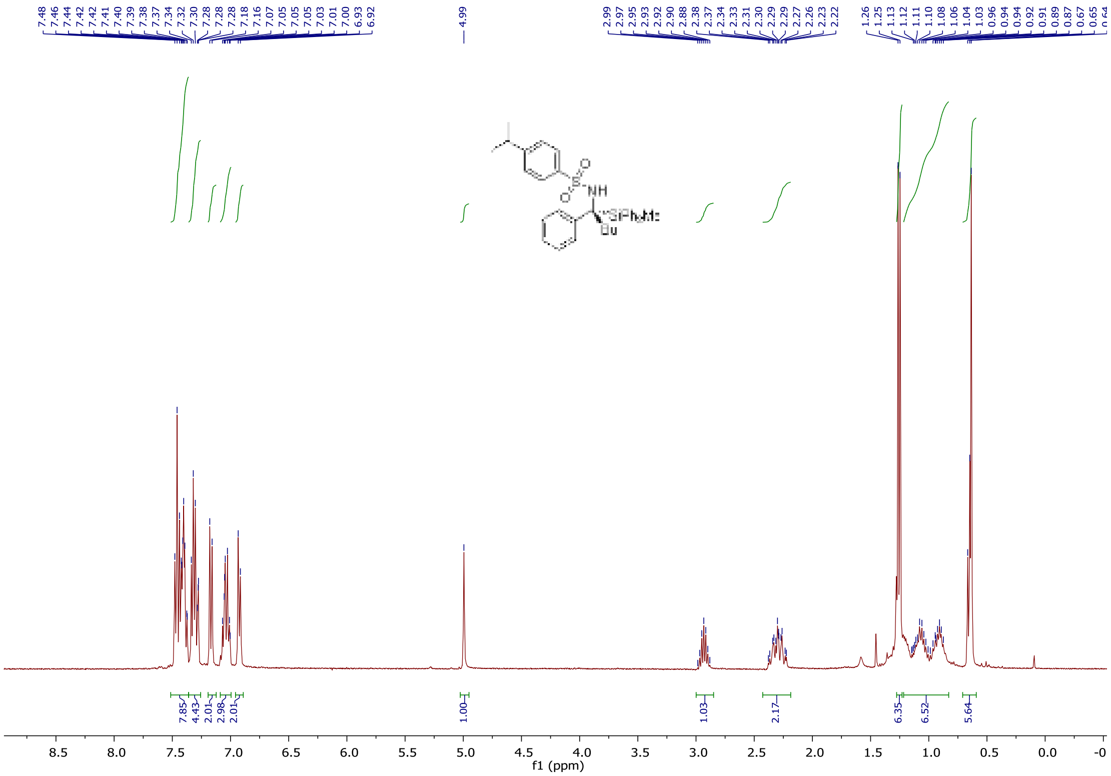

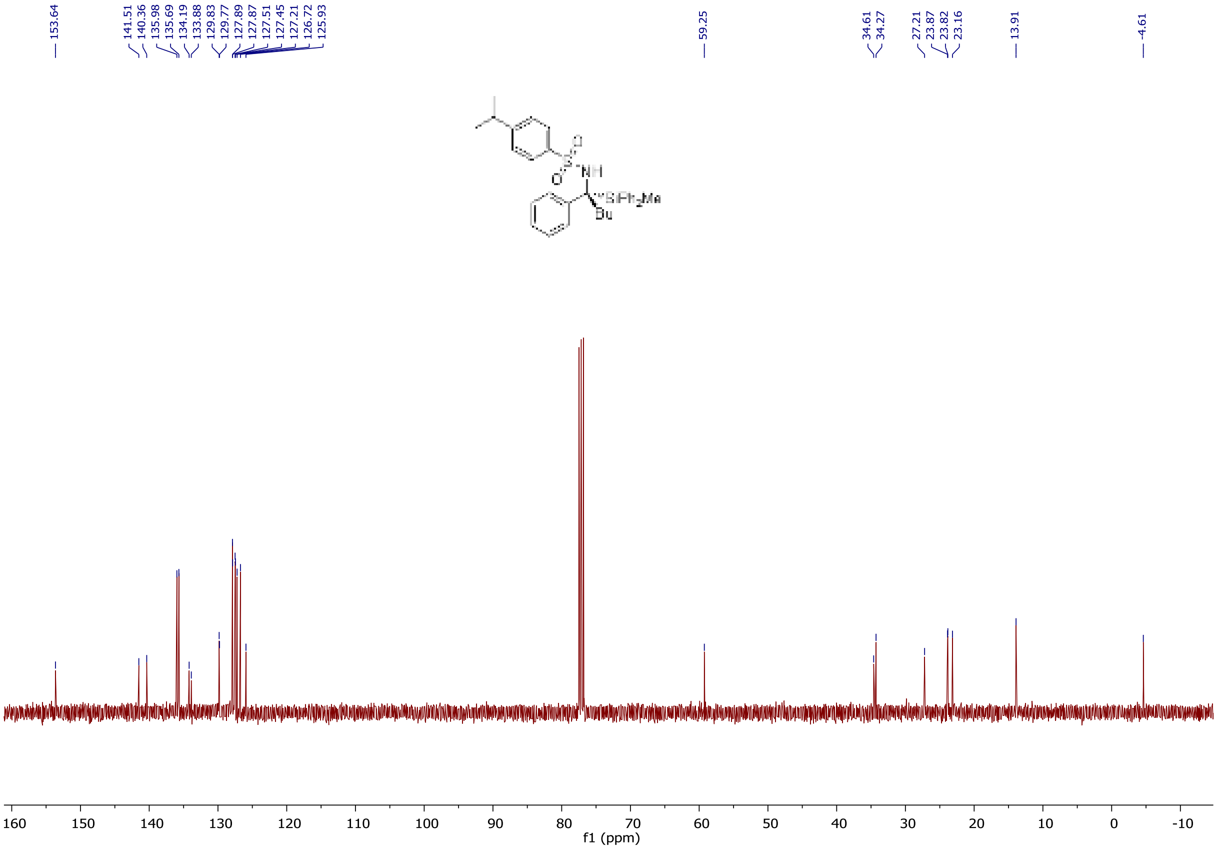

Supplementary Figure 50. 1H, 13C, 19F-NMR spectra of product **5e**

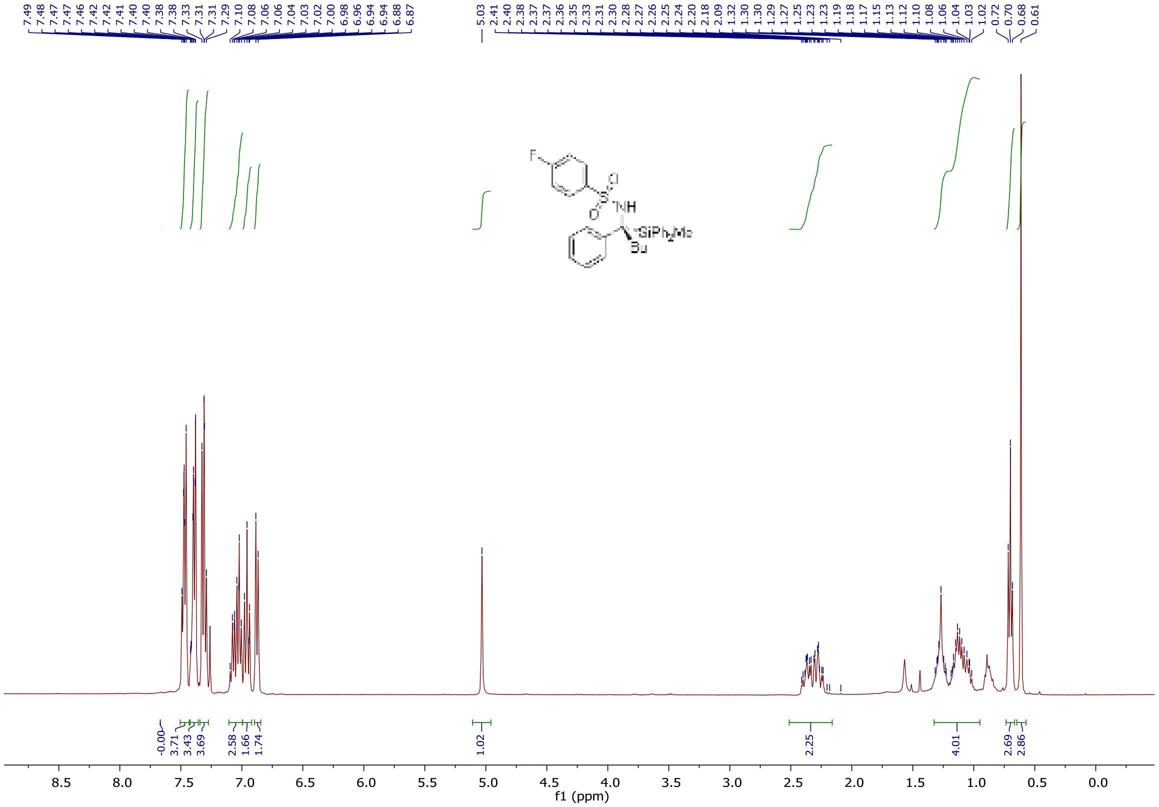

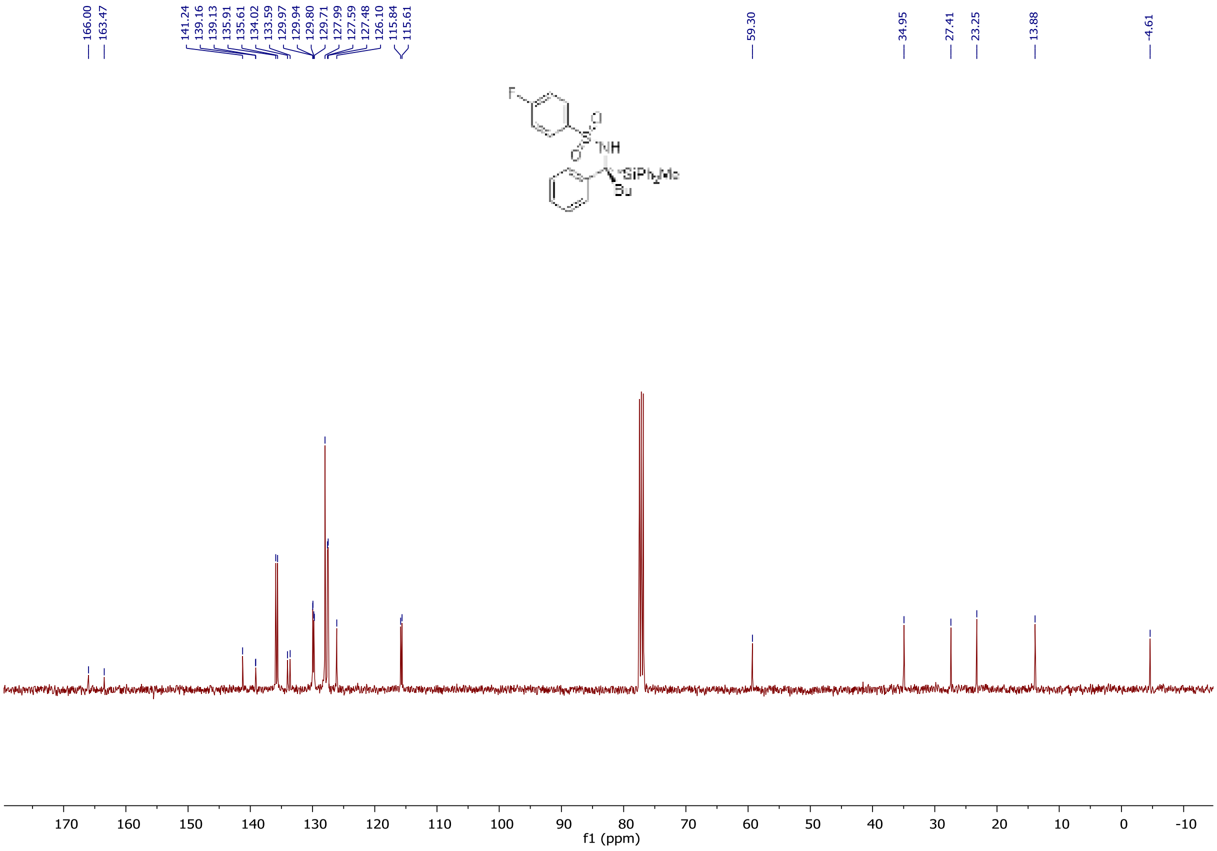

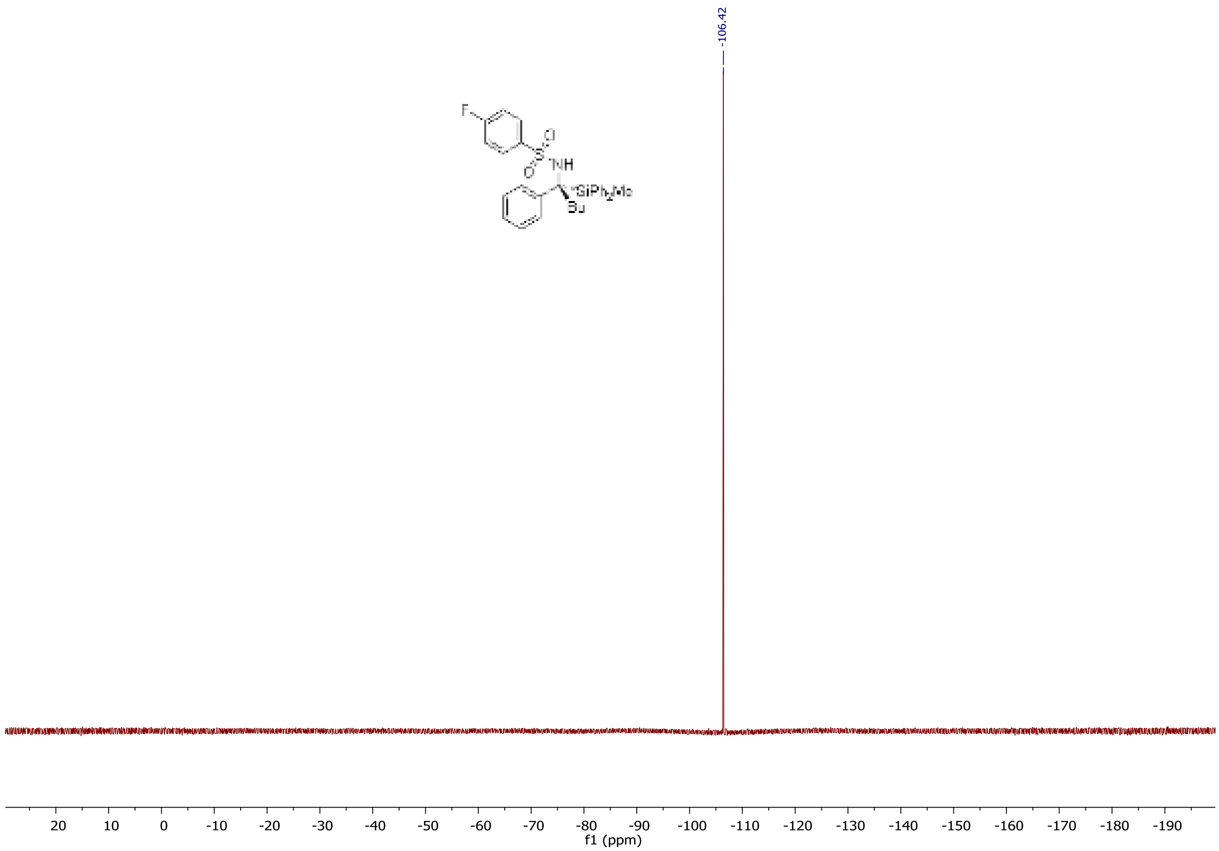

-106.42
F
O
S
NH
O
SiPh2Me
Bu
20
10
0
-10
-20
-30
-40
-50
-60
-70
-80
-90
-100
-110
-120
-130
-140
-150
-160
-170
-180
-190
f1 (ppm)

Supplementary Figure 51. 1H, 13C-NMR spectra of product **5f**.

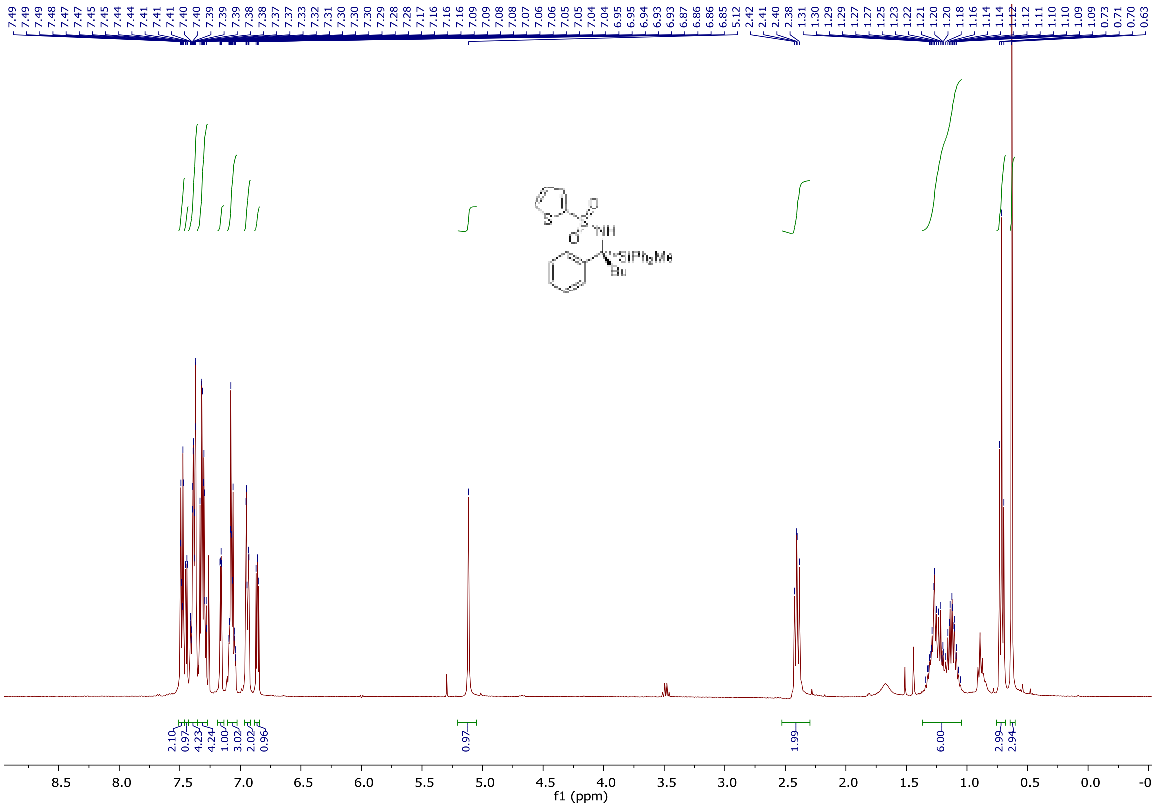

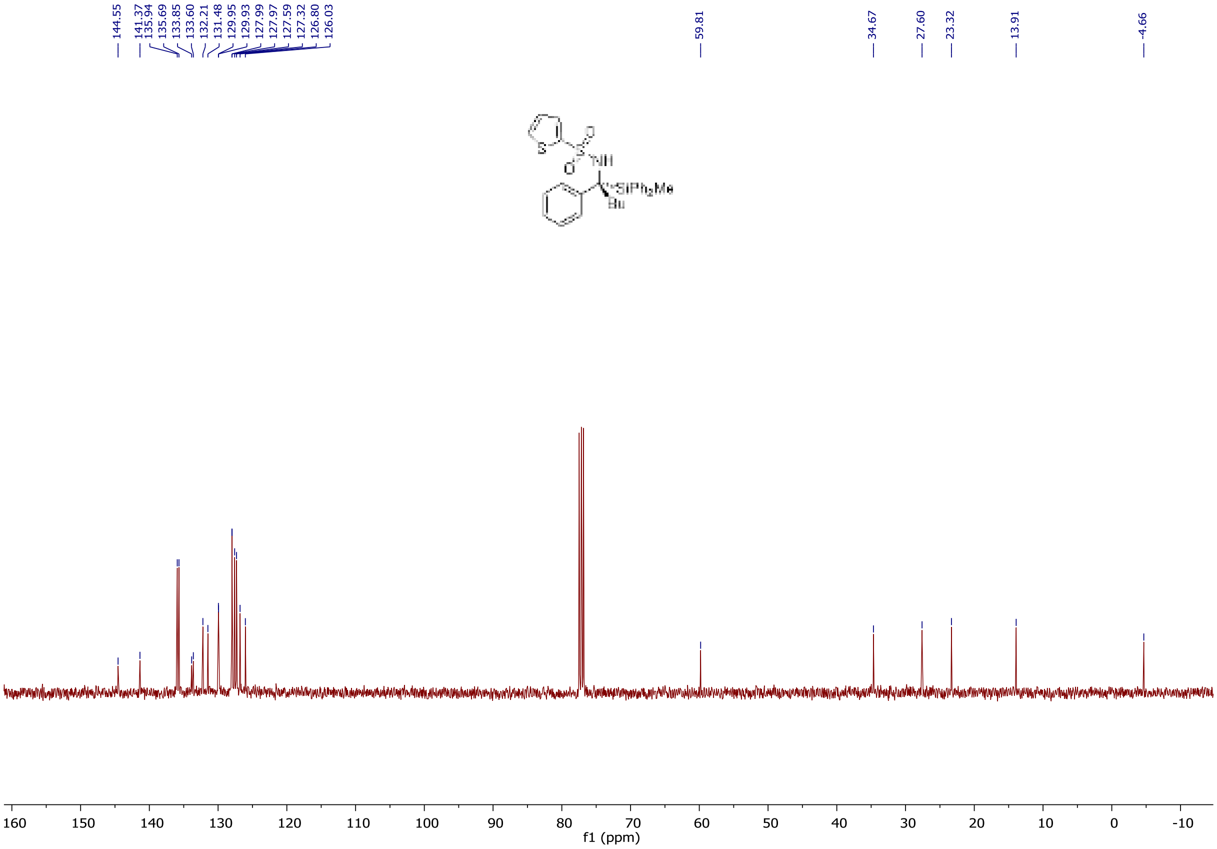

Supplementary Figure 52. 1H, 13C-NMR spectra of product **5g**.

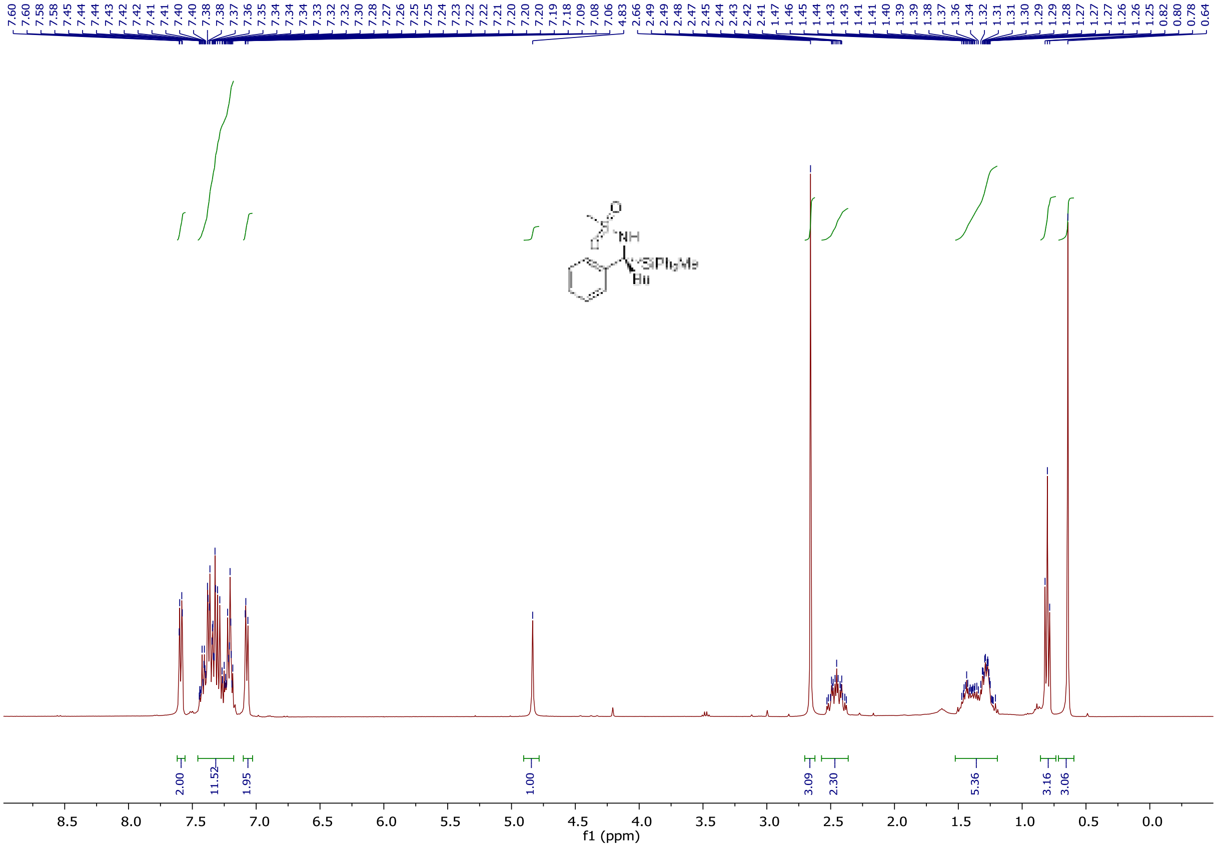

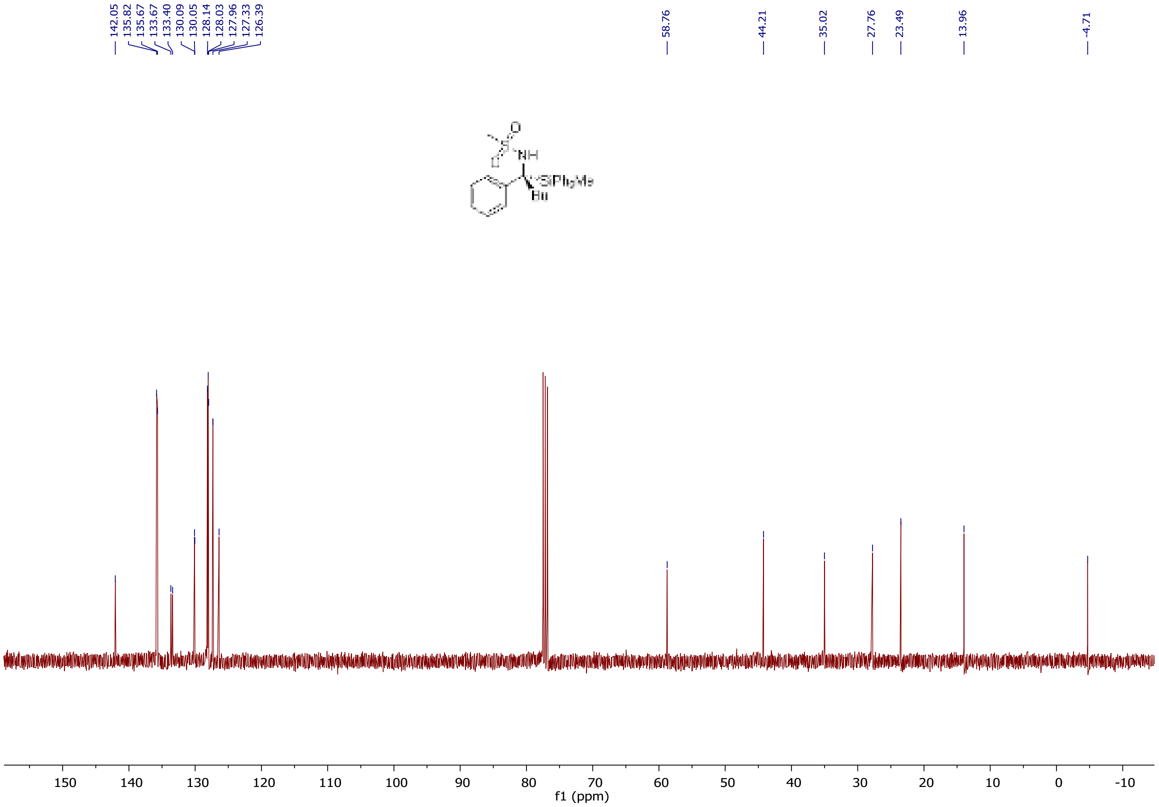

Supplementary Figure 53. 1H, 13C-NMR spectra of product **5h**

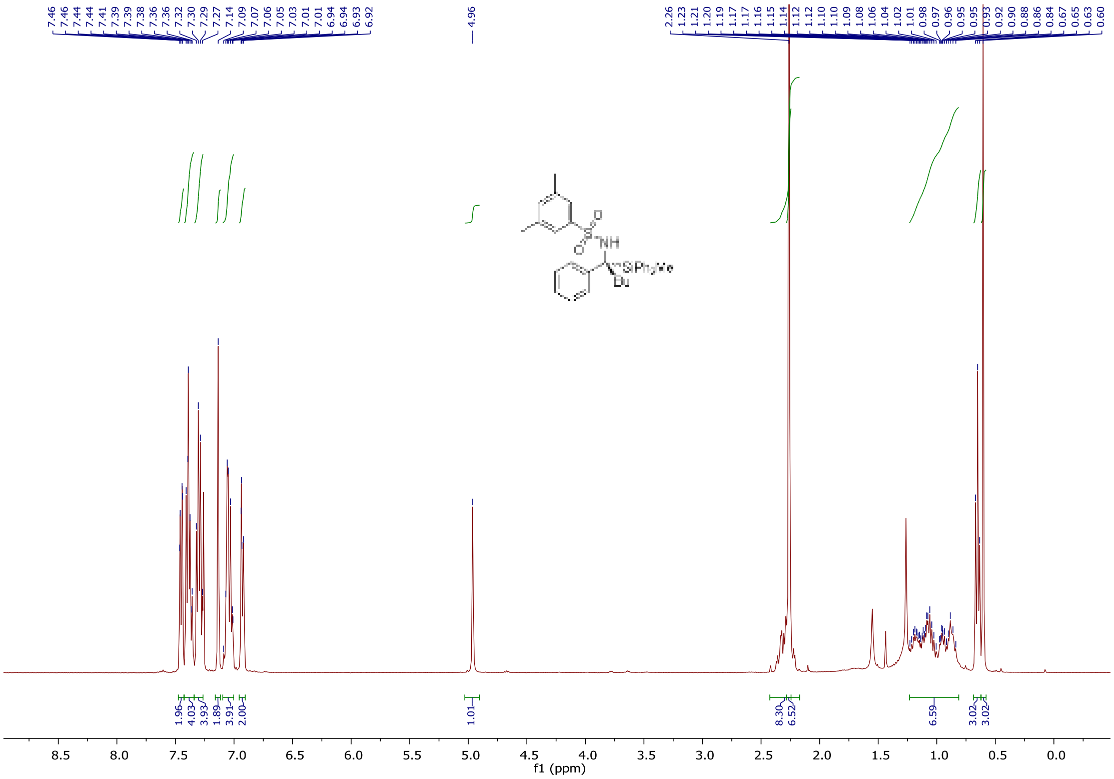

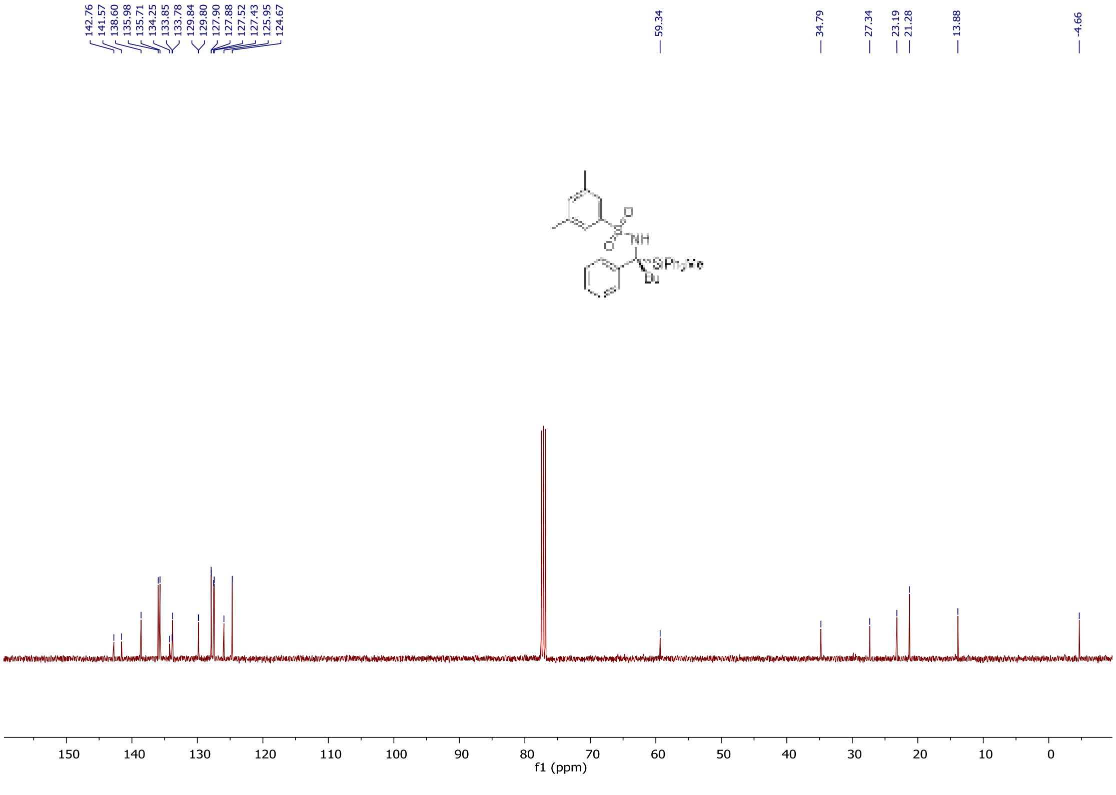

Supplementary Figure 54. 1H, 13C-NMR spectra of product **5i**

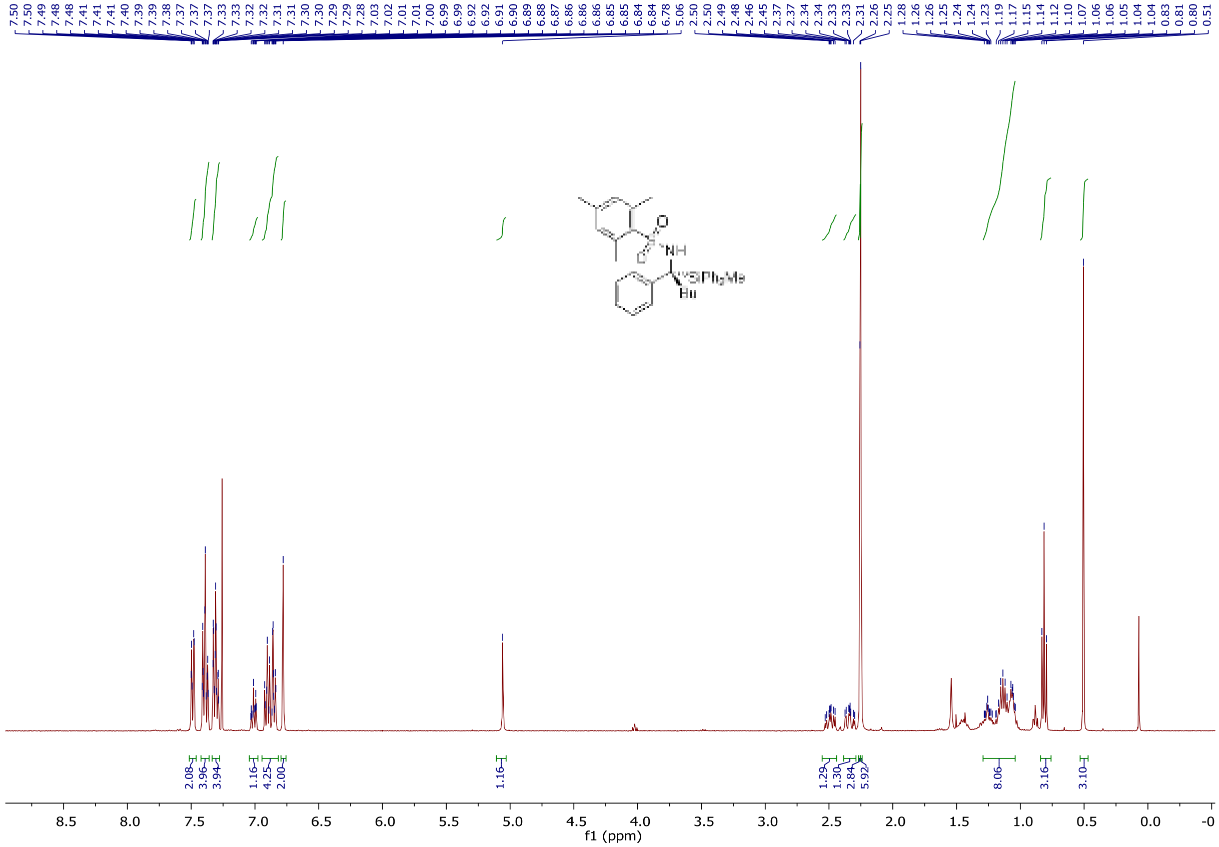

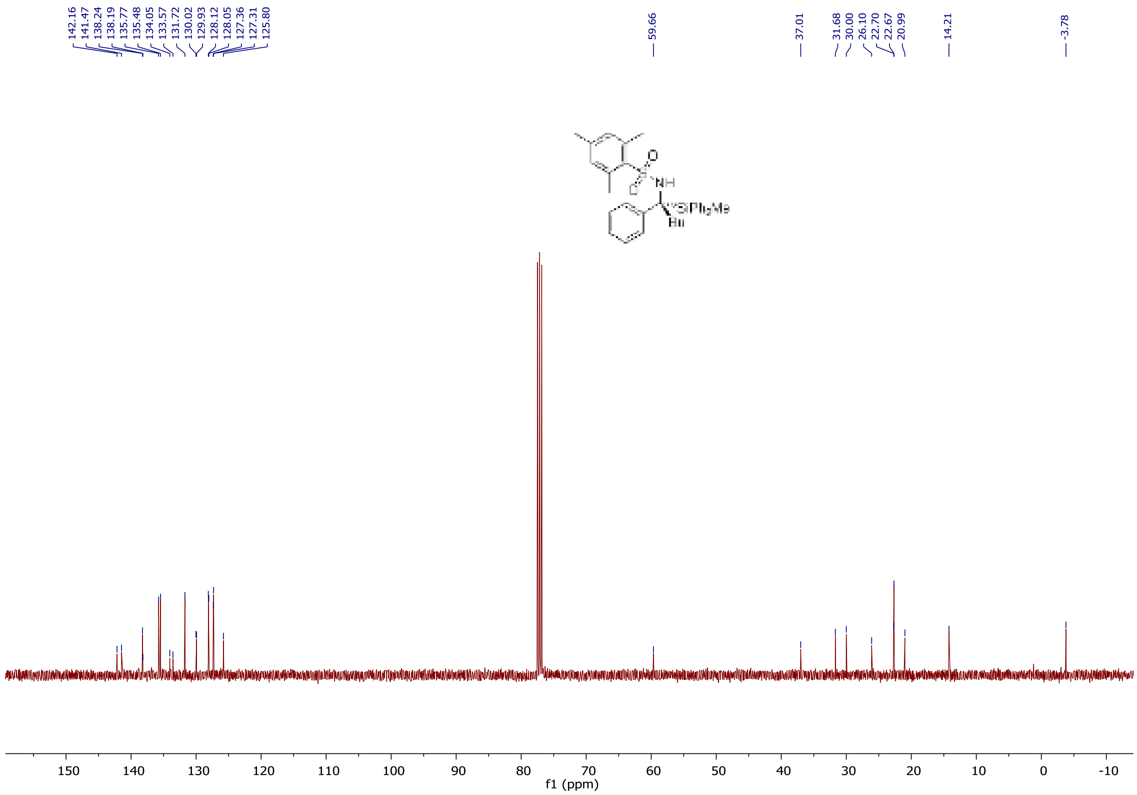

Supplementary Figure 55. 1H, 13C-NMR spectra of product **5j**

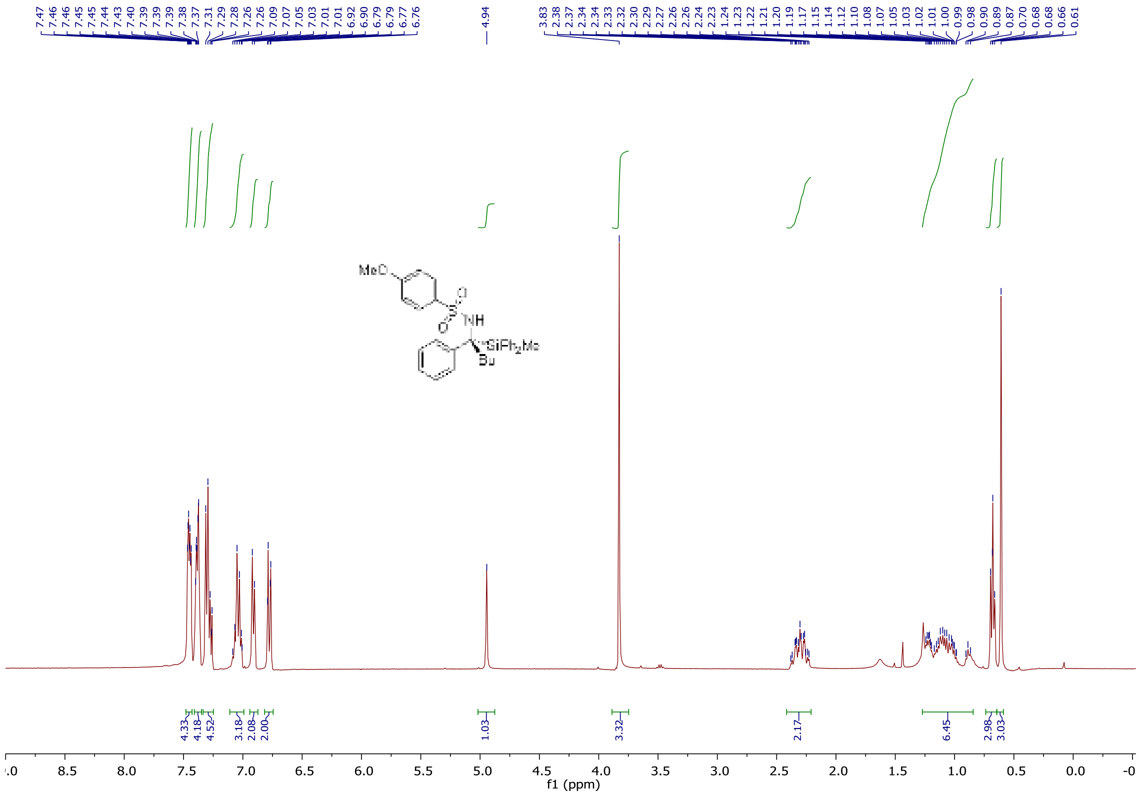

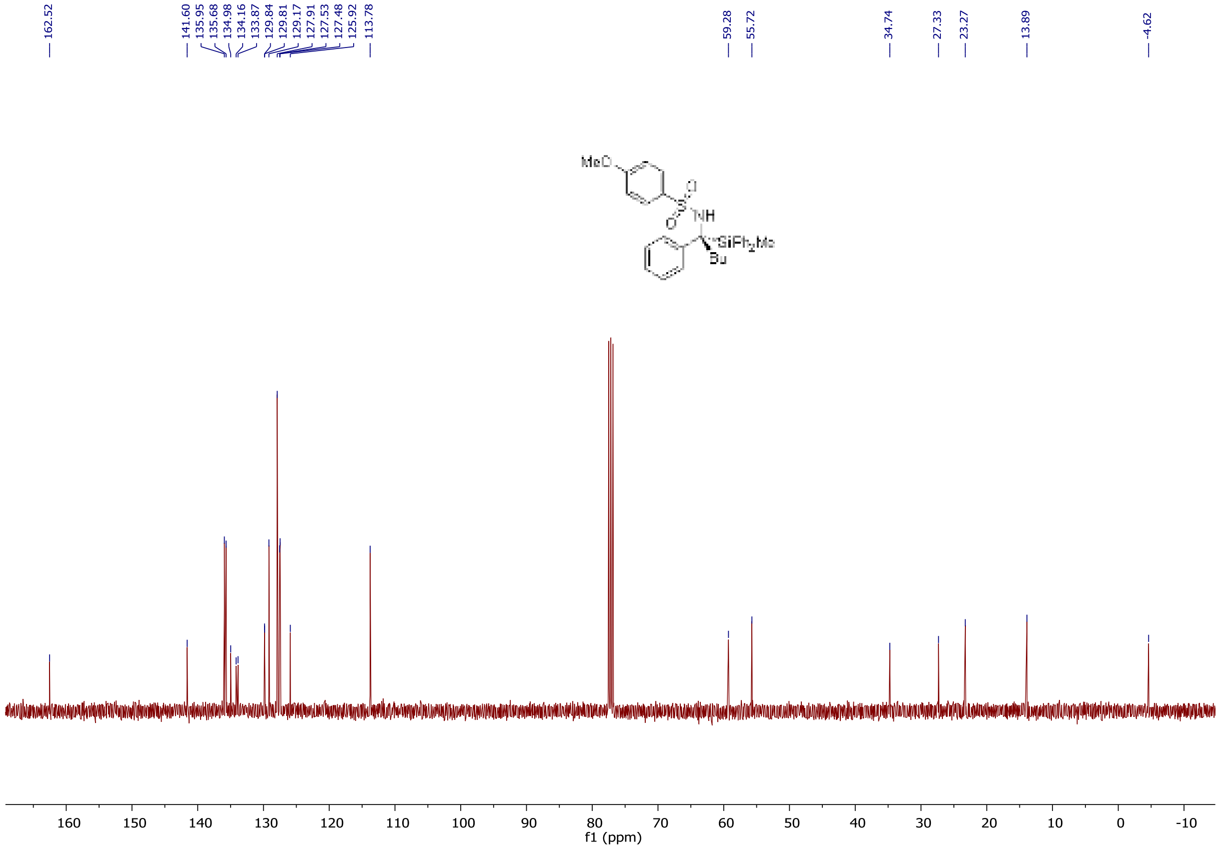

Supplementary Figure 56. 1H, 13C, 19F-NMR spectra of product **6b**

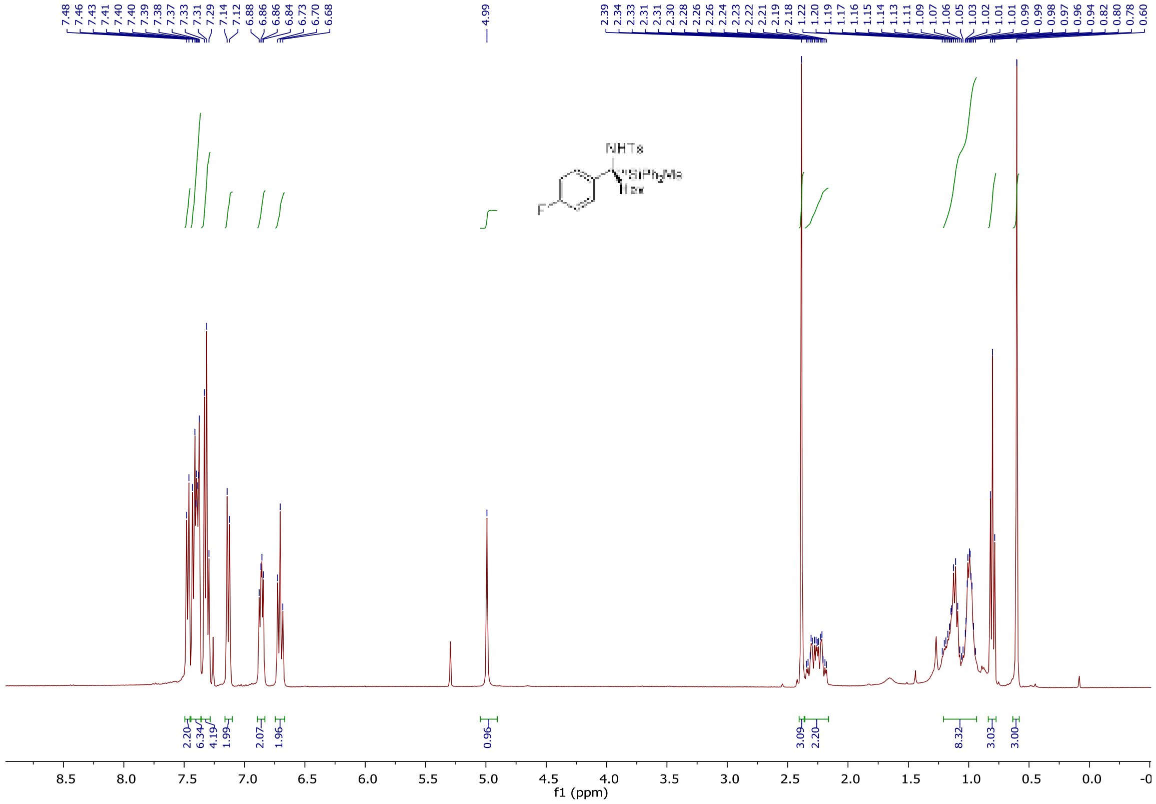

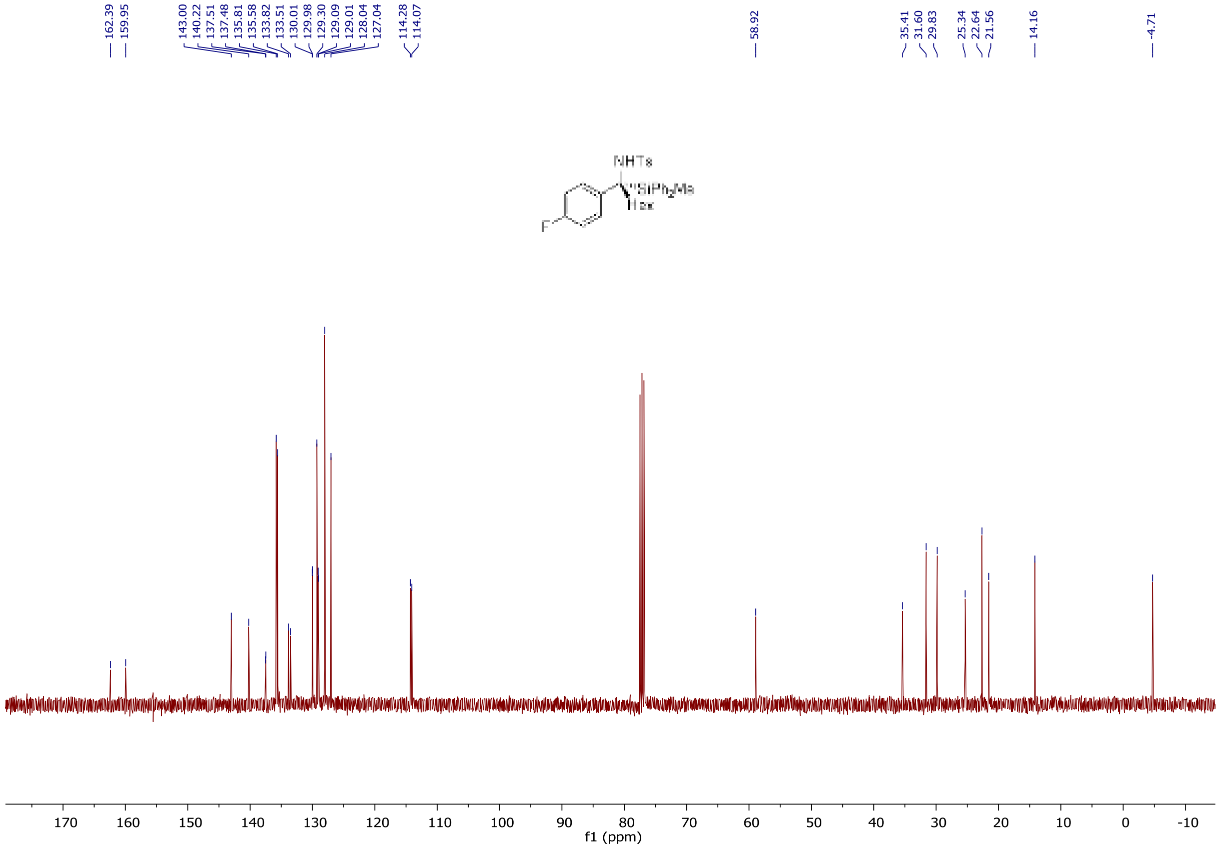

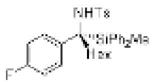
NHTs
SiPh2Me
Hex
F

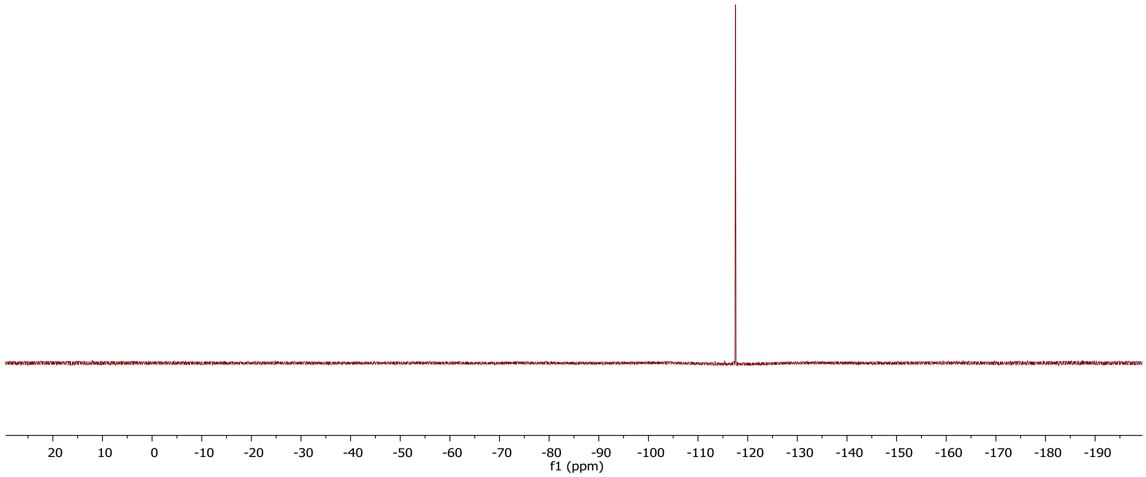
20 10 0 -10 -20 -30 -40 -50 -60 -70 -80 -90 -100 -110 -120 -130 -140 -150 -160 -170 -180 -190
f1 (ppm)

Supplementary Figure 57. 1H, 13C, 19F-NMR spectra of product **6c**

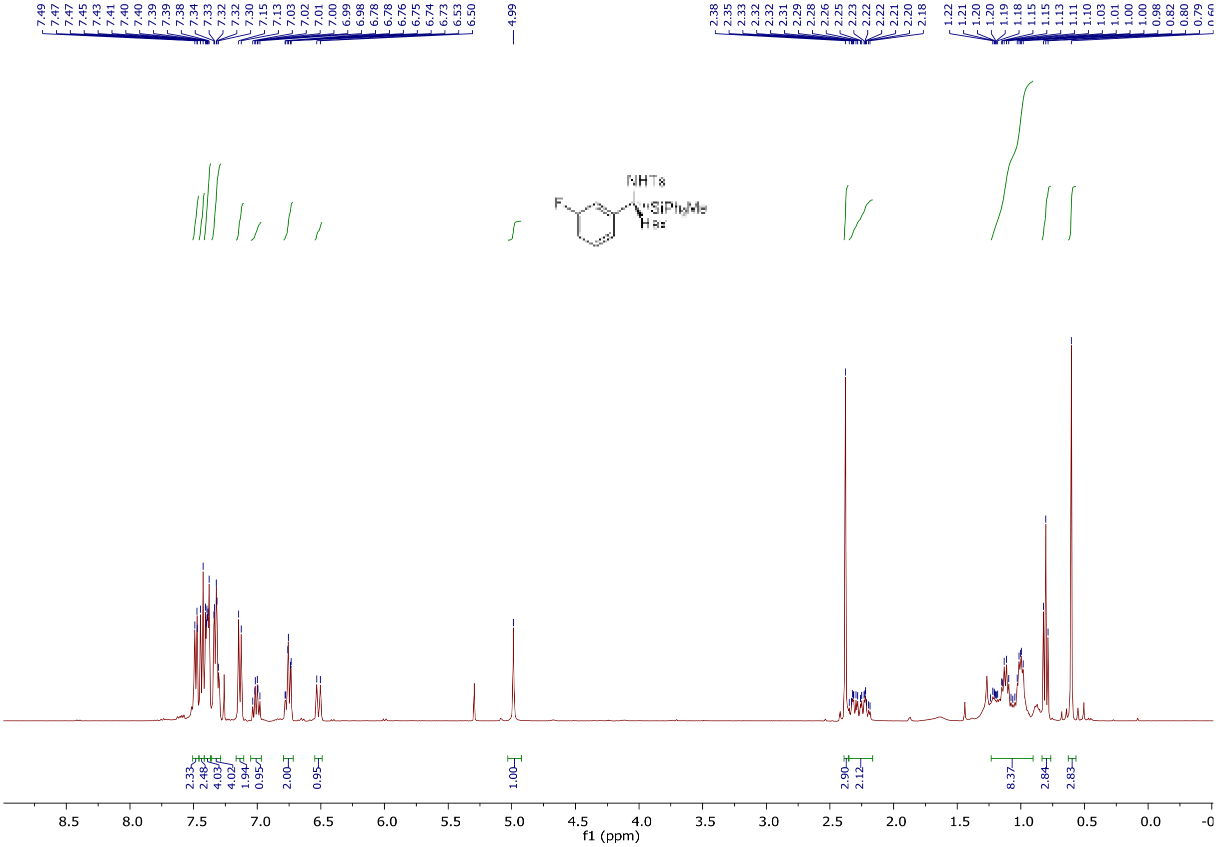

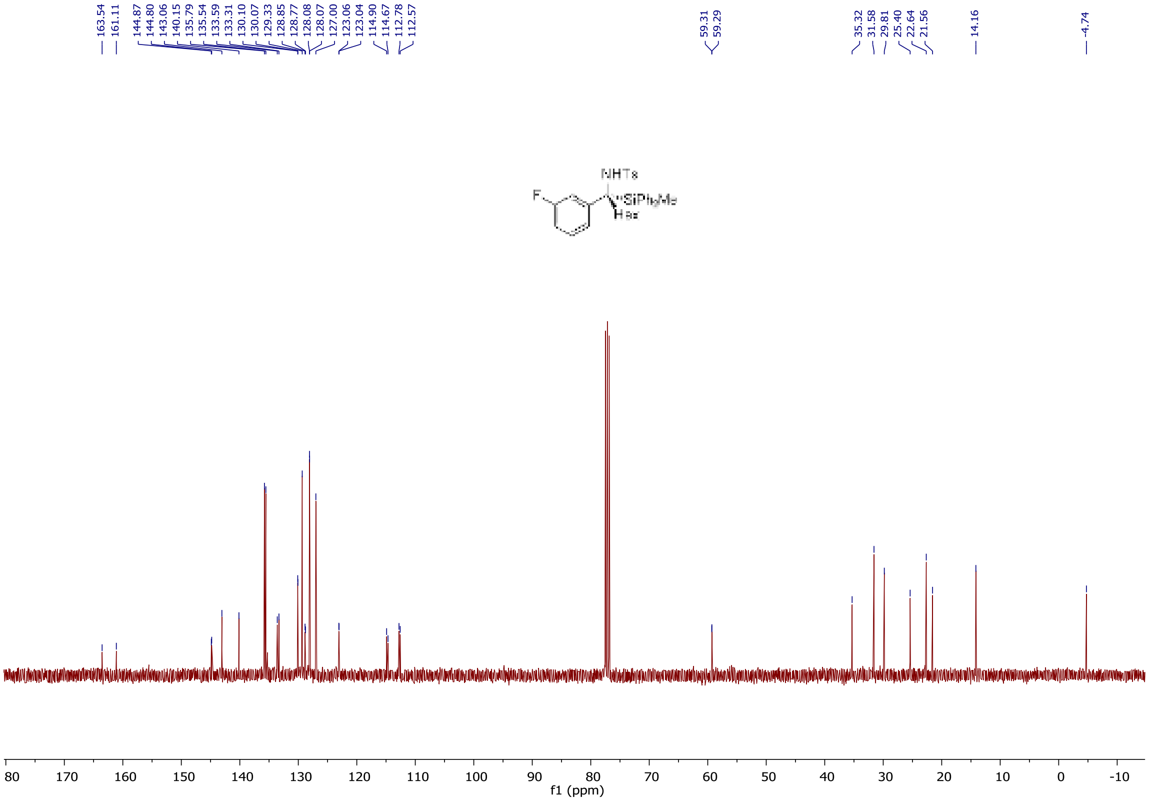

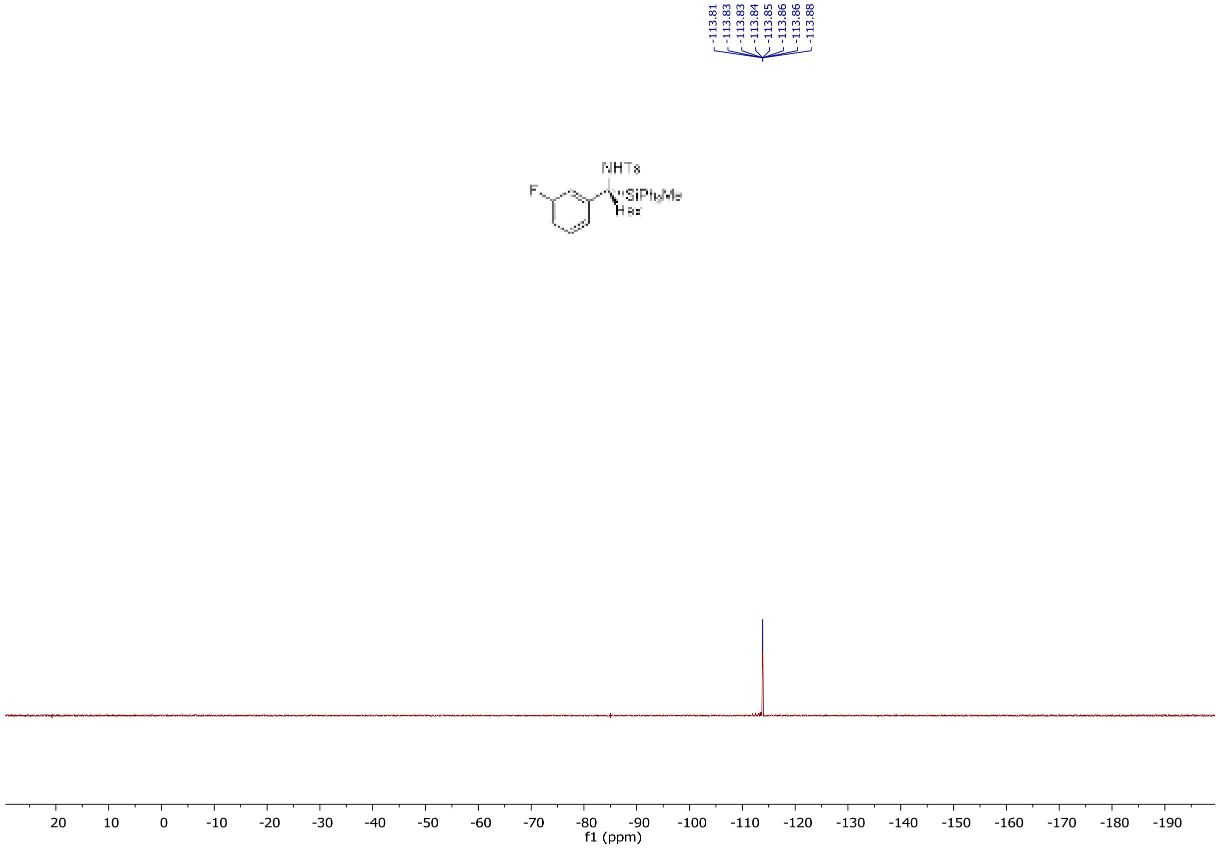
-113.81
-113.83
-113.83
-113.84
-113.85
-113.86
-113.86
-113.88
F
NHTs
20 10 0 -10 -20 -30 -40 -50 -60 -70 -80 -90 -100 -110 -120 -130 -140 -150 -160 -170 -180 -190
f1 (ppm)

Supplementary Figure 58. 1H, 13C, 19F-NMR spectra of product **6d.**

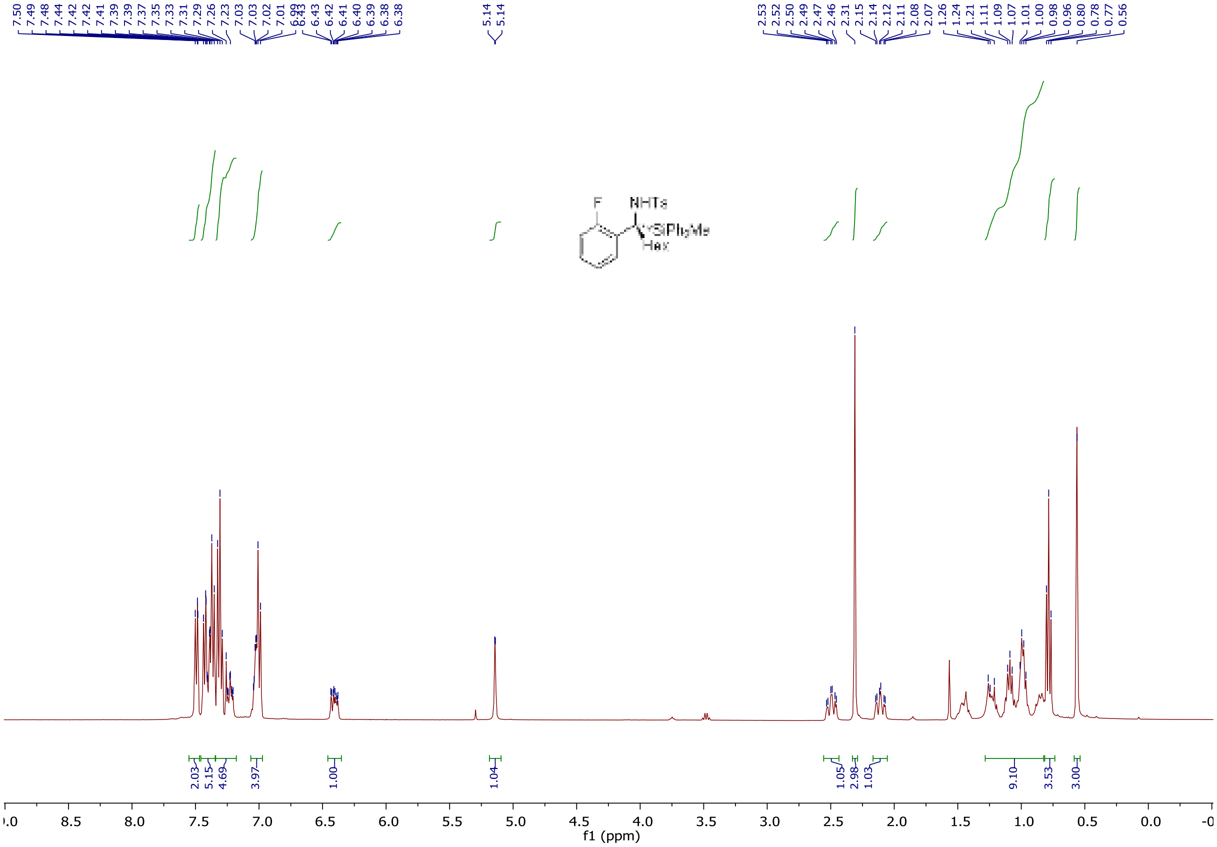

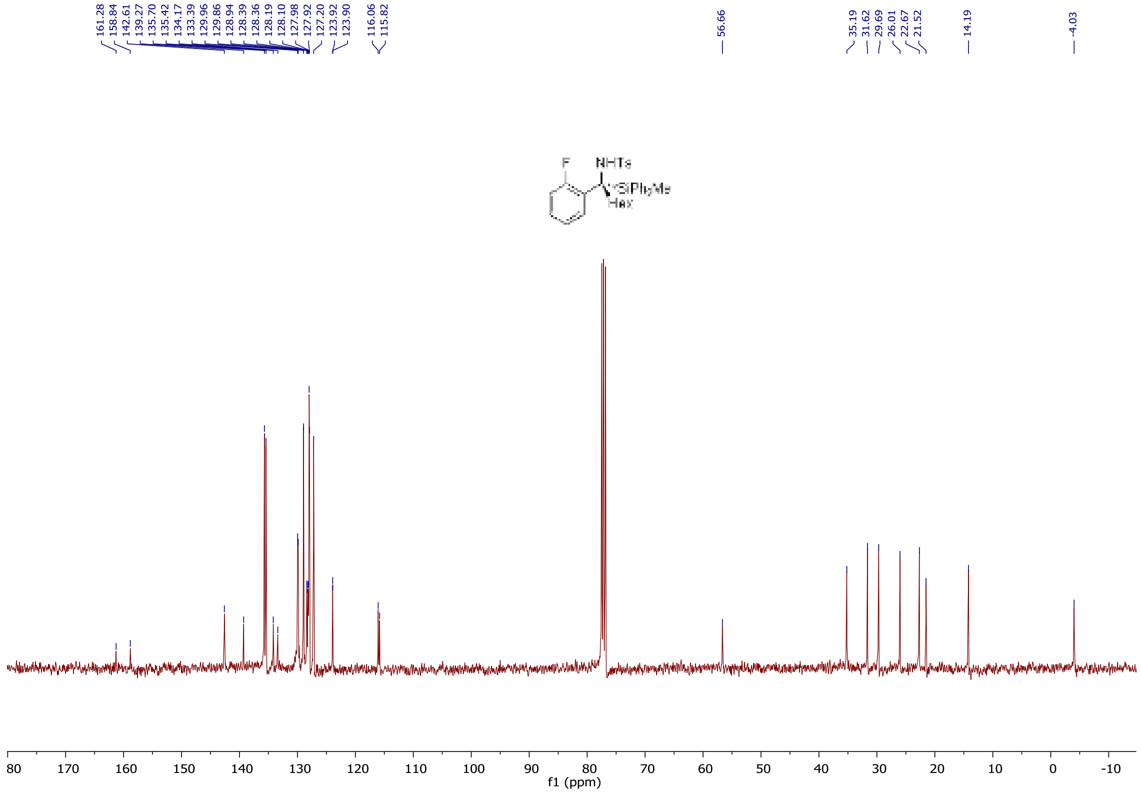

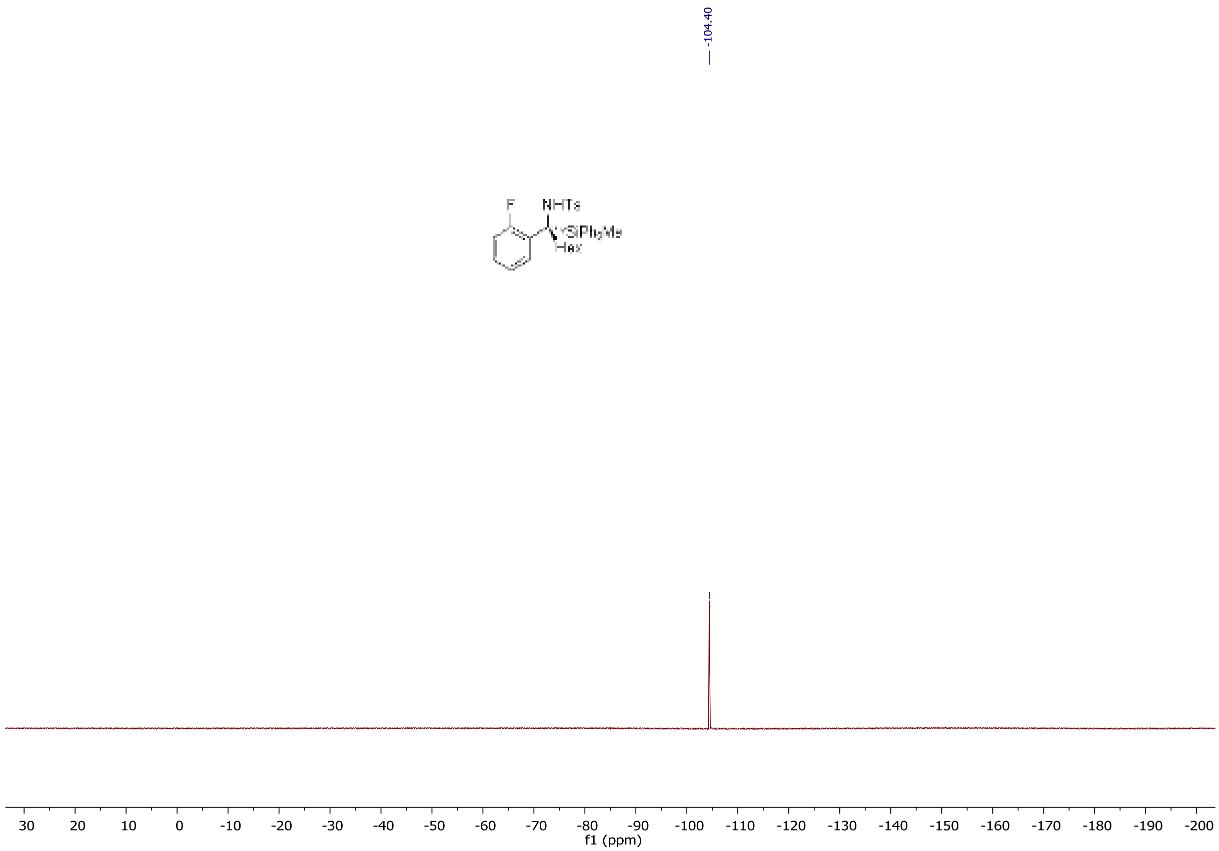
-104.40
F
NHTs
SiPh2Me
Hex
f1 (ppm)

Supplementary Figure 59. 1H, 13C-NMR spectra of product **6e**.

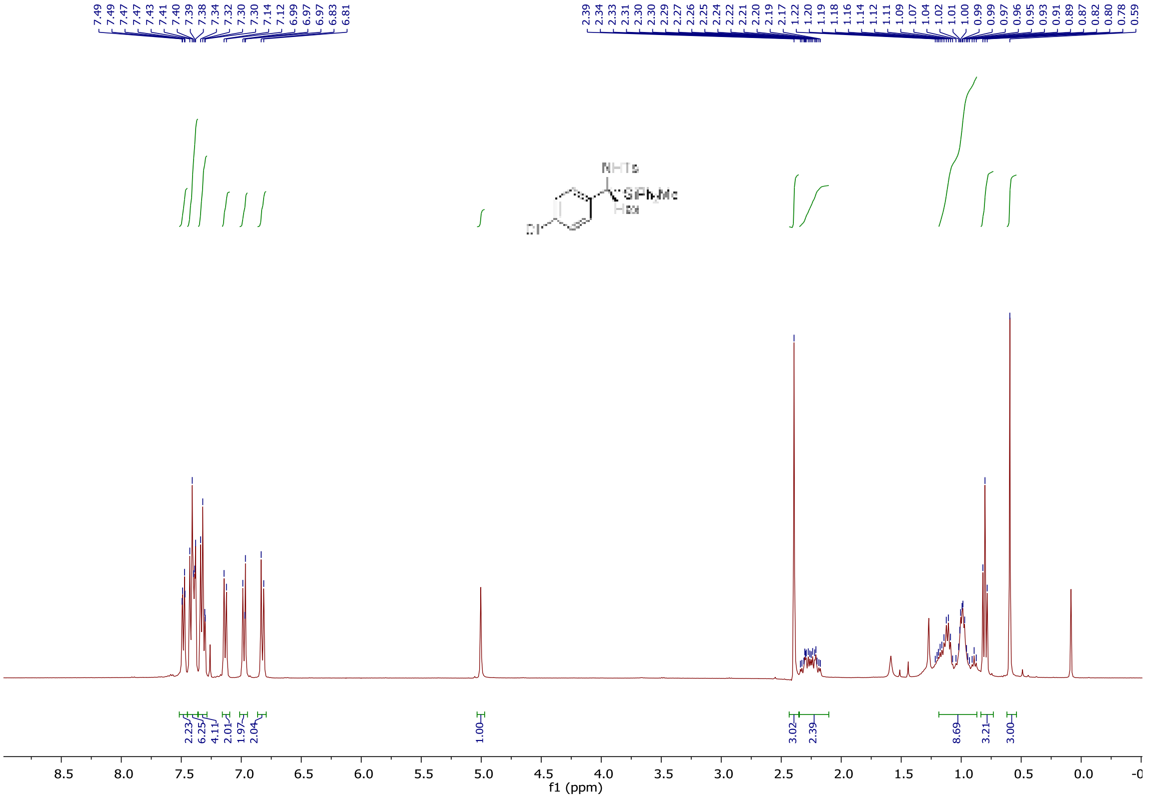

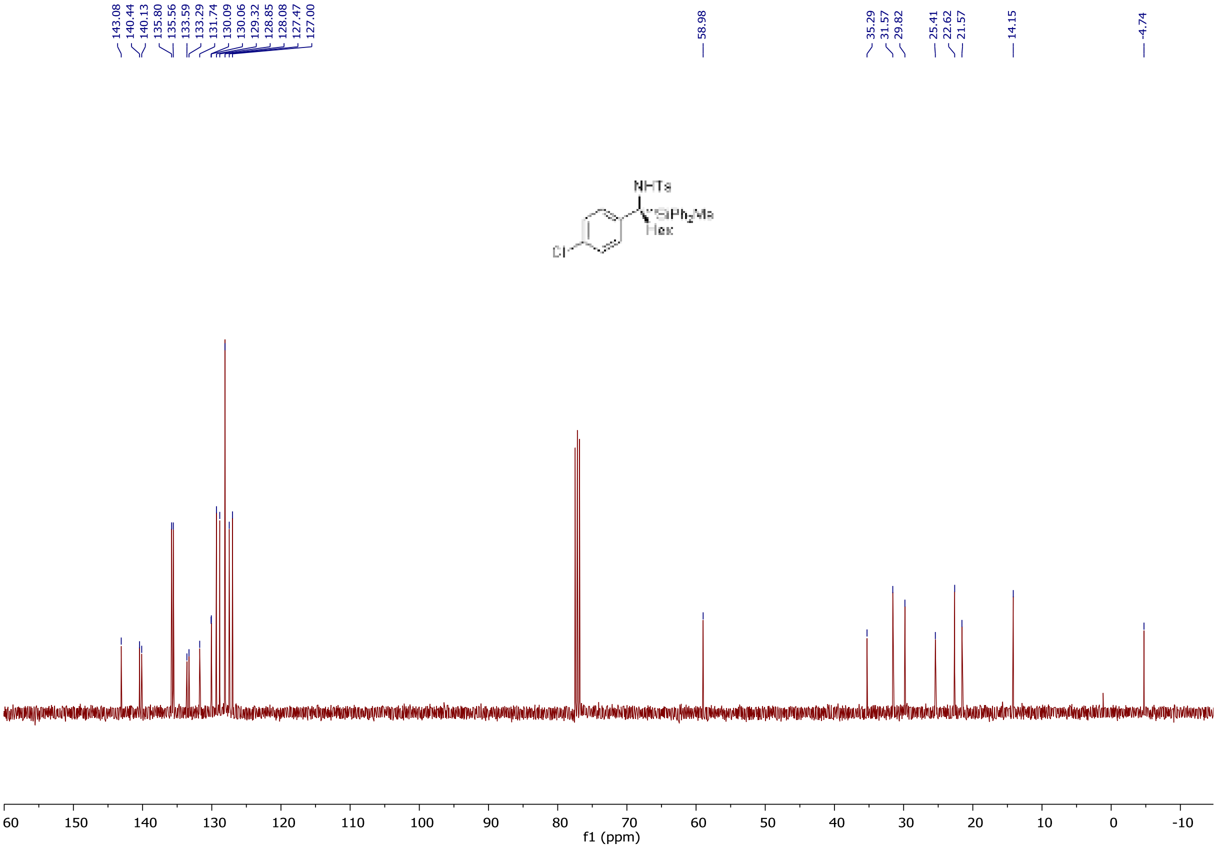

Supplementary Figure 60. 1H, 13C-NMR spectra of product **6f**

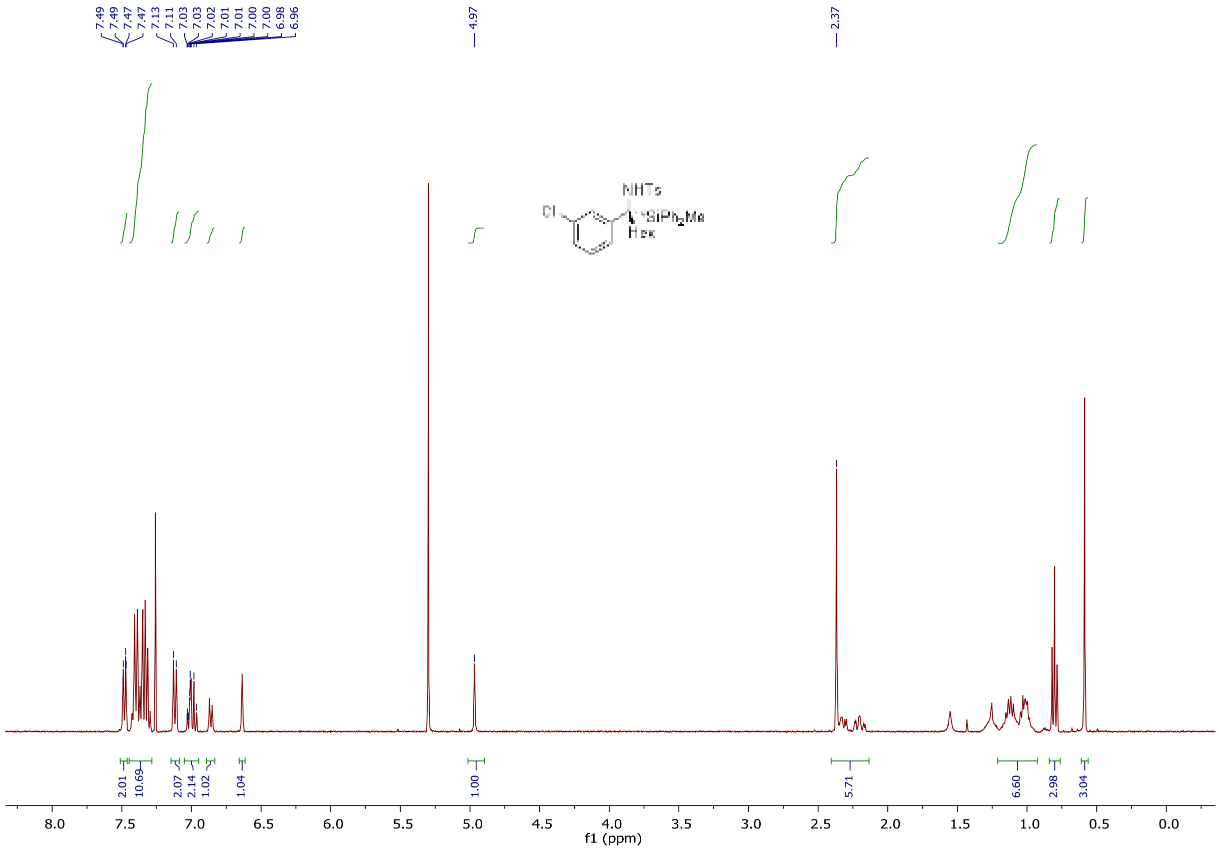

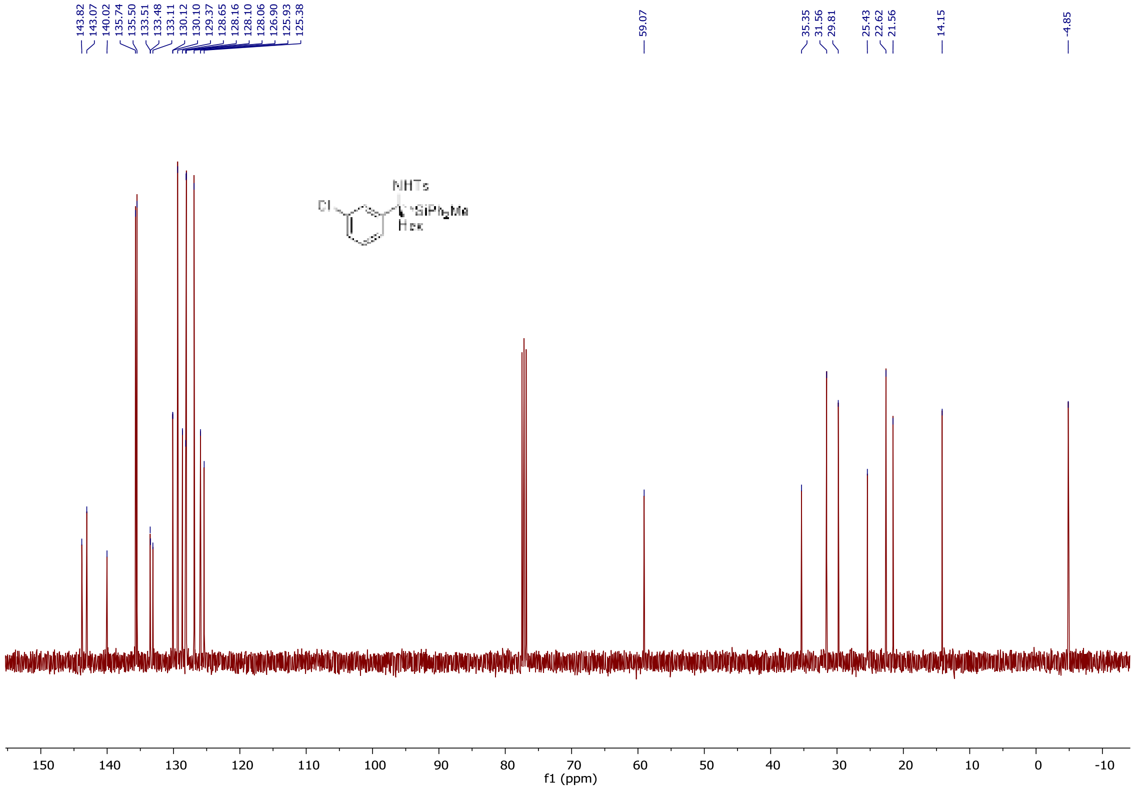

Supplementary Figure 61. 1H, 13C-NMR spectra of product **6g**.

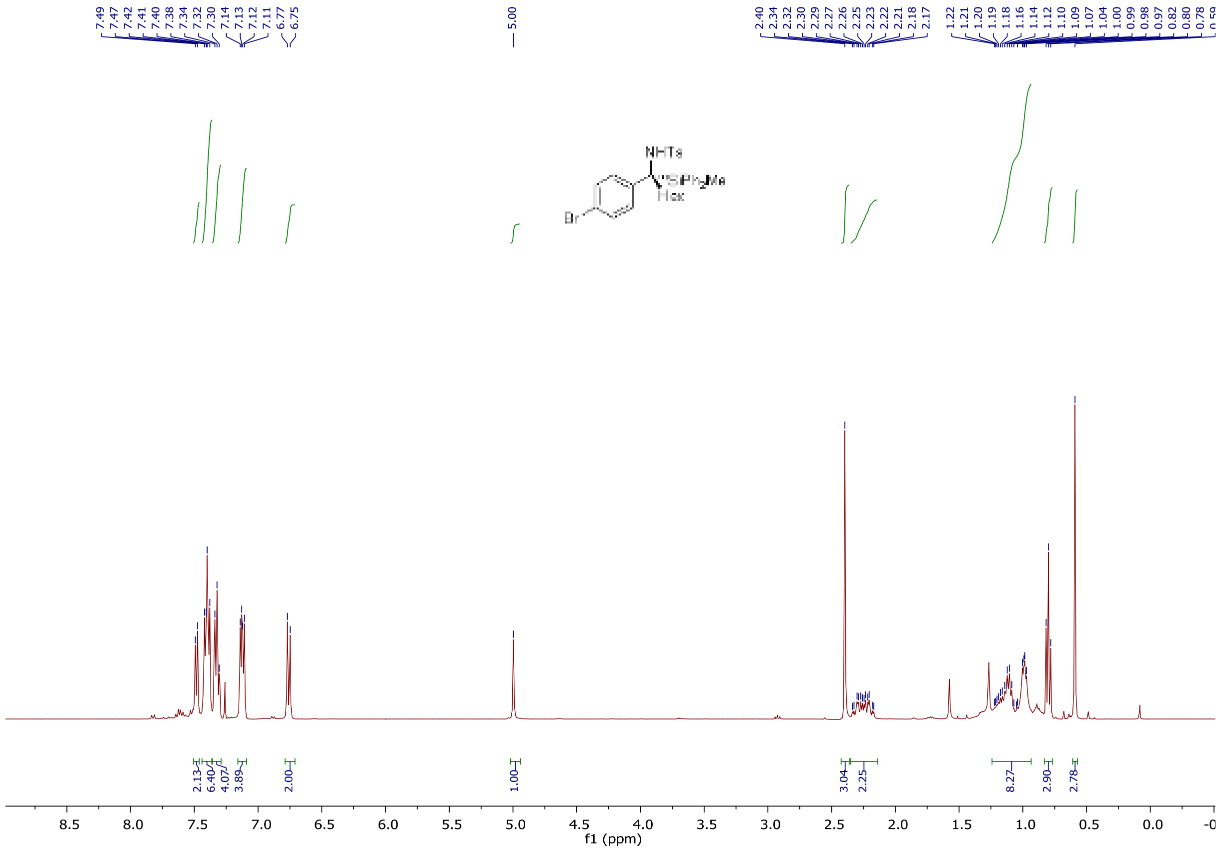

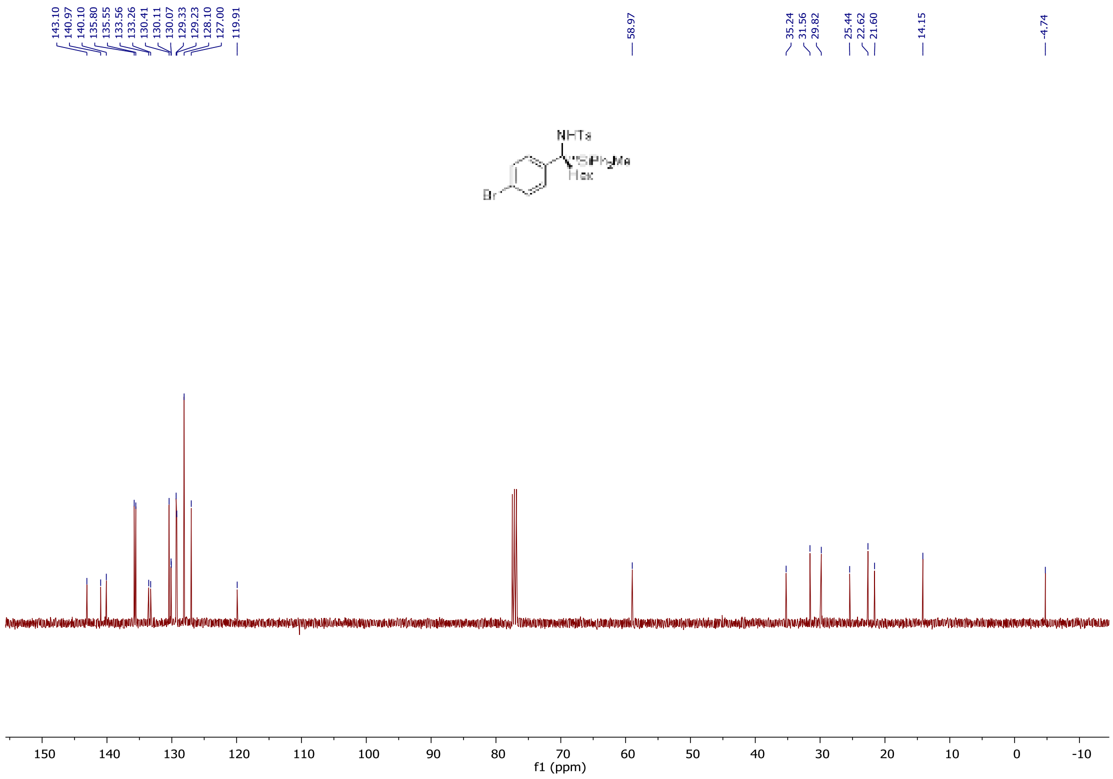

Supplementary Figure 62. 1H, 13C-NMR spectra of product **6h**.

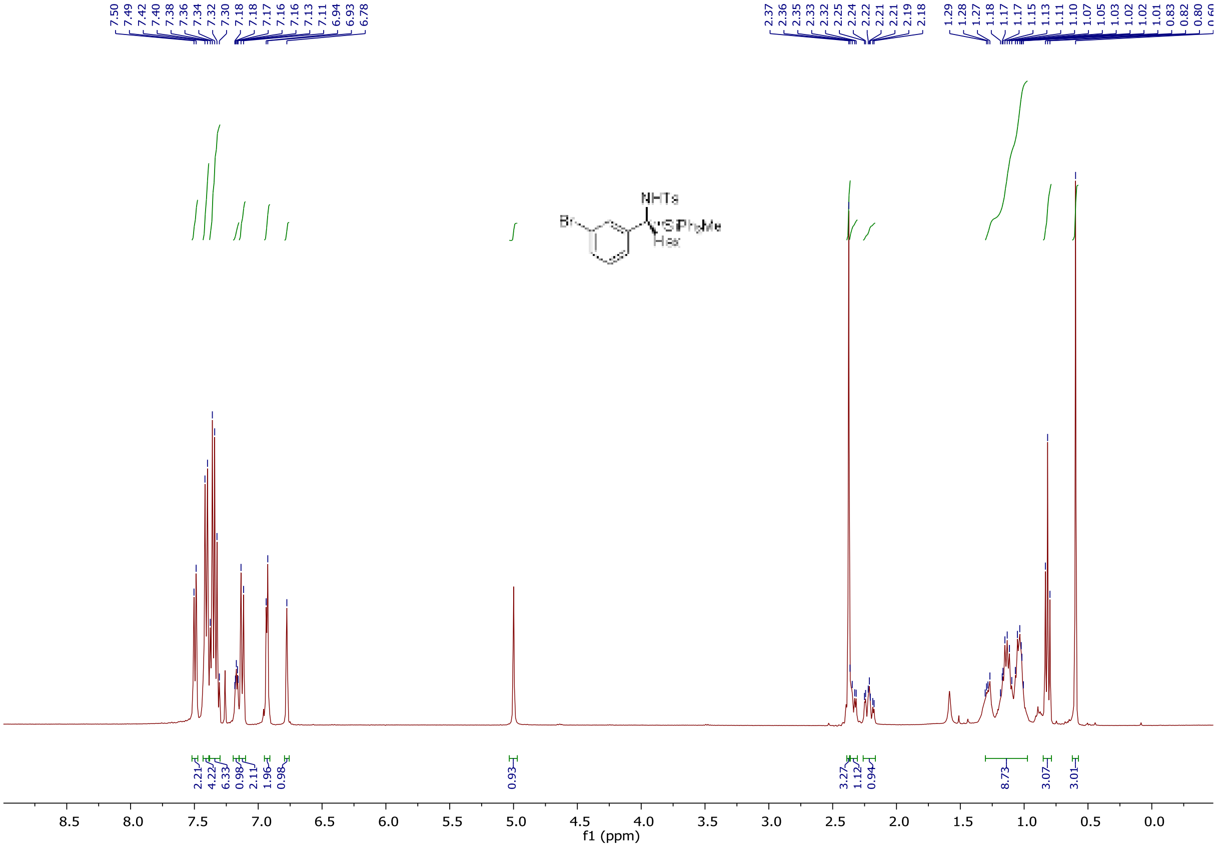

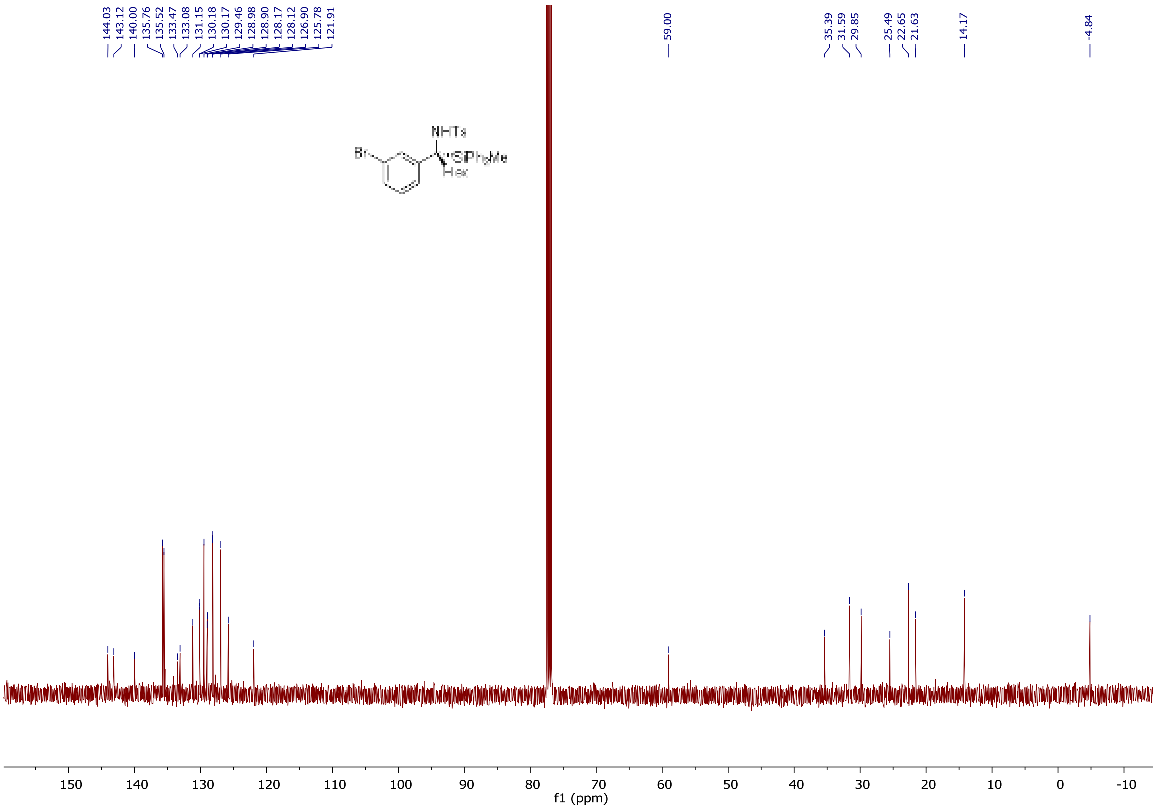

Supplementary Figure 63. 1H, 13C, 19F-NMR spectra of product **6i**.

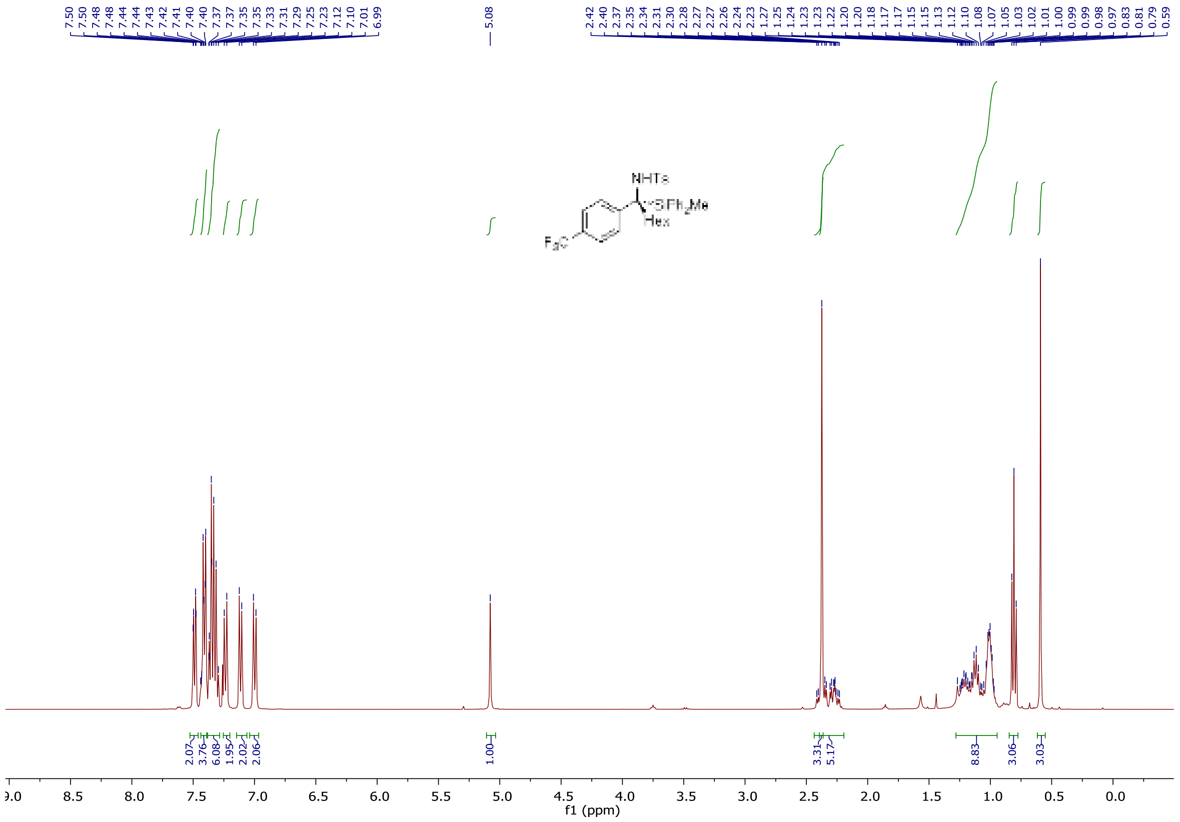

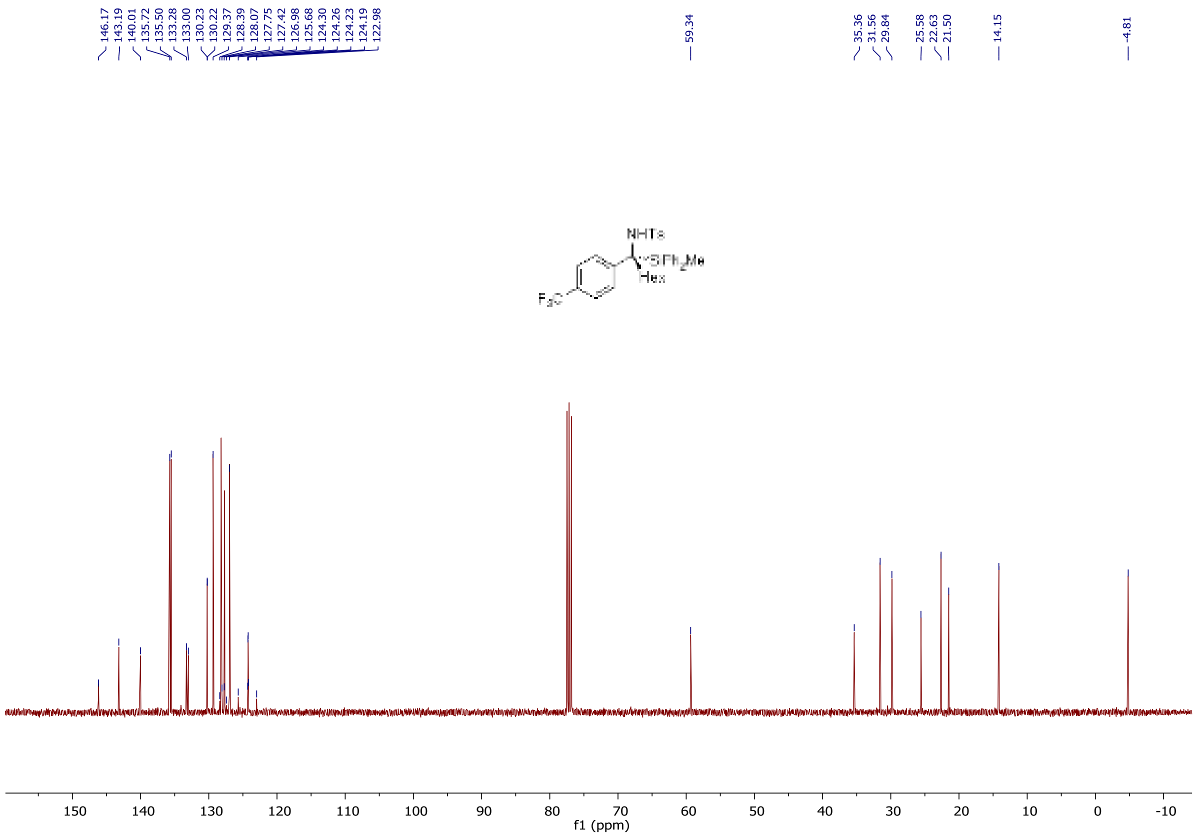

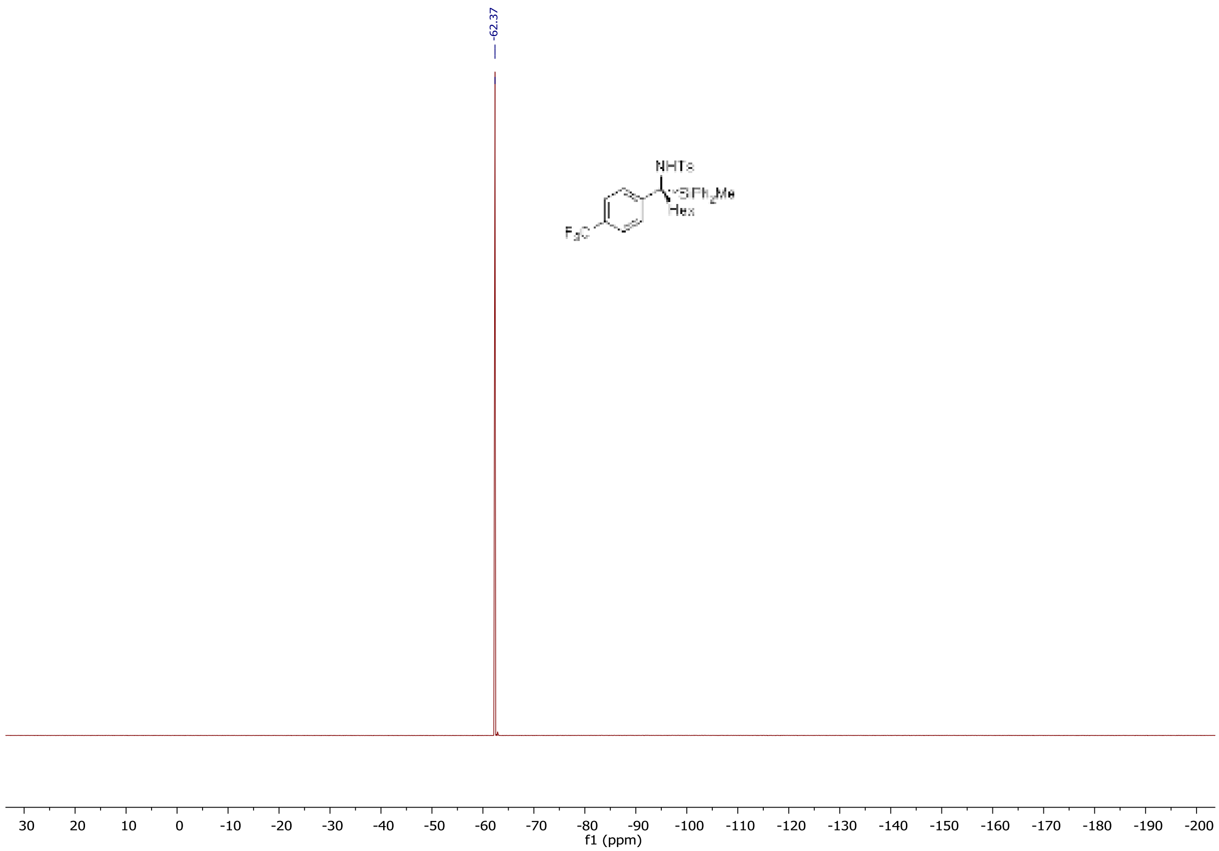
-62.37
NHTs
SiPh2Me
Hex
F3C
30
20
10
0
-10
-20
-30
-40
-50
-60
-70
-80
-90
-100
-110
-120
-130
-140
-150
-160
-170
-180
-190
-200
f1 (ppm)

Supplementary Figure 64. 1H, 13C, 19F-NMR spectra of product **6j**.

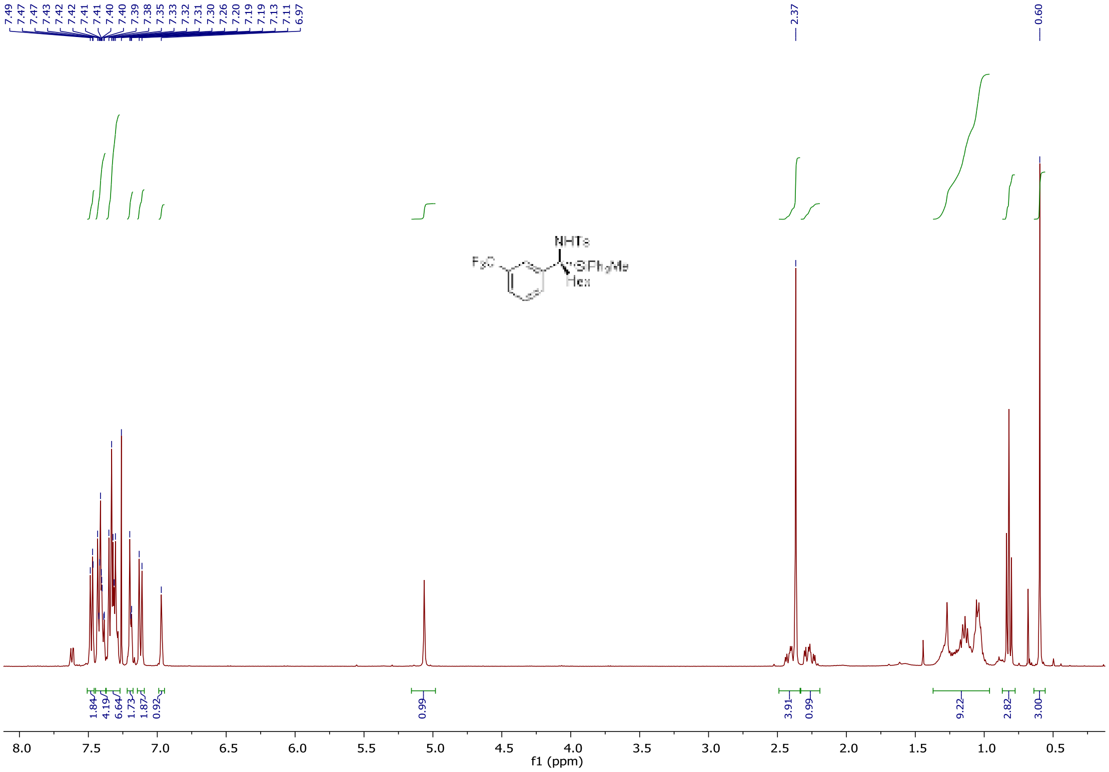

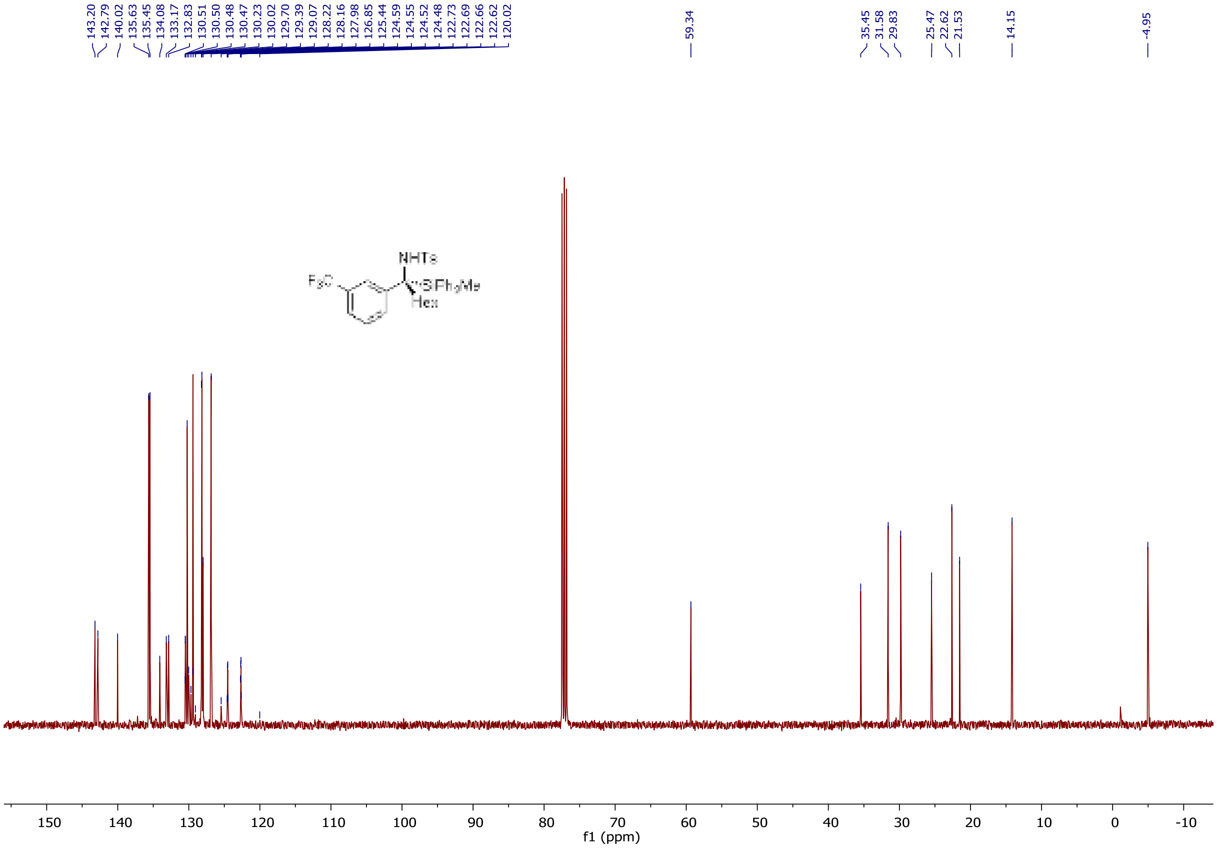

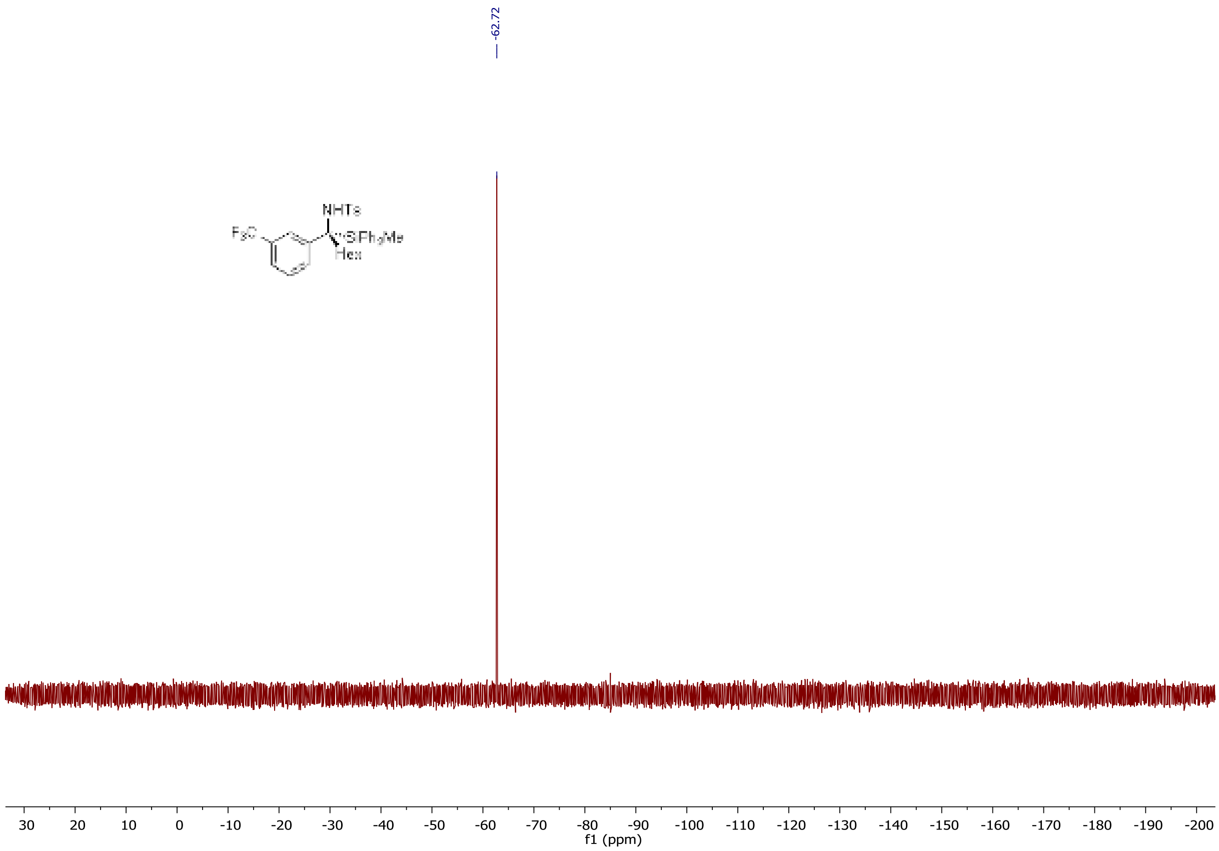

-62.72
NHTs
F3C
SiPh2Me
Hex
30
20
10
0
-10
-20
-30
-40
-50
-60
-70
-80
-90
-100
-110
-120
-130
-140
-150
-160
-170
-180
-190
-200
f1 (ppm)

Supplementary Figure 65. 1H, 13C-NMR spectra of product **6k.**

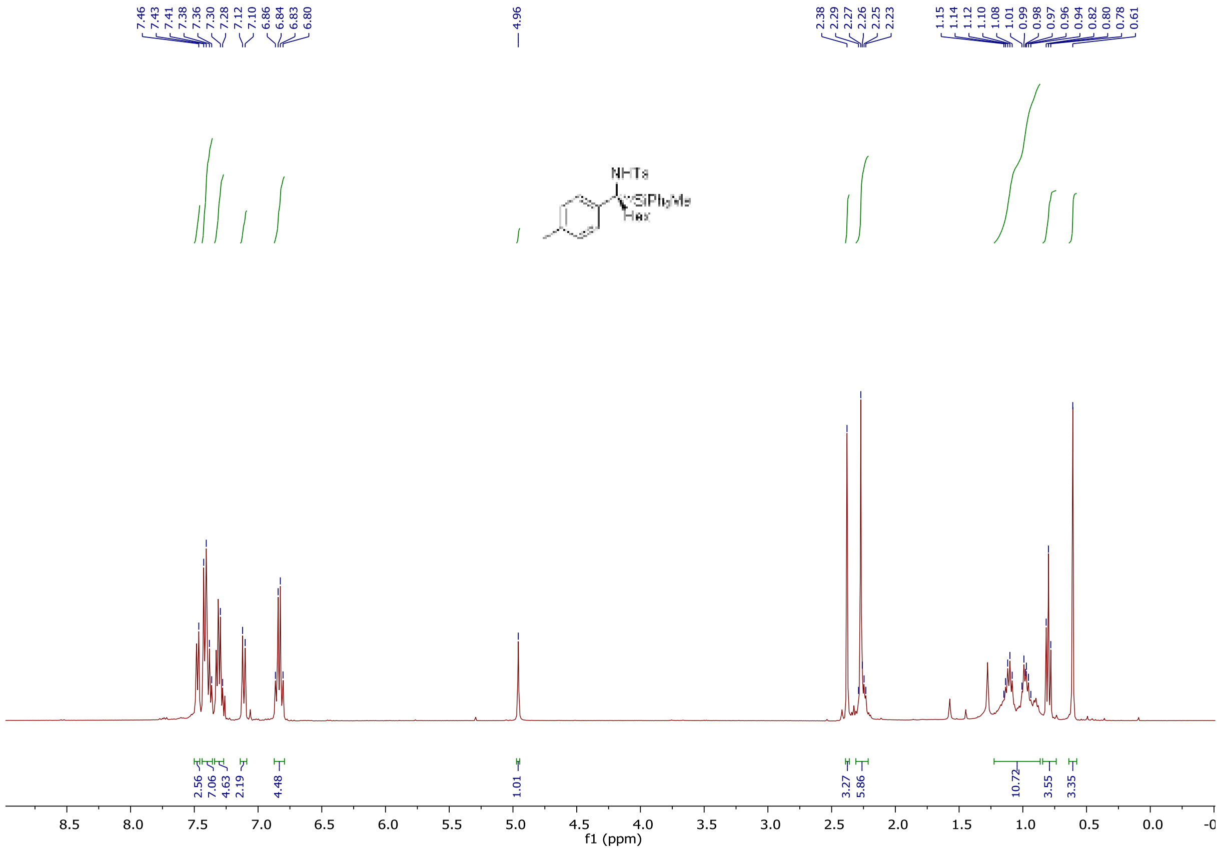

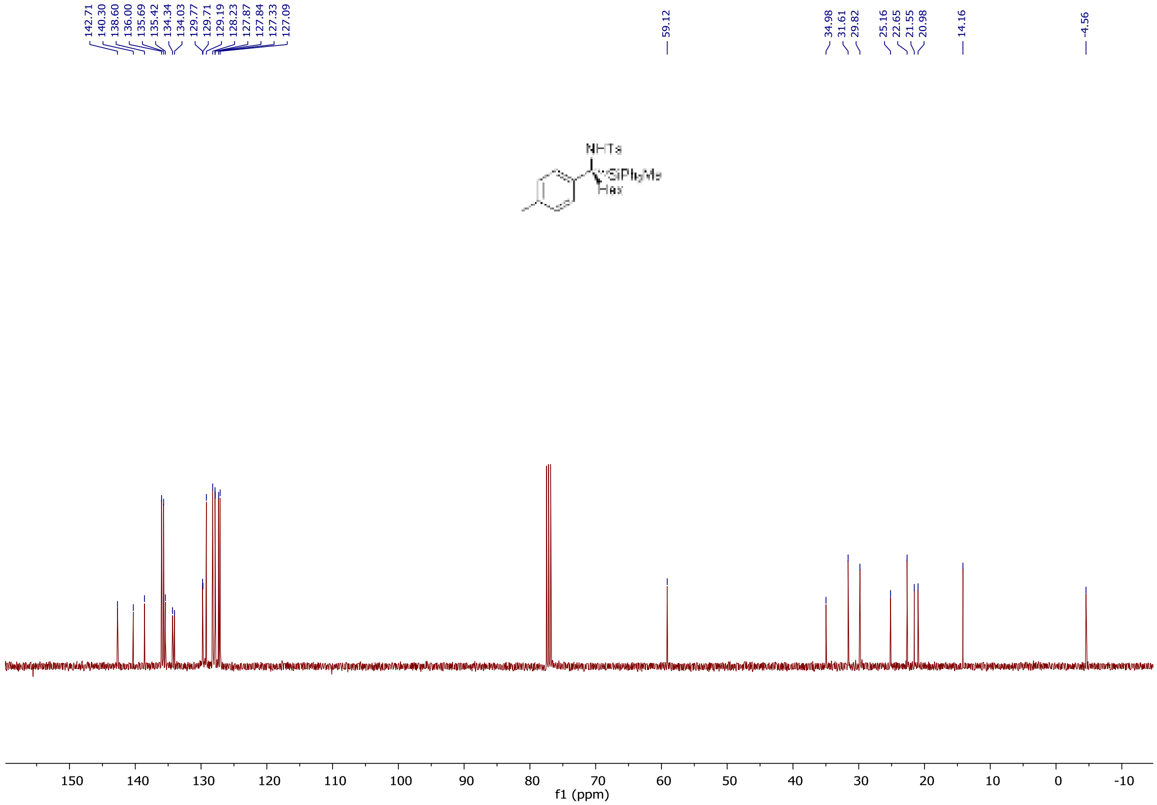

Supplementary Figure 66. 1H, 13C-NMR spectra of product **6l**.

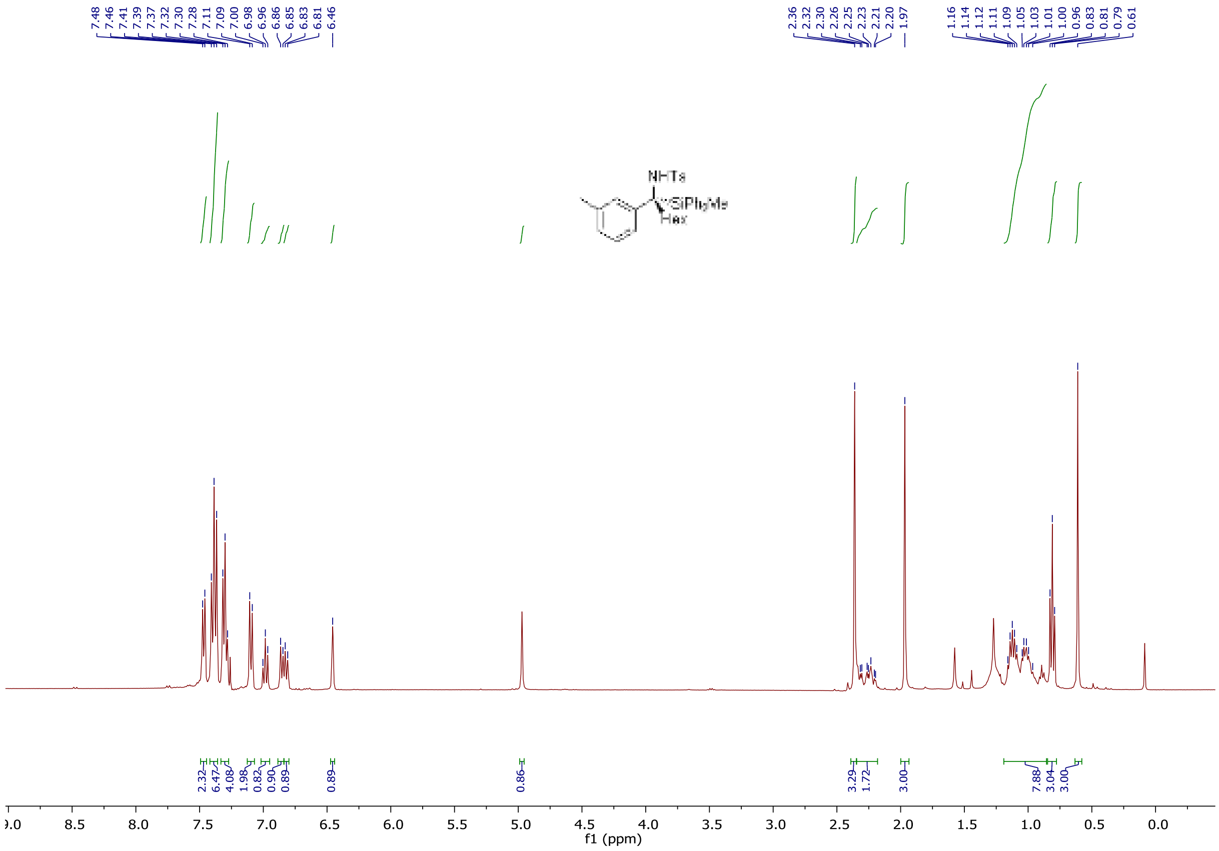

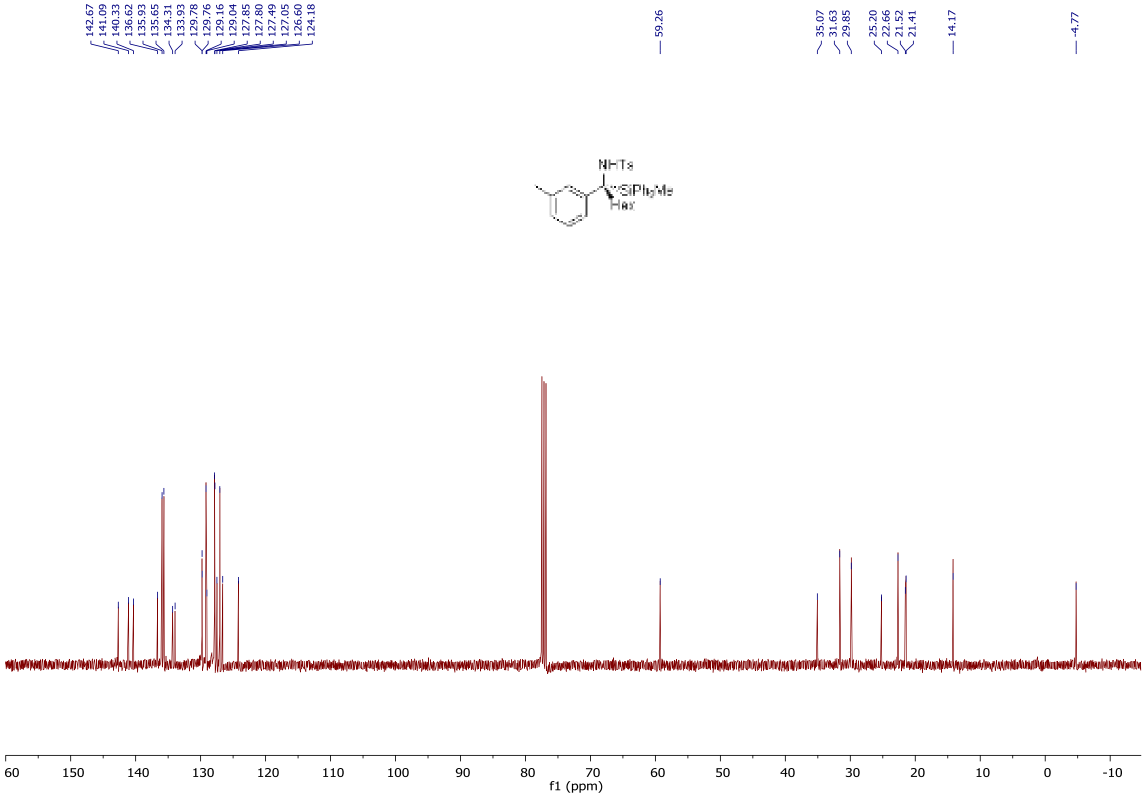

Supplementary Figure 67. 1H, 13C-NMR spectra of product **6m**.

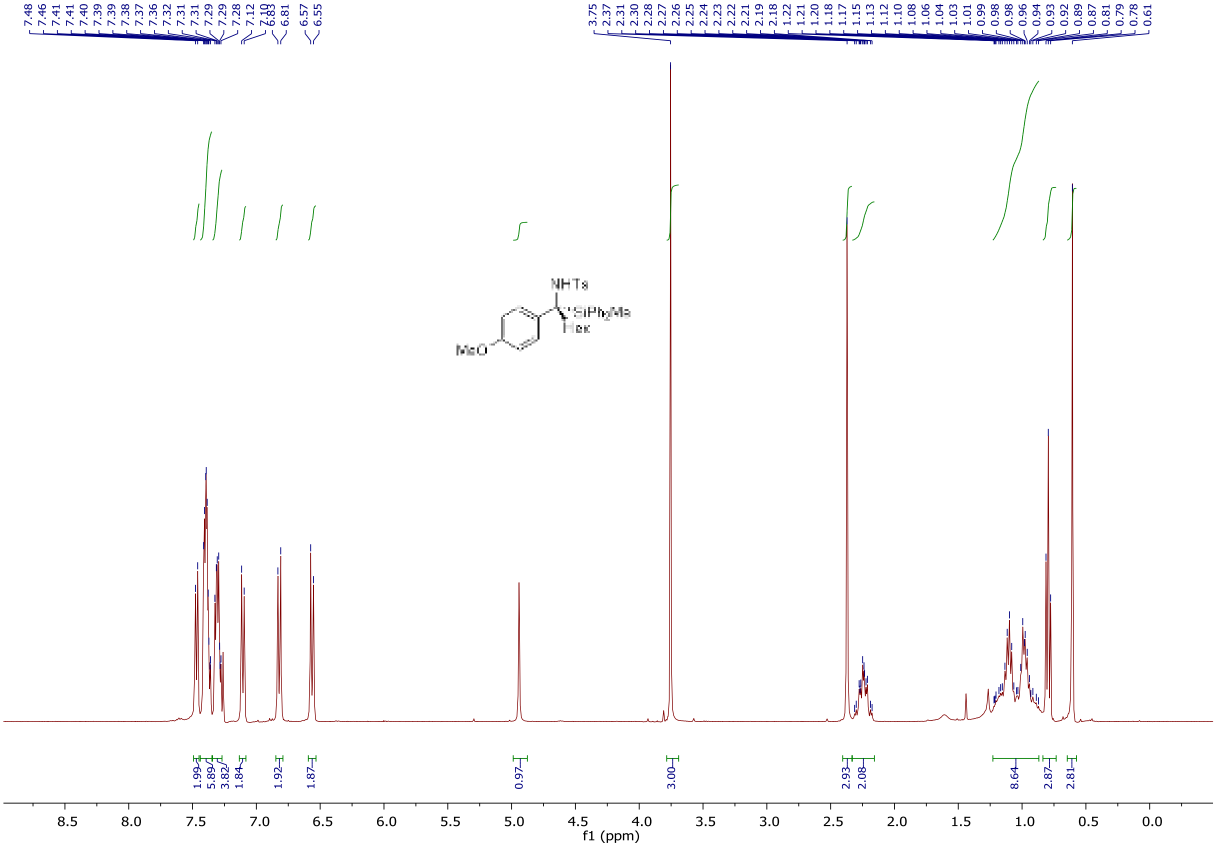

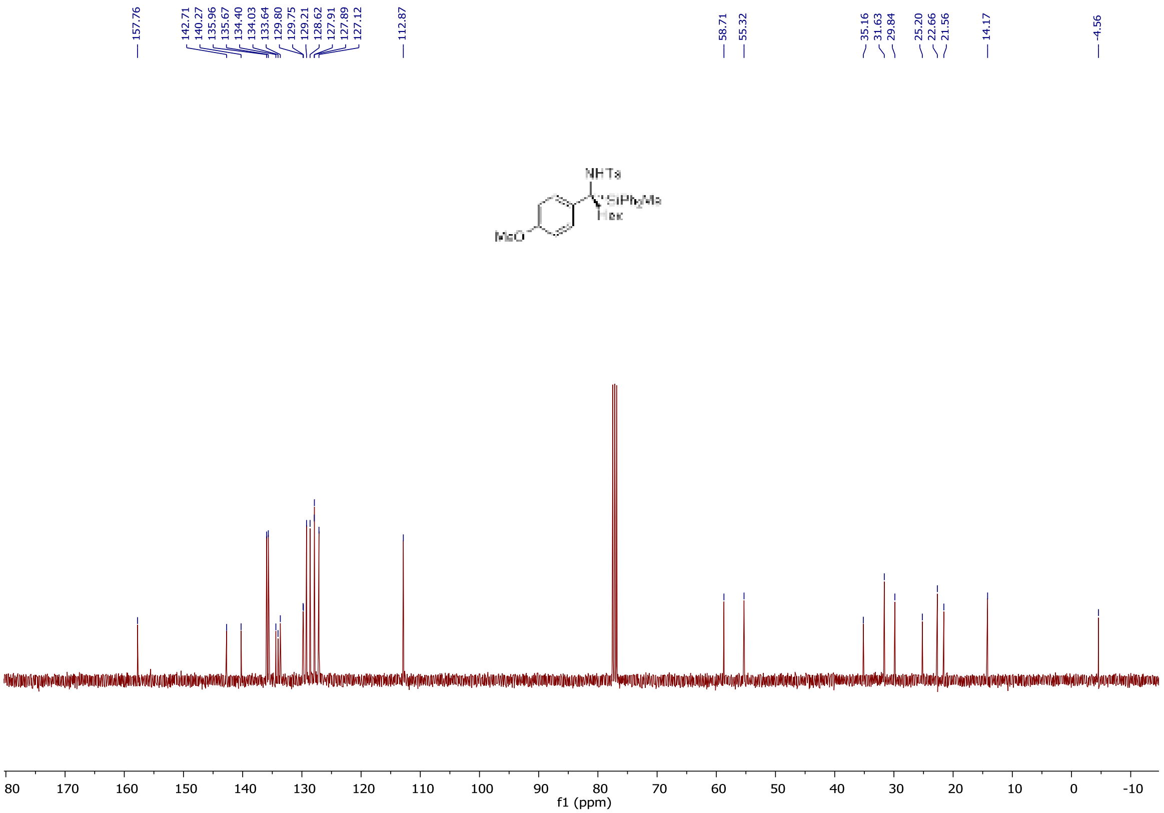

Supplementary Figure 68. 1H, 13C-NMR spectra of product **6n**.

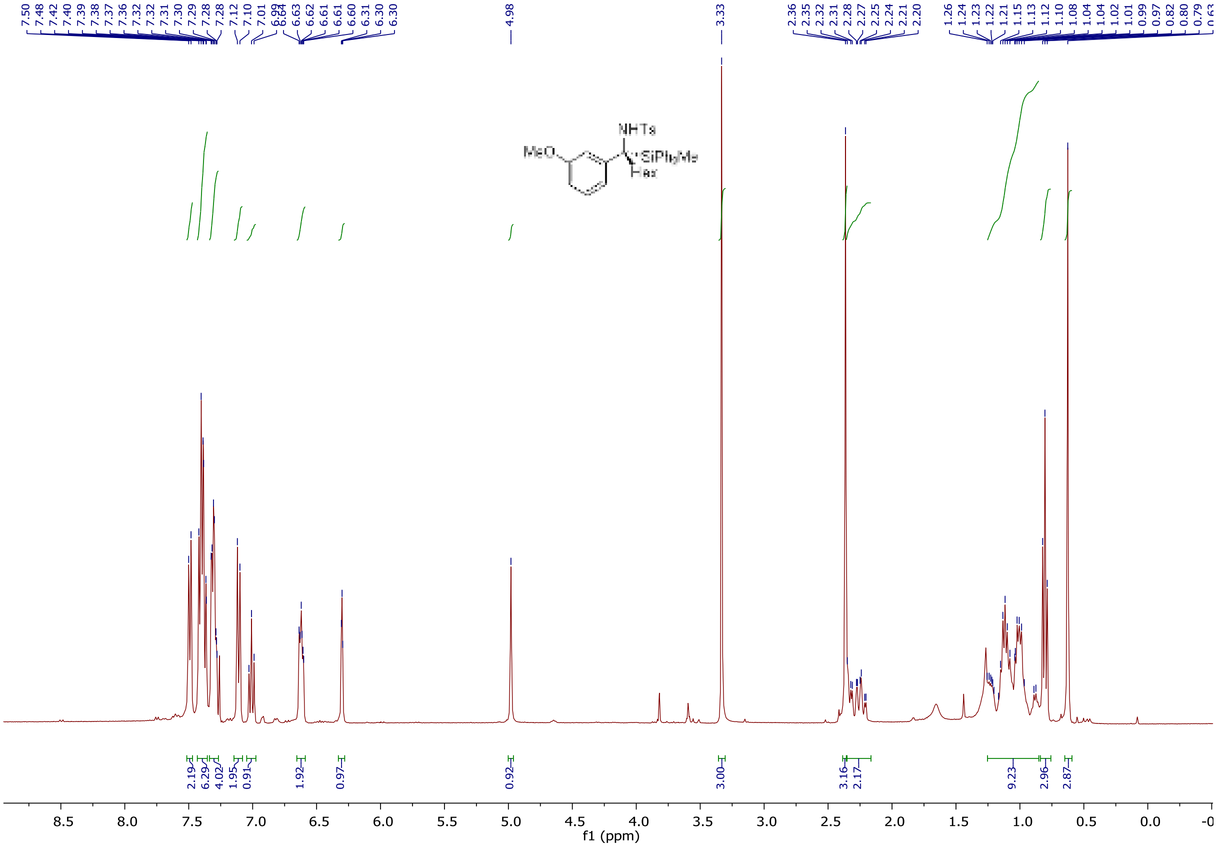

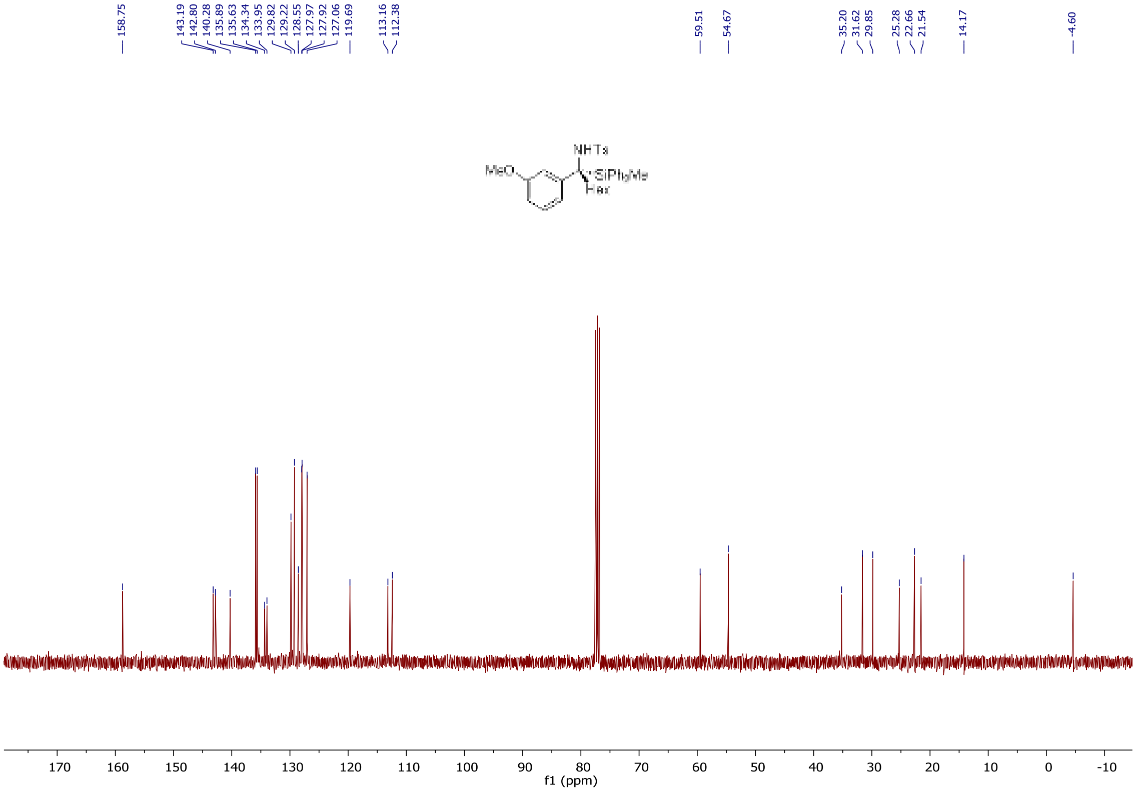

Supplementary Figure 69. 1H, 13C-NMR spectra of product **6o**.

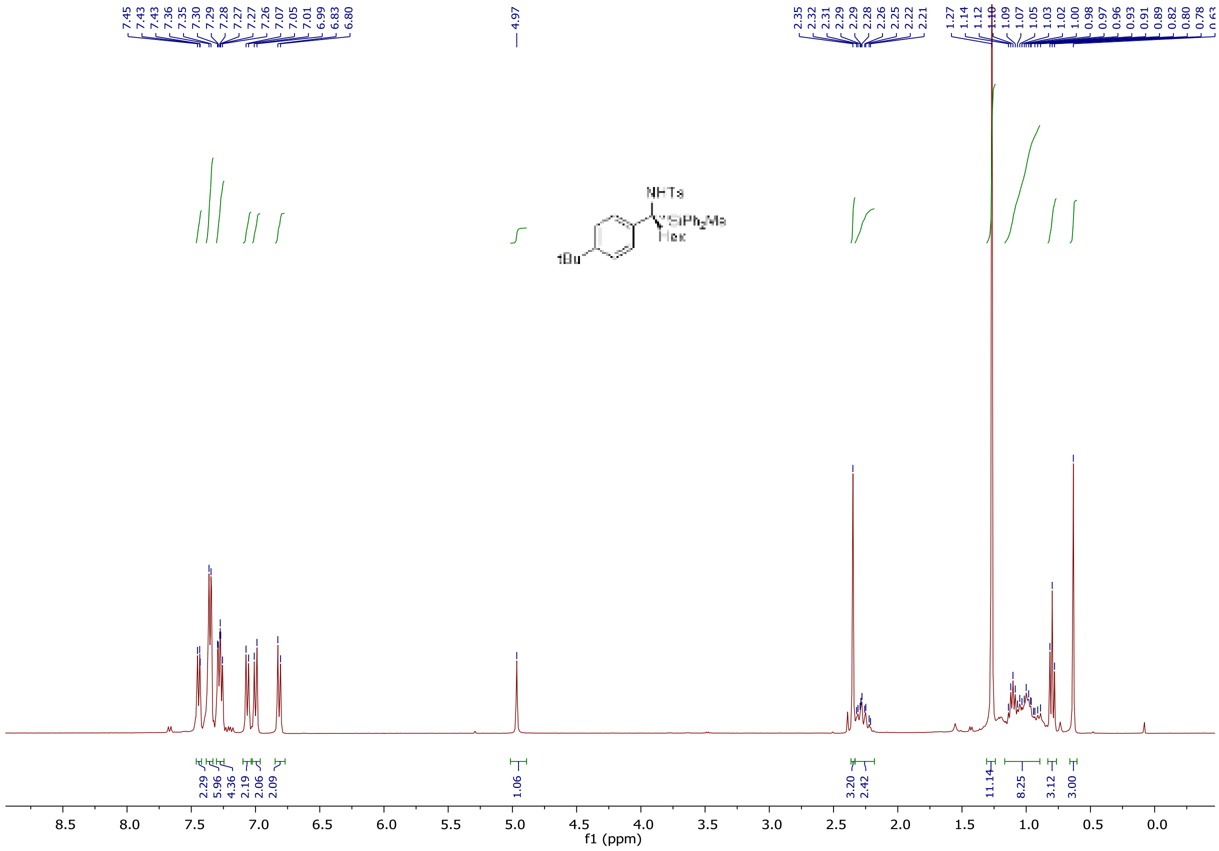

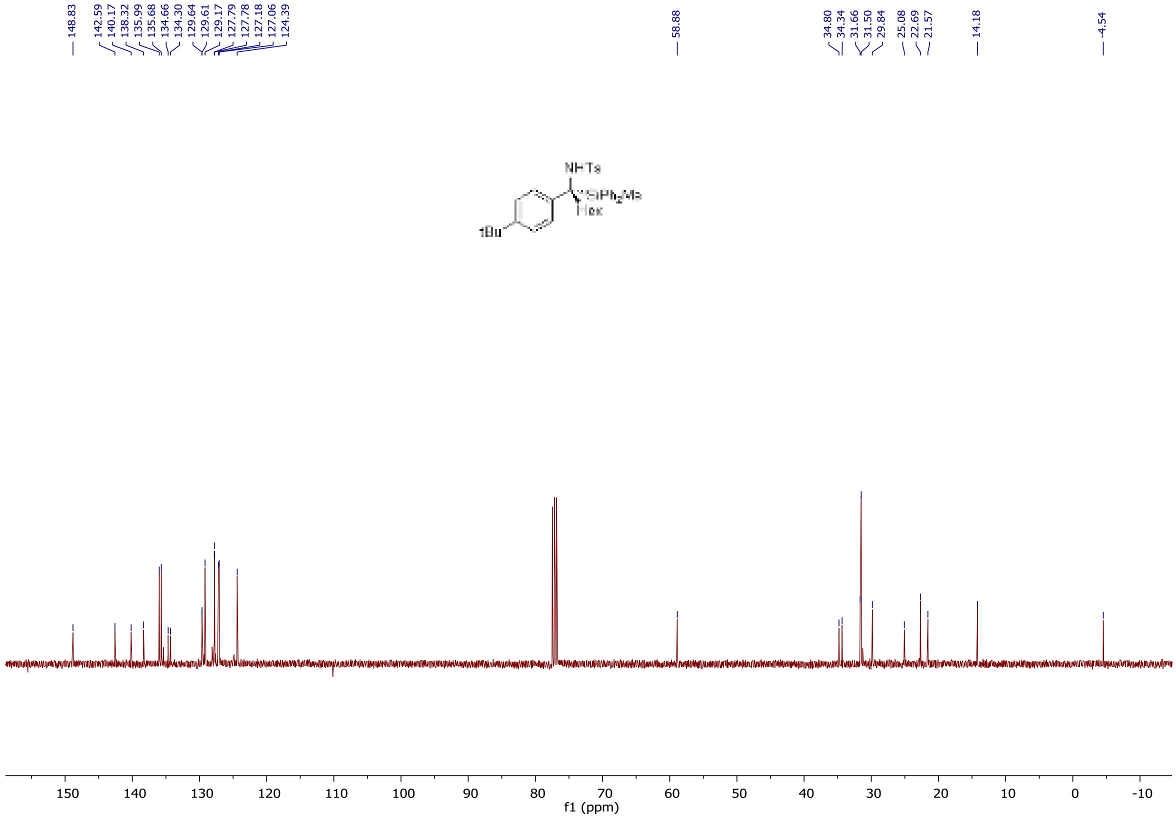

Supplementary Figure 70. 1H, 13C, 19F-NMR spectra of product **6p**.

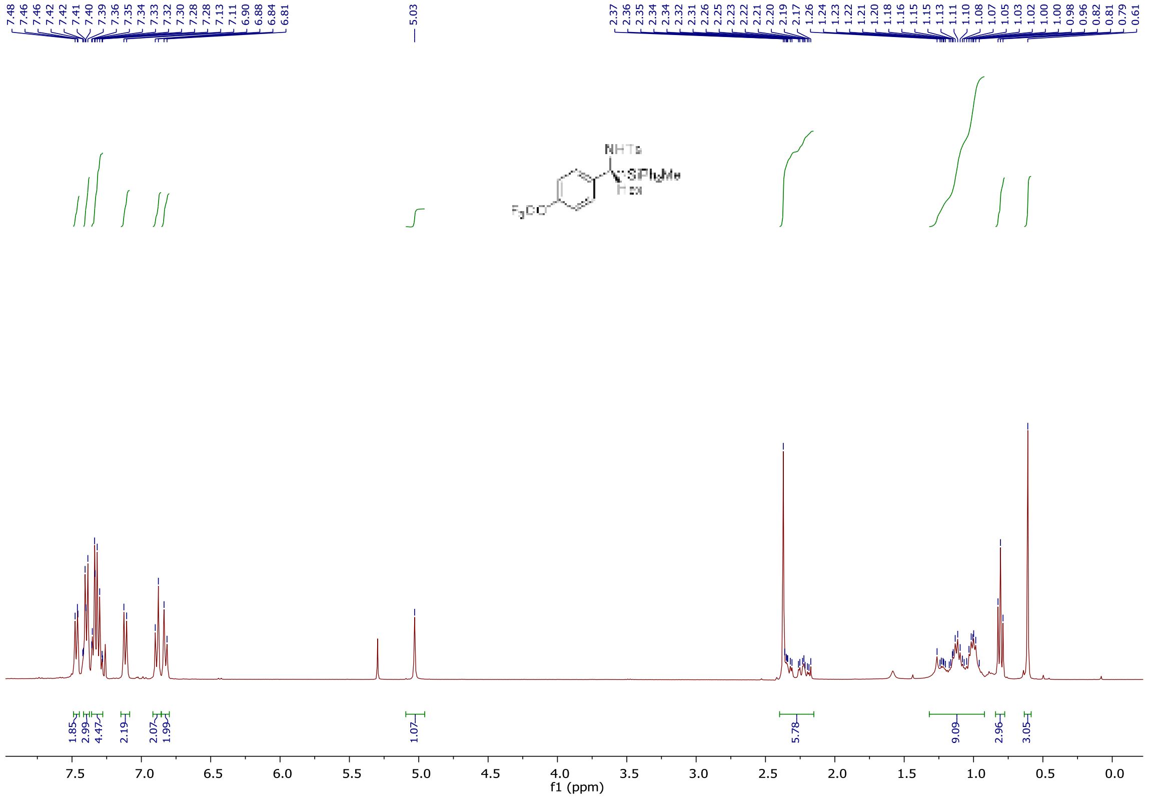

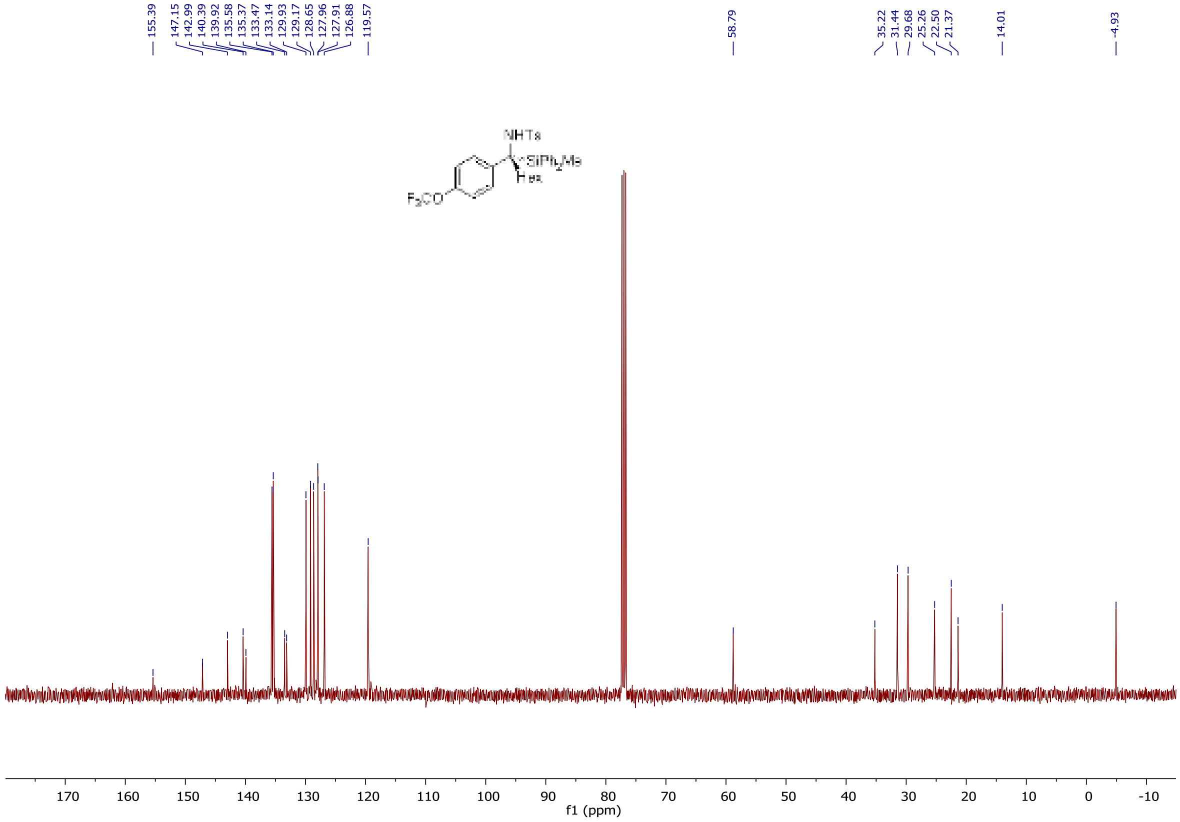

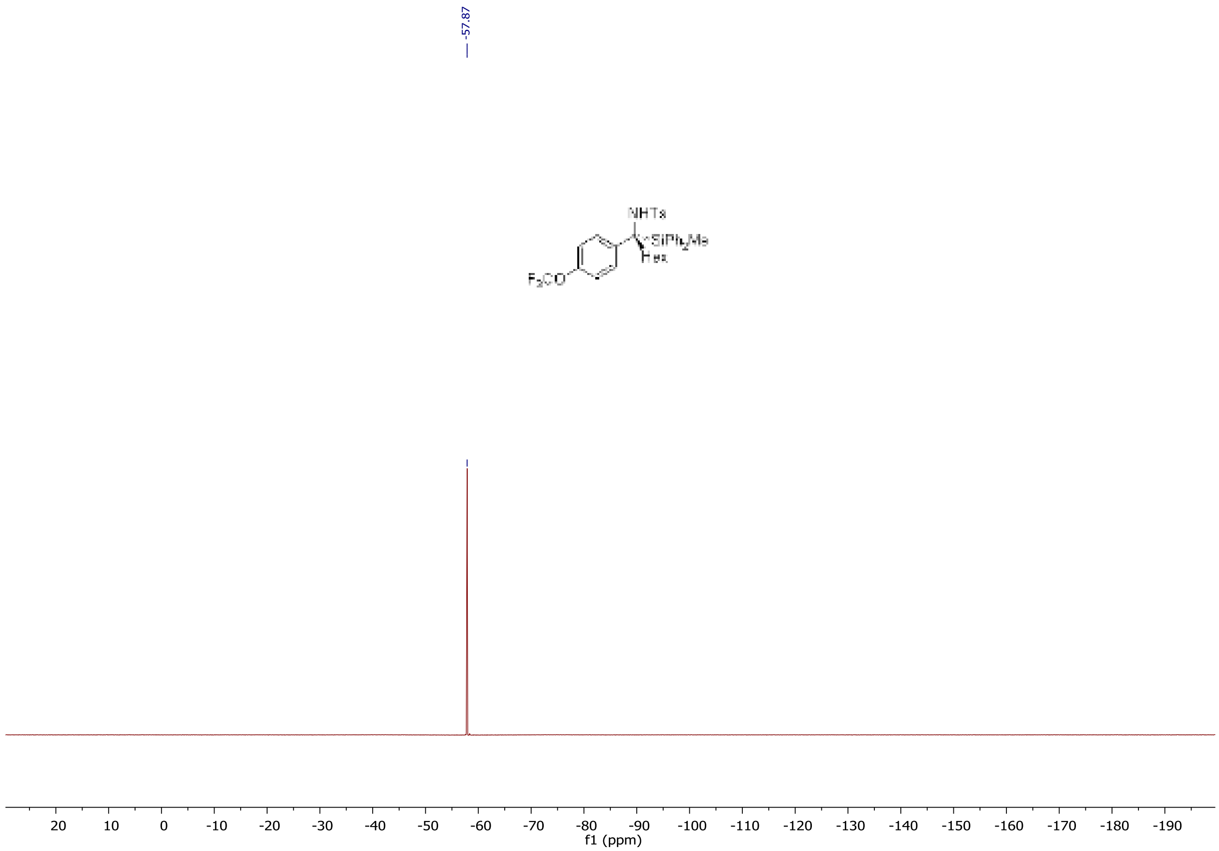
-57.87
NHTs
SiPh2Me
Hex
F3CO
20 10 0 -10 -20 -30 -40 -50 -60 -70 -80 -90 -100 -110 -120 -130 -140 -150 -160 -170 -180 -190
f1 (ppm)

Supplementary Figure 71. 1H, 13C, 19F-NMR spectra of product **6q**.

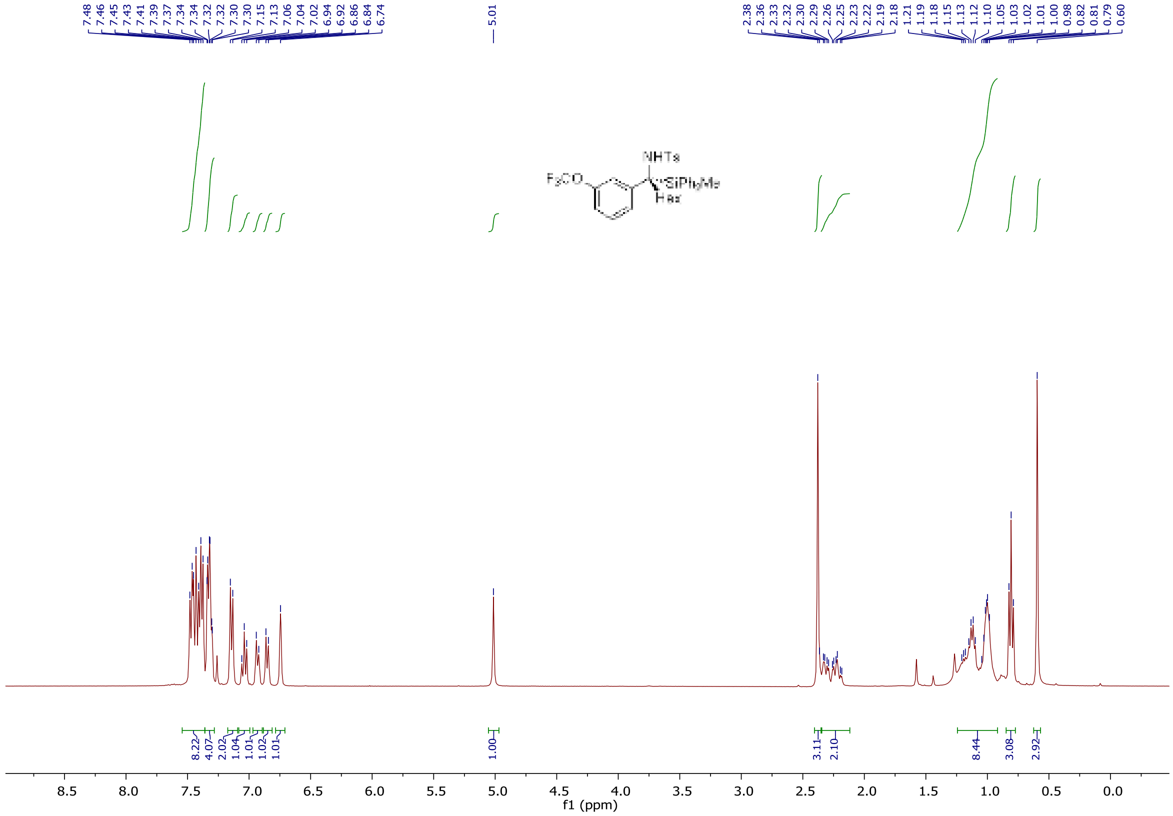

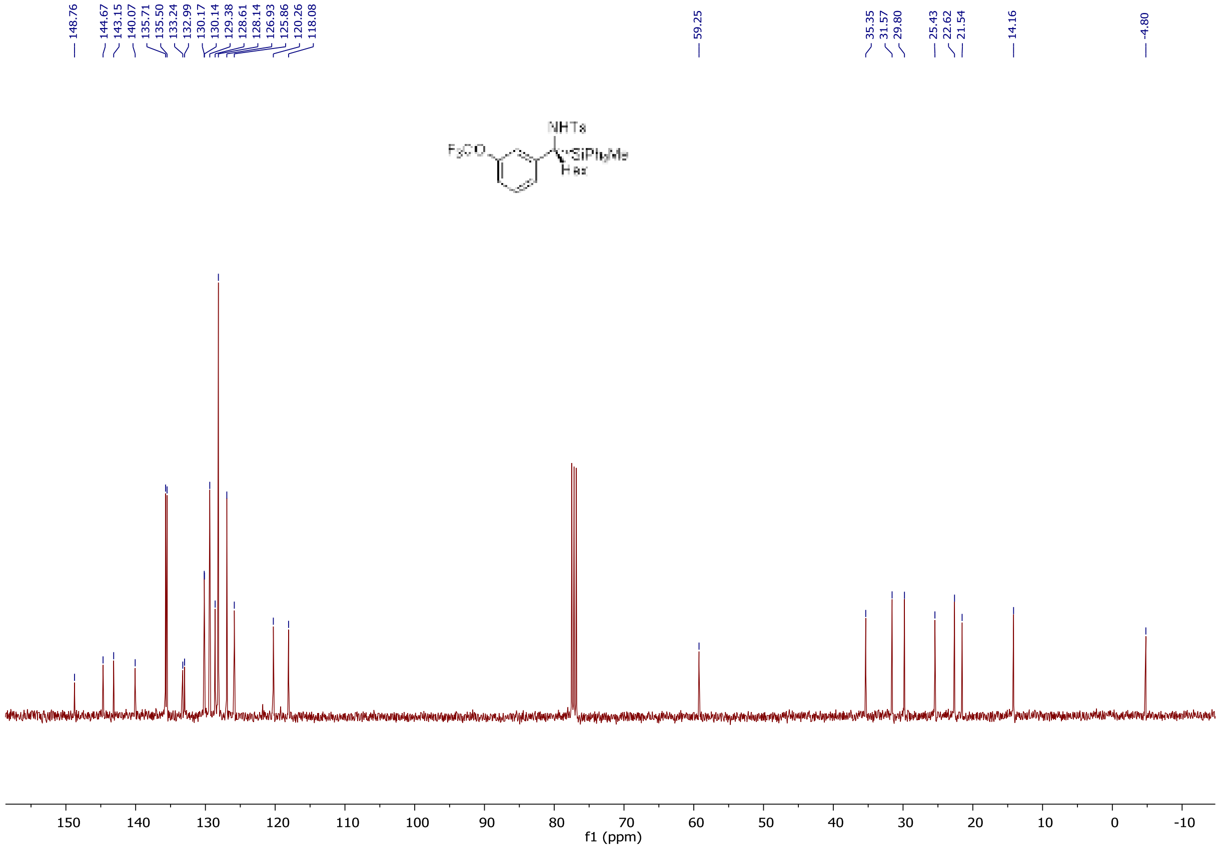

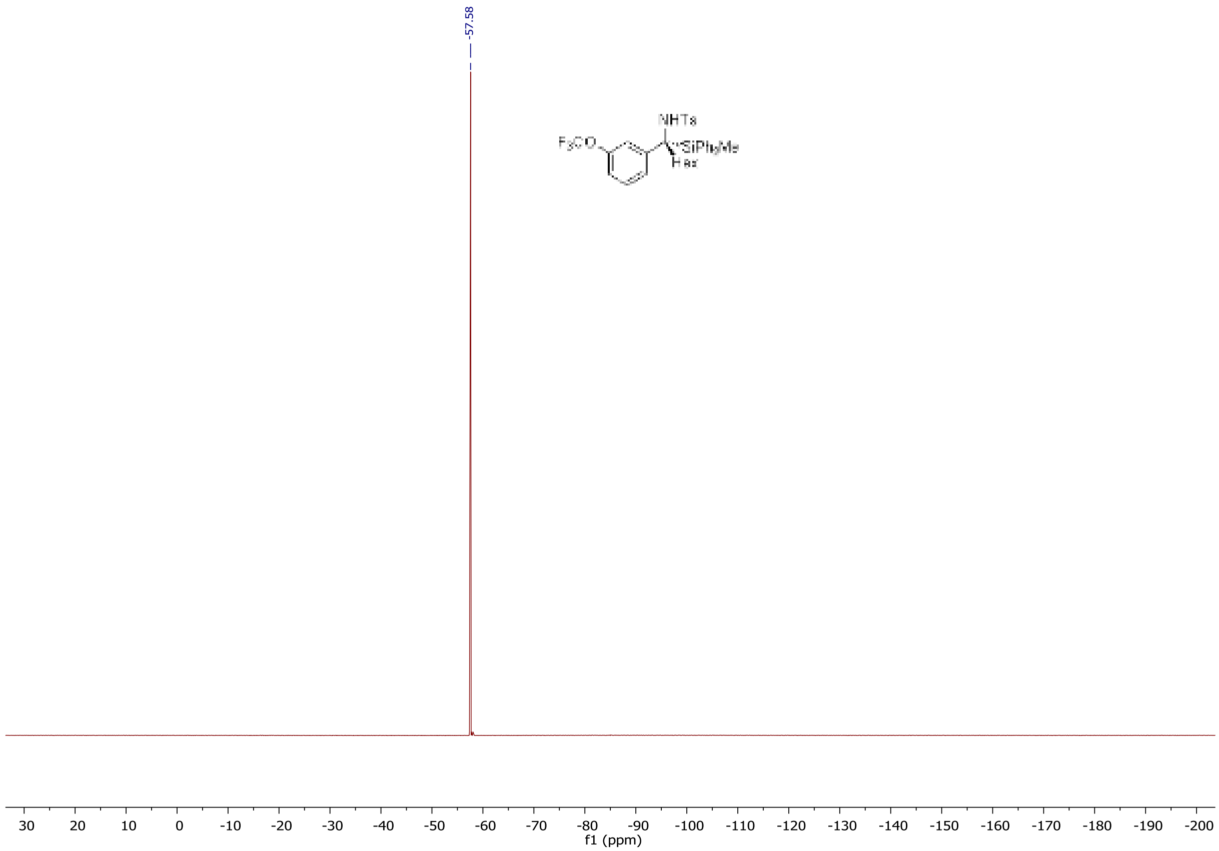

-57.58
NHTs
F3CO
SiPh2Me
H
30
20
10
0
-10
-20
-30
-40
-50
-60
-70
-80
-90
-100
-110
-120
-130
-140
-150
-160
-170
-180
-190
-200
f1 (ppm)

Supplementary Figure 72. 1H, 13C-NMR spectra of product **6r**.

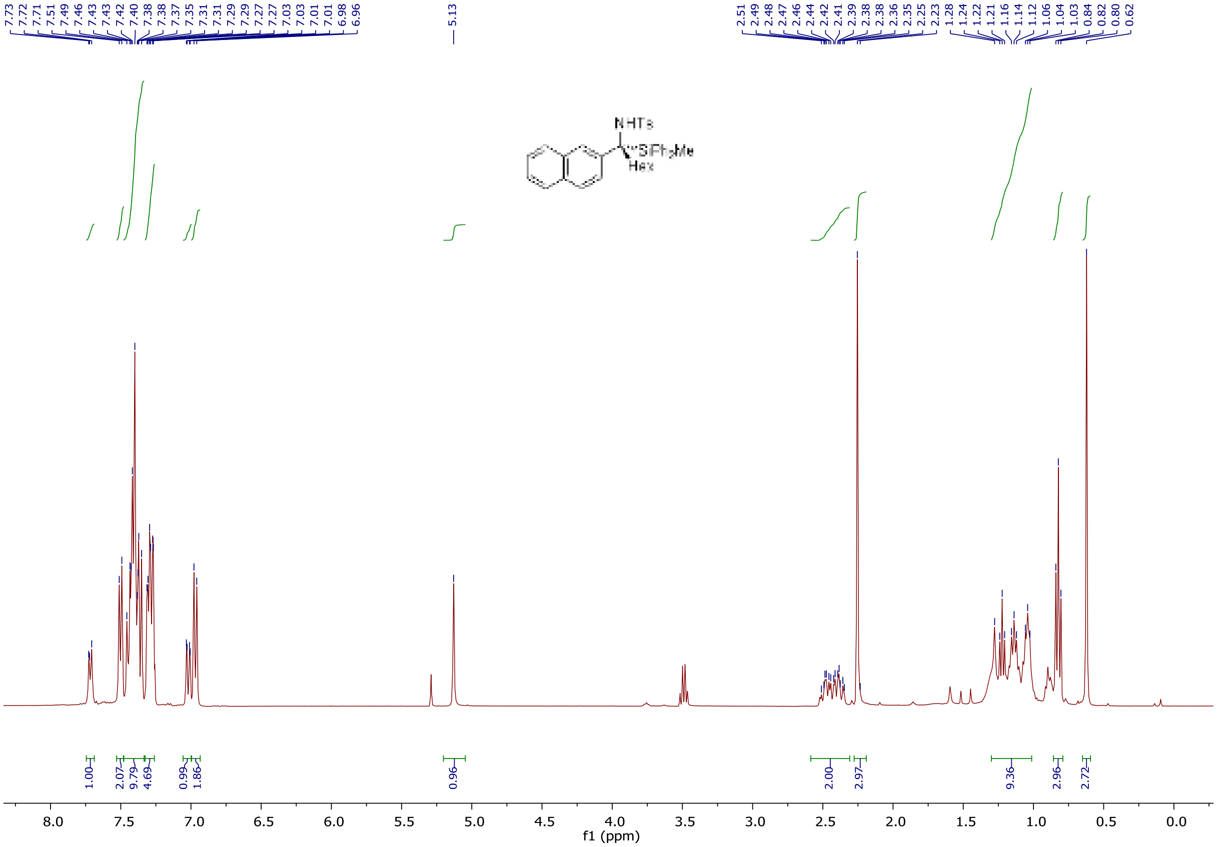

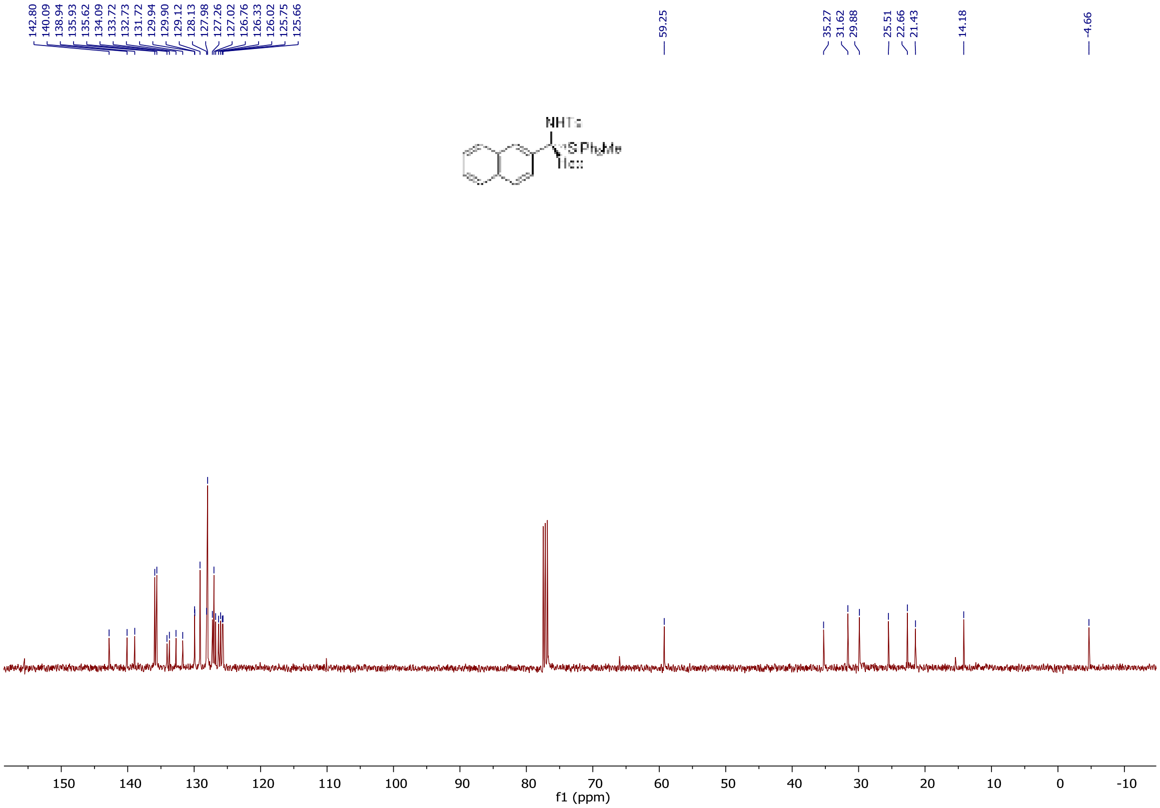

Supplementary Figure 73. 1H, 13C-NMR spectra of product **6s**.

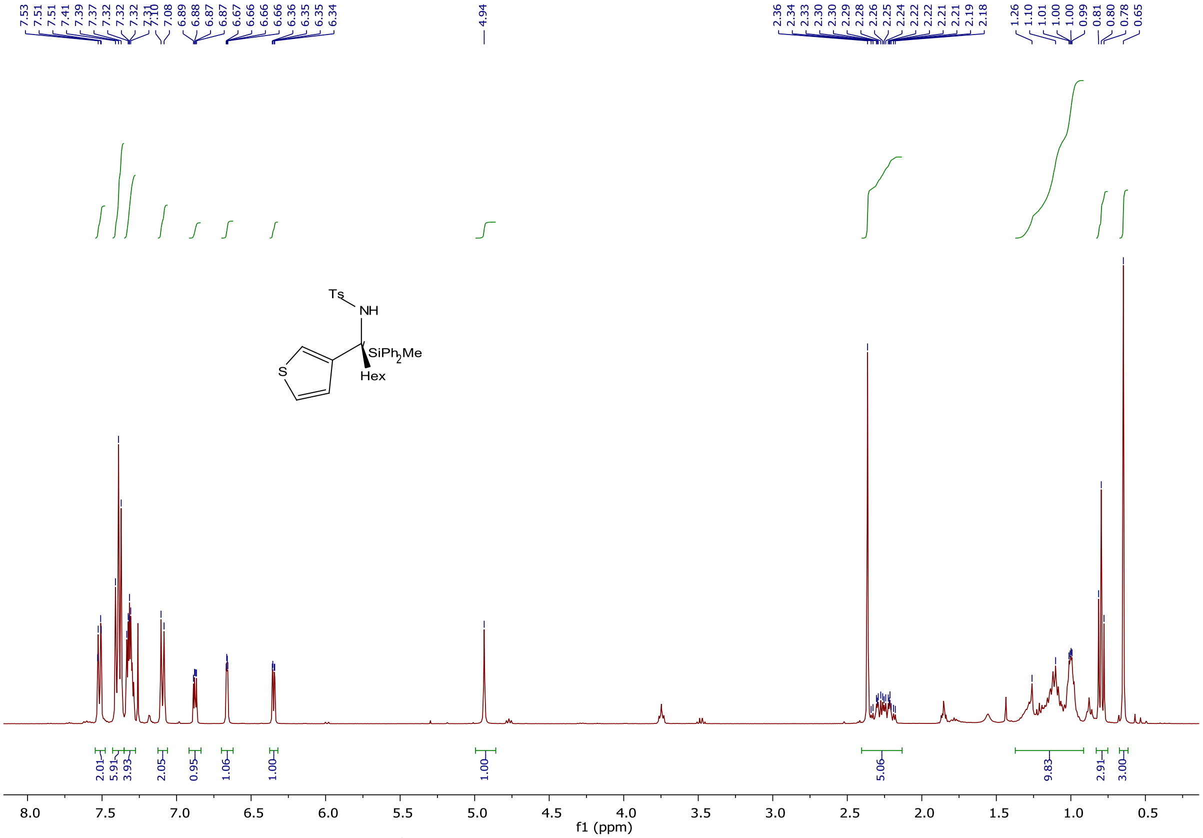

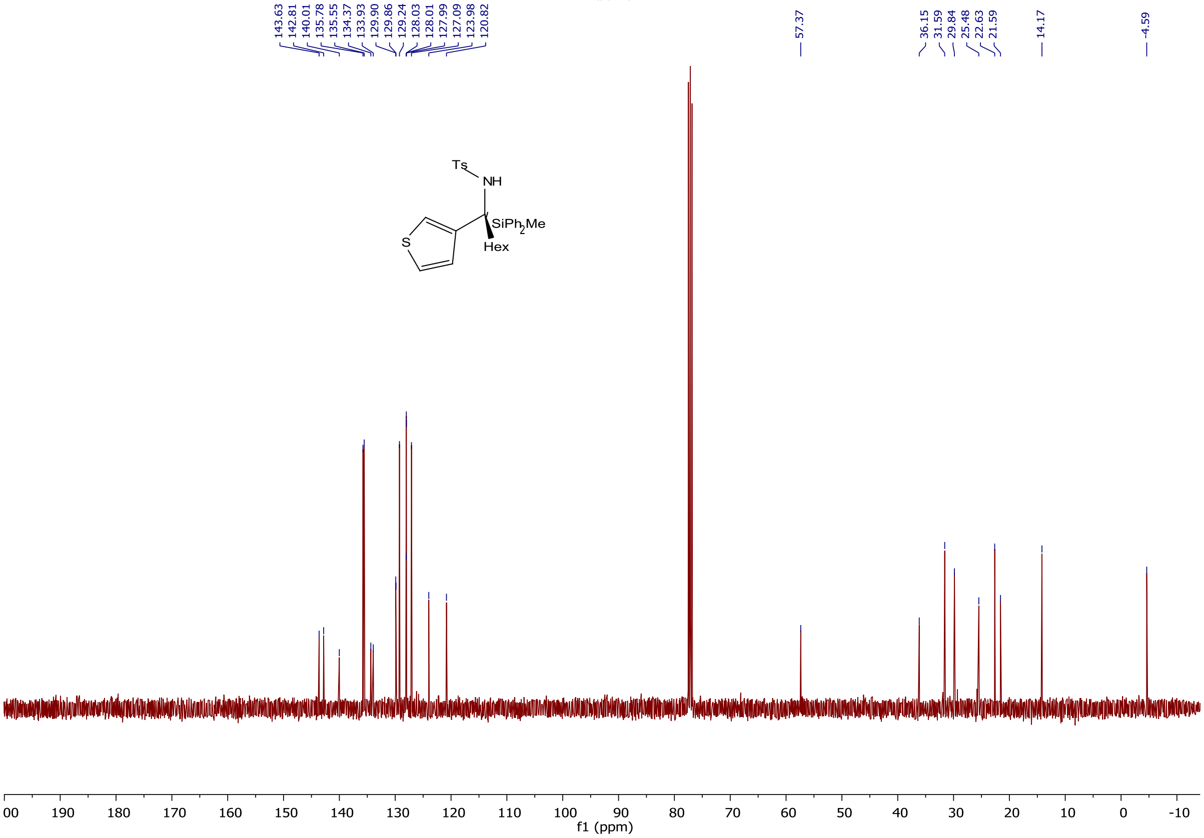

Supplementary Figure 74. 1H, 13C-NMR spectra of product **6t**.

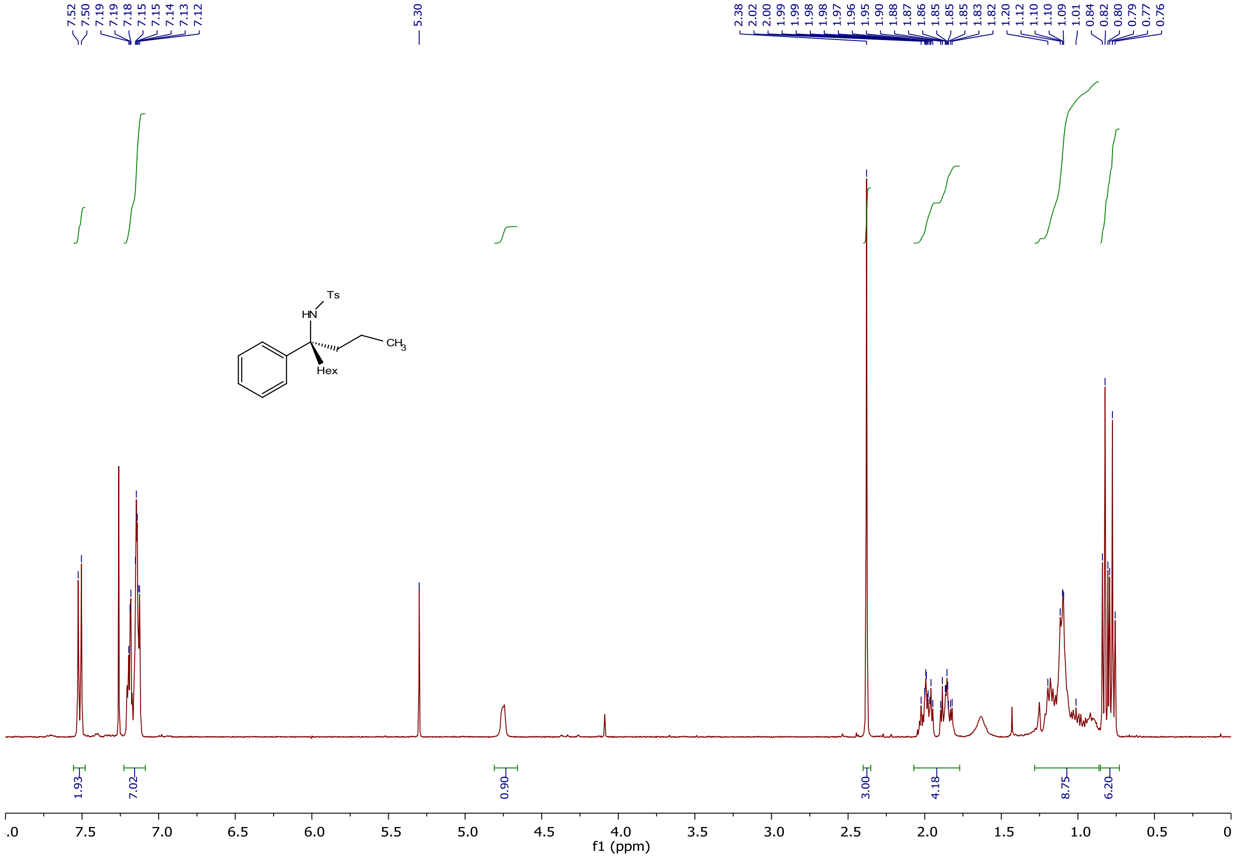

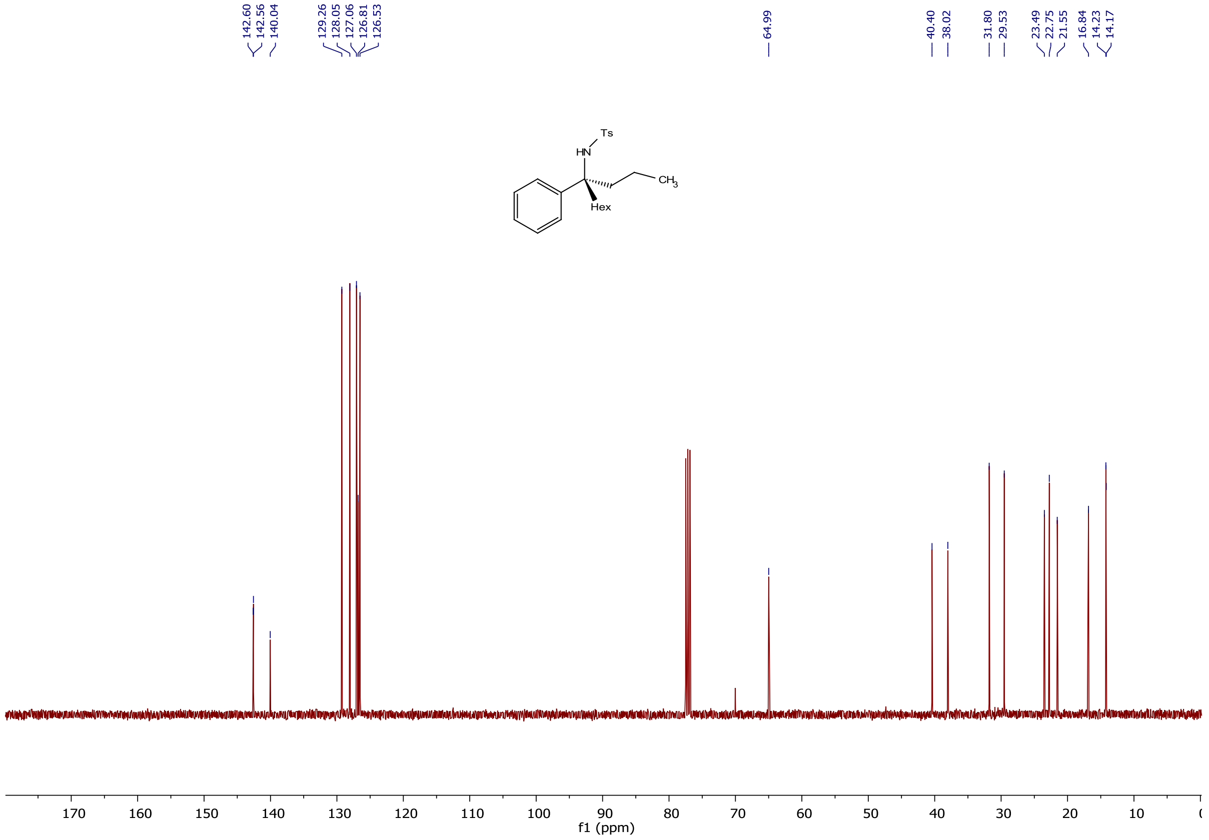

Supplementary Figure 75. 1H, 13C-NMR spectra of product **7a.**

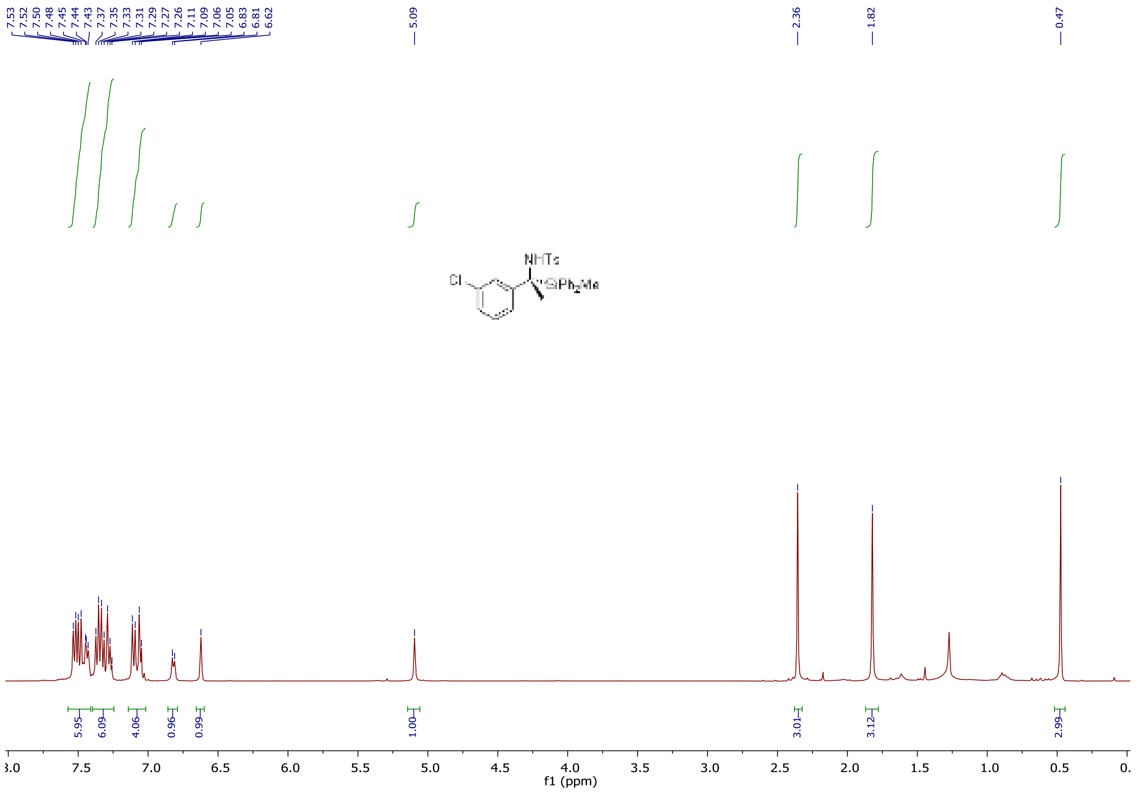

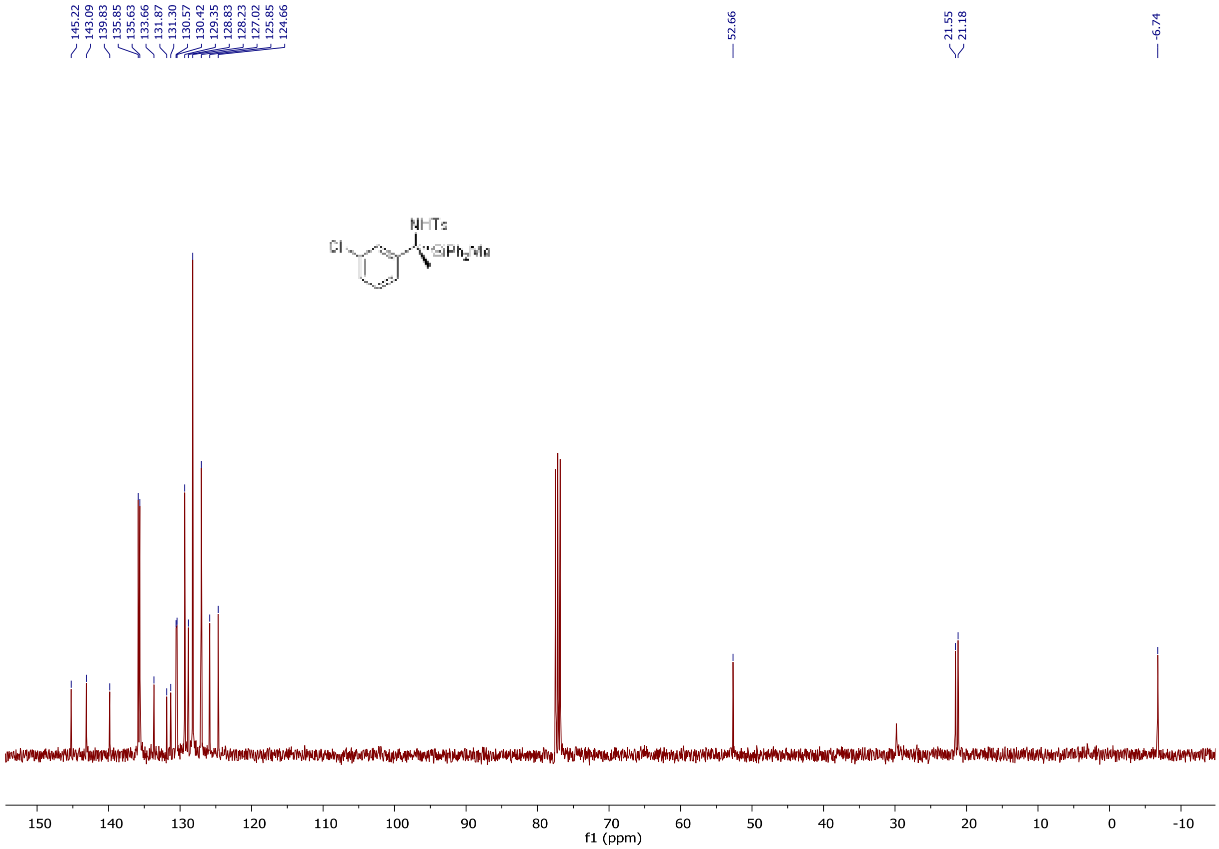

Supplementary Figure 76. 1H, 13C-NMR spectra of product **7b**.

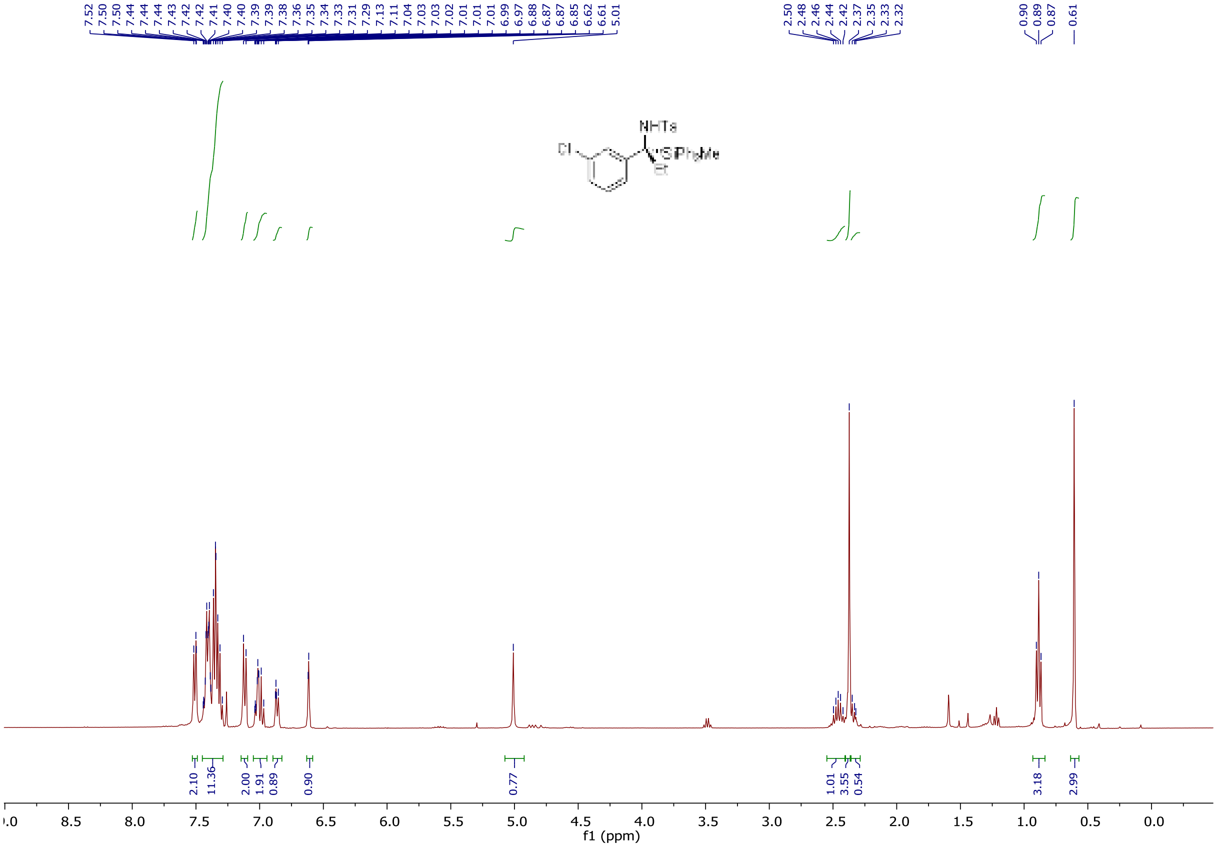

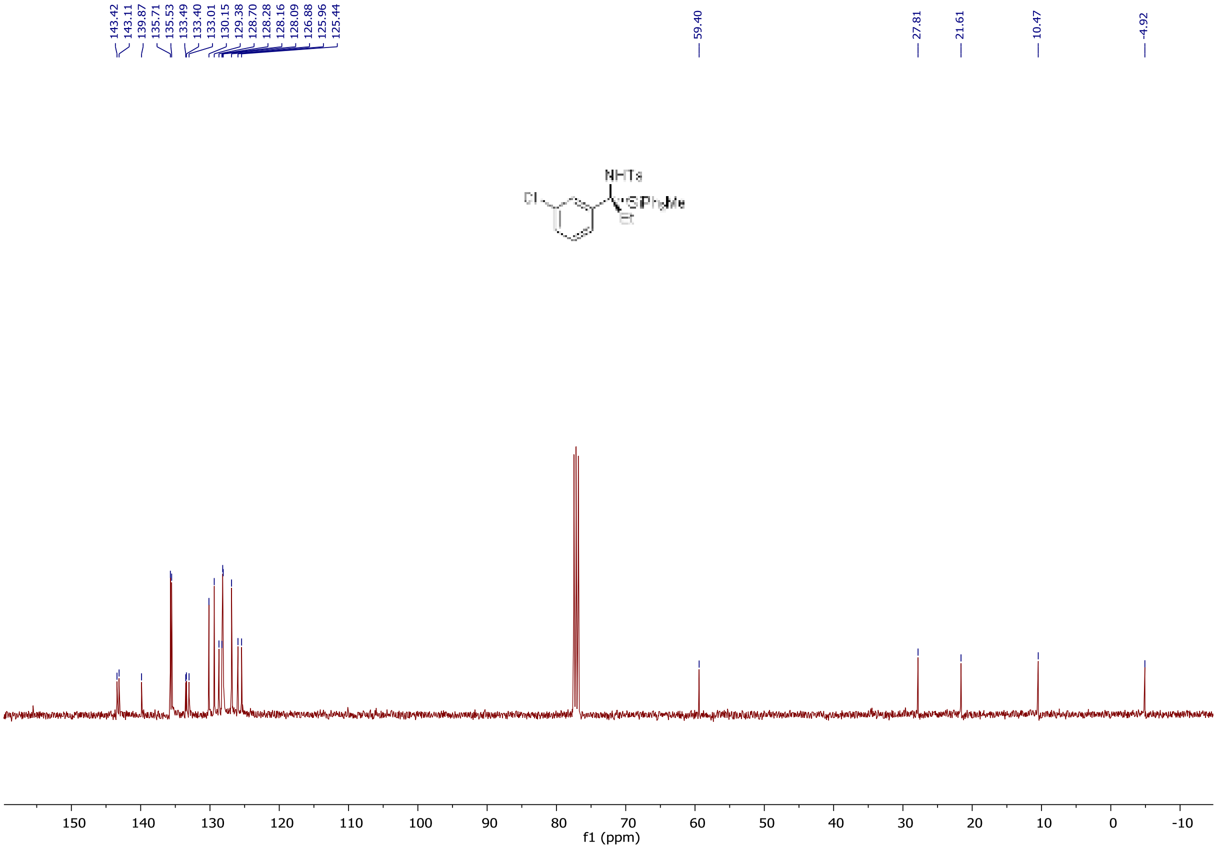

Supplementary Figure 77. 1H, 13C-NMR spectra of product **7c**.

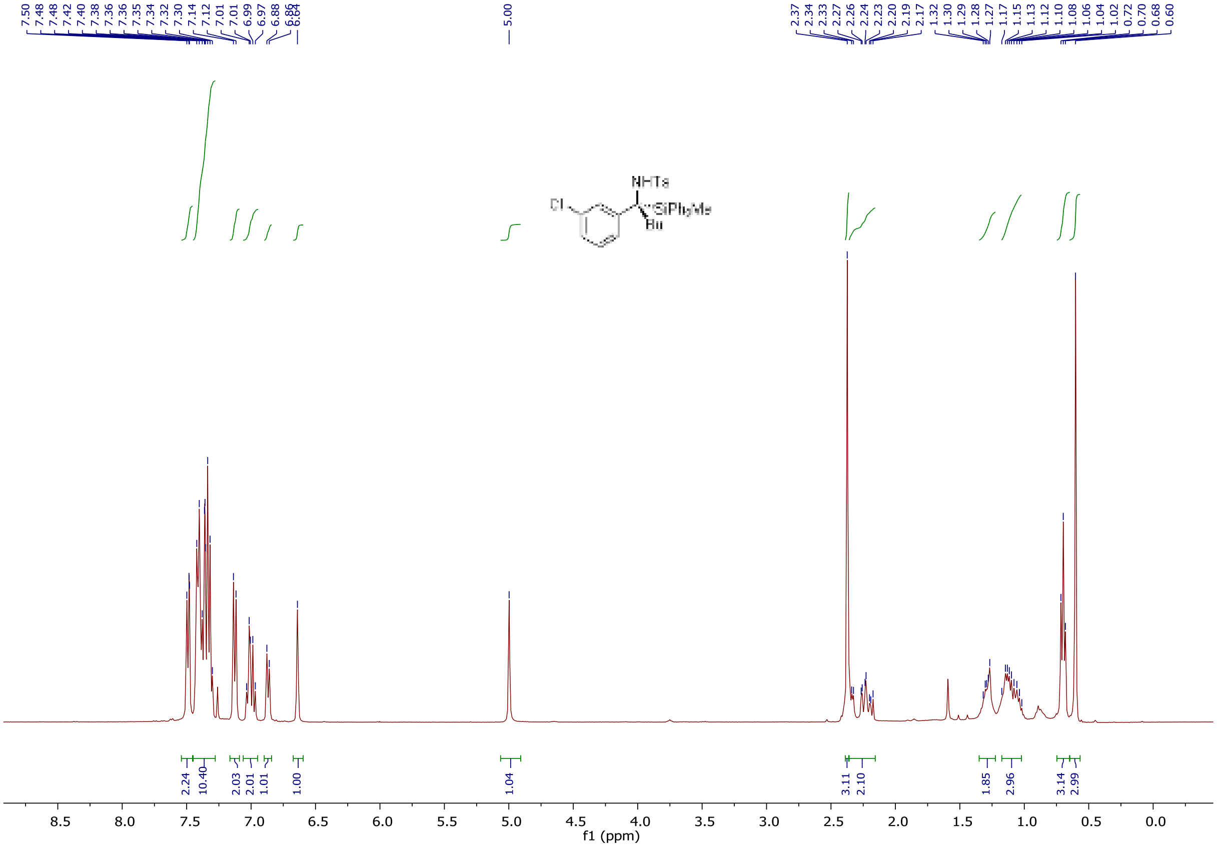

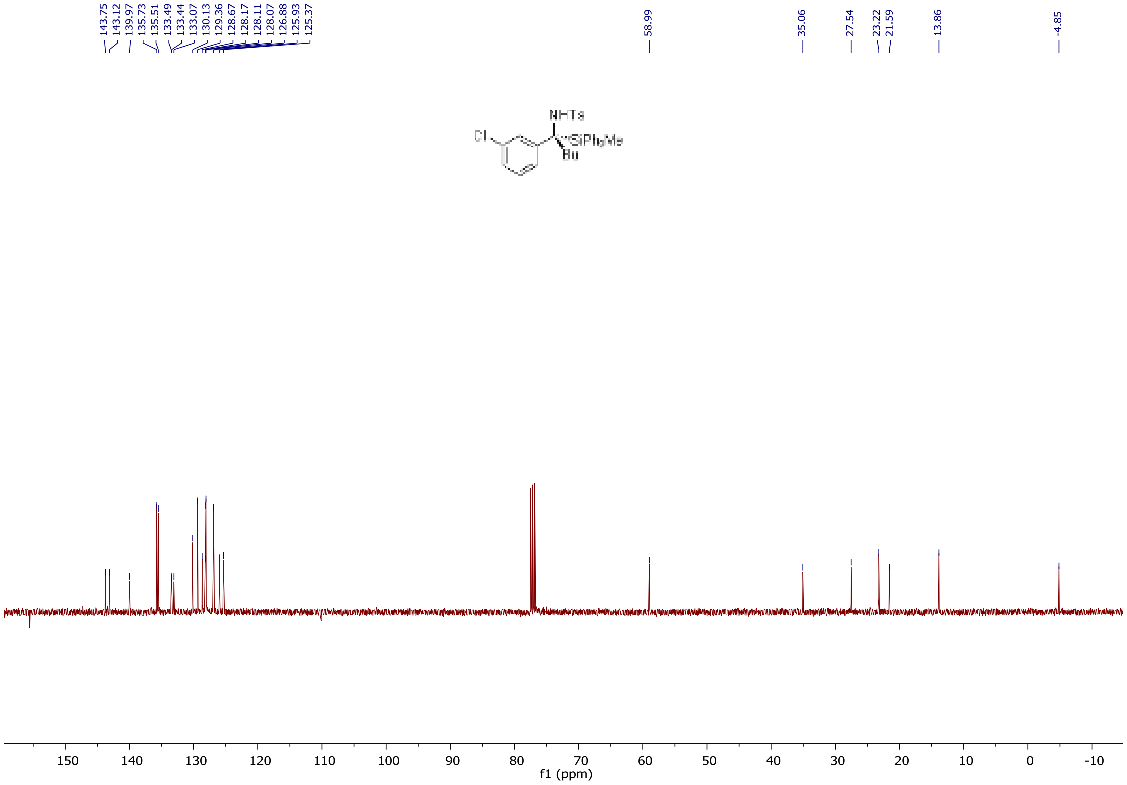

Supplementary Figure 78. 1H, 13C-NMR spectra of product **7d**.

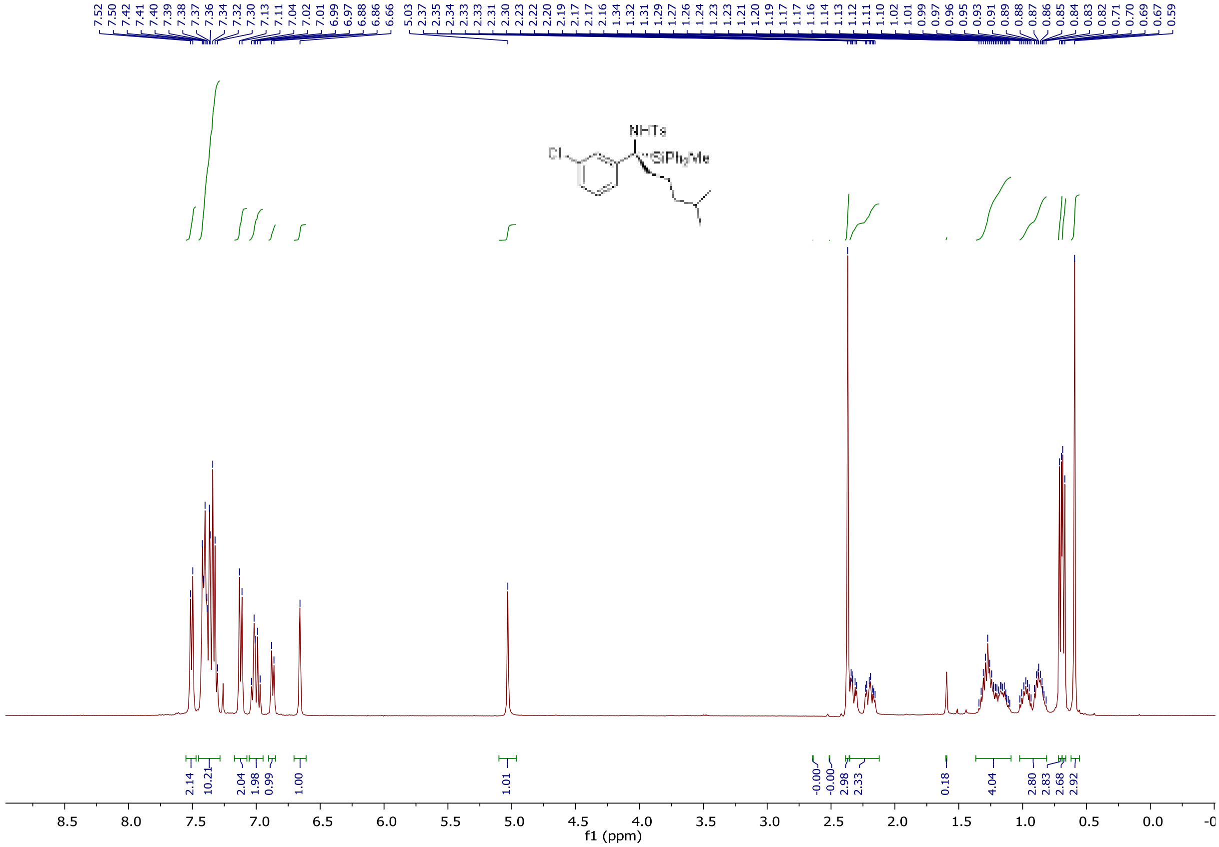

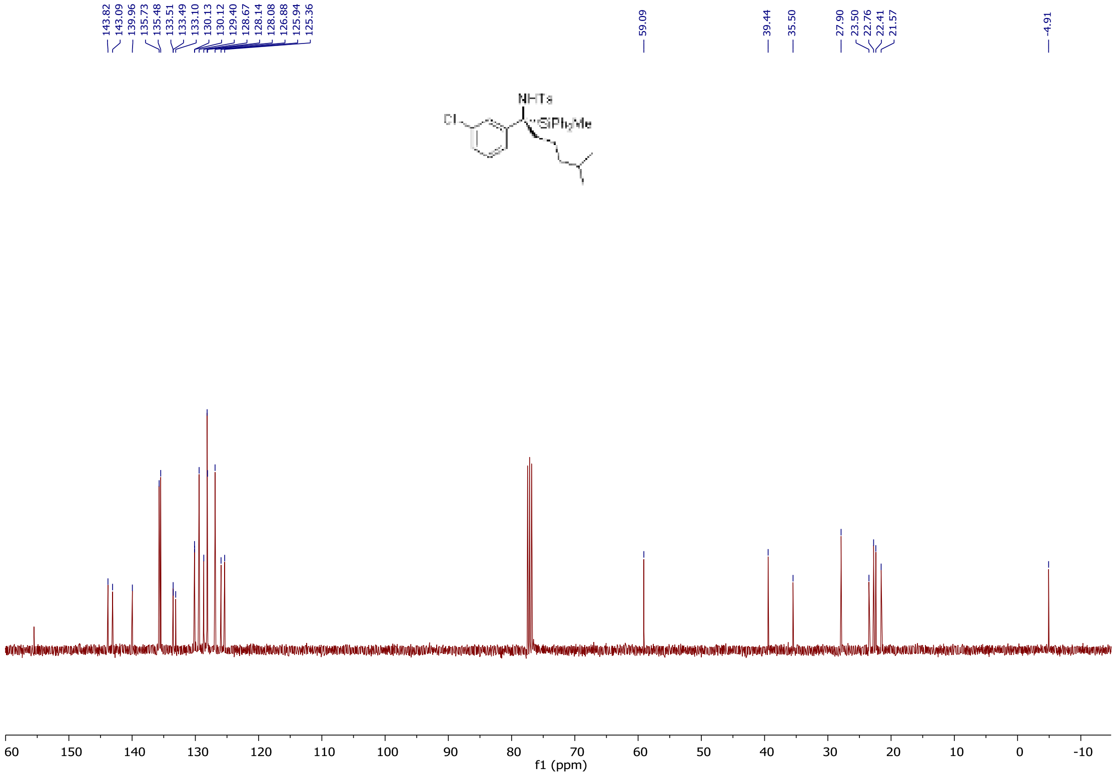

Supplementary Figure 79. 1H, 13C-NMR spectra of product **7e**.

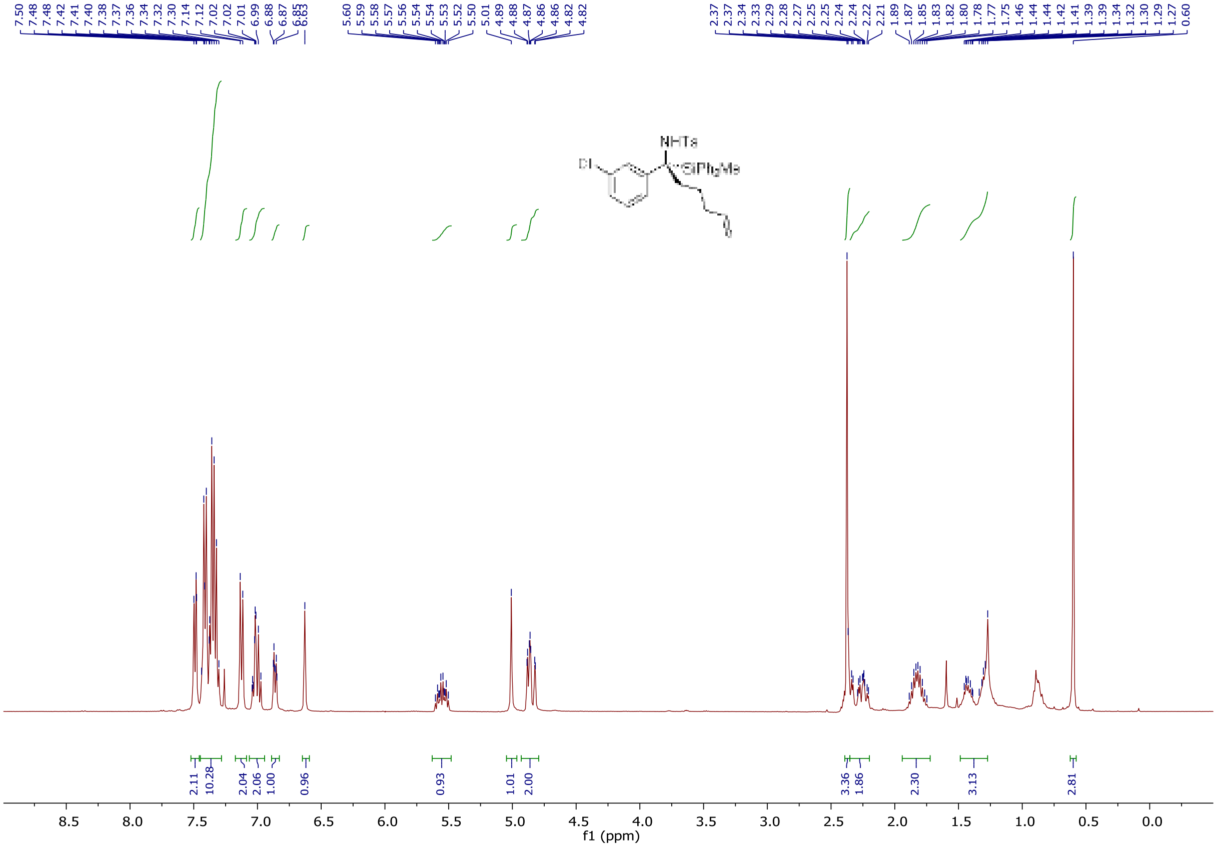

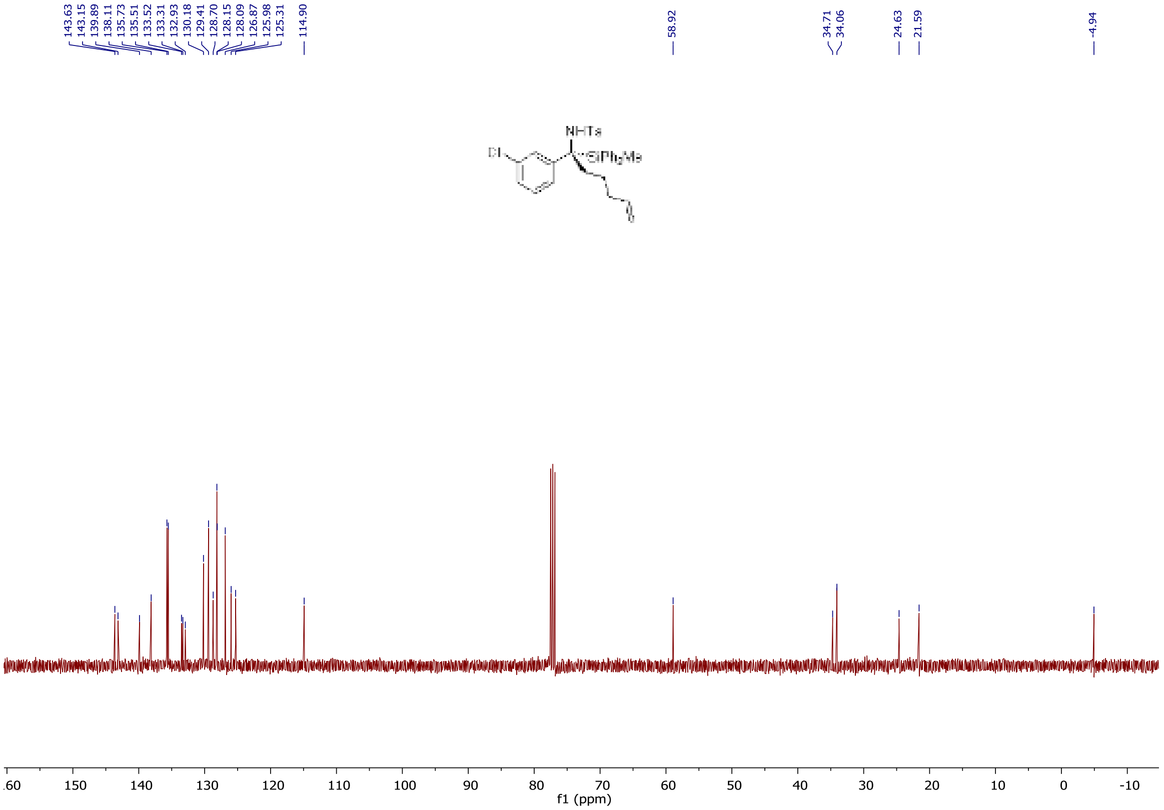

Supplementary Figure 80. 1H, 13C-NMR spectra of product **7f**.

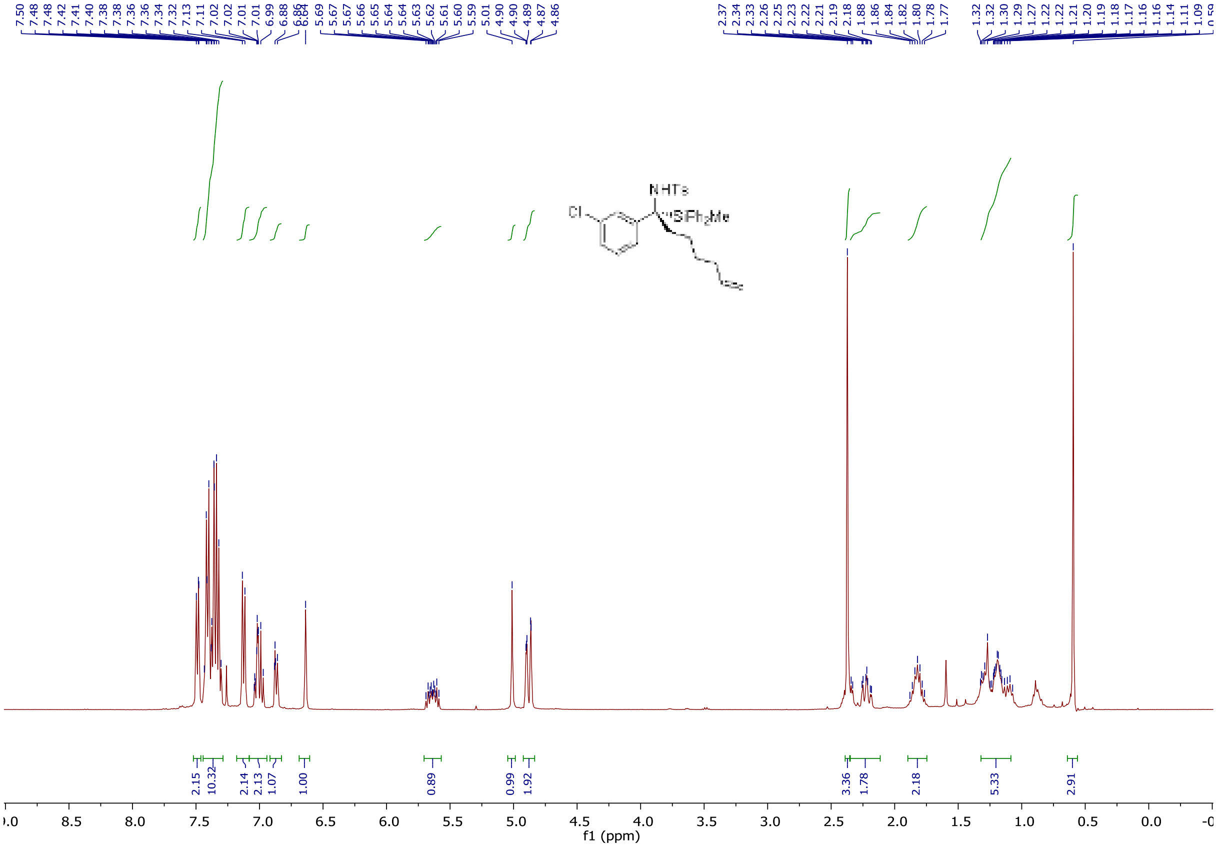

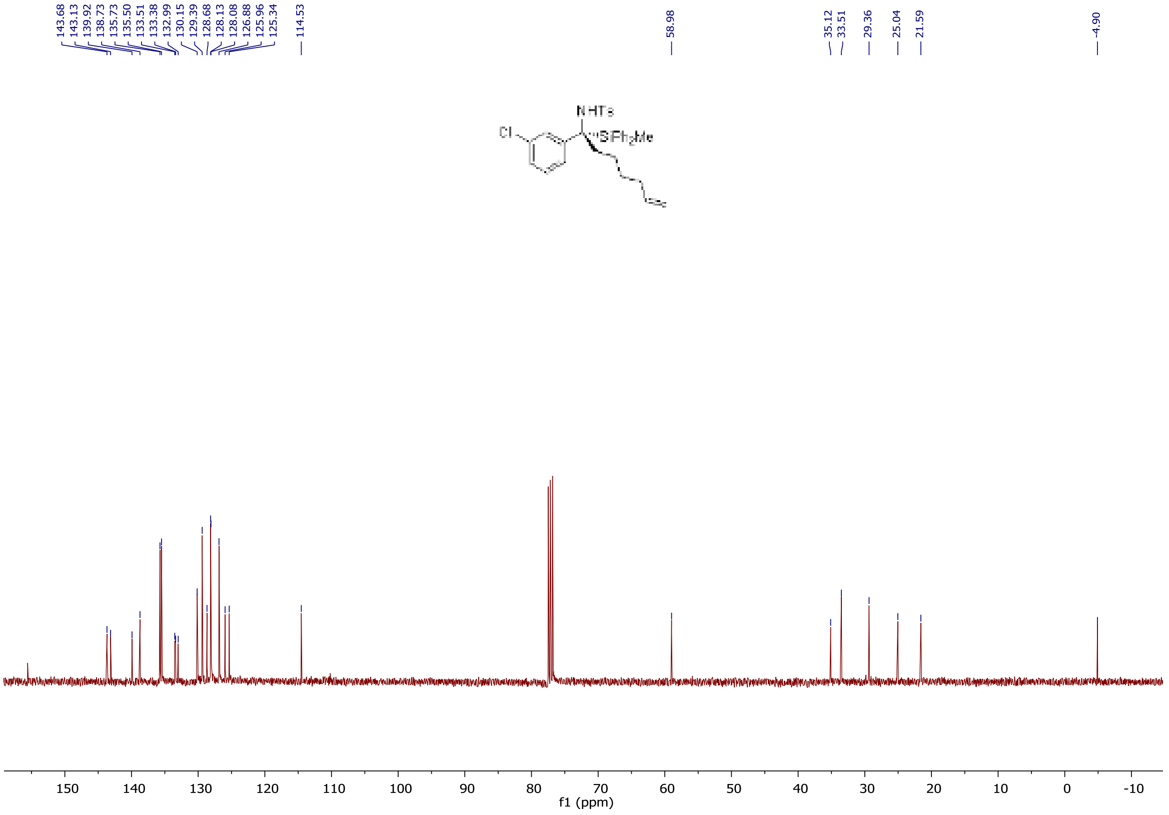

Supplementary Figure 81. 1H, 13C-NMR spectra of product **7g**.

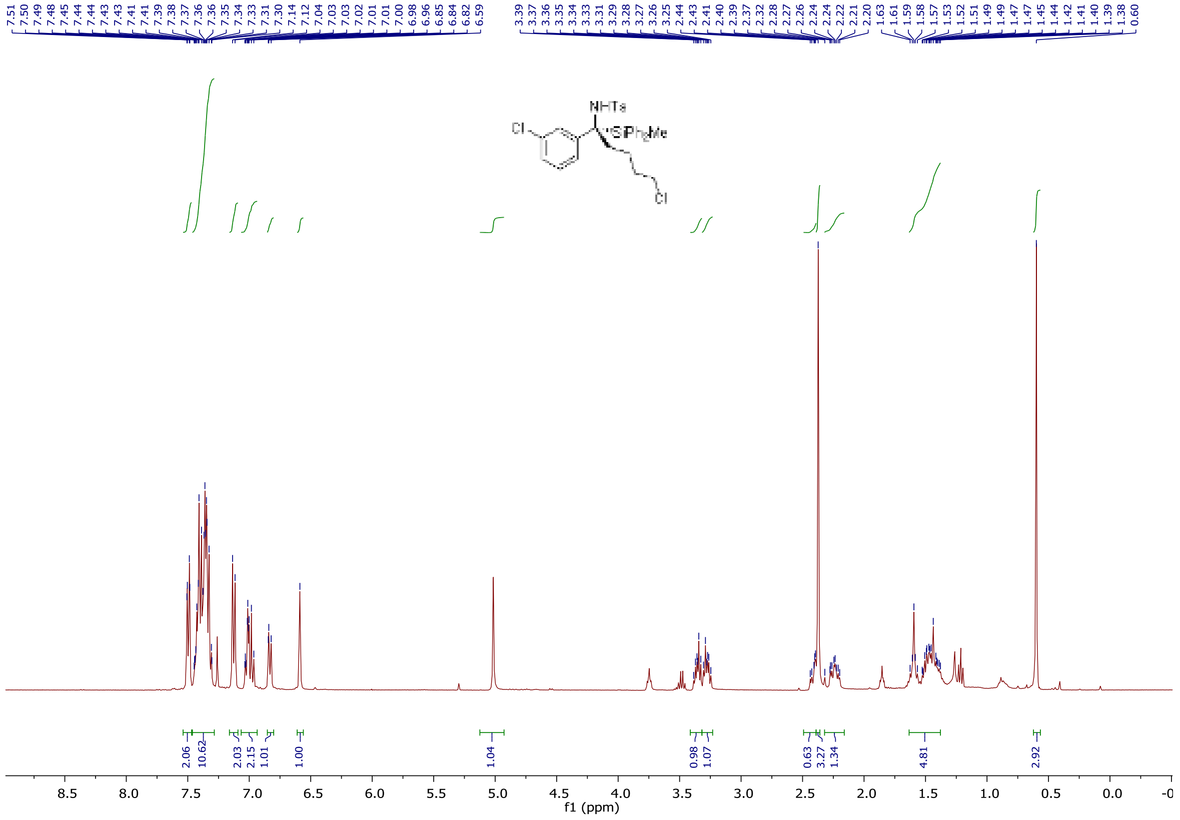

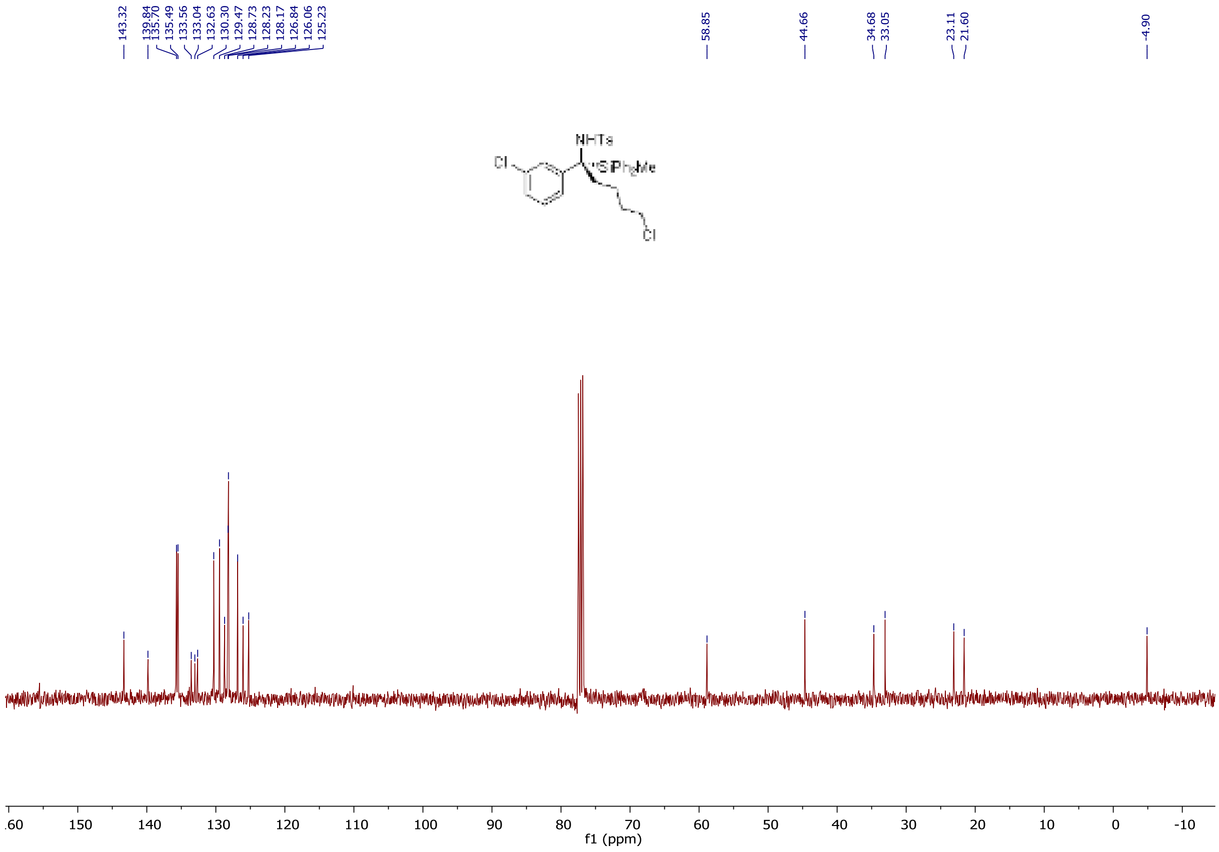

Supplementary Figure 82. 1H, 13C-NMR spectra of product **7h**.

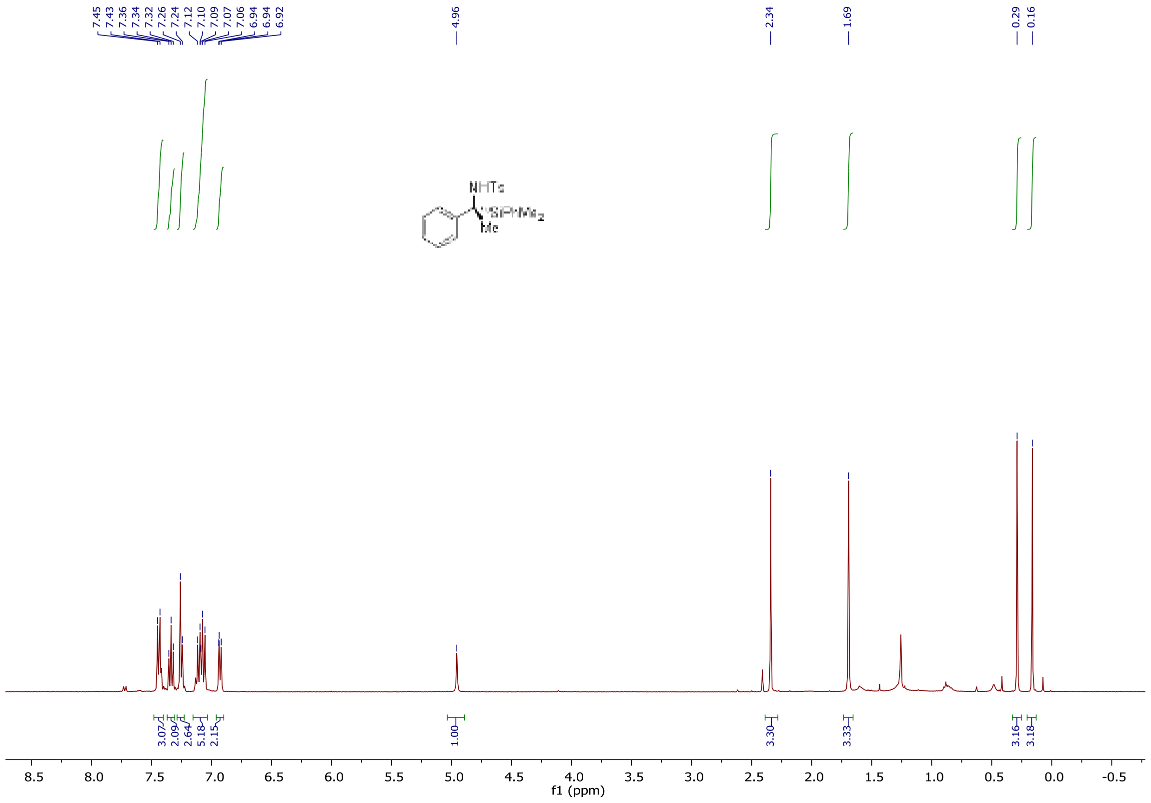

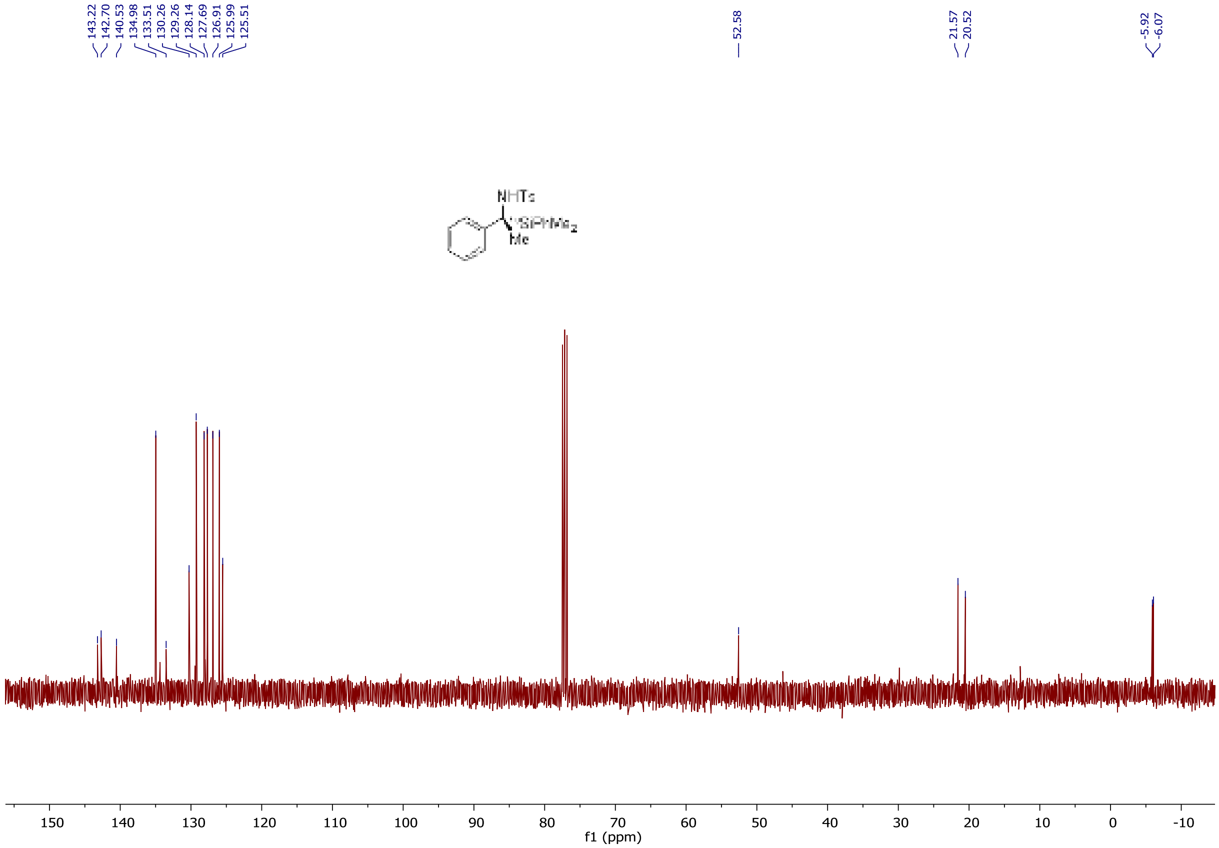

Supplementary Figure 83. 1H, 13C-NMR spectra of product **8**.

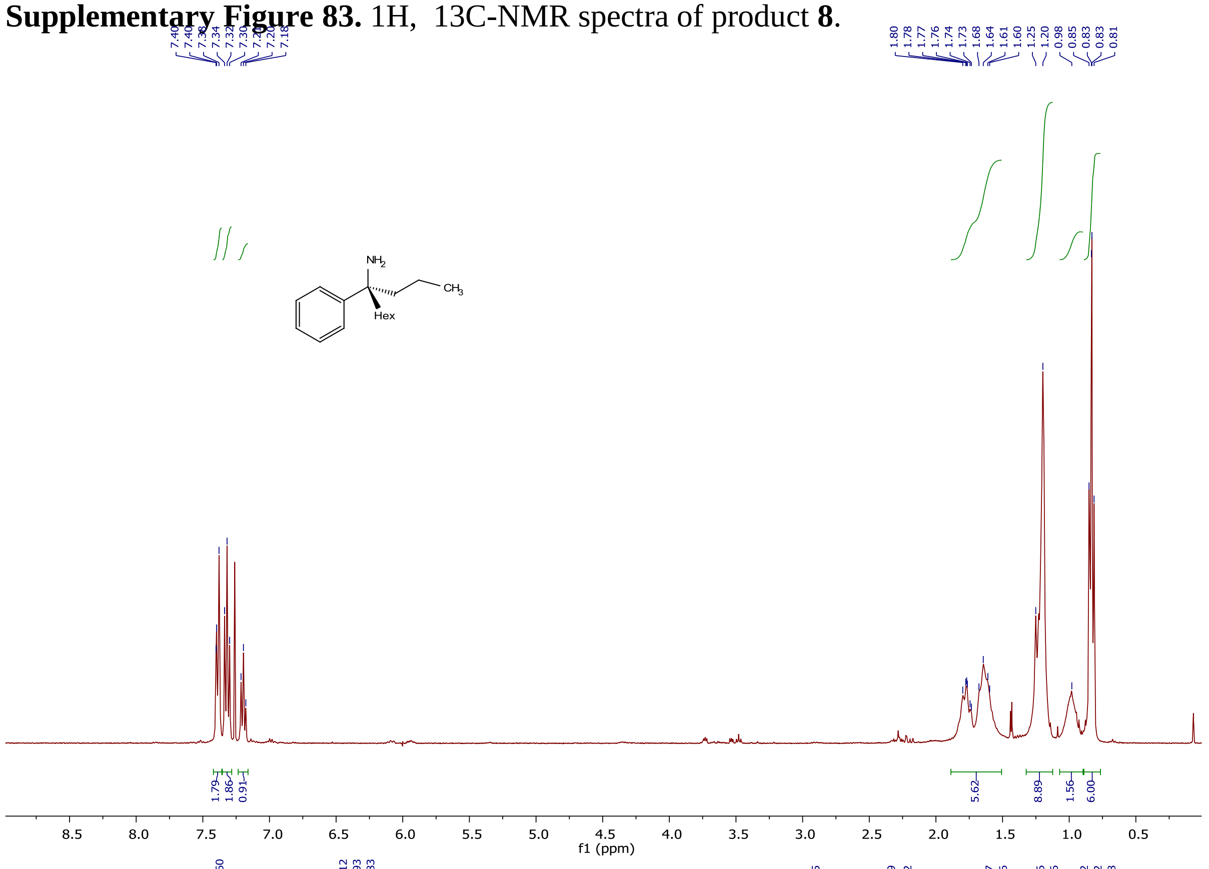

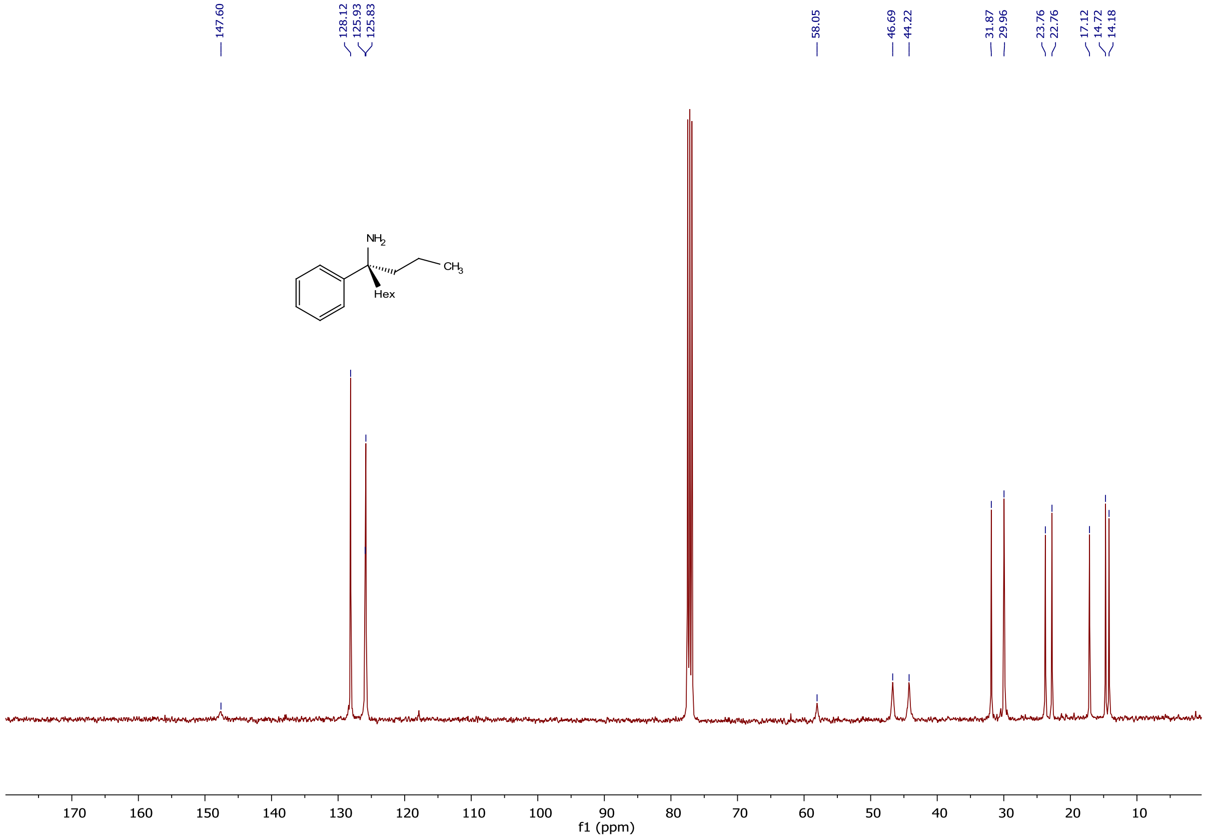

Supplementary Figure 84. HPLC spectra of products **2a.**

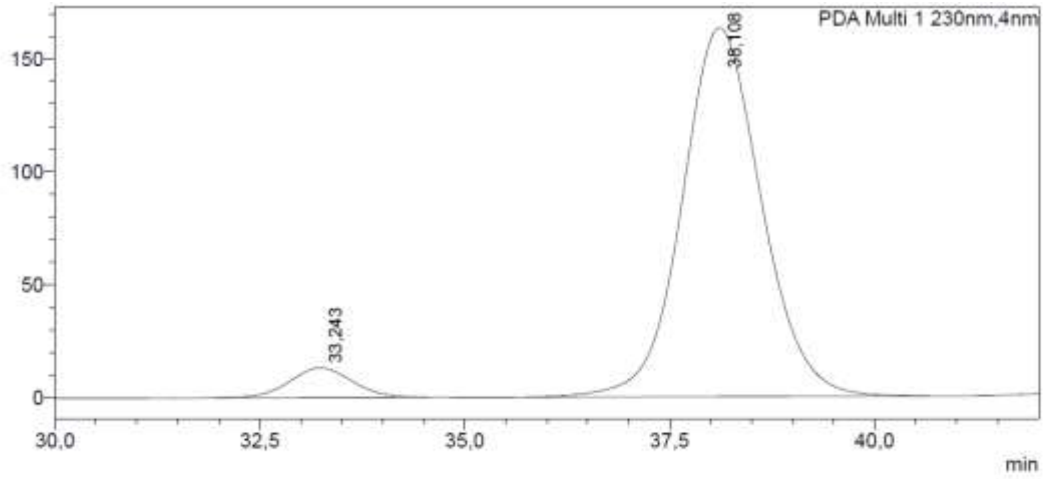

<Peak Table>

PDA Ch1 230nm

Peak#	Ret. Time	Area	Height	Area%
1	33,243	697669	13097	5,975
2	38,108	10978269	163221	94,025
Total		11675938	176318	100,000

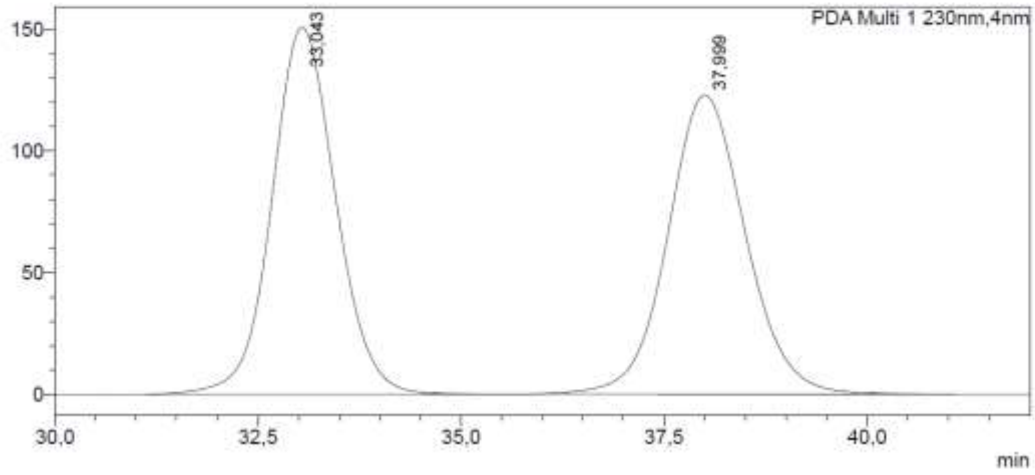

<Peak Table>

PDA Ch1 230nm

Peak#	Ret. Time	Area	Height	Area%
1	33,043	8238268	150518	50,141
2	37,999	8191823	122557	49,859
Total		16430091	273075	100,000

Supplementary Figure 85. HPLC spectra of products **2ab.**

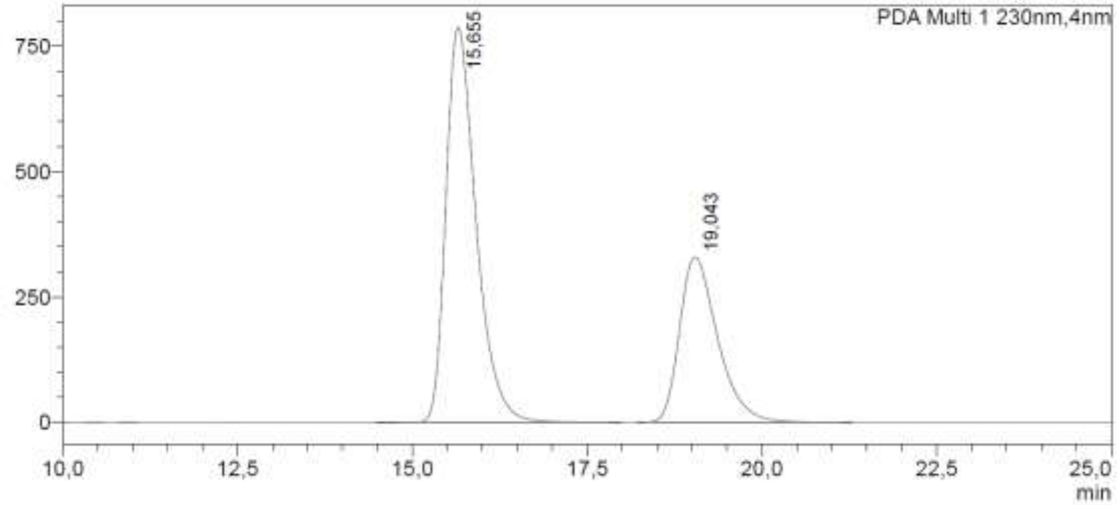

<Peak Table>

PDA Ch1 230nm

Peak#	Ret. Time	Area	Height	Area%
1	15,655	23665874	787689	65,423
2	19,043	12507943	329781	34,577
Total		36173817	1117470	100,000

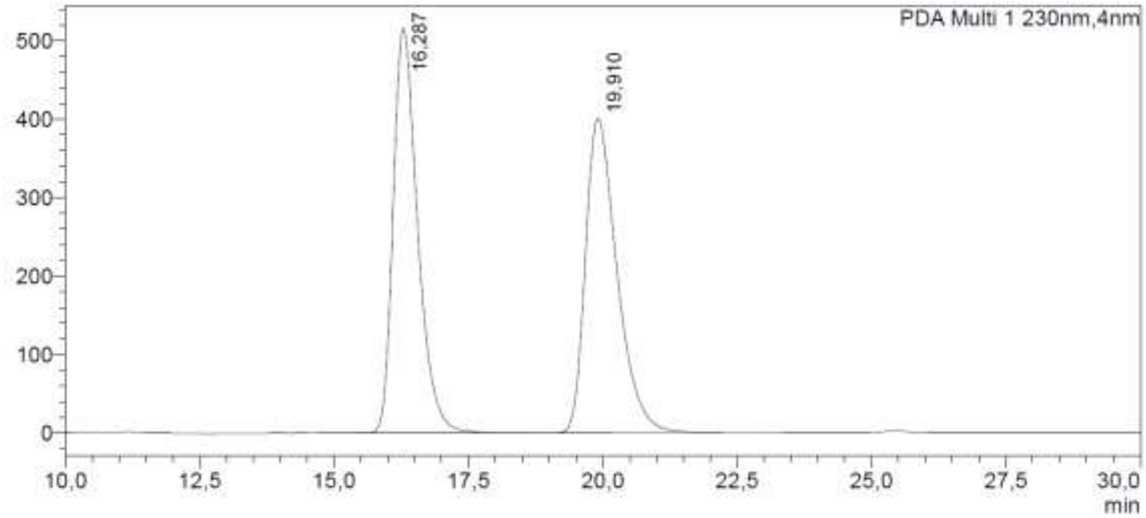

<Peak Table>

PDA Ch1 230nm

Peak#	Ret. Time	Area	Height	Area%
1	16,287	16361212	515709	49,958
2	19,910	16388780	400886	50,042
Total		32749992	916595	100,000

Supplementary Figure 86. HPLC spectra of products **2ac.**

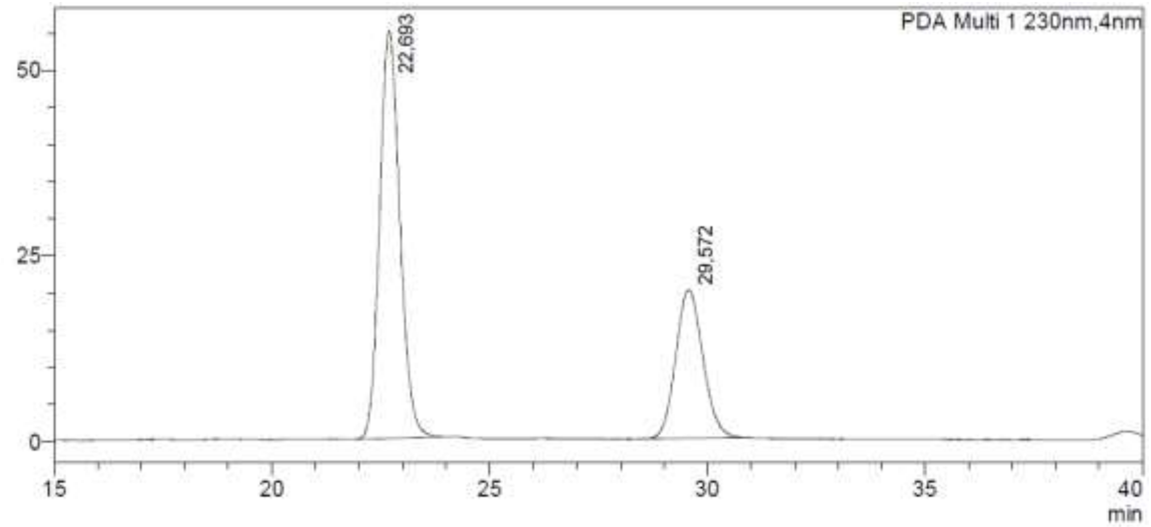

<Peak Table>

PDA Ch1 230nm

Peak#	Ret. Time	Area	Height	Area%
1	22,693	1775576	54869	67,209
2	29,572	866297	19989	32,791
Total		2641873	74858	100,000

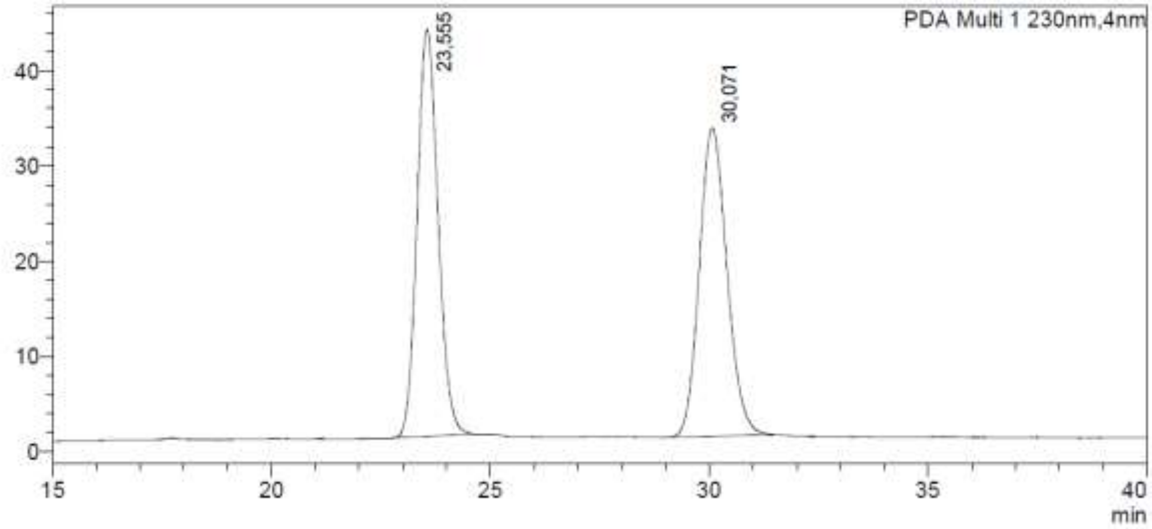

<Peak Table>

PDA Ch1 230nm

Peak#	Ret. Time	Area	Height	Area%
1	23,555	1420073	42711	50,023
2	30,071	1418767	32334	49,977
Total		2838840	75045	100,000

Supplementary Figure 87. HPLC spectra of products **5a.**

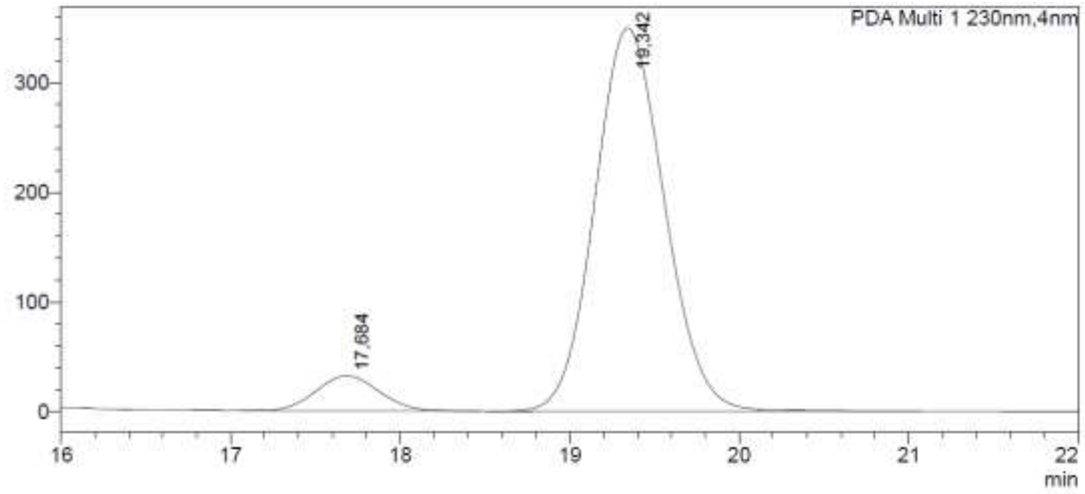

<Peak Table>

PDA Ch1 230nm

Peak#	Ret. Time	Area	Height	Area%
1	17,684	830815	31879	7,700
2	19,342	9959170	348945	92,300
Total		10789985	380824	100,000

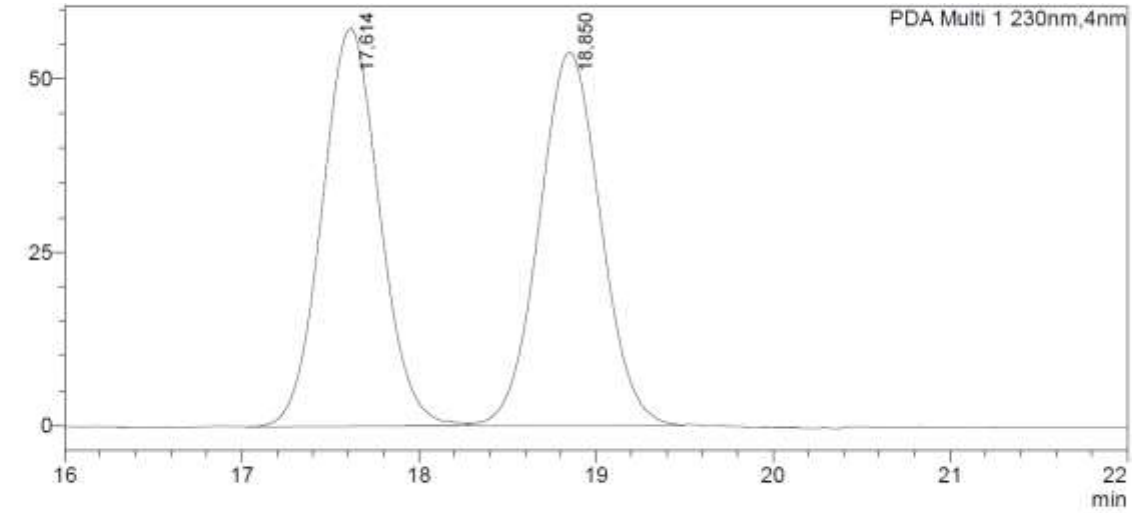

<Peak Table>

PDA Ch1 230nm

Peak#	Ret. Time	Area	Height	Area%
1	17,614	1302134	57349	49,999
2	18,850	1302201	53857	50,001
Total		2604335	111207	100,000

Supplementary Figure 88. HPLC spectra of products **5b.**

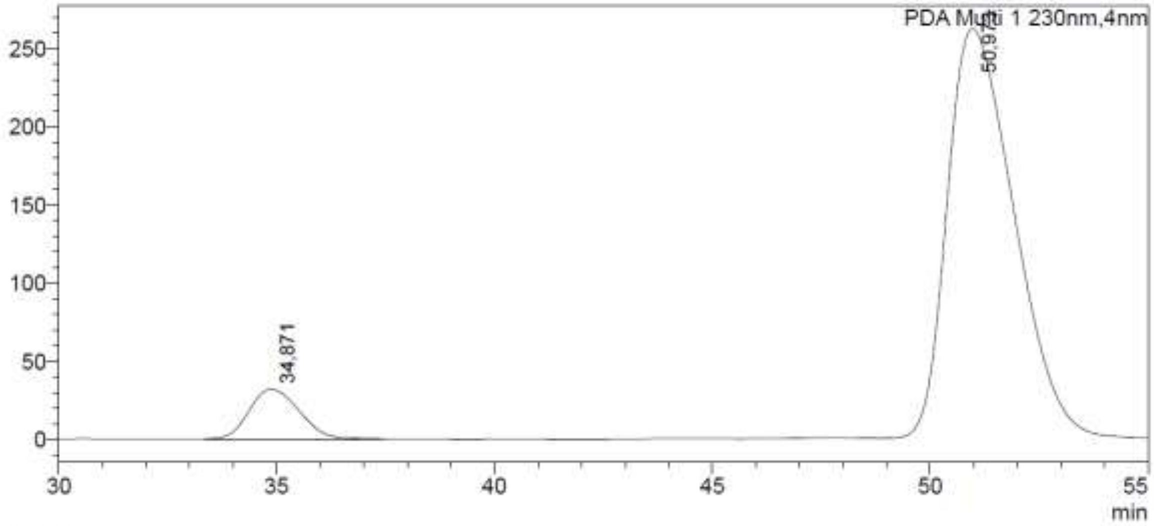

<Peak Table>

PDA Ch1 230nm

Peak#	Ret. Time	Area	Height	Area%
1	34,871	2511190	31688	8,265
2	50,973	27873554	261907	91,735
Total		30384745	293595	100,000

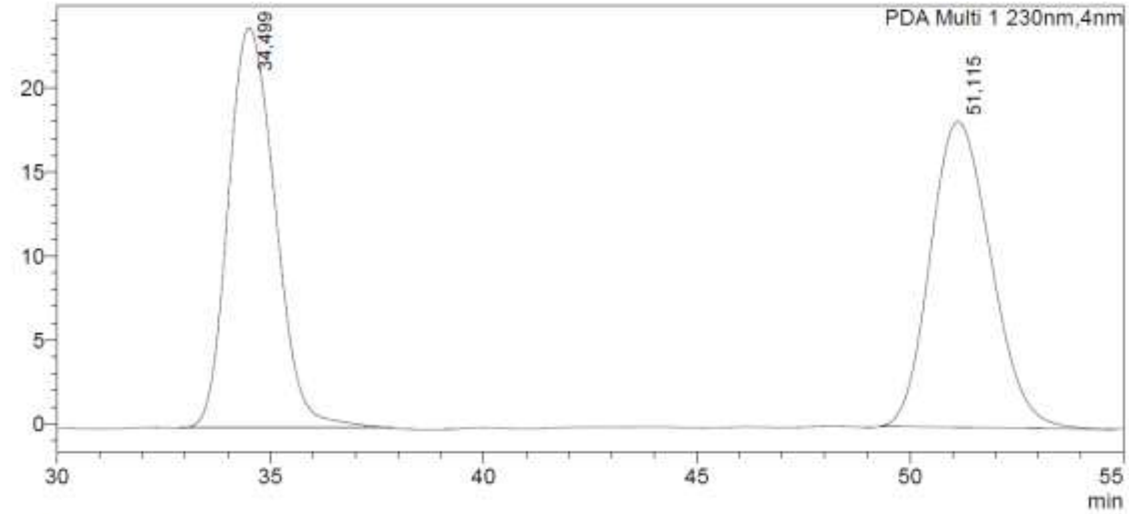

<Peak Table>

PDA Ch1 230nm

Peak#	Ret. Time	Area	Height	Conc.	Unit	Mark	Name
1	34,499	1825018	23839	0,000		M	
2	51,115	1800050	18241	0,000		M	
Total		3625068	42079				

Supplementary Figure 89. HPLC spectra of products **5c.**

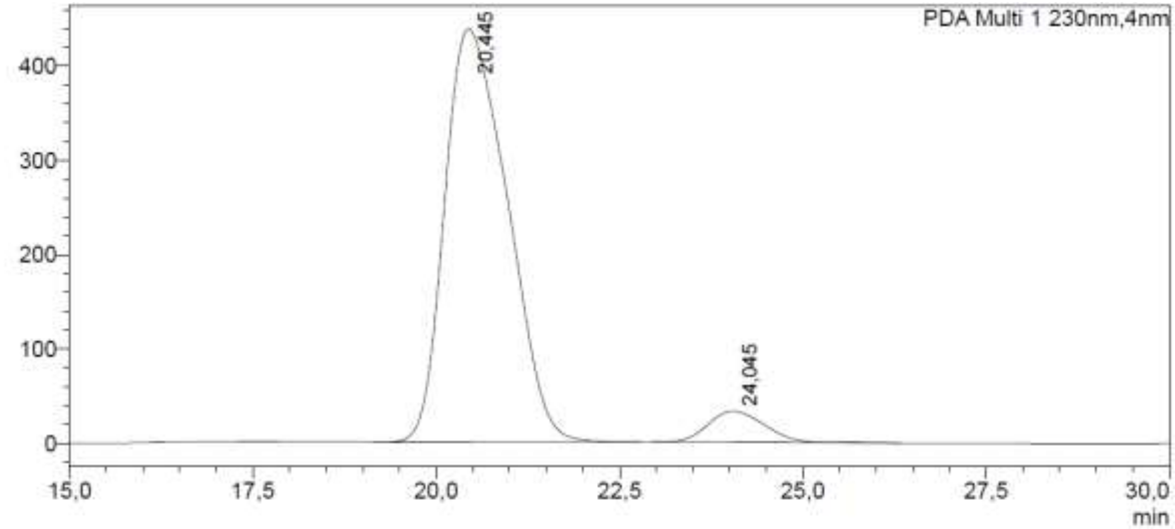

<Peak Table>

PDA Ch1 230nm

Peak#	Ret. Time	Area	Height	Area%
1	20,445	26129020	438659	93,628
2	24,045	1778329	32844	6,372
Total		27907348	471503	100,000

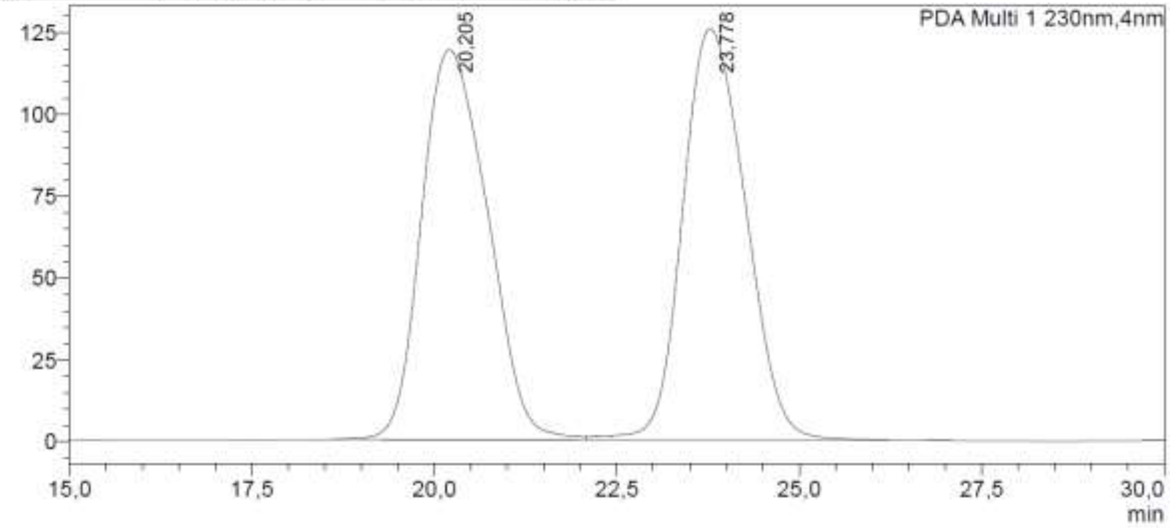

<Peak Table>

PDA Ch1 230nm

Peak#	Ret. Time	Area	Height	Area%
1	20,205	7480802	119073	49,896
2	23,778	7512048	125525	50,104
Total		14992850	244598	100,000

Supplementary Figure 90. HPLC spectra of products **5d.**

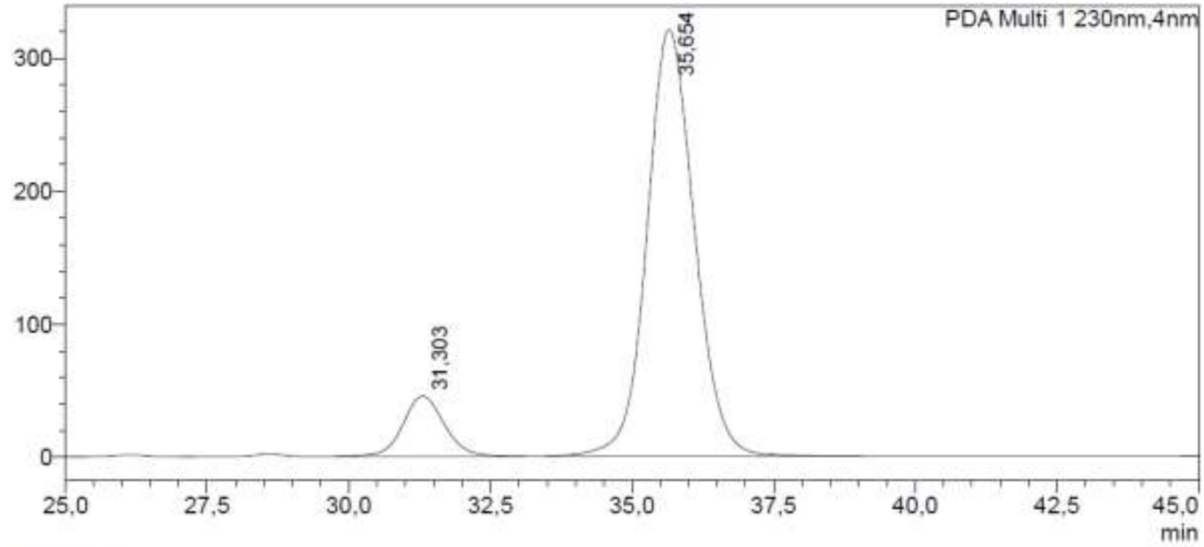

<Peak Table>

PDA Ch1 230nm

Peak#	Ret. Time	Area	Height	Area%
1	31,303	2287670	45115	10,936
2	35,654	18630896	320214	89,064
Total		20918566	365330	100,000

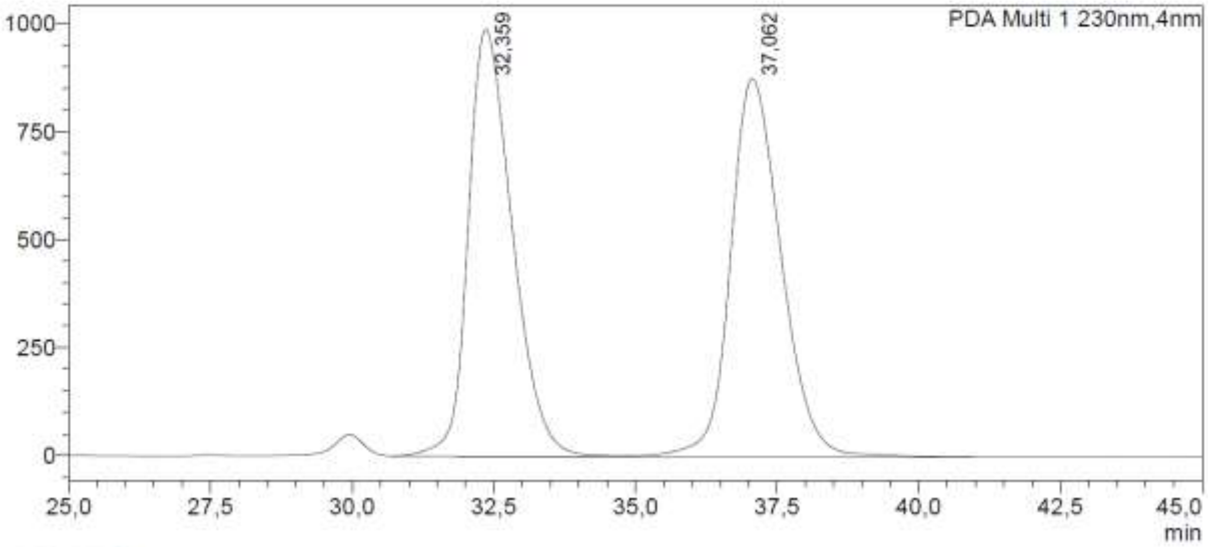

<Peak Table>

PDA Ch1 230nm

Peak#	Ret. Time	Area	Height	Area%
1	32,359	53531913	987861	49,635
2	37,062	54318395	874738	50,365
Total		107850308	1862599	100,000

Supplementary Figure 91. HPLC spectra of products **5e.**

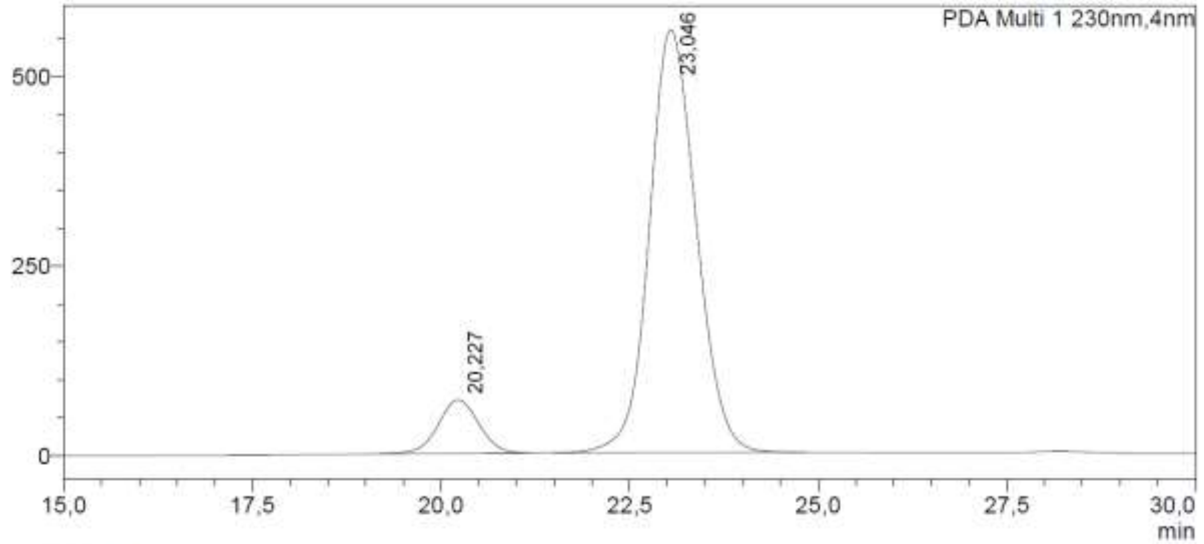

<Peak Table>

PDA Ch1 230nm

Peak#	Ret. Time	Area	Height	Area%
1	20,227	2633967	70431	9,816
2	23,046	24199148	557911	90,184
Total		26833115	628342	100,000

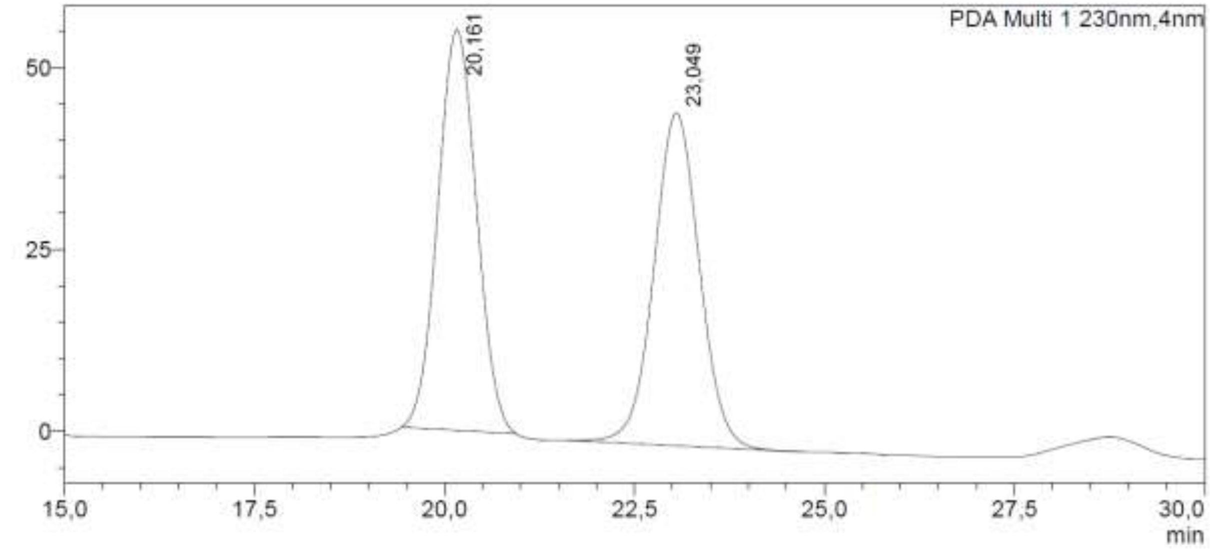

<Peak Table>

PDA Ch1 230nm

Peak#	Ret. Time	Area	Height	Area%
1	20,161	1950921	55035	50,571
2	23,049	1906866	45738	49,429
Total		3857786	100773	100,000

Supplementary Figure 92. HPLC spectra of products **5f.**

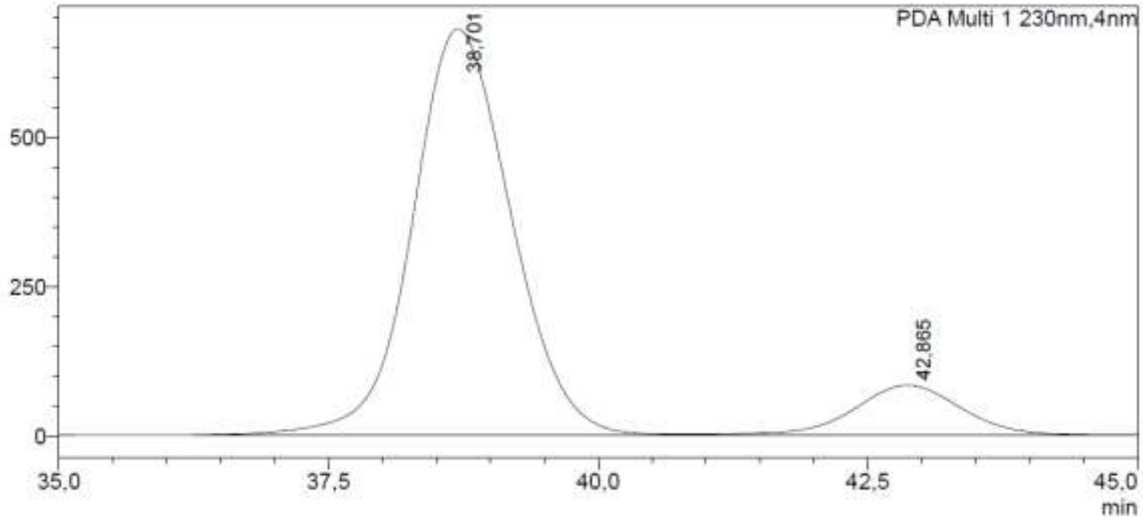

<Peak Table>

PDA Ch1 230nm

Peak#	Ret. Time	Area	Height	Area%
1	38,701	43078913	678880	88,289
2	42,865	5714220	82918	11,711
Total		48793133	761798	100,000

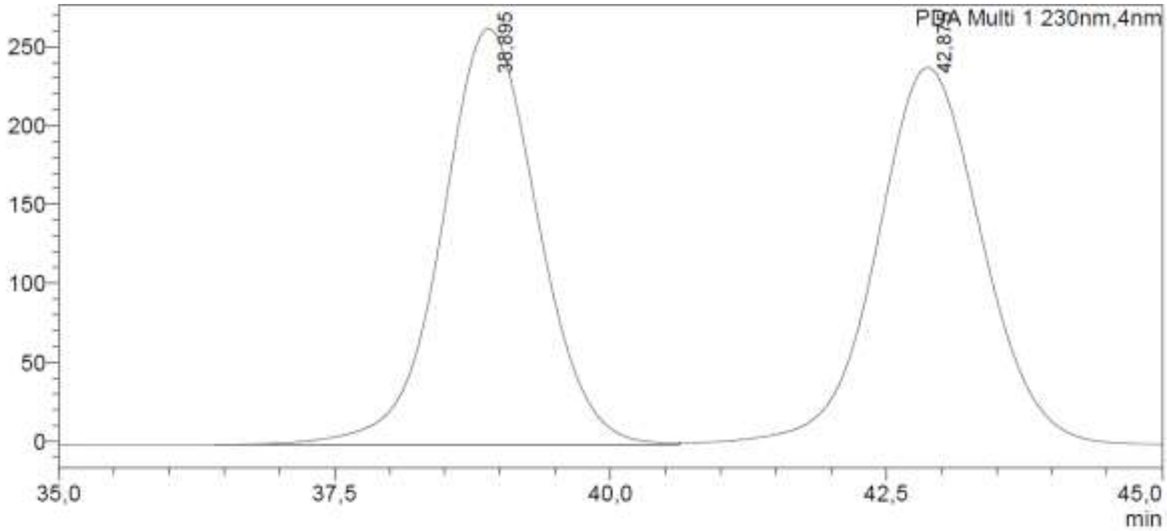

<Peak Table>

PDA Ch1 230nm

Peak#	Ret. Time	Area	Height	Area%
1	38,895	15951198	263308	50,022
2	42,875	15937169	238547	49,978
Total		31888368	501855	100,000

Supplementary Figure 93. HPLC spectra of products **5g.**

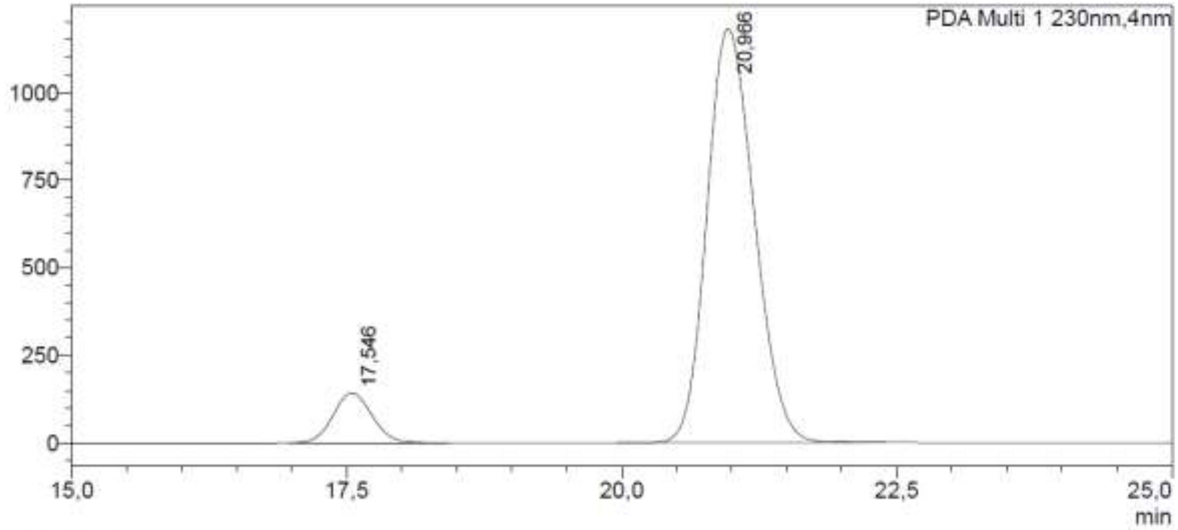

<Peak Table>

PDA Ch1 230nm

Peak#	Ret. Time	Area	Height	Area%
1	17,546	3468786	141867	8,930
2	20,966	35374454	1180145	91,070
Total		38843240	1322012	100,000

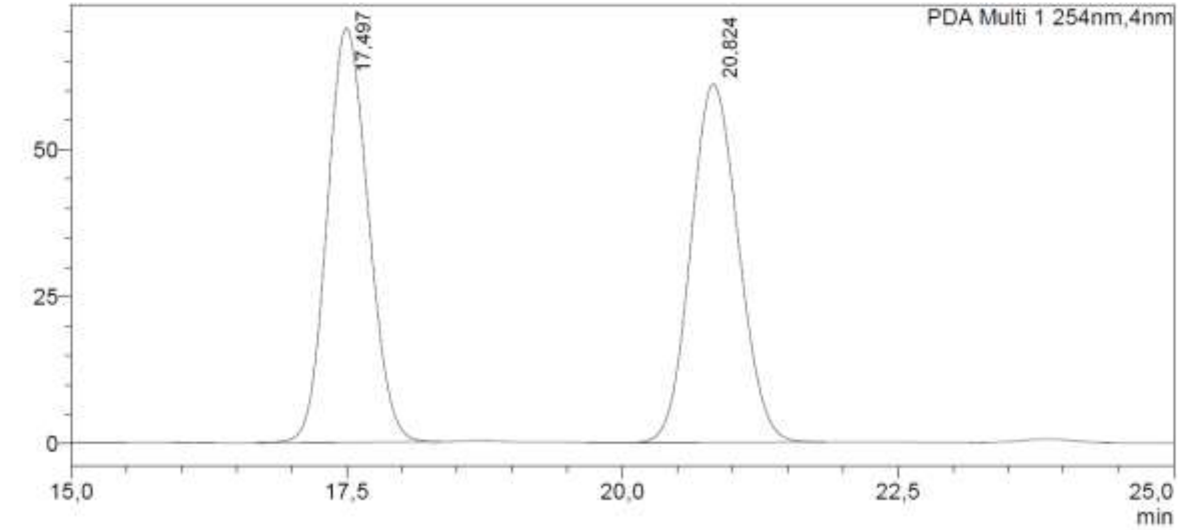

<Peak Table>

PDA Ch1 254nm

Peak#	Ret. Time	Area	Height	Area%
1	17,497	1823479	70369	49,885
2	20,824	1831893	60833	50,115
Total		3655372	131202	100,000

Supplementary Figure 94. HPLC spectra of products **5h.**

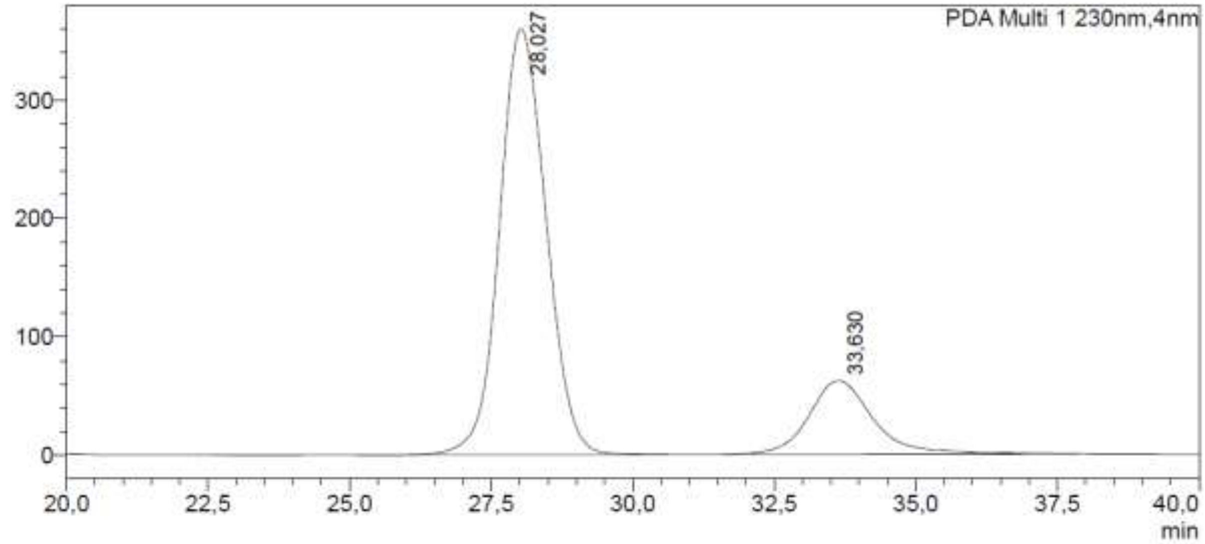

<Peak Table>

PDA Ch1 230nm

Peak#	Ret. Time	Area	Height	Area%
1	28,027	20587678	358990	80,665
2	33,630	4934636	62083	19,335
Total		25522313	421073	100,000

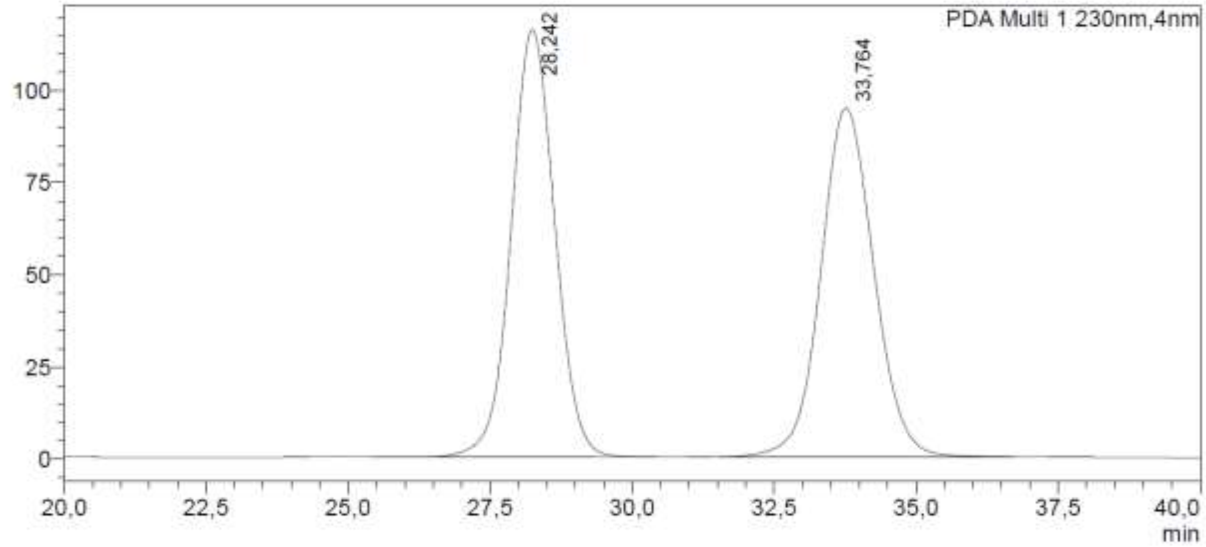

<Peak Table>

PDA Ch1 230nm

Peak#	Ret. Time	Area	Height	Area%
1	28,242	6202782	116009	50,177
2	33,764	6159031	94679	49,823
Total		12361813	210689	100,000

Supplementary Figure 95. HPLC spectra of products **5i.**

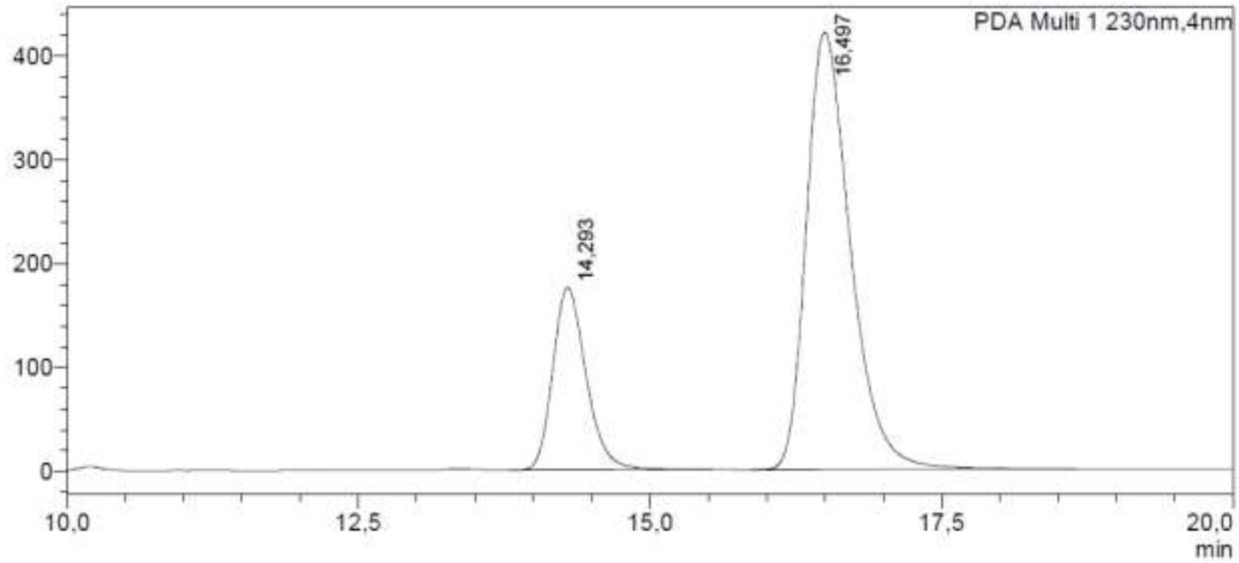

<Peak Table>

PDA Ch1 230nm

Peak#	Ret. Time	Area	Height	Area%
1	14,293	3501312	175476	24,019
2	16,497	11075768	420785	75,981
Total		14577081	596261	100,000

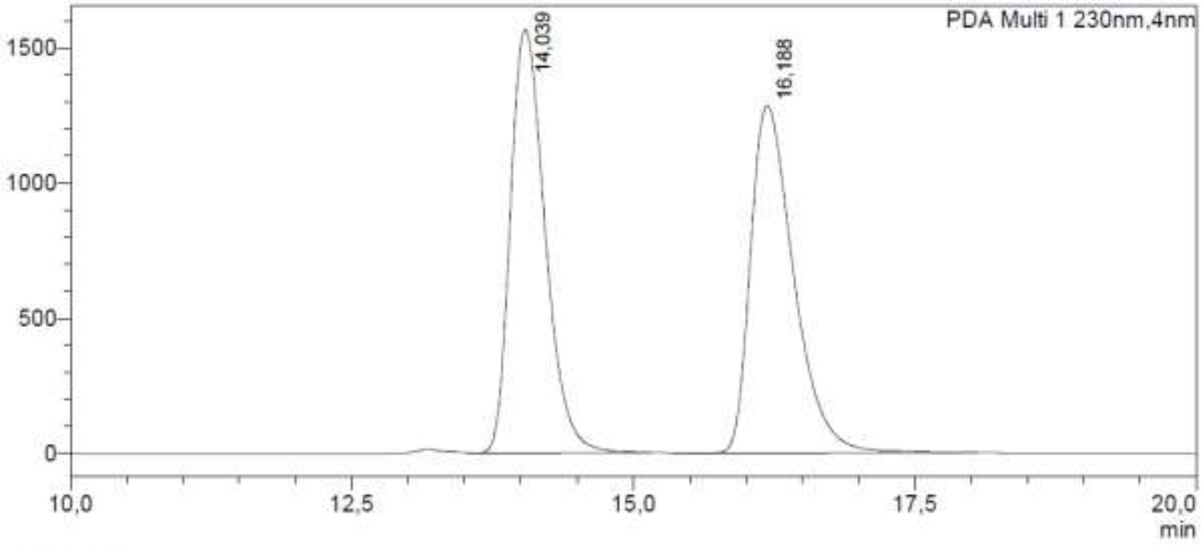

<Peak Table>

PDA Ch1 230nm

Peak#	Ret. Time	Area	Height	Area%
1	14,039	33532217	1564389	49,440
2	16,188	34291876	1284916	50,560
Total		67824094	2849304	100,000

Supplementary Figure 96. HPLC spectra of products **5j.**

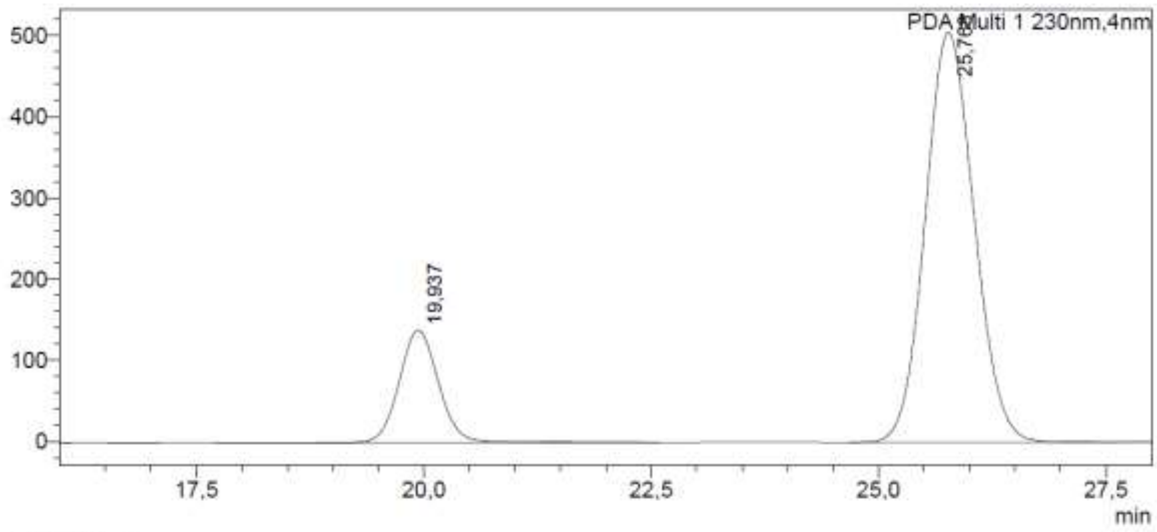

<Peak Table>

PDA Ch1 230nm

Peak#	Ret. Time	Area	Height	Area%
1	19,937	4199470	138380	18,138
2	25,768	18953116	505457	81,862
Total		23152586	643837	100,000

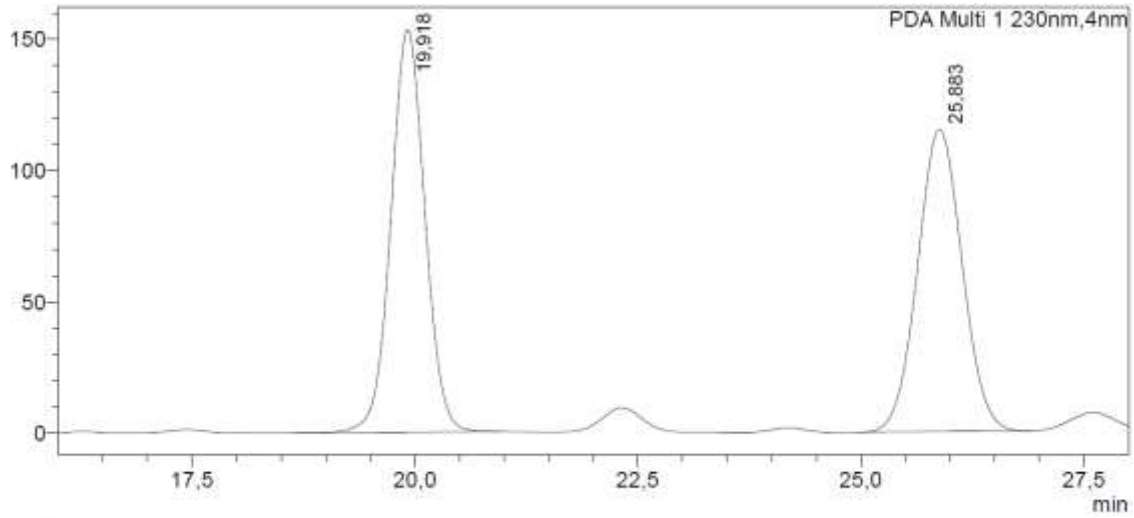

<Peak Table>

PDA Ch1 230nm

Peak#	Ret. Time	Area	Height	Area%
1	19,918	4068734	153279	50,355
2	25,883	4011417	115127	49,645
Total		8080151	268406	100,000

Supplementary Figure 97. HPLC spectra of products **6b.**

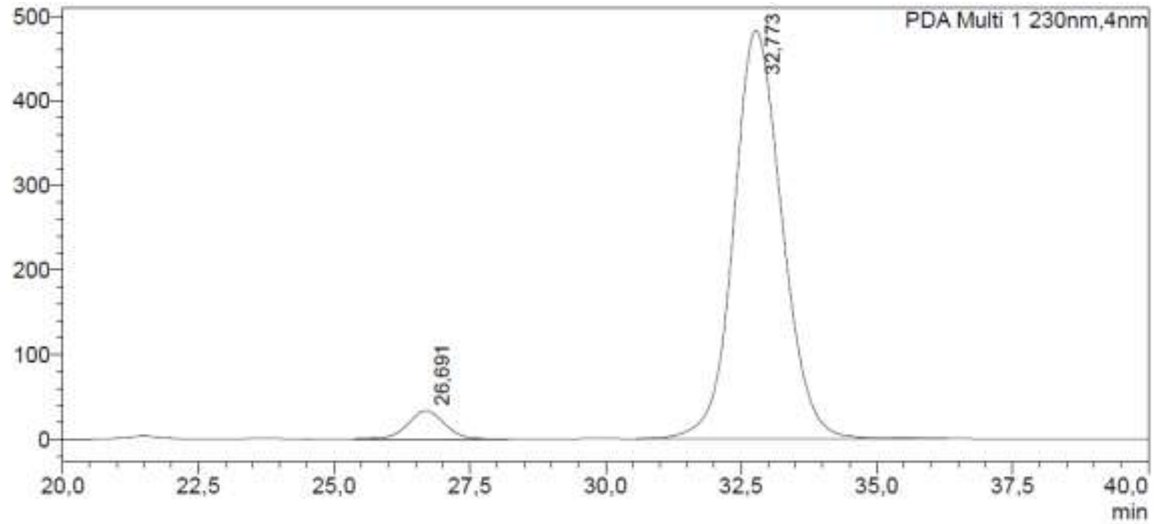

<Peak Table>

PDA Ch1 230nm

Peak#	Ret. Time	Area	Height	Area%
1	26,691	1548544	33211	4,920
2	32,773	29925893	482094	95,080
Total		31474436	515305	100,000

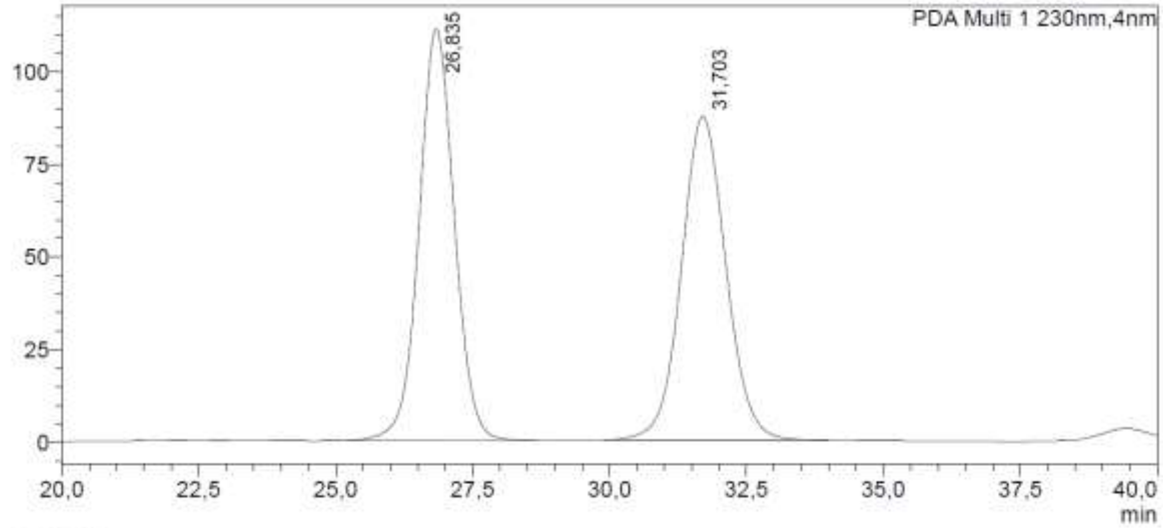

<Peak Table>

PDA Ch1 230nm

Peak#	Ret. Time	Area	Height	Area%
1	26,835	4989233	111127	50,136
2	31,703	4962229	87457	49,864
Total		9951462	198584	100,000

Supplementary Figure 98. HPLC spectra of products **6c.**

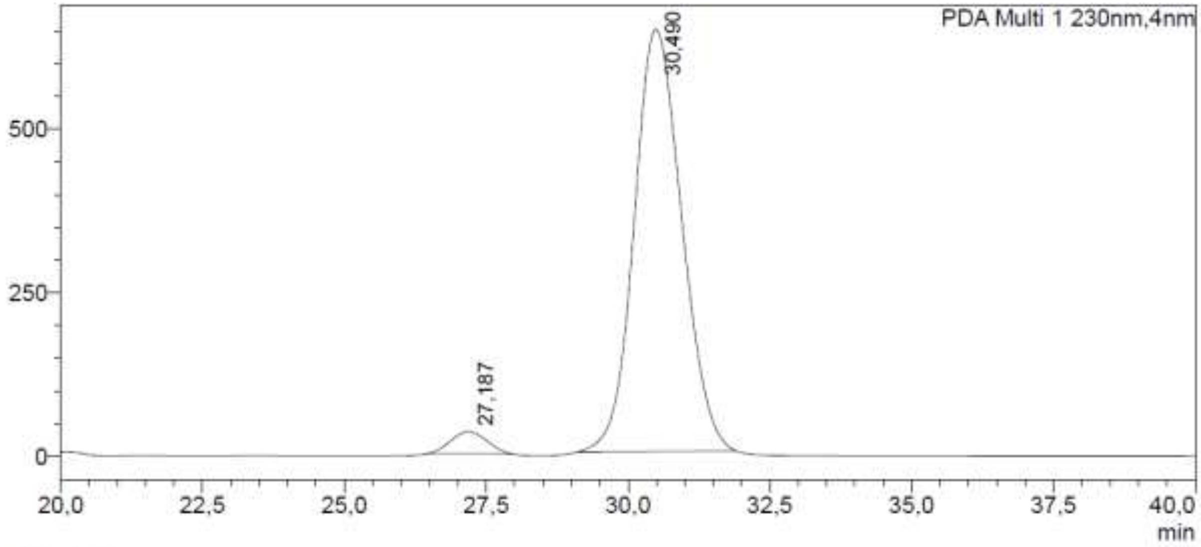

<Peak Table>

PDA Ch1 230nm

Peak#	Ret. Time	Area	Height	Area%
1	27,187	1484016	33693	3,790
2	30,490	37670792	645192	96,210
Total		39154808	678886	100,000

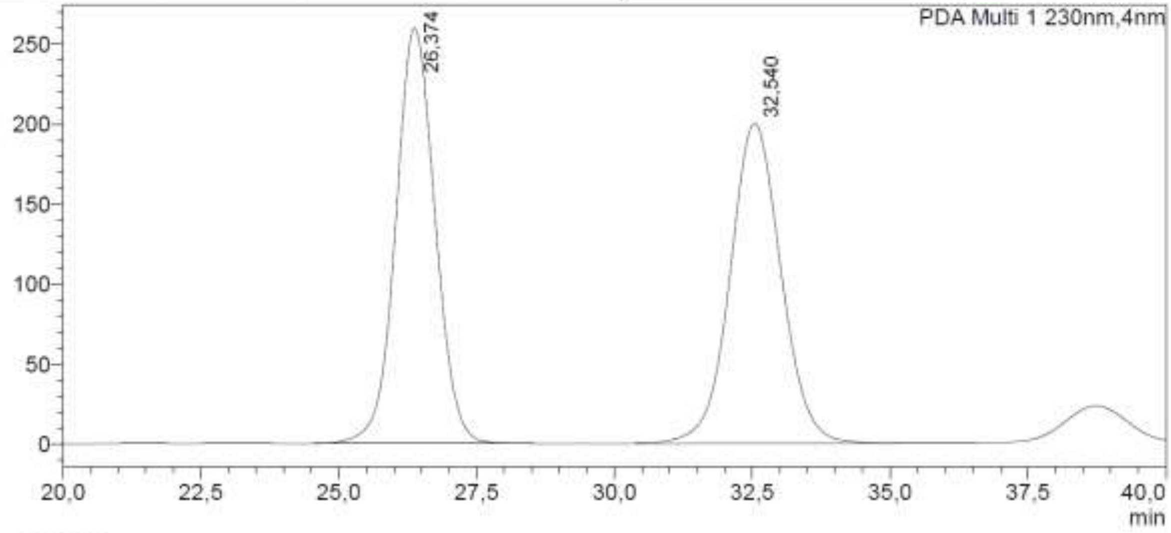

<Peak Table>

PDA Ch1 230nm

Peak#	Ret. Time	Area	Height	Area%
1	26,374	12942243	259623	50,116
2	32,540	12882269	199826	49,884
Total		25824512	459449	100,000

Supplementary Figure 99. HPLC spectra of products **6d.**

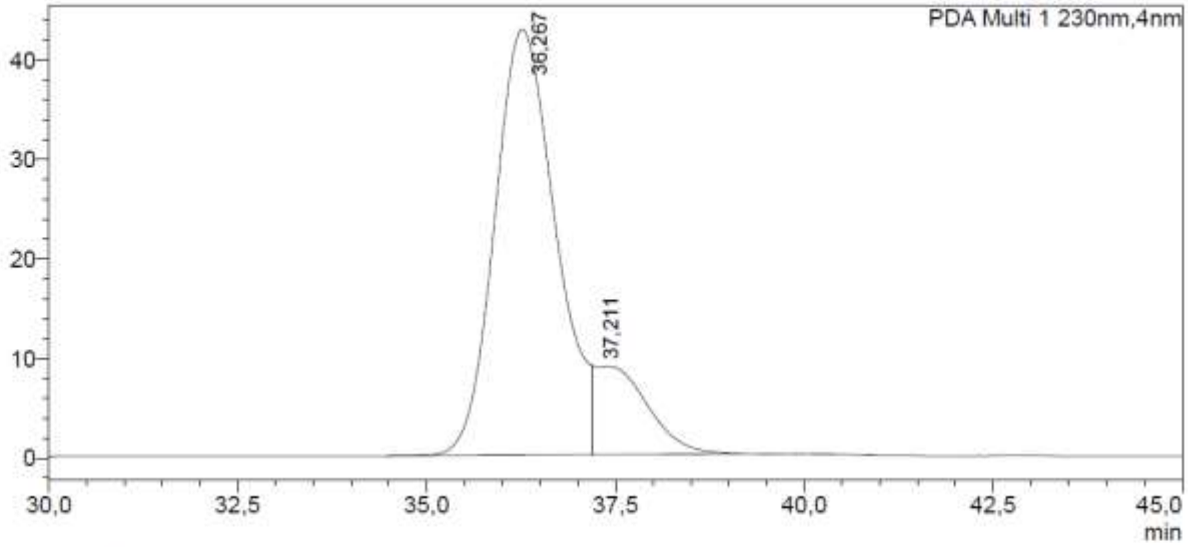

<Peak Table>

PDA Ch1 230nm

Peak#	Ret. Time	Area	Height	Area%
1	36,267	2400564	42793	84,823
2	37,211	429534	8890	15,177
Total		2830098	51683	100,000

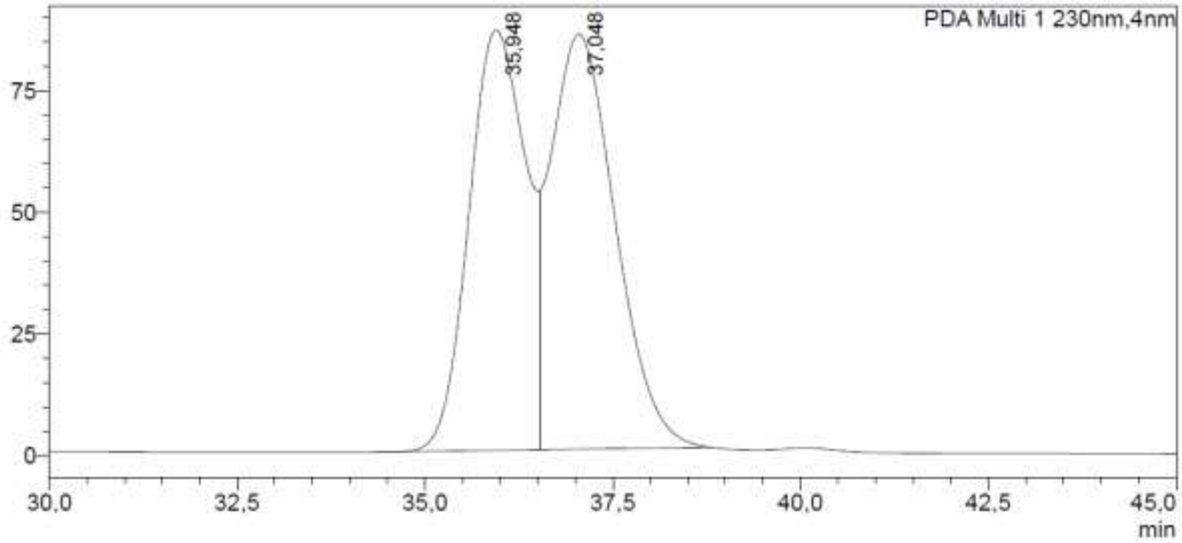

<Peak Table>

PDA Ch1 230nm

Peak#	Ret. Time	Area	Height	Area%
1	35,948	4563294	86395	47,120
2	37,048	5121015	85261	52,880
Total		9684309	171657	100,000

Supplementary Figure 100. HPLC spectra of products **6e.**

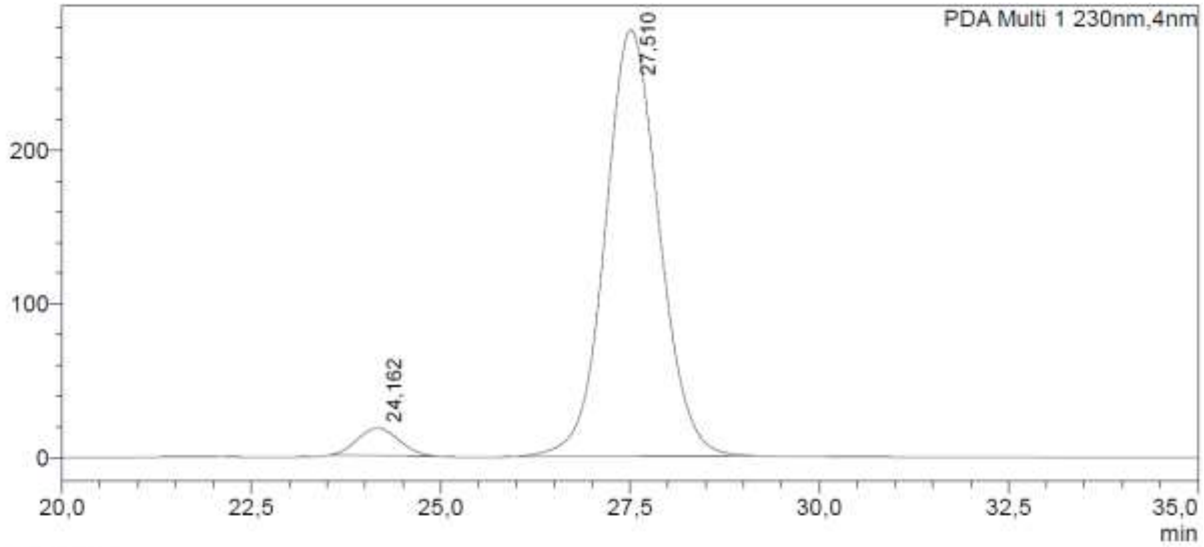

<Peak Table>

PDA Ch1 230nm

Peak#	Ret. Time	Area	Height	Area%
1	24,162	679546	17883	4,722
2	27,510	13710800	277223	95,278
Total		14390346	295107	100,000

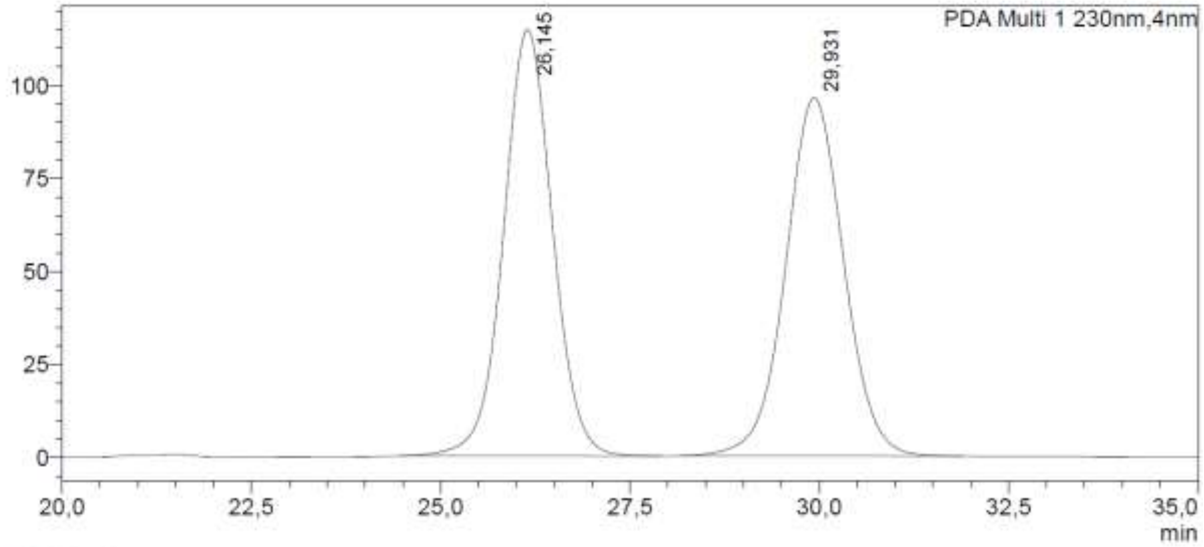

<Peak Table>

PDA Ch1 230nm

Peak#	Ret. Time	Area	Height	Area%
1	26,145	5154446	114483	50,004
2	29,931	5153675	96246	49,996
Total		10308121	210729	100,000

Supplementary Figure 101. HPLC spectra of products **6f.**

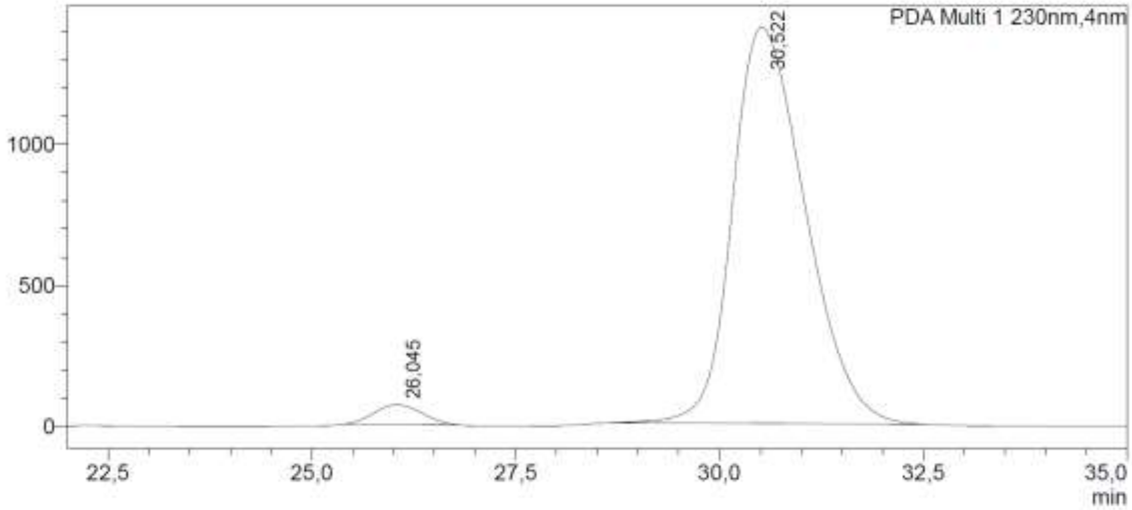

<Peak Table>

PDA Ch1 230nm

Peak#	Ret. Time	Area	Height	Area%
1	26,045	2854315	70383	3,168
2	30,522	87247253	1402940	96,832
Total		90101568	1473323	100,000

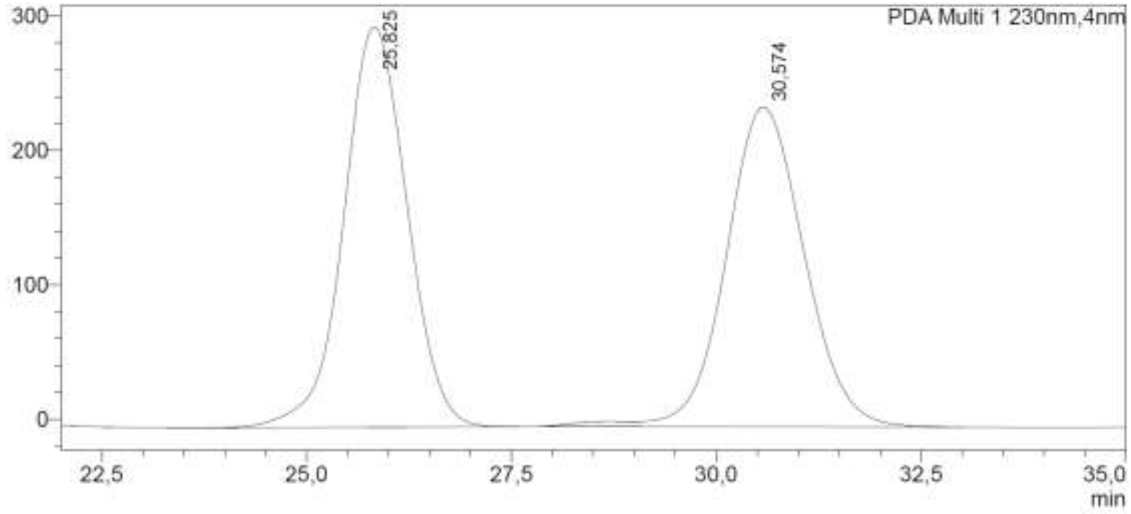

<Peak Table>

PDA Ch1 230nm

Peak#	Ret. Time	Area	Height	Area%
1	25,825	16013391	297375	50,262
2	30,574	15846156	237606	49,738
Total		31859546	534981	100,000

Supplementary Figure 102. HPLC spectra of products **6g.**

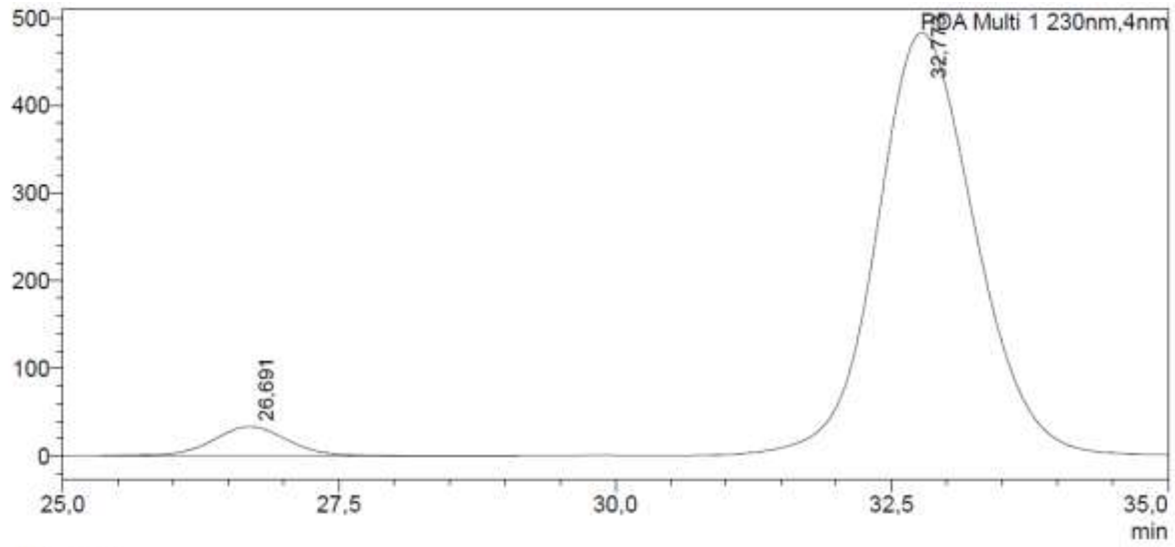

<Peak Table>

PDA Ch1 230nm

Peak#	Ret. Time	Area	Height	Area%
1	26,691	1548544	33211	4,920
2	32,773	29925893	482094	95,080
Total		31474436	515305	100,000

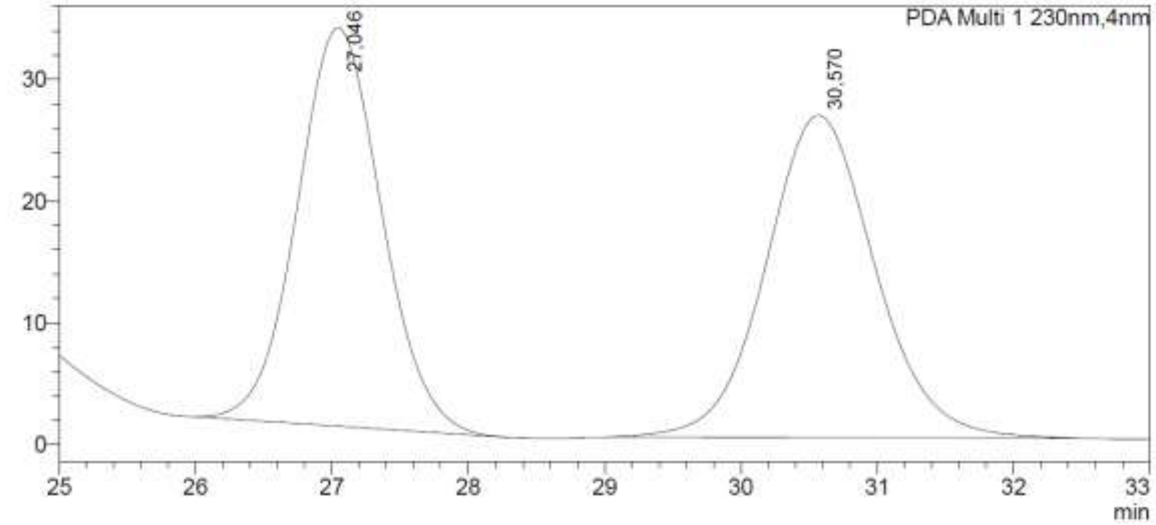

<Peak Table>

PDA Ch1 230nm

Peak#	Ret. Time	Area	Height	Area%
1	27,046	1412384	32742	48,975
2	30,570	1471530	26488	51,025
Total		2883914	59231	100,000

Supplementary Figure 103. HPLC spectra of products **6h.**

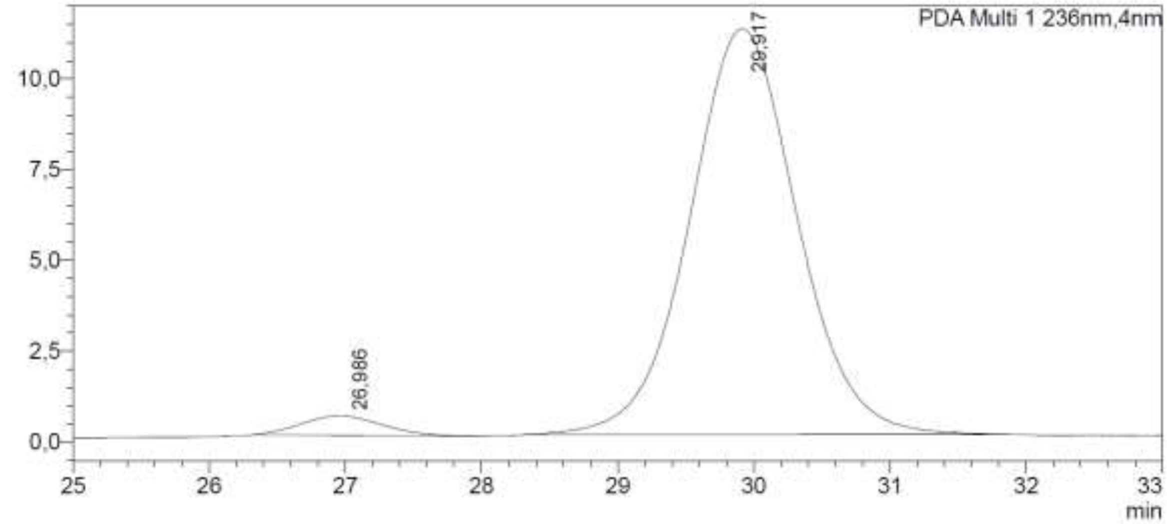

<Peak Table>

PDA Ch1 236nm

Peak#	Ret. Time	Area	Height	Area%
1	26,986	22041	540	3,462
2	29,917	614549	11186	96,538
Total		636590	11725	100,000

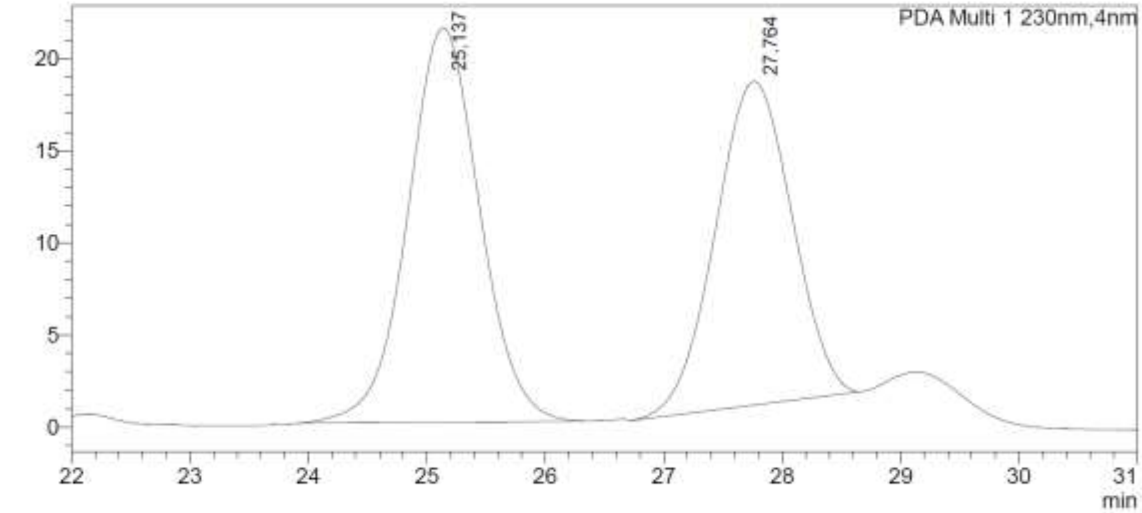

<Peak Table>

PDA Ch1 230nm

Peak#	Ret. Time	Area	Height	Area%
1	25,137	903586	21385	53,400
2	27,764	788516	17552	46,600
Total		1692102	38938	100,000

Supplementary Figure 104. HPLC spectra of products **6i.**

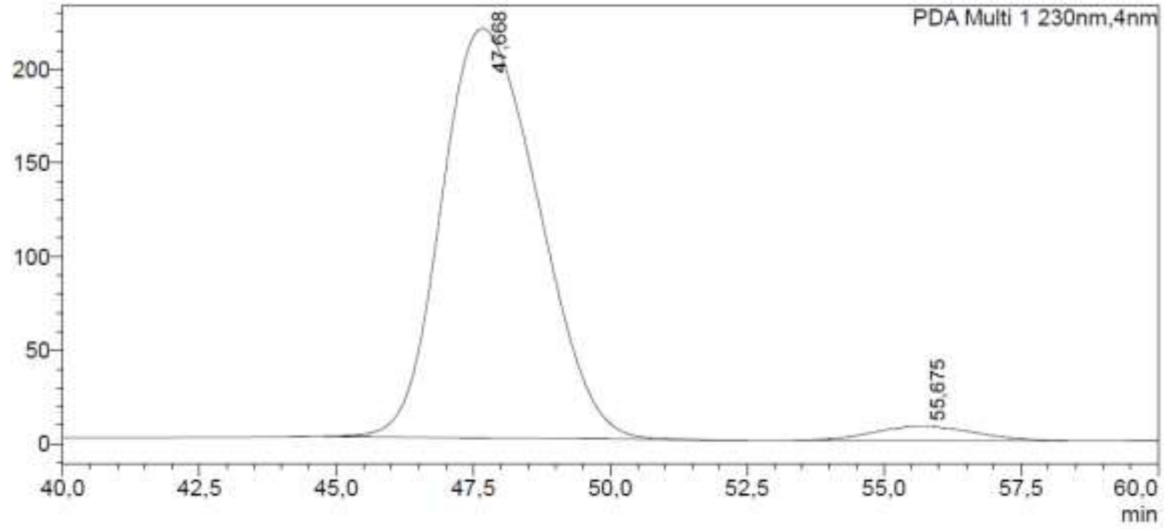

<Peak Table>

PDA Ch1 230nm

Peak#	Ret. Time	Area	Height	Area%
1	47,668	26878661	218535	96,660
2	55,675	928809	7362	3,340
Total		27807470	225897	100,000

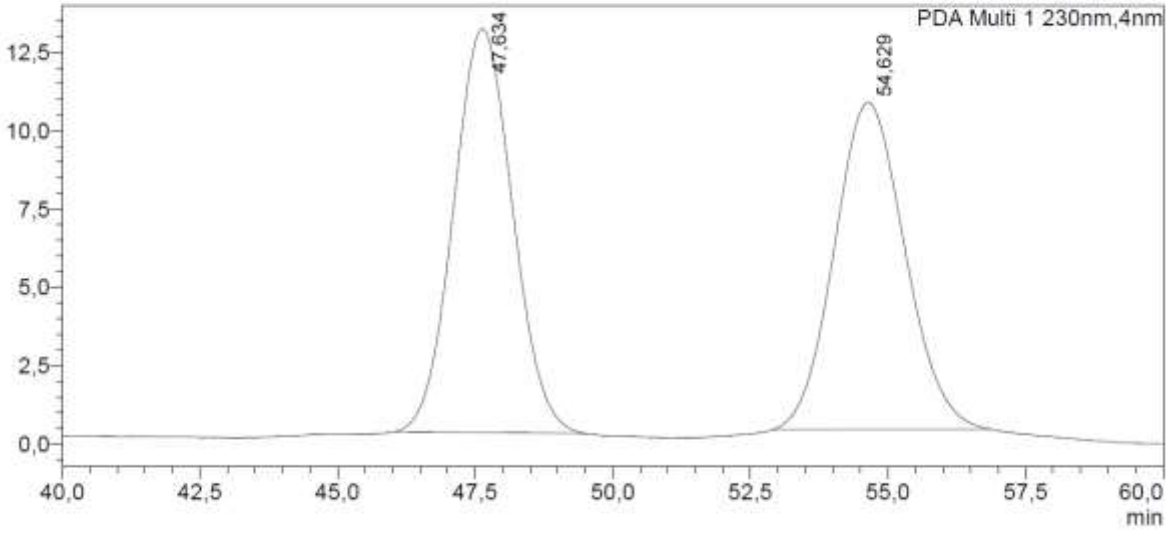

<Peak Table>

PDA Ch1 230nm

Peak#	Ret. Time	Area	Height	Area%
1	47,634	964235	12879	50,856
2	54,629	931785	10435	49,144
Total		1896019	23313	100,000

Supplementary Figure 105. HPLC spectra of products **6j.**

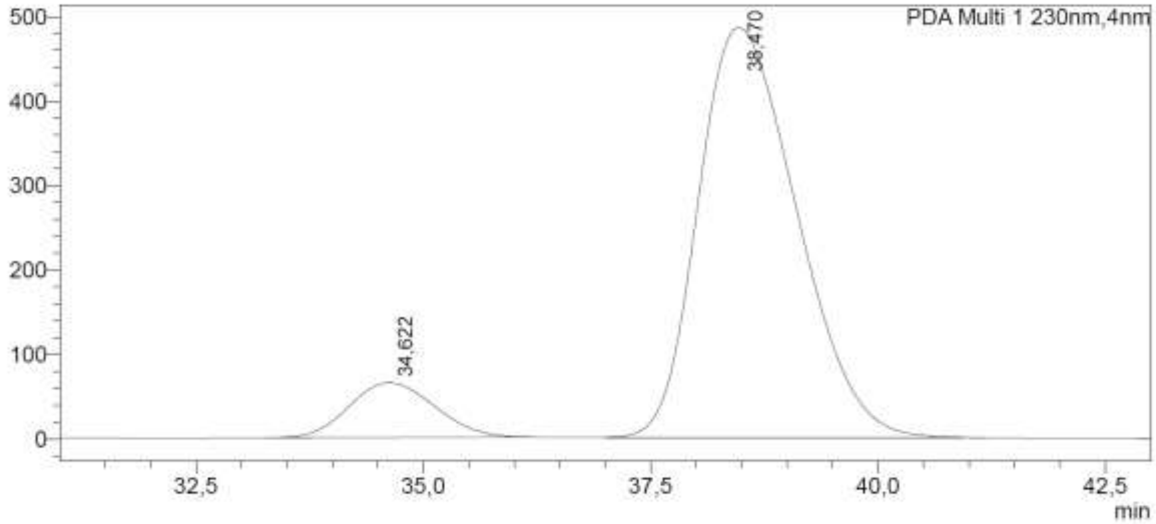

<Peak Table>

PDA Ch1 230nm

Peak#	Ret. Time	Area	Height	Area%
1	34,622	4218643	64504	10,374
2	38,470	36447821	484843	89,626
Total		40666464	549347	100,000

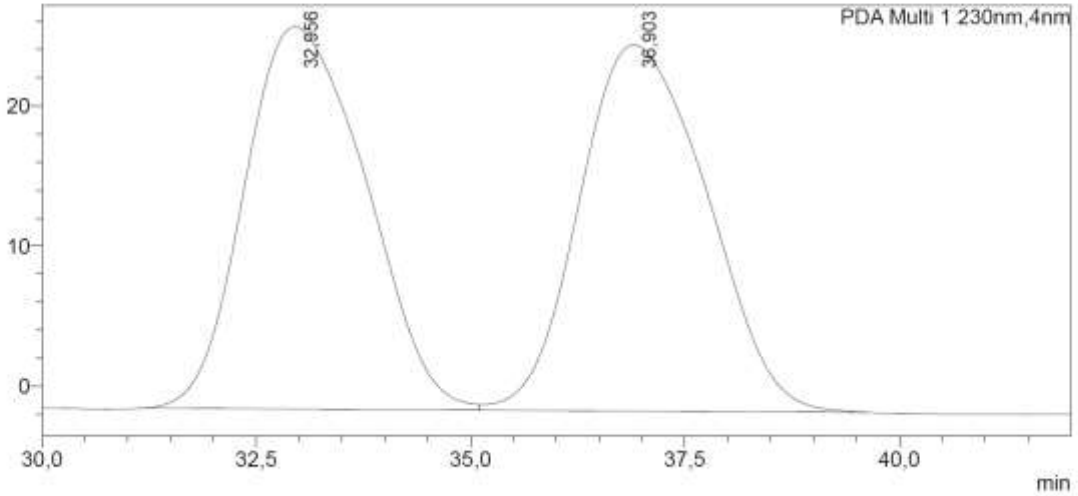

<Peak Table>

PDA Ch1 230nm

Peak#	Ret. Time	Area	Height	Area%
1	32,956	2677709	27225	49,805
2	36,903	2698715	26103	50,195
Total		5376423	53328	100,000

Supplementary Figure 106. HPLC spectra of products **6k.**

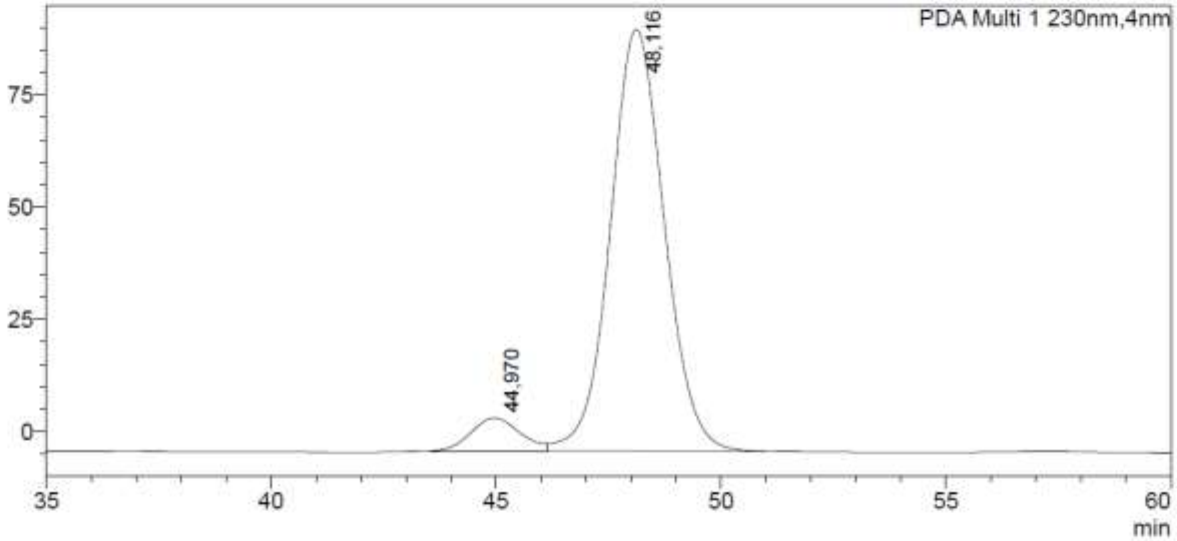

<Peak Table>

PDA Ch1 230nm

Peak#	Ret. Time	Area	Height	Area%
1	44,970	559317	7366	6,647
2	48,116	7854717	93932	93,353
Total		8414035	101297	100,000

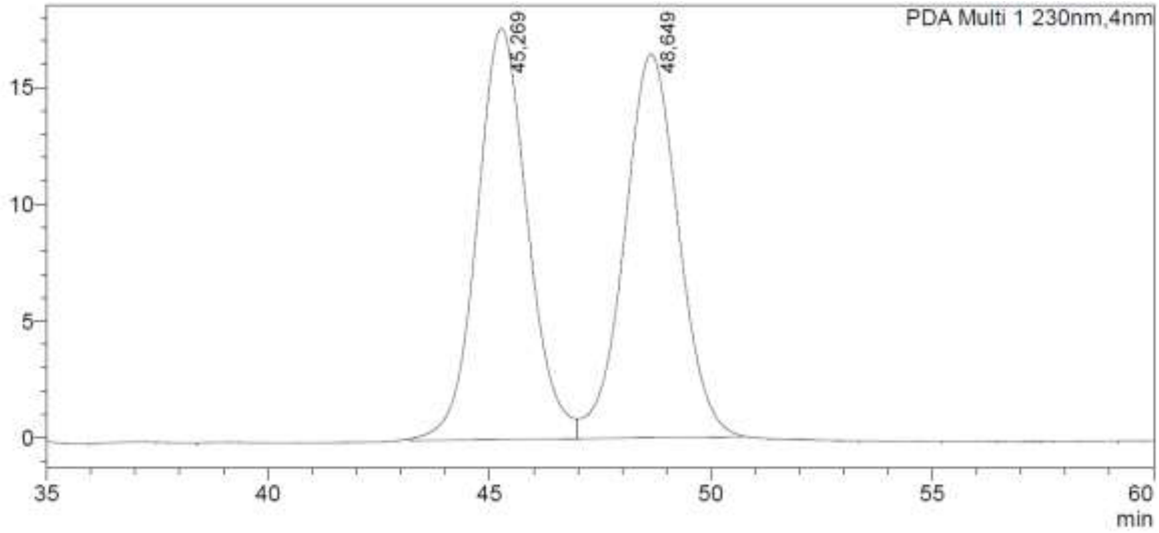

<Peak Table>

PDA Ch1 230nm

Peak#	Ret. Time	Area	Height	Area%
1	45,269	1395981	17591	50,066
2	48,649	1392283	16418	49,934
Total		2788265	34009	100,000

Supplementary Figure 107. HPLC spectra of products **6l.**

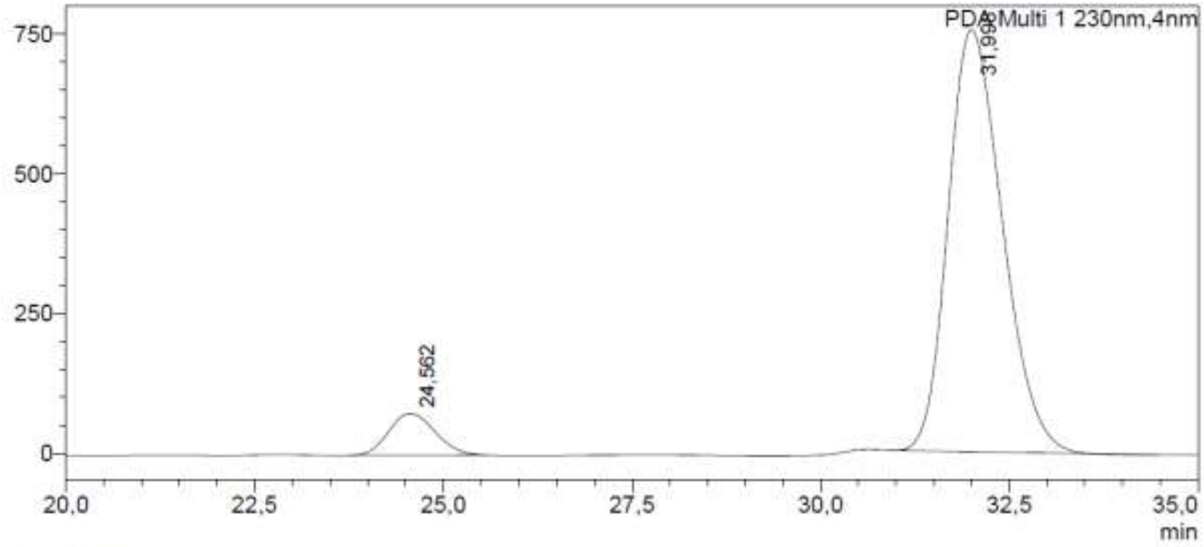

<Peak Table>

PDA Ch1 230nm

Peak#	Ret. Time	Area	Height	Area%
1	24,562	3192205	74586	7,798
2	31,998	37744340	754059	92,202
Total		40936545	828646	100,000

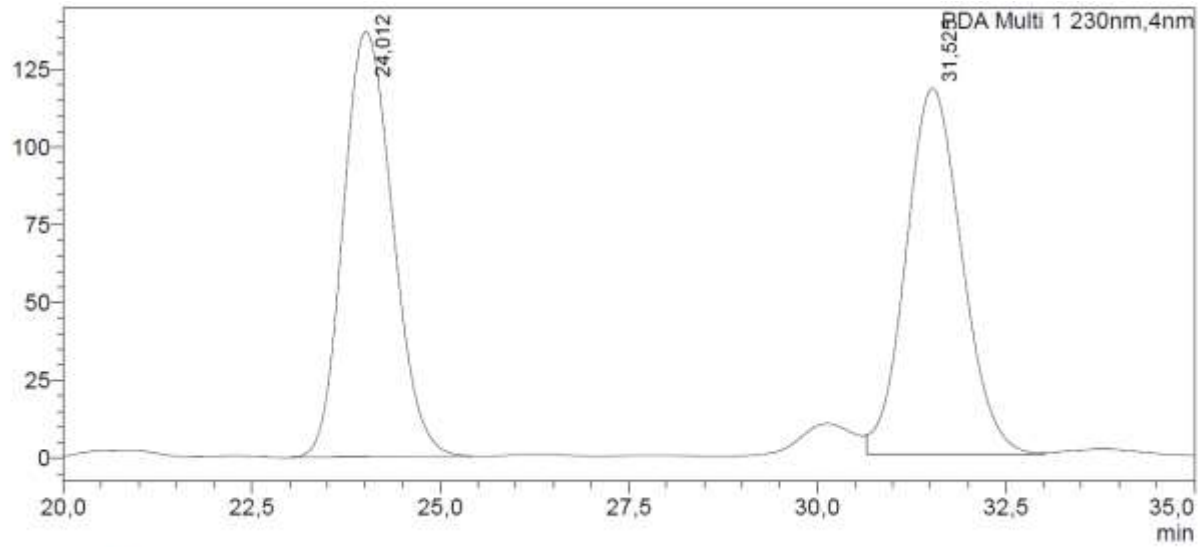

<Peak Table>

PDA Ch1 230nm

Peak#	Ret. Time	Area	Height	Area%
1	24,012	6228722	136558	50,740
2	31,525	6047039	117373	49,260
Total		12275761	253931	100,000

Supplementary Figure 108. HPLC spectra of products **6m.**

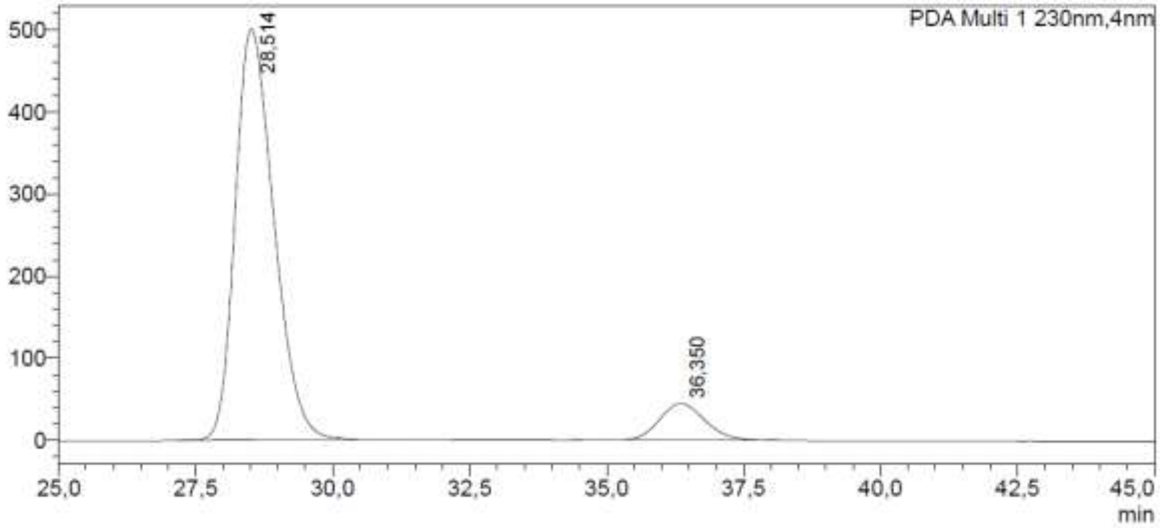

<Peak Table>

PDA Ch1 230nm

Peak#	Ret. Time	Area	Height	Area%
1	28,514	24518373	500339	90,840
2	36,350	2472316	43826	9,160
Total		26990689	544165	100,000

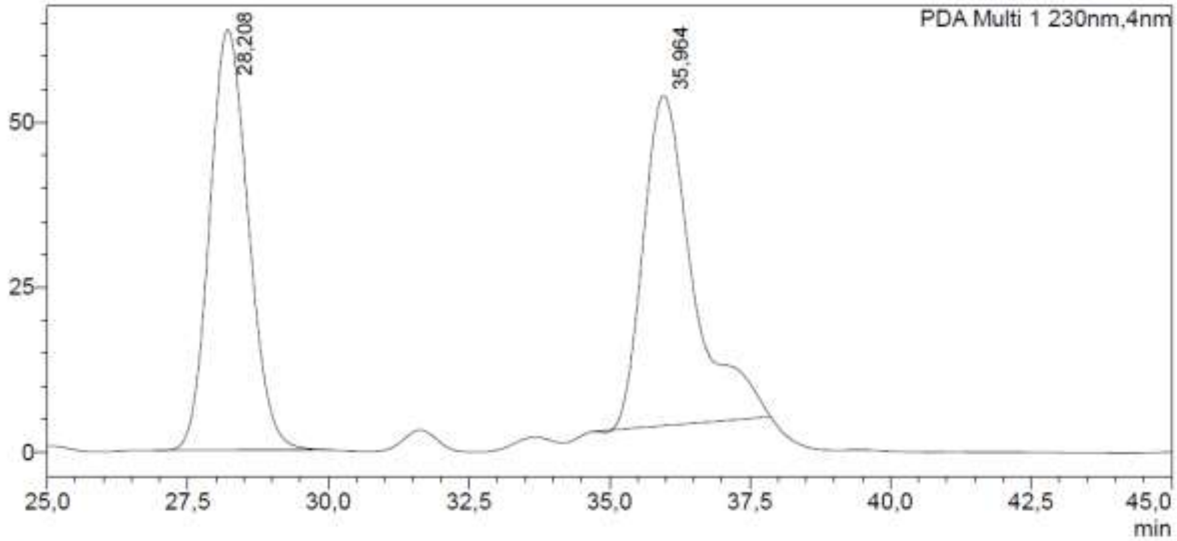

<Peak Table>

PDA Ch1 230nm

Peak#	Ret. Time	Area	Height	Area%
1	28,208	3056421	63746	49,854
2	35,964	3074355	49991	50,146
Total		6130776	113737	100,000

Supplementary Figure 109. HPLC spectra of products **6n**

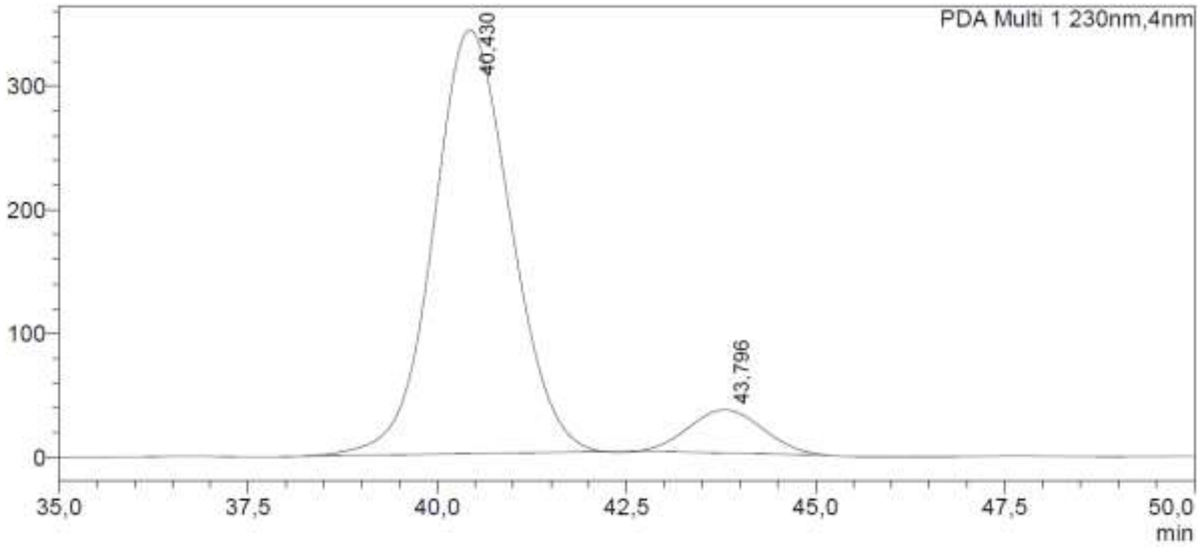

<Peak Table>

PDA Ch1 230nm

Peak#	Ret. Time	Area	Height	Area%
1	40,430	24307031	342204	91,043
2	43,796	2391516	34860	8,957
Total		26698548	377063	100,000

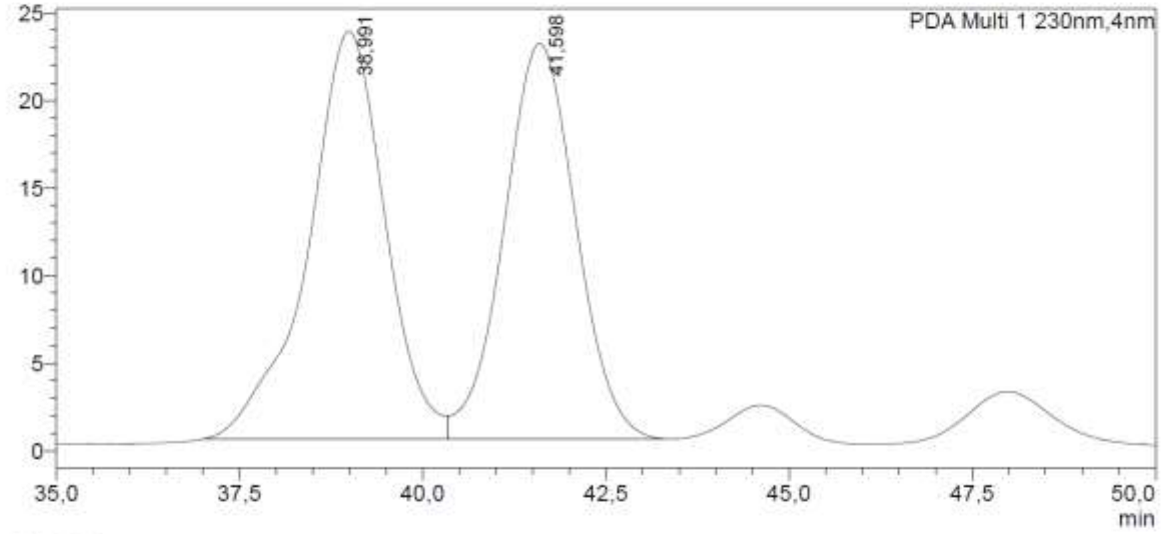

<Peak Table>

PDA Ch1 230nm

Peak#	Ret. Time	Area	Height	Area%
1	38,991	1701634	23237	52,489
2	41,598	1540228	22557	47,511
Total		3241862	45793	100,000

Supplementary Figure 110. HPLC spectra of products **6o**.

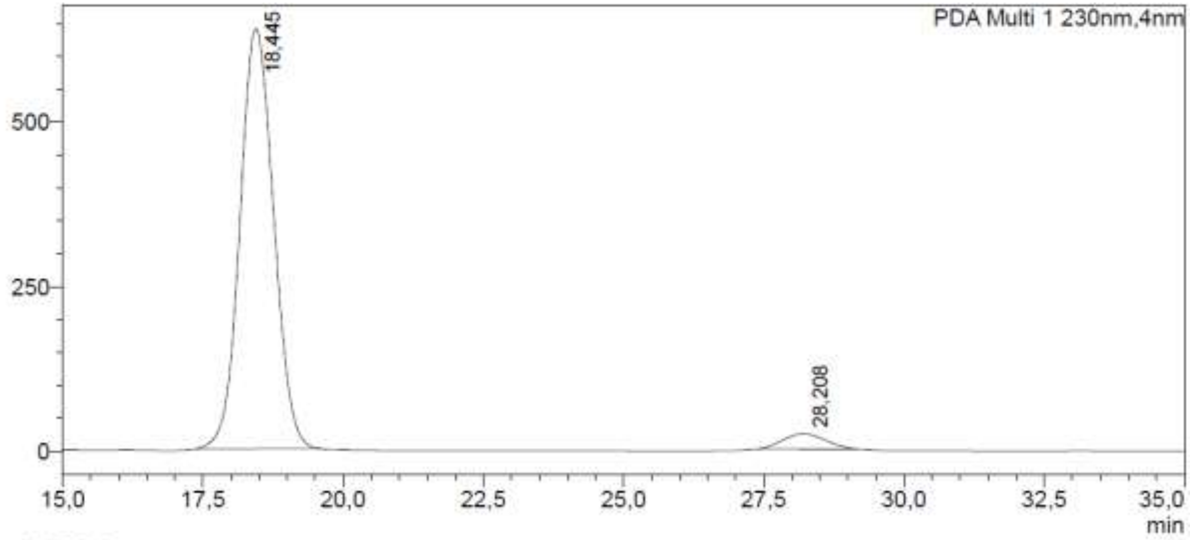

<Peak Table>

PDA Ch1 230nm

Peak#	Ret. Time	Area	Height	Area%
1	18,445	26633984	638280	95,691
2	28,208	1199327	22955	4,309
Total		27833311	661235	100,000

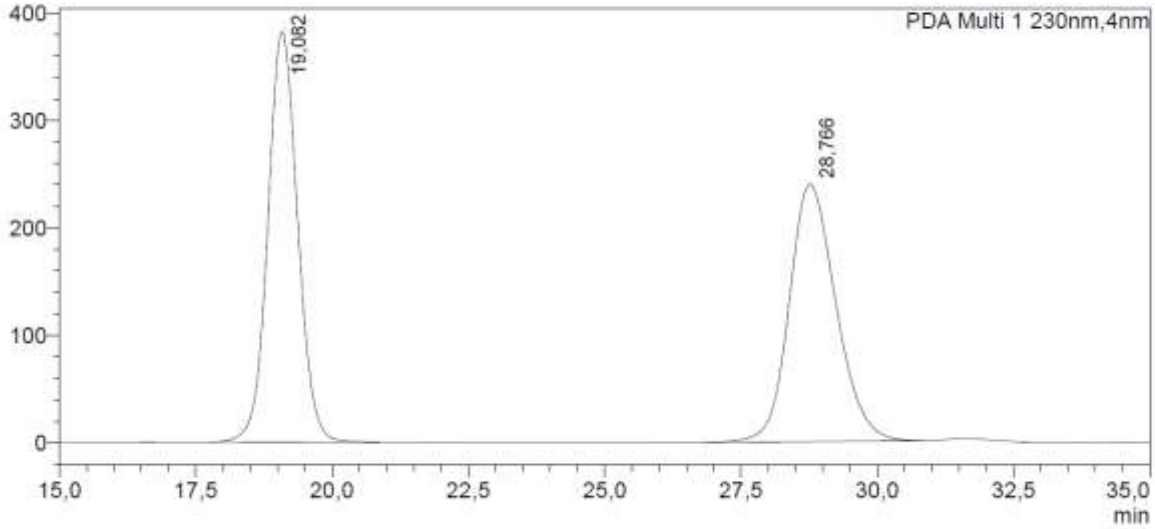

<Peak Table>

PDA Ch1 230nm

Peak#	Ret. Time	Area	Height	Area%
1	19,082	14484671	381921	50,358
2	28,766	14278503	239372	49,642
Total		28763174	621293	100,000

Supplementary Figure 111. HPLC spectra of products **6p**.

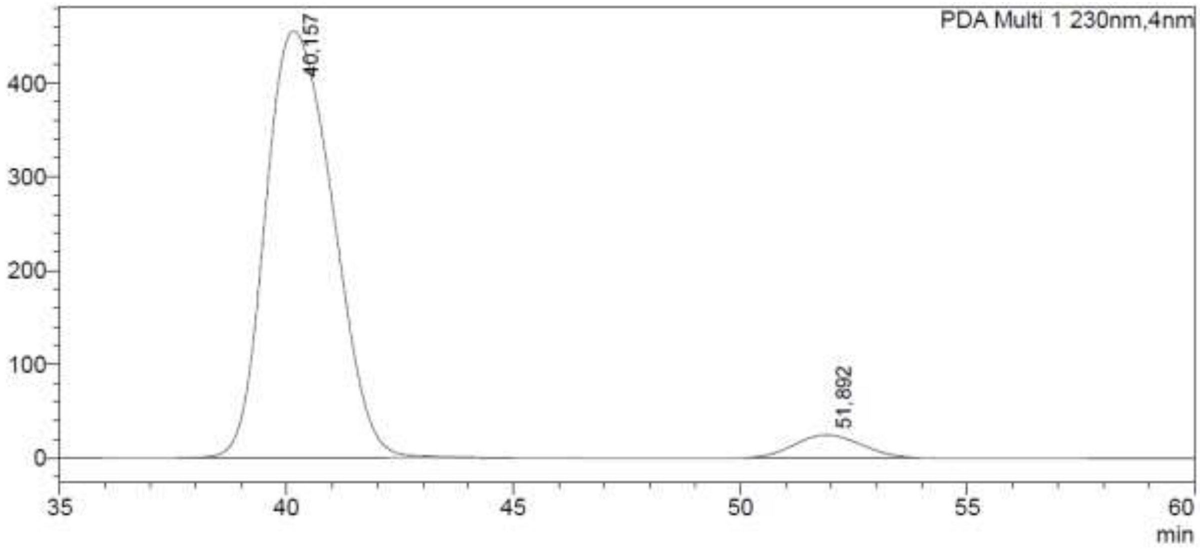

<Peak Table>

PDA Ch1 230nm

Peak#	Ret. Time	Area	Height	Area%
1	40,157	46742747	454801	95,070
2	51,892	2424001	23945	4,930
Total		49166747	478746	100,000

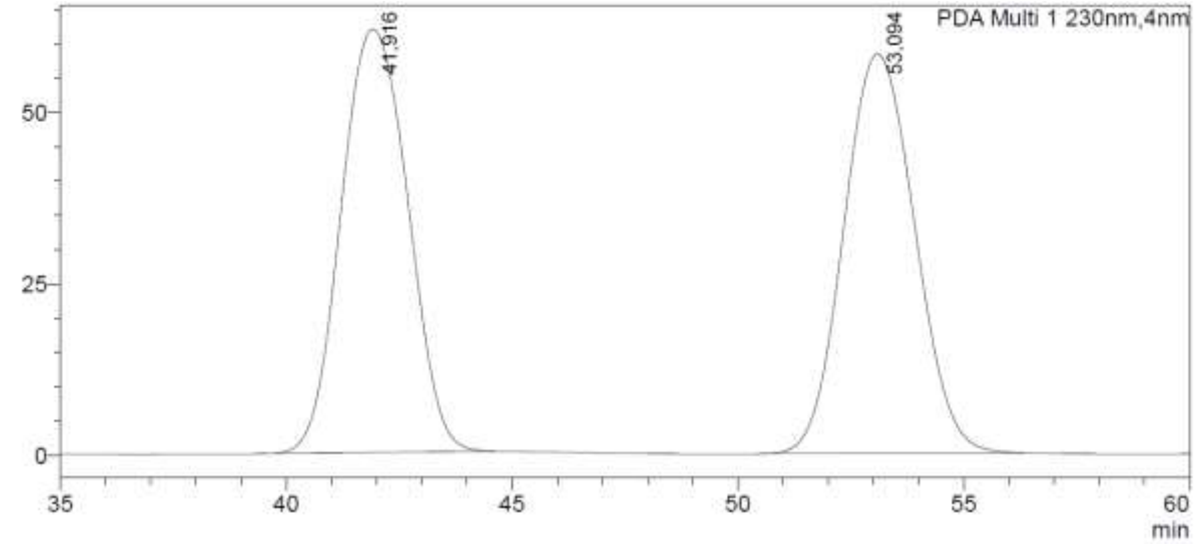

<Peak Table>

PDA Ch1 230nm

Peak#	Ret. Time	Area	Height	Area%
1	41,916	6344380	61730	49,937
2	53,094	6360415	58232	50,063
Total		12704795	119962	100,000

Supplementary Figure 112. HPLC spectra of products **6q**.

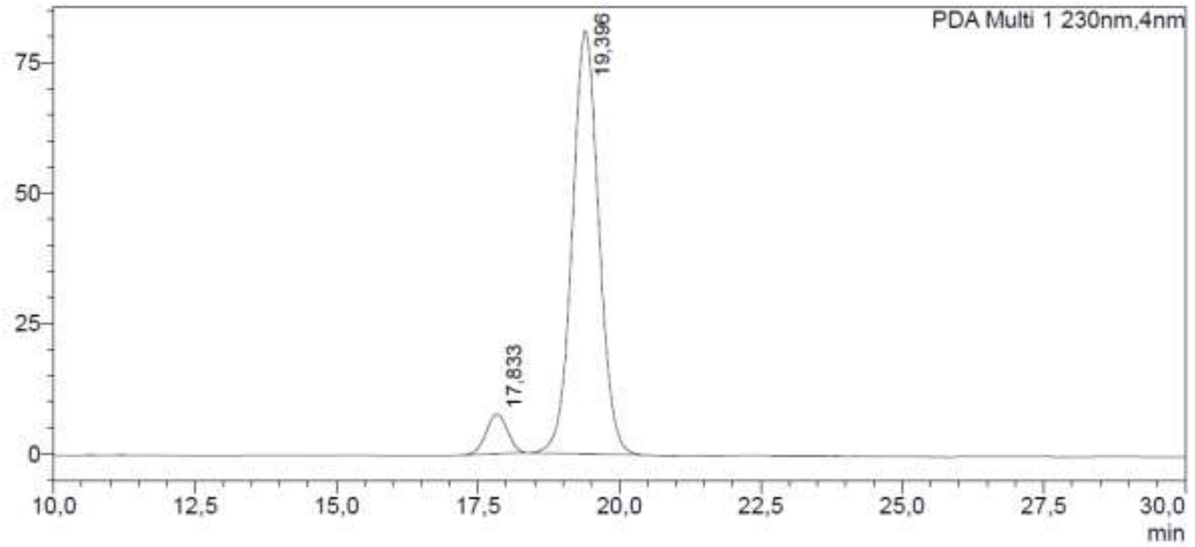

<Peak Table>

PDA Ch1 230nm

Peak#	Ret. Time	Area	Height	Area%
1	17,833	199911	7630	7,010
2	19,396	2651905	81121	92,990
Total		2851816	88751	100,000

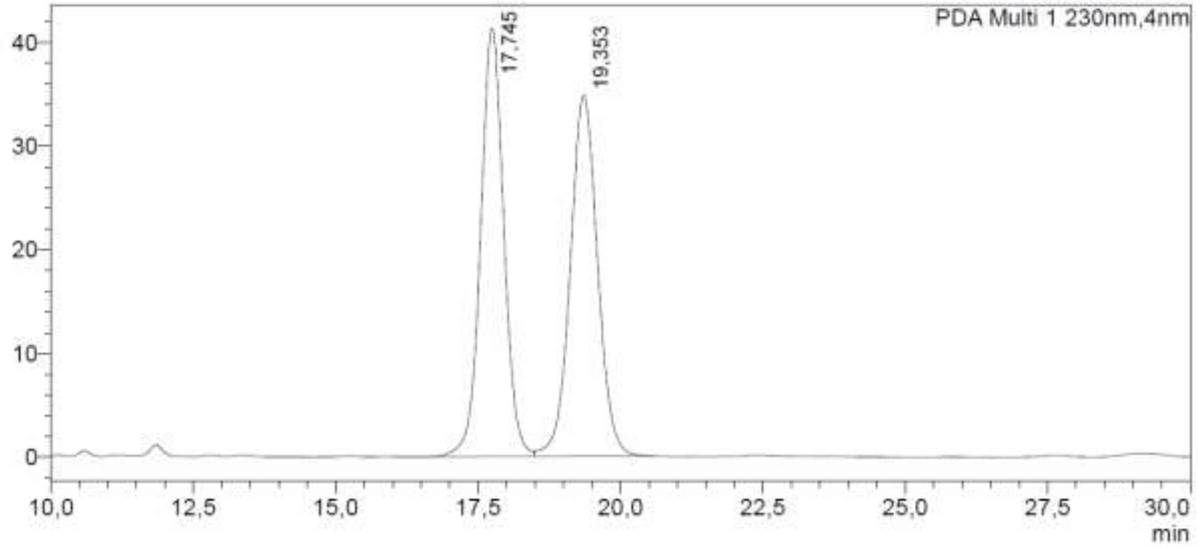

<Peak Table>

PDA Ch1 230nm

Peak#	Ret. Time	Area	Height	Area%
1	17,745	1166108	41192	50,102
2	19,353	1161342	34756	49,898
Total		2327450	75949	100,000

Supplementary Figure 113. HPLC spectra of products **6r**.

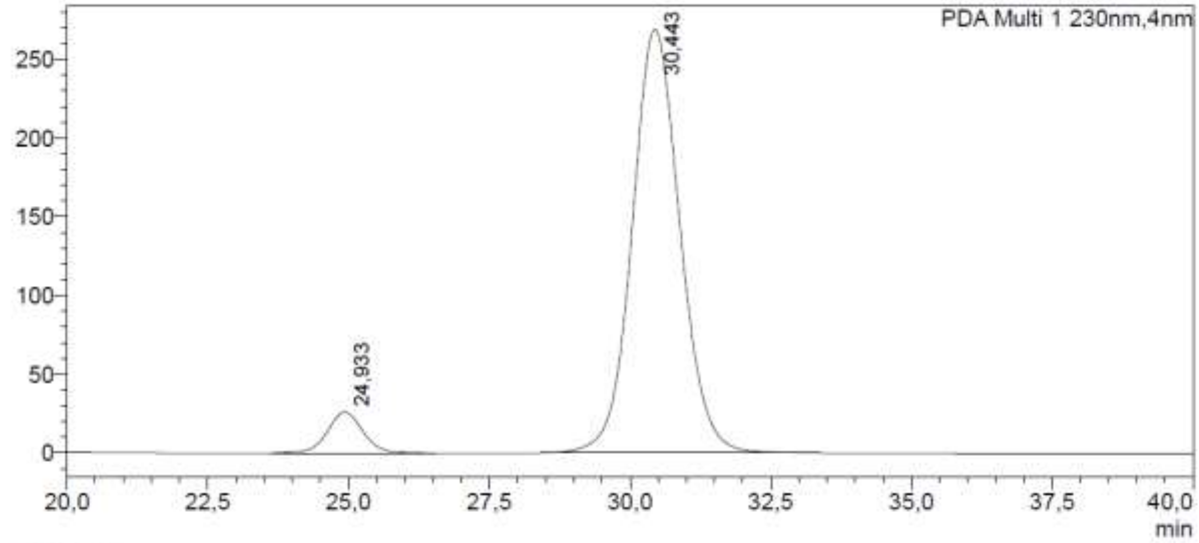

<Peak Table>

PDA Ch1 230nm

Peak#	Ret. Time	Area	Height	Area%
1	24,933	1140639	25970	6,684
2	30,443	15925138	269098	93,316
Total		17065777	295067	100,000

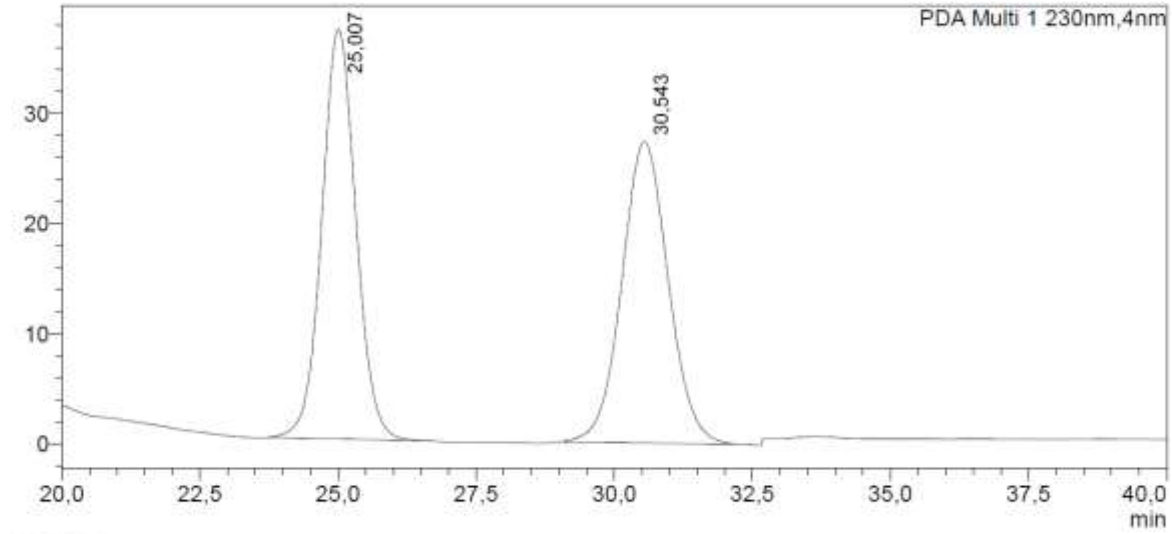

<Peak Table>

PDA Ch1 230nm

Peak#	Ret. Time	Area	Height	Area%
1	25,007	1599548	37222	49,940
2	30,543	1603395	27381	50,060
Total		3202943	64603	100,000

Supplementary Figure 114. HPLC spectra of products **6s**.

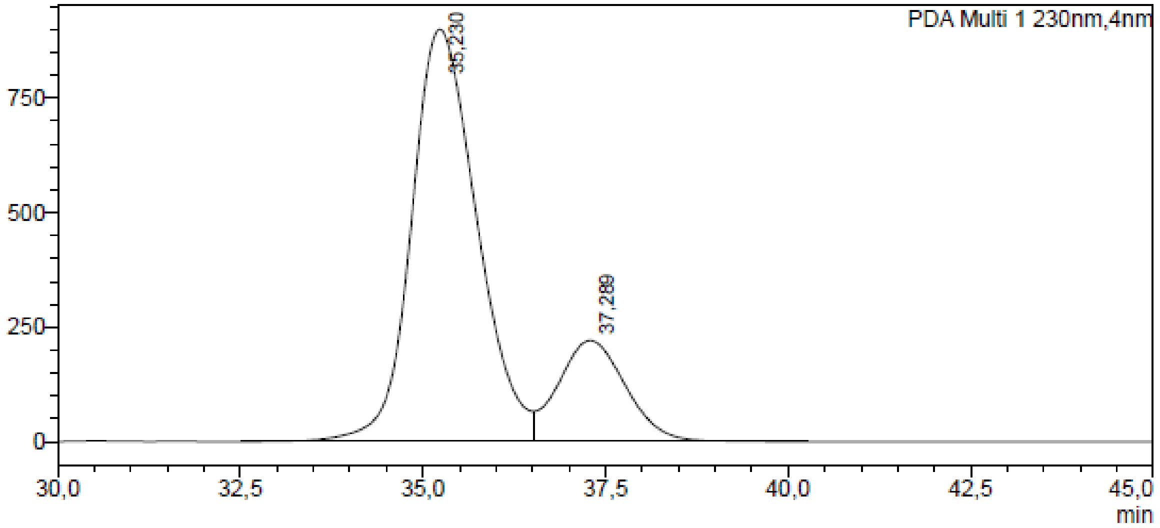

<Peak Table>

PDA Ch1 230nm

Peak#	Ret. Time	Area	Height	Area%
1	35,230	55999894	899376	79,809
2	37,289	14167204	220379	20,191
Total		70167098	1119755	100,000

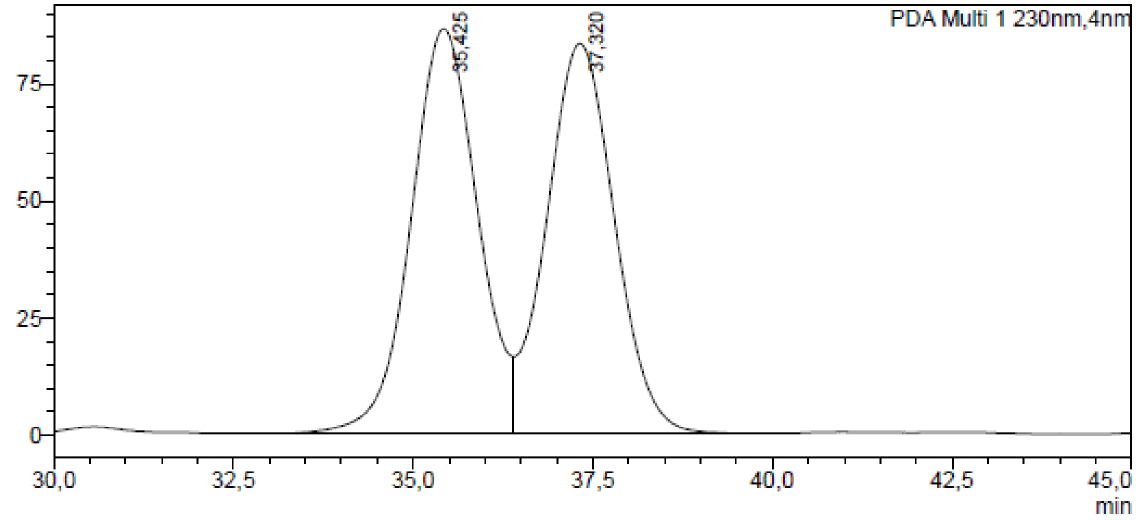

<Peak Table>

PDA Ch1 230nm

Peak#	Ret. Time	Area	Height	Area%
1	35,425	5550413	86323	50,380
2	37,320	5466696	83180	49,620
Total		11017109	169503	100,000

Supplementary Figure 115. HPLC spectra of products **6t**

.

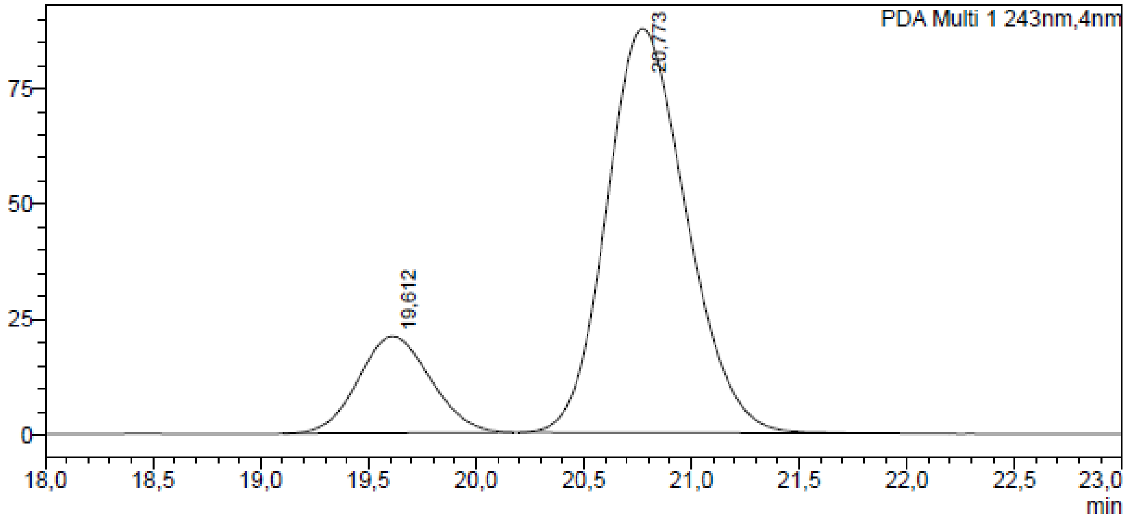

<Peak Table>

PDA Ch1 243nm

Peak#	Ret. Time	Area	Height	Conc.	Area%
1	19,612	490152	20936	17,826	17,826
2	20,773	2259492	87550	82,174	82,174
Total		2749644	108486		100,000

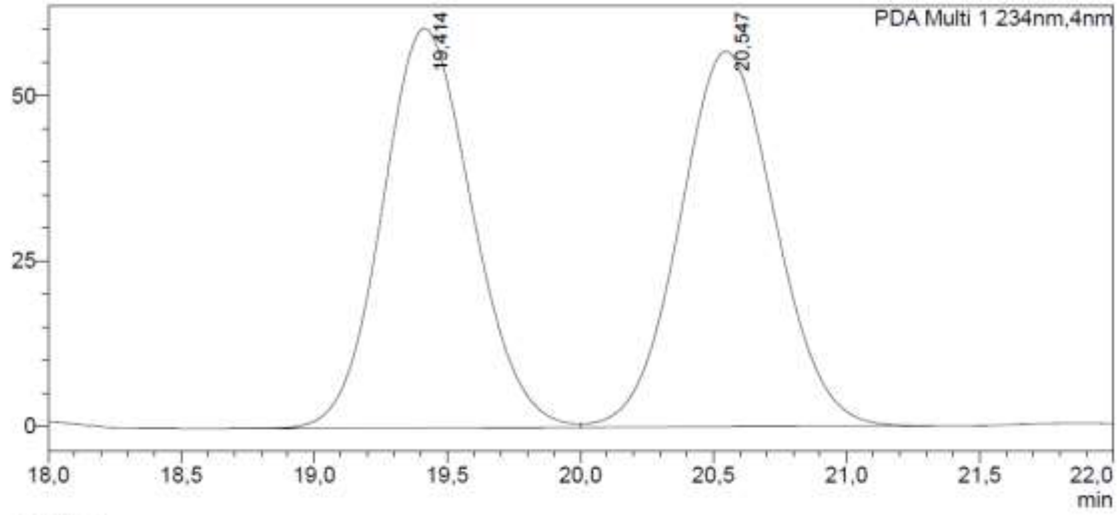

<Peak Table>

PDA Ch1 234nm

Peak#	Ret. Time	Area	Height	Conc.	Area%
1	19,414	1419692	60436	50,005	50,005
2	20,547	1419386	56763	49,995	49,995
Total		2839079	117198		100,000

Supplementary Figure 116. HPLC spectra of products **7a**.

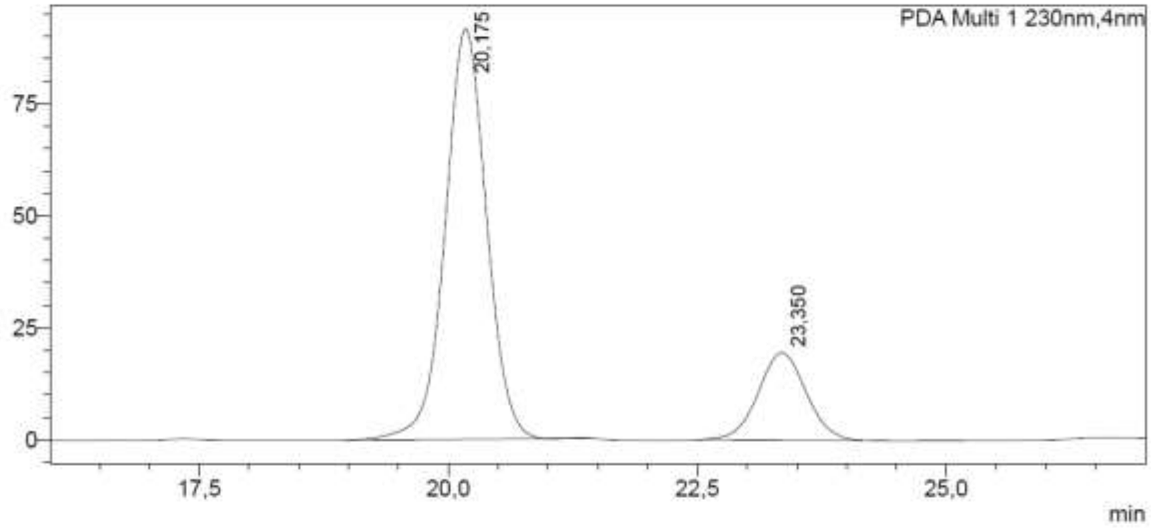

<Peak Table>

PDA Ch1 230nm

Peak#	Ret. Time	Area	Height	Area%
1	20,175	2652097	91592	80,319
2	23,350	649856	19398	19,681
Total		3301953	110990	100,000

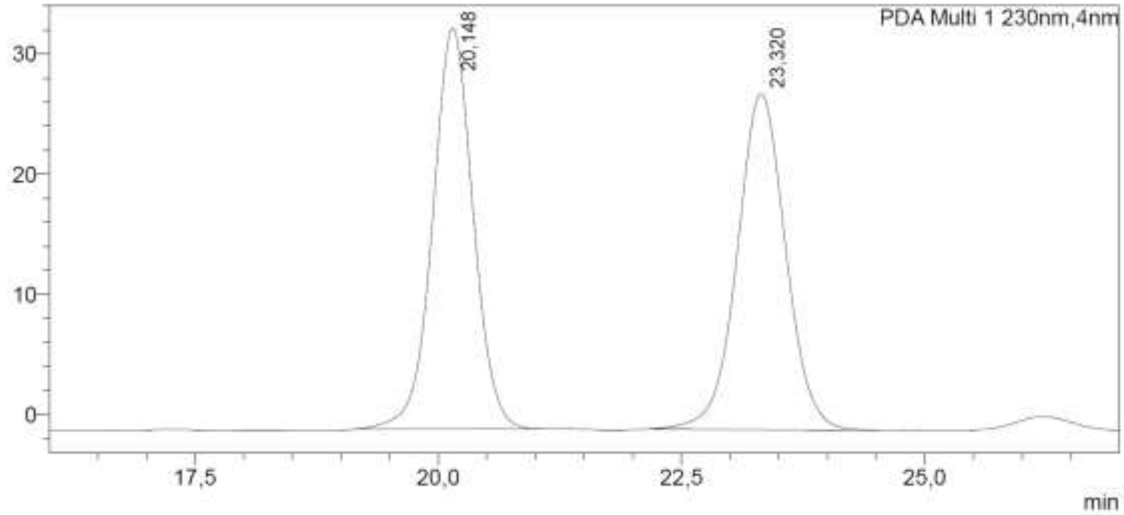

<Peak Table>

PDA Ch1 230nm

Peak#	Ret. Time	Area	Height	Area%
1	20,148	968225	33346	49,945
2	23,320	970346	27948	50,055
Total		1938571	61294	100,000

Mother liquor after recrystallization

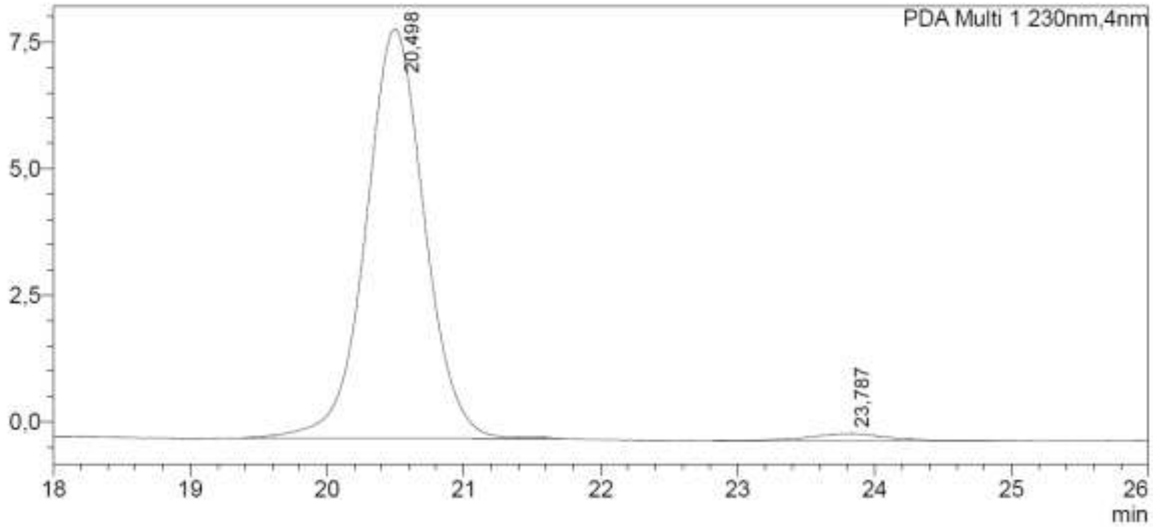

<Peak Table>

PDA Ch1 230nm

Peak#	Ret. Time	Area	Height	Area%
1	20,498	241138	8076	97,971
2	23,787	4994	135	2,029
Total		246132	8211	100,000

Crystals after recrystallization

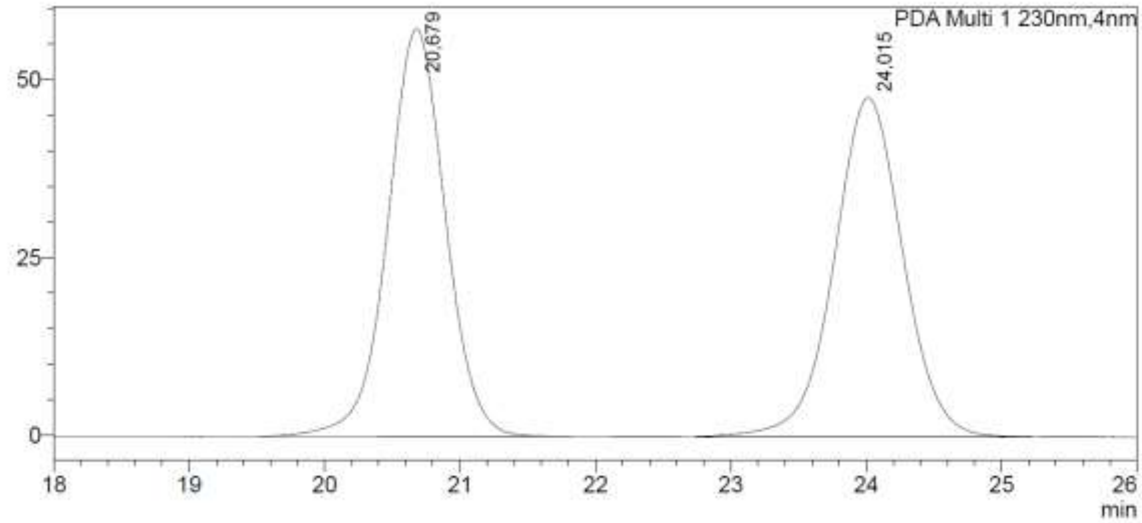

<Peak Table>

PDA Ch1 230nm

Peak#	Ret. Time	Area	Height	Area%
1	20,679	1697524	57246	50,180
2	24,015	1685372	47625	49,820
Total		3382896	104871	100,000

Supplementary Figure 117. HPLC spectra of products **7b**.

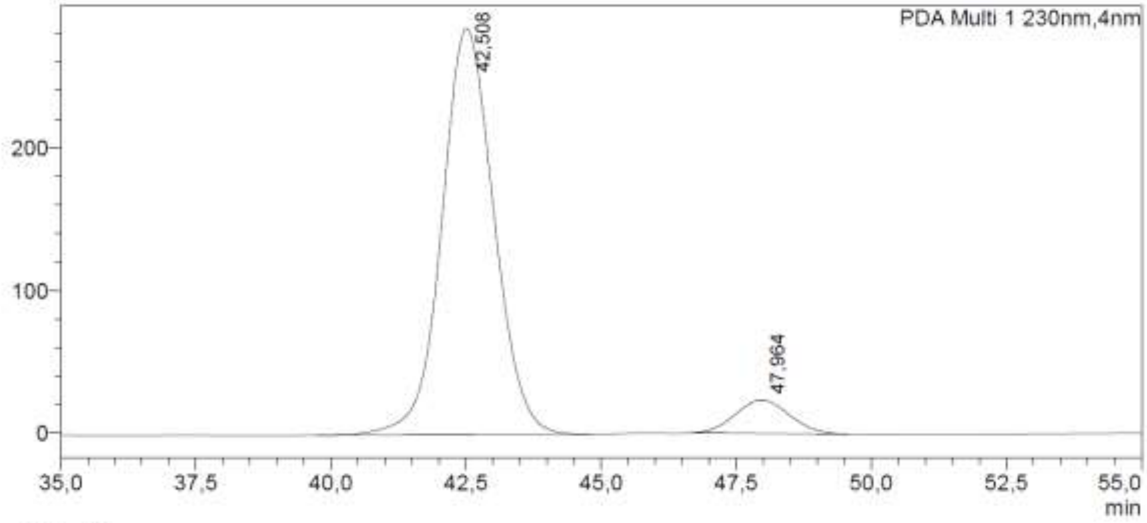

<Peak Table>

PDA Ch1 230nm

Peak#	Ret. Time	Area	Height	Area%
1	42,508	19084462	284225	92,100
2	47,964	1636993	23480	7,900
Total		20721455	307705	100,000

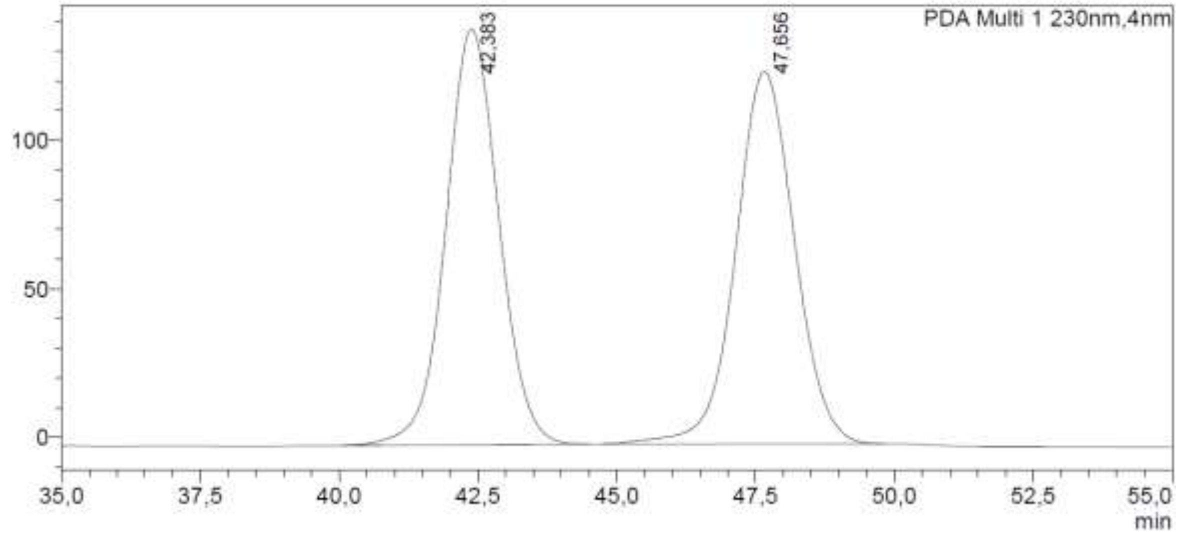

<Peak Table>

PDA Ch1 230nm

Peak#	Ret. Time	Area	Height	Area%
1	42,383	9317399	139956	50,152
2	47,656	9260889	125306	49,848
Total		18578287	265262	100,000

Supplementary Figure118. HPLC spectra of products **7c**.

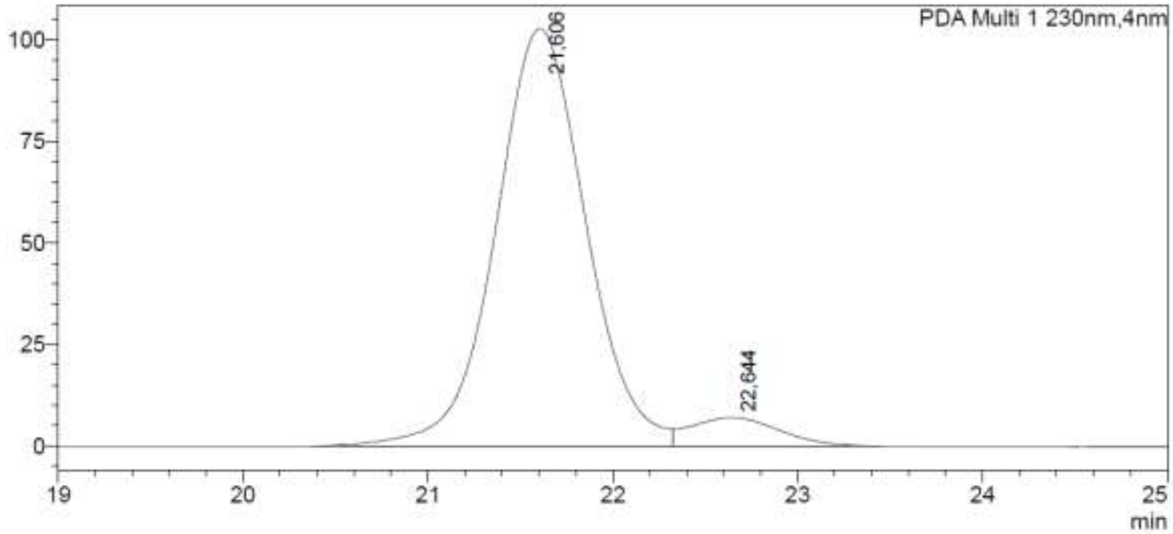

<Peak Table>

PDA Ch1 230nm

Peak#	Ret. Time	Area	Height	Area%
1	21,606	3485931	102731	93,697
2	22,644	234491	7034	6,303
Total		3720422	109766	100,000

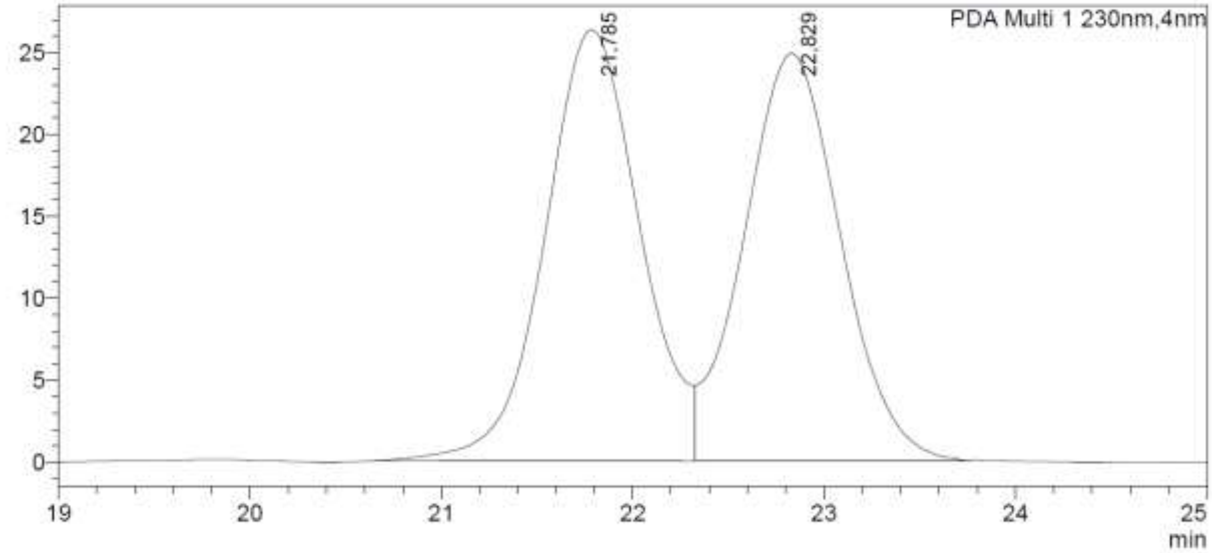

<Peak Table>

PDA Ch1 230nm

Peak#	Ret. Time	Area	Height	Area%
1	21,785	907096	26320	50,840
2	22,829	877107	24895	49,160
Total		1784202	51215	100,000

Supplementary Figure 119. HPLC spectra of products **7d**.

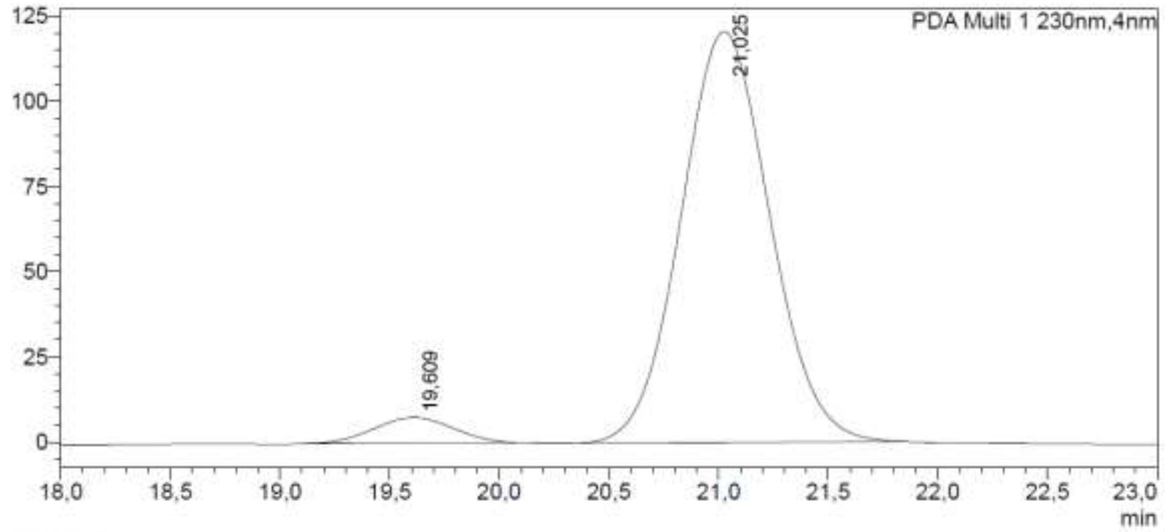

<Peak Table>

PDA Ch1 230nm

Peak#	Ret. Time	Area	Height	Area%
1	19,609	191260	7541	5,323
2	21,025	3401805	120481	94,677
Total		3593065	128022	100,000

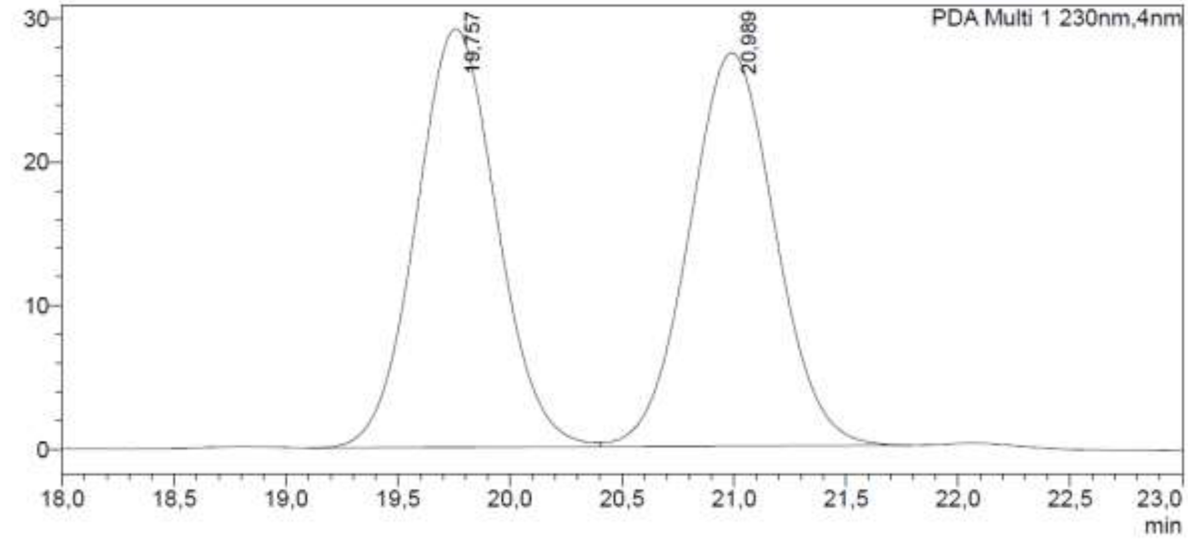

<Peak Table>

PDA Ch1 230nm

Peak#	Ret. Time	Area	Height	Area%
1	19,757	731579	29129	49,999
2	20,989	731610	27367	50,001
Total		1463189	56496	100,000

Supplementary Figure 120. HPLC spectra of products **7e**.

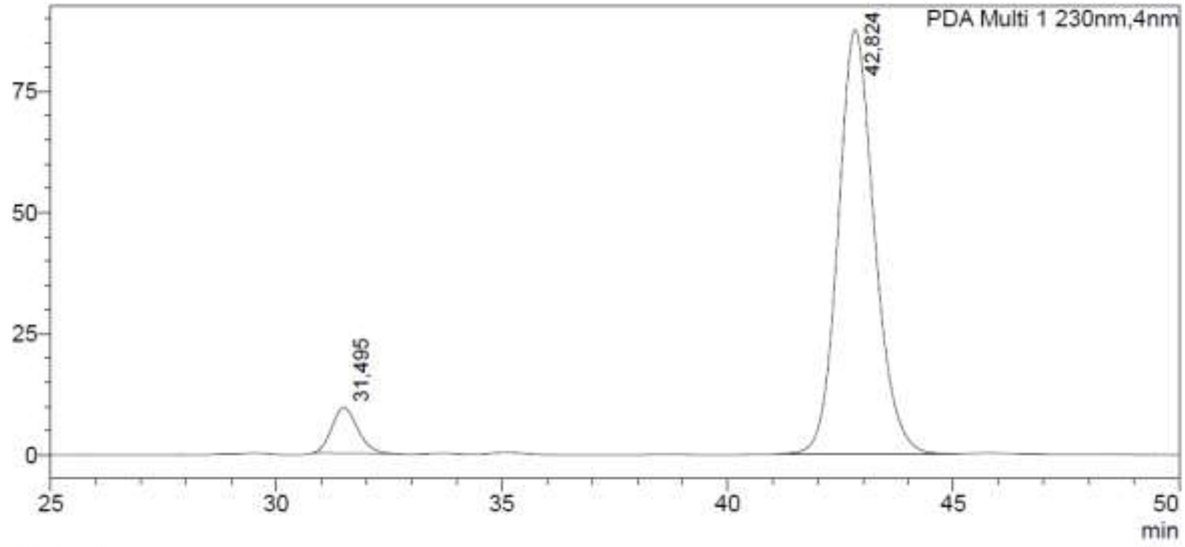

<Peak Table>

PDA Ch1 230nm

Peak#	Ret. Time	Area	Height	Area%
1	31,495	378129	9415	7,166
2	42,824	4898545	87487	92,834
Total		5276674	96902	100,000

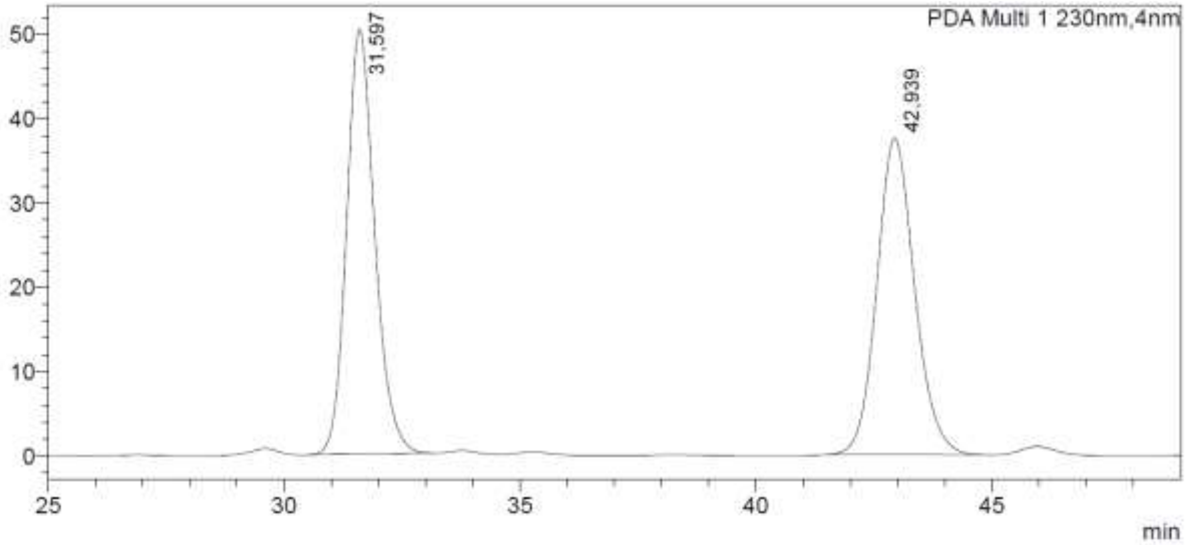

<Peak Table>

PDA Ch1 230nm

Peak#	Ret. Time	Area	Height	Area%
1	31,597	2095398	50398	50,091
2	42,939	2087767	37508	49,909
Total		4183165	87906	100,000

Supplementary Figure 121. HPLC spectra of products **7f**.

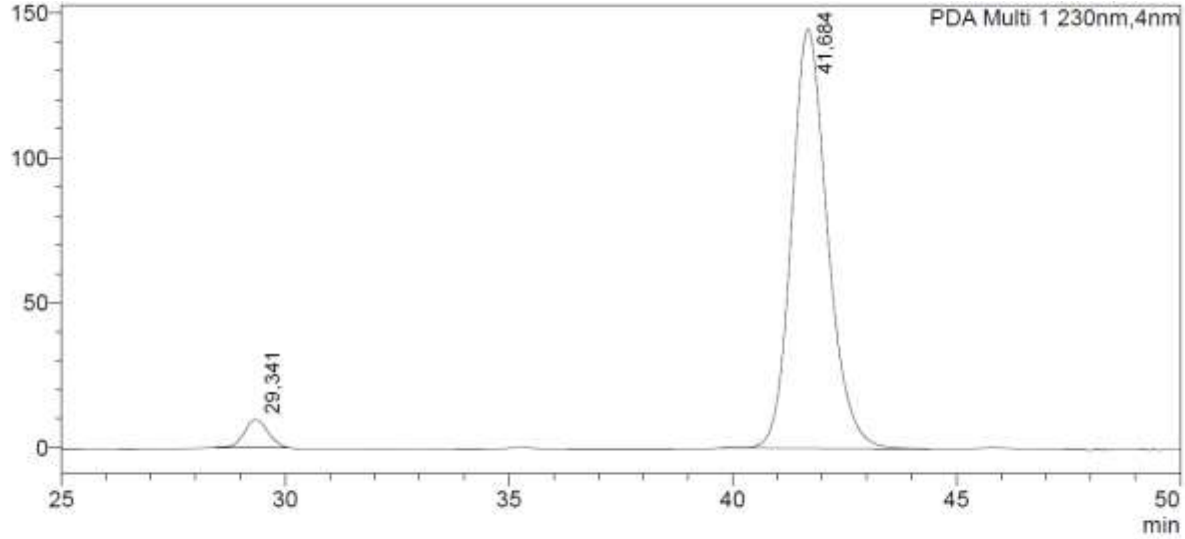

<Peak Table>

PDA Ch1 230nm

Peak#	Ret. Time	Area	Height	Area%
1	29,341	346533	9582	4,114
2	41,684	8076707	144859	95,886
Total		8423241	154441	100,000

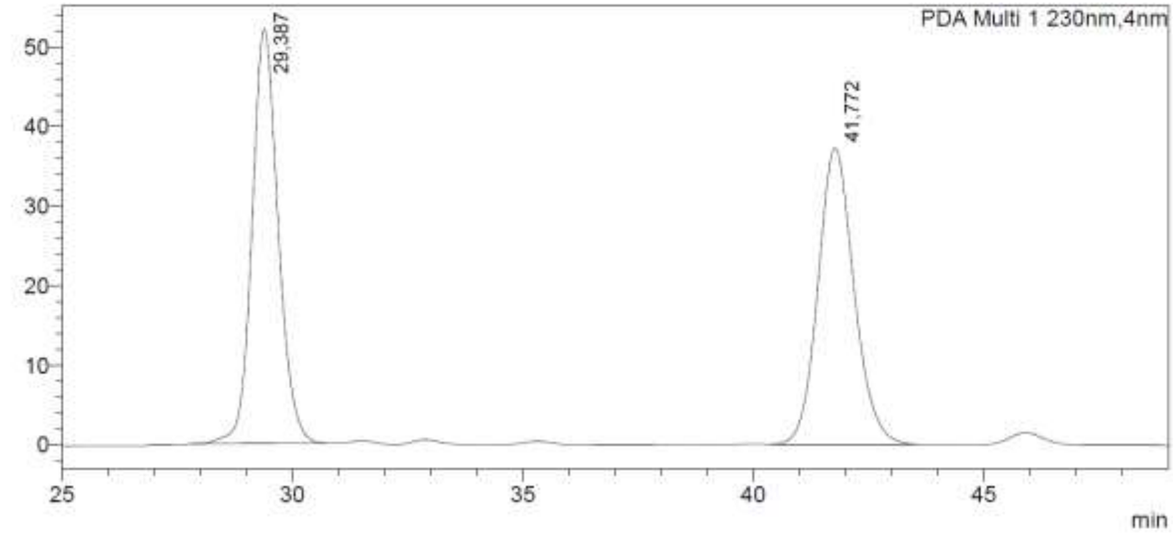

<Peak Table>

PDA Ch1 230nm

Peak#	Ret. Time	Area	Height	Area%
1	29,387	2056440	52092	50,425
2	41,772	2021806	37227	49,575
Total		4078247	89319	100,000

Supplementary Figure 122. HPLC spectra of products **7g**.

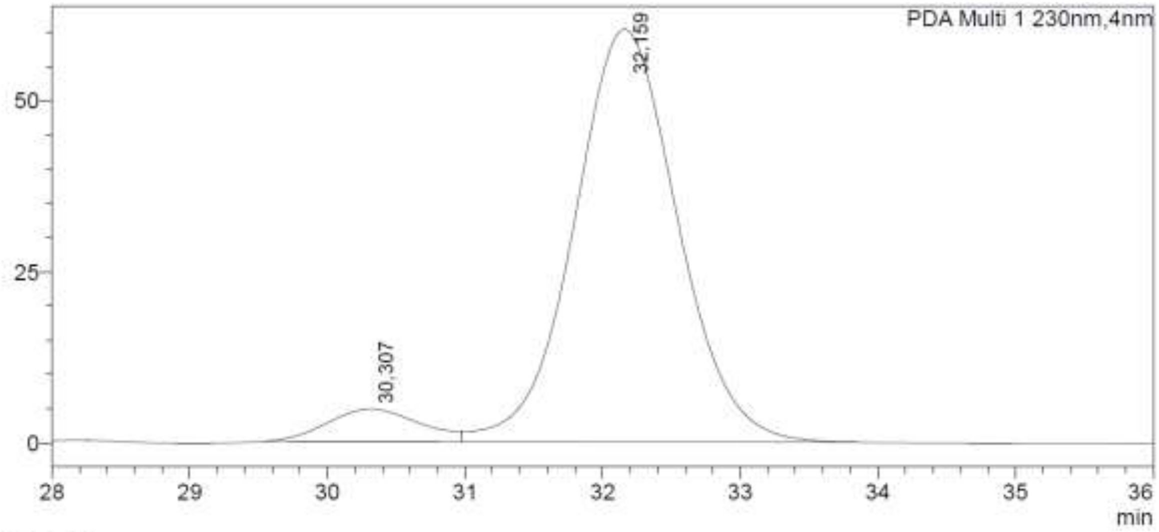

<Peak Table>

PDA Ch1 230nm

Peak#	Ret. Time	Area	Height	Area%
1	30,307	221475	4749	6,564
2	32,159	3152839	60319	93,436
Total		3374315	65068	100,000

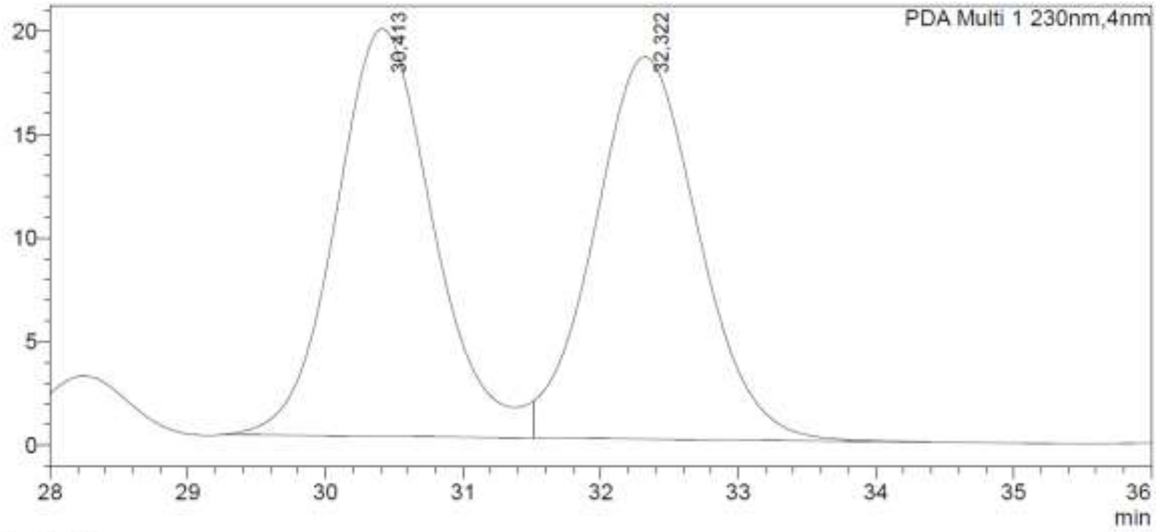

<Peak Table>

PDA Ch1 230nm

Peak#	Ret. Time	Area	Height	Area%
1	30,413	969368	19650	49,944
2	32,322	971533	18441	50,056
Total		1940901	38091	100,000

Supplementary Figure 123. HPLC spectra of products **7h**.

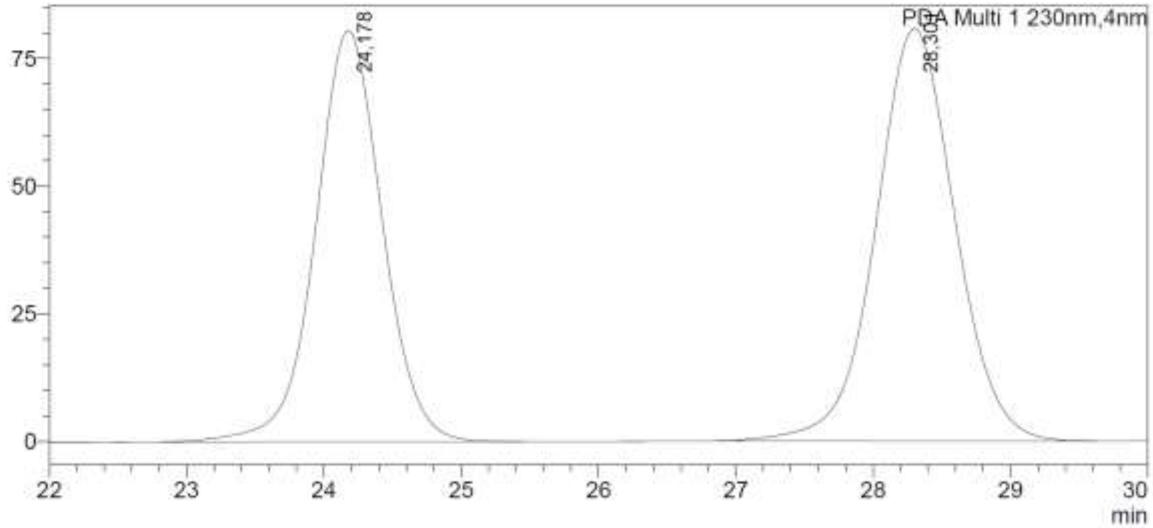

<Peak Table>

PDA Ch1 230nm

Peak#	Ret. Time	Area	Height	Area%
1	24,178	2723438	80525	45,687
2	28,301	3237635	80839	54,313
Total		5961073	161364	100,000

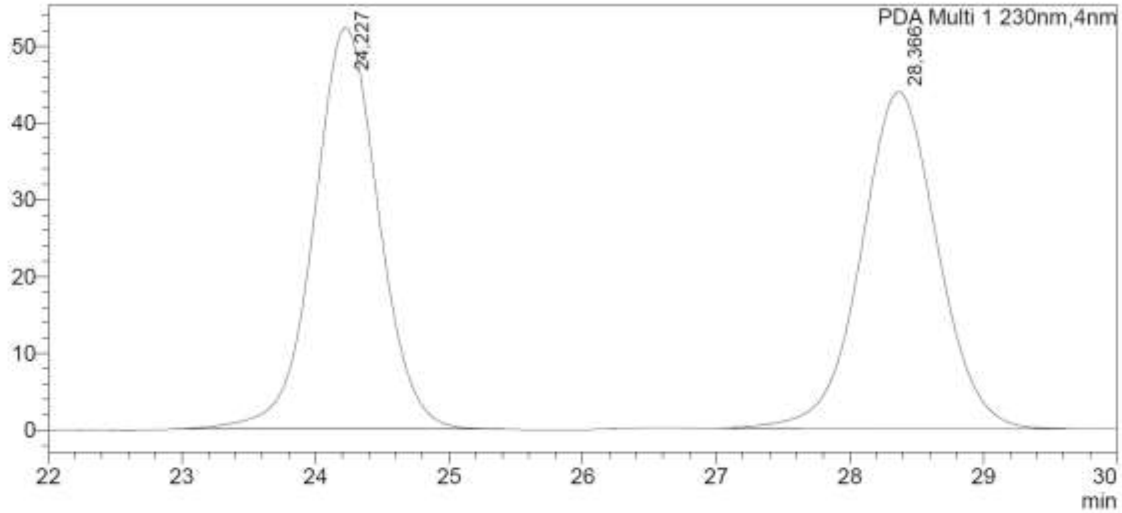

<Peak Table>

PDA Ch1 230nm

Peak#	Ret. Time	Area	Height	Area%
1	24,227	1769548	52356	50,242
2	28,366	1752484	43891	49,758
Total		3522032	96247	100,000

Supplementary Table 1. Crystallographic data for racemic addition product **7a** (left) and ketimine **1g** (right, one of the two independent molecules shown).

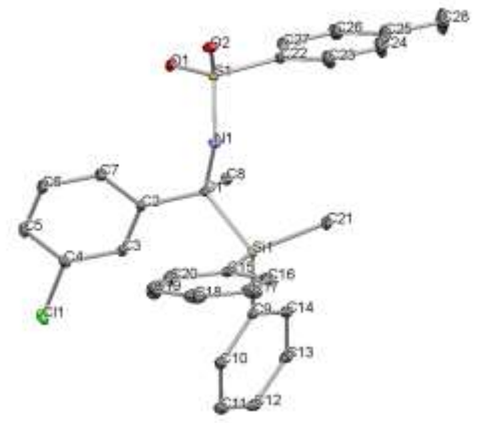

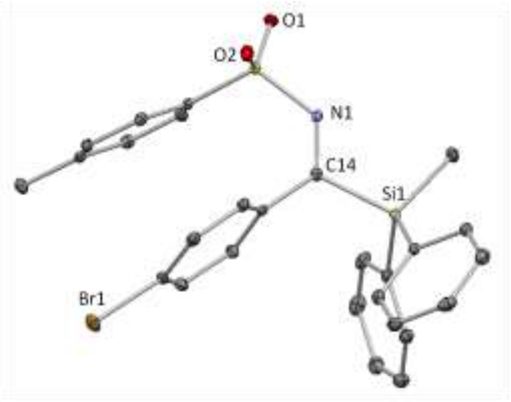

	**7a**	**1g**
chem formula	C28 H28 Cl N O2 S Si	C27 H24 Br N O2 S Si
M_r	506.11	534.53
cryst syst	triclinic	triclinic
color, habit	colorless, needle	colorless, block
size (mm)	0.48 x 0.13 x 0.10	0.41 x 0.24 x 0.18
space group	P-1	P-1
a (Å)	10.7995(8)	9.7694(19)
b (Å)	11.2355(9)	11.256(2)
c (Å)	11.7161(9)	25.107(5)
α, deg	83.678(3)	89.912(8)
β, deg	68.796(3)	83.012(8)
γ, deg	74.110(3)	65.568(7)
V (Å^3)	1274.62(17)	2491.4(9)
Z	2	4
ρ_{calc}, g.cm^{-3}	1.319	1.425
μ(Mo K$\bar{\alpha}$), cm^{-1}	0.305	1.807
F(000)	532	1096
temp (K)	100(2)	100(2)
θ range (deg)	3.149 – 28.347	2.967 – 27.286
data collected (h,k,l)	-14:14, -15:15, -15:15	-12:12, -14:14, -32:32

no. of rflns collected	34812	99451
no. of indpndt reflns	6343	11120
observed reflns	5575 ($F_o \geq 2\ \sigma(F_o)$)	9880
R(F) (%)	3.04	2.85
wR(F^2) (%)	7.94	7.02
GooF	1.053	1.087
Weighting a,b	0.0355, 0.7422	0.0299, 1.7688
params refined	310	599
restraints	0	0
min, max resid dens	-0.410, 0.377	-0.514, 0.404

Single crystals of compounds **7a** and **1g** were mounted on top of a cryoloop and transferred into the cold nitrogen stream (100 K) of a Bruker-AXS D8 Venture diffractometer. Data collection and reduction was done using the Bruker software suite APEX2.[1] The final unit cell was obtained from the xyz centroids of 9516 (**7a**) and 9590 (**1g**) reflections after integration. A multiscan absorption correction was applied, based on the intensities of symmetry-related reflections measured at different angular settings (*SADABS*). The structures were solved by direct methods using *SHELXT* (**7a**)[2] or *SHELXS* (**1g**)[1] and refinement of the structures was performed using *SHLELXL*.[3] The hydrogen atoms were generated by geometrical considerations, constrained to idealised geometries and allowed to ride on their carrier atoms with an isotropic displacement parameter related to the equivalent displacement parameter of their carrier atoms.

Supplementary discussions:

Configuration of silyl ketimines:

All the ketimines prepared for this work were obtained with more than 95% configurational purity. According to the literature, ketimines derived from aryl alkyl ketones are typically obtained as a mixture of *E* and *Z* isomers, with the former being the major isomer where the *N*-protecting group is positioned opposite to the larger phenyl substituent. In the case of aryl silyl ketimines, both substituents at the carbon atom of the ketimine moiety are relatively bulky and the C-Si bond is longer than the C-C bond. Thus, it was not trivial to predict *a priori* which isomer would be obtained. Since our attempts to determine the configuration of the silyl ketimine **1g** by NMR spectroscopy were not successful, we determined the configuration of the ketimine **1g** shown in Supplementary Table 1 by single-crystal X-ray diffraction. The unit cell of **1g** contains two independent molecules, which both have *E*-configuration with the *N*-tosyl protecting group on the same side as the aryl group and both aromatic rings relatively coplanar with each other (17.83 and 23.99° _for the two independent molecules). The distance between both centroids is 3.694 and 3.589 Å, respectively. These metrical parameters could be indicative of a weak attractive interaction between the aromatic rings that contributes to stabilizing the *E*-isomer. Furthermore, the bulkiness of the $SiPh_2Me$ moiety compared to the aryl group, combined with the possible hyper-conjugation of the *Si-C* bond with the anti-bonding orbital of the *N-S* bond, could be responsible for the stabilisation of the *E*-configuration. It is worth noting that the aryl group is rotated 75-77°out of the plane of the imine moiety. This disruption of the efficient conjugation between the aryl and imine moieties, caused by the loss of planarity, might affect the reactivity of the ketimine substrates. All the imines were assigned *E*-configuration by analogy with single-X-ray diffraction data obtained for **1g** (Crystal data and details on data collection and refinement are presented in Supplementary Table 1).

Supplementary methods:

General information:

All reactions using oxygen- and/or moisture-sensitive materials were carried out with anhydrous solvents (vide infra) under a nitrogen atmosphere using oven dried glassware and standard Schlenk techniques. Analytical thin-layer chromatography was performed on pre-coated glass-

backed plates (Silica Gel 60 F254; Merck), and visualized using UV light (254 nm). Flash column chromatography was carried out using Merck silica gel 60, 0.25 mm. Cooling of reaction mixtures to -78 °C was achieved using a Julabo FT902 immersion cooler. NMR spectra were recorded at room temperature; Mass spectra were recorded on an AEI-MS-902 mass spectrometer (EI+) or a LTQ Orbitrap XL (ESI+). 1H-, 13C- and 19F- NMR spectra were recorded on a Varian AMX400 (400, 100.59 and 376 MHz respectively) using $CDCl_3$ as solvent. Chemical shift values are reported in ppm with the solvent resonance as the internal standard ($CDCl_3$: δ 7.26 for 1H, δ 77.0 for 13C). Data are reported as follows: chemical shifts, multiplicity (s = singlet, d = doublet, t = triplet, q = quartet, br = broad, m = multiplet), coupling constants (Hz), and integration. Optical rotations were measured on a Schmidt + Haensch polarimeter (Polartronic MH8) with a 10 cm cell (c given in g/100 mL). Enantiomeric excesses were determined by HPLC analysis using a Shimadzu LC-10ADVP HPLC equipped with a Shimadzu SPD-M10AVP diode array detector. Crystals of **1g** and **7a** were obtained by crystallization from a diethyl ether solution (See Supplementary Table 1).

Chemicals

Dry MTBE, THF, CH_2Cl_2, Et_2O, PhMe, were collected fresh from a solvent purification system. Unless stated otherwise, commercially available reagents were purchased from Sigma-Aldrich (*n*-HexMgBr and *i*PenMgBr (2 M in Et_2O) and MeMgBr and EtMgBr (3 M in Et_2O)). All other Grignard reagents were prepared from the corresponding alkyl bromides and Mg in Et_2O. Ligands **L1** – **L2** and **L4** – **L7** were purchased from Sigma Aldrich and Solvias. Ligand **L3** was synthesized according to the reported procedure[4].

Characterization and synthesis of compounds

New acylsilanes, 9:

Acylsilane precursors **9** for the synthesis of silyl ketimine were prepared according to the literature procedure[5], analytical data are provided only for new acylsilane molecules (**9d**, **9e**, **9f**, **9g**, **9i**, **9j**, **9p**, **9q**).

(2-fluorophenyl)(methyldiphenylsilyl)methanone, ***9d***

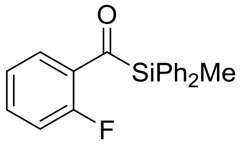

^{1}H NMR (400 MHz, $CDCl_3$) δ 7.65 – 7.53 (m, 5H), 7.49 – 7.34 (m, 7H), 7.22 – 7.15 (m, 1H), 7.13 – 6.95 (m, 1H), 0.87 (d, J = 2.3 Hz, 3H).

^{13}C NMR (101 MHz, $CDCl_3$) δ 231.5, 161.7 (d, $J_{C\text{-}F}$ = 253.8 Hz), 135.1, 135.1 (d, $J_{C\text{-}F}$ = 0.7 Hz), 134.3 (d, $J_{C\text{-}F}$ = 8.6 Hz), 133.3 (d, $J_{C\text{-}F}$ = 2.3 Hz), 130.0, 128.1, 124.6 (d, $J_{C\text{-}F}$ = 3.3 Hz), 116.3 (d, $J_{C\text{-}F}$ = 21.9 Hz), -4.4.

^{19}F NMR (376 MHz, $CDCl_3$) δ -111.7.

HRMS (ESI+, m/Z): calcd for $C_{20}H_{17}FOSiNa$ $[M+Na]^+$: 343.0925; found: 343.0923.

*(4-chlorophenyl)(methyldiphenylsilyl)methanone, **9e***

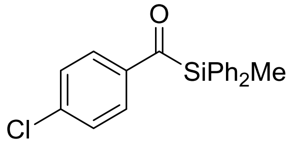

^{1}H NMR (400 MHz, $CDCl_3$) δ 7.70 (d, J = 8.5 Hz,2H), 7.61 – 7.55 (m, 4H), 7.48 – 7.42 (m, 2H), 7.42 – 7.36 (m, 4H), 7.31 (d, J = 8.5 Hz, 2H), 0.86 (s, 3H).

^{13}C NMR (101 MHz, $CDCl_3$) δ 230.7, 140.0, 139.4, 135.2, 133.5, 130.3, 129.7, 129.0, 128.4, -3.3.

HRMS (ESI+, m/Z): calcd for $C_{20}H_{17}ClOSiNa$ $[M+Na]^+$: 359.0629; found:359.0628.

*(3-chlorophenyl)(methyldiphenylsilyl)methanone, **9f***

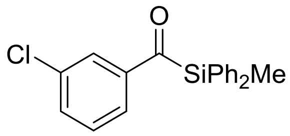

^{1}H NMR (400 MHz, $CDCl_3$) δ 7.73 (t, J = 1.6 Hz, 1H), 7.65 – 7.53 (m, 5H), 7.49 – 7.38 (m, 7H), 7.26 (t, J = 7.8 Hz, 1H), 0.89 (s, 3H).

^{13}C NMR (101 MHz, $CDCl_3$) δ 231.0, 143.1, 135.2, 135.1, 133.3, 132.8, 130.4, 129.9, 128.5, 127.5, 127.1, -3.3.

HRMS (ESI+, m/Z): calcd for $C_{20}H_{17}ClOSiNa$ $[M+Na]^+$: 359.0629; found: 359.0623.

*(3-bromophenyl)(methyldiphenylsilyl)methanone, **9h***

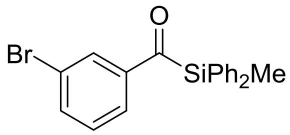

^{1}H NMR (400 MHz, $CDCl_3$) δ 7.86 (t, *J* = 1.6 Hz, 1H), 7.65 (d, *J* = 7.7 Hz, 1H) 7.61 – 7.56 (m, 5H), 7.48 – 7.38 (m, 6H), 7.19 (t, *J* = 7.8 Hz, 1H), 0.87 (s, 3H).

^{13}C NMR (101 MHz, $CDCl_3$) δ 230.9, 143.2, 135.7, 135.2, 133.3, 130.5, 130.4, 130.2, 128.5, 127.5, 123.2, -3.3.

HRMS (ESI+, m/Z): calcd for $C_{20}H_{17}BrOSiNa$ $[M+Na]^+$: 403.0124; found:403.0125.

*(methyldiphenylsilyl)(4-(trifluoromethyl)phenyl)methanone, **9i***

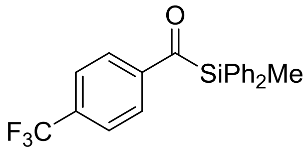

^{1}H NMR (400 MHz, $CDCl_3$) δ 7.84 (d, *J* = 8.1 Hz, 2H), 7.65 – 7.56 (m, 6H), 7.50 – 7.44 (m, 2H), 7.43 – 7.37 (m, 4H), 0.88 (s, 3H).

^{13}C NMR (101 MHz, $CDCl_3$) δ 232.0, 143.8, 135.2, 134.0 (q, $J_{C\text{-}F}$ = 32.5 Hz), 133.1, 130.5, 130.5, 128.5, 128.4, 125.8(q, $J_{C\text{-}F}$ = 3.6 Hz), 123.7(q, $J_{C\text{-}F}$ = 272.8 Hz), -3.4.

^{19}F NMR (376 MHz, $CDCl_3$) δ -63.08.

HRMS (ESI+, m/Z): calcd for $C_{21}H_{17}F_3OSiNa$ $[M+Na]^+$: 393.0893; found: 393.0891.

*(methyldiphenylsilyl)(3-(trifluoromethyl)phenyl)methanone, **9j***

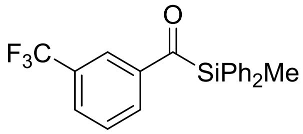

^{1}H NMR (400 MHz, $CDCl_3$) δ 8.01 (s, *J* = 1.6 Hz, 1H), 7.92 (d, *J* = 7.7 Hz, 1H), 7.71 (d, *J* = 7.8 Hz, 1H), 7.61 (dd, *J* = 8.0, 1.5 Hz, 4H), 7.49 – 7.38 (m, 7H), 0.89 (s, 3H).

^{13}C NMR (101 MHz, $CDCl_3$) δ 231.3, 141.9, 135.2, 133.1, 131.5 (c, $J_{C\text{-}F}$ = 1.1 Hz) , 131.2 (d, $J_{C\text{-}F}$ = 32.9 Hz), 130.5, 129.3, 129.3 (d, $J_{C\text{-}F}$ = 3.6 Hz), 128.5, 125.0 (d, $J_{C\text{-}F}$ = 3.8 Hz), 123.7 (d, $J_{C\text{-}F}$ = 272.9 Hz) -3.5.

^{19}F NMR (376 MHz, $CDCl_3$) δ -63.02.

HRMS (ESI+, m/Z): calcd for $C_{21}H_{17}F_3OSiNa$ $[M+Na]^+$: 393.0893; found: 393.0885.

(methyldiphenylsilyl)(4-(trifluoromethoxy)phenyl)methanone, ***9p***

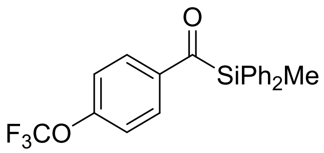

^1H NMR (400 MHz, $CDCl_3$) δ 7.82 (d, J = 8.7 Hz, 2H), 7.65 – 7.55 (m, 4H), 7.51 – 7.37 (m, 6H), 7.17 (d, J = 8.3 Hz, 2H), 0.88 (s, 3H).

^{13}C NMR (101 MHz, $CDCl_3$) δ 230.5, 152.4, 139.8, 135.2, 133.4, 130.4, 130.2, 128.5, 120.4, 73.3, -3.3.

^{19}F NMR (376 MHz, $CDCl_3$) δ -57.56.

HRMS (ESI+, m/Z): calcd for $C_{21}H_{17}F_3O_2SiNa$ $[M+Na]^+$: 409.0842; found: 409.0842.

(methyldiphenylsilyl)(3-(trifluoromethoxy)phenyl)methanone, ***9q***

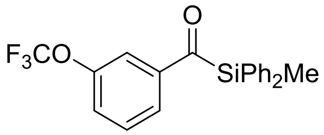

^1H NMR (400 MHz, $CDCl_3$) δ 7.76 – 7.70 (m, 1H), 7.648 – 7.62(m, 5H), 7.52 – 7.28 (m, 8H), 0.92 (s, 3H).

^{19}F NMR (189 MHz, $CDCl_3$) δ -57.93.

^{13}C NMR (101 MHz, $CDCl_3$) δ 230.7, 149.5, 143.1, 135.1, 133.1, 130.3, 130.1, 128.4, 126.8, 125.2, 120.3 (q, J_{C-F} = 258.2 Hz), 119.8, -3.5.

HRMS (ESI+, m/Z): calcd for $C_{21}H_{17}F_3O_2SiNa$ $[M+Na]^+$: 409.0842; found: 409.0832.

Synthesis and characterization of silyl ketimines:

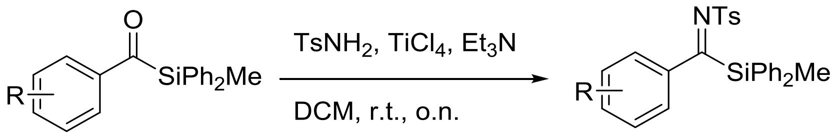

Silyl ketimines were synthesized according to the literature procedure.[6]

(E)-4-methyl-N-((methyldiphenylsilyl)(phenyl)methylene)benzenesulfonamide, ***1a***[7]

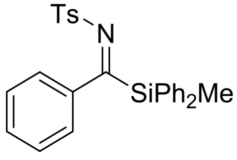

^{1}H NMR (400 MHz, $CDCl_3$) δ 7.70 (d, *J* = 8.1 Hz, 2H), 7.47 (d, *J* = 7.3 Hz, 2H), 7.44 – 7.38 (m, 2H), 7.35 – 7.27 (m, 5H), 7.25 – 7.19 (m, 4H), 7.05 (d, *J* = 7.5 Hz, 2H), 2.41 (s, 3H), 0.73 (s, 3H).

^{13}C NMR (101 MHz, $CDCl_3$) δ 201.9, 143.6, 138.3, 135.4, 132.5, 130.3, 129.6, 129.4, 128.4, 128.2, 127.9, 127.7, 125.9, 21.7, -4.1.

HRMS (ESI+, m/Z): calcd for $C_{27}H_{26}NO_2SSi$ $[M+H]^+$: 456.1448; found: 456.1443.

(E)-4-methyl-N-((methyldiphenylsilyl)(phenyl)methylene)benzenesulfonamide, ***1ab***[7]

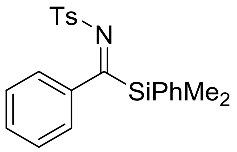

^{1}H NMR (400 MHz, $CDCl_3$) δ 7.73 (d, *J* = 8.2 Hz, 2H), 7.46– 7.37 (m, 3H), 7.35 – 7.22 (m, 7H), 7.03 (d, *J* = 5.1 Hz, 2H), 2.41 (s, 3H), 0.49 (s, 6H).

^{13}C NMR (101 MHz, $CDCl_3$) δ 203.7, 143.5, 138.2, 134.3, 130.1, 129.4, 128.1, 127.9, 127.6, 125.4, 21.6, -3.7.

HRMS (ESI+, m/Z): calcd for $C_{22}H_{24}NO_2SSi$ $[M+H]^+$: 394.1292; found: 394.1293.

(E)-4-methyl-N-(phenyl(triethylsilyl)methylene)benzenesulfonamide, ***1ac***

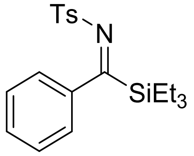

^{1}H NMR (400 MHz, $CDCl_3$) δ 7.72 (d, *J* = 8.1 Hz, 2H), 7.39 – 7.32 (m, 3H), 7.25 – 7.21 (m, 2H), 7.19 – 7.14 (m, 2H), 2.40 (s, 3H), 0.93 – 0.85 (m, 9H), 0.76 – 0.67 (m, 6H).

^{13}C NMR (101 MHz, $CDCl_3$) δ 205.4, 143.4, 138.5, 129.4, 129.1, 128.2, 127.6, 124.7, 21.7, 7.2, 2.9.

HRMS (ESI+, m/Z): calcd for $C_{20}H_{28}NO_2SSi$ $[M+H]^+$: 374.1605; found: 374.1599.

*(E)-4-methyl-N-(phenyl(triphenylsilyl)methylene)benzenesulfonamide, **1ad***

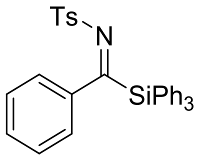

^{1}H NMR (400 MHz, CDCl$_3$) δ 7.67 (d, *J* = 8.0 Hz, 2H), 7.49 – 7.40 (m, 9H), 7.35 – 7.26 (m, 7H), 7.22 – 7.16 (m, 4H), 7.09 – 7.03 (m, 2H), 2.40 (s, 3H).

^{13}C NMR (101 MHz, CDCl$_3$) δ 200.7, 143.5, 139.7, 138.3, 136.5, 131.0, 130.47, 129.6, 129.4, 128.1, 127.8, 127.6, 126.2, 21.7.

HRMS (ESI+, m/Z): calcd for $C_{32}H_{28}NO_2SSi$ [M+H]$^+$: 518.1605; found: 518.1602.

*(E)-N-((tert-butyldiphenylsilyl)(phenyl)methylene)-4-methylbenzenesulfonamide, **1ae***

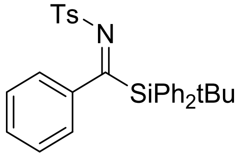

^{1}H NMR (400 MHz, CDCl$_3$) δ 7.79 (d, *J* = 8.1 Hz, 2H), 7.53 – 7.48 (m, 4H), 7.41 – 7.33 (m, 2H), 7.30 – 7.22 (m, 6H), 7.20 – 7.13 (m, 1H), 7.09 (t, *J* 7.9 Hz, 2H), 6.97 (d, *J* = 7.2 Hz, 2H), 2.41 (s, 3H), 1.11 (s, 9H).

^{13}C NMR (101 MHz, CDCl$_3$) δ 202.1, 143.6, 140.1, 138.4, 136.5, 131.3, 129.9, 129.5, 129.2, 127.8, 127.6, 127.5, 125.9, 27.9, 21.7, 19.7.

HRMS (ESI+, m/Z): calcd for $C_{30}H_{32}NO_2SSi$ [M+H]$^+$: 498.1918; found: 498.1916.

*(E)-N-((4-fluorophenyl)(methyldiphenylsilyl)methylene)-4-methylbenzenesulfonamide, **1b***

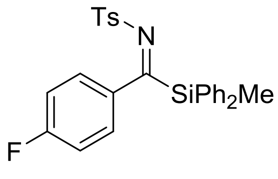

^{1}H NMR (400 MHz, CDCl$_3$) δ 7.69 (d, *J* = 8.1 Hz, 2H), 7.45 – 7.35 (m, 6H), 7.29 (t, *J* = 7.4 Hz, 4H), 7.21 (d, *J* = 7.9 Hz, 2H), 7.10 – 7.02 (m, 2H), 6.88 (t, *J* = 8.5 Hz, 2H), 2.37 (s, 3H), 0.71 (s, 3H).

^{13}C NMR (101 MHz, $CDCl_3$) δ 200.7, 163.2 (d, J_{C-F} = 251.0 Hz), 143.7, 138.1, 135.3, 132.3, 130.4, 129.5, 128.5, 128.4, 128.2, 127.5, 115.0 (d, J_{C-F} = 21.9 Hz)., 21.70, -3.9.

^{19}F NMR (376 MHz, $CDCl_3$) δ -109.98.

HRMS (ESI+, m/Z): calcd for $C_{27}H_{25}FNO_2SSi$ $[M+H]^+$: 474.1354; found: 474.1352.

(E)-N-((3-fluorophenyl)(methyldiphenylsilyl)methylene)-4-methylbenzenesulfonamide, ***1c***

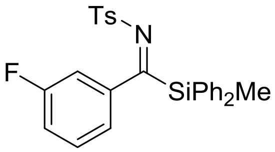

H NMR (400 MHz, $CDCl_3$) δ 7.72 (d, J = 8.2 Hz, 2H), 7.47 (d, J = 7.7 Hz, 2H), 7.46– 7.17 (m, 9H), 6.97 (td, J = 8.5, 2.3 Hz, 1H), 6.86 (d, J = 7.6 Hz, 1H), 6.65 (d, J = 7.3 Hz, 1H), 2.42 (s, 3H), 0.74 (s, 3H).

^{19}F NMR (376 MHz, $CDCl_3$) δ -110.07.

^{13}C NMR (101 MHz, $CDCl_3$) δ 200.2, 166.6, 162.0 (d, J = 248.2 Hz), 143.9, 137.9 (d, J = 7.4 Hz), 135.3, 132.0, 130.5, 129.8 (d, J = 7.8 Hz), 129.5, 128.3, 127.7, 121.6*, 116.3 (d, J = 21.2 Hz), 112.8 (d, J = 23.4 Hz), 21.71, -4.6.

HRMS (ESI+, m/Z): calcd for $C_{27}H_{25}FNO_2SSi$ $[M+H]^+$: 474.1354; found: 474.1352.

(E)-N-((2-fluorophenyl)(methyldiphenylsilyl)methylene)-4-methylbenzenesulfonamide, ***1d***

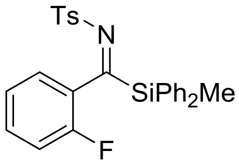

^{1}H NMR (400 MHz, $CDCl_3$) δ 7.71 (d, J = 7.9 Hz, 2H), 7.47(d, J = 6.8 Hz, 4H), 7.40 (d, J = 7.4 Hz, 2H), 7.32– 7.21 (m, 7H), 7.03 (t, J = 7.4 Hz, 1H), 6.92 (t, J = 6.6 Hz, 1H), 6.84 (t, J = 8.8 Hz, 1H), 2.41 (s, 3H), 0.70 (s, 3H).

^{19}F NMR (376 MHz, $CDCl_3$) δ -110.86.

^{13}C NMR (101 MHz, $CDCl_3$) δ 197.7, 156.6 (d, J_{C-F} = 246.2 Hz), 143.9, 137.3, 135.3, 131.9, 130.9 (d, J_{C-F} = 7.7 Hz), 130.3, 129.5, 128.0, 127.8, 127.5 (d, J_{C-F} = 18.2 Hz), 126.8 (d, J_{C-F} = 2.6 Hz), 123.9 (d, J_{C-F} = 2.6 Hz), 115.2 (d, J_{C-F} = 20.7 Hz), 21.7, -4.8.

HRMS (ESI+, m/Z): calcd for $C_{27}H_{25}FNO_2SSi$ $[M+H]^+$: 474.1354; found: 474.1351.

(E)-N- ((4-chlorophenyl)(methyldiphenylsilyl)methylene)-4-methylbenzenesulfonamide, ***1e***

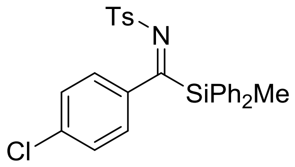

^{1}H NMR (400 MHz, $CDCl_3$) δ 7.71 (d, *J* = 8.2 Hz, 2H), 7.47 – 7.44 (m, 4H), 7.43 – 7.40 (m, 2H), 7.33 (t, *J* = 7.3 Hz, 4H), 7.25 (d, *J* = 7.8 Hz, 4H), 7.20 (d, *J* = 8.3 Hz, 2H), 6.98 (d, *J* = 8.1 Hz, 2H), 2.43 (s, 3H), 0.73 (s, 3H).

^{13}C NMR (101 MHz, $CDCl_3$) δ 200.5, 143.8, 138.0, 135.7, 135.3, 132.0, 130.4, 129.5, 128.2, 128.2, 127.6, 127.4, 21.7, -4.2.

HRMS (ESI+, m/Z): calcd for $C_{27}H_{25}ClNO_2SSi$ $[M+H]^+$: 490.0158; found: 490.1058.

(E)-N-((3-chlorophenyl)(methyldiphenylsilyl)methylene)-4-methylbenzenesulfonamide, ***1f***

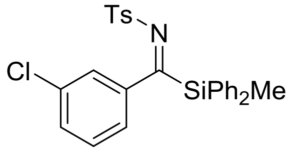

H NMR (400 MHz, $CDCl_3$) δ 7.69 (d, *J* = 8.3 Hz, 2H), 7.50 – 7.42 (m, 7H), 7.37 – 7.33 (m, 4H), 7.24 (d, *J* = 8.0 Hz, 2H), 7.16 (t, *J* = 7.8 Hz, 1H), 6.93 (d, *J* = 7.6 Hz, 1H), 6.82 (s, 1H), 2.42 (s, 3H), 0.76 (s, 3H).

^{13}C NMR (101 MHz, $CDCl_3$) δ 200.0, 143.9, 141.0, 137.7, 135.3, 134.0, 131.8, 130.5, 129.5, 129.3, 128.2, 127.7, 125.5, 123.9, 21.7, -4.4.

HRMS (ESI+, m/Z): calcd for $C_{27}H_{25}ClNO_2SSi$ $[M+H]^+$: 490.1058; found: 490.1057.

(E)-N-((4-bromophenyl)(methyldiphenylsilyl)methylene)-4-methylbenzenesulfonamide, ***1g***

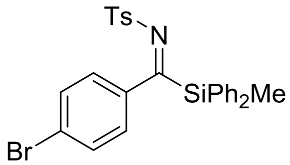

^{1}H NMR (400 MHz, $CDCl_3$) δ 7.71 (d, *J* = 8.2 Hz, 2H), 7.47– 7.40 (m, 6H), 7.37 – 7.30 (m, 6H), 7.25 (d, *J* = 7.9 Hz, 2H), 6.90 (d, *J* = 8.1 Hz, 2H) 2.43 (s, 3H), 0.73 (s, 3H).

^{13}C NMR (101 MHz, $CDCl_3$) δ 200.5, 143.8, 138.5, 137.9, 135.3, 131.9, 131.1, 130.5, 129.5, 128.3, 127.6, 127.5, 124.1, 21.7, -4.1.

HRMS (ESI+, m/Z): calcd for $C_{27}H_{25}BrNO_2SSi$ $[M+H]^+$: 534.0553; found: 534.0555.

(E)-N-((3-bromophenyl)(methyldiphenylsilyl)methylene)-4-methylbenzenesulfonamide, ***1h***

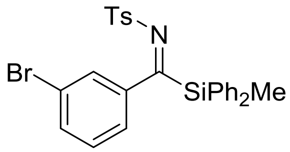

H NMR (400 MHz, $CDCl_3$) δ 7.71 (d, *J* = 7.9 Hz, 2H), 7.51 (d, *J* = 7.4 Hz, 4H), 7.48– 7.35 (m, 7H), 7.25 (d, *J* = 8.2 Hz, 2H), 7.11 (t, *J* = 7.8 Hz, 1H), 7.01 (d, *J* = 7.7 Hz, 1H), 6.98 (s, 1H), 2.43 (s, 3H), 0.79 (s, 3H).

^{13}C NMR (101 MHz, $CDCl_3$) δ 199.8, 143.9, 141.2, 137.6, 135.2, 132.1, 131.7, 130.7, 130.5, 129.5, 128.2, 127.9, 127.6, 124.3, 122.0, 21.7, -4.4.

HRMS (ESI+, m/Z): calcd for $C_{27}H_{25}BrNO_2SSi$ $[M+H]^+$: 534.0553; found: 534.0542.

(E)-4-methyl-N-((methyldiphenylsilyl)(4-(trifluoromethyl)phenyl)methylene)benzenesulfonamide, ***1i***

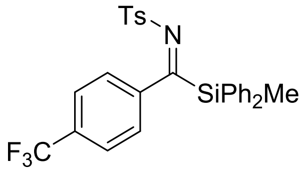

^{1}H NMR (400 MHz, $CDCl_3$) δ 7.70 (d, *J* = 8.2 Hz, 2H), 7.48 – 7.40 (m, 8H), 7.36 – 7.31 (m, 4H), 7.24 (d, *J* = 8.0 Hz, 2H), 7.04 (d, *J* = 7.9 Hz, 2H), 2.42 (s, 3H), 0.74 (s, 3H).

^{19}F NMR (376 MHz, $CDCl_3$) δ -62.87.

^{13}C NMR (101 MHz, $CDCl_3$) δ 200.4, 144.1, 142.9, 137.6, 135.3, 131.4, 130.6, 129.6, 128.3, 127.7, 125.7, 124.9, 124.9, 21.7, -4.6.

HRMS (ESI+, m/Z): calcd for $C_{28}H_{25}F_3NO_2SSi$ $[M+H]^+$: 524.1322; found: 524.1311.

(E)-4-methyl-N-((methyldiphenylsilyl)(3-(trifluoromethyl)phenyl)methylene)benzenesulfonamide, ***1j***

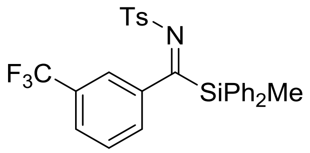

H NMR (400 MHz, $CDCl_3$) δ 7.67 (d, *J* = 8.3 Hz, 2H), 7.52– 7.41 (m, 7H), 7.38– 7.31 (m, 5H), 7.28–7.21 (m, 3H), 6.96 (s, 1H), 2.41 (s, 3H), 0.77 (s, 3H).

^{19}F NMR (376 MHz, $CDCl_3$) δ -63.01.

^{13}C NMR (101 MHz, $CDCl_3$) δ 200.1, 143.9, 137.4, 136.5, 135.2, 131.3, 130.4, 130.0 (c, $J_{C\text{-}F}$ = 32.8 Hz), 129.4, 129.0, 128.4, 128.2, 127.6, 125.7 (c, $J_{C\text{-}F}$ = 3.8 Hz), 123.5 (d, $J_{C\text{-}F}$ = 272.6 Hz), 122.4 (c, $J_{C\text{-}F}$ = 8.6 Hz), 21.5, -4.6.

HRMS (ESI+, m/Z): calcd for $C_{28}H_{25}F_3NO_2SSi$ $[M+H]^+$: 524.1322; found: 524.1324.

(E)-4-methyl-N-((methyldiphenylsilyl)(p-tolyl)methylene)benzenesulfonamide, ***1k***

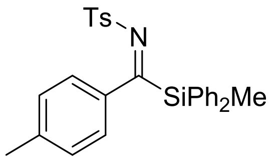

^{1}H NMR (400 MHz, $CDCl_3$) δ 7.73 (d, *J* = 8.2 Hz, 2H), 7.50 – 7.45 (m, 4H), 7.44 – 7.39 (m, 2H), 7.35 – 7.29 (m, 4H), 7.24 (d, *J* = 8.1 Hz, 2H), 7.06 (s, 4H), 2.42 (s, 3H), 2.33 (s, 3H), 0.74 (s, 3H).

^{13}C NMR (101 MHz, $CDCl_3$) δ 201.7, 143.4, 140.3, 138.5, 135.3, 132.8, 130.2, 129.4, 128.7, 128.1, 127.5, 126.4, 21.7, 21.6, -3.8.

HRMS (ESI+, m/Z): calcd for $C_{28}H_{28}NO_2SSi$ $[M+H]^+$: 470.1605; found: 470.1604.

(E)-4-methyl-N-((methyldiphenylsilyl)(m-tolyl)methylene)benzenesulfonamide, ***1l***

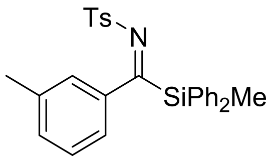

^{1}H NMR (400 MHz, $CDCl_3$) δ 7.69 (d, *J* = 8.1 Hz, 2H), 7.49 – 7.45 (m, 4H), 7.45 – 7.38 (m, 2H), 7.36 – 7.28 (m, 4H), 7.22 (d, *J* = 8.0 Hz, 2H), 7.16 – 7.07 (m, 2H), 6.90 (d, *J* = 7.3 Hz, 1H), 6.74 (s, 1H), 2.41 (s, 3H), 2.19 (s, 3H), 0.73 (s, 3H).

^{13}C NMR (101 MHz, $CDCl_3$) δ 202.1, 143.5, 138.3, 137.5, 135.4, 132.6, 130.3, 130.2, 129.4, 128.3, 128.1, 127.8, 127.6, 126.5, 123.1, 21.7, 21.4, -4.1.

HRMS (ESI+, m/Z): calcd for $C_{28}H_{28}NO_2SSi$ $[M+H]^+$: 470.1605; found: 470.1605.

(E)-N-((4-methoxyphenyl)(methyldiphenylsilyl)methylene)-4-methylbenzenesulfonamide, ***1m***

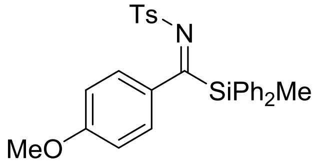

^{1}H NMR (400 MHz, $CDCl_3$) δ 7.73 (d, *J* = 8.2 Hz, 2H), 7.50 – 7.43 (m, 4H), 7.44 – 7.36 (m, 2H), 7.35 – 7.28 (m, 4H), 7.27 – 7.19 (m, 4H), 6.77 (d, *J* = 8.8 Hz, 2H), 3.79 (s, 3H), 2.41 (s, 3H), 0.75 (s, 3H).

^{13}C NMR (101 MHz, $CDCl_3$) δ 200.4, 161.4, 143.3, 138.8, 135.3, 133.2, 130.2, 129.4, 128.2, 127.5, 113.4, 55.4, 21.7, -3.3.

HRMS (ESI+, m/Z): calcd for $C_{28}H_{28}NO_2SSi$ $[M+H]^+$: 486.1554; found: 486.1550.

(*E*)*-N-((3-methoxyphenyl)(methyldiphenylsilyl)methylene)-4-methylbenzenesulfonamide,* ***1n***

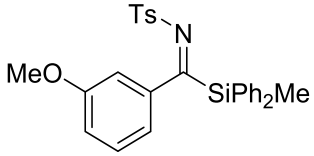

^{1}H NMR (400 MHz, $CDCl_3$) δ 7.70 (d, *J* = 8.1 Hz, 2H), 7.53 – 7.45 (m, 4H), 7.45 – 7.38 (m, 2H), 7.36 – 7.31 (m, 4H), 7.22 (d, *J* = 8.0 Hz, 2H), 7.15 (t, *J* = 8.0 Hz, 1H), 6.83 (dd, *J* = 8.4, 2.5 Hz, 1H), 6.69 (d, *J* = 7.5 Hz, 1H), 6.45 (s, 1H), 3.56 (s, 3H), 2.41 (s, 3H), 0.75 (s, 3H).

^{13}C NMR (101 MHz, $CDCl_3$) δ 201.4, 158.8, 143.5, 140.9, 138.2, 135.3, 132.5, 130.3, 129.4, 129.1, 128.2, 127.6, 118.4, 116.2, 110.5, 55.1, 21.7, -4.2.

HRMS (ESI+, m/Z): calcd for $C_{28}H_{28}NO_2SSi$ $[M+H]^+$: 486.1554; found: 486.1552.

(E)-N-((4-(tert-butyl)phenyl)(methyldiphenylsilyl)methylene)-4-methylbenzenesulfonamide, ***1o***

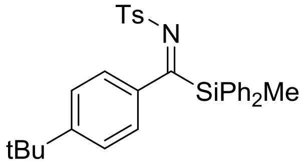

^{1}H NMR (400 MHz, $CDCl_3$) δ 7.67 (d, *J* = 8.2 Hz, 2H), 7.48 – 7.43 (m, 4H), 7.43 – 7.37 (m, 2H), 7.31 (t, *J* = 7.4 Hz, 4H), 7.22 (d, *J* = 8.3 Hz, 2H), 7.19 (d, *J* = 8.1 Hz, 2H), 7.04 (d, *J* = 8.1 Hz, 2H), 2.39 (s, 3H), 1.27 (s, 9H), 0.73 (s, 3H).

^{13}C NMR (101 MHz, $CDCl_3$) δ 201.9, 153.2, 143.3, 138.2, 135.4, 133.0, 130.2, 129.3, 128.1, 127.6, 126.3, 124.9, 34.9, 31.3, 21.7, -3.7.

HRMS (ESI+, m/Z): calcd for $C_{31}H_{34}NO_2SSi$ $[M+H]^+$: 512.2074; found: 512.2071.

*(E)-4-methyl-N-((methyldiphenylsilyl)(4-(trifluoromethoxy)phenyl)methylene)benzenesulfonamide, **1p***

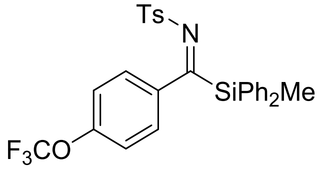

^{1}H NMR (400 MHz, $CDCl_3$) δ 7.69 (d, *J* = 8.3 Hz, 2H), 7.49 – 7.39 (m, 6H), 7.37 – 7.30 (m, 4H), 7.24 (d, *J* = 8.0 Hz, 2H), 7.04 (s, 4H), 2.41 (s, 3H), 0.76 (s, 3H).

^{19}F NMR (376 MHz, $CDCl_3$) δ -57.73.

^{13}C NMR (101 MHz, $CDCl_3$) δ 200.5, 149.8, 143.9, 137.8, 135.3, 131.9, 130.5, 129.5, 128.4, 128.3, 127.6, 120.1, 21.7, -4.4.

HRMS (ESI+, m/Z): calcd for $C_{28}H_{25}F_3NO_3SSi$ $[M+H]^+$: 540.1271; found: 540.1272.

*(E)-4-methyl-N-((methyldiphenylsilyl)(3-(trifluoromethoxy)phenyl)methylene)benzenesulfonamide, **1q***

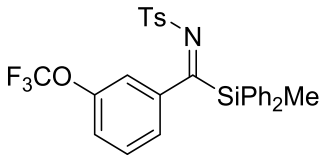

^{1}H NMR (400 MHz, $CDCl_3$) δ 7.66 (d, *J* = 8.0 Hz, 2H), 7.47 – 7.34 (m, 6H), 7.29 (t, *J* = 7.4 Hz, 4H), 7.23 – 7.16 (m, 3H), 7.11 – 7.03 (m, 1H), 6.91 (d, *J* = 7.7 Hz, 1H), 6.72 (s, 1H), 2.36 (s, 3H), 0.71 (s, 3H).

^{19}F NMR (376 MHz, $CDCl_3$) δ -57.83.

^{13}C NMR (101 MHz, $CDCl_3$) δ 199.9, 148.6, 144.0, 141.2, 137.6, 135.2, 131., 130.5, 129.6, 129.5, 128.3, 127.7, 124.0, 121.5, 120.3 (q, $J_{C\text{-}F}$= 257.8 Hz), 118.1, 21.6, -4.5.

HRMS (ESI+, m/Z): calcd for $C_{28}H_{25}F_3NO_3SSi$ $[M+H]^+$: 540.1271; found: 540.1255.

(E)-4-methyl-N-((methyldiphenylsilyl)(naphthalen-2-yl)methylene)benzenesulfonamide, ***1r***

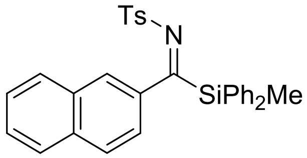

^{1}H NMR (400 MHz, $CDCl_3$) δ 7.79 (d, *J* = 7.9 Hz, 1H), 7.71 (d, *J* = 8.3 Hz, 3H), 7.65 (d, *J* = 8.0 Hz, 1H), 7.56 – 7.40 (m, 9H), 7.38 – 7.30 (m, 4H), 7.22 (d, *J* = 8.6 Hz, 1H), 7.17 (d, *J* = 8.0 Hz, 2H), 2.37 (s, 3H), 0.79 (s, 3H).

^{13}C NMR (101 MHz, $CDCl_3$) δ 201.6, 143.6, 138.2, 137.2, 135.4, 133.4, 132.5, 132.2, 130.3, 129.3, 128.8, 128.2, 127.8, 127.6, 127.3, 126.6, 126.0, 123.4, 21.6, -4.0.

HRMS (ESI+, m/Z): calcd for $C_{31}H_{28}NO_2SSi$ $[M+H]^+$: 506.1605; found: 506.1605.

(*E*)-4-methyl-N-((methyldiphenylsilyl)(thiophen-3-yl)methylene)benzenesulfonamide*,* **1s**

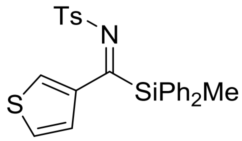

^{1}H NMR (400 MHz, Chloroform-*d*) δ 7.74 (d, *J* = 8.2 Hz, 2H), 7.53 – 7.46 (m, 5H), 7.43 (t, *J* = 7.4 Hz, 2H), 7.37-7.33 (m, 4H), 7.25 (d, *J* = 8.0 Hz, 2H), 7.21-7.15 (m, 2H), 2.42 (s, 3H), 0.79 (s, 3H).

^{13}C NMR (101 MHz, $CDCl_3$) δ 194.1, 143.3, 138.7, 135.3, 133.0, 130.3, 129.8, 129.4, 128.3, 127.8, 127.3, 125.4, 21.7, -3.4.

HRMS (ESI+, m/Z): calcd for $C_{25}H_{24}NO_2S_2Si$ $[M+H]^+$: 462,10122; found: 462,10106.

(E)-3-methyl-N-((methyldiphenylsilyl)(phenyl)methylene)benzenesulfonamide, ***4b***

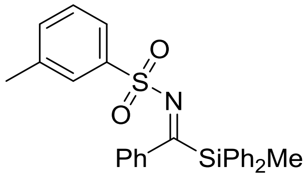

1H NMR (400 MHz, $CDCl_3$) δ 7.72 – 7.65 (m, 2H), 7.55 (d, J = 7.2 Hz, 4H), 7.45 (d, J = 7.2 Hz, 2H), 7.40– 7.24 (m, 9H), 7.12 (d, J = 7.2 Hz, 2H), 2.38 (s,3H), 0.80 (s, 3H).

^{13}C NMR (101 MHz, $CDCl_3$) δ 202.3, 140.8, 139.7, 138.7, 135.2, 133.4, 132.3, 130.2, 129.5, 128.6, 128.1, 127.9, 127.8, 125.7, 124.5, 21.2, -4.2.

HRMS (ESI+, m/Z): calcd for $C_{27}H_{26}NO_2SSi$ $[M+H]^+$: 456.1448; found: 456.1436.

(E)-2-methyl-N-((methyldiphenylsilyl)(phenyl)methylene)benzenesulfonamide, ***4c***

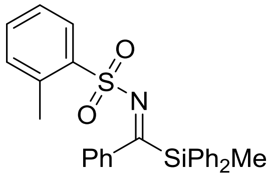

^{1}H NMR (400 MHz, $CDCl_3$) δ 7.90 (d, *J* = 7.9 Hz, 1H), 7.58 – 7.52 (m, 4H), 7.53 – 7.45 (m, 3H), 7.43 – 7.35 (m, 5H), 7.34 – 7.37 (m, 4H), 7.16 (d, *J* = 7.5 Hz, 2H), 2.62 (s, 3H), 0.82 (s, 3H).

^{13}C NMR (101 MHz, $CDCl_3$) δ 202.6, 139.0, 138.2, 135.4, 135.2, 132.8, 132.2, 132.0, 130.2, 129.5, 128.7, 128.1, 127.9, 125.8, 125.6, 20.5, -4.2.

HRMS (ESI+, m/Z): calcd for $C_{27}H_{26}NO_2SSi$ $[M+H]^+$: 456.1448; found: 456.1436.

(E)-4-isopropyl-N-((methyldiphenylsilyl)(phenyl)methylene)benzenesulfonamide, ***4d***

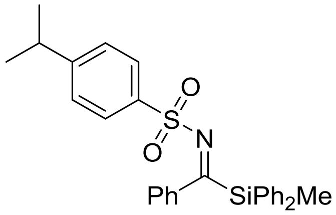

^{1}H NMR (400 MHz, $CDCl_3$) δ 7.75 – 7.68 (m, 2H), 7.49 – 7.44 (m, 4H), 7.43 – 7.37 (m, 1H), 7.34 – 7.24 (m, 8H), 7.23 – 7.17 (m, 2H), 7.02 (d, *J* = 7.6 Hz, 2H), 2.95 (p, *J* = 6.9 Hz, 1H), 1.26 (d, *J* = 6.9 Hz, 6H), 0.73 (s, 3H).

^{13}C NMR (101 MHz, $CDCl_3$) δ 201.8, 154.2, 138.3, 135.3, 132.4, 130.2, 129.4, 128.2, 128.1, 127.8, 127.7, 126.8, 125.8, 34.2, 23.7, -4.2.

HRMS (ESI+, m/Z): calcd for $C_{29}H_{30}NO_2SSi$ $[M+H]^+$: 484.1761; found: 484.1751.

(E)-4-fluoro-N-((methyldiphenylsilyl)(phenyl)methylene)benzenesulfonamide, ***4e***

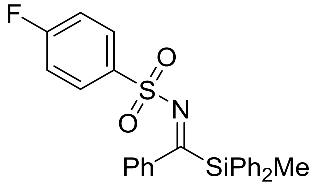

^1H NMR (400 MHz, $CDCl_3$) δ 7.83 (dd, *J* = 8.6, 5.3 Hz, 2H), 7.50 – 7.40 (m, 6H), 7.37 – 7.31 (m, 5H), 7.26 (t, *J* = 7.5 Hz, 2H), 7.13 – 7.07 (m, 4H), 0.76 (s, 3H).

^{13}C NMR (101 MHz, $CDCl_3$) δ 202.7, 165.1 (d, $J_{C\text{-}F}$ = 254.7 Hz), 137.1, 135.2, 132.2, 130.3, 130.2, 130.0, 129.7, 128.1, 127.9, 125.8, 115.9 (d, $J_{C\text{-}F}$ = 22.7 Hz), -4.2.

^{19}F NMR (376 MHz, $CDCl_3$) δ -105.24.

HRMS (ESI+, m/Z): calcd for $C_{26}H_{23}FNO_2SSi$ $[M+H]^+$: 460.1197; found: 460.1185.

(E)-N-((methyldiphenylsilyl)(phenyl)methylene)thiophene-2-sulfonamide, ***4f***

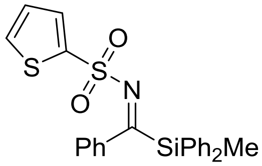

^1H NMR (400 MHz, $CDCl_3$) δ 7.60 (dd, *J* = 5.0, 1.4 Hz, 1H), 7.53 (dd, *J* = 3.8, 1.4 Hz, 1H), 7.52 – 7.46 (m, 4H), 7.45 – 7.38 (m, 2H), 7.37 – 7.29 (m, 5H), 7.27 – 7.21 (m, 2H), 7.06 (d, *J* = 7.5 Hz, 2H), 7.00 (dd, *J* = 5.0, 3.8 Hz, 1H), 0.77 (s, 3H).

^{13}C NMR (101 MHz, $CDCl_3$) δ 202.3, 141.9, 139.6, 135.4, 132.9, 132.9, 132.2, 130.4, 129.8, 128.2, 128.0, 127.0, 125.8, -4.0.

HRMS (ESI+, m/Z): calcd for $C_{24}H_{22}NO_2S_2Si$ $[M+H]^+$: 448.0856; found: 448.0843.

(E)-N-((methyldiphenylsilyl)(phenyl)methylene)methanesulfonamide, ***4g***

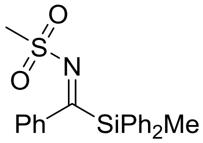

^{1}H NMR (400 MHz, $CDCl_3$) δ 7.58 – 7.52 (m, 4H), 7.50 – 7.43 (m, 2H), 7.43 – 7.31 (m, 5H), 7.31 – 7.25 (m, 2H), 7.14 (d, *J* = 7.5 Hz, 2H), 3.14 (s, 3H), 0.80 (s, 3H).

^{13}C NMR (101 MHz, $CDCl_3$) δ 203.0, 139.6, 135.3, 132.3, 130.4, 129.8, 128.2, 128.0, 125.8, 42.8, -4.0.

HRMS (ESI+, m/Z): calcd for $C_{21}H_{22}NO_2SSi$ $[M+H]^+$: 380.1135; found: 380.1125.

(E)-3,5-dimethyl-N-((methyldiphenylsilyl)(phenyl)methylene)benzenesulfonamide, ***4h***

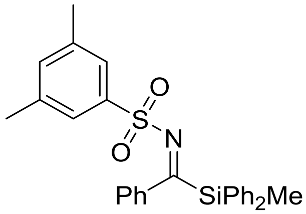

^{1}H NMR (400 MHz, $CDCl_3$) δ 7.50 – 7.46 (m, 4H), 7.44 – 7.37 (m, 4H), 7.35 – 7.25 (m, 5H), 7.25 – 7.18 (m, 2H), 7.12 (s, 1H), 7.02 (d, *J* = 7.5 Hz, 2H), 2.30 (s, 6H), 0.73 (s, 3H).

^{13}C NMR (101 MHz, $CDCl_3$) δ 202.2, 140.6, 138.6, 135.3, 134.4, 132.4, 130.2, 129.4, 128.2, 128.1, 127.8, 125.7, 125.1, 21.2, -4.2.

HRMS (ESI+, m/Z): calcd for $C_{28}H_{28}NO_2SSi$ $[M+H]^+$: 470.1605; found: 470.1596.

(*E*)-2,4,6-trimethyl-*N*-((methyldiphenylsilyl)(phenyl)methylene)benzenesulfonamide, **4i**

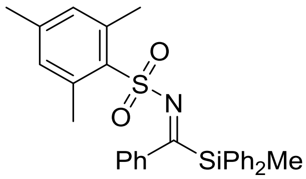

^{1}H NMR (400 MHz, $CDCl_3$) δ 7.46 (dd, *J* = 8.0, 1.4 Hz, 4H), 7.43 – 7.38 (m, 2H), 7.31 (t, *J* = 7.2 Hz, 4H), 7.24 – 7.19 (m, 1H), 7.15 (d, *J* = 7.4 Hz, 2H), 6.91 (d, *J* = 8.0 Hz, 2H), 6.80 (s, 2H), 2.46 (s, 6H), 2.25 (s, 3H), 0.71 (s, 3H).

^{13}C NMR (101 MHz, $CDCl_3$) δ 201.2, 142.3, 139.3, 135.3, 132.4, 132.0, 131.5, 130.2, 129.0, 128.1, 127.7, 125.2, 22.7, 21.0, -4.3.

HRMS (ESI+, m/Z): calcd for $C_{29}H_{30}NO_2SSi$ $[M+H]^+$: 484.1761; found: 484.1749.

(E)-4-methoxy-N-((methyldiphenylsilyl)(phenyl)methylene)benzenesulfonamide, ***4j***

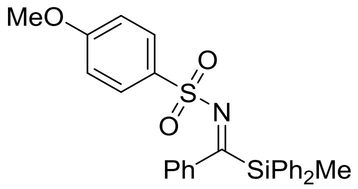

^{1}H NMR (400 MHz, $CDCl_3$) δ 7.79 (d, *J* = 7.2 Hz, 2H), 7.55 – 7.50 (m, 4H), 7.44 (d, *J* = 7.2 Hz, 2H), 7.38– 7.23 (m, 7H), 7.10 (s, 2H), 6.92 (d, *J* = 8.6 Hz, 4H), 2.38 (s,3H), 0.80 (s, 3H).

^{13}C NMR (101 MHz, $CDCl_3$) δ 201.2, 163.0, 139.6, 135.2, 132.6, 132.4, 130.2, 129.7, 129.4, 128.1, 127.8, 125.7, 113.9, 55.6, -4.2.

HRMS (ESI+, m/Z): calcd for $C_{27}H_{26}NO_3SSi$ $[M+H]^+$: 472.1397; found: 472.1383.

(E)-2-methyl-N-((methyldiphenylsilyl)(phenyl)methylene)propane-2-sulfonamide, ***4k***

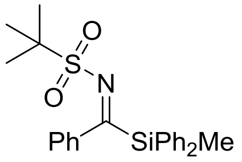

^{1}H NMR (400 MHz, $CDCl_3$) δ 7.59 – 7.52 (m, 4H), 7.48 – 7.43 (m, 2H), 7.42 – 7.36 (m,4H), 7.36 – 7.25 (m, 3H), 7.22 (d, *J* = 6.7 Hz, 2H), 1.43 (s, 9H), 0.78 (s, 3H).

^{13}C NMR (101 MHz, $CDCl_3$) δ 203.9, 140.1, 135.3, 132.5, 130.4, 129.7, 128.2, 128.0, 125.8, 59.3, 24.0, -4.1.

HRMS (ESI+, m/Z): calcd for $C_{24}H_{28}NO_2SSi$ $[M+H]^+$: 422.1605; found: 422.1597.

Cu-catalyzed asymmetric addition of Grignard reagents to silyl ketimines: synthesis and characterization of addition products

General method for the synthesis of racemic amine products:

A Schlenk tube equipped with septum and stirring bar was charged with ketimine (0.05 mmol). Dry MTBE (1 mL) was added and the resulting solution was cooled to –78 °C and stirred for 15 min. Then, the corresponding Grignard reagent (0.1 mmol, in Et_2O) was added dropwise during 1 min by hand. Once the addition was complete, the mixture was stirred for 10h at –78 °C. The reaction was quenched with a saturated aqueous NH_4Cl solution and the mixture was warmed to room temperature, diluted with dichloromethane and the layers were separated. The aqueous layer was extracted with dichloromethane (3 x 5 mL) and the combined organic layers were dried with anhydrous $MgSO_4$, filtered and the solvent was evaporated in *vacuo*. Purification was performed by flash chromatography on silica gel using different mixtures of *n*-pentane:Et_2O as the eluent.

General method for the synthesis of enantioenriched amine products

The general procedure is described for synthesis of product **6e** via the copper-catalysed alkylation of ketimine **1e** with *n*-HexMgBr. A Schlenk tube equipped with septum and stirring bar was charged with CuBr·SMe_2 (0.005 mmol) and ligand (S,R_{Fe})-**L8** (0.006 mmol) and ketimine **1e** (0.1 mmol). Dry MTBE (2.5 mL) was added. The solution was stirred under nitrogen at room temperature for 10 min, cooled to –78 °C and stirred for 15 min. In a separate Schlenk tube, the corresponding Grignard reagent (0.2 mmol, 2M in Et_2O) was diluted with MTBE (combined volume of 1 mL) under nitrogen and added dropwise to the reaction mixture during 40 min using a syringe pump. Once the addition was complete, the mixture was stirred for 10h at –78 °C. The reaction was quenched with a saturated aqueous NH_4Cl solution and the mixture was warmed to room temperature, diluted with dichloromethane and the layers were separated. The aqueous layer was extracted with dichloromethane (3 x 10 mL) and the combined organic layers were dried with anhydrous $MgSO_4$, filtered and the solvent was evaporated in *vacuo*. Purification was performed by flash chromatography on silica gel using different mixtures of *n*-

pentane:Et_2O as the eluent. The enantiomeric excess was determined by chiral HPLC analysis. The main side product to be separated from was the reduction product.

Reduction products were determined as racemic by comparing with reported HPLC data. Addition reduction ratio for all alkylation reaction was determined using ^{1}H-NMR spectroscopy.

Characterization of chiral tertiary α-silyl amines:

*(4-methyl-N-(1-(methyldiphenylsilyl)-1-phenylheptyl)benzenesulfonamide, **2a***

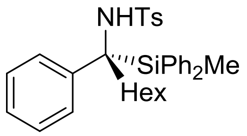

The reaction was performed with 0.1 mmol **1a**, *n*-HexMgBr (0.2 mmol, 2M in Et_2O) diluted with MTBE (1 mL total amount) , CuBr·SMe_2 (1 mg, 0.005 mmol, 5 mol%), ligand (*R,S$_{Fe}$*)-**L8** (3.6 mg, 0.006 mmol, 6 mol%) in 2.5 mL MTBE. Product **2a** was obtained as a white solid after column chromatography (SiO_2, pentane:Et_2O 80:20), [94% yield, 88% e.e.].

^{1}H NMR (400 MHz, $CDCl_3$) δ 7.49 – 7.35 (m, 8H), 7.33 – 7.25 (m, 4H), 7.12 (d, *J* = 7.9 Hz, 2H), 7.09 – 7.01 (m, 3H), 6.93 (d, *J* = 7.5 Hz, 2H), 4.98 (s, 1H), 2.38 (s, 3H), 2.36 – 2.20 (m, 2H), 1.24 – 0.86 (m, 8H), 0.80 (t, *J* = 7.3 Hz, 3H), 0.62 (s, 3H).

^{13}C NMR (101 MHz, $CDCl_3$) δ 142.8, 141.7, 140.2, 135.9, 135.7, 134.1, 133.8, 129.8, 129.8, 129.3, 127.9, 127.9, 127.5, 127.4, 127.1, 125.9, 59.4, 34.9, 31.6, 29.8, 25.1, 22.7, 21.6, 14.2, -4.6.

HRMS (ESI+, m/Z): calcd for $C_{33}H_{40}NO_2SSi$ $[M+H]^+$: 542.2543; found:542.2538.

The enantiomeric ratio was determined by chiral HPLC analysis, Chiralcel OZH column, n-heptane/i-PrOH 97:3, 0.5 mL/min, 40 °C, detection at 230 nm. Retention times 33.2 min (minor) and 38.1 min (major).

*N-(1-(dimethyl(phenyl)silyl)-1-phenylheptyl)-4-methylbenzenesulfonamide, **2ab***

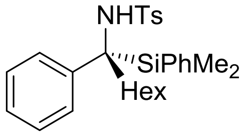

The reaction was performed with 0.1 mmol **1ab**, *n*-HexMgBr (0.2 mmol, 2M in Et_2O) diluted with MTBE (1 mL total amount), CuBr·SMe_2 (1 mg, 0.005 mmol, 5 mol%), ligand(*R,R$_{Fe}$*)-**L4** (3.6 mg,

0.006 mmol, 6 mol%) in 2.5 mL MTBE. Product **2ab** was obtained as white solid after column chromatography (SiO_2, pentane: Et_2O 80:20), [63% yield, 31% e.e.].

^{1}H NMR (400 MHz, $CDCl_3$) δ 7.54 (d, *J* = 8.3 Hz, 2H), 7.38 – 7.31 (m, 1H), 7.26 – 7.21 (m, 2H), 7.21 – 7.15 (m, 4H), 7.11 – 7.06 (m, 3H), 7.00 – 6.95 (m, 2H), 4.72 (s, 1H), 2.40 (s, 3H), 2.19 – 2.09 (m, 1H), 2.02 – 1.92 (m, 1H), 1.21 – 0.86 (m, 8H), 0.82 (t, *J* = 7.3 Hz, 3H), 0.40 (s, 3H), 0.31 (s, 3H).

^{13}C NMR (101 MHz, $CDCl_3$) δ 142.9, 141.8, 140.4, 136.2, 134. 9, 129.4, 129.4, 127.6, 127.0, 127.0, 125.7, 59.1, 34.0, 31.9, 29.8, 23.7, 22.8, 21.6, 14.2, -3.8, -4.2.

HRMS (ESI+, m/Z): calcd for $C_{28}H_{38}NO_2SSi$ $[M+H]^+$: 480.2387; found: 480.2377.

The enantiomeric ratio was determined by chiral HPLC analysis, Chiralcel ODH column, n-heptane/i-PrOH 98:2, 0.5 mL/min, 40 °C, detection at 230 nm. Retention times 15.7 min (major) and 19.0 min (minor).

*4-methyl-N-(1-phenyl-1-(triethylsilyl)heptyl)benzenesulfonamide, **2ac***

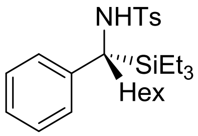

The reaction was performed with 0.1 mmol **1ac**, *n*-HexMgBr (0.2 mmol, 2M in Et_2O) diluted with MTBE (1 mL total amount) , CuBr·SMe_2 (1 mg, 0.005 mmol, 5 mol%), ligand(R,R_{Fe})-**L4** (4.0 mg, 0.006 mmol, 6 mol%) in 2.5 mL MTBE. Product **2ac** was obtained as white solid after column chromatography (SiO_2, pentane: Et_2O 80:20), [61% yield, 34% e.e.].

^{1}H NMR (400 MHz, $CDCl_3$) δ 7.66 (d, *J* = 8.3 Hz, 2H), 7.26 – 7.16 (m, 6H), 7.15 – 7.08 (m, 1H), 4.70 (s, 1H), 2.42 (s, 3H), 2.24 – 2.15 (m, 1H), 2.12 – 2.01 (m, 1H), 1.35 – 0.95 (m, 8H), 0.88 – 0.80 (m, 12H), 0.65 – 0.51 (m, 6H).

^{13}C NMR (101 MHz, $CDCl_3$) δ 143.1, 142. 9, 140.9, 129.4, 127.9, 127.0, 126.5, 125.6, 60.6, 35.3, 32.0, 30.0, 24.7, 22.9, 21.6, 14.2, 8.0, 2.9.

HRMS (ESI+, m/Z): calcd for $C_{26}H_{41}NO_2SSiNa$ $[M+Na]^+$: 482.2520; found:482.2506.

The enantiomeric ratio was determined by chiral HPLC analysis, Chiralcel ODH column, n-heptane/i-PrOH 99:1, 0.5 mL/min, 40 °C, detection at 230 nm. Retention times 22.7 min (major) and 29.6 min (minor).

*4-methyl-N-(1-(methyldiphenylsilyl)-1-phenylpentyl)benzenesulfonamide, **5a***

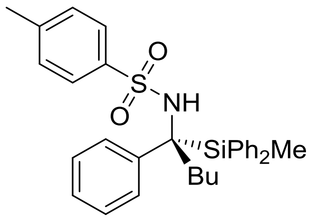

The reaction was performed with 0.1 mmol **1a**, *n*-BuMgBr (0.2 mmol, 2M in Et_2O) diluted with MTBE (1 mL total amount) , CuBr·SMe_2 (1 mg, 0.005 mmol, 5 mol%), ligand(*R,S$_{Fe}$*)-**L8** (3.6 mg, 0.006 mmol, 6 mol%) in 2.5 mL MTBE. The Grignard reagent was added dropwise to the reaction mixture over 2 h using a syringe pump. Product **5a** was obtained as white solid after column chromatography (SiO_2, pentane: Et_2O 80:20), [99% yield, 85% e.e.].

^{1}H NMR (400 MHz, $CDCl_3$) δ 7.48 – 7.35 (m, 8H), 7.30 (td, *J* = 7.3, 3.8 Hz, 4H), 7.12 (d, *J* = 8.0 Hz, 2H), 7.10 – 7.00 (m, 3H), 6.96 – 6.90 (m, 2H), 4.97 (s, 1H), 2.38 (s, 3H), 2.36 – 2.19 (m, 2H), 1.26 – 0.91 (m, 4H), 0.66 (t, *J* = 7.1 Hz, 3H), 0.62 (s, 3H).

^{13}C NMR (101 MHz, $CDCl_3$) δ 142.8, 141.7, 140.3, 135.9, 135.7, 134.1, 133.8, 129.8, 129.8, 129.2, 127.9, 127.5, 127.4, 127.1, 125.9, 59.4, 34.7, 27.3, 23.2, 21.6, 13.8, -4.6.

HRMS (ESI+, m/Z): calcd for $C_{31}H_{35}NO_2SSiNa$ $[M+Na]^+$: 536.2050; found: 536.2040.

The enantiomeric ratio was determined by chiral HPLC analysis, Chiralcel ODH column, n-heptane/i-PrOH 97:3, 0.5 mL/min, 40 °C, detection at 230 nm. Retention times 17.7 min (minor) and 19.3 min (major).

*3-methyl-N-(1-(methyldiphenylsilyl)-1-phenylpentyl)benzenesulfonamide, **5b***

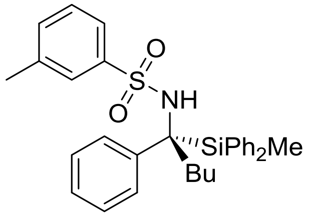

The reaction was performed with 0.1 mmol **4b**, *n*-BuMgBr (0.2 mmol, 2M in Et_2O) diluted with MTBE (1 mL total amount) , CuBr·SMe_2 (1 mg, 0.005 mmol, 5 mol%), ligand(*R,S$_{Fe}$*)-**L8** (3.6 mg, 0.006 mmol, 6 mol%) in 2.5 mL MTBE. The Grignard reagent was added dropwise to the reaction

mixture over 2 h using a syringe pump. Product **5b** was obtained as white solid after column chromatography (SiO_2, pentane: Et_2O 80:20), [78% yield, 83% e.e.].

^{1}H NMR (400 MHz, $CDCl_3$) δ 7.49 – 7.43 (m, 2H), 7.42 – 7.35 (m, 5H), 7.33 – 7.19 (m, 7H), 7.10 – 6.99 (m, 3H), 6.95 – 6.89 (m, 2H), 5.00 (s, 1H), 2.42 – 2.19 (m, 5H), 1.40 – 0.80 (m, 6H), 0.66 (t, *J* = 7.1 Hz, 3H), 0.61 (s, 3H).

^{13}C NMR (101 MHz, $CDCl_3$) δ 142.9, 141.5, 138.7, 135.9, 135.7, 134.1, 133.8, 132.9, 129.9, 129.8, 128. 6, 127.9, 127.9, 127.5, 127.5, 126.0, 124.1, 59.4, 34.8, 27.3, 23.2, 21.4, 13.9, -4.6.

HRMS (ESI+, m/Z): calcd for $C_{31}H_{36}NO_2SSi$ $[M+H]^+$: 514.2231; found:514.2217.

The enantiomeric ratio was determined by chiral HPLC analysis, Chiralcel ODH column, n-heptane/i-PrOH 99:1, 0.5 mL/min, 40 °C, detection at 230 nm. Retention times 34.9 min (minor) and 51.0 min (major).

2-methyl-N-(1-(methyldiphenylsilyl)-1-phenylheptyl)benzenesulfonamide, ***5c***

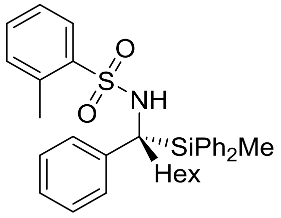

The reaction was performed with 0.1 mmol **4c**, *n*-HexMgBr (0.2 mmol, 2M in Et_2O) diluted with MTBE (1 mL total amount) , CuBr·SMe_2 (1 mg, 0.005 mmol, 5 mol%), ligand(*R,S$_{Fe}$*)-**L8** (3.6 mg, 0.006 mmol, 6 mol%) in 2.5 mL MTBE. The Grignard reagent was added dropwise to the reaction mixture over 2 h using a syringe pump. Product **5c** was obtained as white solid after column chromatography (SiO_2, pentane: Et_2O 70:30), [81% yield, 87% e.e.].

^{1}H NMR (400 MHz, $CDCl_3$) δ 7.51 – 7.46 (m, 3H), 7.43 – 7.37 (m, 4H), 7.36 – 7.28 (m, 5H), 7.20 (d, *J* = 7.6 Hz, 1H), 7.11 – 6.95 (m, 4H), 6.91 (d, *J* = 7.4 Hz, 2H), 5.02 (s, 1H), 2.48 (s, 3H), 2.41 – 2.31 (m, 2H), 1.30 – 0.94 (m, 8H), 0.81 (t, *J* = 7.2 Hz, 3H), 0.56 (s, 3H).

^{13}C NMR (101 MHz, $CDCl_3$) δ 141.8, 141.6, 136.1, 135.8, 135.5, 134.0, 133.6, 132.2, 132.1, 13.0, 129.9, 128.9, 128.1, 128.0, 127.4, 127.3, 126.0, 125.9, 59.6, 36.0, 31. 6, 29.8, 25.7, 22.6, 20.5, 14.2, -4.2.

HRMS (ESI+, m/Z): calcd for $C_{33}H_{40}NO_2SSi$ $[M+H]^+$: 542.2544; found:542.2531.

The enantiomeric ratio was determined by chiral HPLC analysis, Chiralcel ODH column, n-heptane/i-PrOH 99:1, 0.5 mL/min, 40 °C, detection at 230 nm. Retention times 20.4 min (major) and 24.0 min (minor).

*4-isopropyl-N-(1-(methyldiphenylsilyl)-1-phenylpentyl)benzenesulfonamide, **5d***

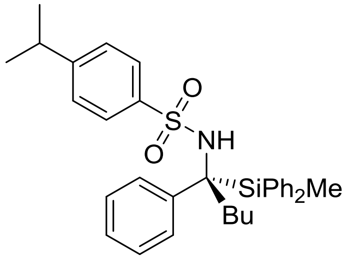

The reaction was performed with 0.1 mmol **4d**, *n*-BuMgBr (0.2 mmol, 2M in Et_2O) diluted with MTBE (1 mL total amount) , CuBr·SMe_2 (1 mg, 0.005 mmol, 5 mol%), ligand(*R,S_{Fe}*)-**L8** (3.6 mg, 0.006 mmol, 6 mol%) in 2.5 mL MTBE. The Grignard reagent was added dropwise to the reaction mixture over 2 h using a syringe pump. Product **5d** was obtained as white solid after column chromatography (SiO_2, pentane: Et_2O 80:20), [68% yield, 78% e.e.].

^{1}H NMR (400 MHz, $CDCl_3$) δ 7.51 – 7.36 (m, 8H), 7.36 – 7.26 (m, 4H), 7.17 (d, *J* = 8.0 Hz, 2H), 7.11 – 6.97 (m, 3H), 6.92 (d, *J* = 7.5 Hz, 2H), 4.99 (s, 1H), 2.93 (hept, *J* = 6.9 Hz, 1H), 2.39 – 2.22 (m, 2H), 1.26 (d, *J* = 6.8 Hz, 6H), 1.18 – 0.83 (m, 6H), 0.70 – 0.58 (m, 6H).

^{13}C NMR (101 MHz, $CDCl_3$) δ 153.6, 141.5, 140.4, 136.0, 135.7 134.2, 133.9, 129.8, 129.8, 127. 9, 127.9, 127.5, 127.4, 127.2, 126.7, 125.9, 59.2, 34.6, 34.3, 27.2, 23.9, 23.8, 23.2, 13.9, -4.6.

HRMS (ESI+, m/Z): calcd for $C_{33}H_{40}NO_2SSi$ $[M+H]^+$: 542.2544; found: 542.2530.

The enantiomeric ratio was determined by chiral HPLC analysis, Chiralcel OZH column, n-heptane/i-PrOH 98:2, 0.5 mL/min, 40 °C, detection at 230 nm. Retention times 31.3 min (minor) and 35.7 min (major).

*4-fluoro-N-(1-(methyldiphenylsilyl)-1-phenylpentyl)benzenesulfonamide, **5e***

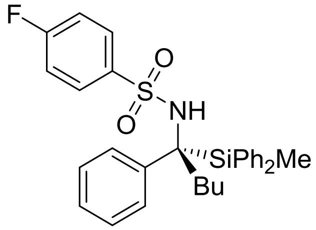

The reaction was performed with 0.1 mmol **4e**, *n*-BuMgBr (0.2 mmol, 2M in Et_2O) diluted with MTBE (1 mL total amount) , CuBr·SMe_2 (1 mg, 0.005 mmol, 5 mol%), ligand(*R,S_{Fe}*)-**L8** (3.6 mg, 0.006 mmol, 6 mol%) in 2.5 mL MTBE. The Grignard reagent was added dropwise to the reaction mixture over 2 h using a syringe pump. Product **5e** was obtained as white solid after column chromatography (SiO_2, pentane: Et_2O 80:20), [73% yield, 80% e.e.].

^{1}H NMR (400 MHz, $CDCl_3$) δ 7.47 (dd, *J* = 8.0, 6.0 Hz, 4H), 7.42 – 7.35 (m, 4H), 7.34 – 7.28 (m, 4H), 7.10 – 7.00 (m, 2H), 6.96 (t, *J* = 8.6 Hz, 2H), 6.88 (d, *J* = 7.5 Hz, 2H), 5.03 (s, 1H), 2.65 – 1.99 (m, 2H), 1.35 – 0.94 (m, 4H), 0.70 (t, *J* = 6.9 Hz,3H), 0.61 (s, 3H).

^{13}C NMR (101 MHz, $CDCl_3$) δ 164.7 (d, $J_{C\text{-}F}$ = 253.8 Hz), 141.2, 139.1 (d, $J_{C\text{-}F}$ = 3.0 Hz), 135.9, 135.6, 134.0, 133.6, 123.0, 129.9, 129.7 (d, $J_{C\text{-}F}$ = 9.5 Hz), 128.0, 127.6, 127.5, 126.1, 115.7 (d, $J_{C\text{-}F}$ = 22.6 Hz)., 59.3, 34.9, 27.4, 23.2, 13.9, -4.6.

^{19}F NMR (376 MHz, $CDCl_3$) δ -106.42.

HRMS (ESI+, m/Z): calcd for $C_{30}H_{33}FNO_2SSi$ $[M+H]^+$: 518.1980; found: 518.1970.

The enantiomeric ratio was determined by chiral HPLC analysis, Chiralcel OZH column, n-heptane/i-PrOH 97:30, 0.5 mL/min, 40 °C, detection at 230 nm. Retention times 20.2 min (minor) and 23.0 min (major).

N-(1-(methyldiphenylsilyl)-1-phenylpentyl)thiophene-2-sulfonamide, ***5f***

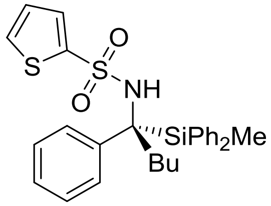

The reaction was performed with 0.1 mmol **4f**, *n*-BuMgBr (0.2 mmol, 2M in Et_2O) diluted with MTBE (1 mL total amount) , CuBr·SMe_2 (1 mg, 0.005 mmol, 5 mol%), ligand(*R,S$_{Fe}$*)-**L8** (3.6 mg, 0.006 mmol, 6 mol%) in 2.5 mL MTBE. The Grignard reagent was added dropwise to the reaction mixture over 2 h using a syringe pump. Product **5f** was obtained as white solid after column chromatography (SiO_2, pentane: Et_2O 80:20), [99% yield, 77% e.e.].

^{1}H NMR (400 MHz, $CDCl_3$) δ 7.51 – 7.46 (m, 2H), 7.45 (dd, *J* = 5.0, 1.4 Hz, 1H), 7.42 – 7.36 (m, 4H), 7.34 – 7.27 (m, 4H), 7.16 (dd, *J* = 3.7, 1.4 Hz, 1H), 7.11 – 7.02 (m, 3H), 6.96 – 6.92 (m, 2H), 6.86 (dd, *J* = 5.0, 3.7 Hz, 1H), 5.12 (s, 1H), 2.40 (dd, *J* = 8.9, 7.4 Hz, 2H), 1.35 – 1.04 (m, 6H), 0.71 (t, *J* = 7.2 Hz, 3H), 0.63 (s, 3H).

^{13}C NMR (101 MHz, $CDCl_3$) δ 144.5, 141.4, 135.9, 135.7, 133.8, 133.6, 132.2, 131.5, 129.9, 129.9, 128.0, 127.97, 127.6, 127.3, 126.8, 126.0, 59.8, 34.7, 27.6, 23.3, 13.9, -4.7.

HRMS (ESI+, m/Z): calcd for $C_{28}H_{32}NO_2S_2Si$ $[M+H]^+$: 506.1638; found:506.1625.

The enantiomeric ratio was determined by chiral HPLC analysis, Chiralcel OZH column, n-heptane/i-PrOH 97:3, 0.5 mL/min, 40 °C, detection at 230 nm. Retention times 38.7 min (major) and 42.3 min (minor).

N-(1-(methyldiphenylsilyl)-1-phenylpentyl)methanesulfonamide, **5g**

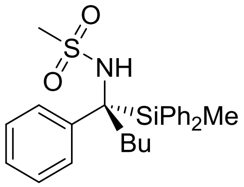

The reaction was performed with 0.1 mmol **4g**, *n*-BuMgBr (0.2 mmol, 2M in Et_2O) diluted with MTBE (1 mL total amount) , CuBr·SMe_2 (1 mg, 0.005 mmol, 5 mol%), ligand(*R,S$_{Fe}$*)-**L8** (3.6 mg, 0.006 mmol, 6 mol%) in 2.5 mL MTBE. The Grignard reagent was added dropwise to the reaction mixture over 2 h using a syringe pump. Product **5g** was obtained as white solid after column chromatography (SiO_2, pentane: Et_2O 80:20), [91% yield, 82% e.e.]

^{1}H NMR (400 MHz, $CDCl_3$) δ 7.61 – 7.56 (m, 2H), 7.47 – 7.16 (m, 11H), 7.12 – 7.04 (m, 2H), 4.83 (s, 1H), 2.66 (s, 3H), 2.56 – 2.32 (m, 2H), 1.52 – 1.19 (m, 4H), 0.80 (t, *J* = 7.2 Hz, 3H), 0.64 (s, 3H).

^{13}C NMR (101 MHz, $CDCl_3$) δ 142.0, 135.8, 135.7, 133.7, 133.4, 130.1, 130.0, 128.1, 128.0, 128.0, 127.3, 126.4, 58.8, 44.2, 35.0, 27.8, 23.5, 14.0, -4.7.

HRMS (ESI+, m/Z): calcd for $C_{31}H_{36}NO_3SSi$ $[M+H]^+$: 438.1918; found: 438.1915.

The enantiomeric ratio was determined by chiral HPLC analysis, Chiralcel OZH column, n-heptane/i-PrOH 97:3, 0.5 mL/min, 40 °C, detection at 230 nm. Retention times 17.6 min (minor) and 21.0 min (major).

3,5-dimethyl-N-(1-(methyldiphenylsilyl)-1-phenylpentyl)benzenesulfonamide, ***5h***

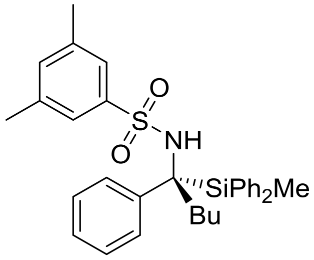

The reaction was performed with 0.1 mmol **4h**, *n*-BuMgBr (0.2 mmol, 2M in Et_2O) diluted with MTBE (1 mL total amount) , $CuBr{\cdot}SMe_2$ (1 mg, 0.005 mmol, 5 mol%), ligand(*R,S_{Fe}*)-**L8** (3.6 mg, 0.006 mmol, 6 mol%) in 2.5 mL MTBE. The Grignard reagent was added dropwise to the reaction mixture over 2 h using a syringe pump. Product **5h** was obtained as white solid after column chromatography (SiO_2, pentane: Et_2O 80:20), [63% yield, 61% e.e.].

^{1}H NMR (400 MHz, $CDCl_3$) δ 7.48 – 7.42 (m, 2H), 7.42 – 7.35 (m, 4H), 7.33 – 7.26 (m, 4H), 7.14 (s, 2H), 7.09 – 7.00 (m, 4H), 6.95 – 6.91 (m, 2H), 4.96 (s, 1H), 2.37 – 2.2 (m, 2H), 2.26 (s, 3H), 1.24 – 0.79 (m, 6H), 0.65 (t, *J* = 7.0 Hz, 3H), 0.60 (s, 3H).

^{13}C NMR (101 MHz, $CDCl_3$) δ 142.8, 141.6, 138.6, 136.0, 135.7, 134.2, 133.8, 133.8, 129.8, 129.8, 127.9, 127.9, 127.5, 127.4, 125.9, 124. 7, 59.3, 34.8, 27.3, 23.2, 21.3, 13.9, -4.7.

HRMS (ESI+, m/Z): calcd for $C_{32}H_{38}NO_2SSi$ $[M+H]^+$: 528.2387; found:528.2373.

The enantiomeric ratio was determined by chiral HPLC analysis, Chiralcel OZH column, n-heptane/i-PrOH 98:2, 0.5 mL/min, 40 °C, detection at 230 nm. Retention times 28.0 min (major) and 33.6min (minor).

2,4,6-trimethyl-N-(1-(methyldiphenylsilyl)-1-phenylpentyl)benzenesulfonamide, ***5i***

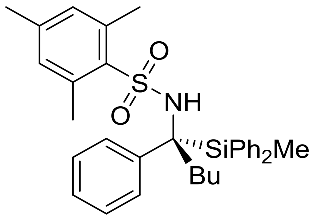

The reaction was performed with 0.1 mmol **4i**, *n*-BuMgBr (0.2 mmol, 2M in Et_2O) diluted with MTBE (1 mL total amount) , CuBr·SMe_2 (1 mg, 0.005 mmol, 5 mol%), ligand(*R,S$_{Fe}$*)-**L8** (3.6 mg, 0.006 mmol, 6 mol%) in 2.5 mL MTBE. The Grignard reagent was added dropwise to the reaction mixture over 2 h using a syringe pump. Product **5i** was obtained as white solid after column chromatography (SiO_2, pentane: Et_2O 80:20), [37% yield, 52% e.e.].

^{1}H NMR (400 MHz, $CDCl_3$) δ 7.50 – 7.47 (m, 2H), 7.42 – 7.36 (m, 4H), 7.34 – 7.28 (m, 4H), 7.05 – 6.96 (m, 1H), 6.94 – 6.82 (m, 4H), 6.78 (s, 2H), 5.06 (s, 1H), 2.55 – 2.45 (m, 1H), 2.38 – 2.29 (m, 1H), 2.26 (s, 3H), 2.25 (s, 6H), 1.30 – 1.03 (m, 6H), 0.81 (t, *J* = 7.1 Hz, 3H), 0.51 (s, 3H).

^{13}C NMR (101 MHz, $CDCl_3$) δ 142.2, 141.5, 138.2, 138.2, 135.8, 135.5, 134.0, 133.6, 131.7, 130.0, 129.9, 128.1, 128.0, 127.4, 127.3, 125.8, 59.7, 37.0, 31.7, 30.0, 26.1, 22.7, 22.7, 21.0, 14.2, -3.8.

HRMS (ESI+, m/Z): calcd for $C_{33}H_{40}NO_2SSi$ $[M+H]^+$: 542.2549; found: 542.25318.

The enantiomeric ratio was determined by chiral HPLC analysis, Chiralcel ODH column, n-heptane/i-PrOH 99:1, 0.5 mL/min, 40 °C, detection at 230 nm. Retention times 14.3 min (minor) and 16.5 min (major).

4-methoxy-N-(1-(methyldiphenylsilyl)-1-phenylpentyl)benzenesulfonamide, ***5j***

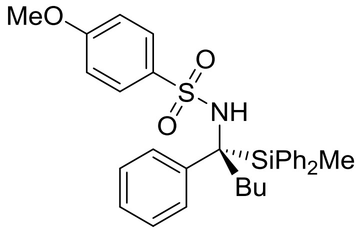

The reaction was performed with 0.1 mmol **4j**, *n*-BuMgBr (0.2 mmol, 2M in Et_2O) diluted with MTBE (1 mL total amount), CuBr·SMe_2 (1 mg, 0.005 mmol, 5 mol%), ligand(*R,S$_{Fe}$*)-**L8** (3.6 mg, 0.006 mmol, 6 mol%) in 2.5 mL MTBE. The Grignard reagent was added dropwise to the reaction mixture over 2 h using a syringe pump. Product **5j** was obtained as white solid after column chromatography (SiO_2, pentane: Et_2O 70:30), [45% yield, 64% e.e.].

^{1}H NMR (400 MHz, $CDCl_3$) δ 7.50 – 7.42 (m, 4H), 7.41 – 7.36 (m, 4H), 7.33 – 7.25 (m, 4H), 7.10 – 7.00 (m, 3H), 6.91 (d, *J* = 7.3 Hz, 2H), 6.81 – 6.75 (m, 2H), 4.94 (s, 1H), 3.83 (s, 3H), 2.47 – 2.14 (m, 2H), 1.26 – 0.86 (m, 6H), 0.68 (t, *J* = 7.0 Hz, 3H), 0.61 (s, 3H).

^{13}C NMR (101 MHz, $CDCl_3$) δ 162.5, 141.6, 135.9, 135.7, 135.0, 134.2, 133.9, 129.8, 129.8, 129.2, 127.9, 127.5, 127.5, 125.9, 113.8, 59.3, 55.7, 34.7, 27.3, 23.3, 13.9, -4.6.

HRMS (ESI+, m/Z): calcd for $C_{31}H_{36}NO_3SSi$ $[M+H]^+$: 530.2180; found:530.2167.

The enantiomeric ratio was determined by chiral HPLC analysis, Chiralcel ODH column, n-heptane/i-PrOH 95:5, 0.5 mL/min, 40 °C, detection at 230 nm. Retention times 19.9 min (minor) and 25.8 min (major).

N-(1-(4-fluorophenyl)-1-(methyldiphenylsilyl)heptyl)-4-methylbenzenesulfonamide, ***6b***

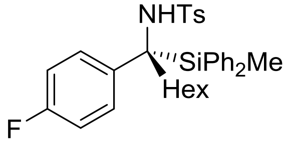

The reaction was performed with 0.1 mmol **1b**, *n*-HexMgBr (0.2 mmol, 2M in Et_2O) diluted with MTBE (1 mL total amount) , $CuBr{\cdot}SMe_2$ (1 mg, 0.005 mmol, 5 mol%), ligand(*R,S$_{Fe}$*)-**L8** (3.6 mg, 0.006 mmol, 6 mol%) in 2.5 mL MTBE. Product **6b** was obtained as white solid after column chromatography (SiO_2, pentane: Et_2O 80:20), [96% yield, 90% e.e.].

^{1}H NMR (400 MHz, $CDCl_3$) δ 7.47 (d, *J* = 7.4 Hz, 1H), 7.45 – 7.36 (m, 6H), 7.35 – 7.28 (m, 4H), 7.13 (d, *J* = 8.0 Hz, 2H), 6.89 – 6.83 (m, 2H), 6.70 (t, *J* = 8.5 Hz, 2H), 4.99 (s, 1H), 2.39 (s, 3H), 2.35 – 2.17 (m, 2H), 1.25 – 0.92 (m, 8H), 0.80 (t, *J* = 7.3 Hz, 3H), 0.60 (s, 3H).

^{13}C NMR (101 MHz, $CDCl_3$) δ 161.2(d, $J_{C\text{-}F}$ = 254.4 Hz), 143.0, 140.2, 137.5(d, $J_{C\text{-}F}$ = 3.1 Hz), 135.8, 135.6, 133.8, 133.5, 130.0, 123.0, 129.3, 129.0(d, $J_{C\text{-}F}$ = 7.8 Hz), 128.0, 127.0, 114.2(d, $J_{C\text{-}F}$ = 21.2 Hz) , 58.9, 35.4, 31.6, 29.8, 25.3, 22.6, 21.6, 14.2, -4.7.

^{19}F NMR (376 MHz MHz, $CDCl_3$) δ=-117.525

HRMS (ESI+, m/Z): calcd for $C_{33}H_{39}FNO_2SSi$ $[M+H]^+$: 560.2449; found:560.2442.

The enantiomeric ratio was determined by chiral HPLC analysis, Chiralcel OZH column, n-heptane/i-PrOH 97:3, 0.5 mL/min, 40 °C, detection at 230 nm. Retention times 27.0min (minor) and 32.3 min (major).

N-(1-(3-fluorophenyl)-1-(methyldiphenylsilyl)heptyl)-4-methylbenzenesulfonamide, ***6c***

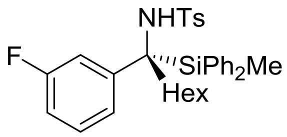

The reaction was performed with 0.1 mmol **1c**, *n*-HexMgBr (0.2 mmol, 2M in Et_2O) diluted with MTBE (1 mL total amount) , CuBr·SMe_2 (1 mg, 0.005 mmol, 5 mol%), ligand(*R,S$_{Fe}$*)-**L8** (3.6 mg, 0.006 mmol, 6 mol%) in 2.5 mL MTBE. Product **6c** was obtained as white solid after column chromatography (SiO_2, pentane: Et_2O 80:20), [88% yield, 92% e.e.].

^{1}H NMR (400 MHz, $CDCl_3$) δ 7.51 – 7.46 (m, 2H), 7.46 – 7.42 (m, 2H), 7.42 – 7.37 (m, 4H), 7.36 – 7.29 (m, 4H), 7.14 (d, *J* = 8.0 Hz, 2H), 7.05 – 6.95 (m, 1H), 6.80 – 6.72 (m, 2H), 6.55 – 6.48 (m, 1H), 4.99 (s, 3H), 2.38 (s, 3H), 2.36 – 2.15 (m, 2H), 1.25 – 0.93 (m, 8H), 0.80 (t, *J* = 7.2 Hz, 3H), 0.60 (s, 3H).

^{13}C NMR (101 MHz, $CDCl_3$) δ 162.3 (d, J_{C-F} = 244.5 Hz), 144.8 (d, J_{C-F} = 6.6 Hz), 143.1, 140.1, 135.8, 135.5, 133.6, 133.3, 130.1, 130.1, 129.3, 128.8 (d, J_{C-F} = 8.3 Hz), 128.1, 128., 127.0, 123.0 (d, J_{C-F} = 2.7 Hz), 114.8 (d, J_{C-F} = 23.3 Hz), 112.7 (d, J_{C-F} = 21.0 Hz), 59.3, 59.3, 35.3, 31.6, 29.8, 25.4, 22.6, 21.6, 14.2, -4.7.

^{19}F NMR (376 MHz MHz, $CDCl_3$) δ=-113.85

HRMS (ESI+, m/Z): calcd for $C_{33}H_{39}FNO_2SSi$ $[M+H]^+$: 560.2449; found:560.2445.

The enantiomeric ratio was determined by chiral HPLC analysis, Chiralcel OZH column, n-heptane/i-PrOH 97:3, 0.5 mL/min, 40 °C, detection at 230 nm. Retention times 27.2min (minor) and 30.5 min (major).

N-(1-(2-fluorophenyl)-1-(methyldiphenylsilyl)heptyl)-4-methylbenzenesulfonamide, ***6d***

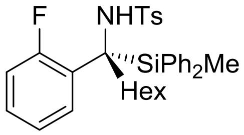

The reaction was performed with 0.1 mmol **1d**, *n*-HexMgBr (0.2 mmol, 2M in Et_2O) diluted with MTBE (1 mL total amount) , CuBr·SMe_2 (1 mg, 0.005 mmol, 5 mol%), ligand(*R,S$_{Fe}$*)-**L8** (3.6 mg, 0.006 mmol, 6 mol%) in 2.5 mL MTBE. Product **6d** was obtained as white solid after column chromatography (SiO_2, pentane: Et_2O 80:20), [87% yield, 70% e.e.].

^{1}H NMR (400 MHz, $CDCl_3$) δ 7.52 – 7.47 (m, 2H), 7.46 – 7.34 (m, 5H), 7.33 – 7.18 (m, 5H), 7.07 – 6.97 (m, 4H), 6.41 (ddd, *J* = 12.7, 6.2, 2.1 Hz, 1H), 5.14 (s, 1H), 2.49 (td, *J* = 13.3, 4.3 Hz, 1H), 2.31 (s, 3H), 2.11 (td, *J* = 14.2, 13.2, 3.9 Hz, 1H), 1.32 – 0.87 (m, 8H), 0.78 (t, *J* = 7.2 Hz, 3H), 0.56 (s, 3H).

^{19}F NMR (376 MHz, $CDCl_3$) δ -104.40.

^{13}C NMR (101 MHz, $CDCl_3$) δ 160.1 (d, J_{C-F} = 245.8 Hz), 142.6, 139.3, 135.7, 135.4, 134.2, 133.4, 129.9 (d, J_{C-F}= 10.1 Hz), 128.9, 128.4 (d, J_{C-F} = 3.2 Hz), 128.2, 128.1, 128.0, 127.9, 127.2, 123.9 (d, J_{C-F}= 2.0 Hz), 115.9 (d, J_{C-F}= 24.3 Hz)., 56.7, 35.2, 31.6, 29.7, 26.0, 22.7, 21.5, 14.2, -4.0.

HRMS (ESI+, m/Z): calcd for $C_{33}H_{39}FNO_2SSi$ $[M+H]^+$: 560.2449; found: 560.2445.

The enantiomeric ratio was determined by chiral HPLC analysis, Chiralcel ODH column, n-heptane/i-PrOH 98:2, 0.5 mL/min, 40 °C, detection at 230 nm. Retention times 36.3min (major) and 37.2 min (minor).

N-(1-(4-chlorophenyl)-1-(methyldiphenylsilyl)heptyl)-4-methylbenzenesulfonamide, ***6e***

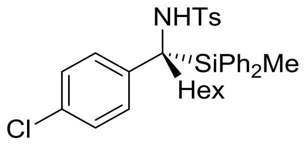

The reaction was performed with 0.1 mmol **1e**, *n*-HexMgBr (0.2 mmol, 2M in Et_2O) diluted with MTBE (1 mL total amount) , $CuBr·SMe_2$ (1 mg, 0.005 mmol, 5 mol%), ligand(*R*,S_{Fe})-**L8** (3.6 mg, 0.006 mmol, 6 mol%) in 2.5 mL MTBE. Product **6e** was obtained as white solid after column chromatography (SiO_2, pentane: Et_2O 80:20), [97% yield, 91% e.e.].

Note: The reaction was also performed on a preparative scale (1mmol) following the general procedure. The product **6f** was obtained with 90% yield, 91% e.e.

^{1}H NMR (400 MHz, $CDCl_3$) δ 7.48 (d, *J* = 6.9 Hz, 2H), 7.44 – 7.37 (m, 6H), 7.32 (t, *J* = 7.2 Hz, 4H), 7.13 (d, *J* = 8.1 Hz, 2H), 6.98 (d, *J* = 8.6 Hz, 2H), 6.82 (d, *J* = 8.7 Hz, 2H), 5.00 (s, 1H), 2.39 (s, 3H), 2.36 – 2.15 (m, 2H), 1.22 – 0.85 (m, 8H), 0.80 (t, *J* = 7.2 Hz, 3H), 0.59 (s, 3H).

^{13}C NMR (101 MHz, $CDCl_3$) δ 143.1, 140.4, 140.1, 135.8, 135.6, 133.6, 133.3, 131.7, 130.1, 130.1, 129.3, 128.8, 128.1, 128.08, 127.5, 127.0, 59.0, 35.3, 31.6, 29.8, 25.4, 22.6, 21.6, 14.1, -4.7.

HRMS (ESI+, m/Z): calcd for $C_{33}H_{39}ClNO_2SSi$ $[M+H]^+$: 576.2154; found:576.2149.

The enantiomeric ratio was determined by chiral HPLC analysis, Chiralcel OZH column, n-heptane/i-PrOH 97:3, 0.5 mL/min, 40 °C, detection at 230 nm. Retention times 24.1 min (minor) and 27.5 min (major).

*N-(1-(3-chlorophenyl)-1-(methyldiphenylsilyl)heptyl)-4-methylbenzenesulfonamide, **6f***

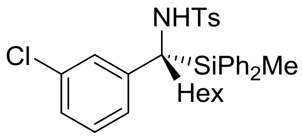

The reaction was performed with 0.1 mmol **1f**, *n*-HexMgBr (0.2 mmol, 2M in Et_2O) diluted with MTBE (1 mL total amount) , CuBr·SMe_2 (1 mg, 0.005 mmol, 5 mol%), ligand(R,S_{Fe})-**L8** (3.6 mg, 0.006 mmol, 6 mol%) in 2.5 mL MTBE. Product **6f** was obtained as white solid after column chromatography (SiO_2, pentane: Et_2O 80:20), [91% yield, 94% e.e.].

Note: The reaction was also performed with reduced catalyst loading (1 mol% of CuBr·SMe_2, 1.1 mol% of **L8**) following the general procedure with 3 h of addition time. The product **6e** was obtained with 68% yield, 90% e.e.

^{1}H NMR (400 MHz, $CDCl_3$) δ 7.48 (dd, *J* = 8.0, 1.4 Hz, 2H), 7.43 – 7.29 (m, 10H), 7.12 (d, *J*= 8.2 Hz, 2H), 7.02 (dt, *J* = 8.0, 1.6 Hz,1H), 6.98 (t, *J* = 7.8 Hz, 1H), 6.86 (dt, *J* = 7.5, 1.6 Hz, 1H), 4.97 (s, 1H), 2.37 (s, 3H), 2.37 – 2.29 (m, 2H), 1.17 – 0.90 (m, 8H), 0.76 (t, *J* = 7.2 Hz, 3H), 0.54 (s, 3H).

^{13}C NMR (101 MHz, $CDCl_3$) δ 143.8, 143.1, 140.0, 135.7, 135.5, 133.5, 133.5, 133.1, 130.1, 130.1, 129.4, 128.6, 128.2, 128.1, 128.1, 126.9, 125.9, 125.4, 59.1, 35.3, 31.6, 29.8, 25.4, 22.6, 21.6, 14.1, -4.8.

HRMS (ESI+, m/Z): calcd for $C_{33}H_{39}ClNO_2SSi$ $[M+H]^+$: 576.2154; found:576.2147.

The enantiomeric ratio was determined by chiral HPLC analysis, Chiralcel OZH column, n-heptane/i-PrOH 97:3, 0.5 mL/min, 40 °C, detection at 230 nm. Retention times 26.0 min (minor) and 30.5 min (major).

*N-(1-(4-bromophenyl)-1-(methyldiphenylsilyl)heptyl)-4-methylbenzenesulfonamide, **6g***

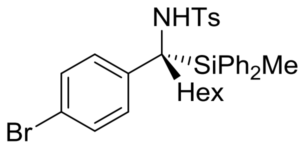

The reaction was performed with 0.1 mmol **1g**, *n*-HexMgBr (0.2 mmol, 2M in Et_2O) diluted with MTBE (1 mL total amount) , CuBr·SMe_2 (1 mg, 0.005 mmol, 5 mol%), ligand(*R,S$_{Fe}$*)-**L8** (3.6 mg, 0.006 mmol, 6 mol%) in 2.5 mL MTBE. Product **6g** was obtained as white solid after column chromatography (SiO_2, pentane: Et_2O 80:20), [89% yield, 90% e.e.].

^{1}H NMR (400 MHz, $CDCl_3$) δ 7.48 (d, *J* = 6.8 Hz, 2H), 7.45 – 7.36 (m, 6H), 7.32 (t, *J* = 6.8 Hz, 4H), 7.15 – 7.10 (m, 4H), 6.76 (d, *J* = 8.7 Hz, 2H), 5.00 (s, 1H), 2.40 (s, 3H), 2.35 – 2.15 (m,2H), 1.25 – 0.93 (m, 8H), 0.80 (t, *J* = 7.2 Hz, 3H), 0.59 (s, 3H).

^{13}C NMR (101 MHz, $CDCl_3$) δ 143.1, 141.0, 140.1, 135.8, 135.5, 133.6, 133.3, 130.4, 130.1, 130.1, 129.3, 129.2, 128.1, 128.1, 127.0, 119.9, 59.0, 35.2, 31.6, 29.8, 25.4, 22.6, 21.6, 14.1, -4.7.

HRMS (ESI+, m/Z): calcd for $C_{33}H_{39}BrNO_2SSi$ $[M+H]^+$: 620.1649; found:620.1646.

The enantiomeric ratio was determined by chiral HPLC analysis, Chiralcel OZH column, n-heptane/i-PrOH 97:3, 0.5 mL/min, 40 °C, detection at 230 nm. Retention times 26.8min (minor) and 29.6 min (major).

N-(1-(3-bromophenyl)-1-(methyldiphenylsilyl)heptyl)-4-methylbenzenesulfonamide, ***6h***

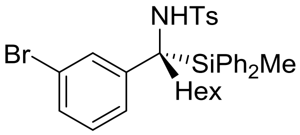

The reaction was performed with 0.1 mmol **1h**, *n*-HexMgBr (0.2 mmol, 2M in Et_2O) diluted with MTBE (1 mL total amount) , CuBr·SMe_2 (1 mg, 0.005 mmol, 5 mol%), ligand(*R,S$_{Fe}$*)-**L8** (3.6 mg, 0.006 mmol, 6 mol%) in 2.5 mL MTBE. Product **6h** was obtained as white solid after column chromatography (SiO_2, pentane: Et_2O 80:20), [90 % yield, 93% e.e.].

^{1}H NMR (400 MHz, $CDCl_3$) δ 7.49 (d, *J* = 6.7 Hz, 2H), 7.41 (d, *J* = 8.0 Hz, 2H), 7.38 – 7.29 (m, 6H), 7.20 – 7.15 (m, 2H), 7.12 (d, *J* = 8.1 Hz, 2H), 6.95 – 6.90 (m, 2H), 6.78 (s, 3H), 5.00 (s, 3H), 2.37 (s, 3H), 2.37 – 2.30 (m, 1H), 2.27 – 2.16 (m, 1H), 1.35 – 0.98 (m, 8H), 0.82 (t, *J* = 7.2 Hz, 3H), 0.60 (s, 3H).

^{13}C NMR (101 MHz, $CDCl_3$) δ 144.0, 143.1, 140.0, 135.8, 135.5, 133.5, 133.1, 131.1, 130.2, 130.2, 129.5, 129.0, 128.9, 128.2, 128.1, 126.9, 125.8, 121.9, 59.0, 35.4, 31.6, 29.8, 25.5, 22.6, 21.6, 14.2, -4.8.

HRMS (ESI+, m/Z): calcd for $C_{33}H_{39}BrNO_2SSi$ $[M+H]^+$: 620.1649; found: 620.1643.

The enantiomeric ratio was determined by chiral HPLC analysis, Chiralcel OZH column, n-heptane/i-PrOH 97:3, 0.5 mL/min, 40 °C, detection at 236 nm. Retention times 27.0 min (minor) and 29.9 min (major).

*4-methyl-N-(1-(methyldiphenylsilyl)-1-(4-(trifluoromethyl)phenyl)heptyl)benzenesulfonamide, **6i***

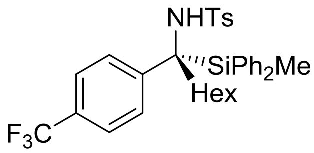

The reaction was performed with 0.1 mmol **1i**, *n*-HexMgBr (0.2 mmol, 2M in Et_2O) diluted with MTBE (1 mL total amount) , CuBr·SMe_2 (1 mg, 0.005 mmol, 5 mol%), ligand(*R,S$_{Fe}$*)-**L8** (3.6 mg, 0.006 mmol, 6 mol%) in 2.5 mL MTBE. Product **6i** was obtained as white solid after column chromatography (SiO_2, pentane: Et_2O 80:20), [89% yield, 93% e.e.].

^{1}H NMR (400 MHz, $CDCl_3$) δ 7.52 – 7.46 (m, 2H), 7.45 – 7.38 (m, 4H), 7.38 – 7.28 (m, 6H), 7.24 (d, *J* = 8.3 Hz, 2H), 7.11 (d, *J* = 8.0 Hz, 2H), 7.00 (d, *J* = 8.2 Hz, 2H), 5.08 (s, 1H), 2.45 – 2.16 (m, 2H), 2.37 (s, 3H), 1.33 – 0.90 (m, 8H), 0.81 (t, *J* = 7.2 Hz, 3H), 0.59 (s, 3H).

^{13}C NMR (101 MHz, $CDCl_3$) δ 146.2 (c, J_{C-F} = 1.2 Hz), 143.2, 140.0, 135.7, 135.5, 133.3, 133.0, 130.2, 130.2, 129.4, 128.2, 128.2, 127.9 (c, J_{C-F} = 32.4 Hz), 127.7, 127.0, 124.3 (c, J_{C-F} = 271.8 Hz), 124.2 (c, J_{C-F} = 3.7 Hz), 59.3, 35.4, 31.6, 29.8, 25.6, 22.6, 21.5, 14.1, -4.8.

^{19}F NMR (376 MHz, $CDCl_3$) δ -62.37.

HRMS (ESI+, m/Z): calcd for $C_{34}H_{39}F_3NO_2SSi$ $[M+H]^+$: 610.2417; found:610.2411.

The enantiomeric ratio was determined by chiral HPLC analysis, Chiralcel ODH column, n-heptane/i-PrOH 99:1, 0.5 mL/min, 40 °C, detection at 230 nm. Retention times 47.7 min (major) and 55.7 min (minor).

*4-methyl-N-(1-(methyldiphenylsilyl)-1-(3-(trifluoromethyl)phenyl)heptyl)benzenesulfonamide, **6j***

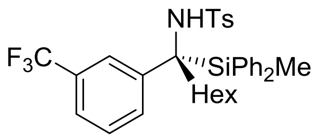

The reaction was performed with 0.1 mmol **1j**, *n*-HexMgBr (0.2 mmol, 2M in Et_2O) diluted with MTBE (1 mL total amount) , CuBr·SMe_2 (1 mg, 0.005 mmol, 5 mol%), ligand(*R,S$_{Fe}$*)-**L8** (3.6 mg,

0.006 mmol, 6 mol%) in 2.5 mL MTBE. Product **6j** was obtained as white solid after column chromatography (SiO_2, pentane: Et_2O 80:20), [77% yield, 79% e.e.].

^{1}H NMR (400 MHz, $CDCl_3$) δ 7.48 (d, *J* = 8.0 Hz, 2H), 7.44 – 7.28 (m, 11H), 7.22 – 7.18 (m, 2H), 7.12 (d, *J* = 8.0 Hz, 2H), 6.97 (s, 1H), 5.06 (s, 1H), 2.45 – 2.22 (m, 2H), 2.37 (s, 3H), 1.33 – 0.99 (m, 8H), 0.82 (t, *J* = 7.2 Hz, 3H), 0.60 (s, 3H).

^{13}C NMR (101 MHz, $CDCl_3$) δ 143.2, 142.8, 140.0, 135.6, 135.4, 134.1, 133.2, 132.8, 130.5 (q, J_{C-F} = 1.4 Hz), 130.2, 129.5 (q, J_{C-F} = 31.9 Hz), 129.4, 128.2, 128.2, 128.0, 126.8, 124.5 (q, J_{C-F} = 3.9 Hz), 124.1 (q, J_{C-F} = 272.9 Hz), 122.7 (q, J_{C-F} = 3.7 Hz), 59.3, 35.4, 31.6, 29.8, 25.5, 22.6, 21.5, 14.1, -4.9.

^{19}F NMR (376 MHz, $CDCl_3$) δ -62.72.

HRMS (ESI+, m/Z): calcd for $C_{34}H_{39}F_3NO_2SSi$ $[M+H]^+$: 610.2417; found:610.2409.

The enantiomeric ratio was determined by chiral HPLC analysis, Chiralcel OZH column, n-heptane/i-PrOH 97:3, 0.5 mL/min, 40 °C, detection at 230 nm. Retention times 34.6 min (minor) and 38.5 min (major).

230 nm. Retention times 34.6 min (minor) and 38.5 min (major).

*4-methyl-N-(1-(methyldiphenylsilyl)-1-(p-tolyl)heptyl)benzenesulfonamide, **6k***

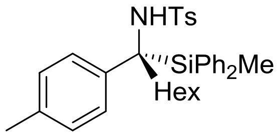

The reaction was performed with 0.1 mmol **1k**, *n*-HexMgBr (0.2 mmol, 2M in Et_2O) diluted with MTBE (1 mL total amount) , CuBr·SMe_2 (1 mg, 0.005 mmol, 5 mol%), ligand(*R,S*$_{Fe}$)-**L8** (3.6 mg, 0.006 mmol, 6 mol%) in 2.5 mL MTBE. Product **6k** was obtained as white solid after column chromatography (SiO_2, pentane: Et_2O 80:20), [83% yield, 87% e.e.].

^{1}H NMR (400 MHz, $CDCl_3$) δ 7.50 – 7.45 (m, 2H), 7.44 – 7.35 (m, 6H), 7.34 – 7.27 (m, 2H), 7.11 (d, *J* = 8.1 Hz, 2H), 6.85 (d, *J* = 8.4 Hz, 2H), 6.81 (d, *J* = 8.4 Hz, 2H), 4.96 (s,3H), 2.38 (s, 3H), 2.30 – 2.22 (m, 5H), 1.24 – 0.85 (m, 8H), 0.80 (t, *J* = 7.3 Hz, 3H), 0.61 (s, 3H).

^{13}C NMR (101 MHz, $CDCl_3$) δ 142.7, 140.3, 138.60, 136.0, 135.7, 135.4, 134.3, 134.0, 129.8, 129.7, 129.2, 128.2, 127.9, 127.8, 127.3, 127.1, 59.1, 35.0, 31.6, 29.8, 25.2, 22.6, 21.5, 21.0, 14.2, -4.6.

HRMS (ESI+, m/Z): calcd for $C_{34}H_{42}NO_2SSi$ $[M+H]^+$: 556.2700; found:556.2693.

The enantiomeric ratio was determined by chiral HPLC analysis, Chiralcel OZH column, n-heptane/i-PrOH 98:2, 0.5 mL/min, 40 °C, detection at 230 nm. Retention times 45.0 min (minor) and 48.1 min (major).

*4-methyl-N-(1-(methyldiphenylsilyl)-1-(m-tolyl)heptyl)benzenesulfonamide, **6l***

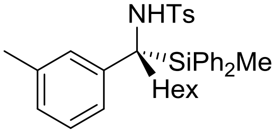

The reaction was performed with 0.1 mmol **1l**, *n*-HexMgBr (0.2 mmol, 2M in Et_2O) diluted with MTBE (1 mL total amount) , CuBr·SMe_2 (1 mg, 0.005 mmol, 5 mol%), ligand(*R,S_{Fe}*)-**L8** (3.6 mg, 0.006 mmol, 6 mol%) in 2.5 mL MTBE. Product **6l** was obtained as white solid after column chromatography (SiO_2, pentane: Et_2O 80:20), [82% yield, 84% e.e.].

^{1}H NMR (400 MHz, $CDCl_3$) δ 7.47 (d, *J* = 6.8 Hz, 2H), 7.43 – 7.35 (m, 6H), 7.34 – 7.27 (m, 4H), 7.10 (d, *J* = 8.0 Hz, 2H), 6.98 (t, *J* = 7.7 Hz, 1H), 6.86 (d, *J* = 7.4 Hz, 1H), 6.82 (d, *J* = 7.9 Hz, 1H), 6.46 (s, 1H), 2.36 (s, 3H), 2.34 – 2.16 (m, 2H), 1.97 (s, 3H), 1.31 – 1.21 (m, 2H), 1.22 – 0.85 (m, 8H), 0.81 (t, *J* = 7.2 Hz, 3H), 0.61 (s, 3H).

^{13}C NMR (101 MHz, $CDCl_3$) δ 142.7, 141.1, 140.3, 136.6, 135.9, 135.6, 134.3, 133.9, 129.8, 129.8, 129.2, 129.0, 127.8, 127.8, 127.5, 127.0, 126.6, 124.2, 59.3, 35.1, 31.6, 29.8, 25.2, 22.7, 21.5, 21.4, 14.2, -4.8.

HRMS (ESI+, m/Z): calcd for $C_{34}H_{42}NO_2SSi$ $[M+H]^+$: 556.2700; found:556.2692.

The enantiomeric ratio was determined by chiral HPLC analysis, Chiralcel ADH column, n-heptane/i-PrOH 98:2, 0.5 mL/min, 40 °C, detection at 230 nm. Retention times 24.0min (minor) and 32.0 min (major).

*N-(1-(4-methoxyphenyl)-1-(methyldiphenylsilyl)heptyl)-4-methylbenzenesulfonamide, **6m***

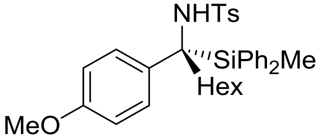

The reaction was performed with 0.1 mmol **1m**, *n*-HexMgBr (0.2 mmol, 2M in Et_2O) diluted with MTBE (1 mL total amount) , CuBr·SMe_2 (1 mg, 0.005 mmol, 5 mol%), ligand(*R,S_{Fe}*)-**L8** (3.6 mg,

0.006 mmol, 6 mol%) in 2.5 mL MTBE. Product **6m** was obtained as white solid after column chromatography (SiO_2, pentane: Et_2O 70:30), [79% yield, 82% e.e.].

^{1}H NMR (400 MHz, $CDCl_3$) δ 7.47 (d, *J* = 6.8 Hz, 2H), 7.43 – 7.35 (m, 6H), 7.34 – 7.27 (m, 4H), 7.11 (d, *J* = 8.0 Hz, 2H), 6.82 (d, *J* = 8.9 Hz, 2H), 6.56 (d, *J* = 8.9 Hz, 2H), 3.75 (s, 3H), 2.37 (s, 3H), 2.33 – 2.15 (m, 2H), 1.24 – 0.87 (m, 8H), 0.79 (t, *J* = 7.2 Hz, 3H), 0.61 (s, 3H).

^{13}C NMR (101 MHz, $CDCl_3$) δ 157.8, 142.7, 140.3, 136.0, 135.7, 134.4, 134.0, 133.6, 129.8, 129.7, 129.2, 128.6, 127.9, 127.9, 127.1, 112.9, 58.7, 55.3, 35.2, 31.6, 29.8, 25.2, 22.7, 21.6, 14.2, -4.6.

HRMS (ESI+, m/Z): calcd for $C_{34}H_{42}NO_3SSi$ $[M+H]^+$: 572.2649; found:572.2644.

The enantiomeric ratio was determined by chiral HPLC analysis, Chiralcel ODH column, n-heptane/i-PrOH 98:2, 0.5 mL/min, 40 °C, detection at 230 nm. Retention times 28.5 min (major) and 36.4 min (minor).

N-(1-(3-methoxyphenyl)-1-(methyldiphenylsilyl)heptyl)-4-methylbenzenesulfonamide, ***6n***

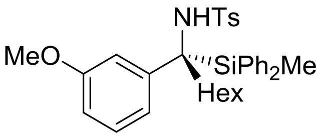

The reaction was performed with 0.1 mmol **1n**, *n*-HexMgBr (0.2 mmol, 2M in Et_2O) diluted with MTBE (1 mL total amount) , CuBr·SMe_2 (1 mg, 0.005 mmol, 5 mol%), ligand(*R,S$_{Fe}$*)-**L8** (3.6 mg, 0.006 mmol, 6 mol%) in 2.5 mL MTBE. Product **6n** was obtained as white solid after column chromatography (SiO_2, pentane: Et_2O 70:30), [72% yield, 82% e.e.].

^{1}H NMR (400 MHz, $CDCl_3$) δ 7.49 (d, *J* = 7.4 Hz, 2H), 7.45 – 7.35 (m, 6H), 7.34 – 7.27 (m, 4H), 7.11 (d, *J* = 7.9 Hz, 2H), 7.01 (t, *J* = 8.0 Hz, 1H), 6.66 – 6.59 (m, 2H), 6.30 (s, 1H), 4.98 (s, 1H), 3.33 (s, 3H), 2.36 (s, 3H), 2.34 – 2.17 (m, 2H), 1.25 – 0.87 (m, 8H), 0.80 (t, *J* = 7.2 Hz, 3H), 0.63 (s, 3H).

^{13}C NMR (101 MHz, $CDCl_3$) δ 158.7, 143.2, 142.8, 140.3, 135.9, 135.6, 134.3, 133.9, 129.8, 129.2, 128.5, 128.0, 127.9, 127.1, 119.7, 113.2, 112.4, 59.5, 54.7, 35.2, 31.6, 29.8, 25.3, 22.7, 21.5, 14.2, -4.6.

HRMS (ESI+, m/Z): calcd for $C_{34}H_{42}NO_3SSi$ $[M+H]^+$: 572.2649; found:572.2644.

The enantiomeric ratio was determined by chiral HPLC analysis, Chiralcel OZH column, n-heptane/i-PrOH 97:3, 0.5 mL/min, 40 °C, detection at 230 nm. Retention times 40.4 min (major) and 43.8 min (minor).

N-(1-(4-(tert-butyl)phenyl)-1-(methyldiphenylsilyl)heptyl)-4-methylbenzenesulfonamide, ***6o***

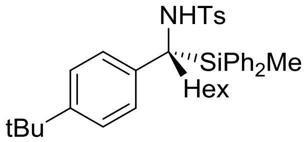

The reaction was performed with 0.1 mmol **1o**, *n*-HexMgBr (0.2 mmol, 2M in Et_2O) diluted with MTBE (1 mL total amount) , CuBr·SMe_2 (1 mg, 0.005 mmol, 5 mol%), ligand(*R,S$_{Fe}$*)-**L8** (3.6 mg, 0.006 mmol, 6 mol%) in 2.5 mL MTBE. Product **6o** was obtained as white solid after column chromatography (SiO_2, pentane: Et_2O 80:20), [87% yield, 91% e.e.].

^{1}H NMR (400 MHz, $CDCl_3$) δ 7.48 – 7.41 (m, 2H), 7.38 – 7.33 (m, 6H), 7.31 – 7.24 (m, 4H), 7.06 (d, *J* = 8.0 Hz, 2H), 7.00 (d, *J* = 8.5 Hz, 2H), 6.82 (d, *J* = 8.6 Hz, 2H), 4.97 (s, 1H), 2.35 (s, 3H), 2.32 – 2.19 (m, 2H), 1.27 (s, 9H), 1.15 – 0.88 (m, 8H), 0.80 (t, *J* = 7.3 Hz, 3H), 0.63 (s, 3H).

^{13}C NMR (101 MHz, $CDCl_3$) δ 148.8, 142.6, 140.2, 138.3, 136.0, 135.7, 134.7, 134.3, 129.6, 129.6, 129.2, 127.8, 127.78, 127.2, 127.1, 124.4, 58.9, 34.8, 34.3, 31.7, 31.5, 29.8, 25.1, 22.7, 21.6, 14.2, -4.5.

HRMS (ESI+, m/Z): calcd for $C_{37}H_{48}NO_2SSi$ $[M+H]^+$: 598.3170; found:598.3165.

The enantiomeric ratio was determined by chiral HPLC analysis, Chiralcel OZH column, n-heptane/i-PrOH 97:3, 0.5 mL/min, 40 °C, detection at 230 nm. Retention times 18.4 min (major) and 28.2 min (minor).

4-methyl-N-(1-(methyldiphenylsilyl)-1-(4-(trifluoromethoxy)phenyl)heptyl)benzenesulfonamide, ***6p***

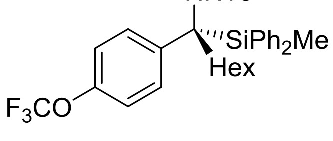

The reaction was performed with 0.1 mmol **1p**, *n*-HexMgBr (0.2 mmol, 2M in Et_2O) diluted with MTBE (1 mL total amount) , CuBr·SMe_2 (1 mg, 0.005 mmol, 5 mol%), ligand(*R,S$_{Fe}$*)-**L8** (3.6 mg, 0.006 mmol, 6 mol%) in 2.5 mL MTBE. Product **6p** was obtained as white solid after column chromatography (SiO_2, pentane: Et_2O 80:20), [91% yield, 90% e.e.].

^{1}H NMR (400 MHz, $CDCl_3$) δ 7.49 – 7.45 (m, 2H), 7.44 – 7.36 (m, 4H), 7.37 – 7.27 (m, 6H), 7.12 (d, *J* = 8.0 Hz, 2H), 6.89 (d, *J* = 9.0 Hz, 2H), 6.83 (d, *J* = 8.6 Hz, 2H), 5.03 (s, 1H), 2.37 (s, 3H), 2.36 – 2.15 (m, 2H), 1.32 – 0.90 (m, 8H), 0.81 (t, *J* = 7.2 Hz, 3H), 0.61 (s, 3H).

^{19}F NMR (376 MHz, $CDCl_3$) δ -57.87.

^{13}C NMR (101 MHz, $CDCl_3$) δ 155.4, 147.1, 143.0, 140.4, 139.9, 135.6, 135.4, 133.5, 133.1, 129.9, 129.2, 128.6, 128.0, 127.9, 126.9, 119.6, 58.8, 35.2, 31.4, 29.7, 25.3, 22.5, 21.4, 14.0, -4.9.

HRMS (ESI+, m/Z): calcd for $C_{34}H_{39}F_3NO_3SSi$ $[M+H]^+$: 626.2367; found:626.2360.

The enantiomeric ratio was determined by chiral HPLC analysis, Chiralcel ODH column, n-heptane/i-PrOH 99:1, 0.5 mL/min, 40 °C, detection at 230 nm. Retention times 40.2 min (major) and 51.9 min (minor).

N-(1-(dimethyl(phenyl)silyl)-1-(3-(trifluoromethoxy)phenyl)heptyl)-4-methylbenzenesulfonamide, ***6q***

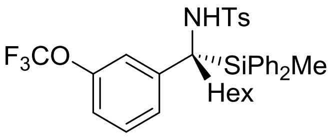

The reaction was performed with 0.1 mmol **1q**, *n*-HexMgBr (0.2 mmol, 2M in Et_2O) diluted with MTBE (1 mL total amount) , $CuBr·SMe_2$ (1 mg, 0.005 mmol, 5 mol%), ligand(*R,S$_{Fe}$*)-**L8** (3.6 mg, 0.006 mmol, 6 mol%) in 2.5 mL MTBE. Product **6q** was obtained as white solid after column chromatography (SiO_2, pentane: Et_2O 80:20), [72% yield, 86% e.e.].

^{1}H NMR (400 MHz, $CDCl_3$) δ 7.50 – 7.35 (m, 8H), 7.36 – 7.29 (m, 4H), 7.14 (d, *J* = 7.9 Hz, 2H), 7.04 (t, *J* = 8.0 Hz, 1H), 6.93 (d, *J* = 8.2 Hz, 1H), 6.85 (d, *J* = 8.0 Hz, 1H), 6.74 (s, 1H), 5.01 (s, 1H), 2.38 (s, 3H), 2.36 – 2.14 (m, 2H), 1.23 – 0.93 (m, 8H), 0.81 (t, *J* = 7.2 Hz, 3H), 0.60 (s, 3H).

^{19}F NMR (376 MHz, $CDCl_3$) δ -57.58.

^{13}C NMR (101 MHz, $CDCl_3$) δ 148.8, 144.7, 143.1, 140.1, 135.7, 135.5, 133.2, 133.0, 130.2, 130.1, 129.4, 128.6, 128.1, 126.9, 125.9, 120.3, 118.1, 59.2, 35.3, 31.6, 29.8, 25.4, 22.6, 21.5, 14.2, -4.8.

HRMS (ESI+, m/Z): calcd for $C_{34}H_{39}F_3NO_3SSi$ $[M+H]^+$: 626.2367; found: 626.2351.

The enantiomeric ratio was determined by chiral HPLC analysis, Chiralcel OZH column, n-heptane/i-PrOH 97:3, 0.5 mL/min, 40 °C, detection at 230 nm. Retention times 17.8 min (minor) and 19.4 min (major).

4-methyl-N-(1-(methyldiphenylsilyl)-1-(naphthalen-2-yl)heptyl)benzenesulfonamide, ***6r***

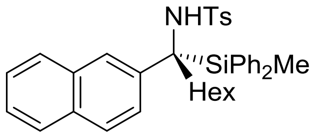

The reaction was performed with 0.1 mmol **1r**, *n*-HexMgBr (0.2 mmol, 2M in Et_2O) diluted with MTBE (1 mL total amount) , CuBr·SMe_2 (1 mg, 0.005 mmol, 5 mol%), ligand(R,S_{Fe})-**L8** (3.6 mg, 0.006 mmol, 6 mol%) in 2.5 mL MTBE. Product **6r** was obtained as white solid after column chromatography (SiO_2, pentane: Et_2O 80:20), [99% yield, 87% e.e.].

^{1}H NMR (400 MHz, $CDCl_3$) δ 7.76 – 7.69 (m, 1H), 7.50 (d, *J* = 7.3 Hz, 2H), 7.47 – 7.33 (m, 9H), 7.33 – 7.25 (m, 5H), 7.02 (d, *J* = 8.8, 1H), 6.97 (d, *J* = 8.0 Hz, 2H), 5.13(s, 1H), 2.53 – 2.33 (m, 2H), 2.26 (s, 3H), 1.34 – 0.99 (m, 8H), 0.82 (t, *J* = 7.2 Hz, 3H), 0.62 (s, 3H).

^{13}C NMR (101 MHz, $CDCl_3$) δ 142.8, 140.1, 138.9, 135.9, 135.6, 134.1, 133.7, 132.7, 131.7, 129.9, 129.9, 129.1, 128.1, 128.0, 127.2, 127.0, 126.8, 126.3, 126.0, 125.7, 125.7, 59.2, 35.3, 31.6, 29.9, 25.5, 22.7, 21.4, 14.2, -4.7.

HRMS (ESI+, m/Z): calcd for $C_{37}H_{42}NO_2SSi$ $[M+H]^+$: 592.2700; found:592.2696.

The enantiomeric ratio was determined by chiral HPLC analysis, Chiralcel OZH column, n-heptane/i-PrOH 96:4, 0.5 mL/min, 40 °C, detection at 230 nm. Retention times 24.9 min (minor) and 30.4 min (major).

4-methyl-N-(1-(methyldiphenylsilyl)-1-(thiophen-3-yl)heptyl)benzenesulfonamide, **6s**

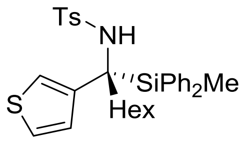

The reaction was performed with 0.1 mmol **1s**, HexMgBr (0.2 mmol, 2M in Et_2O) diluted with Et_2O (1 ml total amount) , CuBr·SMe_2 (1 mg, 0.005 mmol, 5 mol%), ligand(R,S_{Fe})-L6 (3.6 mg, 0.006 mmol, 6 mol%) in 2.5 ml tBuOMe. Product 6s was obtained as white solid after column chromatography (SiO_2, pentane: Et_2O 80:20), [60% yield, 60% e.e.].

^{1}H NMR (400 MHz, Chloroform-*d*) δ 7.53 – 7.50 (m, 2H), 7.41– 7.37 (m, 6H), 7.31 (m, 4H), 7.09 (d, *J* = 8.1 Hz, 2H), 6.88 (dd, *J* = 5.0, 3.0 Hz, 1H), 6.66 (dd, *J* = 2.8, 1.2 Hz, 1H), 6.35 (dd, *J* = 5.1, 1.2 Hz, 1H), 4.94 (s, 1H), 2.36 (s, 3H), 2.34 – 2.17 (m, 2H), 1.33 – 0.94 (m, 8H), 0.80 (t, *J* = 7.2 Hz, 3H), 0.65 (s, 3H).

^{13}C NMR (101 MHz, cdcl$_3$) δ 143.6, 142.8, 140.0, 135.8, 135.5, 134.4, 133.9, 129.9, 129.9, 129.2, 128.0, 128.0, 127.1, 124.0, 120.8, 57.4, 36.1, 31.6, 29.8, 25.5, 22.6, 21.6, 14.2, -4.6.

HRMS (ESI+, m/Z): calcd for $C_{31}H_{37}NO_2S_2SiNa$ [M+Na]$^+$: 570,19272; found: 570,19135.

The enantiomeric ratio was determined by chiral HPLC analysis, Chiracel-OZH column, *n*-heptane/*i*-PrOH 97:3, 0.5ml/min, 40 °C, detection at 230 nm. Retention times: 35.2 min (major) and 37.3 min (minor).

4-methyl-N-(4-phenyldecan-4-yl)benzenesulfonamide **6t**

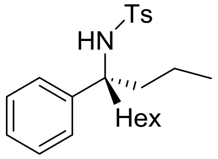

The reaction was performed with 0.1 mmol **1t**, HexMgBr (0.2 mmol, 2M in Et_2O) diluted with Et_2O (1 ml total amount) , CuBr·SMe$_2$ (1 mg, 0.005 mmol, 5 mol%), ligand(R,S$_{Fe}$)-**L1** (3.6 mg, 0.006 mmol, 6 mol%) in 0.5 ml tBuOMe and 0.5ml of Et_2O. Product **6t** was obtained as white solid after column chromatography (SiO_2, pentane: Et_2O 10:1), [89% yield, 64% e.e.].

^{1}H NMR (400 MHz, Chloroform-*d*) δ 7.51 (d, *J* = 8.3 Hz, 2H), 7.21 – 7.12 (m, 7H), 4.74 (s, 1H), 2.38 (s, 3H), 2.01 – 1.95 (m, 2H), 1.90 – 1.82 (m, 2H), 1.20 – 0.91 (m, 10H), 0.82 (t, *J* = 7.1 Hz, 3H), 0.78 (t, *J* = 7.3 Hz, 3H).

^{13}C NMR (101 MHz, cdcl$_3$) δ 142.6, 142.6, 140.0, 129.3, 128.0, 127.1, 126.8, 126.5, 65.0, 40.4, 38.0, 31.8, 29.5, 23.5, 22.7, 21.6, 16.8, 14.2, 14.2.

HRMS (ESI-, m/Z): calcd for $C_{23}H_{32}NO_2S_2$ [M-H]$^-$: 386.21483; found: 386.21518.

The enantiomeric ratio was determined by chiral HPLC analysis, Chiracel-ODH column, *n*-heptane/*i*-PrOH 97:3, 0.5ml/min, 40 °C, detection at 243 nm. Retention times: 19.6 min (minor) and 20.7 min (major).

N-(1-(3-chlorophenyl)-1-(methyldiphenylsilyl)ethyl)-4-methylbenzenesulfonamide, ***7a***

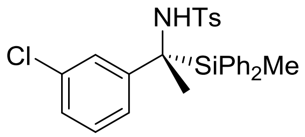

The reaction was performed with 0.1 mmol **1f**, MeMgBr (0.2 mmol, 3M in Et_2O) diluted with MTBE (1 mL total amount), CuBr·SMe_2 (1 mg, 0.005 mmol, 5 mol%), ligand(*R,S_{Fe}*)-**L8** (3.6 mg, 0.006 mmol, 6 mol%) in 2.5 mL MTBE. Product **7a** was obtained as white solid after column chromatography (SiO_2, pentane: Et_2O 80:20), [92% yield, 60% e.e., 96% e.e. of mother liquor after single crystallization from diethyl ether].

^{1}H NMR (400 MHz, $CDCl_3$) δ 7.57 – 7.45 (m, 6H), 7.41 – 7.28 (m, 6H), 7.15 – 7.07 (m, 4H), 6.85 (d, *J* = 6.4 Hz, 1H), 6.65 (s, 1H), 5.12 (s, 1H), 2.38 (s, 3H), 1.85 (s, 3H), 0.50 (s, 3H).

^{13}C NMR (101 MHz, $CDCl_3$) δ 145.2, 143.1, 139.8, 135.8, 135.6, 133.7, 131.9, 131.3, 130.6, 130.4, 129.3, 128.8, 128.2, 127.0, 125.8, 124.7, 52.7, 21.5, 21.2, -6.7.

HRMS (ESI+, m/Z): calcd for $C_{28}H_{29}ClNO_2SSi$ $[M+H]^+$: 506.1371; found:506.1366.

$[\alpha]_D$= +24.4 (c 0.18, $CHCl_3$)

The enantiomeric ratio was determined by chiral HPLC analysis, Chiralcel OZH column, n-heptane/i-PrOH 90:10, 0.5 mL/min, 40 °C, detection at 230 nm. Retention times 20.2 min (major) and 23.3 min (minor).

N-(1-(3-chlorophenyl)-1-(methyldiphenylsilyl)propyl)-4-methylbenzenesulfonamide, ***7b***

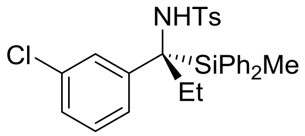

The reaction was performed with 0.1 mmol **1f**, EtMgBr (0.2 mmol, 3M in Et_2O) diluted with MTBE (1 mL total amount) , CuBr·SMe_2 (1 mg, 0.005 mmol, 5 mol%), ligand(*R,S_{Fe}*)-**L8** (3.6 mg,

0.006 mmol, 6 mol%) in 2.5 mL MTBE. Product **7b** was obtained as white solid after column chromatography (SiO_2, pentane: Et_2O 80:20), [81% yield, 84% e.e.].

^1H NMR (400 MHz, $CDCl_3$) δ 7.53 – 7.48 (m,2H), 7.46 – 7.28 (m, 10H), 7.12 (d, *J* = 8.0 Hz, 2H), 7.05 – 6.96 (m, 2H), 6.90 – 6.84 (m, 1H), 6.62 (s, 1H), 5.01 (s, 1H), 2.54 – 2.39 (m, 1H), 2.37 (s, 3H), 2.39 – 2.30 (m, 1H), 0.89 (t, *J* = 7.4 Hz, 3H), 0.61 (s, 3H).

^{13}C NMR (101 MHz, $CDCl_3$) δ 143.4, 143.1, 139.9, 135.7, 135.5, 133.5, 133.4, 133.0, 130.1, 129.4, 128.7, 128.3, 128.2, 128.1, 126.9, 126.0, 125.4, 59.4, 27.8, 21.6, 10.5, -4.9.

HRMS (ESI+, m/Z): calcd for $C_{29}H_{31}ClNO_2SSi$ $[M+H]^+$: 520.1529; found:520.1521.

The enantiomeric ratio was determined by chiral HPLC analysis, Chiralcel OZH column, n-heptane/i-PrOH 97:3, 0.5 mL/min, 40 °C, detection at 230 nm. Retention times 42.5 min (major) and 48.0 min (minor).

N-(1-(3-chlorophenyl)-1-(methyldiphenylsilyl)pentyl)-4-methylbenzenesulfonamide, ***7c***

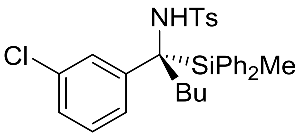

The reaction was performed with 0.1 mmol **1f**, n-BuMgBr (0.2 mmol, 2M in Et_2O) diluted with MTBE (total amount 1 mL) , CuBr·SMe_2 (1 mg, 0.005 mmol, 5 mol%), ligand(*R,S$_{Fe}$*)-**L8** (3.6 mg, 0.006 mmol, 6 mol%) in 2.5 mL MTBE. Product **7c** was obtained as colorless oil after column chromatography (SiO_2, pentane: Et_2O 80:20), [81% yield, 87% e.e.].

^1H NMR (400 MHz, $CDCl_3$) δ 7.52 – 7.46 (m, 2H), 7.45 – 7.28 (m, 10H), 7.13 (d, *J* = 7.9 Hz, 2H), 7.06 – 6.95 (m, 2H), 6.87 (d, *J* = 7.6 Hz, 1H), 6.64 (s, 1H), 5.00 (s, 1H), 2.37 (s, 3H), 2.35 – 2.14 (m, 2H), 1.35 – 1.23 (m, 2H), 1.17 – 1.02 (m, 2H), 0.70 (t, *J* = 6.9 Hz, 3H), 0.60 (s, 3H).

^{13}C NMR (101 MHz, $CDCl_3$) δ 143.7, 143.1, 134.0, 135.7, 135.5, 133.5, 133.4, 133.1, 130.1, 129.4, 128.7, 128.2, 128.1, 128.1, 126.9, 125.9, 125.4, 59.0, 35.1, 27.5, 23.2, 21.6, 13.9, -4.8.

HRMS (ESI+, m/Z): calcd for $C_{31}H_{35}ClNO_2SSi$ $[M+H]^+$: 548.1841; found:548.1834.

The enantiomeric ratio was determined by chiral HPLC analysis, Chiralcel OZH column, n-heptane/i-PrOH 96:4, 0.5 mL/min, 40 °C, detection at 230 nm. Retention times 21.6 min (major) and 22.6 min (minor).

N-(1-(3-chlorophenyl)-5-methyl-1-(methyldiphenylsilyl)hexyl)-4-methylbenzenesulfonamide, ***7d***

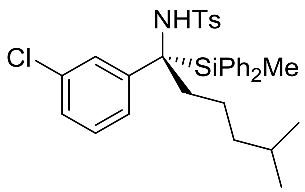

The reaction was performed with 0.1 mmol **1f**, *i*-HexMgBr (0.2 mmol, 1.5M in Et_2O) diluted with MTBE (total amount 1 mL) , CuBr·SMe_2 (1 mg, 0.005 mmol, 5 mol%), ligand(*R,S$_{Fe}$*)-**L8** (3.6 mg, 0.006 mmol, 6 mol%) in 2.5 mL MTBE. Product **7d** was obtained as colorless oil after column chromatography (SiO_2, pentane: Et_2O 80:20), [97% yield, 90% e.e.].

^{1}H NMR (400 MHz, $CDCl_3$) δ 7.51 (d, *J* = 7.3 Hz, 2H), 7.43 – 7.29 (m, 10H), 7.12 (d, *J* = 8.0 Hz, 2H), 7.05 – 7.69 (m, 2H), 6.87 (d, *J* = 7.6 Hz, 2H), 6.66 (s, 1H), 5.03 (s, 1H), 2.37 (s, 3H), 2.35 – 2.14 (m, 2H), 1.35 – 1.10 (m, 4H), 1.04 – 0.80 (m, 2H), 0.70 (d, *J* = 6.8 Hz, 3H), 0.68 (d, *J* = 6.6 Hz, 3H), 0.59 (s, 3H).

^{13}C NMR (101 MHz, $CDCl_3$) δ 143.8, 143.1, 140.0, 135.7, 135.5, 133.5, 133.5, 133.1, 130.1, 130.1, 129.4, 128.7, 128.1, 128.1, 126.9, 125.9, 125.4, 59.1, 39.4, 35.5, 27.9, 23.5, 22.8, 22.4, 21.6, -4.9.

HRMS (ESI+, m/Z): calcd for $C_{33}H_{39}ClNO_2SSi$ $[M+H]^+$: 576.2154; found:576.2147.

The enantiomeric ratio was determined by chiral HPLC analysis, Chiralcel ODH column, n-heptane/i-PrOH 98:2, 0.5 mL/min, 40 °C, detection at 230 nm. Retention times 19.6 min (minor) and 21.0 min (major).

N-(1-(3-chlorophenyl)-1-(methyldiphenylsilyl)hex-5-en-1-yl)-4-methylbenzenesulfonamide, ***7e***

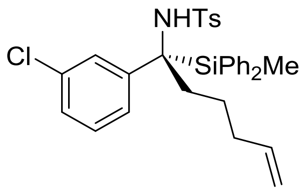

The reaction was performed with 0.1 mmol **1f**, pent-4-en-1-ylmagnesium bromide (0.2 mmol, 1.6M in Et_2O) diluted with MTBE (total amount 1 mL) , CuBr·SMe_2 (1 mg, 0.005 mmol, 5 mol%), ligand(*R,S$_{Fe}$*)-**L8** (3.6 mg, 0.006 mmol, 6 mol%) in 2.5 mL MTBE. Product **7e** was obtained as colorless oil after column chromatography (SiO_2, pentane: Et_2O 80:20), [93% yield, 86% e.e.].

^{1}H NMR (400 MHz, $CDCl_3$) δ 7.51 – 7.46 (m, 2H), 7.45 – 7.29 (m, 10H), 7.13 (d, *J* = 8.0 Hz, 2H), 7.06 – 6.94 (m, 2H), 6.80 – 6.84 (m, 1H), 6.63 (s, 1H), 5.61 – 5.50 (m, 1H), 5.01 (s, 1H), 4.91 – 4.79 (m, 1H), 2.37 (s, 3H), 2.36 – 2.12 (m, 2H), 1.90 – 1.75 (m, 2H), 1.49 – 1.27 (m, 2H), 0.60 (s, 3H).

^{13}C NMR (101 MHz, $CDCl_3$) δ 143.6, 143.1, 139.9, 138.1, 135.7, 135.5, 133.5, 133.3, 132.9, 130.2, 129.4, 128.7, 128.1, 128.1, 126.9, 126.0, 125.3, 114.9, 58.9, 34.7, 34.1, 24.6, 21.6, -4.9.

HRMS (ESI+, m/Z): calcd for $C_{32}H_{35}ClNO_2SSi$ $[M+H]^+$: 560.1841; found:560.1835.

The enantiomeric ratio was determined by chiral HPLC analysis, Chiralcel ADH column, n-heptane/i-PrOH 98:2, 0.5 mL/min, 40 °C, detection at 230 nm. Retention times 31.5 min (minor) and 42.8 min (major).

N-(1-(3-chlorophenyl)-1-(methyldiphenylsilyl)hept-6-en-1-yl)-4-methylbenzenesulfonamide, ***7f***

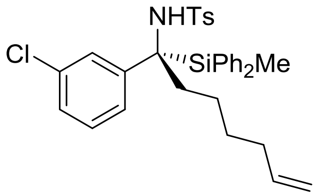

The reaction was performed with 0.1 mmol **1f**, hex-5-en-1-ylmagnesium bromide (0.2 mmol, 1.3M in Et_2O) diluted with MTBE (total amount 1 mL) , $CuBr{\cdot}SMe_2$ (1 mg, 0.005 mmol, 5 mol%), ligand(*R,S_{Fe}*)-**L8** (3.6 mg, 0.006 mmol, 6 mol%) in 2.5 mL MTBE. Product **7f** was obtained as colorless oil after column chromatography (SiO_2, pentane: Et_2O 80:20), [98% yield, 92% e.e.].

^{1}H NMR (400 MHz, $CDCl_3$) δ 7.52 – 7.46 (m, 2H), 7.43 – 7.29 (m, 10H), 7.12 (d, *J* = 8.0 Hz, 2H), 7.06 – 6.95 (m, 2H), 6.90 – 6.84 (m, 1H), 6.64 (s, 1H), 5.70 – 5.58 (m, 1H), 5.01 (s, 1H), 4.93 – 4.83 (m, 2H), 2.37 (s, 3H), 2.35 – 2.12 (m, 2H), 1.90 – 1.74 (m, 2H), 1.35 – 1.03 (m, 4H), 0.59 (s, 3H).

^{13}C NMR (101 MHz, $CDCl_3$) δ 143.7, 143.1, 139.9, 138.7, 135.7, 135.5, 133.5, 133.4, 133.0, 130.1, 129.4, 128.7, 128.1, 128.1, 126.9, 126.0, 125.3, 114.5, 59.0, 35.1, 33.5, 29.4, 25.0, 21.6, -4.9.

HRMS (ESI+, m/Z): calcd for $C_{33}H_{37}ClNO_2SSi$ $[M+H]^+$: 574.1997; found:574.1992.

The enantiomeric ratio was determined by chiral HPLC analysis, Chiralcel ADH column, n-heptane/i-PrOH 98:2, 0.5 mL/min, 40 °C, detection at 230 nm. Retention times 21.3 min (minor) and 41.7 min (major).

N-(5-chloro-1-(3-chlorophenyl)-1-(methyldiphenylsilyl)pentyl)-4-methylbenzenesulfonamide, ***7g***

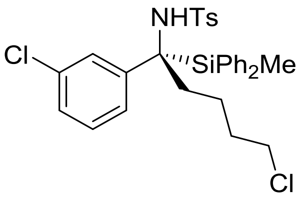

The reaction was performed with 0.1 mmol **1f**, (4-chlorobutyl)magnesium bromide (0.2 mmol, 1M in Et_2O) diluted with MTBE (total amount 1 mL), $CuBr \cdot SMe_2$ (1 mg, 0.005 mmol, 5 mol%), ligand(*R,S$_{Fe}$*)-**L8** (3.6 mg, 0.006 mmol, 6 mol%) in 2.5 mL MTBE. Product **7g** was obtained as colorless oil after column chromatography (SiO_2, pentane: Et_2O 80:20), [60% yield, 87% e.e.].

^{1}H NMR (400 MHz, $CDCl_3$) δ 7.52 – 7.46 (m, 2H), 7.46 – 7.29 (m, 10H), 7.13 (d, *J* = 8.0 Hz, 2H), 7.05 – 6.95 (m, 2H), 6.88 – 6.80 (m, 1H), 6.59 (s, 1H), 3.39 – 3.31 (m, 1H), 3.31 – 3.22 (m, 1H), 2.45 – 2.37(m, 1H), 2.37 (s, 3H), 2.28 – 2.18 (m, 1H), 1.63 – 1.37 (m, 4H), 0.60 (s, 3H).

^{13}C NMR (101 MHz, $CDCl_3$) δ 143.3, 139.8, 135.7, 135.5, 133.6, 133.0, 132.6, 130.3, 129.5, 128.7, 128.2, 128.2, 126.8, 126.1, 125.2, 58.8, 44.7, 34.7, 33.0, 23.1, 21.6, -4.9.

HRMS (ESI+, m/Z): calcd for $C_{31}H_{34}Cl_2NO_2SSi$ $[M+H]^+$: 582.1451; found:582.1446.

The enantiomeric ratio was determined by chiral HPLC analysis, Chiralcel OZH column, n-heptane/i-PrOH 96:4, 0.5 mL/min, 40 °C, detection at 230 nm. Retention times 30.3 min (minor) and 32.2 min (major).

N-(1-(dimethyl(phenyl)silyl)-1-phenylethyl)-4-methylbenzenesulfonamide, ***7h***

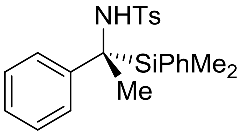

The reaction was performed with 0.1 mmol **1ab**, methylmagnesium bromide (0.2 mmol, 3M in Et_2O) diluted with MTBE (total amount 1 mL), $CuBr \cdot SMe_2$ (1 mg, 0.005 mmol, 5 mol%), ligand(*R,S$_{Fe}$*)-**L8** (3.6 mg, 0.006 mmol, 6 mol%) in 2.5 mL MTBE. Product **7h** was obtained as white solid after column chromatography (SiO_2, pentane: Et_2O 80:20), [95% yield, 10% e.e.].

^{1}H NMR (400 MHz, $CDCl_3$) δ 7.46 – 7.41 (m, 3H), 7.34 (t, *J* = 7.5, 2H), 7.25 (d, *J* = 6.6 Hz, 2H), 7.14 – 7.05 (m, 5H), 6.93 (d, *J* = 6.8 Hz, 2H), 4.96 (s, 1H), 2.34 (s, 3H), 1.69 (s, 3H), 0.29 (s, 3H), 0.16 (s, 3H).

^{13}C NMR (101 MHz, $CDCl_3$) δ 143.2, 142.7, 140.5, 135.0, 133.5, 130.3, 129.3, 128.1, 127.7, 126.9, 126.0, 125.5, 52.6, 21.6, 20.5, -5.9, -6.0.

$[\alpha]_D$= +11 (c 0.2, $CHCl_3$).

The enantiomeric ratio was determined by chiral HPLC analysis, Chiralcel OZH column, n-heptane/i-PrOH 90, 10 mL/min, 40 °C, detection at 230 nm. Retention times 24.2 min (minor) and 28.3 min (major).

Cleavage of N-Ts group

4-phenyldecan-4-amine **8**

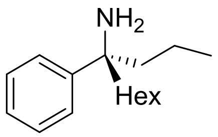

Note: Attempts to deprotect N-sulfonyl protected α-chiral silyl amines were unsuccessful due to their decomposition.

Following a literature procedure[7], gaseous NH_3 was condensed into a flask at –78 °C (ca. 5 mL). Lithium wire (21 mg, 3 mmol) was cut in small pieces and was added portionwise to the liquid NH_3. A solution of **6t** (30 mg, 0.1 mmol) in THF (0.4 ml) was then added to it, and the resulting mixture was stirred for 20 min at –78 °C. The reaction was quenched with NH_4Cl, and the flask was warmed to room temperature to evaporate off the NH_3. Then $NaHCO_3$ was added to basify the mixture and the organic phase was extracted using DCM. It was dried over $MgSO_4$ and concentrated under vacuum. to afford compound. Product **8t** was obtained as a colourless oil in 84% yield after column chromatography (SiO_2, pentane: Et_2O 20:1 ⟶0:1)

^{1}H NMR (400 MHz, Chloroform-*d*) δ 7.39 (d, *J* = 7.4 Hz, 2H), 7.32 (t, *J* = 7.6 Hz, 2H), 7.20 (t, *J* = 7.2 Hz, 1H), 1.85 – 1.50 (m, 6H), 1.27 – 1.14 (m, 8H), 1.05 – 0.92 (m, 2H), 0.83 (t, *J* = 7.2 Hz, 3H), 0.83 (t, *J* = 7.5 Hz, 3H).

^{13}C NMR (101 MHz, $cdcl_3$) δ 147.6, 128.1, 125.9, 125.8, 58.0, 46.7, 44.2, 31.9, 30.0, 23.8, 22.8, 17.1, 14.7, 14.2.

HRMS (ESI+, m/Z): calcd for $C_{16}H_{28}N$ $[M+H]^+$: 234.22163; found: 234.22150.

Supplementary references

[1] Bruker, (2012). *APEX2* (v2012.4-3), *SAINT* (Version 8.18C) and *SADABS* (Version 2012/1). Bruker AXS Inc., Madison, Wisconsin, USA.

[2] Sheldrick, G. M. SHELXT - Integrated space-group and crystal-structure determination. *Acta Cryst.* A**71**, 3-8 (2015).

[3] Sheldrick, G. M. A short history of SHELX. *Acta Cryst.* A**64**, 112-122 (2008).

[4] Craig R.S.; Mans D.J.; and RajanBabu T.V. (R)-2,2'-Binaphtoyl-(S,S)-di(1-phenylethyl) aminophosphine. Scalable protocols for the synthesis of phosphoramidite (Feringa) ligands. *Org. Synth.* **85**, 238-247 (2008).

[5] Romero A.; Woerpel K. A. Stereoselective Synthesis of Highly Substituted γ-Lactams by the [3+2] Annulation of α-Siloxy Allylic Silanes with Chlorosulfonyl Isocyanate. *Org. Lett.* **8**, 2127-2130 (2006).

[6] Kondo, Y., Sasaki, M., Kawahata, M., Yamaguchi, K. & Takeda, K. Enantioselective Synthesis of α-Silylamines by Meerwein–Ponndorf–Verley-Type Reduction of α-Silylimines by a Chiral Lithium Amide. *J. Org. Chem.* **79**, 3601-3609 (2014).

[7] Shintani, R., Takeda, M., Tsuji, T. & Hayashi, T. Rhodium-Catalyzed Asymmetric Arylation of N-Tosyl Ketimines. *J. Am. Chem. Soc.* **132***,* 13168–13169 (2010).
